# Data of electronic, reactivity, optoelectronic, linear and non-linear optical parameters of doping graphene oxide nanosheet with aluminum atom

**DOI:** 10.1016/j.dib.2022.107840

**Published:** 2022-01-19

**Authors:** Crevain Souop Tala Foadin, Fridolin Tchangnwa Nya, Alhadji Malloum, Jeanet Conradie

**Affiliations:** a Materials Science Laboratory, Department of Physics, Faculty of Science, University of Maroua, P.O. Box 814, Maroua, Cameroon; b Department of Chemistry, University of the Free State, PO Box 339, Bloemfontein 9300, South Africa

**Keywords:** Graphene oxide nanosheet, Aluminum-doping, Electronic parameters, Reactivity parameters, Optoelectronic parameters, Linear and nonlinear optical parameters

## Abstract

We have established a design to increase the absorption capacity, optoelectronic, linear and nonlinear optical properties of the graphene oxide nanosheet (GON) based on the coronene molecule [C_24_H_12_] with the help of doping, using the aluminum atom. The attachment of functional groups to the coronene surface was defined according to the Lerf-Klinowski model, based on experimental predictions [1]. Two GON structures (GON1 and GON2 with formula (C_24_H_11_)(O)(OH)COOH)) have been proposed for this purpose, and it should be noted that each of them is distinguished by a different distribution of functional groups within their honeycomb lattice. A series of substitutions of the carbon atoms of the two isomers considered GON1 and GON2 were performed with the aluminum atom, resulting in the abbreviated derivative systems GON1-Alx and GON2-Alx (*x* = 1–6), respectively to each of the GON1 and GON2 units. In this work, we provide data carried out in the gas phase, from density functional theory (DFT) methods that allowed us to understand the effects of aluminum atom doping on the circular graphene oxide nanosheets. First, we report the wavenumber data related to the IR spectrum peak characteristics computed at the B3LYP, B3LYP-D3 and ωB97XD/6–31+G(d,p) levels of theory, that allowed us to validate the designs of both proposed graphene oxide models. Then, we provide electronic, reactivity, optoelectronic, linear and nonlinear optical data parameters of both graphene oxide nanosheets and their aluminum-doped derivatives computed at the B3LYP, B3LYP-D3 and /6-31+G(d,p) levels of theory. Finally the UV-vis spectra of the investigated compounds evaluated from time-dependent (TD) B3LYP and B3LYP-D3/6-31+G(d,p) levels of theory and the HOMO & LUMO orbitals of the derivatives of graphene oxide isomers computed at the B3LYP/6-31+G(d,p) level of theory are provided. In addition, the raw data of UV-vis spectra, optoelectronic parameters, Cartesian coordinates of all studied compounds and also those of IR spectra of both studied graphene oxide models are provided as supplementary file. The data reported in this work are useful to expose some specific positions of aluminum within circular model of graphene oxide nanosheet that improve its electronic, reactive, optoelectronic, linear and nonlinear optical characteristics. All the formulas and details of calculation performed to obtain the data reported in this work are provided in our previous work (Foadin et al., 2020) and summarized in the experimental section of this paper. To learn more about the ideal doping positions of the aluminum atom within both proposed graphene oxide designs that increase their electronic, reactivity, optoelectronic, linear optical and nonlinear optical properties, respectively, please see the corresponding main research paper (Foadin et al., 2022).

## Specifications Table

**Table:** 

Subject	Chemistry
Specific subject area	Physical and Theoretical Chemistry
Type of data	Table Graph Figure
How the data were acquired	All calculation performed in this work are computed using the quantum computational chemistry program Gaussian 16 suite of programs. The electronic energies, HOMO and LUMO orbitals and data of reactivity, linear and nonlinear optical parameters provided, are extracted from Gaussian output files. The UV–Vis spectra of all studied compounds and their relative optoelectronic data parameters were simulated from calculated oscillator strengths by GaussSum 3.0 software [4].
Data format	Raw Analyzed
Description of data collection	The optimization calculations to obtain the electronic, reactivity, linear and nonlinear optical parameters; and the excited states calculations to obtain UV-vis spectra and the data of optoelectronic parameters, have been performed on the CHPC clusters (Center for High Performance Computing) in South Africa. The analysis of the obtained parameters, as well as the HOMO
	and LUMO orbitals have been performed using the facilities of our laboratory (Materials Science Laboratory of the Department of Physics, Faculty of Science, and University of Maroua).
Description of data collection	DFT calculations were performed using the resources of the Center of High Performance Computing (CHPC), South Africa
Data source location	Institution: Materials Science Unit, Department of Physic, University of Maroua City/Town/Region: Maroua Country: Cameroon
Data accessibility	With the article
Related research article	C.S.T. Foadin, F. Tchangnwa Nya, A. Malloum, J. Conradie, Enhancement of absorption capacity, optical and non-linear optical properties of graphene oxide nanosheet, J. Mol. Graph. Model. 111 (2022) 108075. https://doi.org/10.1016/j.jmgm.2021.108075 [3].

## Value of the Data

• The data reported in this work will provide new insights for the aluminum-substituent effect on the geometric structure, reactivity, optoelectronic, linear and nonlinear optical properties of graphene oxide nanosheet models based on the coronene molecule.
• The data will be useful for the researchers in engineering, chemistry and physics to propose new efficient materials which can able to replace graphene oxide nanosheet in the technological applications such as optical switching, optical limiting, saturable absorption, frequency conversion, pulse shaping, light-to-energy conversion, drug delivery process, …etc.
• These data inform us about the ideal doping positions of the aluminum atom within the coronene molecule-based graphene oxide nanosheet that enhance their intrinsic characteristics, useful for further investigations.
• The Cartesian coordinates provided as supplementary file, would be useful for further investigations on circular model of graphene oxide nanosheet and its aluminum doped-derivatives.

## 1 Data Description

**Fig. 1:**
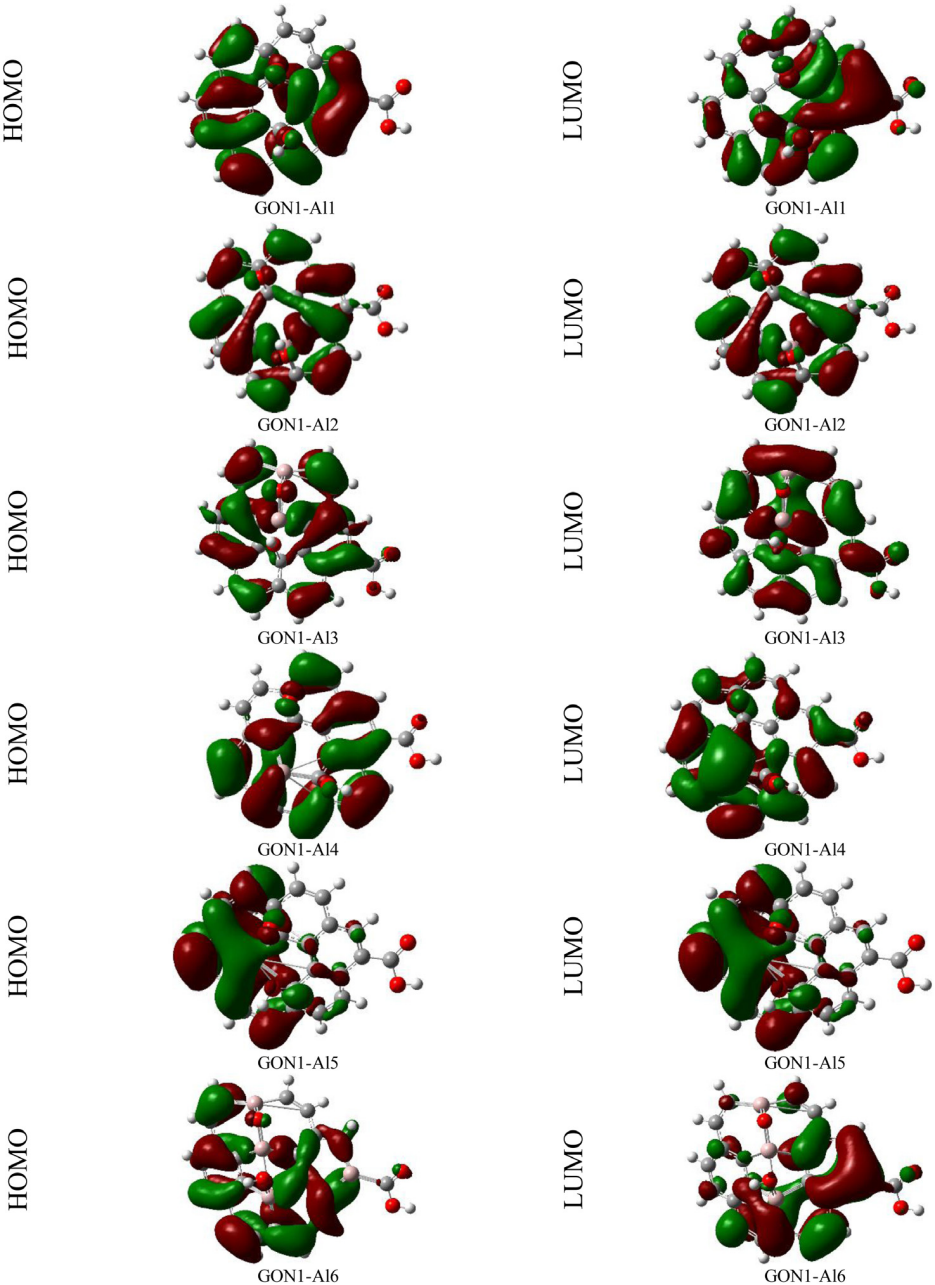
HOMO and LUMO orbitals of GON1 isomer derivatives (GON1-Alx) computed in gas phase at the B3LYP/6-31+G(d,p) level of theory.

**Fig. 2:**
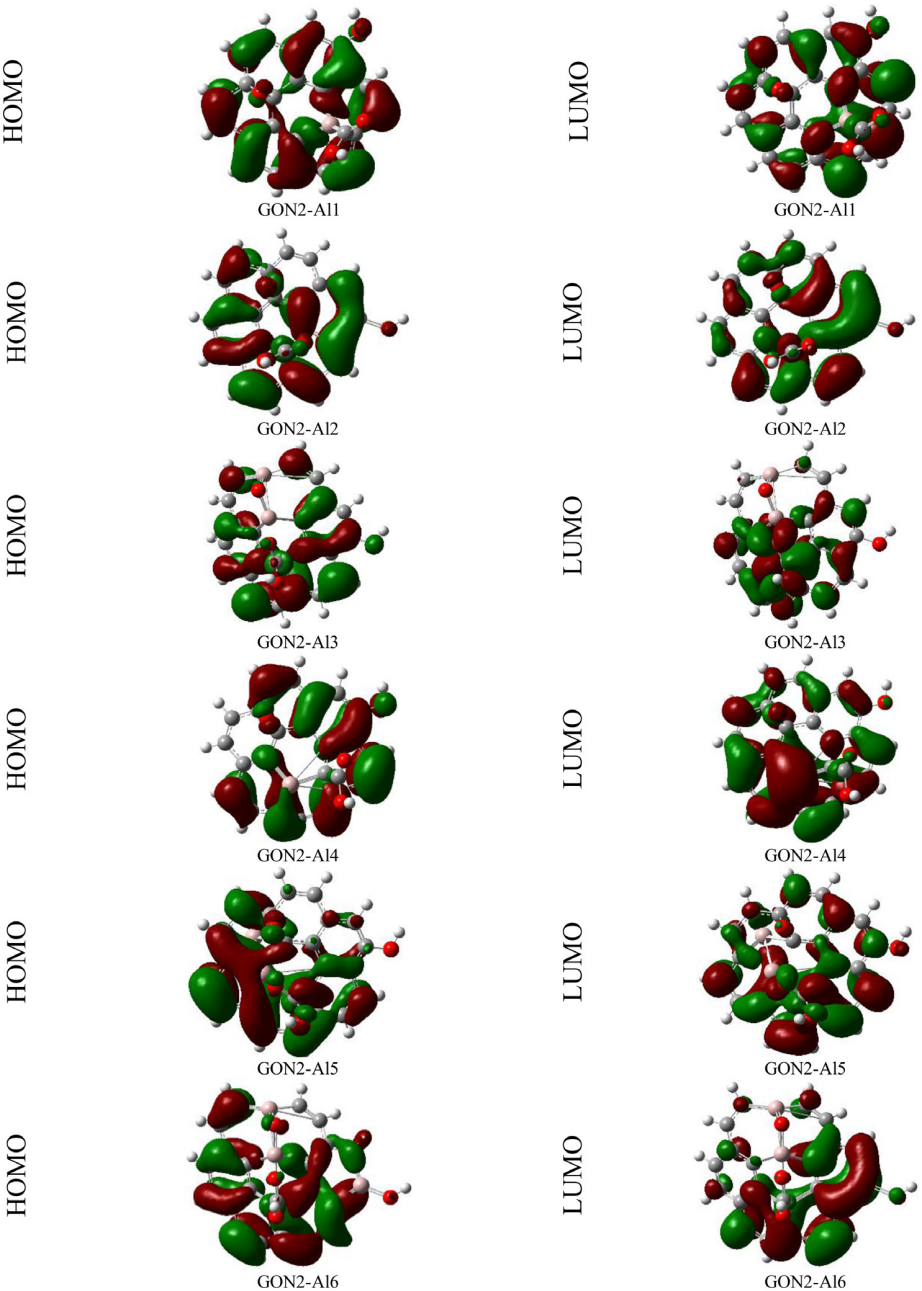
HOMO and LUMO orbitals of GON2 isomer derivatives (GON2-Alx) computed in gas phase at the B3LYP/6-31+G(d,p) level of theory.

**Fig. 3:**
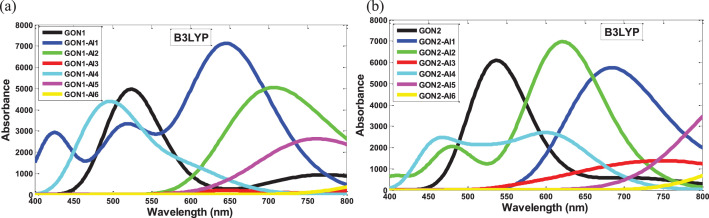
The UV-vis spectra of the graphene oxide isomers and theirs doped derivatives calculated in gas phase at the time-dependent (TD) B3LYP/6-31+G(d,p) level of theory: (a) graph of GON1 isomer and its GON1-Alx derivatives, (b) graph of GON2 isomer and its GON2-Alx derivatives.

**Fig. 4:**
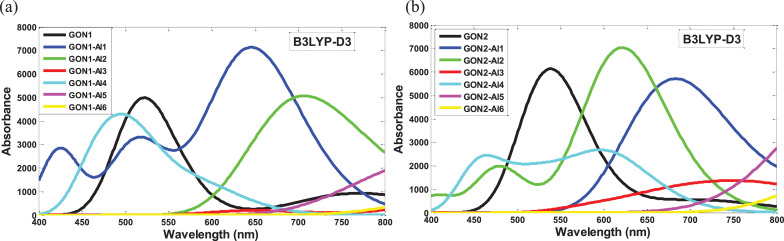
The UV-vis spectra of the graphene oxide isomers and theirs doped derivatives calculated in gas phase at the time-dependent (TD) B3LYP-D3/6-31+G(d,p) level of theory: (a) graph of GON1 isomer and its GON1-Alx derivatives, (b) graph of GON2 isomer and its GON2-Alx derivatives.

The data provided in this work were useful in understanding the effects of aluminum atom doping on circular graphene oxide model. All performed calculations were carried out in the gas phase. Figs. 1 and 2 show the HOMO and LUMO orbitals of the graphene oxide isomers derivatives (GON1-Alx and GON2-Alx) obtained from the B3LYP/6-31+G(d,p) theory level. The UV-vis spectra of the two graphene oxide isomers and their aluminum-doped derivatives evaluated at the time-dependent (TD) B3LYP and B3LYP-D3/6-31+G(d,p) level of theory are displayed in Figs. 3 and 4. Tables 1 and 2 report the wavenumbers associated to the IR spectra characteristic peaks of both proposed graphene oxide isomers computed at the B3LYP, B3LYP-D3 and ωB97XD/6-31+G(d,p) levels of theory and those associated to the IR spectra characteristic peaks reported in the literature in order to validate the structural model of both proposed graphene oxide nanosheets. Tables 3 and 4 provide the electronic energies and reactivity parameter data of both graphene oxide isomers (GON1 and GON2) and their aluminum-doped derivatives (GON1-Alx and GON2-Alx, *x* = 1–6), calculated at the B3LYP, B3LYP-D3 and ωB97XD/6-31+G(d,p) levels of theory. Tables 5 and 6 report the linear and nonlinear optical parameter data of graphene oxide nanosheets and their derivatives followed of those of *p*-nitro aniline (which is used as a benchmark compound to study the nonlinear optical properties) calculated at the B3LYP, B3LYP-D3 and ωB97XD/6-31+G(d,p) levels of theory. Table 7, Table 8, Table 9, Table 10, Table 11, Table 12 report the optoelectronic parameters data of both graphene oxide isomers and their doped derivatives, calculated at the B3LYP, B3LYP-D3 and ωB97XD/6-31+G(d,p) levels of theory. The calculation details of all reported results are described in the next section. In addition, we provide in the supplementary material the UV-vis spectra data of all studied compounds computed at the B3LYP, B3LYP-D3/6-31+G(d,p) levels of theory, the optoelectronic data parameters of all studied compounds computed at the B3LYP, B3LYP-D3 and ωB97XD /6-31+G(d,p) levels of theory, the IR data spectra of both studied graphene oxide nanosheets computed at the B3LYP, B3LYP-D3 and ωB97XD/6-31+G(d,p) levels of theory and the Cartesian coordinates of all studied structures optimized at the B3LYP, B3LYP-D3 and ωB97XD/6-31+G(d,p) levels of theory.

**Table 1:** Peak assignments of the IR spectra of GON1 isomer obtained at the B3LYP, B3LYP-D3 and ωB97XD/6-31+G(d,p) levels of theory.

Vibrational modes of GON1	Group	B3LYP	B3LYP-D3	ωB97XD	Exp
		λ (cm^−1^)	λ (cm^−1^)	λ (cm^−1^)	λ (cm^−1^)
δ(CC) + δ(-COOH) + -COOH[δoop(OH)]	–COOH, skeletal	605–672(652^a^, 616^b^)	605 −672	672	600^a^
δ(COC) + δoop(CH)	–O–, –COOH, skeletal	836–880(800–852^a^, 864^b^)	836–880	880	850^a^
–COOH[ν(C–O)] + δ(CC) + ν(C–C) + δ(CH)	–COOH, skeletal	1037–1176(1068^a^, 1128^b^)	1037–1176	1208	1070^a^
-OH[δ(COH)] + -COOH[δ(COH)] + ν(C–C) + δ(CC)	–OH, –COOH, skeletal	1376(1439–1484^a^, 1328^b^)	1376	1408	1430^a^, 1400^c^
-COOH[ν(C=O)]	–COOH	1776(1720^a^, 1816^b^)	1776	1832	1720^a^, 1750^c^, 1727^d^
ν(C–H)	skeletal	3192(3139–3232^a^, 3192^b^)	3192	3216	3100–3300^a^
ν(O–H)	–OH	3752(3658^a^, 3744^b^)	3752	3848	3450^c^, 3411^e^

When values are presented as in “605–672″, it means that multiple peaks were identified in this range associated with the same type of vibrations.

a theoretical and experimental data obtained from Ref. [5].

b theoretical data obtained from Ref. [2].

c experimental data obtained from Ref. [6].

d experimental data obtained from Ref. [7].

e experimental data obtained from Ref. [8].

**Table 2:** Peak assignments of the IR spectra of GON2 isomer obtained at the B3LYP, B3LYP-D3 and ωB97XD/6-31+G(d,p) levels of theory.

		B3LYP	B3LYP-D3	ωB97XD	Exp
Vibrational modes of GON2	Groups	λ (cm^−1^)	λ (cm^−1^)	λ (cm^−1^)	λ (cm^−1^)
δ(CC) + ν_as_(C–O-C) + -COOH[δ(OH)]	skeletal; –COOH;	605–656(652^a^, 616^b^)	605–656	656	600^a^
δ(COC) + δoop(CH)	–O–, skeletal, –COOH	836–880(800–852^a^, 864^b^)	836–880	880	850^a^
–COOH[ν(C–O)] + –OH[ν(C–O)] + δ(CH) + ν(C–C) + ν_as_(C–O-C)	–COOH, -OH, skeletal; –O–	1020–1152(1068^a^, 1128^b^)	1020–1152	1200	1070^a^
ν(C–C)	skeletal	1336(1439–1484^a^, 1328^b^)	1336	1368	1430^a^, 1400^c^
ν(C–C)	skeletal	1640(1063–1668^a^)	1640	1680	1635^c^, 1618^d^
-COOH[ν(C = O)]	–COOH	1808(1720^a^, 1816^b^)	1808	1856	1720^a^, 1750^c^, 1727^d^
ν(C–H)	skeletal	3184(3139–3232^a^, 3192^b^)	3184	3216	3100–3300^a^
ν(O–H)	–OH	3745–3832(3658^a^, 3744^b^)	3745–3832	3920	3450^c^, 3411^e^

When values are presented as in “605–656″, it means that multiple peaks were identified in this range associated with the same type of vibrations.

a theoretical and experimental data obtained from Ref. [5].

b theoretical data obtained from Ref. [2].

c experimental data obtained from Ref. [6].

d experimental data obtained from Ref. [7].

e experimental data obtained from Ref. [8].

**Table 3:** The electronic and reactivity properties of GON1 isomer and its GON1-Alx derivatives calculated in gas phase at the B3LYP, B3LYP-D3 and ωB97XD/6-31+G(d,p) levels of theory.

		Methods/basis set				Methods/basis set		
Systems	Properties	B3LYP/6-31+G(d,p)	B3LYP-D3/6-31+G(d,p)	ωB97XD/6-31+G(d,p)	Properties	B3LYP/6-31+G(d,p)	B3LYP-D3/6-31+G(d,p)	ωB97XD/6-31+G(d,p)
GON1	E_HOMO_ (eV)	‒5.23	‒5.22	‒6.98	ΔNmax (eV)	5.31	5.30	1.70
	E_LUMO_ (eV)	‒3.57	‒3.57	‒1.81	ω (eV)	11.68	11.65	3.73
	E_gap_ (eV)	1.66	1.66	5.17	VIP(eV)	6.51	5.17	6.70
	µ (eV)	‒4.40	‒4.39	‒4.39	VEA(eV)	2.33	3.67	2.13
	ɳ (eV)	0.83	0.83	2.58				

GON1-Al1	E_HOMO_ (eV)	‒5.70	‒5.70	‒7.48	ΔNmax (eV)	3.77	3.77	1.53
	E_LUMO_ (eV)	‒3.31	‒3.31	‒1.56	ω (eV)	8.50	8.50	3.44
	E_gap_ (eV)	2.39	2.39	5.92	VIP(eV)	7.00	5.68	7.26
	µ (eV)	‒4.51	‒4.50	‒4.52	VEA(eV)	2.05	3.36	1.78
	ɳ (eV)	1.19	1.19	2.96				

GON1-Al2	E_HOMO_ (eV)	‒5.98	‒5.98	‒7.80	ΔNmax (eV)	5.26	5.26	1.95
	E_LUMO_ (eV)	‒4.07	‒4.07	‒2.51	ω (eV)	13.21	13.21	5.02
	E_gap_ (eV)	1.91	1.91	5.29	VIP(eV)	7.28	5.96	7.59
	µ (eV)	‒5.02	‒5.02	‒5.15	VEA(eV)	2.83	4.15	2.85
	ɳ (eV)	0.95	0.95	2.64				

GON1-Al3	E_HOMO_ (eV)	‒6.02	‒6.00	‒7.21	ΔNmax (eV)	4.94	4.89	1.84
	E_LUMO_ (eV)	‒3.99	‒3.96	‒2.13	ω (eV)	12.36	12.18	4.29
	E_gap_ (eV)	2.03	2.04	5.08	VIP(eV)	7.33	5.95	6.93
	µ (eV)	‒5.01	‒4.98	‒4.67	VEA(eV)	2.74	4.08	2.49
	ɳ (eV)	1.01	1.02	2.54				
GON1-Al4	E_HOMO_ (eV)	‒5.87	‒5.87	‒7.76	ΔNmax (eV)	3.27	3.25	1.38
	E_LUMO_ (eV)	‒3.12	‒3.11	‒1.23	ω (eV)	7.35	7.29	3.09
	E_gap_ (eV)	2.75	2.76	6.53	VIP(eV)	7.17	5.80	7.66
	µ (eV)	‒4.49	‒4.49	‒4.49	VEA(eV)	1.85	3.21	1.39
	ɳ (eV)	1.37	1.38	3.27				

GON1-Al5	E_HOMO_ (eV)	‒4.76	‒4.77	‒6.54	ΔNmax (eV)	5.17	6.46	1.58
	E_LUMO_ (eV)	‒3.22	‒3.49	‒1.48	ω (eV)	10.31	13.35	3.17
	E_gap_ (eV)	1.54	1.28	5.06	VIP(eV)	6.08	4.50	6.25
	µ (eV)	‒3.99	‒4.13	‒4.01	VEA(eV)	1.98	3.79	1.82
	ɳ (eV)	0.77	0.64	2.53				

GON1-Al6	E_HOMO_ (eV)	‒5.47	‒5.46	‒7.26	ΔNmax (eV)	6.20	6.19	1.86
	E_LUMO_ (eV)	‒3.95	‒3.94	‒2.18	ω (eV)	14.60	14.57	4.38
	E_gap_ (eV)	1.52	1.52	5.09	VIP(eV)	6.72	5.36	7.09
	µ (eV)	‒4.71	‒4.70	‒4.72	VEA(eV)	2.68	4.03	2.59
	ɳ (eV)	0.76	0.76	2.54

**Table 4:** The electronic and reactivity properties of GON2 isomer and its GON2-Alx derivatives calculated in gas phase at the B3LYP, B3LYP-D3 and ωB97XD/6-31+G(d,p) levels of theory.

		Methods/basis set				Methods/basis set		
Systems	Properties	B3LYP/6-31+G(d,p)	B3LYPD3/6-31+G(d,p)	ωB97XD/6-31+G(d,p)	Properties	B3LYP/6-31+G(d,p)	B3LYPD3/6-31+G(d,p)	ωB97XD/6-31+G(d,p)
GON2	E_HOMO_ (eV)	‒4.77	‒4.76	‒6.51	ΔNmax (eV)	4.74	4.73	1.52
	E_LUMO_ (eV)	‒3.11	‒3.10	‒1.33	ω (eV)	9.33	9.30	2.97
	E_gap_ (eV)	1.66	1.66	5.17	VIP(eV)	6.05	4.64	6.21
	µ (eV)	‒3.94	‒3.93	‒3.92	VEA(eV)	1.88	3.27	1.65
	ɳ (eV)	0.83	0.83	2.59				

GON2-Al1	E_HOMO_ (eV)	‒5.90	‒5.90	‒7.68	ΔNmax (eV)	4.79	4.79	1.87
	E_LUMO_ (eV)	‒3.86	‒3.86	‒2.32	ω (eV)	11.68	11.67	4.67
	E_gap_ (eV)	2.04	2.04	5.35	VIP(eV)	7.18	5.88	7.46
	µ (eV)	‒4.88	‒4.88	‒5.00	VEA(eV)	2.64	3.93	2.67
	ɳ (eV)	1.02	1.02	2.68				

GON2-Al2	E_HOMO_ (eV)	‒5.42	‒5.41	‒7.19	ΔNmax (eV)	3.39	3.39	1.39
	E_LUMO_ (eV)	‒2.95	‒2.94	‒1.17	ω (eV)	7.09	7.07	2.91
	E_gap_ (eV)	2.47	2.47	6.01	VIP(eV)	6.72	5.31	6.96
	µ (eV)	‒4.18	‒4.17	‒4.18	VEA(eV)	1.67	3.06	1.41
	ɳ (eV)	1.23	1.23	3.01				

GON2-Al3	E_HOMO_ (eV)	‒4.69	‒4.68	‒6.46	ΔNmax (eV)	4.58	4.58	1.49
	E_LUMO_ (eV)	‒3.01	‒3.01	‒1.26	ω (eV)	8.82	8.81	2.87
	E_gap_ (eV)	1.68	1.68	5.20	VIP(eV)	5.93	4.49	6.15
	µ (eV)	‒3.85	‒3.84	‒3.86	VEA(eV)	1.81	3.24	1.62
	ɳ (eV)	0.84	0.84	2.60				

GON2-Al4	E_HOMO_ (eV)	‒5.60	‒5.60	‒7.45	ΔNmax (eV)	3.23	3.22	1.33
	E_LUMO_ (eV)	‒2.95	‒2.94	‒1.06	ω (eV)	6.92	6.87	2.84
	E_gap_ (eV)	2.64	2.66	6.39	VIP(eV)	6.91	5.53	7.25
	µ (eV)	‒4.28	‒4.27	‒4.26	VEA(eV)	1.68	3.06	1.25
	ɳ (eV)	1.32	1.33	3.19				

GON2-Al5	E_HOMO_ (eV)	‒4.49	‒4.49	‒6.61	ΔNmax (eV)	6.32	6.50	1.55
	E_LUMO_ (eV)	‒3.26	‒3.29	‒1.43	ω (eV)	12.25	12.64	3.13
	E_gap_ (eV)	1.23	1.20	5.18	VIP(eV)	5.77	4.30	6.30
	µ (eV)	‒3.88	‒3.89	‒4.02	VEA(eV)	2.01	3.50	1.73
	ɳ (eV)	0.61	0.60	2.59				

GON2-Al6	E_HOMO_ (eV)	‒5.28	‒5.28	‒7.04	ΔNmax (eV)	6.41	6.41	1.85
	E_LUMO_ (eV)	‒3.85	‒3.85	‒2.09	ω (eV)	14.62	14.62	4.22
	E_gap_ (eV)	1.42	1.42	4.95	VIP(eV)	6.53	5.13	6.83
	µ (eV)	‒4.56	‒4.56	‒4.57	VEA(eV)	2.60	3.98	2.49
	ɳ (eV)	0.71	0.71	2.48

**Table 5:** Dipole moment (mμ); average polarizability (<α>) and major tensor (a.u.); first hyperpolarizability order ($\beta_{\text{tot}}$) and major contributing tensor (a.u.) of GON1 isomer and its GON1-Alx derivatives calculated in gas phase at the B3LYP, B3LYP-D3 and ωB97XD/6-31+G(d,p) levels of theory.

Systems	methods	mµ (D)	$\alpha_{xx}$	$\alpha_{yy}$	$\alpha_{zz}$	<α>	$\beta_{xxx}$	$\beta_{yyy}$	$\beta_{zzz}$	$\beta_{xyy}$	$\beta_{xzz}$	$\beta_{yxx}$	$\beta_{yzz}$	$\beta_{zxx}$	$\beta_{zyy}$	$\beta_{tot}$	$\beta_{tot}\;\left(esu\right)$
GON1	B3LYP	4.08	439.61	407.12	154.73	333.82	‒89.90	‒284.34	‒247.77	179.80	101.65	‒88.53	‒327.39	‒17.54	‒279.97	907.96	7.84E-30
	B3LYP-D3	4.01	438.81	406.63	154.81	333.42	‒98.07	‒248.11	‒246.02	169.26	102.51	‒89.05	‒312.66	‒21.25	‒254.02	850.98	7.35E-30
	ωB97XD	3.85	406.95	376.05	151.60	311.54	‒98.52	‒140.47	‒143.26	90.13	7.76	‒51.30	‒186.08	3.35	‒121.53	459.48	3.97E-30

GON1-Al1	B3LYP	4.70	481.44	416.34	163.69	353.82	2878.20	262.70	129.84	552.21	117.56	514.69	40.49	52.64	58.96	3649.01	3.15E-29
	B3LYP-D3	4.68	480.73	416.30	163.42	353.48	2835.06	288.20	128.45	567.32	116.47	531.22	43.08	52.35	59.93	3631.00	3.14E-29
	ωB97XD	4.48	435.14	386.24	159.25	326.88	1266.21	226.06	129.00	363.24	74.07	286.73	23.32	37.69	59.91	1800.20	1.56E-29

GON1-Al2	B3LYP	4.49	460.24	434.38	185.42	360.01	‒1382.98	2457.03	225.87	‒88.74	35.61	1130.71	2.01	22.70	276.01	3901.79	3.37E-29
	B3LYP-D3	4.47	459.38	433.67	185.89	359.65	‒1367.20	2453.59	227.46	‒90.72	36.56	1134.16	1.27	19.24	272.75	3895.02	3.37E-29
	ωB97XD	4.41	425.86	398.88	182.50	335.75	‒1027.71	1967.10	133.25	‒145.29	12.71	1022.65	6.80	‒41.59	177.31	3224.58	2.79E-29

GON1-Al3	B3LYP	3.18	489.40	441.3	181.98	370.89	‒393.20	‒204.47	205.64	‒129.32	‒3.8	‒233.93	‒2.84	‒320.3	‒153.66	737.36	6.37E-30
	B3LYP-D3	3.83	486.49	442.81	181.37	370.23	‒651.88	‒219.60	199.89	‒174.23	28.06	‒315.61	7.16	‒198.61	‒123.78	964.73	8.33E-30
	ωB97XD	5.27	417.94	402.68	195.53	338.72	916.66	‒684.31	‒95.50	35.54	18.8	‒255.74	11.65	‒171.59	‒225.69	1430.95	1.24E-29

GON1-Al4	B3LYP	3.34	439.68	392.56	186.29	339.51	‒149.65	378.93	‒87.68	2.10	100.22	175.02	124.09	45.43	‒68.59	688.67	5.95E-30
	B3LYP-D3	3.28	438.26	391.45	186.95	338.89	‒136.61	374.72	‒94.95	‒0.56	98.54	176.39	126.59	36.64	‒72.51	691.29	5.97E-30
	ωB97XD	3.12	399.10	361.06	182.73	314.30	‒155.51	125.96	‒54.78	‒13.02	68.08	74.18	85.76	22.76	‒71.64	320.27	2.77E-30

GON1-Al5	B3LYP	2.70	479.47	439.50	219.30	379.42	‒2986.58	‒1101.49	‒730.74	‒402.64	120.32	‒163.67	144.45	220.25	229.21	3467.11	3.00E-29
	B3LYP-D3	3.36	520.44	452.87	222.18	398.50	298.49	‒342.67	770.17	816.01	410.82	‒177.66	‒89.56	475.75	215.07	2198.42	1.90E-29
	ωB97XD	2.82	437.01	412.01	214.93	354.65	1568.95	‒523.20	820.95	‒177.45	456.82	‒514.39	‒14.86	166.38	‒116.55	2298.30	1.99E-29

GON1-Al6	B3LYP	3.75	586.71	474.85	211.08	424.21	6810.28	149.13	‒71.15	1403.49	154.52	‒194.16	14.12	‒620.05	‒170.56	8412.6	7.27E-29
	B3LYP-D3	3.73	585.06	474.71	210.54	423.43	6948.05	216.90	‒91.83	1438.29	166.15	‒92.15	16.69	‒706.8	‒177.17	8609.14	7.44E-29
	ωB97XD	3.90	500.37	428.92	205.93	378.41	3360.15	805.17	‒62.50	1098.54	69.14	691.39	18.80	‒354.83	−172.92	4811.02	4.16E-29

*p*-nitro aniline (PNA)	B3LYP	7.93	…	…	…	102.79	…	…	…	…	…	…	…	…	…	1791.04	1.55E-29
	B3LYP-D3	7.93	…	…	…	102.82	…	…	…	…	…	…	…	…	…	1792.75	1.55E-29
	ωB97XD	7.54	…	…	…	97.51	…	…	…	…	…	…	…	…	…	1443.20	1.25E-29

**Table 6:** Dipole moment (mμ); average polarizability (<α>) and major tensor (a.u.); first hyperpolarizability order ($\beta_{\text{tot}}$) and major contributing tensor (a.u.) of GON2 isomer and its GON2-Alx derivatives calculated in gas phase at the B3LYP, B3LYP-D3 and ωB97XD/6-31+G(d,p) levels of theory.

Systems	methods	mµ (D)	$\alpha_{xx}$	$\alpha_{yy}$	$\alpha_{zz}$	<α>	$\beta_{xxx}$	$\beta_{yyy}$	$\beta_{zzz}$	$\beta_{xyy}$	$\beta_{xzz}$	$\beta_{yxx}$	$\beta_{yzz}$	$\beta_{zxx}$	$\beta_{zyy}$	$\beta_{tot}$	$\beta_{tot}\;\left(esu\right)$
GON2	B3LYP	2.49	401.63	402.39	170.71	324.91	‒983.98	678.21	65.69	‒159.25	‒16.34	85.48	90.67	134.79	39.15	1460.12	1.26E-29
	B3LYP-D3	2.41	401.64	402.55	170.08	324.76	–1001.70	675.40	64.57	‒160.22	‒13.73	86.80	90.64	129.73	44.75	1471.94	1.27E-29
	ωB97XD	2.42	376.03	371.20	164.96	304.06	‒442.71	522.71	110.30	‒90.50	‒19.04	78.84	64.29	112.44	63.65	911.24	7.87E-30

GON2-Al1	B3LYP	6.65	434.47	429.54	226.08	363.36	‒1944.08	1253.02	1776.16	‒385.33	686.38	‒2.12	‒388.01	731.22	138.67	3231.98	2.79E-29
	B3LYP-D3	6.65	434.28	428.97	226.01	363.09	‒1942.80	1267.94	1775.57	‒375.23	682.35	16.11	‒384.81	715.52	139.54	3225.56	2.79E-29
	ωB97XD	6.66	398.91	404.3	210.83	338.01	‒1586.91	941.14	659.13	‒639.96	168.26	81.43	‒59.27	442.28	‒29.71	2512.84	2.17E-29

GON2-Al2	B3LYP	2.48	432.13	404.14	179.51	338.59	‒736.08	‒434.89	114.89	‒338.37	‒78.20	‒500.65	‒52.26	68.45	76.97	1540.16	1.33E-29
	B3LYP-D3	2.42	432.16	404.08	179.02	338.42	‒731.34	‒436.4	114.9	‒341.04	‒78.84	‒503.16	‒51.34	74.45	77.35	1542.18	1.33E-29
	ωB97XD	2.27	398.57	375.7	173.18	315.82	‒526.18	‒382.00	124.93	‒291	‒53.57	‒369.86	‒38.55	78.28	84.72	1210.72	1.05E-29

GON2-Al	B3LYP	2.76	450.98	415.29	202.75	356.34	315.77	621.62	‒231.80	186.39	26.58	‒91.07	44.47	‒197.7	‒99.39	943.36	8.15E-30
	B3LYP-D3	2.80	451.01	415.36	202.45	356.28	325.92	620.70	‒233.25	188.95	25.33	‒94.63	45.32	‒200.06	‒99.27	949.70	8.20E-30
	ωB97XD	3.00	413.18	384.13	195.89	331.06	176.42	448.24	‒21.58	157.61	97.91	68.61	28.78	‒73.50	‒62.67	713.57	6.16E-30

GON2-Al4	B3LYP	3.19	406.19	394.78	202.5	334.49	1080.35	700.35	‒132.38	478.31	37.59	722.59	184.22	‒141.35	‒45.81	2287.59	1.98E-29
	B3LYP-D3	3.16	405.42	394.35	202.53	334.1	1065.1	688.06	‒143.78	468.30	30.49	714.47	186.09	‒150.58	‒50.18	2255.69	1.95E-29
	ωB97XD	3.13	370.90	362.02	196.16	309.69	633.32	238.28	‒91.93	196.39	‒1.09	390.99	119.42	‒108.25	‒32.10	1140.65	9.85E-30

GON2-Al5	B3LYP	1.54	500.56	446.67	241.42	396.22	‒1671.30	677.26	465.07	‒501.77	94.84	678.2	‒100.98	‒741.92	‒17.50	2445.28	2.11E-29
	B3LYP-D3	1.72	503.36	448.39	241.35	397.70	‒935.37	510.49	510.47	‒387.66	133.88	550.47	‒103.05	‒596.71	‒11.15	1530.08	1.32E-29
	ωB97XD	4.27	476.31	407.10	230.02	371.14	1562.44	1061.64	‒353.65	166.77	294.89	512.11	143.43	‒162.58	‒212.19	2752.50	2.38E-29

GON2-Al6	B3LYP	1.94	533.19	459.38	241.86	411.48	5818.77	541.67	‒512.28	1114.08	220.97	140.77	82.77	‒415.83	–44.08	7260.02	6.27E-29
	B3LYP-D3	1.92	531.68	457.48	242.19	410.45	‒1015.44	1184.08	4511.81	‒727.21	‒1427.85	401.37	741.58	705.89	813.22	7199.94	6.22E-29
	ωB97XD	2.62	464.72	416.22	232.19	371.04	2478.58	1290.67	‒252.15	1040.93	116.23	757.23	138.44	‒251.92	‒266.82	4311.95	3.73E-29

*p*-nitro aniline (PNA)	B3LYP	7.93	…	…	…	102.79	…	…	…	…	…	…	…	…	…	1791.04	1.55E-29
	B3LYP-D3	7.93	…	…	…	102.82	…	…	…	…	…	…	…	…	…	1792.75	1.55E-29
	ωB97XD	7.54	…	…	…	97.51	…	…	…	…	…	…	…	…	…	1443.20	1.25E-29

**Table 7:** Maximum transition energy (E), absorption maximum wavelength (λ), oscillator strength (*f_os_*), light harvesting efficiency (LHE) and nature of the associated electronic transitions followed by their major contribution of GON1 isomer and its GON1-Alx derivatives calculated in gas phase at the time-dependent (TD) ωB97XD/6-31+G(d,p) level of theory.

Systems	E (eV)	λ_max_ (nm)	*f_os_*	LHE	MO transition
GON1	2.83	438.35	0.10	0.20	H-1(α) →L+1(α) (10%), HOMO(α)→LUMO(α) (51%), HOMO(β)→LUMO(β) (13%)
GON1-Al1	2.51	494.32	0.15	0.29	HOMO→LUMO (94%)
GON1-Al2	2.24	553.18	0.09	0.19	H-2→LUMO (38%), H-1→LUMO (36%), HOMO→LUMO (15%)
GON1-Al3	1.64	754.39	0.03	0.06	HOMO(β) →LUMO(β) (78%)
GON1-Al4	3.18	389.78	0.08	0.17	H-2→LUMO (25%), H-1→LUMO (41%), HOMO→LUMO (19%)
GON1-Al5	1.90	654.03	0.02	0.05	HOMO(β) →LUMO(β) (79%)
GON1-Al6	1.80	687.73	0.03	0.06	H-1(α)→LUMO(α) (10%), HOMO(β)→LUMO(β) (33%)

**Table 8:** Maximum transition energy (E), absorption maximum wavelength (λ), oscillator strength (*f_os_*), light harvesting efficiency (LHE) and the associated electronic transitions followed by their major contribution of GON2 isomer and its $\text{GON}2-Al_{X}$ derivatives calculated in gas phase at the time-dependent (TD) ωB97XD/6-31+G(d,p) level of theory.

Systems	E (eV)	λ_max_ (nm)	*f_os_*	LHE	MO transition
GON2	2.75	451.64	0.10	0.21	HOMO(α) →L+1(α) (56%), HOMO(β) →LUMO(β) (27%)
GON2-Al1	2.30	538.83	0.10	0.20	H-1→LUMO (72%), HOMO→LUMO (17%)
GON2-Al2	2.53	490.13	0.13	0.26	HOMO→LUMO (95%)
GON2-Al3	2.48	499.39	0.04	0.08	HOMO(α) →LUMO(α) (72%), HOMO(β) →LUMO(β) (15%)
GON2-Al4	3.56	348.35	0.07	0.15	H-2→LUMO (65%)
GON2-Al5	2.06	601.81	0.04	0.08	HOMO(α) →LUMO(α) (18%), HOMO(β) →LUMO(β) (53%)
GON2-Al6	1.81	684.84	0.04	0.08	H-2(β) →LUMO(β) (13%), HOMO(β) →LUMO(β) (46%)

**Table 9:** Maximum transition energy (E), absorption maximum wavelength (λ), oscillator strength (*f_os_*), light harvesting efficiency (LHE) and the associated electronic transitions followed by their major contribution of GON1 isomer and its GON1-Alx derivatives calculated in gas phase at the time-dependent (TD) B3LYP/6-31+G(d,p) level of theory.

Systems	E (eV)	λ_max_ (nm)	*f_os_*	LHE	MO transition
GON1	2.38	521.31	0.07	0.14	HOMO(α) →LUMO(α) (71%), H-1(β) →LUMO(β) (10%)
GON1-Al1	1.92	645.35	0.10	0.20	HOMO→LUMO (96%)
GON1-Al2	1.77	700.91	0.07	0.14	H-2→LUMO (55%), H-1→LUMO (34%)
GON1-Al3	1.93	644.34	0.00	0.01	HOMO(α) →LUMO(α) (34%), HOMO(β) →L+1(β) (30%)
GON1-Al4	2.42	513.60	0.04	0.09	H-1→LUMO (88%)
GON1-Al5	1.66	748.47	0.02	0.05	HOMO(α) →LUMO(α) (86%), HOMO(β) →LUMO(β) (10%)
GON1-Al6	…	…	…	...	…

**Table 10:** Maximum transition energy (E), absorption maximum wavelength (λ), oscillator strength (*f_os_*), light harvesting efficiency (LHE) and the associated electronic transitions followed by their major contribution of GON2 isomer and its GON2-Alx derivatives calculated in gas phase at the time-dependent (TD) B3LYP/6-31+G(d,p) level of theory.

Systems	E (eV)	λ_max_ (nm)	*f_os_*	LHE	MO transition
GON2	2.33	533.40	0.08	0.16	HOMO(α)→L+1(α) (77%), HOMO(β)→LUMO(β) (17%)
GON2-Al1	1.83	678.44	0.07	0.15	H-1→LUMO (75%), HOMO→LUMO (14%)
GON2-Al2	2.00	620.70	0.10	0.20	HOMO→LUMO (98%)
GON2-Al3	1.57	789.61	0.01	0.03	HOMO(β)→LUMO(β) (83%)
GON2-Al4	2.04	609.05	0.03	0.08	HOMO→LUMO (97%)
GON2-Al5	1.66	746.40	0.00	0.01	H-1(β)→LUMO(β) (86%)
GON2-Al6	…	…	…	...	…

**Table 11:** Maximum transition energy (E), absorption maximum wavelength (λ), oscillator strength (*f_os_*), light harvesting efficiency (LHE) and the associated electronic transitions followed by their major contribution of GON1 isomer and its GON1-Alx derivatives calculated in gas phase at the time-dependent (TD) B3LYP-D3/6-31+G(d,p) level of theory.

Systems	E (eV)	λ_max_ (nm)	*f_os_*	LHE	MO transition
GON1	2.39	519.98	0.07	0.14	HOMO(α)→LUMO(α) (71%)
GON1-Al1	1.92	645.62	0.1	0.2	HOMO→LUMO (96%)
GON1-Al2	1.77	701.19	0.07	0.14	H-2→LUMO (45%), H-1→LUMO (45%)
GON1-Al3	1.91	648.52	0	0.01	H-1(α)→LUMO(α) (13%), HOMO(α)→LUMO(α) (36%), HOMO(β)→L+1(β) (31%)
GON1-Al4	2.42	512.56	0.04	0.09	H-1→LUMO (88%)
GON1-Al5	1.58	784.96	0.01	0.02	H-1(β)→LUMO(β) (87%)
GON1-Al6	…	…	…	...	…

**Table 12:** Maximum transition energy (E), absorption maximum wavelength (λ), oscillator strength (*f_os_*), light harvesting efficiency (LHE) and the associated electronic transitions followed by their major contribution of GON2 isomer and its GON2-Alx derivatives calculated in gas phase at the time-dependent (TD) B3LYP-D3/6-31+G(d,p) level of theory.

Systems	E (eV)	λ_max_ (nm)	*f_os_*	LHE	MO transition
GON2	2.32	534.81	0.08	0.16	HOMO(α)→L+1(α) (76%), HOMO(β)→LUMO(β) (18%)
GON2-Al1	1.83	677.44	0.07	0.15	H-1→LUMO (75%), HOMO→LUMO (14%)
GON2-Al2	2.00	620.48	0.10	0.20	HOMO→LUMO (98%)
GON2-Al3	1.57	789.86	0.01	0.03	HOMO(β)→LUMO(β) (83%)
GON2-Al4	2.05	605.86	0.03	0.07	HOMO→LUMO (97%)
GON2-Al5	1.65	750.01	0.00	0.01	H-1(β)→LUMO(β) (87%)
GON2-Al6	…	…	…	...	…

## 2 Experimental Design, Materials and Methods

All theoretical calculations achieved on all studied compounds were performed with the Gaussian 16 suite of programs, as also described in our previous work [2,3]. The structures modeled have been fully optimized in the gas phase using the B3LYP, B3LYP-D3 and ωB97XD/6-31+G(d,p) levels of theory. Then, we have carried out the frequency calculations on the optimized B3LYP, B3LYP-D3 and ωB97XD structures in order to confirm the true minimum energy optimization. The excitation states of all studied compounds were performed using time-dependent (TD) calculations at the levels of theory used. The UV–Vis spectra of studied compounds and their optoelectronic parameter were simulated from calculated oscillator strengths by GaussSum 3.0 software [4]. Grimme dispersion correction term (D3) associated with the B3LYP-D3 functional was implemented by adding the key option *"iop(3/124=30)*" to the B3LYP hybrid functional. The HOMO and LUMO orbitals, generated from the FCHK files (Gaussian Formatted Checkpoint Files) of Gaussian output files were analyzed to confirm the push-pull models of both studied graphene oxide isomers. Note that we have not provided the FCHK files due to their size. The peak assignment data of the IR spectra, provided as supplementary material, were compared with those of theoretical [2,5] and experimental [6], [7], [8] IR spectra of graphene oxide nanosheets found in the literature in order to validate the structural model of both proposed graphene oxide nanosheets. The frontier molecular orbital energies such as LUMO energy ($E_{\text{LUMO}}$) and HOMO energy ($E_{\text{HOMO}}$) were found from output files of optimized geometries of the studied compounds. The others electronic energies and reactivity parameters such as HOMO-LUMO gap energy ($E_{\text{gap}}$), chemical potential(μ), global hardness (η), maximum amount of electronic charge index (ΔNmax), global electrophilicity (ω), vertical ionization potential (VIP) and vertical electron affinity (VEA)) were computed from the formulas found in our preview work [2]. The data of dipole moment (mμ) and major tensor components of polarizability ($\alpha_{\text{xx}}$, $\alpha_{\text{yy}}$ and $\alpha_{\text{zz}}$) and first hyperpolarizability order ($\beta_{\text{xxx}}$, $\beta_{\text{yyy}}$, $\beta_{\text{zzz}}$, $\beta_{\text{xyy}}$, $\beta_{\text{xzz}}$, $\beta_{\text{yxx}}$, $\beta_{\text{yzz}}$, $\beta_{\text{zxx}}$ and $\beta_{zyy}$) were obtained from output files of optimization calculation by adding the key option *"polar"* to the keyword calculation code. The average polarizability (<α>) and first hyperpolarizability order ($\beta_{\text{tot}}$) were calculated using the formulas found in our previous work [2]. Maximum transition energy (E), absorption maximum wavelength (λ), oscillator strength (*f_os_*), light-harvesting efficiency (LHE) and nature of the associated electronic transitions followed by their major contribution were acquired from data optoelectronic parameters files provide as supplementary file.

## Supporting Information

The raw data of UV-vis spectra of all studied compounds computed at the time-dependent (TD) B3LYP, B3LYP-D3/6-31+G(d,p) levels of theory, optoelectronic parameters of all studied compounds computed at the B3LYP, B3LYP-D3 and ωB97XD/6-31+G(d,p) levels of theory, IR spectra of both graphene oxide isomers computed at the B3LYP, B3LYP-D3 and ωB97XD/6-31+G(d,p) levels of theory and Cartesian coordinates of all studied structures optimized at the B3LYP, B3LYP-D3 and ωB97XD/6-31+G(d,p) levels of theory are provided as supplementary file.

## CRediT authorship contribution statement

**Crevain Souop Tala Foadin:** Conceptualization, Methodology, Validation, Investigation, Data curation, Formal analysis, Writing – original draft. **Fridolin Tchangnwa Nya:** Conceptualization, Investigation, Data curation, Formal analysis, Writing – review & editing, Supervision. **Alhadji Malloum:** Investigation, Data curation, Formal analysis, Writing – review & editing. **Jeanet Conradie:** Writing – review & editing.

## Declaration of Competing Interest

The authors declare that they have no known competing financial interests or personal relationships which have, or could be perceived to have, influenced the work reported in this article.

## Appendix Supplementary materials

Image, application 1
