# Supplementary material for: Data of electronic, reactivity, optoelectronic, linear and non-linear optical parameters of doping graphene oxide nanosheet with aluminum atom
Source: Data Brief. 2022 Jan 19;41:107840. doi: 10.1016/j.dib.2022.107840 (PMC8801356; doi:10.1016/j.dib.2022.107840)
Supplement: Supplementary file 1 [file mmc1.zip › supplementary file/Cartesian coordinates/Cartesian coordinates OF GON1 AND ITS DERIVATIVES (GON1-Alx) B3LYP.rtf]

 Cartesian coordinates of GON1
 ---------------------------------------------------------------------
 Center     Atomic      Atomic             Coordinates (Angstroms)
 Number     Number       Type             X           Y           Z
 ---------------------------------------------------------------------
      1          6           0       -3.803559    1.952464   -0.412036
      2          6           0       -3.407684    0.559055   -0.283380
      3          6           0       -2.061076    0.225693    0.004945
      4          6           0       -1.086136    1.299453    0.309957
      5          6           0       -1.537744    2.741123    0.159323
      6          6           0       -2.935252    2.974914   -0.263859
      7          6           0       -1.631025   -1.098722   -0.007651
      8          6           0        0.354355    0.986544    0.119058
      9          6           0        0.792751   -0.335452    0.115763
     10          6           0       -0.212204   -1.437374    0.424200
     11          6           0        2.173288   -0.655496   -0.031050
     12          6           0        2.627070    1.742741   -0.194842
     13          6           0        1.269416    2.051574   -0.070334
     14          6           0        0.799285    3.421025   -0.212914
     15          6           0       -0.507152    3.751375   -0.161538
     16          1           0       -0.834576    4.768750   -0.358049
     17          1           0        1.545918    4.182891   -0.421065
     18          1           0       -4.833426    2.154131   -0.696097
     19          1           0       -3.242129    3.999302   -0.457488
     20          1           0        3.350877    2.538994   -0.332274
     21          6           0       -4.321034   -0.485488   -0.526044
     22          6           0       -3.903894   -1.806847   -0.523325
     23          6           0       -2.552789   -2.140540   -0.282976
     24          1           0       -5.359364   -0.243931   -0.736861
     25          1           0       -4.615517   -2.602585   -0.727152
     26          6           0       -2.088512   -3.502741   -0.348504
     27          6           0       -0.744965   -3.804353   -0.271408
     28          6           0        0.229892   -2.805445   -0.056547
     29          1           0       -2.814830   -4.289268   -0.532839
     30          1           0       -0.411912   -4.827957   -0.425932
     31          6           0        2.548384   -2.046188   -0.116777
     32          1           0        3.593378   -2.299043   -0.211092
     33          6           0        1.607727   -3.054269   -0.166862
     34          1           0        1.939308   -4.075401   -0.341075
     35          6           0        4.582792    0.320806   -0.281884
     36          8           0        5.296925    1.223087   -0.681884
     37          8           0        5.123952   -0.856541    0.127437
     38          1           0        6.084841   -0.762697    0.010308
     39          8           0       -1.390426    2.108900    1.464808
     40          8           0       -0.197029   -1.482324    1.899517
     41          1           0       -0.804067   -2.184002    2.181551
     42          6           0        3.097087    0.428788   -0.159808
 ---------------------------------------------------------------------                       

Cartesian coordinates of GON1-Al1 
--------------------------------------------------------------------
enter     Atomic      Atomic             Coordinates (Angstroms)
umber     Number       Type             X           Y           Z
--------------------------------------------------------------------
    1          6           0       -4.057041    1.956977   -0.401791
    2          6           0       -3.636126    0.568191   -0.326698
    3          6           0       -2.282320    0.234326   -0.064171
    4          6           0       -1.280951    1.299553    0.230252
    5          6           0       -1.782965    2.716673    0.155543
    6          6           0       -3.195424    2.969651   -0.209924
    7          6           0       -1.882189   -1.096464   -0.046415
    8          6           0        0.188490    0.983290    0.033754
    9          6           0        0.577064   -0.347522    0.064943
   10          6           0       -0.470278   -1.430914    0.400984
   11          6           0        1.959776   -0.802346   -0.044799
   12          6           0        2.473145    2.162005   -0.175353
   13          6           0        1.096174    2.170902   -0.127316
   14          6           0        0.488545    3.505381   -0.267204
   15          6           0       -0.816120    3.781243   -0.162120
   16          1           0       -1.190278    4.792552   -0.298199
   17          1           0        1.187311    4.311430   -0.471695
   18          1           0       -5.096812    2.156115   -0.648509
   19          1           0       -3.508617    4.004472   -0.318852
   20          1           0        2.928896    3.145157   -0.281373
   21          6           0       -4.563066   -0.462888   -0.551500
   22          6           0       -4.167175   -1.790697   -0.522717
   23          6           0       -2.826165   -2.128670   -0.271670
   24          1           0       -5.599948   -0.207068   -0.751957
   25          1           0       -4.890847   -2.582344   -0.697398
   26          6           0       -2.402461   -3.514576   -0.254967
   27          6           0       -1.092082   -3.849407   -0.148576
   28          6           0       -0.066548   -2.847413   -0.018081
   29          1           0       -3.159549   -4.283436   -0.383432
   30          1           0       -0.781045   -4.889037   -0.211623
   31          6           0        2.250449   -2.148345   -0.113651
   32          1           0        3.289767   -2.463495   -0.184389
   33          6           0        1.251195   -3.165577   -0.154875
   34          1           0        1.542195   -4.198601   -0.330994
   35          6           0        5.360313    0.143367   -0.185282
   36          8           0        6.211211    1.014637   -0.185983
   37          8           0        5.713903   -1.179333   -0.235473
   38          1           0        6.690256   -1.223804   -0.265227
   39          8           0       -1.573384    2.055949    1.438599
   40          8           0       -0.460616   -1.432550    1.870886
   41          1           0       -1.104337   -2.090309    2.176214
   42         13           0        3.370666    0.493716   -0.112847
--------------------------------------------------------------------

Cartesian coordinates of GON1-Al2                          
 ---------------------------------------------------------------------
 Center     Atomic      Atomic             Coordinates (Angstroms)
 Number     Number       Type             X           Y           Z 
 ---------------------------------------------------------------------
      1          6           0       -3.767498    2.010373   -0.664425
      2          6           0       -3.365863    0.616370   -0.565867
      3          6           0       -2.138465    0.254261    0.063072
      4          6           0       -1.148607    1.341660    0.486822
      5          6           0       -1.604582    2.765932    0.284037
      6          6           0       -2.959684    3.012809   -0.271574
      7          6           0       -1.813340   -1.091406    0.197091
      8          6           0        0.359871    1.093474    0.354137
      9          6           0        0.900129   -0.185538    0.481932
     10          6           0        2.221348   -0.491192    0.059445
     11          6           0        2.572288    1.898346   -0.248333
     12          6           0        1.218007    2.171495   -0.000023
     13          6           0        0.714355    3.530883   -0.122789
     14          6           0       -0.593933    3.816508    0.004122
     15          1           0       -0.955346    4.831947   -0.138543
     16          1           0        1.428760    4.319338   -0.345567
     17          1           0       -4.743053    2.230975   -1.091008
     18          1           0       -3.263982    4.048927   -0.398833
     19          1           0        3.244676    2.715727   -0.487627
     20          6           0       -4.176479   -0.399083   -1.116637
     21          6           0       -3.750955   -1.723593   -1.151338
     22          6           0       -2.535163   -2.082669   -0.521687
     23          1           0       -5.120970   -0.122748   -1.577701
     24          1           0       -4.341955   -2.467021   -1.682180
     25          6           0       -1.940325   -3.384171   -0.805596
     26          6           0       -0.612565   -3.818147   -0.675245
     27          6           0        0.381539   -3.032625   -0.047883
     28          1           0       -2.583009   -4.048802   -1.384649
     29          1           0       -0.358129   -4.730752   -1.223178
     30          6           0        2.573420   -1.889224   -0.241917
     31          1           0        3.588856   -2.030218   -0.594496
     32          6           0        1.765942   -3.024064   -0.360036
     33          1           0        2.239737   -3.880509   -0.849960
     34          6           0        4.549926    0.522496   -0.585080
     35          8           0        5.172613    1.405954   -1.144292
     36          8           0        5.170608   -0.612563   -0.166401
     37          1           0        6.105555   -0.519550   -0.417678
     38          8           0       -1.553339    2.166443    1.611795
     39          8           0       -0.228145   -2.068967    2.871768
     40          1           0       -0.480635   -1.468784    3.578412
     41         13           0       -0.223855   -1.620898    1.188130
     42          6           0        3.093254    0.598140   -0.254839
 ---------------------------------------------------------------------

Cartesian coordinates of GON1-Al3 
 ---------------------------------------------------------------------
 Center     Atomic      Atomic             Coordinates (Angstroms)
 Number     Number       Type             X           Y           Z
 ---------------------------------------------------------------------
      1          6           0        4.038484   -1.027018   -0.985400
      2          6           0        3.289692    0.203740   -0.645860
      3          6           0        1.978649    0.323526   -0.144907
      4          6           0        3.697801   -2.334002   -0.876726
      5          6           0        1.267069    1.554628   -0.166735
      6          6           0       -0.448123   -0.843835    0.093846
      7          6           0       -1.016391    0.460233    0.012852
      8          6           0       -2.442465    0.615519   -0.065582
      9          6           0       -2.587933   -1.787033   -0.463089
     10          6           0       -1.192815   -1.976034   -0.289888
     11          6           0       -0.722092   -3.354562   -0.566474
     12          6           0        0.522728   -3.888926   -0.580459
     13          1           0        0.546459   -4.934646   -0.892962
     14          1           0       -1.552843   -3.999356   -0.860807
     15          1           0        5.002794   -0.787202   -1.439073
     16          1           0        4.450263   -3.017757   -1.274318
     17          1           0       -3.207158   -2.641656   -0.715685
     18          6           0        3.983021    1.423806   -0.896664
     19          6           0        3.382998    2.650478   -0.719057
     20          6           0        2.016040    2.757482   -0.371742
     21          1           0        5.014194    1.373051   -1.236117
     22          1           0        3.949083    3.561232   -0.896033
     23          6           0        1.371688    4.028080   -0.277175
     24          6           0        0.026136    4.104793   -0.070908
     25          6           0       -0.772517    2.921052    0.021365
     26          1           0        1.969624    4.927609   -0.395877
     27          1           0       -0.471045    5.069107   -0.015894
     28          6           0       -2.999640    1.931659    0.002700
     29          1           0       -4.072893    2.052739   -0.013581
     30          6           0       -2.193897    3.031065    0.069189
     31          1           0       -2.634906    4.023225    0.110531
     32          6           0       -4.710819   -0.634072   -0.382788
     33          8           0       -5.312935   -1.518983   -0.962208
     34          8           0       -5.378714    0.352825    0.268654
     35          1           0       -6.324951    0.166723    0.141730
     36          8           0        1.915753   -2.262344    1.618258
     37          8           0        0.766131    0.261691    2.804429
     38          1           0        1.016379   -0.022935    3.688517
     39          6           0       -3.219778   -0.568386   -0.279599
     40          6           0       -0.171594    1.643342    0.001748
     41         13           0        2.095077   -2.935792    0.052202
     42         13           0        1.121513   -0.744890    1.444306
 ---------------------------------------------------------------------

Cartesian coordinates of GON1-Al4 
 ---------------------------------------------------------------------
 Center     Atomic      Atomic             Coordinates (Angstroms)
 Number     Number       Type             X           Y           Z
 ---------------------------------------------------------------------
      1          6           0       -3.768384    2.017817   -0.573571
      2          6           0       -3.421547    0.655489   -0.166873
      3          6           0       -2.175987    0.415822    0.366529
      4          6           0       -1.105769    1.422565    0.486057
      5          6           0       -2.878973    3.029764   -0.467329
      6          6           0        0.359557    1.003222    0.305421
      7          6           0        0.880462   -0.324866    0.331923
      8          6           0        2.246146   -0.592355    0.002959
      9          6           0        2.630879    1.787985   -0.178916
     10          6           0        1.269090    2.060132   -0.018137
     11          6           0        0.831004    3.415600   -0.322833
     12          6           0       -0.462299    3.781127   -0.374145
     13          1           0       -0.758114    4.759277   -0.744515
     14          1           0        1.605492    4.120895   -0.611860
     15          1           0       -4.738734    2.202080   -1.029001
     16          1           0       -3.122363    4.011307   -0.868293
     17          1           0        3.317194    2.605487   -0.372479
     18          6           0       -4.377956   -0.433881   -0.486230
     19          6           0       -4.151247   -1.785320   -0.559542
     20          6           0       -2.881672   -2.415637   -0.194104
     21          1           0       -5.361980   -0.095072   -0.805768
     22          1           0       -4.970810   -2.402671   -0.933124
     23          6           0       -2.097194   -3.217093   -0.976928
     24          6           0       -0.647328   -3.471591   -0.834471
     25          6           0        0.336608   -2.746371   -0.205279
     26          1           0       -2.502832   -3.631201   -1.907839
     27          1           0       -0.287066   -4.279525   -1.471798
     28          6           0        2.643365   -1.979173   -0.287275
     29          1           0        3.686573   -2.165601   -0.498840
     30          6           0        1.745085   -2.985660   -0.465901
     31          1           0        2.077306   -3.943272   -0.861112
     32          6           0        4.621759    0.411030   -0.361942
     33          8           0        5.294317    1.300455   -0.850078
     34          8           0        5.203442   -0.726493    0.103189
     35          1           0        6.157002   -0.626964   -0.060305
     36          8           0       -1.364484    2.456222    1.461864
     37          8           0        0.617206   -2.009328    2.097869
     38          1           0        1.557926   -2.242192    2.033981
     39          6           0        3.142984    0.493119   -0.163661
     40          6           0        0.133992   -1.594308    0.785277
     41         13           0       -1.756806   -1.330164    0.870739
     42          6           0       -1.510070    2.854706    0.077073
 ---------------------------------------------------------------------

Cartesian coordinates of GON1-Al5
 ---------------------------------------------------------------------
 Center     Atomic      Atomic             Coordinates (Angstroms)
 Number     Number       Type             X           Y           Z
 ---------------------------------------------------------------------
      1          6           0       -3.769433    1.806590   -0.200131
      2          6           0       -3.547473    0.411558   -0.286698
      3          6           0       -0.733426    1.073320    0.643037
      4          6           0       -2.687565    2.727136   -0.158027
      5          6           0        0.735722    0.889617    0.609063
      6          6           0        1.217808   -0.450961    0.543368
      7          6           0        2.528113   -0.742044    0.042097
      8          6           0        2.953439    1.677684    0.086213
      9          6           0        1.594294    1.957799    0.364326
     10          6           0        1.032634    3.300941    0.230246
     11          6           0       -0.297295    3.542902    0.212365
     12          1           0       -0.660130    4.547904    0.011404
     13          1           0        1.727837    4.120248    0.065123
     14          1           0       -4.751166    2.241694   -0.418405
     15          1           0       -2.937106    3.782123   -0.239246
     16          1           0        3.653977    2.492779   -0.064753
     17          6           0       -4.450378   -0.681922   -0.561626
     18          6           0       -3.935822   -2.002020   -0.532649
     19          6           0       -2.576627   -2.223587   -0.192155
     20          1           0       -5.506062   -0.529924   -0.797301
     21          1           0       -4.606539   -2.828178   -0.780595
     22          6           0       -1.947955   -3.500285   -0.120971
     23          6           0       -0.597225   -3.772274    0.092539
     24          6           0        0.494989   -2.804835    0.207196
     25          1           0       -2.566871   -4.386367   -0.290779
     26          1           0       -0.290564   -4.813495    0.020544
     27          6           0        2.788331   -2.093858   -0.348996
     28          1           0        3.764363   -2.355937   -0.729752
     29          6           0        1.796221   -3.051228   -0.333653
     30          1           0        2.001435   -4.034975   -0.747085
     31          6           0        4.868009    0.301259   -0.463218
     32          8           0        5.563336    1.266359   -0.725296
     33          8           0        5.398646   -0.950140   -0.453972
     34          1           0        6.335889   -0.843774   -0.690237
     35          8           0       -1.284918    1.878477    1.746434
     36          8           0       -0.716087   -1.353966    1.721508
     37          1           0       -1.044572   -2.238743    1.969012
     38          6           0        3.421994    0.381657   -0.099559
     39          6           0        0.348423   -1.556033    0.799110
     40         13           0       -1.919092   -0.461277    0.331308
     41          6           0       -1.312346    2.474920    0.430757
     42         13           0       -2.262056    1.393799   -1.976932
 ---------------------------------------------------------------------

Cartesian coordinates of GON1-Al6
 ---------------------------------------------------------------------
 Center     Atomic      Atomic             Coordinates (Angstroms)
 Number     Number       Type             X           Y           Z
 ---------------------------------------------------------------------
      1          6           0        4.564227   -1.320194   -0.636701
      2          6           0        3.828617   -0.043913   -0.501725
      3          6           0        4.141793   -2.594451   -0.427285
      4          6           0       -0.608844   -0.925695    0.231062
      5          6           0       -1.188615    0.302548    0.304268
      6          6           0       -2.477975    0.903405    0.157541
      7          6           0       -2.579031   -2.120744   -0.634755
      8          6           0       -1.209089   -2.079311   -0.518541
      9          6           0       -0.430383   -3.193273   -1.122877
     10          6           0        0.858574   -3.610496   -1.018859
     11          1           0        1.061139   -4.456900   -1.683563
     12          1           0       -1.072272   -3.757914   -1.803708
     13          1           0        5.588055   -1.176092   -0.993518
     14          1           0        4.899691   -3.353332   -0.630449
     15          1           0       -2.991007   -3.033500   -1.069695
     16          6           0        4.451741    1.101750   -1.061941
     17          6           0        3.853892    2.360012   -1.084658
     18          6           0        2.565782    2.538346   -0.532733
     19          1           0        5.434642    0.986854   -1.514329
     20          1           0        4.380592    3.194545   -1.544080
     21          6           0        1.873371    3.827187   -0.616297
     22          6           0        0.567838    4.174078   -0.304011
     23          6           0       -0.435746    3.287999    0.231816
     24          1           0        2.456471    4.617450   -1.089699
     25          1           0        0.254244    5.171544   -0.624846
     26          6           0       -2.713907    2.281797    0.019995
     27          1           0       -3.749227    2.558417   -0.177705
     28          6           0       -1.791851    3.385365   -0.030912
     29          1           0       -2.212621    4.313653   -0.431367
     30          6           0       -5.698922   -0.581700   -0.268631
     31          8           0       -6.404456   -1.511153    0.081132
     32          8           0       -6.248009    0.578441   -0.741655
     33          1           0       -7.220383    0.473514   -0.733384
     34          8           0        1.772923   -2.486095    1.695113
     35          8           0        0.642296    0.489480    2.400196
     36          1           0        1.314050    0.798750    3.025379
     37          6           0        2.557912    0.126841    0.125615
     38          6           0        1.965644    1.405459    0.072615
     39         13           0        2.325251   -3.016498    0.141901
     40         13           0        1.206958   -0.919136    1.255235
     41         13           0        0.238581    1.593779    0.888099
     42         13           0       -3.679681   -0.600640   -0.170253
 ---------------------------------------------------------------------
