# Supplementary material for: Data of electronic, reactivity, optoelectronic, linear and non-linear optical parameters of doping graphene oxide nanosheet with aluminum atom
Source: Data Brief. 2022 Jan 19;41:107840. doi: 10.1016/j.dib.2022.107840 (PMC8801356; doi:10.1016/j.dib.2022.107840)
Supplement: Supplementary file 1 [file mmc1.zip › supplementary file/Cartesian coordinates/Cartesian coordinates OF GON1 AND ITS DERIVATIVES (GON1-Alx) B3LYP-D3.rtf]

Cartesian coordinates of GON1
 ---------------------------------------------------------------------
 Center     Atomic      Atomic             Coordinates (Angstroms)
 Number     Number       Type             X           Y           Z
 ---------------------------------------------------------------------
      1          6           0       -3.805201    1.950131   -0.403334
      2          6           0       -3.407558    0.557092   -0.278055
      3          6           0       -2.059316    0.224309    0.000876
      4          6           0       -1.085200    1.297178    0.306761
      5          6           0       -1.538900    2.738563    0.162488
      6          6           0       -2.937104    2.972914   -0.257107
      7          6           0       -1.628325   -1.098454   -0.016967
      8          6           0        0.354238    0.987056    0.111751
      9          6           0        0.793355   -0.333510    0.105266
     10          6           0       -0.209494   -1.435498    0.411583
     11          6           0        2.173553   -0.651966   -0.037350
     12          6           0        2.625671    1.746720   -0.199114
     13          6           0        1.267372    2.053651   -0.074730
     14          6           0        0.795366    3.422574   -0.214377
     15          6           0       -0.511306    3.751062   -0.159737
     16          1           0       -0.842036    4.767608   -0.354110
     17          1           0        1.540398    4.185853   -0.423237
     18          1           0       -4.836195    2.151352   -0.683629
     19          1           0       -3.243605    3.997694   -0.448350
     20          1           0        3.347410    2.545232   -0.335512
     21          6           0       -4.321473   -0.488364   -0.514860
     22          6           0       -3.903427   -1.809623   -0.513119
     23          6           0       -2.550451   -2.141705   -0.281567
     24          1           0       -5.361395   -0.247972   -0.718940
     25          1           0       -4.616054   -2.606160   -0.710039
     26          6           0       -2.085261   -3.503515   -0.342842
     27          6           0       -0.740973   -3.803616   -0.272843
     28          6           0        0.233720   -2.803121   -0.068499
     29          1           0       -2.812055   -4.291836   -0.517233
     30          1           0       -0.408303   -4.827902   -0.423353
     31          6           0        2.550555   -2.041574   -0.126725
     32          1           0        3.595912   -2.293368   -0.220699
     33          6           0        1.611292   -3.050609   -0.178997
     34          1           0        1.943957   -4.071318   -0.353365
     35          6           0        4.581351    0.319422   -0.277795
     36          8           0        5.296627    1.197251   -0.726681
     37          8           0        5.115601   -0.833929    0.203588
     38          1           0        6.077671   -0.756453    0.086452
     39          8           0       -1.387211    2.101478    1.465483
     40          8           0       -0.195425   -1.480924    1.885957
     41          1           0       -0.810898   -2.176402    2.164892
     42          6           0        3.095595    0.432995   -0.162317
 ---------------------------------------------------------------------

Cartesian coordinates of GON1-Al1
 ---------------------------------------------------------------------
 Center     Atomic      Atomic             Coordinates (Angstroms)
 Number     Number       Type             X           Y           Z
 ---------------------------------------------------------------------
      1          6           0       -4.072066    1.925285   -0.400962
      2          6           0       -3.637078    0.540785   -0.329991
      3          6           0       -2.279548    0.219851   -0.073167
      4          6           0       -1.289199    1.293114    0.225432
      5          6           0       -1.805974    2.704921    0.158144
      6          6           0       -3.220672    2.945872   -0.206555
      7          6           0       -1.866850   -1.105954   -0.057606
      8          6           0        0.182408    0.993087    0.027688
      9          6           0        0.582793   -0.333388    0.056916
     10          6           0       -0.453517   -1.424879    0.392107
     11          6           0        1.968716   -0.774913   -0.047653
     12          6           0        2.456148    2.195644   -0.171265
     13          6           0        1.078403    2.190422   -0.126894
     14          6           0        0.456456    3.518768   -0.263526
     15          6           0       -0.850832    3.780557   -0.156809
     16          1           0       -1.235908    4.788096   -0.289508
     17          1           0        1.144743    4.334251   -0.466654
     18          1           0       -5.113949    2.115228   -0.646119
     19          1           0       -3.543852    3.977905   -0.311367
     20          1           0        2.904902    3.182624   -0.272537
     21          6           0       -4.554578   -0.499650   -0.551064
     22          6           0       -4.146123   -1.823738   -0.520219
     23          6           0       -2.801109   -2.148027   -0.272820
     24          1           0       -5.594793   -0.254877   -0.747943
     25          1           0       -4.863456   -2.622420   -0.688768
     26          6           0       -2.364209   -3.529405   -0.245165
     27          6           0       -1.050679   -3.851366   -0.138341
     28          6           0       -0.034563   -2.839231   -0.018379
     29          1           0       -3.114242   -4.306711   -0.363727
     30          1           0       -0.731311   -4.889021   -0.191322
     31          6           0        2.275252   -2.117624   -0.110062
     32          1           0        3.318734   -2.420386   -0.175591
     33          6           0        1.286449   -3.145029   -0.149047
     34          1           0        1.588844   -4.176154   -0.317003
     35          6           0        5.346606    0.153093   -0.186173
     36          8           0        6.224285    0.997076   -0.198624
     37          8           0        5.656897   -1.183202   -0.224304
     38          1           0        6.630647   -1.264034   -0.256919
     39          8           0       -1.588882    2.039479    1.437960
     40          8           0       -0.449631   -1.420640    1.861175
     41          1           0       -1.098634   -2.074259    2.164054
     42         13           0        3.363810    0.533760   -0.111830
 ---------------------------------------------------------------------

Cartesian coordinates of GON1-Al2
 ---------------------------------------------------------------------
 Center     Atomic      Atomic             Coordinates (Angstroms)
 Number     Number       Type             X           Y           Z
 ---------------------------------------------------------------------
      1          6           0       -3.770065    1.999206   -0.665117
      2          6           0       -3.363428    0.606585   -0.568850
      3          6           0       -2.137311    0.247104    0.062607
      4          6           0       -1.151490    1.336813    0.487597
      5          6           0       -1.612122    2.759409    0.288437
      6          6           0       -2.967502    3.003650   -0.267562
      7          6           0       -1.809782   -1.097238    0.199229
      8          6           0        0.356701    1.093661    0.351794
      9          6           0        0.899966   -0.183050    0.480117
     10          6           0        2.221167   -0.485591    0.056698
     11          6           0        2.564541    1.904763   -0.256310
     12          6           0        1.210146    2.173970   -0.004880
     13          6           0        0.702321    3.531812   -0.125638
     14          6           0       -0.605978    3.813825    0.006589
     15          1           0       -0.971230    4.828191   -0.132878
     16          1           0        1.413600    4.322578   -0.350438
     17          1           0       -4.745666    2.217218   -1.093009
     18          1           0       -3.275389    4.039008   -0.391160
     19          1           0        3.233684    2.724207   -0.498257
     20          6           0       -4.169209   -0.410470   -1.123655
     21          6           0       -3.739743   -1.733703   -1.157772
     22          6           0       -2.525933   -2.090261   -0.522837
     23          1           0       -5.112929   -0.136933   -1.587913
     24          1           0       -4.326575   -2.477772   -1.692301
     25          6           0       -1.926286   -3.389558   -0.806224
     26          6           0       -0.597660   -3.818884   -0.669946
     27          6           0        0.390912   -3.031356   -0.037201
     28          1           0       -2.563572   -4.055463   -1.389779
     29          1           0       -0.334191   -4.727854   -1.219607
     30          6           0        2.577292   -1.882006   -0.246803
     31          1           0        3.589917   -2.019683   -0.608720
     32          6           0        1.773308   -3.019543   -0.357178
     33          1           0        2.243072   -3.875567   -0.851562
     34          6           0        4.544512    0.527691   -0.589534
     35          8           0        5.161332    1.391490   -1.184696
     36          8           0        5.167811   -0.587058   -0.121882
     37          1           0        6.103155   -0.507401   -0.375276
     38          8           0       -1.557136    2.157317    1.615180
     39          8           0       -0.225152   -2.045036    2.877377
     40          1           0       -0.477786   -1.431993    3.572867
     41         13           0       -0.220410   -1.615963    1.191533
     42          6           0        3.088042    0.605718   -0.260182
 ---------------------------------------------------------------------

Cartesian coordinates of GON1-Al3
 ---------------------------------------------------------------------
 Center     Atomic      Atomic             Coordinates (Angstroms)
 Number     Number       Type             X           Y           Z
 ---------------------------------------------------------------------
      1          6           0        3.992560   -1.025192   -1.106940
      2          6           0        3.263475    0.205436   -0.728847
      3          6           0        1.967176    0.319398   -0.195377
      4          6           0        3.656747   -2.331605   -0.975844
      5          6           0        1.257450    1.549083   -0.166046
      6          6           0       -0.446214   -0.852688    0.121985
      7          6           0       -1.017751    0.449440    0.045950
      8          6           0       -2.443372    0.607559   -0.024589
      9          6           0       -2.597706   -1.802434   -0.377446
     10          6           0       -1.200924   -1.989969   -0.223857
     11          6           0       -0.734498   -3.374191   -0.477828
     12          6           0        0.511899   -3.903759   -0.522917
     13          1           0        0.528344   -4.958461   -0.804997
     14          1           0       -1.571242   -4.031918   -0.722835
     15          1           0        4.933631   -0.788148   -1.608750
     16          1           0        4.387163   -3.017060   -1.410440
     17          1           0       -3.221534   -2.664280   -0.590920
     18          6           0        3.952923    1.429804   -0.970893
     19          6           0        3.360688    2.652975   -0.745822
     20          6           0        2.004495    2.755047   -0.356156
     21          1           0        4.974464    1.387222   -1.339666
     22          1           0        3.924838    3.566448   -0.914743
     23          6           0        1.365913    4.022599   -0.202136
     24          6           0        0.027394    4.094032    0.050061
     25          6           0       -0.770916    2.909115    0.128107
     26          1           0        1.961289    4.925122   -0.310487
     27          1           0       -0.464405    5.057150    0.153463
     28          6           0       -2.996889    1.918186    0.136352
     29          1           0       -4.069171    2.041331    0.158193
     30          6           0       -2.189688    3.013870    0.236798
     31          1           0       -2.629880    4.000465    0.352785
     32          6           0       -4.712886   -0.636462   -0.387584
     33          8           0       -5.363696   -1.655947   -0.248569
     34          8           0       -5.315079    0.530239   -0.736878
     35          1           0       -6.264416    0.333291   -0.810905
     36          8           0        1.976676   -2.239337    1.590752
     37          8           0        0.822538    0.296501    2.764007
     38          1           0        1.105092    0.042416    3.647430
     39          6           0       -3.224025   -0.574050   -0.241172
     40          6           0       -0.174243    1.633414    0.045502
     41         13           0        2.102610   -2.934460    0.029741
     42         13           0        1.153750   -0.739607    1.422534
 --------------------------------------------------------------------- 

 Cartesian coordinates of GON1-Al4
 ---------------------------------------------------------------------
 Center     Atomic      Atomic             Coordinates (Angstroms)
 Number     Number       Type             X           Y           Z
 ---------------------------------------------------------------------
      1          6           0       -3.768981    2.009659   -0.572947
      2          6           0       -3.420421    0.648490   -0.164331
      3          6           0       -2.174685    0.409253    0.368817
      4          6           0       -1.106590    1.418571    0.485765
      5          6           0       -2.882432    3.023406   -0.466283
      6          6           0        0.358362    1.003231    0.301726
      7          6           0        0.880024   -0.322798    0.326569
      8          6           0        2.245350   -0.588165   -0.003121
      9          6           0        2.627737    1.792521   -0.182559
     10          6           0        1.265382    2.062237   -0.021873
     11          6           0        0.824018    3.416755   -0.325803
     12          6           0       -0.470054    3.778852   -0.374316
     13          1           0       -0.770197    4.756104   -0.743128
     14          1           0        1.595230    4.125093   -0.616343
     15          1           0       -4.739426    2.191523   -1.029189
     16          1           0       -3.126643    4.004908   -0.866432
     17          1           0        3.313539    2.610932   -0.375118
     18          6           0       -4.377522   -0.440295   -0.481397
     19          6           0       -4.148749   -1.791155   -0.550670
     20          6           0       -2.877763   -2.418029   -0.183822
     21          1           0       -5.361862   -0.102880   -0.801417
     22          1           0       -4.964889   -2.413274   -0.923373
     23          6           0       -2.093780   -3.203736   -0.982160
     24          6           0       -0.644105   -3.460627   -0.848201
     25          6           0        0.339705   -2.741381   -0.214237
     26          1           0       -2.503836   -3.599120   -1.919130
     27          1           0       -0.285561   -4.262368   -1.494286
     28          6           0        2.644855   -1.972788   -0.299392
     29          1           0        3.687562   -2.157958   -0.514432
     30          6           0        1.747628   -2.979341   -0.479111
     31          1           0        2.078875   -3.935628   -0.877999
     32          6           0        4.618955    0.411077   -0.359570
     33          8           0        5.292339    1.280302   -0.881427
     34          8           0        5.194743   -0.707670    0.156784
     35          1           0        6.149755   -0.623413   -0.005225
     36          8           0       -1.366381    2.450341    1.463812
     37          8           0        0.632466   -2.008493    2.091984
     38          1           0        1.575986   -2.228760    2.027498
     39          6           0        3.139920    0.497926   -0.166749
     40          6           0        0.138807   -1.594128    0.783448
     41         13           0       -1.752639   -1.333369    0.882795
     42          6           0       -1.513922    2.849264    0.079052
 ---------------------------------------------------------------------

Cartesian coordinates of GON1-Al5
 ---------------------------------------------------------------------
 Center     Atomic      Atomic             Coordinates (Angstroms)
 Number     Number       Type             X           Y           Z
 ---------------------------------------------------------------------
      1          6           0       -3.442450    2.117506   -0.130177
      2          6           0       -3.261360    0.687142   -0.312004
      3          6           0       -0.560892    1.548135    0.923122
      4          6           0       -2.435213    3.033863    0.130672
      5          6           0        0.764549    1.062905    0.623184
      6          6           0        1.063558   -0.349523    0.530600
      7          6           0        2.345645   -0.754428    0.084076
      8          6           0        3.045852    1.588012   -0.058970
      9          6           0        1.724486    2.024473    0.197511
     10          6           0        1.319537    3.370220   -0.049719
     11          6           0       -0.013979    3.705767   -0.050945
     12          1           0       -0.328099    4.694444   -0.375896
     13          1           0        2.071643    4.098292   -0.338393
     14          1           0       -4.435784    2.539163   -0.302810
     15          1           0       -2.708772    4.086239    0.076710
     16          1           0        3.817869    2.317106   -0.283192
     17          6           0       -4.333235   -0.202796   -0.599405
     18          6           0       -4.119130   -1.606211   -0.586236
     19          6           0       -2.892483   -2.075514   -0.071634
     20          1           0       -5.322961    0.178363   -0.865347
     21          1           0       -4.874390   -2.265017   -1.020324
     22          6           0       -2.292356   -3.313104   -0.357875
     23          6           0       -0.906061   -3.569170   -0.315706
     24          6           0        0.161508   -2.655238   -0.094823
     25          1           0       -2.898586   -4.117938   -0.783924
     26          1           0       -0.598018   -4.557684   -0.652014
     27          6           0        2.534448   -2.118701   -0.342059
     28          1           0        3.523837   -2.437158   -0.637602
     29          6           0        1.488133   -2.995822   -0.480305
     30          1           0        1.672694   -3.979746   -0.905159
     31          6           0        4.819671   -0.031803   -0.331635
     32          8           0        5.599685    0.760030   -0.829804
     33          8           0        5.242101   -1.249060    0.104987
     34          1           0        6.195336   -1.289259   -0.081500
     35          8           0       -1.376929    0.831528    1.676781
     36          8           0       -0.693561   -1.582343    1.905771
     37          1           0       -0.770570   -2.545683    2.013196
     38          6           0        3.375042    0.248159   -0.081876
     39          6           0       -0.025376   -1.359332    0.548867
     40         13           0       -1.935093   -0.571135    0.709027
     41          6           0       -1.014051    2.760552    0.337538
     42         13           0       -1.879495    1.071680   -1.922783
 ---------------------------------------------------------------------

Cartesian coordinates of GON1-Al6
 ---------------------------------------------------------------------
 Center     Atomic      Atomic             Coordinates (Angstroms)
 Number     Number       Type             X           Y           Z
 ---------------------------------------------------------------------
      1          6           0        4.570399   -1.279240   -0.637377
      2          6           0        3.820589   -0.010360   -0.511356
      3          6           0        4.162890   -2.556753   -0.417679
      4          6           0       -0.592578   -0.940595    0.242663
      5          6           0       -1.184201    0.281375    0.316679
      6          6           0       -2.483298    0.863348    0.174764
      7          6           0       -2.556029   -2.163851   -0.605384
      8          6           0       -1.185298   -2.102240   -0.500641
      9          6           0       -0.393772   -3.200510   -1.116655
     10          6           0        0.899620   -3.603478   -1.012597
     11          1           0        1.116367   -4.439432   -1.686057
     12          1           0       -1.026508   -3.764983   -1.806321
     13          1           0        5.592612   -1.127559   -0.996137
     14          1           0        4.930028   -3.307905   -0.615919
     15          1           0       -2.960946   -3.080657   -1.038582
     16          6           0        4.424002    1.137582   -1.088600
     17          6           0        3.809803    2.387821   -1.118005
     18          6           0        2.525433    2.555535   -0.554412
     19          1           0        5.404030    1.032420   -1.549593
     20          1           0        4.321054    3.224276   -1.591141
     21          6           0        1.816611    3.835189   -0.643159
     22          6           0        0.510122    4.167680   -0.320112
     23          6           0       -0.476343    3.273070    0.233300
     24          1           0        2.384678    4.627993   -1.130413
     25          1           0        0.177553    5.156928   -0.647292
     26          6           0       -2.741953    2.237037    0.033494
     27          1           0       -3.782950    2.495119   -0.160075
     28          6           0       -1.834009    3.351913   -0.026738
     29          1           0       -2.264675    4.272508   -0.434452
     30          6           0       -5.683425   -0.604889   -0.301367
     31          8           0       -6.420001   -1.567278   -0.183911
     32          8           0       -6.188948    0.646269   -0.542364
     33          1           0       -7.162578    0.573455   -0.595627
     34          8           0        1.799234   -2.467755    1.704066
     35          8           0        0.653781    0.498685    2.406035
     36          1           0        1.339800    0.819833    3.009529
     37          6           0        2.553235    0.150119    0.125125
     38          6           0        1.945596    1.421136    0.066948
     39         13           0        2.353733   -2.999110    0.154262
     40         13           0        1.222013   -0.907513    1.262271
     41         13           0        0.225206    1.592644    0.895799
     42         13           0       -3.667519   -0.652229   -0.147862
 ---------------------------------------------------------------------
