# Supplementary material for: Data of electronic, reactivity, optoelectronic, linear and non-linear optical parameters of doping graphene oxide nanosheet with aluminum atom
Source: Data Brief. 2022 Jan 19;41:107840. doi: 10.1016/j.dib.2022.107840 (PMC8801356; doi:10.1016/j.dib.2022.107840)
Supplement: Supplementary file 1 [file mmc1.zip › supplementary file/Cartesian coordinates/Cartesian coordinates OF GON1 AND ITS DERIVATIVES (GON1-Alx) wB97XD.rtf]

 Cartesian coordinates of GON1
 ---------------------------------------------------------------------
 Center     Atomic      Atomic             Coordinates (Angstroms)
 Number     Number       Type             X           Y           Z
 ---------------------------------------------------------------------
      1          6           0       -3.797962    1.937854   -0.409043
      2          6           0       -3.392169    0.542578   -0.287987
      3          6           0       -2.054641    0.218494    0.003707
      4          6           0       -1.087765    1.293884    0.318888
      5          6           0       -1.543017    2.724157    0.177650
      6          6           0       -2.939349    2.956388   -0.249920
      7          6           0       -1.618950   -1.101419   -0.010978
      8          6           0        0.351377    0.988185    0.122482
      9          6           0        0.792460   -0.330500    0.119554
     10          6           0       -0.205002   -1.432411    0.432825
     11          6           0        2.164121   -0.640878   -0.028055
     12          6           0        2.609248    1.747307   -0.204423
     13          6           0        1.255642    2.048407   -0.069353
     14          6           0        0.780047    3.420170   -0.205979
     15          6           0       -0.520286    3.741466   -0.145298
     16          1           0       -0.854230    4.757377   -0.335529
     17          1           0        1.522257    4.184489   -0.418305
     18          1           0       -4.826728    2.135148   -0.698065
     19          1           0       -3.249383    3.980761   -0.435713
     20          1           0        3.329162    2.545959   -0.351178
     21          6           0       -4.296179   -0.498946   -0.542739
     22          6           0       -3.873822   -1.815186   -0.540922
     23          6           0       -2.529058   -2.139050   -0.290326
     24          1           0       -5.333753   -0.261759   -0.760096
     25          1           0       -4.580033   -2.613357   -0.751800
     26          6           0       -2.060496   -3.504965   -0.348735
     27          6           0       -0.723591   -3.798017   -0.268345
     28          6           0        0.243494   -2.791649   -0.054191
     29          1           0       -2.784895   -4.293242   -0.530556
     30          1           0       -0.383587   -4.819793   -0.416024
     31          6           0        2.552708   -2.033023   -0.109051
     32          1           0        3.600354   -2.278456   -0.198492
     33          6           0        1.621612   -3.039040   -0.160505
     34          1           0        1.955063   -4.059792   -0.329642
     35          6           0        4.561269    0.324808   -0.295967
     36          8           0        5.260977    1.188423   -0.780253
     37          8           0        5.102238   -0.798917    0.214506
     38          1           0        6.058124   -0.720193    0.085727
     39          8           0       -1.393880    2.091148    1.460409
     40          8           0       -0.197520   -1.474344    1.886852
     41          1           0       -0.826237   -2.150356    2.167383
     42          6           0        3.076866    0.438508   -0.166594
 ---------------------------------------------------------------------

 Cartesian coordinates of GON1-Al1
 ---------------------------------------------------------------------
 Center     Atomic      Atomic             Coordinates (Angstroms)
 Number     Number       Type             X           Y           Z
 ---------------------------------------------------------------------
      1          6           0       -4.052189    1.946274   -0.386665
      2          6           0       -3.625919    0.556106   -0.305805
      3          6           0       -2.278002    0.229872   -0.055486
      4          6           0       -1.278532    1.296341    0.229694
      5          6           0       -1.780398    2.703811    0.152031
      6          6           0       -3.193633    2.954568   -0.208335
      7          6           0       -1.875203   -1.096945   -0.036182
      8          6           0        0.185703    0.978306    0.018133
      9          6           0        0.569841   -0.338866    0.049741
     10          6           0       -0.460767   -1.424103    0.400877
     11          6           0        1.960173   -0.790175   -0.068178
     12          6           0        2.462134    2.171797   -0.170786
     13          6           0        1.094553    2.168876   -0.146552
     14          6           0        0.476783    3.499556   -0.306801
     15          6           0       -0.821469    3.768337   -0.189658
     16          1           0       -1.201080    4.775695   -0.335249
     17          1           0        1.167735    4.306096   -0.533140
     18          1           0       -5.092473    2.142124   -0.631432
     19          1           0       -3.507110    3.987842   -0.325992
     20          1           0        2.913903    3.156402   -0.276855
     21          6           0       -4.549764   -0.472099   -0.517666
     22          6           0       -4.150934   -1.795687   -0.485036
     23          6           0       -2.812970   -2.124818   -0.244794
     24          1           0       -5.588733   -0.220484   -0.710477
     25          1           0       -4.874279   -2.589557   -0.648211
     26          6           0       -2.387383   -3.516947   -0.220412
     27          6           0       -1.085781   -3.844005   -0.140662
     28          6           0       -0.056620   -2.827924   -0.044356
     29          1           0       -3.147812   -4.286369   -0.318448
     30          1           0       -0.769673   -4.882228   -0.190261
     31          6           0        2.251379   -2.118798   -0.173266
     32          1           0        3.289847   -2.435137   -0.251618
     33          6           0        1.243141   -3.140022   -0.227332
     34          1           0        1.531861   -4.167327   -0.433848
     35          6           0        5.338112    0.132097   -0.148451
     36          8           0        6.196516    0.989145   -0.097154
     37          8           0        5.674921   -1.181549   -0.245137
     38          1           0        6.645624   -1.235846   -0.257982
     39          8           0       -1.558243    2.054134    1.416407
     40          8           0       -0.436599   -1.431936    1.850172
     41          1           0       -1.086453   -2.074758    2.158778
     42         13           0        3.359413    0.507068   -0.098760
 ---------------------------------------------------------------------

 Cartesian coordinates of GON1-Al2
 ---------------------------------------------------------------------
 Center     Atomic      Atomic             Coordinates (Angstroms)
 Number     Number       Type             X           Y           Z
 ---------------------------------------------------------------------
      1          6           0       -3.768932    1.959934   -0.682596
      2          6           0       -3.338091    0.568629   -0.597556
      3          6           0       -2.131686    0.225978    0.058056
      4          6           0       -1.170530    1.323082    0.507691
      5          6           0       -1.645845    2.731344    0.320815
      6          6           0       -2.995990    2.963947   -0.252619
      7          6           0       -1.787169   -1.113287    0.190448
      8          6           0        0.337659    1.098525    0.374534
      9          6           0        0.895222   -0.171632    0.505837
     10          6           0        2.209658   -0.453425    0.079967
     11          6           0        2.520078    1.925969   -0.257720
     12          6           0        1.171650    2.178350    0.012258
     13          6           0        0.648661    3.535931   -0.099317
     14          6           0       -0.654275    3.800647    0.044937
     15          1           0       -1.031288    4.812044   -0.081112
     16          1           0        1.351148    4.333967   -0.323245
     17          1           0       -4.739942    2.166367   -1.124844
     18          1           0       -3.320675    3.995612   -0.359626
     19          1           0        3.176026    2.750755   -0.517887
     20          6           0       -4.109091   -0.446417   -1.187163
     21          6           0       -3.659210   -1.759490   -1.222940
     22          6           0       -2.469835   -2.101481   -0.552287
     23          1           0       -5.041202   -0.181204   -1.678023
     24          1           0       -4.216894   -2.505556   -1.784440
     25          6           0       -1.851165   -3.398017   -0.825816
     26          6           0       -0.534184   -3.819938   -0.664684
     27          6           0        0.431077   -3.013870   -0.021095
     28          1           0       -2.475127   -4.067585   -1.419699
     29          1           0       -0.252906   -4.731229   -1.197819
     30          6           0        2.596120   -1.853835   -0.200206
     31          1           0        3.620101   -1.977284   -0.536413
     32          6           0        1.823167   -2.997664   -0.306779
     33          1           0        2.313898   -3.859409   -0.765575
     34          6           0        4.501663    0.571963   -0.625388
     35          8           0        5.082340    1.425414   -1.259049
     36          8           0        5.154890   -0.505272   -0.142376
     37          1           0        6.077705   -0.412307   -0.418749
     38          8           0       -1.592043    2.122964    1.622978
     39          8           0       -0.226075   -2.072827    2.884018
     40          1           0       -0.479817   -1.487242    3.596036
     41         13           0       -0.212440   -1.611435    1.212042
     42          6           0        3.052350    0.636343   -0.263330
 ---------------------------------------------------------------------

 Cartesian coordinates of GON1-Al3
 ---------------------------------------------------------------------
 Center     Atomic      Atomic             Coordinates (Angstroms)
 Number     Number       Type             X           Y           Z
 ---------------------------------------------------------------------
      1          6           0        4.073289   -0.788888   -1.074688
      2          6           0        3.220235    0.411909   -0.882312
      3          6           0        2.055971    0.425433   -0.095333
      4          6           0        3.856339   -2.077885   -0.724865
      5          6           0        1.214545    1.543638   -0.123754
      6          6           0       -0.420378   -1.021096    0.352565
      7          6           0       -1.024354    0.236671    0.288412
      8          6           0       -2.410214    0.386516    0.078378
      9          6           0       -2.443194   -1.985938   -0.499696
     10          6           0       -1.105623   -2.149714   -0.119640
     11          6           0       -0.486199   -3.488726   -0.293011
     12          6           0        0.811006   -3.866487   -0.218254
     13          1           0        0.953993   -4.924336   -0.452841
     14          1           0       -1.219368   -4.247305   -0.577692
     15          1           0        4.984968   -0.562924   -1.633979
     16          1           0        4.657826   -2.745357   -1.050441
     17          1           0       -3.012839   -2.830052   -0.877129
     18          6           0        3.581234    1.594227   -1.557670
     19          6           0        2.845653    2.759634   -1.407422
     20          6           0        1.646895    2.765816   -0.672122
     21          1           0        4.479134    1.609762   -2.170712
     22          1           0        3.196818    3.685898   -1.854252
     23          6           0        0.944222    3.981043   -0.351126
     24          6           0       -0.223090    3.949251    0.363639
     25          6           0       -0.864329    2.729806    0.700187
     26          1           0        1.350489    4.928787   -0.692391
     27          1           0       -0.768828    4.872434    0.540052
     28          6           0       -3.029729    1.631476    0.479884
     29          1           0       -4.107491    1.708927    0.484800
     30          6           0       -2.277147    2.715772    0.830080
     31          1           0       -2.777395    3.654498    1.053846
     32          6           0       -4.554736   -0.812801   -0.731362
     33          8           0       -5.185621   -1.836591   -0.888089
     34          8           0       -5.135608    0.390297   -0.929690
     35          1           0       -6.051593    0.205004   -1.180112
     36          8           0        2.146715   -2.000211    1.870419
     37          8           0        0.389410    0.936063    2.078545
     38          1           0        0.958954    1.631297    2.439880
     39          6           0       -3.111744   -0.768738   -0.360969
     40          6           0       -0.126329    1.439209    0.621470
     41         13           0        2.346921   -2.768759    0.322829
     42         13           0        1.237865   -0.659230    1.316181
 ---------------------------------------------------------------------

 Cartesian coordinates of GON1-Al4
 ---------------------------------------------------------------------
 Center     Atomic      Atomic             Coordinates (Angstroms)
 Number     Number       Type             X           Y           Z
 ---------------------------------------------------------------------
      1          6           0       -3.758774    1.982798   -0.588644
      2          6           0       -3.404983    0.622581   -0.179151
      3          6           0       -2.175242    0.388912    0.367304
      4          6           0       -1.115018    1.408407    0.495270
      5          6           0       -2.886013    2.996642   -0.459862
      6          6           0        0.351819    1.001343    0.316231
      7          6           0        0.879867   -0.319991    0.348623
      8          6           0        2.238314   -0.574771    0.022022
      9          6           0        2.603416    1.794970   -0.189608
     10          6           0        1.246796    2.055130   -0.010068
     11          6           0        0.801048    3.415241   -0.299973
     12          6           0       -0.487293    3.768845   -0.339032
     13          1           0       -0.793376    4.752721   -0.683030
     14          1           0        1.570032    4.128410   -0.582571
     15          1           0       -4.725295    2.160036   -1.053561
     16          1           0       -3.133749    3.981224   -0.848937
     17          1           0        3.281407    2.615752   -0.400157
     18          6           0       -4.364237   -0.466266   -0.497806
     19          6           0       -4.135455   -1.806405   -0.566017
     20          6           0       -2.857316   -2.434969   -0.204070
     21          1           0       -5.352930   -0.129132   -0.803220
     22          1           0       -4.956982   -2.435009   -0.910857
     23          6           0       -2.050301   -3.139220   -1.036706
     24          6           0       -0.597434   -3.411583   -0.877634
     25          6           0        0.361135   -2.722083   -0.201312
     26          1           0       -2.429324   -3.465678   -2.010977
     27          1           0       -0.230708   -4.201913   -1.532281
     28          6           0        2.663015   -1.965857   -0.241472
     29          1           0        3.714605   -2.144985   -0.416510
     30          6           0        1.783300   -2.971729   -0.423941
     31          1           0        2.125548   -3.941467   -0.776080
     32          6           0        4.592897    0.425238   -0.393217
     33          8           0        5.235966    1.270343   -0.976297
     34          8           0        5.191935   -0.645181    0.166849
     35          1           0        6.137162   -0.556906   -0.021232
     36          8           0       -1.387493    2.419620    1.467955
     37          8           0        0.625936   -2.001877    2.100644
     38          1           0        1.568358   -2.208797    2.043709
     39          6           0        3.117908    0.507530   -0.170388
     40          6           0        0.145570   -1.592253    0.804589
     41         13           0       -1.744513   -1.354805    0.867105
     42          6           0       -1.524155    2.823317    0.099887
 ---------------------------------------------------------------------

 Cartesian coordinates of GON1-Al5
 ---------------------------------------------------------------------
 Center     Atomic      Atomic             Coordinates (Angstroms)
 Number     Number       Type             X           Y           Z
 ---------------------------------------------------------------------
      1          6           0       -3.742686   -1.811054    0.249417
      2          6           0       -3.515937   -0.422284    0.318838
      3          6           0       -0.737539   -1.078767   -0.667866
      4          6           0       -2.660199   -2.726358    0.199614
      5          6           0        0.731429   -0.890971   -0.639442
      6          6           0        1.208543    0.451288   -0.568264
      7          6           0        2.498250    0.741175   -0.049256
      8          6           0        2.939894   -1.663805   -0.090005
      9          6           0        1.585909   -1.946055   -0.384342
     10          6           0        1.033409   -3.293269   -0.242863
     11          6           0       -0.288023   -3.536907   -0.216203
     12          1           0       -0.647904   -4.540660   -0.007199
     13          1           0        1.733406   -4.107574   -0.076909
     14          1           0       -4.716366   -2.241194    0.504120
     15          1           0       -2.901832   -3.780713    0.300500
     16          1           0        3.639221   -2.478465    0.071062
     17          6           0       -4.419729    0.671269    0.617690
     18          6           0       -3.910653    1.984013    0.576665
     19          6           0       -2.562860    2.202942    0.204503
     20          1           0       -5.466871    0.510723    0.878073
     21          1           0       -4.572590    2.811107    0.840308
     22          6           0       -1.949284    3.489674    0.110681
     23          6           0       -0.615332    3.763514   -0.125119
     24          6           0        0.477121    2.785738   -0.221092
     25          1           0       -2.578327    4.369157    0.270414
     26          1           0       -0.307825    4.805971   -0.090803
     27          6           0        2.750120    2.083346    0.370274
     28          1           0        3.718442    2.340280    0.774380
     29          6           0        1.758517    3.030594    0.354537
     30          1           0        1.948392    4.008715    0.786647
     31          6           0        4.834404   -0.278772    0.482285
     32          8           0        5.512919   -1.226357    0.815777
     33          8           0        5.366301    0.956534    0.392707
     34          1           0        6.296104    0.868371    0.645603
     35          8           0       -1.297481   -1.892466   -1.735351
     36          8           0       -0.718003    1.348422   -1.735943
     37          1           0       -1.052653    2.226047   -1.983135
     38          6           0        3.394117   -0.374616    0.101136
     39          6           0        0.338649    1.554015   -0.827293
     40         13           0       -1.914186    0.450395   -0.332318
     41          6           0       -1.305562   -2.472781   -0.433806
     42         13           0       -2.167078   -1.362117    1.906848
 ---------------------------------------------------------------------

 Cartesian coordinates of GON1-Al6
 ---------------------------------------------------------------------
 Center     Atomic      Atomic             Coordinates (Angstroms)
 Number     Number       Type             X           Y           Z
 ---------------------------------------------------------------------
      1          6           0        4.621737   -1.307800   -0.498247
      2          6           0        3.833723   -0.050362   -0.447135
      3          6           0        4.206984   -2.575024   -0.290085
      4          6           0       -0.607653   -0.872499    0.245224
      5          6           0       -1.250498    0.303565    0.357750
      6          6           0       -2.537436    0.893496    0.216789
      7          6           0       -2.511981   -2.084437   -0.712400
      8          6           0       -1.154998   -1.985844   -0.607576
      9          6           0       -0.313956   -2.991740   -1.311313
     10          6           0        0.967353   -3.392586   -1.164282
     11          1           0        1.240250   -4.142554   -1.913601
     12          1           0       -0.896626   -3.469110   -2.102627
     13          1           0        5.667022   -1.141558   -0.772295
     14          1           0        4.983596   -3.332006   -0.401492
     15          1           0       -2.885953   -2.972467   -1.224626
     16          6           0        4.427825    1.081342   -1.041844
     17          6           0        3.799093    2.320195   -1.108266
     18          6           0        2.515890    2.483118   -0.565765
     19          1           0        5.418934    0.979619   -1.478618
     20          1           0        4.309599    3.153519   -1.586677
     21          6           0        1.818470    3.782696   -0.661346
     22          6           0        0.535823    4.134716   -0.341280
     23          6           0       -0.478006    3.237438    0.193821
     24          1           0        2.411613    4.568425   -1.128798
     25          1           0        0.226335    5.144238   -0.620491
     26          6           0       -2.765362    2.252607    0.021449
     27          1           0       -3.799935    2.534535   -0.169823
     28          6           0       -1.815639    3.336776   -0.089190
     29          1           0       -2.216446    4.247964   -0.542583
     30          6           0       -5.682469   -0.675359   -0.235542
     31          8           0       -6.360682   -1.651833    0.010414
     32          8           0       -6.257127    0.505096   -0.581987
     33          1           0       -7.220889    0.375254   -0.587537
     34          8           0        1.705942   -2.495813    1.693075
     35          8           0        0.627228    0.506554    2.412080
     36          1           0        1.295687    0.833391    3.024652
     37          6           0        2.553346    0.095777    0.144549
     38          6           0        1.932427    1.360596    0.053713
     39         13           0        2.349418   -2.925158    0.145509
     40         13           0        1.191372   -0.909283    1.287053
     41         13           0        0.221556    1.574899    0.885485
     42         13           0       -3.671893   -0.645942   -0.142531
 ---------------------------------------------------------------------
