# Supplementary material for: Data of electronic, reactivity, optoelectronic, linear and non-linear optical parameters of doping graphene oxide nanosheet with aluminum atom
Source: Data Brief. 2022 Jan 19;41:107840. doi: 10.1016/j.dib.2022.107840 (PMC8801356; doi:10.1016/j.dib.2022.107840)
Supplement: Supplementary file 1 [file mmc1.zip › supplementary file/Cartesian coordinates/Cartesian coordinates OF GON2 AND ITS DERIVATIVES (GON2-Alx) B3LYP.rtf]

Cartesian coordinates of GON2
 ---------------------------------------------------------------------
 Center     Atomic      Atomic             Coordinates (Angstroms)
 Number     Number       Type             X           Y           Z
 ---------------------------------------------------------------------
      1          6           0       -3.661595    1.623021   -0.414648
      2          6           0       -3.065616    0.295758   -0.409020
      3          6           0       -1.683533    0.137544   -0.136323
      4          6           0       -0.868192    1.323137    0.235076
      5          6           0       -1.524640    2.682510    0.208556
      6          6           0       -2.952401    2.745831   -0.173953
      7          6           0       -1.074431   -1.111495   -0.231948
      8          6           0        0.589443    1.236040   -0.029401
      9          6           0        1.205427   -0.008223   -0.125799
     10          6           0        2.593484   -0.120880   -0.373096
     11          6           0        3.342736    1.074506   -0.499535
     12          6           0        2.729659    2.317783   -0.408667
     13          6           0        1.345927    2.422042   -0.188773
     14          6           0        0.679260    3.715260   -0.199746
     15          6           0       -0.656567    3.850095   -0.060906
     16          1           0       -1.129339    4.824236   -0.154997
     17          1           0        1.295002    4.593974   -0.375951
     18          1           0       -4.715188    1.694100   -0.673892
     19          1           0       -3.412726    3.726207   -0.264703
     20          1           0        3.321921    3.223339   -0.526787
     21          6           0       -3.817066   -0.844519   -0.748601
     22          6           0       -3.212328   -2.087911   -0.854107
     23          6           0       -1.831569   -2.247478   -0.613724
     24          1           0       -4.879812   -0.738901   -0.950341
     25          1           0       -3.800575   -2.955982   -1.139616
     26          6           0       -1.174738   -3.516577   -0.794911
     27          6           0        0.198264   -3.624826   -0.746412
     28          6           0        1.028193   -2.526987   -0.435140
     29          1           0       -1.780204   -4.378543   -1.059881
     30          1           0        0.673756   -4.565424   -1.014804
     31          6           0        3.200449   -1.414568   -0.529746
     32          1           0        4.272008   -1.468197   -0.679446
     33          6           0        2.427280   -2.555832   -0.600929
     34          1           0        2.895452   -3.503608   -0.856266
     35          8           0       -1.251565    1.987038    1.465657
     36          8           0        4.686303    0.939501   -0.729162
     37          1           0        5.101203    1.810454   -0.777283
     38          6           0        0.488051   -1.502470    1.722094
     39          8           0        1.378957   -1.089744    2.426170
     40          8           0       -0.522669   -2.265887    2.190639
     41          1           0       -0.363036   -2.389628    3.142200
     42          6           0        0.393392   -1.268310    0.160547
 ---------------------------------------------------------------------

Cartesian coordinates of GON2-Al1
 ---------------------------------------------------------------------
 Center     Atomic      Atomic             Coordinates (Angstroms)
 Number     Number       Type             X           Y           Z
 ---------------------------------------------------------------------
      1          6           0       -4.036802   -0.855255   -0.388583
      2          6           0       -2.756631   -1.531815   -0.535898
      3          6           0       -1.557928   -0.935775   -0.050914
      4          6           0       -1.582555    0.502245    0.479481
      5          6           0       -2.935362    1.162497    0.532483
      6          6           0       -4.131802    0.392797    0.105717
      7          6           0       -0.359491   -1.636204   -0.166128
      8          6           0       -0.393967    1.428836    0.210026
      9          6           0        0.899393    0.935834    0.103797
     10          6           0        1.952048    1.687137   -0.489265
     11          6           0        1.721720    3.071848   -0.720326
     12          6           0        0.463304    3.619177   -0.451102
     13          6           0       -0.615506    2.816168   -0.042696
     14          6           0       -1.942859    3.395353    0.100286
     15          6           0       -3.022237    2.636136    0.363864
     16          1           0       -4.010608    3.086346    0.419541
     17          1           0       -2.050537    4.468766   -0.035802
     18          1           0       -4.931015   -1.383419   -0.711122
     19          1           0       -5.096060    0.891517    0.168121
     20          1           0        0.297698    4.682360   -0.616411
     21          6           0       -2.681302   -2.779723   -1.189744
     22          6           0       -1.456606   -3.383941   -1.462751
     23          6           0       -0.265170   -2.793056   -0.983765
     24          1           0       -3.600273   -3.243336   -1.538956
     25          1           0       -1.424505   -4.292123   -2.061005
     26          6           0        1.028049   -3.250694   -1.499563
     27          6           0        2.265077   -2.601507   -1.534118
     28          6           0        2.482413   -1.347044   -0.897957
     29          1           0        0.967659   -4.155188   -2.106184
     30          1           0        3.013182   -3.038684   -2.202313
     31          6           0        3.123806    1.036945   -1.047757
     32          1           0        3.837796    1.721877   -1.500789
     33          6           0        3.364643   -0.325175   -1.309435
     34          1           0        4.198010   -0.527855   -1.989091
     35          8           0       -2.271334    0.700850    1.744215
     36          8           0        2.738059    3.829242   -1.222071
     37          1           0        2.441523    4.738749   -1.362610
     38          6           0        2.012125   -1.318837    2.434727
     39          8           0        2.955033   -0.730903    2.939187
     40          8           0        1.397577   -2.337231    3.114673
     41          1           0        1.854910   -2.426632    3.974462
     42         13           0        1.269336   -0.905672    0.583989
 ---------------------------------------------------------------------

Cartesian coordinates of GON2-Al2
 ---------------------------------------------------------------------
 Center     Atomic      Atomic             Coordinates (Angstroms)
 Number     Number       Type             X           Y           Z
 ---------------------------------------------------------------------
      1          6           0       -3.381870    2.375289   -0.457005
      2          6           0       -3.069848    0.957560   -0.530053
      3          6           0       -1.759247    0.492132   -0.246230
      4          6           0       -0.683986    1.445245    0.158984
      5          6           0       -1.077018    2.886258    0.246126
      6          6           0       -2.459778    3.287207   -0.106343
      7          6           0       -1.474774   -0.865935   -0.298696
      8          6           0        0.752610    1.037524   -0.095252
      9          6           0        1.035292   -0.318631   -0.148191
     10          6           0        2.370605   -0.875677   -0.334803
     11          6           0        3.111892    2.054579   -0.342847
     12          6           0        1.745909    2.159169   -0.202373
     13          6           0        1.241133    3.543347   -0.156617
     14          6           0       -0.031586    3.904359    0.041745
     15          1           0       -0.323661    4.951283    0.054679
     16          1           0        1.995520    4.313639   -0.290190
     17          1           0       -4.394554    2.681911   -0.706673
     18          1           0       -2.696263    4.347668   -0.085620
     19          1           0        3.632631    3.010386   -0.387456
     20          6           0       -4.059691    0.030884   -0.890116
     21          6           0       -3.769030   -1.323516   -0.961248
     22          6           0       -2.481145   -1.794514   -0.660398
     23          1           0       -5.062791    0.386008   -1.110442
     24          1           0       -4.540462   -2.034875   -1.243544
     25          6           0       -2.173404   -3.207475   -0.732313
     26          6           0       -0.905574   -3.660727   -0.582683
     27          6           0        0.194558   -2.768046   -0.329677
     28          1           0       -2.980208   -3.895929   -0.968291
     29          1           0       -0.674521   -4.713095   -0.725541
     30          6           0        2.558683   -2.234703   -0.433147
     31          1           0        3.568836   -2.621579   -0.551639
     32          6           0        1.485136   -3.175490   -0.484695
     33          1           0        1.691903   -4.213143   -0.735470
     34          8           0       -0.930389    2.078443    1.456602
     35          8           0        5.479193   -0.265686   -0.614663
     36          1           0        6.279253    0.263395   -0.657930
     37          6           0       -0.115541   -1.486438    1.728117
     38          8           0        0.794662   -1.181829    2.461331
     39          8           0       -1.264937   -2.034169    2.180289
     40          1           0       -1.179557   -2.104833    3.146645
     41         13           0        3.871151    0.315520   -0.433936
     42          6           0       -0.098212   -1.336842    0.156744
 ---------------------------------------------------------------------

 Cartesian coordinates of GON2-Al3
 ---------------------------------------------------------------------
 Center     Atomic      Atomic             Coordinates (Angstroms)
 Number     Number       Type             X           Y           Z
 ---------------------------------------------------------------------
      1          6           0        3.609480    1.559928   -0.904615
      2          6           0        2.206010    2.010952   -0.736325
      3          6           0        1.174633    1.290451   -0.070930
      4          6           0        4.225584    0.368095   -0.672098
      5          6           0       -0.158124    1.690439   -0.224294
      6          6           0        0.086691   -1.397087    0.147350
      7          6           0       -1.160939   -0.787573   -0.021616
      8          6           0       -2.312917   -1.528345   -0.409330
      9          6           0       -0.837268   -3.436642   -0.801871
     10          6           0        0.284174   -2.718399   -0.341800
     11          6           0        1.593190   -3.402187   -0.490842
     12          6           0        2.874885   -2.955337   -0.391643
     13          1           0        3.608892   -3.724111   -0.646838
     14          1           0        1.461794   -4.439705   -0.811731
     15          1           0        4.215907    2.336693   -1.379225
     16          1           0        5.269697    0.363226   -0.995427
     17          1           0       -0.712310   -4.454989   -1.168251
     18          6           0        1.867352    3.233700   -1.355189
     19          6           0        0.567620    3.718005   -1.332492
     20          6           0       -0.483665    2.958410   -0.780838
     21          1           0        2.643033    3.817723   -1.845266
     22          1           0        0.339089    4.686765   -1.769526
     23          6           0       -1.829627    3.463523   -0.769681
     24          6           0       -2.883888    2.649231   -0.437777
     25          6           0       -2.711019    1.286345   -0.108331
     26          1           0       -2.005520    4.481950   -1.104270
     27          1           0       -3.902249    3.012558   -0.555301
     28          6           0       -3.627603   -0.956566   -0.406097
     29          1           0       -4.476022   -1.600289   -0.604442
     30          6           0       -3.796735    0.400462   -0.253990
     31          1           0       -4.786715    0.832482   -0.380943
     32          8           0        2.744245   -0.775977    1.761910
     33          8           0       -3.219779   -3.587490   -1.166976
     34          1           0       -2.965604   -4.493515   -1.385074
     35          6           0       -1.171672    0.824604    1.828504
     36          8           0       -0.118709    0.442864    2.400877
     37          8           0       -2.159962    1.299008    2.557842
     38          1           0       -1.895486    1.277697    3.496096
     39          6           0       -1.338198    0.761576    0.315424
     40          6           0       -2.108285   -2.876212   -0.787072
     41         13           0        3.453581   -1.187756    0.235887
     42         13           0        1.224963   -0.202087    1.206752
 ---------------------------------------------------------------------

Cartesian coordinates of GON2-Al4
 ---------------------------------------------------------------------
 Center     Atomic      Atomic             Coordinates (Angstroms)
 Number     Number       Type             X           Y           Z
 ---------------------------------------------------------------------
      1          6           0       -4.098284    0.507988   -0.372656
      2          6           0       -3.146934   -0.581289   -0.135293
      3          6           0       -1.888512   -0.267313    0.325100
      4          6           0       -1.366093    1.101956    0.489378
      5          6           0       -3.744602    1.800152   -0.194842
      6          6           0        0.106120    1.372747    0.160602
      7          6           0        1.147327    0.407774    0.027593
      8          6           0        2.432223    0.768350   -0.460331
      9          6           0        1.747573    3.085472   -0.503864
     10          6           0        0.435945    2.727606   -0.157389
     11          6           0       -0.577740    3.769101   -0.275321
     12          6           0       -1.898765    3.537155   -0.171478
     13          1           0       -2.624926    4.313391   -0.399801
     14          1           0       -0.226458    4.760219   -0.551253
     15          1           0       -5.088880    0.269703   -0.753518
     16          1           0       -4.438780    2.594185   -0.462116
     17          1           0        1.970314    4.130287   -0.711557
     18          6           0       -3.536804   -1.954102   -0.545584
     19          6           0       -2.743512   -3.053038   -0.778819
     20          6           0       -1.306555   -3.090827   -0.515812
     21          1           0       -4.588393   -2.063763   -0.805334
     22          1           0       -3.234546   -3.927203   -1.212300
     23          6           0       -0.293677   -3.474883   -1.354010
     24          6           0        1.118734   -3.058609   -1.278989
     25          6           0        1.723031   -2.001173   -0.638692
     26          1           0       -0.521591   -4.026752   -2.273778
     27          1           0        1.763491   -3.586638   -1.981160
     28          6           0        3.405337   -0.258554   -0.829102
     29          1           0        4.392934    0.081425   -1.119393
     30          6           0        3.066658   -1.566777   -0.988046
     31          1           0        3.761917   -2.261878   -1.453053
     32          8           0       -1.957356    1.839721    1.584687
     33          8           0        4.002482    2.444661   -1.077157
     34          1           0        4.081939    3.401377   -1.184305
     35          6           0        1.787538   -1.271391    1.764049
     36          8           0        2.095020   -0.409467    2.560899
     37          8           0        1.946140   -2.601146    2.061837
     38          1           0        2.359131   -2.635832    2.940880
     39          6           0        1.109350   -1.077175    0.430593
     40          6           0        2.733860    2.126006   -0.670774
     41         13           0       -0.709934   -1.679604    0.573970
     42          6           0       -2.390102    2.216855    0.250844
 ---------------------------------------------------------------------

Cartesian coordinates of GON2-Al5
 ---------------------------------------------------------------------
 Center     Atomic      Atomic             Coordinates (Angstroms)
 Number     Number       Type             X           Y           Z
 ---------------------------------------------------------------------
      1          6           0       -3.531436    1.678291   -0.165610
      2          6           0       -3.177325    0.306806   -0.303718
      3          6           0       -0.421728    1.172168    0.543597
      4          6           0       -2.554788    2.689445   -0.046466
      5          6           0        1.045748    1.112195    0.412408
      6          6           0        1.626897   -0.168362    0.228967
      7          6           0        2.947249   -0.276147   -0.303097
      8          6           0        3.177536    2.157731   -0.115867
      9          6           0        1.806100    2.273451    0.217040
     10          6           0        1.120839    3.561633    0.192493
     11          6           0       -0.226196    3.688851    0.241411
     12          1           0       -0.678636    4.667957    0.101796
     13          1           0        1.730808    4.449886    0.044227
     14          1           0       -4.545711    2.027181   -0.389142
     15          1           0       -2.897196    3.720092   -0.095009
     16          1           0        3.780759    3.055330   -0.239874
     17          6           0       -3.958312   -0.828349   -0.700586
     18          6           0       -3.306636   -2.080811   -0.837505
     19          6           0       -1.926151   -2.190510   -0.527238
     20          1           0       -5.026328   -0.762994   -0.925502
     21          1           0       -3.885652   -2.934315   -1.199248
     22          6           0       -1.156357   -3.345584   -0.798627
     23          6           0        0.238611   -3.524683   -0.721928
     24          6           0        1.238056   -2.560384   -0.374750
     25          1           0       -1.682556   -4.205563   -1.225936
     26          1           0        0.613261   -4.463795   -1.126695
     27          6           0        3.408046   -1.531700   -0.810976
     28          1           0        4.416282   -1.590781   -1.205276
     29          6           0        2.553162   -2.604740   -0.926857
     30          1           0        2.866710   -3.493785   -1.468096
     31          8           0       -0.977557    1.865648    1.721014
     32          8           0        5.012606    0.764453   -0.835351
     33          1           0        5.431152    1.630212   -0.928239
     34          6           0        0.144223   -1.734284    1.650364
     35          8           0       -0.884645   -1.125609    2.059789
     36          8           0        0.560013   -2.796419    2.342399
     37          1           0       -0.099949   -2.977915    3.034940
     38          6           0        0.903177   -1.426921    0.437847
     39          6           0        3.722037    0.916238   -0.400811
     40         13           0       -1.492719   -0.464945    0.343844
     41          6           0       -1.133961    2.530166    0.449060
     42         13           0       -1.964128    1.413168   -1.937357
 ---------------------------------------------------------------------
                            
Cartesian coordinates of GON2-Al6
 ---------------------------------------------------------------------
 Center     Atomic      Atomic             Coordinates (Angstroms)
 Number     Number       Type             X           Y           Z
 ---------------------------------------------------------------------
      1          6           0        4.316567   -0.894769   -0.473863
      2          6           0        3.415102    0.279888   -0.423709
      3          6           0        4.050284   -2.222555   -0.368493
      4          6           0       -0.877312   -1.125916    0.012378
      5          6           0       -1.601950    0.027217   -0.048437
      6          6           0       -2.950173    0.418707   -0.355700
      7          6           0       -2.644183   -2.635937   -0.807773
      8          6           0       -1.300009   -2.381421   -0.715855
      9          6           0       -0.375932   -3.361386   -1.354857
     10          6           0        0.951352   -3.630792   -1.250687
     11          1           0        1.265432   -4.409844   -1.952204
     12          1           0       -0.937309   -3.963346   -2.074338
     13          1           0        5.348228   -0.606476   -0.695314
     14          1           0        4.926904   -2.860012   -0.499488
     15          1           0       -2.915346   -3.603634   -1.236285
     16          6           0        3.944211    1.460232   -1.006300
     17          6           0        3.191424    2.613104   -1.187141
     18          6           0        1.842231    2.653739   -0.768081
     19          1           0        4.969959    1.446259   -1.368271
     20          1           0        3.637341    3.472103   -1.685103
     21          6           0        1.022819    3.813091   -1.131192
     22          6           0       -0.349814    4.007620   -1.089598
     23          6           0       -1.283401    3.055375   -0.564283
     24          1           0        1.583932    4.606037   -1.625978
     25          1           0       -0.724254    4.896559   -1.604931
     26          6           0       -3.363366    1.706470   -0.742782
     27          1           0       -4.412610    1.786410   -1.026023
     28          6           0       -2.609464    2.914730   -0.937690
     29          1           0       -3.126424    3.701816   -1.496277
     30          8           0        1.567799   -2.471473    1.515149
     31          8           0       -5.656269   -1.381994   -0.301695
     32          1           0       -6.171083   -2.188788   -0.391032
     33          6           0       -0.351669    1.140532    2.478559
     34          8           0        0.258246    0.036395    2.642333
     35          8           0       -0.747637    1.758596    3.577778
     36          1           0       -0.483052    1.232158    4.361301
     37         13           0        2.265766   -2.909931    0.004652
     38         13           0        0.872802   -0.940106    1.136058
     39         13           0       -0.507059    1.638426    0.493072
     40         13           0       -3.944993   -1.258037   -0.399566
     41          6           0        2.088041    0.328383    0.109577
     42          6           0        1.341320    1.511021   -0.077304
 ---------------------------------------------------------------------
