# Supplementary material for: Data of electronic, reactivity, optoelectronic, linear and non-linear optical parameters of doping graphene oxide nanosheet with aluminum atom
Source: Data Brief. 2022 Jan 19;41:107840. doi: 10.1016/j.dib.2022.107840 (PMC8801356; doi:10.1016/j.dib.2022.107840)
Supplement: Supplementary file 1 [file mmc1.zip › supplementary file/Cartesian coordinates/Cartesian coordinates OF GON2 AND ITS DERIVATIVES (GON2-Alx) B3LYP-D3.rtf]

Cartesian coordinates of GON2
 ---------------------------------------------------------------------
 Center     Atomic      Atomic             Coordinates (Angstroms)
 Number     Number       Type             X           Y           Z
 ---------------------------------------------------------------------
      1          6           0       -3.647243    1.654140   -0.409986
      2          6           0       -3.063039    0.321792   -0.407292
      3          6           0       -1.681037    0.152090   -0.144612
      4          6           0       -0.854564    1.328489    0.226652
      5          6           0       -1.499257    2.692954    0.207888
      6          6           0       -2.927132    2.770304   -0.170913
      7          6           0       -1.083411   -1.100769   -0.242293
      8          6           0        0.600484    1.230302   -0.041391
      9          6           0        1.204752   -0.018058   -0.139592
     10          6           0        2.592863   -0.144340   -0.374596
     11          6           0        3.353835    1.044125   -0.494915
     12          6           0        2.751069    2.293048   -0.406277
     13          6           0        1.367421    2.409786   -0.192638
     14          6           0        0.711784    3.708474   -0.201058
     15          6           0       -0.622831    3.854018   -0.061466
     16          1           0       -1.089120    4.831286   -0.153616
     17          1           0        1.334696    4.582427   -0.375818
     18          1           0       -4.701088    1.734947   -0.665380
     19          1           0       -3.377423    3.755408   -0.258874
     20          1           0        3.353093    3.192644   -0.519210
     21          6           0       -3.825827   -0.813449   -0.738614
     22          6           0       -3.232386   -2.062814   -0.842215
     23          6           0       -1.851616   -2.232913   -0.609970
     24          1           0       -4.889047   -0.700085   -0.933433
     25          1           0       -3.830344   -2.927260   -1.118269
     26          6           0       -1.206010   -3.508977   -0.780201
     27          6           0        0.166119   -3.629369   -0.733125
     28          6           0        1.006063   -2.536449   -0.433766
     29          1           0       -1.819683   -4.368700   -1.033158
     30          1           0        0.632264   -4.577675   -0.990249
     31          6           0        3.188394   -1.444442   -0.519592
     32          1           0        4.260738   -1.508868   -0.658899
     33          6           0        2.405533   -2.579259   -0.590019
     34          1           0        2.867277   -3.533094   -0.833884
     35          8           0       -1.227636    1.989762    1.461606
     36          8           0        4.697777    0.895876   -0.714820
     37          1           0        5.124522    1.761234   -0.755309
     38          6           0        0.469884   -1.494386    1.708530
     39          8           0        1.354734   -1.071423    2.413747
     40          8           0       -0.538529   -2.265600    2.169000
     41          1           0       -0.389670   -2.389923    3.121839
     42          6           0        0.381653   -1.267840    0.147794
 ---------------------------------------------------------------------

Cartesian coordinates of GON2-Al1
 ---------------------------------------------------------------------
 Center     Atomic      Atomic             Coordinates (Angstroms)
 Number     Number       Type             X           Y           Z
 ---------------------------------------------------------------------
      1          6           0       -4.043441   -0.813814   -0.391079
      2          6           0       -2.770413   -1.503668   -0.537807
      3          6           0       -1.566345   -0.921764   -0.050808
      4          6           0       -1.575674    0.516454    0.477909
      5          6           0       -2.921012    1.190489    0.531795
      6          6           0       -4.125872    0.434185    0.104924
      7          6           0       -0.376470   -1.636197   -0.161187
      8          6           0       -0.378581    1.430103    0.205597
      9          6           0        0.909137    0.924204    0.102173
     10          6           0        1.970617    1.662196   -0.491156
     11          6           0        1.754135    3.048187   -0.727174
     12          6           0        0.500580    3.608803   -0.460886
     13          6           0       -0.586363    2.818308   -0.050393
     14          6           0       -1.907408    3.411546    0.091992
     15          6           0       -2.993943    2.664419    0.359476
     16          1           0       -3.977729    3.124124    0.416073
     17          1           0       -2.004417    4.485627   -0.047124
     18          1           0       -4.943038   -1.332204   -0.714615
     19          1           0       -5.084543    0.943097    0.168743
     20          1           0        0.347174    4.673143   -0.629683
     21          6           0       -2.707607   -2.751871   -1.192302
     22          6           0       -1.489060   -3.369949   -1.462248
     23          6           0       -0.292275   -2.793639   -0.979044
     24          1           0       -3.630556   -3.205314   -1.544320
     25          1           0       -1.466201   -4.277636   -2.061658
     26          6           0        0.996926   -3.265234   -1.492822
     27          6           0        2.240834   -2.629158   -1.524124
     28          6           0        2.469254   -1.377516   -0.887416
     29          1           0        0.928230   -4.168721   -2.100085
     30          1           0        2.987182   -3.071303   -2.190936
     31          6           0        3.136688    0.998110   -1.045544
     32          1           0        3.858813    1.673570   -1.499884
     33          6           0        3.363255   -0.367529   -1.301173
     34          1           0        4.194232   -0.584436   -1.979267
     35          8           0       -2.260096    0.723273    1.743458
     36          8           0        2.779022    3.793309   -1.230284
     37          1           0        2.493866    4.705174   -1.377423
     38          6           0        1.991751   -1.320886    2.437317
     39          8           0        2.948662   -0.745334    2.929970
     40          8           0        1.354426   -2.315998    3.131955
     41          1           0        1.807139   -2.406819    3.993903
     42         13           0        1.255497   -0.918074    0.586874
 ---------------------------------------------------------------------

Cartesian coordinates of GON2-Al2
 ---------------------------------------------------------------------
 Center     Atomic      Atomic             Coordinates (Angstroms)
 Number     Number       Type             X           Y           Z
 ---------------------------------------------------------------------
      1          6           0       -3.380068    2.374659   -0.452748
      2          6           0       -3.068138    0.957449   -0.532949
      3          6           0       -1.756216    0.492034   -0.258848
      4          6           0       -0.680460    1.441985    0.148795
      5          6           0       -1.073973    2.881216    0.248322
      6          6           0       -2.457402    3.284627   -0.098945
      7          6           0       -1.472435   -0.864862   -0.311961
      8          6           0        0.754856    1.036569   -0.109837
      9          6           0        1.036221   -0.318884   -0.163931
     10          6           0        2.370767   -0.878718   -0.338909
     11          6           0        3.114570    2.052390   -0.346017
     12          6           0        1.747917    2.158199   -0.209440
     13          6           0        1.242128    3.541888   -0.158043
     14          6           0       -0.030095    3.901122    0.046080
     15          1           0       -0.323114    4.947575    0.065184
     16          1           0        1.994055    4.314947   -0.290391
     17          1           0       -4.393388    2.682973   -0.697821
     18          1           0       -2.693771    4.344812   -0.070667
     19          1           0        3.639090    3.006500   -0.386340
     20          6           0       -4.059934    0.029938   -0.885901
     21          6           0       -3.770349   -1.325115   -0.954100
     22          6           0       -2.480917   -1.795391   -0.659149
     23          1           0       -5.064808    0.383865   -1.099912
     24          1           0       -4.544725   -2.036936   -1.226918
     25          6           0       -2.174533   -3.209032   -0.715263
     26          6           0       -0.907286   -3.662707   -0.561990
     27          6           0        0.194443   -2.769182   -0.321707
     28          1           0       -2.982800   -3.899840   -0.938952
     29          1           0       -0.679636   -4.717754   -0.689357
     30          6           0        2.558822   -2.238935   -0.419336
     31          1           0        3.569551   -2.627850   -0.526387
     32          6           0        1.485292   -3.179963   -0.464446
     33          1           0        1.693407   -4.221213   -0.698435
     34          8           0       -0.923629    2.063536    1.452536
     35          8           0        5.473359   -0.280333   -0.603229
     36          1           0        6.278290    0.241632   -0.640720
     37          6           0       -0.120012   -1.465201    1.715046
     38          8           0        0.781867   -1.136789    2.447736
     39          8           0       -1.264304   -2.028002    2.161677
     40          1           0       -1.191288   -2.088992    3.129304
     41         13           0        3.869623    0.311821   -0.433178
     42          6           0       -0.097247   -1.332534    0.144106
 ---------------------------------------------------------------------

Cartesian coordinates of GON2-Al3
 ---------------------------------------------------------------------
 Center     Atomic      Atomic             Coordinates (Angstroms)
 Number     Number       Type             X           Y           Z
 ---------------------------------------------------------------------
      1          6           0        3.611137    1.558925   -0.900734
      2          6           0        2.207676    2.010524   -0.734858
      3          6           0        1.175518    1.289291   -0.071675
      4          6           0        4.225755    0.366361   -0.667120
      5          6           0       -0.156854    1.688957   -0.228026
      6          6           0        0.088540   -1.396533    0.144292
      7          6           0       -1.158954   -0.787645   -0.026757
      8          6           0       -2.311296   -1.528755   -0.409614
      9          6           0       -0.835595   -3.436480   -0.802666
     10          6           0        0.286135   -2.718387   -0.343180
     11          6           0        1.595604   -3.401676   -0.490448
     12          6           0        2.876783   -2.953532   -0.389419
     13          1           0        3.612003   -3.721850   -0.643015
     14          1           0        1.466311   -4.439739   -0.810743
     15          1           0        4.220187    2.334876   -1.373500
     16          1           0        5.270784    0.360930   -0.987950
     17          1           0       -0.713451   -4.454946   -1.169479
     18          6           0        1.869076    3.234073   -1.352673
     19          6           0        0.569560    3.718912   -1.329484
     20          6           0       -0.481943    2.958243   -0.779852
     21          1           0        2.643789    3.820096   -1.841987
     22          1           0        0.342050    4.688939   -1.764178
     23          6           0       -1.827259    3.464319   -0.764634
     24          6           0       -2.882206    2.649567   -0.435973
     25          6           0       -2.709785    1.285551   -0.114175
     26          1           0       -2.002685    4.484934   -1.092744
     27          1           0       -3.899882    3.016219   -0.548826
     28          6           0       -3.626140   -0.957604   -0.400800
     29          1           0       -4.475408   -1.602394   -0.591976
     30          6           0       -3.795750    0.399528   -0.251492
     31          1           0       -4.787438    0.829293   -0.372418
     32          8           0        2.739601   -0.775603    1.762855
     33          8           0       -3.219507   -3.588298   -1.163914
     34          1           0       -2.969075   -4.496280   -1.377140
     35          6           0       -1.176104    0.825167    1.821404
     36          8           0       -0.124230    0.443122    2.396282
     37          8           0       -2.167819    1.300891    2.544076
     38          1           0       -1.911122    1.282688    3.484329
     39          6           0       -1.337288    0.760713    0.308326
     40          6           0       -2.107145   -2.876808   -0.786524
     41         13           0        3.453159   -1.187466    0.240130
     42         13           0        1.222438   -0.201760    1.206925
 ---------------------------------------------------------------------

Cartesian coordinates of GON2-Al4
 ---------------------------------------------------------------------
 Center     Atomic      Atomic             Coordinates (Angstroms)
 Number     Number       Type             X           Y           Z
 ---------------------------------------------------------------------
      1          6           0       -4.096196    0.507429   -0.376810
      2          6           0       -3.146865   -0.582087   -0.133615
      3          6           0       -1.888628   -0.269431    0.327976
      4          6           0       -1.365576    1.099870    0.487780
      5          6           0       -3.742816    1.799355   -0.199645
      6          6           0        0.104670    1.370520    0.156417
      7          6           0        1.142819    0.405570    0.020465
      8          6           0        2.429420    0.764429   -0.463385
      9          6           0        1.747729    3.082235   -0.502463
     10          6           0        0.435218    2.725672   -0.158631
     11          6           0       -0.578179    3.767239   -0.277641
     12          6           0       -1.898957    3.534406   -0.175457
     13          1           0       -2.626379    4.308939   -0.404819
     14          1           0       -0.228440    4.758935   -0.553613
     15          1           0       -5.085660    0.268999   -0.760557
     16          1           0       -4.434893    2.594205   -0.469341
     17          1           0        1.974766    4.126454   -0.708078
     18          6           0       -3.538959   -1.955113   -0.539659
     19          6           0       -2.745432   -3.055010   -0.765618
     20          6           0       -1.309136   -3.092497   -0.498131
     21          1           0       -4.589966   -2.064777   -0.801742
     22          1           0       -3.232476   -3.931704   -1.198181
     23          6           0       -0.303439   -3.467818   -1.348167
     24          6           0        1.110616   -3.057787   -1.280983
     25          6           0        1.717300   -2.002241   -0.642433
     26          1           0       -0.544146   -4.007086   -2.272079
     27          1           0        1.751154   -3.587852   -1.985456
     28          6           0        3.402692   -0.263693   -0.828598
     29          1           0        4.392532    0.074000   -1.113615
     30          6           0        3.062668   -1.571246   -0.988243
     31          1           0        3.758480   -2.268875   -1.448271
     32          8           0       -1.955869    1.838591    1.582979
     33          8           0        4.003817    2.439374   -1.072126
     34          1           0        4.087709    3.395801   -1.176367
     35          6           0        1.792955   -1.261948    1.754855
     36          8           0        2.075897   -0.392590    2.552881
     37          8           0        1.994138   -2.587920    2.041902
     38          1           0        2.409748   -2.619714    2.919509
     39          6           0        1.105113   -1.078046    0.425675
     40          6           0        2.733456    2.121815   -0.669707
     41         13           0       -0.712714   -1.680298    0.591669
     42          6           0       -2.388977    2.214527    0.248654
 ---------------------------------------------------------------------

Cartesian coordinates of GON2-Al5
 ---------------------------------------------------------------------
 Center     Atomic      Atomic             Coordinates (Angstroms)
 Number     Number       Type             X           Y           Z
 ---------------------------------------------------------------------
      1          6           0       -3.510295    1.727096   -0.221666
      2          6           0       -3.175891    0.344623   -0.298310
      3          6           0       -0.423199    1.181122    0.527272
      4          6           0       -2.529065    2.726266   -0.085172
      5          6           0        1.043665    1.103348    0.409364
      6          6           0        1.614722   -0.181806    0.236437
      7          6           0        2.937811   -0.301885   -0.285638
      8          6           0        3.187083    2.130259   -0.106785
      9          6           0        1.816287    2.257873    0.218252
     10          6           0        1.147155    3.554045    0.192716
     11          6           0       -0.197469    3.699902    0.236990
     12          1           0       -0.637764    4.684597    0.100032
     13          1           0        1.769956    4.434181    0.049521
     14          1           0       -4.510472    2.079083   -0.497000
     15          1           0       -2.849152    3.760797   -0.178813
     16          1           0        3.798871    3.021973   -0.229682
     17          6           0       -3.959386   -0.771116   -0.729119
     18          6           0       -3.321868   -2.033975   -0.856708
     19          6           0       -1.951771   -2.161944   -0.514400
     20          1           0       -5.015652   -0.682493   -0.996899
     21          1           0       -3.899183   -2.875022   -1.248889
     22          6           0       -1.190135   -3.318112   -0.805122
     23          6           0        0.203535   -3.513021   -0.733486
     24          6           0        1.214048   -2.571594   -0.368116
     25          1           0       -1.724467   -4.162775   -1.252510
     26          1           0        0.566856   -4.449298   -1.154982
     27          6           0        3.394566   -1.563197   -0.786474
     28          1           0        4.405077   -1.628581   -1.173665
     29          6           0        2.533300   -2.629934   -0.908056
     30          1           0        2.842977   -3.520924   -1.448350
     31          8           0       -0.985414    1.877817    1.701570
     32          8           0        5.014381    0.717922   -0.811681
     33          1           0        5.442374    1.578393   -0.908156
     34          6           0        0.116482   -1.739777    1.647057
     35          8           0       -0.881556   -1.085074    2.079894
     36          8           0        0.454830   -2.855518    2.297734
     37          1           0       -0.232613   -3.031318    2.963618
     38          6           0        0.888131   -1.437974    0.452411
     39          6           0        3.722770    0.882559   -0.384859
     40         13           0       -1.516770   -0.448560    0.383926
     41          6           0       -1.120874    2.551757    0.435263
     42         13           0       -1.822641    1.367261   -1.906655
 ---------------------------------------------------------------------

Cartesian coordinates of GON2-Al6
 ---------------------------------------------------------------------
 Center     Atomic      Atomic             Coordinates (Angstroms)
 Number     Number       Type             X           Y           Z
 ---------------------------------------------------------------------
      1          6           0        4.321175   -0.885728   -0.441186
      2          6           0        3.416171    0.286877   -0.404963
      3          6           0        4.054630   -2.213938   -0.342202
      4          6           0       -0.868333   -1.121368    0.003486
      5          6           0       -1.597807    0.027922   -0.052453
      6          6           0       -2.948934    0.412403   -0.354795
      7          6           0       -2.629859   -2.640104   -0.810391
      8          6           0       -1.287192   -2.373575   -0.731742
      9          6           0       -0.356671   -3.331859   -1.393108
     10          6           0        0.970485   -3.598909   -1.283241
     11          1           0        1.296567   -4.356291   -2.002869
     12          1           0       -0.909048   -3.914737   -2.135158
     13          1           0        5.356541   -0.596467   -0.644072
     14          1           0        4.934407   -2.850271   -0.458047
     15          1           0       -2.899620   -3.606408   -1.243404
     16          6           0        3.943012    1.465494   -0.993042
     17          6           0        3.186540    2.614429   -1.183020
     18          6           0        1.836465    2.653616   -0.766583
     19          1           0        4.969787    1.454950   -1.352367
     20          1           0        3.631614    3.471387   -1.685267
     21          6           0        1.014591    3.809536   -1.137309
     22          6           0       -0.358338    4.001367   -1.096287
     23          6           0       -1.289419    3.050541   -0.564614
     24          1           0        1.573603    4.600900   -1.637012
     25          1           0       -0.737144    4.885265   -1.617090
     26          6           0       -3.367377    1.698274   -0.742536
     27          1           0       -4.417561    1.775144   -1.023138
     28          6           0       -2.614065    2.906599   -0.940868
     29          1           0       -3.128235    3.692008   -1.504387
     30          8           0        1.554018   -2.476278    1.506503
     31          8           0       -5.643762   -1.398697   -0.288638
     32          1           0       -6.155481   -2.207754   -0.375168
     33          6           0       -0.365446    1.131900    2.476044
     34          8           0        0.246395    0.028007    2.637110
     35          8           0       -0.773859    1.741453    3.575288
     36          1           0       -0.516795    1.212109    4.359314
     37         13           0        2.266659   -2.903237    0.001595
     38         13           0        0.872169   -0.939090    1.136092
     39         13           0       -0.509436    1.638784    0.494415
     40         13           0       -3.934606   -1.269630   -0.391871
     41          6           0        2.087084    0.333022    0.122655
     42          6           0        1.337759    1.512756   -0.071586
 ---------------------------------------------------------------------
