# Supplementary material for: Data of electronic, reactivity, optoelectronic, linear and non-linear optical parameters of doping graphene oxide nanosheet with aluminum atom
Source: Data Brief. 2022 Jan 19;41:107840. doi: 10.1016/j.dib.2022.107840 (PMC8801356; doi:10.1016/j.dib.2022.107840)
Supplement: Supplementary file 1 [file mmc1.zip › supplementary file/Cartesian coordinates/Cartesian coordinates OF GON2 AND ITS DERIVATIVES (GON2-Alx) wB97XD.rtf]

Cartesian coordinates of GON2
 ---------------------------------------------------------------------
 Center     Atomic      Atomic             Coordinates (Angstroms)
 Number     Number       Type             X           Y           Z
 ---------------------------------------------------------------------
      1          6           0       -3.658647    1.602026   -0.413860
      2          6           0       -3.051977    0.275228   -0.413242
      3          6           0       -1.678835    0.127191   -0.142041
      4          6           0       -0.871398    1.313222    0.235434
      5          6           0       -1.531316    2.660460    0.217173
      6          6           0       -2.958528    2.719890   -0.167975
      7          6           0       -1.063243   -1.115405   -0.237863
      8          6           0        0.582786    1.235265   -0.035613
      9          6           0        1.203425   -0.004066   -0.131000
     10          6           0        2.582683   -0.108344   -0.372139
     11          6           0        3.324826    1.082738   -0.504677
     12          6           0        2.707404    2.320713   -0.417719
     13          6           0        1.329396    2.414845   -0.194013
     14          6           0        0.655939    3.709110   -0.199868
     15          6           0       -0.672227    3.834048   -0.055068
     16          1           0       -1.151515    4.804891   -0.144865
     17          1           0        1.266149    4.590345   -0.379302
     18          1           0       -4.711297    1.668001   -0.675963
     19          1           0       -3.422636    3.698344   -0.254424
     20          1           0        3.293267    3.229717   -0.538007
     21          6           0       -3.794620   -0.862014   -0.756208
     22          6           0       -3.183898   -2.099403   -0.858455
     23          6           0       -1.809682   -2.248286   -0.613079
     24          1           0       -4.857017   -0.762632   -0.960726
     25          1           0       -3.766820   -2.970868   -1.142981
     26          6           0       -1.147056   -3.521462   -0.779326
     27          6           0        0.218532   -3.620823   -0.725181
     28          6           0        1.038607   -2.513470   -0.422057
     29          1           0       -1.749503   -4.387990   -1.034072
     30          1           0        0.701936   -4.561543   -0.975995
     31          6           0        3.202235   -1.402770   -0.511992
     32          1           0        4.275502   -1.450229   -0.649851
     33          6           0        2.438812   -2.541381   -0.577464
     34          1           0        2.911185   -3.491270   -0.813946
     35          8           0       -1.253812    1.967058    1.450024
     36          8           0        4.660557    0.952152   -0.732009
     37          1           0        5.072614    1.819078   -0.780174
     38          6           0        0.483017   -1.476162    1.711281
     39          8           0        1.359233   -1.042333    2.414619
     40          8           0       -0.512220   -2.245988    2.175061
     41          1           0       -0.361371   -2.357523    3.124173
     42          6           0        0.398671   -1.260387    0.159047
 ---------------------------------------------------------------------

Cartesian coordinates of GON2-Al1
 ---------------------------------------------------------------------
 Center     Atomic      Atomic             Coordinates (Angstroms)
 Number     Number       Type             X           Y           Z
 ---------------------------------------------------------------------
      1          6           0       -3.960224   -1.122741   -0.391159
      2          6           0       -2.630288   -1.704296   -0.548462
      3          6           0       -1.488088   -1.041109   -0.044130
      4          6           0       -1.614635    0.381536    0.501900
      5          6           0       -3.001295    0.939520    0.567180
      6          6           0       -4.142238    0.096577    0.128662
      7          6           0       -0.245247   -1.655782   -0.157915
      8          6           0       -0.500947    1.387674    0.222207
      9          6           0        0.820837    0.988544    0.111143
     10          6           0        1.804892    1.805826   -0.493864
     11          6           0        1.475453    3.158242   -0.750436
     12          6           0        0.187239    3.616234   -0.473705
     13          6           0       -0.818340    2.743597   -0.042000
     14          6           0       -2.184490    3.231709    0.116488
     15          6           0       -3.196175    2.402577    0.398504
     16          1           0       -4.212526    2.781259    0.471098
     17          1           0       -2.368245    4.293814   -0.021838
     18          1           0       -4.813103   -1.707925   -0.725177
     19          1           0       -5.139053    0.522924    0.205370
     20          1           0       -0.059938    4.660874   -0.652321
     21          6           0       -2.467818   -2.921472   -1.227617
     22          6           0       -1.204402   -3.435503   -1.496609
     23          6           0       -0.070155   -2.784057   -0.984796
     24          1           0       -3.348853   -3.436786   -1.600302
     25          1           0       -1.105359   -4.324439   -2.115388
     26          6           0        1.263982   -3.165677   -1.480850
     27          6           0        2.447781   -2.450584   -1.501640
     28          6           0        2.557656   -1.167729   -0.884417
     29          1           0        1.268724   -4.081671   -2.072917
     30          1           0        3.244572   -2.846558   -2.135212
     31          6           0        3.015329    1.238979   -1.058344
     32          1           0        3.670045    1.973982   -1.523582
     33          6           0        3.360075   -0.095083   -1.310982
     34          1           0        4.199318   -0.243224   -1.994595
     35          8           0       -2.304587    0.523518    1.753526
     36          8           0        2.422055    3.969762   -1.279823
     37          1           0        2.059675    4.845774   -1.442734
     38          6           0        2.128945   -1.157398    2.423238
     39          8           0        3.091859   -0.540627    2.842455
     40          8           0        1.562871   -2.129139    3.185865
     41          1           0        2.062961   -2.164757    4.018995
     42         13           0        1.318240   -0.815299    0.602926
 ---------------------------------------------------------------------

Cartesian coordinates of GON2-Al2
 ---------------------------------------------------------------------
 Center     Atomic      Atomic             Coordinates (Angstroms)
 Number     Number       Type             X           Y           Z
 ---------------------------------------------------------------------
      1          6           0       -3.365242    2.384507   -0.447744
      2          6           0       -3.059281    0.961951   -0.518509
      3          6           0       -1.757013    0.495600   -0.246402
      4          6           0       -0.676901    1.440673    0.156126
      5          6           0       -1.060384    2.875796    0.246873
      6          6           0       -2.440274    3.285452   -0.103714
      7          6           0       -1.479105   -0.860373   -0.297316
      8          6           0        0.752282    1.024385   -0.111165
      9          6           0        1.021818   -0.320162   -0.166641
     10          6           0        2.360965   -0.885378   -0.356022
     11          6           0        3.112307    2.034995   -0.333615
     12          6           0        1.754487    2.141574   -0.215071
     13          6           0        1.249976    3.528940   -0.178835
     14          6           0       -0.014126    3.890368    0.029103
     15          1           0       -0.303189    4.937584    0.039572
     16          1           0        2.002903    4.298075   -0.324306
     17          1           0       -4.375499    2.696638   -0.697969
     18          1           0       -2.668595    4.347323   -0.088853
     19          1           0        3.636520    2.988781   -0.374615
     20          6           0       -4.053019    0.044310   -0.865743
     21          6           0       -3.769331   -1.309054   -0.930572
     22          6           0       -2.487098   -1.779676   -0.638829
     23          1           0       -5.056189    0.402078   -1.079406
     24          1           0       -4.547127   -2.018130   -1.199329
     25          6           0       -2.191029   -3.202625   -0.691945
     26          6           0       -0.935136   -3.656325   -0.553769
     27          6           0        0.179114   -2.755340   -0.338500
     28          1           0       -3.008631   -3.888080   -0.894899
     29          1           0       -0.709214   -4.712743   -0.666655
     30          6           0        2.538977   -2.230119   -0.473625
     31          1           0        3.545069   -2.626259   -0.594000
     32          6           0        1.450020   -3.166327   -0.528135
     33          1           0        1.646609   -4.201173   -0.794872
     34          8           0       -0.908847    2.070717    1.433772
     35          8           0        5.467846   -0.287058   -0.563183
     36          1           0        6.274746    0.223523   -0.593912
     37          6           0       -0.108252   -1.468790    1.707632
     38          8           0        0.812332   -1.171052    2.425145
     39          8           0       -1.250249   -1.996878    2.172462
     40          1           0       -1.156428   -2.063160    3.133112
     41         13           0        3.864030    0.293931   -0.412370
     42          6           0       -0.104093   -1.329615    0.148317
 ---------------------------------------------------------------------

Cartesian coordinates of GON2-Al3
 ---------------------------------------------------------------------
 Center     Atomic      Atomic             Coordinates (Angstroms)
 Number     Number       Type             X           Y           Z
 ---------------------------------------------------------------------
      1          6           0       -3.584145   -1.606235   -0.894743
      2          6           0       -2.165652   -2.029222   -0.738083
      3          6           0       -1.156717   -1.299383   -0.067799
      4          6           0       -4.207701   -0.426206   -0.670546
      5          6           0        0.181607   -1.676768   -0.223355
      6          6           0       -0.111918    1.391308    0.152875
      7          6           0        1.142235    0.797733   -0.018790
      8          6           0        2.273511    1.548309   -0.412090
      9          6           0        0.781266    3.426273   -0.813649
     10          6           0       -0.319896    2.700484   -0.337390
     11          6           0       -1.638977    3.378574   -0.470255
     12          6           0       -2.905312    2.911428   -0.381876
     13          1           0       -3.651365    3.675296   -0.613776
     14          1           0       -1.519694    4.425334   -0.763108
     15          1           0       -4.183952   -2.399812   -1.348515
     16          1           0       -5.256699   -0.440072   -0.975720
     17          1           0        0.640180    4.439325   -1.187856
     18          6           0       -1.809958   -3.229309   -1.375089
     19          6           0       -0.506162   -3.693449   -1.352130
     20          6           0        0.522504   -2.926461   -0.787331
     21          1           0       -2.573421   -3.817979   -1.877815
     22          1           0       -0.261196   -4.653335   -1.798737
     23          6           0        1.876848   -3.422318   -0.766191
     24          6           0        2.912566   -2.603214   -0.423921
     25          6           0        2.715178   -1.242200   -0.099361
     26          1           0        2.062303   -4.440287   -1.095385
     27          1           0        3.936376   -2.954868   -0.522525
     28          6           0        3.602784    0.999686   -0.399986
     29          1           0        4.439717    1.659839   -0.592033
     30          6           0        3.791917   -0.345193   -0.239408
     31          1           0        4.788841   -0.764130   -0.349632
     32          8           0       -2.750339    0.734487    1.758694
     33          8           0        3.151955    3.598877   -1.186993
     34          1           0        2.890689    4.496877   -1.408377
     35          6           0        1.181243   -0.807326    1.828055
     36          8           0        0.131403   -0.445316    2.402964
     37          8           0        2.175645   -1.264788    2.543152
     38          1           0        1.919785   -1.250554    3.478716
     39          6           0        1.339879   -0.738872    0.317847
     40          6           0        2.056347    2.883160   -0.800017
     41         13           0       -3.446293    1.132503    0.228732
     42         13           0       -1.225212    0.182381    1.213701
 ---------------------------------------------------------------------

Cartesian coordinates of GON2-Al4
 ---------------------------------------------------------------------
 Center     Atomic      Atomic             Coordinates (Angstroms)
 Number     Number       Type             X           Y           Z
 ---------------------------------------------------------------------
      1          6           0       -4.077784    0.526754   -0.380579
      2          6           0       -3.133488   -0.567735   -0.139672
      3          6           0       -1.883314   -0.270536    0.322990
      4          6           0       -1.357491    1.098356    0.495178
      5          6           0       -3.720595    1.808340   -0.187783
      6          6           0        0.111083    1.365658    0.164009
      7          6           0        1.144960    0.398279    0.032071
      8          6           0        2.421338    0.750377   -0.456500
      9          6           0        1.746248    3.058371   -0.523501
     10          6           0        0.444345    2.706881   -0.157070
     11          6           0       -0.564484    3.760397   -0.255048
     12          6           0       -1.877187    3.534240   -0.138438
     13          1           0       -2.603066    4.317913   -0.337303
     14          1           0       -0.209970    4.752312   -0.522068
     15          1           0       -5.066556    0.295479   -0.768793
     16          1           0       -4.408349    2.609218   -0.448825
     17          1           0        1.969477    4.100728   -0.741301
     18          6           0       -3.545344   -1.939839   -0.537818
     19          6           0       -2.770251   -3.039301   -0.757759
     20          6           0       -1.324327   -3.087093   -0.504547
     21          1           0       -4.601959   -2.043098   -0.777645
     22          1           0       -3.269295   -3.926092   -1.150326
     23          6           0       -0.346070   -3.377291   -1.399990
     24          6           0        1.085390   -2.996250   -1.317007
     25          6           0        1.700374   -1.989172   -0.639119
     26          1           0       -0.604174   -3.826617   -2.364886
     27          1           0        1.716930   -3.516475   -2.036008
     28          6           0        3.408749   -0.282166   -0.795364
     29          1           0        4.408011    0.054165   -1.046613
     30          6           0        3.071175   -1.578405   -0.950665
     31          1           0        3.778307   -2.294087   -1.361040
     32          8           0       -1.941798    1.818093    1.584602
     33          8           0        3.983414    2.401859   -1.110361
     34          1           0        4.068397    3.352180   -1.225938
     35          6           0        1.796334   -1.274728    1.754034
     36          8           0        2.119708   -0.414183    2.539160
     37          8           0        1.957330   -2.594017    2.044623
     38          1           0        2.390104   -2.632432    2.908463
     39          6           0        1.098716   -1.078990    0.437710
     40          6           0        2.724913    2.096297   -0.690115
     41         13           0       -0.710223   -1.686476    0.579489
     42          6           0       -2.367323    2.208719    0.270772
 ---------------------------------------------------------------------

Cartesian coordinates of GON2-Al5
 ---------------------------------------------------------------------
 Center     Atomic      Atomic             Coordinates (Angstroms)
 Number     Number       Type             X           Y           Z
 ---------------------------------------------------------------------
      1          6           0       -1.952474    3.267336   -0.852055
      2          6           0       -2.319261    1.888456   -0.941526
      3          6           0        0.311399    1.485930    0.498527
      4          6           0       -0.773965    3.740940   -0.341515
      5          6           0        1.566406    0.722738    0.302917
      6          6           0        1.432741   -0.654681    0.075574
      7          6           0        2.518926   -1.432845   -0.344587
      8          6           0        3.924824    0.549868   -0.218173
      9          6           0        2.802236    1.340579    0.089782
     10          6           0        2.840787    2.796199    0.007084
     11          6           0        1.719507    3.541157   -0.002194
     12          1           0        1.786129    4.607609   -0.204882
     13          1           0        3.804462    3.271428   -0.157574
     14          1           0       -2.661082    4.021915   -1.204532
     15          1           0       -0.628633    4.820574   -0.345375
     16          1           0        4.894585    1.024025   -0.356989
     17          6           0       -3.559733    1.399097   -1.406135
     18          6           0       -3.774299    0.025184   -1.345520
     19          6           0       -2.786822   -0.823047   -0.648046
     20          1           0       -4.327505    2.044906   -1.836932
     21          1           0       -4.641533   -0.420482   -1.833249
     22          6           0       -2.529544   -2.116334   -1.138633
     23          6           0       -1.405519   -2.947501   -0.948877
     24          6           0       -0.148463   -2.524125   -0.525282
     25          1           0       -3.253305   -2.521578   -1.852616
     26          1           0       -1.454097   -3.929196   -1.413506
     27          6           0        2.283553   -2.800768   -0.734822
     28          1           0        3.136350   -3.413589   -1.006343
     29          6           0        1.023585   -3.295609   -0.863929
     30          1           0        0.875005   -4.293039   -1.268622
     31          8           0        0.210431    2.442690    1.565419
     32          8           0        4.829405   -1.603978   -0.804647
     33          1           0        5.634386   -1.081606   -0.861170
     34          6           0       -0.300405   -1.369018    1.640528
     35          8           0        0.336941   -1.021906    2.602156
     36          8           0       -1.570434   -1.922538    1.870803
     37          1           0       -1.606663   -2.047567    2.832427
     38          6           0        0.084913   -1.275538    0.215967
     39          6           0        3.784729   -0.810433   -0.439283
     40         13           0       -3.609693   -1.023780    1.275881
     41          6           0        0.371023    2.970743    0.236717
     42         13           0       -1.212900    0.461664   -0.218927
 ---------------------------------------------------------------------

 Cartesian coordinates of GON2-Al6
 ---------------------------------------------------------------------
 Center     Atomic      Atomic             Coordinates (Angstroms)
 Number     Number       Type             X           Y           Z
 ---------------------------------------------------------------------
      1          6           0        4.358006   -0.861189   -0.294421
      2          6           0        3.416090    0.289451   -0.339516
      3          6           0        4.102404   -2.183678   -0.214134
      4          6           0       -0.869326   -1.075541   -0.003633
      5          6           0       -1.650841    0.022496   -0.043518
      6          6           0       -2.995289    0.399292   -0.338297
      7          6           0       -2.551363   -2.613636   -0.893835
      8          6           0       -1.231032   -2.294058   -0.825965
      9          6           0       -0.256590   -3.145742   -1.567374
     10          6           0        1.060423   -3.404179   -1.418550
     11          1           0        1.438187   -4.065967   -2.203153
     12          1           0       -0.766529   -3.637032   -2.399835
     13          1           0        5.399724   -0.546641   -0.402052
     14          1           0        4.990830   -2.815646   -0.241205
     15          1           0       -2.787761   -3.555563   -1.393061
     16          6           0        3.923389    1.447970   -0.959777
     17          6           0        3.148704    2.576410   -1.185633
     18          6           0        1.808167    2.606741   -0.771540
     19          1           0        4.953873    1.446428   -1.307869
     20          1           0        3.583410    3.427038   -1.706467
     21          6           0        0.981260    3.774354   -1.147844
     22          6           0       -0.373129    3.969243   -1.109721
     23          6           0       -1.316087    2.999485   -0.596218
     24          1           0        1.550768    4.570178   -1.627373
     25          1           0       -0.752318    4.873006   -1.591492
     26          6           0       -3.399855    1.664733   -0.766671
     27          1           0       -4.449081    1.748265   -1.046316
     28          6           0       -2.619813    2.854821   -0.998683
     29          1           0       -3.111997    3.623955   -1.600636
     30          8           0        1.488898   -2.500275    1.478999
     31          8           0       -5.583265   -1.555454   -0.159260
     32          1           0       -6.059358   -2.382407   -0.225102
     33          6           0       -0.410502    1.114811    2.475796
     34          8           0        0.203228    0.022508    2.657293
     35          8           0       -0.836328    1.727971    3.555964
     36          1           0       -0.585215    1.208649    4.341632
     37         13           0        2.283756   -2.827912   -0.008920
     38         13           0        0.854189   -0.936796    1.159100
     39         13           0       -0.525697    1.621371    0.489428
     40         13           0       -3.900651   -1.323627   -0.360727
     41          6           0        2.084929    0.314718    0.154702
     42          6           0        1.317862    1.479877   -0.069580
 ---------------------------------------------------------------------
