# Supplementary material for: Data of electronic, reactivity, optoelectronic, linear and non-linear optical parameters of doping graphene oxide nanosheet with aluminum atom
Source: Data Brief. 2022 Jan 19;41:107840. doi: 10.1016/j.dib.2022.107840 (PMC8801356; doi:10.1016/j.dib.2022.107840)
Supplement: Supplementary file 1 [file mmc1.zip › supplementary file/DATA OF THE UV-VIS SPECTRA/DATA OF THE UV-VIS SPECTRA OF GON1 AND ITS DERIVATIVES (GON1-Alx) B3LYP.docx]

**Data of the UV-Vis spectra of GON1 isomer and its aluminum-doped derivatives (GON1-Alx), computed at the B3LYP/6-31+g(d,p) level of theory**

| **GON1** | | **GON1-Al1** | | **GON1-Al2** | |
| --- | --- | --- | --- | --- | --- |
| Wavelength (nm) | Abs | Wavelength (nm) | Abs | Wavelength (nm) | Abs |
| 2000.0 | 3.10713775202e-06 | 2000.0 | 1.29707551427e-11 | 2000.0 | 2.45784461627 |
| 1977.5873434410018 | 4.10087849743e-06 | 1977.5873434410018 | 1.87066937621e-11 | 1977.5873434410018 | 2.83591074588 |
| 1955.671447196871 | 5.40170184884e-06 | 1955.671447196871 | 2.69256456095e-11 | 1955.671447196871 | 3.26563896168 |
| 1934.2359767891683 | 7.10103461256e-06 | 1934.2359767891683 | 3.86787586151e-11 | 1934.2359767891683 | 3.75302341837 |
| 1913.265306122449 | 9.31643938645e-06 | 1913.265306122449 | 5.54518757833e-11 | 1913.265306122449 | 4.30459102473 |
| 1892.7444794952683 | 1.21987578068e-05 | 1892.7444794952683 | 7.9340926813e-11 | 1892.7444794952683 | 4.92742538845 |
| 1872.6591760299625 | 1.59411103492e-05 | 1872.6591760299625 | 1.13296269453e-10 | 1872.6591760299625 | 5.62918839721 |
| 1852.9956763434218 | 2.07902082826e-05 | 1852.9956763434218 | 1.61462347909e-10 | 1852.9956763434218 | 6.41813881644 |
| 1833.7408312958437 | 2.70605379159e-05 | 1833.7408312958437 | 2.29648826243e-10 | 1833.7408312958437 | 7.30314723859 |
| 1814.8820326678765 | 3.51521039169e-05 | 1814.8820326678765 | 3.25982667734e-10 | 1814.8820326678765 | 8.29370667855 |
| 1796.4071856287424 | 4.55725714491e-05 | 1796.4071856287424 | 4.61808691199e-10 | 1796.4071856287424 | 9.39993807502 |
| 1778.3046828689983 | 5.8964831053e-05 | 1778.3046828689983 | 6.52930577357e-10 | 1778.3046828689983 | 10.6325899317 |
| 1760.5633802816901 | 7.61412312837e-05 | 1760.5633802816901 | 9.21317299467e-10 | 1760.5633802816901 | 12.0030313149 |
| 1743.1725740848342 | 9.81259886684e-05 | 1743.1725740848342 | 1.29744440433e-09 | 1743.1725740848342 | 13.523237418 |
| 1726.1219792865363 | 0.000126207600091 | 1726.1219792865363 | 1.82349930331e-09 | 1726.1219792865363 | 15.2057669114 |
| 1709.4017094017095 | 0.000162003457861 | 1709.4017094017095 | 2.55775981028e-09 | 1709.4017094017095 | 17.0637303153 |
| 1693.002257336343 | 0.000207539312276 | 1693.002257336343 | 3.58056225685e-09 | 1693.002257336343 | 19.110748672 |
| 1676.9144773616547 | 0.000265346751491 | 1676.9144773616547 | 5.00241839533e-09 | 1676.9144773616547 | 21.3609018476 |
| 1661.1295681063123 | 0.000338582486443 | 1661.1295681063123 | 6.97503046734e-09 | 1661.1295681063123 | 23.8286658644 |
| 1645.6390565002741 | 0.000431173953338 | 1645.6390565002741 | 9.70620631015e-09 | 1645.6390565002741 | 26.5288387592 |
| 1630.4347826086955 | 0.000547996593239 | 1630.4347826086955 | 1.34800108089e-08 | 1630.4347826086955 | 29.4764545707 |
| 1615.5088852988692 | 0.00069508915466 | 1615.5088852988692 | 1.86839318976e-08 | 1615.5088852988692 | 32.6866851952 |
| 1600.8537886872998 | 0.000879914509405 | 1600.8537886872998 | 2.58454217702e-08 | 1600.8537886872998 | 36.1747299965 |
| 1586.4621893178212 | 0.00111167479443 | 1586.4621893178212 | 3.56809398162e-08 | 1586.4621893178212 | 39.9556932323 |
| 1572.3270440251572 | 0.00140169121511 | 1572.3270440251572 | 4.91616283187e-08 | 1572.3270440251572 | 44.0444495436 |
| 1558.4415584415583 | 0.001763860591 | 1558.4415584415583 | 6.76010662721e-08 | 1558.4415584415583 | 48.455497966 |
| 1544.799176107106 | 0.00221520271874 | 1544.799176107106 | 9.27722620775e-08 | 1544.799176107106 | 53.2028051388 |
| 1531.3935681470139 | 0.00277651489311 | 1531.3935681470139 | 1.2706327919e-07 | 1531.3935681470139 | 58.2996386252 |
| 1518.2186234817814 | 0.00347315249272 | 1518.2186234817814 | 1.73683789125e-07 | 1518.2186234817814 | 63.7583915005 |
| 1505.2684395383842 | 0.00433595742779 | 1505.2684395383842 | 2.36938603372e-07 | 1505.2684395383842 | 69.590399613 |
| 1492.5373134328358 | 0.00540235948819 | 1492.5373134328358 | 3.22589090542e-07 | 1492.5373134328358 | 75.8057531769 |
| 1480.0197335964478 | 0.00671767924692 | 1480.0197335964478 | 4.38329642297e-07 | 1480.0197335964478 | 82.413104604 |
| 1467.7103718199608 | 0.00833666518733 | 1467.7103718199608 | 5.94414395987e-07 | 1467.7103718199608 | 89.4194747218 |
| 1455.604075691412 | 0.0103253021519 | 1455.604075691412 | 8.04479737255e-07 | 1455.604075691412 | 96.8300597563 |
| 1443.6958614051973 | 0.0127629330709 | 1443.6958614051973 | 1.08662129957e-06 | 1443.6958614051973 | 104.648041668 |
| 1431.9809069212408 | 0.0157447412271 | 1431.9809069212408 | 1.46480100912e-06 | 1431.9809069212408 | 112.874404616 |
| 1420.4545454545455 | 0.0193846460515 | 1420.4545454545455 | 1.97068113996e-06 | 1420.4545454545455 | 121.507760484 |
| 1409.1122592766555 | 0.0238186716134 | 1409.1122592766555 | 2.64600952393e-06 | 1409.1122592766555 | 130.544186522 |
| 1397.9496738117427 | 0.0292088535472 | 1397.9496738117427 | 3.54571446223e-06 | 1397.9496738117427 | 139.977078248 |
| 1386.9625520110958 | 0.035747757111 | 1386.9625520110958 | 4.74191131605e-06 | 1386.9625520110958 | 149.797020791 |
| 1376.1467889908256 | 0.0436636863535 | 1376.1467889908256 | 6.32907743417e-06 | 1376.1467889908256 | 159.991681824 |
| 1365.4984069185252 | 0.0532266718996 | 1365.4984069185252 | 8.43072073772e-06 | 1365.4984069185252 | 170.54572923 |
| 1355.0135501355014 | 0.064755332563 | 1355.0135501355014 | 1.12079532671e-05 | 1355.0135501355014 | 181.440776439 |
| 1344.688480502017 | 0.078624713745 | 1344.688480502017 | 1.48704883828e-05 | 1344.688480502017 | 192.655358296 |
| 1334.5195729537365 | 0.0952752132333 | 1334.5195729537365 | 1.9690714061e-05 | 1334.5195729537365 | 204.164939997 |
| 1324.5033112582782 | 0.115222712407 | 1324.5033112582782 | 2.60216608447e-05 | 1324.5033112582782 | 215.941961396 |
| 1314.6362839614374 | 0.13907003778 | 1314.6362839614374 | 3.4319888773e-05 | 1314.6362839614374 | 227.955918614 |
| 1304.9151805132665 | 0.167519884036 | 1304.9151805132665 | 4.51745717483e-05 | 1304.9151805132665 | 240.173484466 |
| 1295.3367875647668 | 0.201389334951 | 1295.3367875647668 | 5.93443708074e-05 | 1295.3367875647668 | 252.558668797 |
| 1285.8979854264894 | 0.241626122579 | 1285.8979854264894 | 7.78040721793e-05 | 1285.8979854264894 | 265.073019274 |
| 1276.5957446808509 | 0.289326767377 | 1276.5957446808509 | 0.000101803436758 | 1276.5957446808509 | 277.675862686 |
| 1267.427122940431 | 0.345756742319 | 1267.427122940431 | 0.000132941282418 | 1267.427122940431 | 290.324586189 |
| 1258.3892617449665 | 0.412372801906 | 1258.3892617449665 | 0.000173258520387 | 1258.3892617449665 | 302.974957366 |
| 1249.4793835901708 | 0.490847612056 | 1249.4793835901708 | 0.000225354716305 | 1249.4793835901708 | 315.58148134 |
| 1240.6947890818858 | 0.583096808529 | 1240.6947890818858 | 0.000292533774554 | 1240.6947890818858 | 328.097792579 |
| 1232.0328542094455 | 0.691308599419 | 1232.0328542094455 | 0.000378985584649 | 1232.0328542094455 | 340.477078402 |
| 1223.4910277324632 | 0.817976010792 | 1223.4910277324632 | 0.000490011960214 | 1223.4910277324632 | 352.67253063 |
| 1215.0668286755772 | 0.965931853194 | 1215.0668286755772 | 0.000632306989694 | 1215.0668286755772 | 364.637821229 |
| 1206.7578439259853 | 1.13838646016 | 1206.7578439259853 | 0.000814304055738 | 1206.7578439259853 | 376.327597306 |
| 1198.5617259288852 | 1.33896821729 | 1198.5617259288852 | 0.00104660432727 | 1198.5617259288852 | 387.697990319 |
| 1190.4761904761904 | 1.57176686183 | 1190.4761904761904 | 0.0013425045528 | 1190.4761904761904 | 398.707133968 |
| 1182.4990145841543 | 1.84137948719 | 1182.4990145841543 | 0.00171864556349 | 1182.4990145841543 | 409.3156849 |
| 1174.6280344557556 | 2.15295913449 | 1174.6280344557556 | 0.0021958071172 | 1174.6280344557556 | 419.487340089 |
| 1166.8611435239206 | 2.51226579382 | 1166.8611435239206 | 0.00279987967873 | 1166.8611435239206 | 429.189344607 |
| 1159.19629057187 | 2.92571957083 | 1159.19629057187 | 0.00356304954655 | 1159.19629057187 | 438.39298341 |
| 1151.6314779270633 | 3.40045570041 | 1151.6314779270633 | 0.00452524052347 | 1151.6314779270633 | 447.074050815 |
| 1144.1647597254005 | 3.94438100797 | 1144.1647597254005 | 0.00573586322234 | 1144.1647597254005 | 455.213291445 |
| 1136.794240242516 | 4.56623133087 | 1136.794240242516 | 0.00725593224292 | 1136.794240242516 | 462.796806683 |
| 1129.5180722891566 | 5.27562931905 | 1129.5180722891566 | 0.00916062201156 | 1129.5180722891566 | 469.816421002 |
| 1122.334455667789 | 6.08314193422 | 1122.334455667789 | 0.0115423442093 | 1122.334455667789 | 476.270002977 |
| 1115.2416356877322 | 7.00033686385 | 1115.2416356877322 | 0.0145144436075 | 1115.2416356877322 | 482.16173633 |
| 1108.2379017362393 | 8.03983695988 | 1108.2379017362393 | 0.0182156249691 | 1108.2379017362393 | 487.502336982 |
| 1101.3215859030836 | 9.21537170427 | 1101.3215859030836 | 0.0228152416567 | 1101.3215859030836 | 492.309212789 |
| 1094.4910616563297 | 10.5418245966 | 1094.4910616563297 | 0.0285195969097 | 1094.4910616563297 | 496.606563434 |
| 1087.7447425670775 | 12.0352752543 | 1087.7447425670775 | 0.0355794316088 | 1087.7447425670775 | 500.425418772 |
| 1081.081081081081 | 13.7130349169 | 1081.081081081081 | 0.0442987979367 | 1081.081081081081 | 503.803614824 |
| 1074.4985673352435 | 15.5936739539 | 1074.4985673352435 | 0.0550455468346 | 1074.4985673352435 | 506.785707521 |
| 1067.995728017088 | 17.697039895 | 1067.995728017088 | 0.0682636887185 | 1067.995728017088 | 509.422825252 |
| 1061.5711252653928 | 20.0442644332 | 1061.5711252653928 | 0.0844879216819 | 1061.5711252653928 | 511.772462192 |
| 1055.2233556102708 | 22.6577578029 | 1055.2233556102708 | 0.104360659471 | 1055.2233556102708 | 513.898215298 |
| 1048.951048951049 | 25.5611889005 | 1048.951048951049 | 0.128651932909 | 1048.951048951049 | 515.86946878 |
| 1042.752867570386 | 28.7794495129 | 1042.752867570386 | 0.158282583173 | 1042.752867570386 | 517.761030632 |
| 1036.6275051831374 | 32.3386010344 | 1036.6275051831374 | 0.194351213259 | 1036.6275051831374 | 519.65272663 |
| 1030.5736860185502 | 36.2658021059 | 1030.5736860185502 | 0.238165414933 | 1030.5736860185502 | 521.628957846 |
| 1024.5901639344263 | 40.5892156883 | 1024.5901639344263 | 0.291277842196 | 1024.5901639344263 | 523.77822832 |
| 1018.6757215619693 | 45.3378942037 | 1018.6757215619693 | 0.355527758255 | 1018.6757215619693 | 526.192650015 |
| 1012.829169480081 | 50.5416415292 | 1012.829169480081 | 0.433088740735 | 1012.829169480081 | 528.967432486 |
| 1007.0493454179255 | 56.2308508257 | 1007.0493454179255 | 0.526523288506 | 1007.0493454179255 | 532.200364941 |
| 1001.3351134846461 | 62.4363174155 | 1001.3351134846461 | 0.638845132241 | 1001.3351134846461 | 535.991298397 |
| 995.6853634251576 | 69.1890261986 | 995.6853634251576 | 0.773590108308 | 995.6853634251576 | 540.441635545 |
| 990.0990099009902 | 76.519913414 | 990.0990099009902 | 0.934896510706 | 990.0990099009902 | 545.653835697 |
| 984.5749917952082 | 84.459602903 | 984.5749917952082 | 1.12759588658 | 984.5749917952082 | 551.730941774 |
| 979.1122715404699 | 93.0381174237 | 979.1122715404699 | 1.35731528574 | 979.1122715404699 | 558.776135698 |
| 973.7098344693281 | 102.284565988 | 973.7098344693281 | 1.63059201123 | 973.7098344693281 | 566.892327875 |
| 968.3666881859263 | 112.226808646 | 968.3666881859263 | 1.95500194398 | 968.3666881859263 | 576.181785534 |
| 963.0818619582664 | 122.891100606 | 963.0818619582664 | 2.33930252741 | 963.0818619582664 | 586.745803722 |
| 957.8544061302682 | 134.301718098 | 957.8544061302682 | 2.79359149387 | 957.8544061302682 | 598.684421615 |
| 952.6833915528738 | 146.480568845 | 952.6833915528738 | 3.32948239193 | 952.6833915528738 | 612.096185539 |
| 947.5679090334806 | 159.446790552 | 947.5679090334806 | 3.96029792719 | 947.5679090334806 | 627.077958832 |
| 942.5070688030161 | 173.216341283 | 942.5070688030161 | 4.70128205625 | 942.5070688030161 | 643.724777233 |
| 937.4999999999999 | 187.801586086 | 937.4999999999999 | 5.56983167053 | 937.4999999999999 | 662.129747108 |
| 932.5458501709667 | 203.210884659 | 932.5458501709667 | 6.58574856875 | 932.5458501709667 | 682.383982337 |
| 927.643784786642 | 219.448185277 | 927.643784786642 | 7.77151224146 | 927.643784786642 | 704.576574327 |
| 922.7929867733004 | 236.512630506 | 922.7929867733004 | 9.15257377325 | 922.7929867733004 | 728.794588191 |
| 917.9926560587514 | 254.398180591 | 917.9926560587514 | 10.7576709054 | 917.9926560587514 | 755.123076882 |
| 913.2420091324201 | 273.093260552 | 913.2420091324201 | 12.6191639906 | 913.2420091324201 | 783.645103891 |
| 908.5402786190186 | 292.5804372 | 908.5402786190186 | 14.7733922068 | 908.5402786190186 | 814.441764091 |
| 903.8867128653209 | 312.836132336 | 903.8867128653209 | 17.2610489838 | 903.8867128653209 | 847.592191482 |
| 899.2805755395683 | 333.830378297 | 899.2805755395683 | 20.1275751209 | 899.2805755395683 | 883.173541938 |
| 894.7211452430658 | 355.526621912 | 894.7211452430658 | 23.423567551 | 894.7211452430658 | 921.260938703 |
| 890.2077151335311 | 377.881582603 | 890.2077151335311 | 27.2052011215 | 890.2077151335311 | 961.927368243 |
| 885.7395925597874 | 400.845170034 | 885.7395925597874 | 31.5346601319 | 885.7395925597874 | 1005.24351423 |
| 881.316098707403 | 424.360466178 | 881.316098707403 | 36.4805756812 | 881.316098707403 | 1051.27751793 |
| 876.9365682548962 | 448.363776067 | 876.9365682548962 | 42.1184641533 | 876.9365682548962 | 1100.09465406 |
| 872.6003490401396 | 472.78475081 | 872.6003490401396 | 48.5311614014 | 872.6003490401396 | 1151.7569123 |
| 868.3068017366135 | 497.546585583 | 868.3068017366135 | 55.8092464 | 868.3068017366135 | 1206.3224761 |
| 864.0552995391705 | 522.566294449 | 864.0552995391705 | 64.0514473207 | 864.0552995391705 | 1263.84509225 |
| 859.8452278589854 | 547.755062829 | 859.8452278589854 | 73.3650221719 | 859.8452278589854 | 1324.37332662 |
| 855.6759840273816 | 573.018677401 | 855.6759840273816 | 83.8661053332 | 855.6759840273816 | 1387.94970407 |
| 851.5469770082316 | 598.258032114 | 851.5469770082316 | 95.6800105362 | 851.5469770082316 | 1454.60973292 |
| 847.457627118644 | 623.369707825 | 847.457627118644 | 108.941480108 | 847.457627118644 | 1524.38081734 |
| 843.4073657576608 | 648.246621929 | 843.4073657576608 | 123.794869621 | 843.4073657576608 | 1597.281064 |
| 839.3956351426972 | 672.778743212 | 839.3956351426972 | 140.394256521 | 839.3956351426972 | 1673.31799238 |
| 835.421888053467 | 696.853866006 | 835.421888053467 | 158.903460821 | 835.421888053467 | 1752.48716137 |
| 831.4855875831485 | 720.358436663 | 831.4855875831485 | 179.495965628 | 831.4855875831485 | 1834.77072798 |
| 827.5862068965516 | 743.178424375 | 827.5862068965516 | 202.354725105 | 827.5862068965516 | 1920.13595695 |
| 823.7232289950576 | 765.200227426 | 823.7232289950576 | 227.671847462 | 823.7232289950576 | 2008.53370316 |
| 819.8961464881114 | 786.311605184 | 819.8961464881114 | 255.648140822 | 819.8961464881114 | 2099.89689127 |
| 816.1044613710554 | 806.402625463 | 816.1044613710554 | 286.492510266 | 816.1044613710554 | 2194.13901955 |
| 812.3476848090983 | 825.366616364 | 812.3476848090983 | 320.421195056 | 812.3476848090983 | 2291.15271703 |
| 808.6253369272237 | 843.101111345 | 808.6253369272237 | 357.656836068 | 808.6253369272237 | 2390.80838428 |
| 804.9369466058491 | 859.50877607 | 804.9369466058491 | 398.427364703 | 804.9369466058491 | 2492.95294983 |
| 801.2820512820513 | 874.498305594 | 801.2820512820513 | 442.964706168 | 801.2820512820513 | 2597.40877428 |
| 797.6601967561818 | 887.985280607 | 797.6601967561818 | 491.503291888 | 797.6601967561818 | 2703.97273454 |
| 794.0709370037056 | 899.892971825 | 794.0709370037056 | 544.278378004 | 794.0709370037056 | 2812.41551945 |
| 790.5138339920949 | 910.153082161 | 790.5138339920949 | 601.524169433 | 790.5138339920949 | 2922.48116729 |
| 786.9884575026232 | 918.706417054 | 786.9884575026232 | 663.47175173 | 786.9884575026232 | 3033.88687312 |
| 783.4943849569078 | 925.503474175 | 783.4943849569078 | 730.346836066 | 783.4943849569078 | 3146.3230916 |
| 780.0312012480499 | 930.504944846 | 780.0312012480499 | 802.367325926 | 780.0312012480499 | 3259.45395734 |
| 776.598498576236 | 933.682120633 | 776.598498576236 | 879.740717622 | 776.598498576236 | 3372.91804105 |
| 773.1958762886597 | 935.017199902 | 773.1958762886597 | 962.661350402 | 773.1958762886597 | 3486.32945516 |
| 769.8229407236336 | 934.503490531 | 769.8229407236336 | 1051.30752567 | 769.8229407236336 | 3599.27931757 |
| 766.4793050587633 | 932.145506424 | 766.4793050587633 | 1145.83851866 | 766.4793050587633 | 3711.33757671 |
| 763.1645891630628 | 927.958956994 | 763.1645891630628 | 1246.39150975 | 763.1645891630628 | 3822.05519542 |
| 759.8784194528876 | 921.970630332 | 759.8784194528876 | 1353.07846618 | 759.8784194528876 | 3930.96668493 |
| 756.6204287515762 | 914.218172286 | 756.6204287515762 | 1465.98300863 | 756.6204287515762 | 4037.59297435 |
| 753.390256152687 | 904.749765174 | 753.390256152687 | 1585.15730032 | 753.390256152687 | 4141.44459455 |
| 750.1875468867216 | 893.623711284 | 750.1875468867216 | 1710.61899925 | 750.1875468867216 | 4242.02514983 |
| 747.011952191235 | 880.907927673 | 747.011952191235 | 1842.34831668 | 747.011952191235 | 4338.83504426 |
| 743.86312918423 | 866.679359975 | 743.86312918423 | 1980.28522722 | 743.86312918423 | 4431.37542483 |
| 740.7407407407408 | 851.023324082 | 740.7407407407408 | 2124.32687702 | 740.7407407407408 | 4519.15229816 |
| 737.6444553725104 | 834.032785464 | 737.6444553725104 | 2274.3252377 | 737.6444553725104 | 4601.68077326 |
| 734.5739471106758 | 815.807586728 | 734.5739471106758 | 2430.08505347 | 734.5739471106758 | 4678.48937919 |
| 731.528895391368 | 796.453634608 | 731.528895391368 | 2591.36212852 | 731.528895391368 | 4749.12440373 |
| 728.5089849441475 | 776.082058006 | 728.5089849441475 | 2757.86199995 | 728.5089849441475 | 4813.15419675 |
| 725.5139056831922 | 754.808348964 | 725.5139056831922 | 2929.23903947 | 725.5139056831922 | 4870.17338142 |
| 722.543352601156 | 732.751498482 | 722.543352601156 | 3105.09602367 | 722.543352601156 | 4919.80691571 |
| 719.5970256656271 | 710.033138966 | 719.5970256656271 | 3284.98420884 | 719.5970256656271 | 4961.71394801 |
| 716.6746297181079 | 686.776704766 | 716.6746297181079 | 3468.40394117 | 716.6746297181079 | 4995.59141213 |
| 713.7758743754462 | 663.106621803 | 713.7758743754462 | 3654.80582765 | 713.7758743754462 | 5021.17731001 |
| 710.9004739336492 | 639.147536577 | 710.9004739336492 | 3843.59248643 | 710.9004739336492 | 5038.2536341 |
| 708.0481472740146 | 615.023594111 | 708.0481472740146 | 4034.12088843 | 708.0481472740146 | 5046.64888599 |
| 705.2186177715091 | 590.857773417 | 705.2186177715091 | 4225.70529388 | 705.2186177715091 | 5046.24015333 |
| 702.4116132053383 | 566.771288058 | 702.4116132053383 | 4417.62077992 | 702.4116132053383 | 5036.95471318 |
| 699.6268656716418 | 542.883058216 | 699.6268656716418 | 4609.1073461 | 699.6268656716418 | 5018.77113649 |
| 696.8641114982578 | 519.309259522 | 696.8641114982578 | 4799.3745765 | 696.8641114982578 | 4991.71987574 |
| 694.1230911614992 | 496.162952601 | 694.1230911614992 | 4987.60682797 | 694.1230911614992 | 4955.88332524 |
| 691.4035492048858 | 473.553796021 | 691.4035492048858 | 5172.96890536 | 691.4035492048858 | 4911.395351 |
| 688.7052341597796 | 451.587844047 | 688.7052341597796 | 5354.61217621 | 688.7052341597796 | 4858.44029522 |
| 686.027898467871 | 430.367429281 | 686.027898467871 | 5531.68106911 | 686.027898467871 | 4797.25146781 |
| 683.371298405467 | 409.991129024 | 683.371298405467 | 5703.31989264 | 683.371298405467 | 4728.10914467 |
| 680.7351940095303 | 390.553812963 | 680.7351940095303 | 5868.6799048 | 680.7351940095303 | 4651.33809985 |
| 678.1193490054249 | 372.146768596 | 678.1193490054249 | 6026.92655725 | 678.1193490054249 | 4567.30470473 |
| 675.5235307363206 | 354.857899728 | 675.5235307363206 | 6177.2468334 | 675.5235307363206 | 4476.41363375 |
| 672.9475100942127 | 338.771992306 | 672.9475100942127 | 6318.85659603 | 672.9475100942127 | 4379.10422111 |
| 670.3910614525139 | 323.971040935 | 670.3910614525139 | 6451.00785728 | 670.3910614525139 | 4275.84651724 |
| 667.8539626001781 | 310.534628549 | 667.8539626001781 | 6572.9958828 | 667.8539626001781 | 4167.13709769 |
| 665.335994677312 | 298.540350958 | 665.335994677312 | 6684.16604193 | 665.335994677312 | 4053.49467928 |
| 662.8369421122403 | 288.064277296 | 662.8369421122403 | 6783.92031719 | 662.8369421122403 | 3935.45560034 |
| 660.3565925599823 | 279.18143689 | 660.3565925599823 | 6871.72338952 | 660.3565925599823 | 3813.56922232 |
| 657.8947368421053 | 271.966322525 | 657.8947368421053 | 6947.1082195 | 657.8947368421053 | 3688.39331023 |
| 655.4511688879178 | 266.493399796 | 655.4511688879178 | 7009.68105084 | 655.4511688879178 | 3560.48944756 |
| 653.0256856769699 | 262.837611907 | 653.0256856769699 | 7059.1257688 | 653.0256856769699 | 3430.41854005 |
| 650.6180871828237 | 261.074869151 | 650.6180871828237 | 7095.20755423 | 650.6180871828237 | 3298.73645932 |
| 648.2281763180639 | 261.282512207 | 648.2281763180639 | 7117.77578267 | 648.2281763180639 | 3165.98987397 |
| 645.8557588805166 | 263.539738428 | 645.8557588805166 | 7126.76612783 | 645.8557588805166 | 3032.71231173 |
| 643.5006435006435 | 267.927980414 | 643.5006435006435 | 7122.20183876 | 643.5006435006435 | 2899.42049113 |
| 641.1626415900834 | 274.531226362 | 641.1626415900834 | 7104.1941713 | 641.1626415900834 | 2766.6109564 |
| 638.8415672913118 | 283.436272021 | 638.8415672913118 | 7072.9419649 | 638.8415672913118 | 2634.75704335 |
| 636.5372374283895 | 294.732894456 | 636.5372374283895 | 7028.73036785 | 636.5372374283895 | 2504.30619862 |
| 634.2494714587738 | 308.513938376 | 634.2494714587738 | 6971.92872437 | 634.2494714587738 | 2375.67766866 |
| 631.9780914261638 | 324.875306341 | 631.9780914261638 | 6902.98764832 | 631.9780914261638 | 2249.26056886 |
| 629.7229219143577 | 343.915844938 | 629.7229219143577 | 6822.43531848 | 629.7229219143577 | 2125.41233785 |
| 627.4837900020916 | 365.737119802 | 627.4837900020916 | 6730.87303987 | 627.4837900020916 | 2004.45757603 |
| 625.2605252188412 | 390.443073332 | 625.2605252188412 | 6628.97012494 | 625.2605252188412 | 1886.68726252 |
| 623.0529595015576 | 418.139560011 | 623.0529595015576 | 6517.45815595 | 623.0529595015576 | 1772.35833957 |
| 620.8609271523178 | 448.933755418 | 620.8609271523178 | 6397.12469699 | 620.8609271523178 | 1661.69364921 |
| 618.6842647968654 | 482.933436362 | 618.6842647968654 | 6268.80652997 | 618.6842647968654 | 1554.8822029 |
| 616.5228113440197 | 520.246130971 | 616.5228113440197 | 6133.38249318 | 616.5228113440197 | 1452.07976142 |
| 614.3764079459348 | 560.978139166 | 614.3764079459348 | 5991.76600455 | 614.3764079459348 | 1353.40969959 |
| 612.2448979591836 | 605.233425612 | 612.2448979591836 | 5844.89735357 | 612.2448979591836 | 1258.96412798 |
| 610.1281269066504 | 653.112389046 | 610.1281269066504 | 5693.73584682 | 610.1281269066504 | 1168.80524187 |
| 608.0259424402108 | 704.710513777 | 608.0259424402108 | 5539.25189125 | 608.0259424402108 | 1082.96686718 |
| 605.9381943041809 | 760.116911141 | 605.9381943041809 | 5382.41909817 | 605.9381943041809 | 1001.45617165 |
| 603.864734299517 | 819.412760764 | 603.864734299517 | 5224.20648765 | 603.864734299517 | 924.255510281 |
| 601.8054162487462 | 882.66966356 | 601.8054162487462 | 5065.57086981 | 601.8054162487462 | 851.324373961 |
| 599.7600959616153 | 949.947920579 | 599.7600959616153 | 4907.44947462 | 599.7600959616153 | 782.60141127 |
| 597.7286312014345 | 1021.29475388 | 597.7286312014345 | 4750.75289644 | 597.7286312014345 | 718.006494707 |
| 595.7108816521048 | 1096.74248771 | 595.7108816521048 | 4596.35841378 | 595.7108816521048 | 657.442804182 |
| 593.7067088858104 | 1176.30671035 | 593.7067088858104 | 4445.1037382 | 593.7067088858104 | 600.798902519 |
| 591.7159763313609 | 1259.98443861 | 591.7159763313609 | 4297.78123925 | 591.7159763313609 | 547.950779864 |
| 589.7385492431688 | 1347.75230914 | 589.7385492431688 | 4155.13268592 | 589.7385492431688 | 498.763846221 |
| 587.7742946708463 | 1439.56482161 | 587.7742946708463 | 4017.84453715 | 587.7742946708463 | 453.094853765 |
| 585.8230814294083 | 1535.35266072 | 585.8230814294083 | 3886.54380773 | 585.8230814294083 | 410.793733109 |
| 583.8847800700661 | 1635.02112442 | 583.8847800700661 | 3761.79452839 | 583.8847800700661 | 371.705330235 |
| 581.9592628516003 | 1738.44868669 | 581.9592628516003 | 3644.09481253 | 581.9592628516003 | 335.671033323 |
| 580.046403712297 | 1845.48572321 | 580.046403712297 | 3533.87453554 | 580.046403712297 | 302.530281138 |
| 578.1460782424359 | 1955.95342833 | 578.1460782424359 | 3431.49362694 | 578.1460782424359 | 272.121947016 |
| 576.2581636573184 | 2069.64295082 | 576.2581636573184 | 3337.24096974 | 576.2581636573184 | 244.285594686 |
| 574.3825387708214 | 2186.314775 | 574.3825387708214 | 3251.33389661 | 574.3825387708214 | 218.862604217 |
| 572.5190839694656 | 2305.69837207 | 572.5190839694656 | 3173.91826804 | 572.5190839694656 | 195.697168308 |
| 570.6676811869887 | 2427.4921445 | 570.6676811869887 | 3105.06911325 | 570.6676811869887 | 174.637160777 |
| 568.8282138794084 | 2551.3636836 | 568.8282138794084 | 3044.79181154 | 568.8282138794084 | 155.534880632 |
| 567.000567000567 | 2676.9503573 | 567.000567000567 | 2993.02378837 | 567.000567000567 | 138.247676389 |
| 565.1846269781461 | 2803.86024164 | 565.1846269781461 | 2949.63669803 | 565.1846269781461 | 122.638456381 |
| 563.3802816901408 | 2931.67340548 | 563.3802816901408 | 2914.43906259 | 563.3802816901408 | 108.576091681 |
| 561.5874204417821 | 3059.94355338 | 561.5874204417821 | 2887.17933481 | 561.5874204417821 | 95.9357189382 |
| 559.8059339428997 | 3188.20002703 | 559.8059339428997 | 2867.54935136 | 559.8059339428997 | 84.5989509506 |
| 558.0357142857143 | 3315.95016041 | 558.0357142857143 | 2855.18814146 | 558.0357142857143 | 74.4540030967 |
| 556.2766549230483 | 3442.68197888 | 556.2766549230483 | 2849.6860549 | 556.2766549230483 | 65.3957439262 |
| 554.52865064695 | 3567.86722677 | 554.52865064695 | 2850.58917283 | 554.52865064695 | 57.3256782365 |
| 552.791597567717 | 3690.9647029 | 552.791597567717 | 2857.40396387 | 552.791597567717 | 50.1518708534 |
| 551.0653930933137 | 3811.42387807 | 551.0653930933137 | 2869.60214782 | 551.0653930933137 | 43.7888191207 |
| 549.3499359091741 | 3928.6887635 | 549.3499359091741 | 2886.62572851 | 549.3499359091741 | 38.1572817981 |
| 547.645125958379 | 4042.20199422 | 547.645125958379 | 2907.8921575 | 547.645125958379 | 33.1840716818 |
| 545.950864422202 | 4151.40908714 | 545.950864422202 | 2932.79958966 | 545.950864422202 | 28.8018188153 |
| 544.2670537010159 | 4255.76282927 | 544.2670537010159 | 2960.73219196 | 544.2670537010159 | 24.9487106699 |
| 542.5935973955508 | 4354.72774832 | 542.5935973955508 | 2991.06546656 | 542.5935973955508 | 21.5682151496 |
| 540.9304002884962 | 4447.78461491 | 540.9304002884962 | 3023.17154938 | 540.9304002884962 | 18.6087917341 |
| 539.2773683264425 | 4534.43492374 | 539.2773683264425 | 3056.42444594 | 539.2773683264425 | 16.023595525 |
| 537.6344086021505 | 4614.20529941 | 537.6344086021505 | 3090.2051661 | 537.6344086021505 | 13.7701784124 |
| 536.0014293371448 | 4686.65177248 | 536.0014293371448 | 3123.90672068 | 536.0014293371448 | 11.8101910469 |
| 534.3783398646241 | 4751.36387143 | 534.3783398646241 | 3156.93894312 | 534.3783398646241 | 10.1090887824 |
| 532.7650506126798 | 4807.96847746 | 532.7650506126798 | 3188.73310091 | 532.7650506126798 | 8.63584426656 |
| 531.1614730878186 | 4856.13339136 | 531.1614730878186 | 3218.74626282 | 531.1614730878186 | 7.36266889198 |
| 529.5675198587819 | 4895.57056439 | 529.5675198587819 | 3246.46538941 | 529.5675198587819 | 6.26474489286 |
| 527.9831045406547 | 4926.03894907 | 527.9831045406547 | 3271.41111677 | 527.9831045406547 | 5.3199694782 |
| 526.4081417792595 | 4947.34693028 | 526.4081417792595 | 3293.1412053 | 526.4081417792595 | 4.5087120353 |
| 524.8425472358292 | 4959.35430241 | 524.8425472358292 | 3311.25362849 | 524.8425472358292 | 3.81358511784 |
| 523.2862375719518 | 4961.97376399 | 523.2862375719518 | 3325.38927917 | 523.2862375719518 | 3.21922965014 |
| 521.7391304347826 | 4955.17190775 | 521.7391304347826 | 3335.23427452 | 521.7391304347826 | 2.71211453259 |
| 520.2011444425177 | 4938.969691 | 520.2011444425177 | 3340.52184426 | 520.2011444425177 | 2.28035062167 |
| 518.6721991701245 | 4913.4423779 | 518.6721991701245 | 3341.03379058 | 518.6721991701245 | 1.91351887873 |
| 517.1522151353215 | 4878.71895298 | 517.1522151353215 | 3336.60151225 | 517.1522151353215 | 1.60251233405 |
| 515.6411137848057 | 4834.98101207 | 515.6411137848057 | 3327.10658962 | 515.6411137848057 | 1.33939139273 |
| 514.1388174807198 | 4782.46114435 | 514.1388174807198 | 3312.4809314 | 514.1388174807198 | 1.11725191504 |
| 512.6452494873547 | 4721.44082608 | 512.6452494873547 | 3292.70648848 | 512.6452494873547 | 0.930105433598 |
| 511.1603339580848 | 4652.24785315 | 511.1603339580848 | 3267.81454439 | 511.1603339580848 | 0.772770819448 |
| 509.683995922528 | 4575.25334588 | 509.683995922528 | 3237.884596 | 509.683995922528 | 0.640776677724 |
| 508.2161612739285 | 4490.86836505 | 508.2161612739285 | 3203.04284212 | 508.2161612739285 | 0.530273737196 |
| 506.7567567567567 | 4399.54018307 | 506.7567567567567 | 3163.46030159 | 506.7567567567567 | 0.437956495332 |
| 505.3057099545225 | 4301.74825842 | 505.3057099545225 | 3119.35058558 | 505.3057099545225 | 0.360993388851 |
| 503.8629492777964 | 4197.99996483 | 503.8629492777964 | 3070.96735216 | 503.8629492777964 | 0.296964777147 |
| 502.4284039524367 | 4088.82612937 | 502.4284039524367 | 3018.60147383 | 502.4284039524367 | 0.243808050732 |
| 501.00200400801606 | 3974.77643487 | 501.00200400801606 | 2962.57795089 | 501.00200400801606 | 0.199769207149 |
| 499.5836802664446 | 3856.41474342 | 499.5836802664446 | 2903.25260523 | 499.5836802664446 | 0.163360271293 |
| 498.1733643307871 | 3734.31439684 | 498.1733643307871 | 2841.0085906 | 498.1733643307871 | 0.133321974402 |
| 496.7709885742673 | 3609.05354963 | 496.7709885742673 | 2776.25275576 | 496.7709885742673 | 0.108591145059 |
| 495.3764861294584 | 3481.21058755 | 495.3764861294584 | 2709.41189753 | 495.3764861294584 | 0.0882723053943 |
| 493.98979087765514 | 3351.35968258 | 493.98979087765514 | 2640.92894015 | 493.98979087765514 | 0.071613005609 |
| 492.61083743842363 | 3220.0665316 | 492.61083743842363 | 2571.25907668 | 492.61083743842363 | 0.0579824691398 |
| 491.2395611593253 | 3087.88432205 | 491.2395611593253 | 2500.86590709 | 491.2395611593253 | 0.0468531589279 |
| 489.8758981058131 | 2955.34996339 | 489.8758981058131 | 2430.21760571 | 489.8758981058131 | 0.0377849117952 |
| 488.5197850512946 | 2822.98061812 | 488.5197850512946 | 2359.78314911 | 488.5197850512946 | 0.0304113226431 |
| 487.17115946735953 | 2691.27056088 | 487.17115946735953 | 2290.02863287 | 487.17115946735953 | 0.0244280928535 |
| 485.82995951416996 | 2560.68838843 | 485.82995951416996 | 2221.41370336 | 485.82995951416996 | 0.0195830877385 |
| 484.49612403100775 | 2431.67459783 | 484.49612403100775 | 2154.38812776 | 484.49612403100775 | 0.015667876107 |
| 483.16959252697694 | 2304.63954428 | 483.16959252697694 | 2089.38852298 | 483.16959252697694 | 0.0125105509692 |
| 481.8503051718599 | 2179.96178441 | 481.8503051718599 | 2026.83526107 | 481.8503051718599 | 0.00996965410662 |
| 480.5382027871216 | 2057.98680554 | 480.5382027871216 | 1967.12956589 | 480.5382027871216 | 0.00792904877373 |
| 479.23322683706067 | 1939.02613594 | 479.23322683706067 | 1910.65081329 | 479.23322683706067 | 0.00629360424059 |
| 477.9353194201051 | 1823.35682656 | 477.9353194201051 | 1857.75404407 | 477.9353194201051 | 0.00498557335155 |
| 476.64442326024783 | 1711.22128987 | 476.64442326024783 | 1808.76769694 | 476.64442326024783 | 0.00394155988295 |
| 475.3604816986214 | 1602.82747791 | 475.3604816986214 | 1763.99156617 | 475.3604816986214 | 0.0031099863603 |
| 474.08343868520853 | 1498.34937751 | 474.08343868520853 | 1723.69498703 | 474.08343868520853 | 0.00244898527856 |
| 472.8132387706856 | 1397.92779835 | 472.8132387706856 | 1688.11524995 | 472.8132387706856 | 0.0019246474919 |
| 471.5498270983967 | 1301.67142661 | 471.5498270983967 | 1657.45624362 | 471.5498270983967 | 0.00150957103339 |
| 470.29314939645707 | 1209.65811531 | 470.29314939645707 | 1631.88732545 | 470.29314939645707 | 0.00118166191955 |
| 469.04315196998124 | 1121.93638112 | 469.04315196998124 | 1611.5424175 | 469.04315196998124 | 0.000923145710479 |
| 467.7997816934352 | 1038.52707672 | 467.7997816934352 | 1596.5193253 | 467.7997816934352 | 0.000719754850581 |
| 466.5629860031104 | 959.425207455 | 466.5629860031104 | 1586.87927644 | 466.5629860031104 | 0.000560062212943 |
| 465.33271288971605 | 884.601861323 | 465.33271288971605 | 1582.64667603 | 465.33271288971605 | 0.000434935913994 |
| 464.10891089108907 | 814.006222216 | 464.10891089108907 | 1583.80907584 | 464.10891089108907 | 0.000337094443405 |
| 462.8915290850177 | 747.567637186 | 462.8915290850177 | 1590.31735438 | 462.8915290850177 | 0.000260744551364 |
| 461.68051708217905 | 685.197710215 | 461.68051708217905 | 1602.08610499 | 461.68051708217905 | 0.000201287225668 |
| 460.47582501918646 | 626.792396644 | 460.47582501918646 | 1618.99422966 | 460.47582501918646 | 0.000155079541817 |
| 459.2774035517452 | 572.234074502 | 459.2774035517452 | 1640.88573607 | 459.2774035517452 | 0.00011924224028 |
| 458.0852038479157 | 521.393571182 | 458.0852038479157 | 1667.57073577 | 458.0852038479157 | 9.15046293436e-05 |
| 456.89917758148033 | 474.132126317 | 456.89917758148033 | 1698.82664113 | 456.89917758148033 | 7.00798763233e-05 |
| 455.7192769254139 | 430.30327416 | 455.7192769254139 | 1734.39955859 | 455.7192769254139 | 5.35649751653e-05 |
| 454.54545454545456 | 389.754631294 | 454.54545454545456 | 1774.00587539 | 454.54545454545456 | 4.08607006351e-05 |
| 453.3776635937736 | 352.329577983 | 453.3776635937736 | 1817.33403615 | 453.3776635937736 | 3.11077091734e-05 |
| 452.2158577027434 | 317.868823955 | 452.2158577027434 | 1864.04650505 | 452.2158577027434 | 2.36356510822e-05 |
| 451.05999097880016 | 286.211851757 | 451.05999097880016 | 1913.78190794 | 451.05999097880016 | 1.79227410035e-05 |
| 449.9100179964007 | 257.198233089 | 449.9100179964007 | 1966.15734768 | 449.9100179964007 | 1.35637134484e-05 |
| 448.7658937920718 | 230.66881562 | 448.7658937920718 | 2020.77088412 | 448.7658937920718 | 1.02444843026e-05 |
| 447.6275738585497 | 206.466779748 | 447.6275738585497 | 2077.20416864 | 447.6275738585497 | 7.72216210471e-06 |
| 446.49501413900873 | 184.438566506 | 446.49501413900873 | 2135.02522079 | 446.49501413900873 | 5.80931658298e-06 |
| 445.36817102137763 | 164.434679383 | 445.36817102137763 | 2193.791333 | 445.36817102137763 | 4.36162668791e-06 |
| 444.247001332741 | 146.310364216 | 444.247001332741 | 2253.05208663 | 444.247001332741 | 3.26820473969e-06 |
| 443.13146233382565 | 129.926172432 | 443.13146233382565 | 2312.3524608 | 443.13146233382565 | 2.44403452166e-06 |
| 442.02151171357 | 115.1484139 | 442.02151171357 | 2371.23601304 | 442.02151171357 | 1.82407517463e-06 |
| 440.9171075837742 | 101.849506416 | 440.9171075837742 | 2429.24810867 | 440.9171075837742 | 1.35867456563e-06 |
| 439.8182084738308 | 89.908229394 | 439.8182084738308 | 2485.93917414 | 439.8182084738308 | 1.01000953582e-06 |
| 438.72477332553376 | 79.2098897705 | 438.72477332553376 | 2540.8679475 | 438.72477332553376 | 7.49329479133e-07 |
| 437.636761487965 | 69.6464083272 | 437.636761487965 | 2593.60469785 | 437.636761487965 | 5.54826860436e-07 |
| 436.5541327124563 | 61.1163347407 | 436.5541327124563 | 2643.73438462 | 436.5541327124563 | 4.09995842752e-07 |
| 435.4768471476266 | 53.5247996162 | 435.4768471476266 | 2690.85972646 | 435.4768471476266 | 3.02370030175e-07 |
| 434.4048653344918 | 46.7834115931 | 434.4048653344918 | 2734.60414954 | 434.4048653344918 | 2.22553971335e-07 |
| 433.3381482016467 | 40.8101073481 | 433.3381482016467 | 2774.6145849 | 433.3381482016467 | 1.63481746383e-07 |
| 432.2766570605187 | 35.5289619693 | 432.2766570605187 | 2810.56408529 | 432.2766570605187 | 1.1985068018e-07 |
| 431.22035360069 | 30.8699667621 | 431.22035360069 | 2842.15423295 | 431.22035360069 | 8.76897945205e-08 |
| 430.1691998852882 | 26.7687810742 | 430.1691998852882 | 2869.1173113 | 430.1691998852882 | 6.40316828992e-08 |
| 429.1231583464454 | 23.1664642237 | 429.1231583464454 | 2891.21821583 | 429.1231583464454 | 4.66635842871e-08 |
| 428.0821917808219 | 20.0091930821 | 428.0821917808219 | 2908.25608166 | 428.0821917808219 | 3.39389644494e-08 |
| 427.0462633451957 | 17.2479703193 | 427.0462633451957 | 2920.06560861 | 427.0462633451957 | 2.46352170855e-08 |
| 426.01533655211585 | 14.8383277727 | 426.01533655211585 | 2926.51806763 | 426.01533655211585 | 1.78464366403e-08 |
| 424.9893752656184 | 12.7400288655 | 424.9893752656184 | 2927.52197639 | 424.9893752656184 | 1.29027995183e-08 |
| 423.96834369700395 | 10.9167734729 | 423.96834369700395 | 2923.02343567 | 423.96834369700395 | 9.31008611704e-09 |
| 422.9522064006767 | 9.33590813669 | 422.9522064006767 | 2913.00612244 | 422.9522064006767 | 6.70441302152e-09 |
| 421.9409282700422 | 7.96814405487 | 421.9409282700422 | 2897.4909397 | 421.9409282700422 | 4.81842537749e-09 |
| 420.93447453346425 | 6.78728482654 | 420.93447453346425 | 2876.53532781 | 420.93447453346425 | 3.45610420056e-09 |
| 419.9328107502799 | 5.76996552804 | 419.9328107502799 | 2850.23224603 | 419.9328107502799 | 2.47403495831e-09 |
| 418.93590280687056 | 4.89540431999 | 418.93590280687056 | 2818.70883746 | 418.93590280687056 | 1.76751109946e-09 |
| 417.94371691278906 | 4.14516745049 | 417.94371691278906 | 2782.12479468 | 417.94371691278906 | 1.26024731583e-09 |
| 416.9562195969423 | 3.50294821987 | 416.9562195969423 | 2740.67044685 | 416.9562195969423 | 8.96781676801e-10 |
| 415.97337770382694 | 2.95436020998 | 415.97337770382694 | 2694.56459312 | 415.97337770382694 | 6.36876148633e-10 |
| 414.99515838981876 | 2.48674485332 | 414.99515838981876 | 2644.05210951 | 414.99515838981876 | 4.51398962593e-10 |
| 414.0215291195142 | 2.08899322342 | 414.0215291195142 | 2589.40135993 | 414.0215291195142 | 3.19303325838e-10 |
| 413.0524576621231 | 1.7513817656 | 413.0524576621231 | 2530.9014434 | 413.0524576621231 | 2.25415430144e-10 |
| 412.08791208791206 | 1.46542155454 | 412.08791208791206 | 2468.85931177 | 412.08791208791206 | 1.58818523219e-10 |
| 411.1278607646978 | 1.22372055879 | 411.1278607646978 | 2403.59679299 | 411.1278607646978 | 1.11675004618e-10 |
| 410.17227235438884 | 1.01985831127 | 410.17227235438884 | 2335.44755573 | 410.17227235438884 | 7.83696880657e-11 |
| 409.22111580957574 | 0.848272324644 | 409.22111580957574 | 2264.754051 | 409.22111580957574 | 5.4888020753e-11 |
| 408.2743603701687 | 0.704155550459 | 408.2743603701687 | 2191.86446605 | 408.2743603701687 | 3.83658068637e-11 |
| 407.33197556008145 | 0.583364156981 | 407.33197556008145 | 2117.12972452 | 407.33197556008145 | 2.67638393979e-11 |
| 406.39393118396094 | 0.48233489152 | 406.39393118396094 | 2040.90056544 | 406.39393118396094 | 1.86333013337e-11 |
| 405.46019732396263 | 0.398011295915 | 405.46019732396263 | 1963.52473168 | 405.46019732396263 | 1.29469810629e-11 |
| 404.53074433656957 | 0.327778056783 | 404.53074433656957 | 1885.34429567 | 404.53074433656957 | 8.97810192405e-12 |
| 403.6055428494551 | 0.269402793168 | 403.6055428494551 | 1806.693148 | 403.6055428494551 | 6.2135223279e-12 |
| 402.68456375838923 | 0.220984611714 | 402.68456375838923 | 1727.89467081 | 402.68456375838923 | 4.29169162189e-12 |
| 401.76777822418643 | 0.180908791769 | 401.76777822418643 | 1649.25961513 | 401.76777822418643 | 2.95839708941e-12 |
| 400.85515766969536 | 0.147806998614 | 400.85515766969536 | 1571.08419745 | 400.85515766969536 | 2.03526862591e-12 |
| 399.9466737768297 | 0.120522461026 | 399.9466737768297 | 1493.64842751 | 399.9466737768297 | 1.39741153046e-12 |
| 399.0422984836393 | 0.0980795887323 | 399.0422984836393 | 1417.21467542 | 399.0422984836393 | 9.57556084202e-13 |
| 398.14200398142003 | 0.0796575450002 | 398.14200398142003 | 1342.02648304 | 398.14200398142003 | 6.54849395355e-13 |
| 397.24576271186436 | 0.0645673290108 | 397.24576271186436 | 1268.30762068 | 397.24576271186436 | 4.46946926096e-13 |
| 396.3535473642489 | 0.0522319611722 | 396.3535473642489 | 1196.26138715 | 396.3535473642489 | 3.04444261023e-13 |
| 395.46533087266016 | 0.0421694016963 | 395.46533087266016 | 1126.07014778 | 395.46533087266016 | 2.06965016202e-13 |
| 394.5810864132579 | 0.0339778682385 | 394.5810864132579 | 1057.89510262 | 394.5810864132579 | 1.404182011e-13 |
| 393.7007874015748 | 0.027323251933 | 393.7007874015748 | 991.876273683 | 393.7007874015748 | 9.5079563931e-14 |
| 392.82440748985204 | 0.0219283625849 | 392.82440748985204 | 928.132698668 | 392.82440748985204 | 6.42522396523e-14 |
| 391.9519205644107 | 0.0175637629958 | 391.9519205644107 | 866.762815865 | 391.9519205644107 | 4.3333789858e-14 |
| 391.08330074305826 | 0.0140399793637 | 391.08330074305826 | 807.845023833 | 391.08330074305826 | 2.91677136953e-14 |
| 390.2185223725286 | 0.0112008994223 | 390.2185223725286 | 751.438397935 | 390.2185223725286 | 1.95936531972e-14 |
| 389.3575600259571 | 0.00891819250679 | 389.3575600259571 | 697.583544957 | 389.3575600259571 | 1.31360792057e-14 |
| 388.5003885003885 | 0.00708660613479 | 388.5003885003885 | 646.303576491 | 388.5003885003885 | 8.78928228686e-15 |
| 387.6469828143171 | 0.00562001206783 | 387.6469828143171 | 597.605181479 | 387.6469828143171 | 5.86919286119e-15 |
| 386.7973182052604 | 0.00444809128275 | 386.7973182052604 | 551.479778422 | 386.7973182052604 | 3.91147592287e-15 |
| 385.95137012736393 | 0.00351356196347 | 385.95137012736393 | 507.904728121 | 385.95137012736393 | 2.60159838893e-15 |
| 385.1091142490372 | 0.0027698676485 | 385.1091142490372 | 466.844588418 | 385.1091142490372 | 1.72693965317e-15 |
| 384.2705264506212 | 0.00217925417426 | 384.2705264506212 | 428.252393313 | 384.2705264506212 | 1.14406679617e-15 |
| 383.4355828220859 | 0.00171117417027 | 383.4355828220859 | 392.070939852 | 383.4355828220859 | 7.564198449e-16 |
| 382.6042596607575 | 0.00134096672011 | 382.6042596607575 | 358.234067432 | 382.6042596607575 | 4.9912780049e-16 |
| 381.77653346907607 | 0.00104876752557 | 381.77653346907607 | 326.667915539 | 381.77653346907607 | 3.2869864873e-16 |
| 380.95238095238096 | 0.00081861161887 | 380.95238095238096 | 297.292147369 | 380.95238095238096 | 2.16033643619e-16 |
| 380.1317790167258 | 0.00063769647101 | 380.1317790167258 | 270.021128345 | 380.1317790167258 | 1.4170402143e-16 |
| 379.31470476672143 | 0.000495778344764 | 379.31470476672143 | 244.765050075 | 379.31470476672143 | 9.27641721332e-17 |
| 378.5011355034065 | 0.000384679034938 | 378.5011355034065 | 221.430991872 | 378.5011355034065 | 6.060600871e-17 |
| 377.69104872214524 | 0.0002978838118 | 377.69104872214524 | 199.92391351 | 377.69104872214524 | 3.95174040711e-17 |
| 376.88442211055275 | 0.000230214515064 | 376.88442211055275 | 180.147574382 | 376.88442211055275 | 2.57157055593e-17 |
| 376.081233546446 | 0.00017756440608 | 376.081233546446 | 162.005375645 | 376.081233546446 | 1.6701127607e-17 |
| 375.28146109582184 | 0.000136683638095 | 375.28146109582184 | 145.401123304 | 375.28146109582184 | 1.08250636123e-17 |
| 374.48508301086 | 0.000105006104808 | 374.48508301086 | 130.239711414 | 374.48508301086 | 7.0024889618e-18 |
| 373.69207772795215 | 8.05100257392e-05 | 373.69207772795215 | 116.427725727 | 373.69207772795215 | 4.52076281109e-18 |
| 372.9024238657551 | 6.16059666609e-05 | 372.9024238657551 | 103.873969114 | 372.9024238657551 | 2.91278428923e-18 |
| 372.11610022326965 | 4.70471129445e-05 | 372.11610022326965 | 92.4899109899 | 372.11610022326965 | 1.87301923862e-18 |
| 371.33308577794276 | 3.58575461932e-05 | 371.33308577794276 | 82.1900637354 | 371.33308577794276 | 1.20202490497e-18 |
| 370.55335968379444 | 2.72750489579e-05 | 370.55335968379444 | 72.8922897465 | 370.55335968379444 | 7.69878172487e-19 |
| 369.7769012695673 | 2.0705603418e-05 | 369.7769012695673 | 64.5180432526 | 369.7769012695673 | 4.92116425068e-19 |
| 369.0036900369003 | 1.56872790422e-05 | 369.0036900369003 | 56.9925514495 | 369.0036900369003 | 3.13943161312e-19 |
| 368.23370565852457 | 1.1861639657e-05 | 368.23370565852457 | 50.2449397727 | 368.23370565852457 | 1.9988099693e-19 |
| 367.4669279764821 | 8.95115758781e-06 | 367.4669279764821 | 44.2083063195 | 367.4669279764821 | 1.27007480127e-19 |
| 366.7033370003667 | 6.74141477849e-06 | 366.7033370003667 | 38.8197505111 | 366.7033370003667 | 8.05423700795e-20 |
| 365.9429129055867 | 5.06710916832e-06 | 365.9429129055867 | 34.0203610875 | 365.9429129055867 | 5.09749518089e-20 |
| 365.1856360316494 | 3.80107848915e-06 | 365.1856360316494 | 29.7551684481 | 365.1856360316494 | 3.2197826476e-20 |
| 364.4314868804664 | 2.84571089019e-06 | 364.4314868804664 | 25.9730662134 | 364.4314868804664 | 2.02970817126e-20 |
| 363.68044611468054 | 2.12623897099e-06 | 363.68044611468054 | 22.6267066805 | 363.68044611468054 | 1.2769619531e-20 |
| 362.93249455601256 | 1.58551636687e-06 | 362.93249455601256 | 19.6723746075 | 362.93249455601256 | 8.0178814071e-21 |
| 362.1876131836291 | 1.17995852047e-06 | 362.1876131836291 | 17.0698434814 | 362.1876131836291 | 5.02433527017e-21 |
| 361.4457831325301 | 8.76395432444e-07 | 361.4457831325301 | 14.782218121 | 361.4457831325301 | 3.14220784363e-21 |
| 360.7069856919562 | 6.49637104625e-07 | 360.7069856919562 | 12.7757671445 | 360.7069856919562 | 1.96122995517e-21 |
| 359.97120230381563 | 4.80594602237e-07 | 359.97120230381563 | 11.0197484976 | 359.97120230381563 | 1.22168555922e-21 |
| 359.2384145611304 | 3.54833245714e-07 | 359.2384145611304 | 9.48623090613 | 359.2384145611304 | 7.59499821021e-22 |
| 358.50860420650093 | 2.61461088979e-07 | 358.50860420650093 | 8.14991377951 | 358.50860420650093 | 4.71230318003e-22 |
| 357.7817531305903 | 1.92276927112e-07 | 357.7817531305903 | 6.98794777046 | 357.7817531305903 | 2.9179381741e-22 |
| 357.057843370626 | 1.41118718426e-07 | 357.057843370626 | 5.97975788079 | 357.057843370626 | 1.80325132566e-22 |
| 356.33685710892024 | 1.03366406188e-07 | 356.33685710892024 | 5.10687070727 | 356.33685710892024 | 1.11217658503e-22 |
| 355.6187766714082 | 7.55634105569e-08 | 355.6187766714082 | 4.35274714225 | 355.6187766714082 | 6.84586842443e-23 |
| 354.9035845262037 | 5.51291156573e-08 | 354.9035845262037 | 3.70262158525 | 354.9035845262037 | 4.20552928202e-23 |
| 354.1912632821723 | 4.01409677701e-08 | 354.1912632821723 | 3.14334848505 | 354.1912632821723 | 2.57839878297e-23 |
| 353.48179568752204 | 2.91697017876e-08 | 353.48179568752204 | 2.66325681767 | 353.48179568752204 | 1.57767239565e-23 |
| 352.77516462841015 | 2.11550217387e-08 | 352.77516462841015 | 2.25201291411 | 352.77516462841015 | 9.63431582784e-24 |
| 352.07135312756714 | 1.5312013312e-08 | 352.07135312756714 | 1.90049188259 | 352.07135312756714 | 5.87167817896e-24 |
| 351.3703443429374 | 1.10608487676e-08 | 351.3703443429374 | 1.60065772319 | 351.3703443429374 | 3.57141996842e-24 |
| 350.6721215663355 | 7.97410489109e-09 | 350.6721215663355 | 1.3454521066 | 350.6721215663355 | 2.16798819809e-24 |
| 349.9766682221185 | 5.73736878421e-09 | 349.9766682221185 | 1.12869168247 | 349.9766682221185 | 1.31343993812e-24 |
| 349.2839678658749 | 4.1198454067e-09 | 349.2839678658749 | 0.944973695122 | 349.2839678658749 | 7.94146881812e-25 |
| 348.59400418312805 | 2.95247619177e-09 | 348.59400418312805 | 0.789589614059 | 348.59400418312805 | 4.79213192118e-25 |
| 347.90676098805517 | 2.11168540611e-09 | 347.90676098805517 | 0.658446431006 | 347.90676098805517 | 2.88598459261e-25 |
| 347.2222222222222 | 1.50733353694e-09 | 347.2222222222222 | 0.547995234606 | 347.2222222222222 | 1.73458890535e-25 |
| 346.54037195333257 | 1.0738084911e-09 | 346.54037195333257 | 0.455166644644 | 346.54037195333257 | 1.0404864606e-25 |
| 345.8611943739912 | 7.63451817598e-10 | 345.8611943739912 | 0.377312669809 | 345.8611943739912 | 6.22893236593e-26 |
| 345.1846738004832 | 5.41718615724e-10 | 345.1846738004832 | 0.312154544203 | 345.1846738004832 | 3.72158643692e-26 |
| 344.5107946715664 | 3.83621741981e-10 | 344.5107946715664 | 0.25773609698 | 344.5107946715664 | 2.21911555813e-26 |
| 343.8395415472779 | 2.71125266533e-10 | 343.8395415472779 | 0.212382215272 | 343.8395415472779 | 1.32059314683e-26 |
| 343.17089910775564 | 1.91237957267e-10 | 343.17089910775564 | 0.174661971666 | 343.17089910775564 | 7.84323943373e-27 |
| 342.50485215207215 | 1.34621840842e-10 | 342.50485215207215 | 0.143356002885 | 342.50485215207215 | 4.64899650806e-27 |
| 341.84138559708293 | 9.45789041797e-11 | 341.84138559708293 | 0.117427745038 | 341.84138559708293 | 2.75017468312e-27 |
| 341.1804844762879 | 6.63147832472e-11 | 341.1804844762879 | 0.0959981518533 | 341.1804844762879 | 1.62367331326e-27 |
| 340.522133938706 | 4.6404890716e-11 | 340.522133938706 | 0.0783235450347 | 340.522133938706 | 9.56696837775e-28 |
| 339.86631924776253 | 3.24081676441e-11 | 339.86631924776253 | 0.0637762695998 | 339.86631924776253 | 5.62583949039e-28 |
| 339.2130257801899 | 2.25882466601e-11 | 339.2130257801899 | 0.0518278511468 | 339.2130257801899 | 3.3017002985e-28 |
| 338.56223902494077 | 1.57125939745e-11 | 338.56223902494077 | 0.042034376089 | 338.56223902494077 | 1.93386108286e-28 |
| 337.91394458211306 | 1.09081368101e-11 | 337.91394458211306 | 0.0340238395337 | 337.91394458211306 | 1.13044701502e-28 |
| 337.2681281618887 | 7.55771629936e-12 | 337.2681281618887 | 0.0274852283996 | 337.2681281618887 | 6.59496449946e-29 |
| 336.6247755834829 | 5.22598206882e-12 | 336.6247755834829 | 0.0221591293214 | 336.6247755834829 | 3.83983025087e-29 |
| 335.9838727741068 | 3.60647200608e-12 | 335.9838727741068 | 0.0178296717119 | 335.9838727741068 | 2.23125343657e-29 |
| 335.3454057679409 | 2.4839024494e-12 | 335.3454057679409 | 0.0143176359174 | 335.3454057679409 | 1.29396667606e-29 |
| 334.709360705121 | 1.70735498024e-12 | 334.709360705121 | 0.0114745746395 | 334.709360705121 | 7.48918561615e-30 |
| 334.07572383073494 | 1.1712522416e-12 | 334.07572383073494 | 0.00917781266995 | 334.07572383073494 | 4.32596907079e-30 |
| 333.44448149383123 | 8.01889198522e-13 | 333.44448149383123 | 0.00732620548654 | 333.44448149383123 | 2.49384546686e-30 |
| 332.81562014643885 | 5.47918054153e-13 | 332.81562014643885 | 0.00583655141056 | 332.81562014643885 | 1.43480532583e-30 |
| 332.1891263425977 | 3.73640699768e-13 | 332.1891263425977 | 0.00464056486917 | 332.1891263425977 | 8.23860601325e-31 |
| 331.5649867374005 | 2.54290456233e-13 | 331.5649867374005 | 0.00368232989297 | 331.5649867374005 | 4.72119351545e-31 |
| 330.9431880860452 | 1.72720252128e-13 | 330.9431880860452 | 0.00291616338094 | 330.9431880860452 | 2.70014559439e-31 |
| 330.323717242898 | 1.17082984275e-13 | 330.323717242898 | 0.00230482695113 | 330.323717242898 | 1.54120310656e-31 |
| 329.70656116056705 | 7.92102932672e-14 | 329.70656116056705 | 0.00181803444897 | 329.70656116056705 | 8.77950199974e-32 |
| 329.0917068889864 | 5.34818936944e-14 | 329.0917068889864 | 0.00143120948475 | 329.0917068889864 | 4.9913405434e-32 |
| 328.47914157451 | 3.60387109761e-14 | 328.47914157451 | 0.00112445379922 | 328.47914157451 | 2.8320559807e-32 |
| 327.86885245901635 | 2.423645172e-14 | 327.86885245901635 | 0.00088169289193 | 327.86885245901635 | 1.60370239944e-32 |
| 327.26082687902255 | 1.62669504804e-14 | 327.26082687902255 | 0.000689970266988 | 327.26082687902255 | 9.06323074915e-33 |
| 326.6550522648083 | 1.08963380289e-14 | 326.6550522648083 | 0.000538865929219 | 326.6550522648083 | 5.11186773668e-33 |
| 326.05151613955 | 7.28437519867e-15 | 326.05151613955 | 0.000420018469202 | 326.05151613955 | 2.87748781107e-33 |
| 325.4502061184639 | 4.86005700066e-15 | 325.4502061184639 | 0.000326733272885 | 325.4502061184639 | 1.6165334283e-33 |
| 324.8511099079588 | 3.23614322906e-15 | 324.8511099079588 | 0.000253662139649 | 324.8511099079588 | 9.063442836e-34 |
| 324.25421530479895 | 2.15055930279e-15 | 324.25421530479895 | 0.000196541946424 | 324.25421530479895 | 5.0715302829e-34 |
| 323.65951019527455 | 1.42630507315e-15 | 323.65951019527455 | 0.000151982004087 | 323.65951019527455 | 2.832188527e-34 |
| 323.0669825543829 | 9.44084237507e-16 | 323.0669825543829 | 0.000117291460526 | 323.0669825543829 | 1.57849281067e-34 |
| 322.4766204450177 | 6.23657834059e-16 | 322.4766204450177 | 9.03395523896e-05 | 322.4766204450177 | 8.78011835623e-35 |
| 321.88841201716735 | 4.1116802423e-16 | 321.88841201716735 | 6.94427302682e-05 | 321.88841201716735 | 4.87411133597e-35 |
| 321.3023455071222 | 2.70538824943e-16 | 321.3023455071222 | 5.32737114084e-05 | 321.3023455071222 | 2.70039834551e-35 |
| 320.71840923669015 | 1.77654896126e-16 | 320.71840923669015 | 4.07883777614e-05 | 320.71840923669015 | 1.49312970012e-35 |
| 320.1365916124213 | 1.16429244107e-16 | 320.1365916124213 | 3.11671596034e-05 | 320.1365916124213 | 8.23957000812e-36 |
| 319.5568811248402 | 7.61525218416e-17 | 319.5568811248402 | 2.37681472864e-05 | 319.5568811248402 | 4.53783682363e-36 |
| 318.97926634768737 | 4.97100061452e-17 | 318.97926634768737 | 1.80896743215e-05 | 318.97926634768737 | 2.49419563517e-36 |
| 318.40373593716834 | 3.23847581964e-17 | 318.40373593716834 | 1.37405298241e-05 | 318.40373593716834 | 1.3681996271e-36 |
| 317.8302786312109 | 2.10559487762e-17 | 317.8302786312109 | 1.0416301045e-05 | 317.8302786312109 | 7.4904124726e-37 |
| 317.2588832487309 | 1.36630068746e-17 | 317.2588832487309 | 7.88062895561e-06 | 317.2588832487309 | 4.092600117e-37 |
| 316.6895386889053 | 8.84820289972e-18 | 316.6895386889053 | 5.95039162827e-06 | 316.6895386889053 | 2.23167117562e-37 |
| 316.1222339304531 | 5.7187508196e-18 | 316.1222339304531 | 4.48401991642e-06 | 316.1222339304531 | 1.21450248801e-37 |
| 315.55695803092453 | 3.68879558025e-18 | 315.55695803092453 | 3.37230486292e-06 | 315.55695803092453 | 6.5963535761e-38 |
| 314.99370012599746 | 2.37468122459e-18 | 314.99370012599746 | 2.5311823743e-06 | 314.99370012599746 | 3.57558212279e-38 |
| 314.432449428781 | 1.52567968171e-18 | 314.432449428781 | 1.89608306575e-06 | 314.432449428781 | 1.93431366933e-38 |
| 313.8731952291274 | 9.78269967097e-19 | 313.8731952291274 | 1.41751803886e-06 | 313.8731952291274 | 1.04434587939e-38 |
| 313.31592689295036 | 6.26024591468e-19 | 313.31592689295036 | 1.05763823336e-06 | 313.31592689295036 | 5.62728779985e-39 |
| 312.76063386155124 | 3.99817114281e-19 | 312.76063386155124 | 7.87558825822e-07 | 312.76063386155124 | 3.02615528443e-39 |
| 312.2073056509522 | 2.54840636551e-19 | 312.2073056509522 | 5.85283359366e-07 | 312.2073056509522 | 1.62412931478e-39 |
| 311.65593185123623 | 1.62111302892e-19 | 311.65593185123623 | 4.34096878802e-07 | 311.65593185123623 | 8.69936020863e-40 |
| 311.1065021258944 | 1.02918921206e-19 | 311.1065021258944 | 3.21324960555e-07 | 311.1065021258944 | 4.65041097892e-40 |
| 310.5590062111801 | 6.52100402616e-20 | 310.5590062111801 | 2.3737751306e-07 | 310.5590062111801 | 2.48103373637e-40 |
| 310.01343391546965 | 4.12354772491e-20 | 310.01343391546965 | 1.75013681764e-07 | 310.01343391546965 | 1.32102585346e-40 |
| 309.4697751186301 | 2.60234514735e-20 | 309.4697751186301 | 1.28778018916e-07 | 309.4697751186301 | 7.01984103465e-41 |
| 308.9280197713932 | 1.6390646391e-20 | 308.9280197713932 | 9.45689988248e-08 | 308.9280197713932 | 3.7228929714e-41 |
| 308.3881578947368 | 1.03030208707e-20 | 308.3881578947368 | 6.93095622393e-08 | 308.3881578947368 | 1.97047594424e-41 |
| 307.8501795792714 | 6.46353927252e-21 | 307.8501795792714 | 5.0696132985e-08 | 307.8501795792714 | 1.04087610007e-41 |
| 307.31407498463426 | 4.04681655558e-21 | 307.31407498463426 | 3.70078473312e-08 | 307.31407498463426 | 5.48737005512e-42 |
| 306.77983433888943 | 2.52868049037e-21 | 306.77983433888943 | 2.69618772589e-08 | 306.77983433888943 | 2.88713289027e-42 |
| 306.2474479379338 | 1.57692744349e-21 | 306.2474479379338 | 1.96039571114e-08 | 306.2474479379338 | 1.51602587065e-42 |
| 305.7169061449098 | 9.81446834162e-22 | 305.7169061449098 | 1.42257336506e-08 | 305.7169061449098 | 7.94481454051e-43 |
| 305.1881993896236 | 6.09619933467e-22 | 305.1881993896236 | 1.03025069338e-08 | 305.1881993896236 | 4.15526023522e-43 |
| 304.6613181679699 | 3.77910408511e-22 | 304.6613181679699 | 7.44643616479e-09 | 304.6613181679699 | 2.16895232028e-43 |
| 304.1362530413625 | 2.33806112766e-22 | 304.1362530413625 | 5.37144754717e-09 | 304.1362530413625 | 1.12989762361e-43 |
| 303.61299463617036 | 1.44364424149e-22 | 303.61299463617036 | 3.8669763897e-09 | 303.61299463617036 | 5.87442682488e-44 |
| 303.09153364316023 | 8.89614427962e-23 | 303.09153364316023 | 2.77836317882e-09 | 303.09153364316023 | 3.04810002464e-44 |
| 302.571860816944 | 5.47117701473e-23 | 302.571860816944 | 1.99224993784e-09 | 302.571860816944 | 1.5784478704e-44 |
| 302.0539669754329 | 3.35812631564e-23 | 302.0539669754329 | 1.4257255816e-09 | 302.0539669754329 | 8.15771606934e-45 |
| 301.5378429992964 | 2.05707726035e-23 | 301.5378429992964 | 1.01827568921e-09 | 301.5378429992964 | 4.20769489005e-45 |
| 301.02347983142687 | 1.25759699828e-23 | 301.02347983142687 | 7.25825332131e-10 | 301.02347983142687 | 2.16599379697e-45 |
| 300.5108684764098 | 7.67307943479e-24 | 300.5108684764098 | 5.16340487708e-10 | 300.5108684764098 | 1.11277533162e-45 |

| **GON1-Al3** | | **GON1-Al4** | | **GON1-Al5** | |
| --- | --- | --- | --- | --- | --- |
| Wavelength (nm) | Abs | Wavelength (nm) | Abs | Wavelength (nm) | Abs |
| 2000.0 | 4.11729500628 | 2000.0 | 2.96521040313e-16 | 2000.0 | 1.56899444888e-05 |
| 1977.5873434410018 | 4.8036676086 | 1977.5873434410018 | 4.47473948562e-16 | 1977.5873434410018 | 2.05134780585e-05 |
| 1955.671447196871 | 5.59338302014 | 1955.671447196871 | 6.73933911914e-16 | 1955.671447196871 | 2.67674057681e-05 |
| 1934.2359767891683 | 6.50005384144 | 1934.2359767891683 | 1.01298771247e-15 | 1934.2359767891683 | 3.4859617348e-05 |
| 1913.265306122449 | 7.53876615789 | 1913.265306122449 | 1.51959675492e-15 | 1913.265306122449 | 4.53094270641e-05 |
| 1892.7444794952683 | 8.72618991996 | 1892.7444794952683 | 2.27504426205e-15 | 1892.7444794952683 | 5.87765949448e-05 |
| 1872.6591760299625 | 10.080690288 | 1872.6591760299625 | 3.39929344251e-15 | 1872.6591760299625 | 7.60975111918e-05 |
| 1852.9956763434218 | 11.62243869 | 1852.9956763434218 | 5.06902899566e-15 | 1852.9956763434218 | 9.83302057405e-05 |
| 1833.7408312958437 | 13.3735221467 | 1833.7408312958437 | 7.54393968394e-15 | 1833.7408312958437 | 0.000126810203277 |
| 1814.8820326678765 | 15.3580492241 | 1814.8820326678765 | 1.12049249596e-14 | 1814.8820326678765 | 0.000163219672933 |
| 1796.4071856287424 | 17.6022507702 | 1796.4071856287424 | 1.66095172609e-14 | 1796.4071856287424 | 0.000209672833687 |
| 1778.3046828689983 | 20.1345733948 | 1778.3046828689983 | 2.45721060611e-14 | 1778.3046828689983 | 0.000268821185373 |
| 1760.5633802816901 | 22.9857634572 | 1760.5633802816901 | 3.62798151071e-14 | 1760.5633802816901 | 0.000343982857181 |
| 1743.1725740848342 | 26.1889391404 | 1743.1725740848342 | 5.34595208567e-14 | 1743.1725740848342 | 0.000439301219047 |
| 1726.1219792865363 | 29.7796480187 | 1726.1219792865363 | 7.86180688672e-14 | 1726.1219792865363 | 0.000559938906958 |
| 1709.4017094017095 | 33.7959073733 | 1709.4017094017095 | 1.15387029918e-13 | 1709.4017094017095 | 0.000712314594207 |
| 1693.002257336343 | 38.2782243839 | 1693.002257336343 | 1.69016432455e-13 | 1693.002257336343 | 0.000904391223016 |
| 1676.9144773616547 | 43.2695932244 | 1676.9144773616547 | 2.47080333773e-13 | 1676.9144773616547 | 0.00114602602343 |
| 1661.1295681063123 | 48.8154660329 | 1661.1295681063123 | 3.60482960565e-13 | 1661.1295681063123 | 0.00144939452087 |
| 1645.6390565002741 | 54.9636947073 | 1645.6390565002741 | 5.2489038328e-13 | 1645.6390565002741 | 0.00182950290495 |
| 1630.4347826086955 | 61.7644405052 | 1630.4347826086955 | 7.62763325934e-13 | 1630.4347826086955 | 0.00230480563813 |
| 1615.5088852988692 | 69.2700485162 | 1615.5088852988692 | 1.10623731703e-12 | 1615.5088852988692 | 0.00289794806406 |
| 1600.8537886872998 | 77.534884214 | 1600.8537886872998 | 1.60119460582e-12 | 1600.8537886872998 | 0.00363665707638 |
| 1586.4621893178212 | 86.6151295046 | 1586.4621893178212 | 2.31300858742e-12 | 1586.4621893178212 | 0.00455480667441 |
| 1572.3270440251572 | 96.5685359655 | 1572.3270440251572 | 3.33463024557e-12 | 1572.3270440251572 | 0.0056936895124 |
| 1558.4415584415583 | 107.454133312 | 1558.4415584415583 | 4.79794690616e-12 | 1558.4415584415583 | 0.00710353039211 |
| 1544.799176107106 | 119.331891552 | 1544.799176107106 | 6.88970304397e-12 | 1544.799176107106 | 0.00884528310664 |
| 1531.3935681470139 | 132.262335781 | 1531.3935681470139 | 9.87376717873e-12 | 1531.3935681470139 | 0.0109927581661 |
| 1518.2186234817814 | 146.306113137 | 1518.2186234817814 | 1.41222070422e-11 | 1518.2186234817814 | 0.0136351357727 |
| 1505.2684395383842 | 161.523512064 | 1505.2684395383842 | 2.01585634133e-11 | 1505.2684395383842 | 0.0168799260101 |
| 1492.5373134328358 | 177.973934745 | 1492.5373134328358 | 2.87179805527e-11 | 1492.5373134328358 | 0.0208564466124 |
| 1480.0197335964478 | 195.715324322 | 1480.0197335964478 | 4.08305780139e-11 | 1480.0197335964478 | 0.0257198979141 |
| 1467.7103718199608 | 214.803549331 | 1467.7103718199608 | 5.79367956873e-11 | 1467.7103718199608 | 0.0316561246858 |
| 1455.604075691412 | 235.291748645 | 1455.604075691412 | 8.20466265158e-11 | 1455.604075691412 | 0.0388871655374 |
| 1443.6958614051973 | 257.229641089 | 1443.6958614051973 | 1.15958956647e-10 | 1443.6958614051973 | 0.0476777024305 |
| 1431.9809069212408 | 280.662804822 | 1431.9809069212408 | 1.63563043173e-10 | 1431.9809069212408 | 0.0583425355581 |
| 1420.4545454545455 | 305.631932451 | 1420.4545454545455 | 2.30251982613e-10 | 1420.4545454545455 | 0.0712552223865 |
| 1409.1122592766555 | 332.172068768 | 1409.1122592766555 | 3.23488530973e-10 | 1409.1122592766555 | 0.0868580339448 |
| 1397.9496738117427 | 360.311838839 | 1397.9496738117427 | 4.53577716319e-10 | 1397.9496738117427 | 0.105673396395 |
| 1386.9625520110958 | 390.072674987 | 1386.9625520110958 | 6.34719506645e-10 | 1386.9625520110958 | 0.128317001388 |
| 1376.1467889908256 | 421.468051961 | 1376.1467889908256 | 8.86439896066e-10 | 1376.1467889908256 | 0.155512784534 |
| 1365.4984069185252 | 454.502740219 | 1365.4984069185252 | 1.23553216457e-09 | 1365.4984069185252 | 0.188109987292 |
| 1355.0135501355014 | 489.172087793 | 1355.0135501355014 | 1.71868438296e-09 | 1355.0135501355014 | 0.227102533424 |
| 1344.688480502017 | 525.461341632 | 1344.688480502017 | 2.3860279062e-09 | 1344.688480502017 | 0.273650966549 |
| 1334.5195729537365 | 563.345019577 | 1334.5195729537365 | 3.30591908695e-09 | 1334.5195729537365 | 0.329107209958 |
| 1324.5033112582782 | 602.786344223 | 1324.5033112582782 | 4.57136853091e-09 | 1324.5033112582782 | 0.395042423146 |
| 1314.6362839614374 | 643.736749873 | 1314.6362839614374 | 6.30866638882e-09 | 1314.6362839614374 | 0.473278241155 |
| 1304.9151805132665 | 686.135473524 | 1304.9151805132665 | 8.6889280852e-09 | 1304.9151805132665 | 0.565921692073 |
| 1295.3367875647668 | 729.90924039 | 1295.3367875647668 | 1.19435149325e-08 | 1295.3367875647668 | 0.675404094413 |
| 1285.8979854264894 | 774.972053801 | 1285.8979854264894 | 1.63845841902e-08 | 1285.8979854264894 | 0.804524238773 |
| 1276.5957446808509 | 821.225098503 | 1276.5957446808509 | 2.2432413668e-08 | 1276.5957446808509 | 0.956496156533 |
| 1267.427122940431 | 868.556765319 | 1267.427122940431 | 3.0651652913e-08 | 1267.427122940431 | 1.13500177146 |
| 1258.3892617449665 | 916.842803912 | 1258.3892617449665 | 4.17993094137e-08 | 1258.3892617449665 | 1.34424871714 |
| 1249.4793835901708 | 965.946608968 | 1249.4793835901708 | 5.68881260086e-08 | 1249.4793835901708 | 1.58903358322 |
| 1240.6947890818858 | 1015.71964357 | 1240.6947890818858 | 7.7270097823e-08 | 1240.6947890818858 | 1.87481082578 |
| 1232.0328542094455 | 1066.00200178 | 1232.0328542094455 | 1.04746280444e-07 | 1232.0328542094455 | 2.20776754015 |
| 1223.4910277324632 | 1116.62311067 | 1223.4910277324632 | 1.41710839769e-07 | 1223.4910277324632 | 2.59490424854 |
| 1215.0668286755772 | 1167.40256999 | 1215.0668286755772 | 1.91339593467e-07 | 1215.0668286755772 | 3.04412179716 |
| 1206.7578439259853 | 1218.15112589 | 1206.7578439259853 | 2.57836226109e-07 | 1206.7578439259853 | 3.56431438927 |
| 1198.5617259288852 | 1268.67177283 | 1198.5617259288852 | 3.46753089042e-07 | 1198.5617259288852 | 4.16546869913 |
| 1190.4761904761904 | 1318.76097603 | 1190.4761904761904 | 4.65408226262e-07 | 1190.4761904761904 | 4.8587689181 |
| 1182.4990145841543 | 1368.21000459 | 1182.4990145841543 | 6.23426251777e-07 | 1182.4990145841543 | 5.65670747622 |
| 1174.6280344557556 | 1416.80636389 | 1174.6280344557556 | 8.33438254809e-07 | 1174.6280344557556 | 6.57320106133 |
| 1166.8611435239206 | 1464.33531368 | 1166.8611435239206 | 1.11198541139e-06 | 1166.8611435239206 | 7.62371142197 |
| 1159.19629057187 | 1510.58145689 | 1159.19629057187 | 1.48068290653e-06 | 1159.19629057187 | 8.82537029112 |
| 1151.6314779270633 | 1555.33038275 | 1151.6314779270633 | 1.96771569829e-06 | 1151.6314779270633 | 10.1971076047 |
| 1144.1647597254005 | 1598.37034656 | 1144.1647597254005 | 2.60975628766e-06 | 1144.1647597254005 | 11.7597820134 |
| 1136.794240242516 | 1639.49396735 | 1136.794240242516 | 3.45441785318e-06 | 1136.794240242516 | 13.536312498 |
| 1129.5180722891566 | 1678.49992414 | 1129.5180722891566 | 4.56338490359e-06 | 1129.5180722891566 | 15.5518097036 |
| 1122.334455667789 | 1715.19463104 | 1122.334455667789 | 6.01639924752e-06 | 1122.334455667789 | 17.8337053985 |
| 1115.2416356877322 | 1749.3938712 | 1115.2416356877322 | 7.91632308395e-06 | 1115.2416356877322 | 20.4118782568 |
| 1108.2379017362393 | 1780.92437006 | 1108.2379017362393 | 1.03955551853e-05 | 1108.2379017362393 | 23.3187739451 |
| 1101.3215859030836 | 1809.62528845 | 1101.3215859030836 | 1.36241426358e-05 | 1101.3215859030836 | 26.5895172849 |
| 1094.4910616563297 | 1835.34961742 | 1094.4910616563297 | 1.78200119664e-05 | 1094.4910616563297 | 30.2620140471 |
| 1087.7447425670775 | 1857.9654573 | 1087.7447425670775 | 2.32618428411e-05 | 1087.7447425670775 | 34.37703974 |
| 1081.081081081081 | 1877.35716525 | 1081.081081081081 | 3.03052282894e-05 | 1081.081081081081 | 38.9783125597 |
| 1074.4985673352435 | 1893.42635689 | 1074.4985673352435 | 3.94029120757e-05 | 1074.4985673352435 | 44.1125475063 |
| 1067.995728017088 | 1906.09274984 | 1067.995728017088 | 5.11300711095e-05 | 1067.995728017088 | 49.8294885216 |
| 1061.5711252653928 | 1915.29483875 | 1061.5711252653928 | 6.62158245969e-05 | 1061.5711252653928 | 56.181915389 |
| 1055.2233556102708 | 1920.99039384 | 1055.2233556102708 | 8.55824086469e-05 | 1055.2233556102708 | 63.225622056 |
| 1048.951048951049 | 1923.15677732 | 1048.951048951049 | 0.000110393763024 | 1048.951048951049 | 71.0193630004 |
| 1042.752867570386 | 1921.79107438 | 1042.752867570386 | 0.00014211564463 | 1042.752867570386 | 79.6247642705 |
| 1036.6275051831374 | 1916.9100382 | 1036.6275051831374 | 0.000182589820347 | 1036.6275051831374 | 89.1061958886 |
| 1030.5736860185502 | 1908.54985069 | 1030.5736860185502 | 0.000234125411727 | 1030.5736860185502 | 99.5306024309 |
| 1024.5901639344263 | 1896.76570315 | 1024.5901639344263 | 0.000299611078937 | 1024.5901639344263 | 110.967288777 |
| 1018.6757215619693 | 1881.63120357 | 1018.6757215619693 | 0.00038265245627 | 1018.6757215619693 | 123.487658273 |
| 1012.829169480081 | 1863.23761923 | 1012.829169480081 | 0.000487740103083 | 1012.829169480081 | 137.164900888 |
| 1007.0493454179255 | 1841.6929657 | 1007.0493454179255 | 0.000620454230978 | 1007.0493454179255 | 152.073629307 |
| 1001.3351134846461 | 1817.12095481 | 1001.3351134846461 | 0.000787713630818 | 1001.3351134846461 | 168.289461433 |
| 995.6853634251576 | 1789.65981604 | 995.6853634251576 | 0.000998077574632 | 995.6853634251576 | 185.888548251 |
| 990.0990099009902 | 1759.46100716 | 990.0990099009902 | 0.00126211103206 | 990.0990099009902 | 204.947046696 |
| 984.5749917952082 | 1726.68783098 | 984.5749917952082 | 0.00159282534556 | 984.5749917952082 | 225.540537813 |
| 979.1122715404699 | 1691.51397585 | 979.1122715404699 | 0.0020062085816 | 979.1122715404699 | 247.743391291 |
| 973.7098344693281 | 1654.12199837 | 973.7098344693281 | 0.00252186214673 | 973.7098344693281 | 271.628078261 |
| 968.3666881859263 | 1614.70176664 | 968.3666881859263 | 0.00316376295921 | 968.3666881859263 | 297.264435146 |
| 963.0818619582664 | 1573.44888263 | 963.0818619582664 | 0.00396117353192 | 963.0818619582664 | 324.718882241 |
| 957.8544061302682 | 1530.56310185 | 957.8544061302682 | 0.00494972578261 | 957.8544061302682 | 354.053601704 |
| 952.6833915528738 | 1486.24676794 | 952.6833915528738 | 0.006172708277 | 952.6833915528738 | 385.325680567 |
| 947.5679090334806 | 1440.70327881 | 947.5679090334806 | 0.00768259095991 | 947.5679090334806 | 418.586225387 |
| 942.5070688030161 | 1394.13560036 | 942.5070688030161 | 0.00954282626879 | 942.5070688030161 | 453.879456113 |
| 937.4999999999999 | 1346.74484199 | 937.4999999999999 | 0.0118299708793 | 937.4999999999999 | 491.241787692 |
| 932.5458501709667 | 1298.72890727 | 932.5458501709667 | 0.014636178225 | 932.5458501709667 | 530.700908807 |
| 927.643784786642 | 1250.28123114 | 927.643784786642 | 0.0180721183757 | 927.643784786642 | 572.274868005 |
| 922.7929867733004 | 1201.58961385 | 922.7929867733004 | 0.0222703888611 | 922.7929867733004 | 615.971178167 |
| 917.9926560587514 | 1152.83515963 | 917.9926560587514 | 0.0273894875776 | 917.9926560587514 | 661.78595093 |
| 913.2420091324201 | 1104.19132679 | 913.2420091324201 | 0.0336184270019 | 913.2420091324201 | 709.703073188 |
| 908.5402786190186 | 1055.82309386 | 908.5402786190186 | 0.0411820775216 | 908.5402786190186 | 759.69343813 |
| 903.8867128653209 | 1007.88624505 | 903.8867128653209 | 0.050347336721 | 903.8867128653209 | 811.714243506 |
| 899.2805755395683 | 960.526776004 | 899.2805755395683 | 0.0614302308602 | 899.2805755395683 | 865.708369831 |
| 894.7211452430658 | 913.880420003 | 894.7211452430658 | 0.074804064452 | 894.7211452430658 | 921.603851078 |
| 890.2077151335311 | 868.072292562 | 890.2077151335311 | 0.0909087436482 | 890.2077151335311 | 979.313450035 |
| 885.7395925597874 | 823.216651426 | 885.7395925597874 | 0.110261408924 | 885.7395925597874 | 1038.73434997 |
| 881.316098707403 | 779.416767485 | 881.316098707403 | 0.133468522106 | 881.316098707403 | 1099.74797343 |
| 876.9365682548962 | 736.764901103 | 876.9365682548962 | 0.161239561902 | 876.9365682548962 | 1162.219938 |
| 872.6003490401396 | 695.34237733 | 872.6003490401396 | 0.194402490423 | 872.6003490401396 | 1226.00015777 |
| 868.3068017366135 | 655.219752684 | 868.3068017366135 | 0.233921160548 | 868.3068017366135 | 1290.92309761 |
| 864.0552995391705 | 616.457065526 | 864.0552995391705 | 0.280914839803 | 864.0552995391705 | 1356.808186 |
| 859.8452278589854 | 579.104161536 | 859.8452278589854 | 0.336680030552 | 859.8452278589854 | 1423.4603904 |
| 855.6759840273816 | 543.201085462 | 855.6759840273816 | 0.402714767993 | 855.6759840273816 | 1490.67095682 |
| 851.5469770082316 | 508.778530123 | 851.5469770082316 | 0.480745576506 | 851.5469770082316 | 1558.21831391 |
| 847.457627118644 | 475.858333558 | 847.457627118644 | 0.572757260486 | 847.457627118644 | 1625.86913901 |
| 843.4073657576608 | 444.454015296 | 843.4073657576608 | 0.681025697617 | 843.4073657576608 | 1693.37958186 |
| 839.3956351426972 | 414.571342925 | 839.3956351426972 | 0.808153789733 | 839.3956351426972 | 1760.49663913 |
| 835.421888053467 | 386.208920368 | 835.421888053467 | 0.957110708612 | 835.421888053467 | 1826.95967096 |
| 831.4855875831485 | 359.35878974 | 831.4855875831485 | 1.13127455036 | 831.4855875831485 | 1892.50204798 |
| 827.5862068965516 | 334.007039027 | 827.5862068965516 | 1.33447848208 | 827.5862068965516 | 1956.85291574 |
| 823.7232289950576 | 310.13440843 | 823.7232289950576 | 1.5710604276 | 823.7232289950576 | 2019.73906083 |
| 819.8961464881114 | 287.716888751 | 819.8961464881114 | 1.84591629427 | 819.8961464881114 | 2080.88686155 |
| 816.1044613710554 | 266.726305816 | 816.1044613710554 | 2.16455669066 | 816.1044613710554 | 2140.02430399 |
| 812.3476848090983 | 247.130885596 | 812.3476848090983 | 2.53316702357 | 812.3476848090983 | 2196.88304305 |
| 808.6253369272237 | 228.895795321 | 808.6253369272237 | 2.95867079316 | 808.6253369272237 | 2251.20048661 |
| 804.9369466058491 | 211.983656562 | 804.9369466058491 | 3.44879582622 | 804.9369466058491 | 2302.72188026 |
| 801.2820512820513 | 196.355026922 | 801.2820512820513 | 4.01214309994 | 801.2820512820513 | 2351.20236911 |
| 797.6601967561818 | 181.968847598 | 797.6601967561818 | 4.65825771191 | 797.6601967561818 | 2396.40901336 |
| 794.0709370037056 | 168.782854762 | 794.0709370037056 | 5.39770144768 | 794.0709370037056 | 2438.12273374 |
| 790.5138339920949 | 156.753953254 | 790.5138339920949 | 6.24212628413 | 790.5138339920949 | 2476.14016394 |
| 786.9884575026232 | 145.838551717 | 786.9884575026232 | 7.20434804843 | 786.9884575026232 | 2510.27538748 |
| 783.4943849569078 | 135.992858803 | 783.4943849569078 | 8.29841932709 | 783.4943849569078 | 2540.3615378 |
| 780.0312012480499 | 127.173140624 | 780.0312012480499 | 9.53970059145 | 780.0312012480499 | 2566.25224158 |
| 776.598498576236 | 119.335940062 | 776.598498576236 | 10.9449283751 | 776.598498576236 | 2587.82288737 |
| 773.1958762886597 | 112.438259021 | 773.1958762886597 | 12.5322792087 | 773.1958762886597 | 2604.97170335 |
| 769.8229407236336 | 106.43770504 | 769.8229407236336 | 14.3214278906 | 769.8229407236336 | 2617.62063048 |
| 766.4793050587633 | 101.292604091 | 766.4793050587633 | 16.3335985487 | 766.4793050587633 | 2625.71597998 |
| 763.1645891630628 | 96.9620816582 | 763.1645891630628 | 18.5916068387 | 763.1645891630628 | 2629.22886661 |
| 759.8784194528876 | 93.4061144484 | 759.8784194528876 | 21.1198915236 | 759.8784194528876 | 2628.15541213 |
| 756.6204287515762 | 90.5855553535 | 756.6204287515762 | 23.9445335938 | 756.6204287515762 | 2622.51671659 |
| 753.390256152687 | 88.46213441 | 753.390256152687 | 27.0932610289 | 753.390256152687 | 2612.35859769 |
| 750.1875468867216 | 86.998438686 | 750.1875468867216 | 30.5954372611 | 750.1875468867216 | 2597.75110188 |
| 747.011952191235 | 86.1578741063 | 747.011952191235 | 34.4820313928 | 747.011952191235 | 2578.7877935 |
| 743.86312918423 | 85.9046123005 | 743.86312918423 | 38.7855682452 | 743.86312918423 | 2555.58483159 |
| 740.7407407407408 | 86.2035255809 | 740.7407407407408 | 43.5400563767 | 740.7407407407408 | 2528.27984622 |
| 737.6444553725104 | 87.0201131515 | 737.6444553725104 | 48.7808923124 | 737.6444553725104 | 2497.03062907 |
| 734.5739471106758 | 88.3204216022 | 734.5739471106758 | 54.5447393733 | 734.5739471106758 | 2462.0136552 |
| 731.528895391368 | 90.070962663 | 731.528895391368 | 60.8693796899 | 731.528895391368 | 2423.42245499 |
| 728.5089849441475 | 92.2386310818 | 728.5089849441475 | 67.7935382271 | 728.5089849441475 | 2381.46585678 |
| 725.5139056831922 | 94.7906253459 | 725.5139056831922 | 75.3566779472 | 725.5139056831922 | 2336.36612239 |
| 722.543352601156 | 97.6943737908 | 722.543352601156 | 83.5987655807 | 722.543352601156 | 2288.35699862 |
| 719.5970256656271 | 100.917468442 | 719.5970256656271 | 92.5600078801 | 719.5970256656271 | 2237.68170835 |
| 716.6746297181079 | 104.427608701 | 716.6746297181079 | 102.280558675 | 716.6746297181079 | 2184.59090542 |
| 713.7758743754462 | 108.192556746 | 713.7758743754462 | 112.800197547 | 713.7758743754462 | 2129.34061716 |
| 710.9004739336492 | 112.180106232 | 710.9004739336492 | 124.157981487 | 710.9004739336492 | 2072.19019831 |
| 708.0481472740146 | 116.358065592 | 708.0481472740146 | 136.391871458 | 708.0481472740146 | 2013.40031882 |
| 705.2186177715091 | 120.69425694 | 705.2186177715091 | 149.53833642 | 705.2186177715091 | 1953.2310075 |
| 702.4116132053383 | 125.156531247 | 702.4116132053383 | 163.631937963 | 702.4116132053383 | 1891.93977162 |
| 699.6268656716418 | 129.712800162 | 699.6268656716418 | 178.704899395 | 699.6268656716418 | 1829.77981108 |
| 696.8641114982578 | 134.33108449 | 696.8641114982578 | 194.786663694 | 696.8641114982578 | 1766.99834399 |
| 694.1230911614992 | 138.979579058 | 694.1230911614992 | 211.903445441 | 694.1230911614992 | 1703.83505811 |
| 691.4035492048858 | 143.626733342 | 691.4035492048858 | 230.077782405 | 691.4035492048858 | 1640.52070079 |
| 688.7052341597796 | 148.241346954 | 688.7052341597796 | 249.328093021 | 688.7052341597796 | 1577.27581715 |
| 686.027898467871 | 152.792678779 | 686.027898467871 | 269.668246547 | 686.027898467871 | 1514.30964444 |
| 683.371298405467 | 157.250568283 | 683.371298405467 | 291.107153076 | 683.371298405467 | 1451.81916761 |
| 680.7351940095303 | 161.585567282 | 680.7351940095303 | 313.648380973 | 680.7351940095303 | 1389.98833904 |
| 678.1193490054249 | 165.769080213 | 678.1193490054249 | 337.289809543 | 678.1193490054249 | 1328.98746278 |
| 675.5235307363206 | 169.77351081 | 675.5235307363206 | 362.023324876 | 675.5235307363206 | 1268.97274166 |
| 672.9475100942127 | 173.572412881 | 672.9475100942127 | 387.834566836 | 672.9475100942127 | 1210.08598336 |
| 670.3910614525139 | 177.14064283 | 670.3910614525139 | 414.70273502 | 670.3910614525139 | 1152.45445962 |
| 667.8539626001781 | 180.454511439 | 667.8539626001781 | 442.600461242 | 667.8539626001781 | 1096.19091115 |
| 665.335994677312 | 183.491932444 | 665.335994677312 | 471.493755664 | 665.335994677312 | 1041.39368903 |
| 662.8369421122403 | 186.232565421 | 662.8369421122403 | 501.342033076 | 662.8369421122403 | 988.147022337 |
| 660.3565925599823 | 188.65795057 | 660.3565925599823 | 532.098225098 | 660.3565925599823 | 936.521400432 |
| 657.8947368421053 | 190.751633068 | 657.8947368421053 | 563.708983108 | 657.8947368421053 | 886.574057747 |
| 655.4511688879178 | 192.499274819 | 655.4511688879178 | 596.114975646 | 655.4511688879178 | 838.349548106 |
| 653.0256856769699 | 193.888751581 | 653.0256856769699 | 629.25128278 | 653.0256856769699 | 791.88039541 |
| 650.6180871828237 | 194.910233685 | 650.6180871828237 | 663.047888557 | 650.6180871828237 | 747.187807357 |
| 648.2281763180639 | 195.556248768 | 648.2281763180639 | 697.430271149 | 648.2281763180639 | 704.282438968 |
| 645.8557588805166 | 195.821725262 | 645.8557588805166 | 732.320088701 | 645.8557588805166 | 663.165192993 |
| 643.5006435006435 | 195.704015602 | 643.5006435006435 | 767.635957197 | 643.5006435006435 | 623.828044771 |
| 641.1626415900834 | 195.202898492 | 641.1626415900834 | 803.294314927 | 641.1626415900834 | 586.254879756 |
| 638.8415672913118 | 194.320559826 | 638.8415672913118 | 839.210366334 | 638.8415672913118 | 550.422332699 |
| 636.5372374283895 | 193.061552231 | 636.5372374283895 | 875.299096313 | 636.5372374283895 | 516.300618405 |
| 634.2494714587738 | 191.432733485 | 634.2494714587738 | 911.476344244 | 634.2494714587738 | 483.854344938 |
| 631.9780914261638 | 189.443184396 | 631.9780914261638 | 947.659925441 | 631.9780914261638 | 453.043301234 |
| 629.7229219143577 | 187.104107033 | 629.7229219143577 | 983.770786132 | 629.7229219143577 | 423.82321215 |
| 627.4837900020916 | 184.428704481 | 627.4837900020916 | 1019.73417669 | 627.4837900020916 | 396.146455112 |
| 625.2605252188412 | 181.43204354 | 625.2605252188412 | 1055.48082667 | 625.2605252188412 | 369.962733628 |
| 623.0529595015576 | 178.130902073 | 623.0529595015576 | 1090.94810405 | 623.0529595015576 | 345.219703999 |
| 620.8609271523178 | 174.543602842 | 620.8609271523178 | 1126.08114067 | 620.8609271523178 | 321.86355264 |
| 618.6842647968654 | 170.689835923 | 618.6842647968654 | 1160.83390485 | 618.6842647968654 | 299.839522369 |
| 616.5228113440197 | 166.590471849 | 616.5228113440197 | 1195.17020258 | 616.5228113440197 | 279.092386975 |
| 614.3764079459348 | 162.267367807 | 614.3764079459348 | 1229.06458833 | 614.3764079459348 | 259.566874184 |
| 612.2448979591836 | 157.743169188 | 612.2448979591836 | 1262.50316731 | 612.2448979591836 | 241.208037908 |
| 610.1281269066504 | 153.041108899 | 610.1281269066504 | 1295.48427161 | 610.1281269066504 | 223.961581305 |
| 608.0259424402108 | 148.184806751 | 608.0259424402108 | 1328.01899401 | 608.0259424402108 | 207.774132743 |
| 605.9381943041809 | 143.198071221 | 605.9381943041809 | 1360.13156455 | 605.9381943041809 | 192.593477211 |
| 603.864734299517 | 138.104705798 | 603.864734299517 | 1391.85955686 | 603.864734299517 | 178.368746105 |
| 601.8054162487462 | 132.928321973 | 601.8054162487462 | 1423.25391339 | 601.8054162487462 | 165.050568567 |
| 599.7600959616153 | 127.692160802 | 599.7600959616153 | 1454.37878103 | 599.7600959616153 | 152.59118774 |
| 597.7286312014345 | 122.418924795 | 597.7286312014345 | 1485.31115134 | 597.7286312014345 | 140.944545441 |
| 595.7108816521048 | 117.130621658 | 595.7108816521048 | 1516.14030242 | 595.7108816521048 | 130.066338717 |
| 593.7067088858104 | 111.848421238 | 593.7067088858104 | 1546.96704245 | 593.7067088858104 | 119.914051783 |
| 591.7159763313609 | 106.592526751 | 591.7159763313609 | 1577.90275827 | 591.7159763313609 | 110.446966688 |
| 589.7385492431688 | 101.382061174 | 589.7385492431688 | 1609.06827537 | 589.7385492431688 | 101.626155945 |
| 587.7742946708463 | 96.2349694046 | 587.7742946708463 | 1640.59253893 | 587.7742946708463 | 93.4144601541 |
| 585.8230814294083 | 91.1679365831 | 585.8230814294083 | 1672.61112893 | 585.8230814294083 | 85.7764534583 |
| 583.8847800700661 | 86.1963227159 | 583.8847800700661 | 1705.26462512 | 583.8847800700661 | 78.6783993955 |
| 581.9592628516003 | 81.3341135261 | 581.9592628516003 | 1738.69684066 | 581.9592628516003 | 72.0881994918 |
| 580.046403712297 | 76.5938872337 | 580.046403712297 | 1773.05294605 | 580.046403712297 | 65.9753366546 |
| 578.1460782424359 | 71.9867967783 | 578.1460782424359 | 1808.47750689 | 578.1460782424359 | 60.3108151732 |
| 576.2581636573184 | 67.5225668104 | 576.2581636573184 | 1845.11246169 | 576.2581636573184 | 55.0670988687 |
| 574.3825387708214 | 63.2095046233 | 574.3825387708214 | 1883.09506681 | 574.3825387708214 | 50.2180486883 |
| 572.5190839694656 | 59.0545240566 | 572.5190839694656 | 1922.55583757 | 572.5190839694656 | 45.7388608023 |
| 570.6676811869887 | 55.0631812884 | 570.6676811869887 | 1963.61651466 | 570.6676811869887 | 41.6060060399 |
| 568.8282138794084 | 51.2397213434 | 568.8282138794084 | 2006.38808569 | 568.8282138794084 | 37.7971713032 |
| 567.000567000567 | 47.5871340751 | 567.000567000567 | 2050.96889113 | 567.000567000567 | 34.2912034146 |
| 565.1846269781461 | 44.1072183383 | 565.1846269781461 | 2097.44284327 | 565.1846269781461 | 31.0680556968 |
| 563.3802816901408 | 40.8006530443 | 563.3802816901408 | 2145.87778554 | 563.3802816901408 | 28.1087374473 |
| 561.5874204417821 | 37.6670737942 | 561.5874204417821 | 2196.32401761 | 561.5874204417821 | 25.3952663551 |
| 559.8059339428997 | 34.7051538025 | 559.8059339428997 | 2248.81300951 | 559.8059339428997 | 22.9106238124 |
| 558.0357142857143 | 31.9126878639 | 558.0357142857143 | 2303.35632526 | 558.0357142857143 | 20.6387130019 |
| 556.2766549230483 | 29.2866781683 | 556.2766549230483 | 2359.94477338 | 556.2766549230483 | 18.5643195833 |
| 554.52865064695 | 26.8234208394 | 554.52865064695 | 2418.54779826 | 554.52865064695 | 16.6730747635 |
| 552.791597567717 | 24.5185921495 | 552.791597567717 | 2479.11312244 | 552.791597567717 | 14.9514205135 |
| 551.0653930933137 | 22.3673334554 | 551.0653930933137 | 2541.56664618 | 551.0653930933137 | 13.3865766796 |
| 549.3499359091741 | 20.3643339948 | 549.3499359091741 | 2605.81260629 | 549.3499359091741 | 11.9665097409 |
| 547.645125958379 | 18.5039107851 | 547.645125958379 | 2671.73399228 | 547.645125958379 | 10.6799029699 |
| 545.950864422202 | 16.7800849696 | 545.950864422202 | 2739.1932136 | 545.950864422202 | 9.51612777092 |
| 544.2670537010159 | 15.1866540637 | 544.2670537010159 | 2808.0330078 | 544.2670537010159 | 8.46521599263 |
| 542.5935973955508 | 13.7172596536 | 542.5935973955508 | 2878.07757541 | 542.5935973955508 | 7.51783303317 |
| 540.9304002884962 | 12.3654502059 | 540.9304002884962 | 2949.13392409 | 540.9304002884962 | 6.66525158676 |
| 539.2773683264425 | 11.1247387391 | 539.2773683264425 | 3020.99340081 | 539.2773683264425 | 5.89932590632 |
| 537.6344086021505 | 9.98865520175 | 537.6344086021505 | 3093.43338856 | 537.6344086021505 | 5.21246648577 |
| 536.0014293371448 | 8.95079348759 | 536.0014293371448 | 3166.21914114 | 536.0014293371448 | 4.59761509217 |
| 534.3783398646241 | 8.00485309361 | 534.3783398646241 | 3239.10572811 | 534.3783398646241 | 4.04822010318 |
| 532.7650506126798 | 7.14467549806 | 532.7650506126798 | 3311.84006038 | 532.7650506126798 | 3.55821212809 |
| 531.1614730878186 | 6.36427539704 | 531.1614730878186 | 3384.16296623 | 531.1614730878186 | 3.1219799108 |
| 529.5675198587819 | 5.65786699059 | 529.5675198587819 | 3455.81128752 | 529.5675198587819 | 2.73434653039 |
| 527.9831045406547 | 5.01988555446 | 527.9831045406547 | 3526.51996595 | 527.9831045406547 | 2.39054592894 |
| 526.4081417792595 | 4.44500456964 | 526.4081417792595 | 3596.02409058 | 526.4081417792595 | 2.08619980721 |
| 524.8425472358292 | 3.92814870998 | 524.8425472358292 | 3664.06087916 | 524.8425472358292 | 1.81729493716 |
| 523.2862375719518 | 3.46450300905 | 523.2862375719518 | 3730.37156799 | 523.2862375719518 | 1.58016094498 |
| 521.7391304347826 | 3.04951854056 | 521.7391304347826 | 3794.7031875 | 521.7391304347826 | 1.37144862139 |
| 520.2011444425177 | 2.67891495396 | 520.2011444425177 | 3856.81020377 | 520.2011444425177 | 1.18810881603 |
| 518.6721991701245 | 2.34868020761 | 518.6721991701245 | 3916.45600943 | 518.6721991701245 | 1.02737197105 |
| 517.1522151353215 | 2.05506783789 | 517.1522151353215 | 3973.414251 | 517.1522151353215 | 0.886728345521 |
| 515.6411137848057 | 1.79459209389 | 515.6411137848057 | 4027.46998333 | 515.6411137848057 | 0.7639089777 |
| 514.1388174807198 | 1.56402125483 | 514.1388174807198 | 4078.42064572 | 514.1388174807198 | 0.656867425817 |
| 512.6452494873547 | 1.36036943144 | 512.6452494873547 | 4126.07685805 | 512.6452494873547 | 0.563762321783 |
| 511.1603339580848 | 1.1808871346 | 511.1603339580848 | 4170.26303894 | 511.1603339580848 | 0.482940764727 |
| 509.683995922528 | 1.02305087425 | 509.683995922528 | 4210.81785152 | 509.683995922528 | 0.412922573891 |
| 508.2161612739285 | 0.88455203006 | 508.2161612739285 | 4247.59448567 | 508.2161612739285 | 0.352385412907 |
| 506.7567567567567 | 0.763285213555 | 506.7567567567567 | 4280.46078846 | 506.7567567567567 | 0.300150790115 |
| 505.3057099545225 | 0.65733631823 | 505.3057099545225 | 4309.29925716 | 505.3057099545225 | 0.255170932563 |
| 503.8629492777964 | 0.564970432365 | 503.8629492777964 | 4334.00691116 | 503.8629492777964 | 0.21651652475 |
| 502.4284039524367 | 0.484619766911 | 502.4284039524367 | 4354.49506087 | 502.4284039524367 | 0.183365297151 |
| 501.00200400801606 | 0.414871729738 | 501.00200400801606 | 4370.68899257 | 501.00200400801606 | 0.154991444181 |
| 499.5836802664446 | 0.354457257332 | 499.5836802664446 | 4382.52758891 | 499.5836802664446 | 0.130755846494 |
| 498.1733643307871 | 0.302239496015 | 498.1733643307871 | 4389.96290453 | 498.1733643307871 | 0.110097068524 |
| 496.7709885742673 | 0.257202907115 | 496.7709885742673 | 4392.95971597 | 496.7709885742673 | 0.0925230988276 |
| 495.3764861294584 | 0.218442854301 | 495.3764861294584 | 4391.49506379 | 495.3764861294584 | 0.0776037981398 |
| 493.98979087765514 | 0.185155716599 | 493.98979087765514 | 4385.55780344 | 493.98979087765514 | 0.0649640181125 |
| 492.61083743842363 | 0.156629557436 | 492.61083743842363 | 4375.14817961 | 492.61083743842363 | 0.0542773523095 |
| 491.2395611593253 | 0.132235368364 | 491.2395611593253 | 4360.27743623 | 491.2395611593253 | 0.0452604802847 |
| 489.8758981058131 | 0.111418895994 | 489.8758981058131 | 4340.96747217 | 489.8758981058131 | 0.0376680653007 |
| 488.5197850512946 | 0.0936930518509 | 488.5197850512946 | 4317.25054961 | 488.5197850512946 | 0.0312881664625 |
| 487.17115946735953 | 0.0786308975189 | 487.17115946735953 | 4289.16905942 | 487.17115946735953 | 0.0259381266571 |
| 485.82995951416996 | 0.0658591912577 | 485.82995951416996 | 4256.77534491 | 485.82995951416996 | 0.0214608986564 |
| 484.49612403100775 | 0.055052477331 | 484.49612403100775 | 4220.13158242 | 484.49612403100775 | 0.0177217730009 |
| 483.16959252697694 | 0.0459276953583 | 483.16959252697694 | 4179.30971465 | 483.16959252697694 | 0.0146054727703 |
| 481.8503051718599 | 0.0382392840597 | 481.8503051718599 | 4134.39142988 | 481.8503051718599 | 0.0120135820237 |
| 480.5382027871216 | 0.0317747516559 | 480.5382027871216 | 4085.46817824 | 480.5382027871216 | 0.00986227650174 |
| 479.23322683706067 | 0.0263506838357 | 479.23322683706067 | 4032.64121382 | 479.23322683706067 | 0.00808032708133 |
| 477.9353194201051 | 0.0218091594916 | 477.9353194201051 | 3976.02165049 | 477.9353194201051 | 0.00660734842836 |
| 476.64442326024783 | 0.0180145442673 | 476.64442326024783 | 3915.73051736 | 476.64442326024783 | 0.00539226726079 |
| 475.3604816986214 | 0.0148506322626 | 475.3604816986214 | 3851.89879981 | 475.3604816986214 | 0.00439198659396 |
| 474.08343868520853 | 0.012218106919 | 474.08343868520853 | 3784.66745125 | 474.08343868520853 | 0.00357022426003 |
| 472.8132387706856 | 0.0100322930906 | 472.8132387706856 | 3714.18736116 | 472.8132387706856 | 0.00289650585785 |
| 471.5498270983967 | 0.00822117351813 | 471.5498270983967 | 3640.61926565 | 471.5498270983967 | 0.00234529407974 |
| 470.29314939645707 | 0.00672364431262 | 470.29314939645707 | 3564.13358762 | 470.29314939645707 | 0.00189523806626 |
| 469.04315196998124 | 0.00548798555877 | 469.04315196998124 | 3484.91019524 | 469.04315196998124 | 0.00152852804962 |
| 467.7997816934352 | 0.00447052473335 | 467.7997816934352 | 3403.13806909 | 467.7997816934352 | 0.00123034205429 |
| 466.5629860031104 | 0.00363447224803 | 466.5629860031104 | 3319.01487038 | 466.5629860031104 | 0.000988372827223 |
| 465.33271288971605 | 0.00294891004669 | 465.33271288971605 | 3232.74640508 | 465.33271288971605 | 0.000792424467483 |
| 464.10891089108907 | 0.00238791578326 | 464.10891089108907 | 3144.54598111 | 464.10891089108907 | 0.000634069417623 |
| 462.8915290850177 | 0.00192980665639 | 462.8915290850177 | 3054.6336583 | 462.8915290850177 | 0.000506357568194 |
| 461.68051708217905 | 0.0015564884668 | 461.68051708217905 | 2963.23539347 | 461.68051708217905 | 0.000403570216691 |
| 460.47582501918646 | 0.00125289687768 | 460.47582501918646 | 2870.58208552 | 460.47582501918646 | 0.000321012516569 |
| 459.2774035517452 | 0.00100651919094 | 459.2774035517452 | 2776.90852788 | 459.2774035517452 | 0.000254838856444 |
| 458.0852038479157 | 0.000806986195642 | 458.0852038479157 | 2682.45227783 | 458.0852038479157 | 0.000201906329477 |
| 456.89917758148033 | 0.000645724797213 | 456.89917758148033 | 2587.45245443 | 456.89917758148033 | 0.00015965209428 |
| 455.7192769254139 | 0.000515663196485 | 455.7192769254139 | 2492.14847858 | 455.7192769254139 | 0.00012599099748 |
| 454.54545454545456 | 0.00041098135642 | 454.54545454545456 | 2396.77877003 | 454.54545454545456 | 9.92303302775e-05 |
| 453.3776635937736 | 0.000326900375125 | 453.3776635937736 | 2301.57941772 | 453.3776635937736 | 7.79990330508e-05 |
| 452.2158577027434 | 0.000259505179062 | 452.2158577027434 | 2206.78284015 | 452.2158577027434 | 6.11890487692e-05 |
| 451.05999097880016 | 0.00020559566505 | 451.05999097880016 | 2112.6164533 | 451.05999097880016 | 4.79068634152e-05 |
| 449.9100179964007 | 0.000162562058512 | 449.9100179964007 | 2019.30136364 | 449.9100179964007 | 3.74335647526e-05 |
| 448.7658937920718 | 0.000128280823652 | 448.7658937920718 | 1927.05110314 | 448.7658937920718 | 2.9192004563e-05 |
| 447.6275738585497 | 0.000101027964362 | 447.6275738585497 | 1836.07042308 | 447.6275738585497 | 2.2719868346e-05 |
| 446.49501413900873 | 7.94069980451e-05 | 446.49501413900873 | 1746.55416198 | 446.49501413900873 | 1.76476445662e-05 |
| 445.36817102137763 | 6.22892736385e-05 | 445.36817102137763 | 1658.68620212 | 445.36817102137763 | 1.36806465882e-05 |
| 444.247001332741 | 4.87646451285e-05 | 444.247001332741 | 1572.63852722 | 444.247001332741 | 1.05843778807e-05 |
| 443.13146233382565 | 3.81008077205e-05 | 443.13146233382565 | 1488.57039247 | 443.13146233382565 | 8.1726479493e-06 |
| 442.02151171357 | 2.97098603294e-05 | 442.02151171357 | 1406.62761596 | 442.02151171357 | 6.29794552169e-06 |
| 440.9171075837742 | 2.31208795166e-05 | 440.9171075837742 | 1326.94199848 | 440.9171075837742 | 4.8436591934e-06 |
| 439.8182084738308 | 1.7957480522e-05 | 439.8182084738308 | 1249.63087684 | 439.8182084738308 | 3.71780620922e-06 |
| 438.72477332553376 | 1.39195043306e-05 | 438.72477332553376 | 1174.79681334 | 438.72477332553376 | 2.84798919726e-06 |
| 437.636761487965 | 1.07681091687e-05 | 437.636761487965 | 1102.52742222 | 437.636761487965 | 2.17735015688e-06 |
| 436.5541327124563 | 8.31366349967e-06 | 436.5541327124563 | 1032.89533156 | 436.5541327124563 | 1.66133227407e-06 |
| 435.4768471476266 | 6.40593824369e-06 | 435.4768471476266 | 965.958277412 | 435.4768471476266 | 1.26509445548e-06 |
| 434.4048653344918 | 4.92618101142e-06 | 434.4048653344918 | 901.759324897 | 434.4048653344918 | 9.61451920626e-07 |
| 433.3381482016467 | 3.78072681125e-06 | 433.3381482016467 | 840.327209376 | 433.3381482016467 | 7.29239703493e-07 |
| 432.2766570605187 | 2.89585986176e-06 | 432.2766570605187 | 781.676789457 | 432.2766570605187 | 5.52015288435e-07 |
| 431.22035360069 | 2.21369150573e-06 | 431.22035360069 | 725.809602147 | 431.22035360069 | 4.17032522607e-07 |
| 430.1691998852882 | 1.68886124088e-06 | 430.1691998852882 | 672.714509533 | 430.1691998852882 | 3.14431986399e-07 |
| 429.1231583464454 | 1.28590282964e-06 | 429.1231583464454 | 622.368425536 | 429.1231583464454 | 2.36603654116e-07 |
| 428.0821917808219 | 9.77146426547e-07 | 428.0821917808219 | 574.737110708 | 428.0821917808219 | 1.77686352088e-07 |
| 427.0462633451957 | 7.41051613586e-07 | 427.0462633451957 | 529.77602269 | 427.0462633451957 | 1.33175566987e-07 |
| 426.01533655211585 | 5.60885973417e-07 | 426.01533655211585 | 487.431209861 | 426.01533655211585 | 9.96168633585e-08 |
| 424.9893752656184 | 4.23680049747e-07 | 424.9893752656184 | 447.640235775 | 424.9893752656184 | 7.43667778908e-08 |
| 423.96834369700395 | 3.19402831959e-07 | 423.96834369700395 | 410.333122316 | 423.96834369700395 | 5.54067697248e-08 |
| 422.9522064006767 | 2.40312755636e-07 | 422.9522064006767 | 375.433299929 | 422.9522064006767 | 4.11987873216e-08 |
| 421.9409282700422 | 1.80448051749e-07 | 421.9409282700422 | 342.858553977 | 421.9409282700422 | 3.05734004159e-08 |
| 420.93447453346425 | 1.35227458047e-07 | 420.93447453346425 | 312.521956985 | 420.93447453346425 | 2.26433531864e-08 |
| 419.9328107502799 | 1.01138121824e-07 | 419.9328107502799 | 284.332777463 | 419.9328107502799 | 1.6736914555e-08 |
| 418.93590280687056 | 7.5492220068e-08 | 418.93590280687056 | 258.197356929 | 418.93590280687056 | 1.2346609078e-08 |
| 417.94371691278906 | 5.62376057889e-08 | 417.94371691278906 | 234.019947808 | 417.94371691278906 | 9.08986882932e-09 |
| 416.9562195969423 | 4.18108274032e-08 | 416.9562195969423 | 211.703505934 | 416.9562195969423 | 6.67890299496e-09 |
| 415.97337770382694 | 3.10233015174e-08 | 415.97337770382694 | 191.150432481 | 415.97337770382694 | 4.89767810813e-09 |
| 414.99515838981876 | 2.29733631966e-08 | 414.99515838981876 | 172.263261212 | 414.99515838981876 | 3.58437060806e-09 |
| 414.0215291195142 | 1.6978466277e-08 | 414.0215291195142 | 154.94528801 | 414.0215291195142 | 2.61802100747e-09 |
| 413.0524576621231 | 1.25230365209e-08 | 413.0524576621231 | 139.101140645 | 413.0524576621231 | 1.90840648045e-09 |
| 412.08791208791206 | 9.21845522963e-09 | 412.08791208791206 | 124.637287721 | 412.08791208791206 | 1.38837300327e-09 |
| 411.1278607646978 | 6.77242130895e-09 | 411.1278607646978 | 111.462486601 | 411.1278607646978 | 1.0080427421e-09 |
| 410.17227235438884 | 4.96554700278e-09 | 410.17227235438884 | 99.4881709572 | 410.17227235438884 | 7.30447876369e-10 |
| 409.22111580957574 | 3.63351987278e-09 | 409.22111580957574 | 88.6287793008 | 409.22111580957574 | 5.28246952317e-10 |
| 408.2743603701687 | 2.65353790286e-09 | 408.2743603701687 | 78.8020264952 | 408.2743603701687 | 3.81260878538e-10 |
| 407.33197556008145 | 1.934017342e-09 | 407.33197556008145 | 69.9291208001 | 407.33197556008145 | 2.74628088005e-10 |
| 406.39393118396094 | 1.4068012501e-09 | 406.39393118396094 | 61.9349294537 | 406.39393118396094 | 1.97426350614e-10 |
| 405.46019732396263 | 1.0212743868e-09 | 405.46019732396263 | 54.7480961639 | 405.46019732396263 | 1.41645482722e-10 |
| 404.53074433656957 | 7.39927975462e-10 | 404.53074433656957 | 48.3011141551 | 404.53074433656957 | 1.01423309608e-10 |
| 403.6055428494551 | 5.35024621038e-10 | 403.6055428494551 | 42.5303586099 | 403.6055428494551 | 7.24786771396e-11 |
| 402.68456375838923 | 3.86096088199e-10 | 402.68456375838923 | 37.3760824632 | 402.68456375838923 | 5.16916221775e-11 |
| 401.76777822418643 | 2.78070135135e-10 | 401.76777822418643 | 32.7823795453 | 401.76777822418643 | 3.67931932058e-11 |
| 400.85515766969536 | 1.99871378293e-10 | 400.85515766969536 | 28.697119053 | 400.85515766969536 | 2.61367877259e-11 |
| 399.9466737768297 | 1.43378548574e-10 | 399.9466737768297 | 25.0718552461 | 399.9466737768297 | 1.85299541368e-11 |
| 399.0422984836393 | 1.02649080876e-10 | 399.0422984836393 | 21.8617161446 | 399.0422984836393 | 1.31109414064e-11 |
| 398.14200398142003 | 7.33437756814e-11 | 398.14200398142003 | 19.0252748268 | 398.14200398142003 | 9.2582904587e-12 |
| 397.24576271186436 | 5.23008533117e-11 | 397.24576271186436 | 16.5244067326 | 397.24576271186436 | 6.52476861419e-12 |
| 396.3535473642489 | 3.7221305276e-11 | 396.3535473642489 | 14.3241361443 | 396.3535473642489 | 4.5891981712e-12 |
| 395.46533087266016 | 2.64369735255e-11 | 395.46533087266016 | 12.3924747703 | 395.46533087266016 | 3.221409149e-12 |
| 394.5810864132579 | 1.87399827302e-11 | 394.5810864132579 | 10.7002550994 | 394.5810864132579 | 2.25679618356e-12 |
| 393.7007874015748 | 1.32575706299e-11 | 393.7007874015748 | 9.22096092334 | 393.7007874015748 | 1.57788795462e-12 |
| 392.82440748985204 | 9.36043486704e-12 | 392.82440748985204 | 7.93055715943 | 392.82440748985204 | 1.10102543178e-12 |
| 391.9519205644107 | 6.59576865982e-12 | 391.9519205644107 | 6.80732083983 | 391.9519205644107 | 7.66753742488e-13 |
| 391.08330074305826 | 4.63844185492e-12 | 391.08330074305826 | 5.83167487569 | 391.08330074305826 | 5.32907483945e-13 |
| 390.2185223725286 | 3.25548824819e-12 | 390.2185223725286 | 4.98602595835 | 390.2185223725286 | 3.69645218078e-13 |
| 389.3575600259571 | 2.28032873527e-12 | 389.3575600259571 | 4.25460772545 | 389.3575600259571 | 2.5589141778e-13 |
| 388.5003885003885 | 1.59410197927e-12 | 388.5003885003885 | 3.62333010159 | 388.5003885003885 | 1.76792444736e-13 |
| 387.6469828143171 | 1.11217225072e-12 | 387.6469828143171 | 3.0796355217 | 387.6469828143171 | 1.21901495174e-13 |
| 386.7973182052604 | 7.74399960169e-13 | 386.7973182052604 | 2.61236256141 | 386.7973182052604 | 8.38864298282e-14 |
| 385.95137012736393 | 5.38140777544e-13 | 385.95137012736393 | 2.21161733331 | 385.95137012736393 | 5.76118386954e-14 |
| 385.1091142490372 | 3.73219042893e-13 | 385.1091142490372 | 1.86865286111 | 385.1091142490372 | 3.94883606444e-14 |
| 384.2705264506212 | 2.58326524295e-13 | 384.2705264506212 | 1.57575651488 | 384.2705264506212 | 2.70124399585e-14 |
| 383.4355828220859 | 1.78447932075e-13 | 383.4355828220859 | 1.32614547923 | 383.4355828220859 | 1.8441483941e-14 |
| 382.6042596607575 | 1.23024427124e-13 | 382.6042596607575 | 1.11387013232 | 382.6042596607575 | 1.25650798616e-14 |
| 381.77653346907607 | 8.46464007448e-14 | 381.77653346907607 | 0.933725134636 | 381.77653346907607 | 8.54421127073e-15 |
| 380.95238095238096 | 5.81249984006e-14 | 380.95238095238096 | 0.781167963336 | 380.95238095238096 | 5.7985050414e-15 |
| 380.1317790167258 | 3.98340738966e-14 | 380.1317790167258 | 0.652244577132 | 380.1317790167258 | 3.92733034819e-15 |
| 379.31470476672143 | 2.72448115589e-14 | 379.31470476672143 | 0.543521859228 | 379.31470476672143 | 2.65470434527e-15 |
| 378.5011355034065 | 1.85973131983e-14 | 378.5011355034065 | 0.452026458469 | 378.5011355034065 | 1.79090364015e-15 |
| 377.69104872214524 | 1.266933777e-14 | 377.69104872214524 | 0.375189631613 | 377.69104872214524 | 1.20577314764e-15 |
| 376.88442211055275 | 8.61380304576e-15 | 376.88442211055275 | 0.310797680593 | 376.88442211055275 | 8.10207624741e-16 |
| 376.081233546446 | 5.84484869394e-15 | 376.081233546446 | 0.256947576811 | 376.081233546446 | 5.43330859252e-16 |
| 375.28146109582184 | 3.95811967968e-15 | 375.28146109582184 | 0.212007368695 | 375.28146109582184 | 3.63638406122e-16 |
| 374.48508301086 | 2.67511146812e-15 | 374.48508301086 | 0.174580977949 | 374.48508301086 | 2.42891571806e-16 |
| 373.69207772795215 | 1.80439725485e-15 | 373.69207772795215 | 0.143477003083 | 373.69207772795215 | 1.61917008203e-16 |
| 372.9024238657551 | 1.21467402702e-15 | 372.9024238657551 | 0.117681165076 | 372.9024238657551 | 1.07723342556e-16 |
| 372.11610022326965 | 8.16064800399e-16 | 372.11610022326965 | 0.0963320486803 | 372.11610022326965 | 7.15260916456e-17 |
| 371.33308577794276 | 5.47175771764e-16 | 371.33308577794276 | 0.0786998130621 | 371.33308577794276 | 4.73976153616e-17 |
| 370.55335968379444 | 3.66156194322e-16 | 370.55335968379444 | 0.0641675667869 | 370.55335968379444 | 3.1346265842e-17 |
| 369.7769012695673 | 2.44536237055e-16 | 369.7769012695673 | 0.0522151239 | 369.7769012695673 | 2.06896171671e-17 |
| 369.0036900369003 | 1.62988655529e-16 | 369.0036900369003 | 0.0424048797272 | 369.0036900369003 | 1.36287623466e-17 |
| 368.23370565852457 | 1.08419860131e-16 | 368.23370565852457 | 0.0343695665755 | 368.23370565852457 | 8.95978737137e-18 |
| 367.4669279764821 | 7.19776429951e-17 | 367.4669279764821 | 0.0278016705171 | 367.4669279764821 | 5.87863246439e-18 |
| 366.7033370003667 | 4.76896035698e-17 | 366.7033370003667 | 0.0224443106332 | 366.7033370003667 | 3.84939283906e-18 |
| 365.9429129055867 | 3.15345847502e-17 | 365.9429129055867 | 0.0180834013204 | 365.9429129055867 | 2.51562254987e-18 |
| 365.1856360316494 | 2.08107548742e-17 | 365.1856360316494 | 0.0145409363975 | 365.1856360316494 | 1.64072593575e-18 |
| 364.4314868804664 | 1.37064777049e-17 | 364.4314868804664 | 0.0116692507097 | 364.4314868804664 | 1.06798198326e-18 |
| 363.68044611468054 | 9.00951073673e-18 | 363.68044611468054 | 0.00934613066944 | 363.68044611468054 | 6.93791744326e-19 |
| 362.93249455601256 | 5.91035903055e-18 | 362.93249455601256 | 0.00747065968361 | 362.93249455601256 | 4.49812631799e-19 |
| 362.1876131836291 | 3.86958002431e-18 | 362.1876131836291 | 0.00595969770348 | 362.1876131836291 | 2.91052600952e-19 |
| 361.4457831325301 | 2.52843104757e-18 | 361.4457831325301 | 0.00474490622544 | 361.4457831325301 | 1.87952730573e-19 |
| 360.7069856919562 | 1.64882935049e-18 | 360.7069856919562 | 0.00377024101494 | 360.7069856919562 | 1.21133178072e-19 |
| 359.97120230381563 | 1.07309363203e-18 | 359.97120230381563 | 0.00298984467417 | 359.97120230381563 | 7.79138922904e-20 |
| 359.2384145611304 | 6.97006516895e-19 | 359.2384145611304 | 0.0023662799934 | 359.2384145611304 | 5.00154299368e-20 |
| 358.50860420650093 | 4.51828241194e-19 | 358.50860420650093 | 0.00186905288328 | 358.50860420650093 | 3.20427977523e-20 |
| 357.7817531305903 | 2.92312387197e-19 | 357.7817531305903 | 0.00147338065388 | 357.7817531305903 | 2.04877453322e-20 |
| 357.057843370626 | 1.88737578866e-19 | 357.057843370626 | 0.00115916755743 | 357.057843370626 | 1.30736008579e-20 |
| 356.33685710892024 | 1.21620519038e-19 | 356.33685710892024 | 0.000910154918961 | 356.33685710892024 | 8.32594604281e-21 |
| 355.6187766714082 | 7.82154662281e-20 | 355.6187766714082 | 0.000713217910823 | 355.6187766714082 | 5.29187135737e-21 |
| 354.9035845262037 | 5.02013894211e-20 | 354.9035845262037 | 0.000557785152396 | 354.9035845262037 | 3.35677536894e-21 |
| 354.1912632821723 | 3.21570461627e-20 | 354.1912632821723 | 0.000435360897088 | 354.1912632821723 | 2.12506685107e-21 |
| 353.48179568752204 | 2.05576692229e-20 | 353.48179568752204 | 0.000339132666097 | 353.48179568752204 | 1.34264200154e-21 |
| 352.77516462841015 | 1.31162266182e-20 | 352.77516462841015 | 0.000263649856733 | 352.77516462841015 | 8.46613474398e-22 |
| 352.07135312756714 | 8.35182262809e-21 | 352.07135312756714 | 0.000204561143657 | 352.07135312756714 | 5.32779417267e-22 |
| 351.3703443429374 | 5.30751128375e-21 | 351.3703443429374 | 0.000158400450601 | 351.3703443429374 | 3.34616240545e-22 |
| 350.6721215663355 | 3.36618439882e-21 | 350.6721215663355 | 0.000122412939892 | 350.6721215663355 | 2.09741276737e-22 |
| 349.9766682221185 | 2.13069944383e-21 | 349.9766682221185 | 9.4413885362e-05 | 349.9766682221185 | 1.31207334762e-22 |
| 349.2839678658749 | 1.34599608026e-21 | 349.2839678658749 | 7.26744947207e-05 | 349.2839678658749 | 8.19161692323e-23 |
| 348.59400418312805 | 8.48599381266e-22 | 348.59400418312805 | 5.58297602883e-05 | 348.59400418312805 | 5.10409171781e-23 |
| 347.90676098805517 | 5.33947969565e-22 | 347.90676098805517 | 4.2804268618e-05 | 347.90676098805517 | 3.17398316879e-23 |
| 347.2222222222222 | 3.3529917197e-22 | 347.2222222222222 | 3.27526133838e-05 | 347.2222222222222 | 1.96982698782e-23 |
| 346.54037195333257 | 2.10137390794e-22 | 346.54037195333257 | 2.50116523045e-05 | 346.54037195333257 | 1.22008155405e-23 |
| 345.8611943739912 | 1.31435143299e-22 | 345.8611943739912 | 1.90623456154e-05 | 345.8611943739912 | 7.54200738483e-24 |
| 345.1846738004832 | 8.20459189108e-23 | 345.1846738004832 | 1.44993260458e-05 | 345.1846738004832 | 4.65288543598e-24 |
| 344.5107946715664 | 5.11139892055e-23 | 344.5107946715664 | 1.10066916625e-05 | 344.5107946715664 | 2.86480508877e-24 |
| 343.8395415472779 | 3.17804383625e-23 | 343.8395415472779 | 8.33879470149e-06 | 343.8395415472779 | 1.76037468012e-24 |
| 343.17089910775564 | 1.97204718811e-23 | 343.17089910775564 | 6.305031334e-06 | 343.17089910775564 | 1.07957411061e-24 |
| 342.50485215207215 | 1.22127097341e-23 | 342.50485215207215 | 4.757827507e-06 | 342.50485215207215 | 6.60749925277e-25 |
| 341.84138559708293 | 7.54821184621e-24 | 341.84138559708293 | 3.58317156635e-06 | 341.84138559708293 | 4.0360737927e-25 |
| 341.1804844762879 | 4.65600500816e-24 | 341.1804844762879 | 2.69317126084e-06 | 341.1804844762879 | 2.46047177211e-25 |
| 340.522133938706 | 2.86628987842e-24 | 340.522133938706 | 2.02021591994e-06 | 340.522133938706 | 1.49697652353e-25 |
| 339.86631924776253 | 1.76101921352e-24 | 339.86631924776253 | 1.51240828773e-06 | 339.86631924776253 | 9.08968651918e-26 |
| 339.2130257801899 | 1.07980514286e-24 | 339.2130257801899 | 1.12999817524e-06 | 339.2130257801899 | 5.50833234481e-26 |
| 338.56223902494077 | 6.60790821301e-25 | 338.56223902494077 | 8.42604672339e-07 | 338.56223902494077 | 3.33141455453e-26 |
| 337.91394458211306 | 4.03570975876e-25 | 337.91394458211306 | 6.27057554632e-07 | 337.91394458211306 | 2.01082620159e-26 |
| 337.2681281618887 | 2.45987569333e-25 | 337.2681281618887 | 4.6572372282e-07 | 337.2681281618887 | 1.21131670298e-26 |
| 336.6247755834829 | 1.49638625474e-25 | 336.6247755834829 | 3.45212668445e-07 | 336.6247755834829 | 7.282461368e-27 |
| 335.9838727741068 | 9.08472054204e-26 | 335.9838727741068 | 2.55377418404e-07 | 335.9838727741068 | 4.36954268804e-27 |
| 335.3454057679409 | 5.50448569547e-26 | 335.3454057679409 | 1.88545276935e-07 | 335.3454057679409 | 2.61656240403e-27 |
| 334.709360705121 | 3.32858180989e-26 | 334.709360705121 | 1.38926858774e-07 | 334.709360705121 | 1.56373631048e-27 |
| 334.07572383073494 | 2.00881080888e-26 | 334.07572383073494 | 1.021631236e-07 | 334.07572383073494 | 9.32681275279e-28 |
| 333.44448149383123 | 1.20991859268e-26 | 333.44448149383123 | 7.49789740434e-08 | 333.44448149383123 | 5.5518830386e-28 |
| 332.81562014643885 | 7.27294959145e-27 | 332.81562014643885 | 5.49189473248e-08 | 332.81562014643885 | 3.29825836048e-28 |
| 332.1891263425977 | 4.36317183784e-27 | 332.1891263425977 | 4.0146000977e-08 | 332.1891263425977 | 1.95553840234e-28 |
| 331.5649867374005 | 2.61235004528e-27 | 331.5649867374005 | 2.92886766832e-08 | 331.5649867374005 | 1.15713848824e-28 |
| 330.9431880860452 | 1.56098142886e-27 | 330.9431880860452 | 2.13252719365e-08 | 330.9431880860452 | 6.83347551404e-29 |
| 330.323717242898 | 9.30896537889e-28 | 330.323717242898 | 1.54962556198e-08 | 330.323717242898 | 4.02749731192e-29 |
| 329.70656116056705 | 5.54041641035e-28 | 329.70656116056705 | 1.1238188911e-08 | 329.70656116056705 | 2.369006155e-29 |
| 329.0917068889864 | 3.29094566353e-28 | 329.0917068889864 | 8.13398270248e-09 | 329.0917068889864 | 1.39070313004e-29 |
| 328.47914157451 | 1.95090593326e-28 | 328.47914157451 | 5.87553635684e-09 | 328.47914157451 | 8.14779302603e-30 |
| 327.86885245901635 | 1.15422176717e-28 | 327.86885245901635 | 4.23573855433e-09 | 327.86885245901635 | 4.76412181667e-30 |
| 327.26082687902255 | 6.81521410436e-29 | 327.26082687902255 | 3.04753095899e-09 | 327.26082687902255 | 2.78011678751e-30 |
| 326.6550522648083 | 4.01612351273e-29 | 326.6550522648083 | 2.18828788666e-09 | 326.6550522648083 | 1.61912558651e-30 |
| 326.05151613955 | 2.36195669267e-29 | 326.05151613955 | 1.56818802879e-09 | 326.05151613955 | 9.41099070832e-31 |
| 325.4502061184639 | 1.38635391458e-29 | 325.4502061184639 | 1.12157716324e-09 | 325.4502061184639 | 5.45918065907e-31 |
| 324.8511099079588 | 8.12107663346e-30 | 324.8511099079588 | 8.00566707485e-10 | 324.8511099079588 | 3.16050805502e-31 |
| 324.25421530479895 | 4.74777814824e-30 | 324.25421530479895 | 5.70299820872e-10 | 324.25421530479895 | 1.82609618696e-31 |
| 323.65951019527455 | 2.77015799007e-30 | 323.65951019527455 | 4.05458384154e-10 | 323.65951019527455 | 1.05299839888e-31 |
| 323.0669825543829 | 1.61308024813e-30 | 323.0669825543829 | 2.87691263914e-10 | 323.0669825543829 | 6.05995061738e-32 |
| 322.4766204450177 | 9.37442677067e-31 | 322.4766204450177 | 2.03725034731e-10 | 322.4766204450177 | 3.4805491621e-32 |
| 321.88841201716735 | 5.43714332581e-31 | 321.88841201716735 | 1.43979102733e-10 | 321.88841201716735 | 1.99509588514e-32 |
| 321.3023455071222 | 3.14727110723e-31 | 321.3023455071222 | 1.01552790033e-10 | 321.3023455071222 | 1.14134538314e-32 |
| 320.71840923669015 | 1.81817148068e-31 | 320.71840923669015 | 7.14860976114e-11 | 320.71840923669015 | 6.51639966904e-33 |
| 320.1365916124213 | 1.04826923626e-31 | 320.1365916124213 | 5.02213811539e-11 | 320.1365916124213 | 3.71309150927e-33 |
| 319.5568811248402 | 6.03181694578e-32 | 319.5568811248402 | 3.52121907389e-11 | 319.5568811248402 | 2.11154774185e-33 |
| 318.97926634768737 | 3.46386357098e-32 | 318.97926634768737 | 2.46396633171e-11 | 318.97926634768737 | 1.19840460981e-33 |
| 318.40373593716834 | 1.98522948467e-32 | 318.40373593716834 | 1.720734324e-11 | 318.40373593716834 | 6.7880236954e-34 |
| 317.8302786312109 | 1.13552831491e-32 | 317.8302786312109 | 1.19930654147e-11 | 317.8302786312109 | 3.83725394673e-34 |
| 317.2588832487309 | 6.48220157896e-33 | 317.2588832487309 | 8.34226363498e-12 | 317.2588832487309 | 2.16488607146e-34 |
| 316.6895386889053 | 3.69304338386e-33 | 316.6895386889053 | 5.79128509224e-12 | 316.6895386889053 | 1.21895272929e-34 |
| 316.1222339304531 | 2.09982740656e-33 | 316.1222339304531 | 4.01239152898e-12 | 316.1222339304531 | 6.8497700926e-35 |
| 315.55695803092453 | 1.19157148828e-33 | 315.55695803092453 | 2.77439945063e-12 | 315.55695803092453 | 3.84151416442e-35 |
| 314.99370012599746 | 6.74829281996e-34 | 314.99370012599746 | 1.9145733039e-12 | 314.99370012599746 | 2.15013731231e-35 |
| 314.432449428781 | 3.81421393286e-34 | 314.432449428781 | 1.31859775423e-12 | 314.432449428781 | 1.20106708585e-35 |
| 313.8731952291274 | 2.15156014221e-34 | 313.8731952291274 | 9.06337579368e-13 | 313.8731952291274 | 6.69584896032e-36 |
| 313.31592689295036 | 1.21126521517e-34 | 313.31592689295036 | 6.21734497972e-13 | 313.31592689295036 | 3.7254723509e-36 |
| 312.76063386155124 | 6.80553565251e-35 | 312.76063386155124 | 4.25654535738e-13 | 312.76063386155124 | 2.06868494345e-36 |
| 312.2073056509522 | 3.81612590167e-35 | 312.2073056509522 | 2.9083514543e-13 | 312.2073056509522 | 1.14642243251e-36 |
| 311.65593185123623 | 2.13560234232e-35 | 311.65593185123623 | 1.98323340534e-13 | 311.65593185123623 | 6.34062852939e-37 |
| 311.1065021258944 | 1.19276639689e-35 | 311.1065021258944 | 1.34970262205e-13 | 311.1065021258944 | 3.49991307678e-37 |
| 310.5590062111801 | 6.64856188355e-36 | 310.5590062111801 | 9.1672626431e-14 | 310.5590062111801 | 1.92805544793e-37 |
| 310.01343391546965 | 3.69859985551e-36 | 310.01343391546965 | 6.21410484172e-14 | 310.01343391546965 | 1.06003228684e-37 |
| 309.4697751186301 | 2.05345076451e-36 | 309.4697751186301 | 4.20392343272e-14 | 309.4697751186301 | 5.81642296269e-38 |
| 308.9280197713932 | 1.13780687378e-36 | 308.9280197713932 | 2.83836561911e-14 | 308.9280197713932 | 3.1851521318e-38 |
| 308.3881578947368 | 6.29202044258e-37 | 308.3881578947368 | 1.91257817768e-14 | 308.3881578947368 | 1.74077120122e-38 |
| 307.8501795792714 | 3.47255401449e-37 | 307.8501795792714 | 1.28619664673e-14 | 307.8501795792714 | 9.49490271258e-39 |
| 307.31407498463426 | 1.91269273182e-37 | 307.31407498463426 | 8.63242606989e-15 | 307.31407498463426 | 5.16864444962e-39 |
| 306.77983433888943 | 1.05142601485e-37 | 306.77983433888943 | 5.78223422387e-15 | 306.77983433888943 | 2.80801943977e-39 |
| 306.2474479379338 | 5.76832261662e-38 | 306.2474479379338 | 3.86541216795e-15 | 306.2474479379338 | 1.52251252826e-39 |
| 305.7169061449098 | 3.15833125955e-38 | 305.7169061449098 | 2.57889260053e-15 | 305.7169061449098 | 8.23870500698e-40 |
| 305.1881993896236 | 1.7258503241e-38 | 305.1881993896236 | 1.71714915127e-15 | 305.1881993896236 | 4.44932719179e-40 |
| 304.6613181679699 | 9.412086094e-39 | 304.6613181679699 | 1.14109052601e-15 | 304.6613181679699 | 2.39809866701e-40 |
| 304.1362530413625 | 5.1227830834e-39 | 304.1362530413625 | 7.56779738605e-16 | 304.1362530413625 | 1.28996243843e-40 |
| 303.61299463617036 | 2.78268056829e-39 | 303.61299463617036 | 5.00905971131e-16 | 303.61299463617036 | 6.92507361497e-41 |
| 303.09153364316023 | 1.50854426064e-39 | 303.09153364316023 | 3.30887405878e-16 | 303.09153364316023 | 3.71030009307e-41 |
| 302.571860816944 | 8.16187748461e-40 | 302.571860816944 | 2.18143150993e-16 | 302.571860816944 | 1.98395123481e-41 |
| 302.0539669754329 | 4.40716589874e-40 | 302.0539669754329 | 1.4352919276e-16 | 302.0539669754329 | 1.0587422928e-41 |
| 301.5378429992964 | 2.37501326408e-40 | 301.5378429992964 | 9.4248884349e-17 | 301.5378429992964 | 5.63880198116e-42 |
| 301.02347983142687 | 1.27735023696e-40 | 301.02347983142687 | 6.17660045604e-17 | 301.02347983142687 | 2.99723460682e-42 |
| 300.5108684764098 | 6.85632284862e-41 | 300.5108684764098 | 4.03980233177e-17 | 300.5108684764098 | 1.58998104237e-42 |

| **GON1-Al6** | |
| --- | --- |
| Wavelength (nm) | Abs |
| 2000.0 | 14.7000786352 |
| 1977.5873434410018 | 16.7494637149 |
| 1955.671447196871 | 19.0516860696 |
| 1934.2359767891683 | 21.6329484329 |
| 1913.265306122449 | 24.5214608022 |
| 1892.7444794952683 | 27.7474987044 |
| 1872.6591760299625 | 31.3434494931 |
| 1852.9956763434218 | 35.3438445343 |
| 1833.7408312958437 | 39.785375047 |
| 1814.8820326678765 | 44.7068893025 |
| 1796.4071856287424 | 50.1493688469 |
| 1778.3046828689983 | 56.155881407 |
| 1760.5633802816901 | 62.7715081763 |
| 1743.1725740848342 | 70.0432432486 |
| 1726.1219792865363 | 78.0198630917 |
| 1709.4017094017095 | 86.7517641205 |
| 1693.002257336343 | 96.2907666503 |
| 1676.9144773616547 | 106.689883786 |
| 1661.1295681063123 | 118.003054133 |
| 1645.6390565002741 | 130.284837598 |
| 1630.4347826086955 | 143.590073987 |
| 1615.5088852988692 | 157.973504594 |
| 1600.8537886872998 | 173.489357524 |
| 1586.4621893178212 | 190.190898049 |
| 1572.3270440251572 | 208.129945941 |
| 1558.4415584415583 | 227.356362349 |
| 1544.799176107106 | 247.917509467 |
| 1531.3935681470139 | 269.857686898 |
| 1518.2186234817814 | 293.217549314 |
| 1505.2684395383842 | 318.033510624 |
| 1492.5373134328358 | 344.337140546 |
| 1480.0197335964478 | 372.154559997 |
| 1467.7103718199608 | 401.505842288 |
| 1455.604075691412 | 432.404427531 |
| 1443.6958614051973 | 464.856558032 |
| 1431.9809069212408 | 498.860742718 |
| 1420.4545454545455 | 534.407258778 |
| 1409.1122592766555 | 571.477698726 |
| 1397.9496738117427 | 610.044570978 |
| 1386.9625520110958 | 650.070961795 |
| 1376.1467889908256 | 691.510266044 |
| 1365.4984069185252 | 734.305993653 |
| 1355.0135501355014 | 778.391658009 |
| 1344.688480502017 | 823.690751646 |
| 1334.5195729537365 | 870.116813659 |
| 1324.5033112582782 | 917.573592163 |
| 1314.6362839614374 | 965.95530393 |
| 1304.9151805132665 | 1015.14699207 |
| 1295.3367875647668 | 1065.02498119 |
| 1285.8979854264894 | 1115.45742827 |
| 1276.5957446808509 | 1166.30496567 |
| 1267.427122940431 | 1217.42143186 |
| 1258.3892617449665 | 1268.65468339 |
| 1249.4793835901708 | 1319.84748095 |
| 1240.6947890818858 | 1370.83844058 |
| 1232.0328542094455 | 1421.46304045 |
| 1223.4910277324632 | 1471.55467217 |
| 1215.0668286755772 | 1520.94572532 |
| 1206.7578439259853 | 1569.46869269 |
| 1198.5617259288852 | 1616.95728375 |
| 1190.4761904761904 | 1663.24753343 |
| 1182.4990145841543 | 1708.17889332 |
| 1174.6280344557556 | 1751.59529265 |
| 1166.8611435239206 | 1793.3461569 |
| 1159.19629057187 | 1833.28737245 |
| 1151.6314779270633 | 1871.28218676 |
| 1144.1647597254005 | 1907.20203427 |
| 1136.794240242516 | 1940.92727989 |
| 1129.5180722891566 | 1972.3478729 |
| 1122.334455667789 | 2001.3639058 |
| 1115.2416356877322 | 2027.88607419 |
| 1108.2379017362393 | 2051.83603521 |
| 1101.3215859030836 | 2073.14666383 |
| 1094.4910616563297 | 2091.76220792 |
| 1087.7447425670775 | 2107.63834425 |
| 1081.081081081081 | 2120.7421396 |
| 1074.4985673352435 | 2131.05192174 |
| 1067.995728017088 | 2138.55706695 |
| 1061.5711252653928 | 2143.2577113 |
| 1055.2233556102708 | 2145.1643938 |
| 1048.951048951049 | 2144.29764045 |
| 1042.752867570386 | 2140.68749822 |
| 1036.6275051831374 | 2134.37302853 |
| 1030.5736860185502 | 2125.40176953 |
| 1024.5901639344263 | 2113.8291766 |
| 1018.6757215619693 | 2099.71804966 |
| 1012.829169480081 | 2083.13795576 |
| 1007.0493454179255 | 2064.16465427 |
| 1001.3351134846461 | 2042.87953157 |
| 995.6853634251576 | 2019.36905073 |
| 990.0990099009902 | 1993.72422109 |
| 984.5749917952082 | 1966.0400912 |
| 979.1122715404699 | 1936.41526785 |
| 973.7098344693281 | 1904.95146256 |
| 968.3666881859263 | 1871.75306616 |
| 963.0818619582664 | 1836.9267509 |
| 957.8544061302682 | 1800.58109895 |
| 952.6833915528738 | 1762.8262552 |
| 947.5679090334806 | 1723.77360179 |
| 942.5070688030161 | 1683.53545137 |
| 937.4999999999999 | 1642.22475569 |
| 932.5458501709667 | 1599.95482598 |
| 927.643784786642 | 1556.83906165 |
| 922.7929867733004 | 1512.99068391 |
| 917.9926560587514 | 1468.522471 |
| 913.2420091324201 | 1423.54649254 |
| 908.5402786190186 | 1378.1738404 |
| 903.8867128653209 | 1332.51435448 |
| 899.2805755395683 | 1286.67634222 |
| 894.7211452430658 | 1240.76629124 |
| 890.2077151335311 | 1194.88857545 |
| 885.7395925597874 | 1149.14515515 |
| 881.316098707403 | 1103.63527279 |
| 876.9365682548962 | 1058.45514627 |
| 872.6003490401396 | 1013.6976623 |
| 868.3068017366135 | 969.452072886 |
| 864.0552995391705 | 925.80369816 |
| 859.8452278589854 | 882.833639155 |
| 855.6759840273816 | 840.618504323 |
| 851.5469770082316 | 799.230153511 |
| 847.457627118644 | 758.735463163 |
| 843.4073657576608 | 719.196116306 |
| 839.3956351426972 | 680.668420657 |
| 835.421888053467 | 643.203157833 |
| 831.4855875831485 | 606.845466244 |
| 827.5862068965516 | 571.634759767 |
| 823.7232289950576 | 537.604683788 |
| 819.8961464881114 | 504.78310962 |
| 816.1044613710554 | 473.192167735 |
| 812.3476848090983 | 442.848319666 |
| 808.6253369272237 | 413.762467837 |
| 804.9369466058491 | 385.940102043 |
| 801.2820512820513 | 359.381480748 |
| 797.6601967561818 | 334.081844926 |
| 794.0709370037056 | 310.031661694 |
| 790.5138339920949 | 287.216894651 |
| 786.9884575026232 | 265.61929753 |
| 783.4943849569078 | 245.216727515 |
| 780.0312012480499 | 225.98347444 |
| 776.598498576236 | 207.890601975 |
| 773.1958762886597 | 190.906296901 |
| 769.8229407236336 | 174.996222598 |
| 766.4793050587633 | 160.123873011 |
| 763.1645891630628 | 146.250923494 |
| 759.8784194528876 | 133.337575163 |
| 756.6204287515762 | 121.342889643 |
| 753.390256152687 | 110.2251114 |
| 750.1875468867216 | 99.9419751423 |
| 747.011952191235 | 90.4509961533 |
| 743.86312918423 | 81.7097417476 |
| 740.7407407407408 | 73.6760824055 |
| 737.6444553725104 | 66.3084215034 |
| 734.5739471106758 | 59.5659028988 |
| 731.528895391368 | 53.4085959653 |
| 728.5089849441475 | 47.7976579836 |
| 725.5139056831922 | 42.6954740891 |
| 722.543352601156 | 38.0657752369 |
| 719.5970256656271 | 33.8737348823 |
| 716.6746297181079 | 30.0860452786 |
| 713.7758743754462 | 26.6709744658 |
| 710.9004739336492 | 23.5984051681 |
| 708.0481472740146 | 20.8398569232 |
| 705.2186177715091 | 18.3684928535 |
| 702.4116132053383 | 16.1591125329 |
| 699.6268656716418 | 14.1881324359 |
| 696.8641114982578 | 12.4335554512 |
| 694.1230911614992 | 10.8749309234 |
| 691.4035492048858 | 9.49330664637 |
| 688.7052341597796 | 8.27117417619 |
| 686.027898467871 | 7.19240876019 |
| 683.371298405467 | 6.24220510007 |
| 680.7351940095303 | 5.40701007645 |
| 678.1193490054249 | 4.67445346838 |
| 675.5235307363206 | 4.03327760248 |
| 672.9475100942127 | 3.47326676659 |
| 670.3910614525139 | 2.98517712326 |
| 667.8539626001781 | 2.5606677605 |
| 665.335994677312 | 2.19223342284 |
| 662.8369421122403 | 1.87313937585 |
| 660.3565925599823 | 1.59735877215 |
| 657.8947368421053 | 1.35951280848 |
| 655.4511688879178 | 1.154813891 |
| 653.0256856769699 | 0.979011960231 |
| 650.6180871828237 | 0.828344068657 |
| 648.2281763180639 | 0.699487252036 |
| 645.8557588805166 | 0.58951469045 |
| 643.5006435006435 | 0.495855116597 |
| 641.1626415900834 | 0.416255396444 |
| 638.8415672913118 | 0.348746180788 |
| 636.5372374283895 | 0.291610505044 |
| 634.2494714587738 | 0.243355198282 |
| 631.9780914261638 | 0.20268495058 |
| 629.7229219143577 | 0.168478879789 |
| 627.4837900020916 | 0.139769434243 |
| 625.2605252188412 | 0.115723466342 |
| 623.0529595015576 | 0.0956253128952 |
| 620.8609271523178 | 0.0788617211019 |
| 618.6842647968654 | 0.0649084638408 |
| 616.5228113440197 | 0.0533184940028 |
| 614.3764079459348 | 0.0437114947595 |
| 612.2448979591836 | 0.0357646905421 |
| 610.1281269066504 | 0.0292047918931 |
| 608.0259424402108 | 0.0238009560275 |
| 605.9381943041809 | 0.0193586537094 |
| 603.864734299517 | 0.0157143417698 |
| 601.8054162487462 | 0.0127308491287 |
| 599.7600959616153 | 0.0102933924425 |
| 597.7286312014345 | 0.00830614540099 |
| 595.7108816521048 | 0.00668929318253 |
| 593.7067088858104 | 0.00537651061152 |
| 591.7159763313609 | 0.00431280911691 |
| 589.7385492431688 | 0.00345270365552 |
| 587.7742946708463 | 0.0027586563398 |
| 585.8230814294083 | 0.00219975860187 |
| 583.8847800700661 | 0.0017506183498 |
| 581.9592628516003 | 0.0013904227482 |
| 580.046403712297 | 0.0011021510066 |
| 578.1460782424359 | 0.000871914911704 |
| 576.2581636573184 | 0.000688407822753 |
| 574.3825387708214 | 0.000542445489866 |
| 572.5190839694656 | 0.000426584383427 |
| 570.6676811869887 | 0.000334805266138 |
| 568.8282138794084 | 0.000262251525609 |
| 567.000567000567 | 0.000205013340477 |
| 565.1846269781461 | 0.000159950101671 |
| 563.3802816901408 | 0.000124544675497 |
| 561.5874204417821 | 9.67840979354e-05 |
| 559.8059339428997 | 7.50621494476e-05 |
| 558.0357142857143 | 5.80999943446e-05 |
| 556.2766549230483 | 4.48816944528e-05 |
| 554.52865064695 | 3.46019377358e-05 |
| 552.791597567717 | 2.66237715745e-05 |
| 551.0653930933137 | 2.04445089233e-05 |
| 549.3499359091741 | 1.5668293584e-05 |
| 547.645125958379 | 1.19840771979e-05 |
| 545.950864422202 | 9.14798291871e-06 |
| 544.2670537010159 | 6.96921580362e-06 |
| 542.5935973955508 | 5.29883350683e-06 |
| 540.9304002884962 | 4.02081787968e-06 |
| 539.2773683264425 | 3.04499282776e-06 |
| 537.6344086021505 | 2.30141990714e-06 |
| 536.0014293371448 | 1.73597375326e-06 |
| 534.3783398646241 | 1.30685715832e-06 |
| 532.7650506126798 | 9.81862661714e-07 |
| 531.1614730878186 | 7.36225758043e-07 |
| 529.5675198587819 | 5.50945819615e-07 |
| 527.9831045406547 | 4.11475878548e-07 |
| 526.4081417792595 | 3.06702601148e-07 |
| 524.8425472358292 | 2.28154012746e-07 |
| 523.2862375719518 | 1.69385536424e-07 |
| 521.7391304347826 | 1.25505304847e-07 |
| 520.2011444425177 | 9.28079919659e-08 |
| 518.6721991701245 | 6.84930004628e-08 |
| 517.1522151353215 | 5.04480656049e-08 |
| 515.6411137848057 | 3.70834682476e-08 |
| 514.1388174807198 | 2.72053089315e-08 |
| 512.6452494873547 | 1.99188590998e-08 |
| 511.1603339580848 | 1.45550173938e-08 |
| 509.683995922528 | 1.06144735962e-08 |
| 508.2161612739285 | 7.7254119727e-09 |
| 506.7567567567567 | 5.61154284131e-09 |
| 505.3057099545225 | 4.06799450423e-09 |
| 503.8629492777964 | 2.94317350605e-09 |
| 502.4284039524367 | 2.12514613727e-09 |
| 501.00200400801606 | 1.53143706925e-09 |
| 499.5836802664446 | 1.10140469957e-09 |
| 498.1733643307871 | 7.90555026914e-10 |
| 496.7709885742673 | 5.66310596554e-10 |
| 495.3764861294584 | 4.04869135835e-10 |
| 493.98979087765514 | 2.88876389815e-10 |
| 492.61083743842363 | 2.05705931799e-10 |
| 491.2395611593253 | 1.46190438481e-10 |
| 489.8758981058131 | 1.03688001569e-10 |
| 488.5197850512946 | 7.33965113951e-11 |
| 487.17115946735953 | 5.18513068343e-11 |
| 485.82995951416996 | 3.65579106198e-11 |
| 484.49612403100775 | 2.57241121986e-11 |
| 483.16959252697694 | 1.80649510418e-11 |
| 481.8503051718599 | 1.26610739448e-11 |
| 480.5382027871216 | 8.85608265801e-12 |
| 479.23322683706067 | 6.18230084255e-12 |
| 477.9353194201051 | 4.30720917426e-12 |
| 476.64442326024783 | 2.99487806036e-12 |
| 475.3604816986214 | 2.07825902882e-12 |
| 474.08343868520853 | 1.43932064923e-12 |
| 472.8132387706856 | 9.9483895526e-13 |
| 471.5498270983967 | 6.86254735374e-13 |
| 470.29314939645707 | 4.72449364104e-13 |
| 469.04315196998124 | 3.24610470976e-13 |
| 467.7997816934352 | 2.22590754037e-13 |
| 466.5629860031104 | 1.52331267253e-13 |
| 465.33271288971605 | 1.04041912416e-13 |
| 464.10891089108907 | 7.09193805661e-14 |
| 462.8915290850177 | 4.82457292662e-14 |
| 461.68051708217905 | 3.27559463099e-14 |
| 460.47582501918646 | 2.21951844252e-14 |
| 459.2774035517452 | 1.50094471763e-14 |
| 458.0852038479157 | 1.01299654862e-14 |
| 456.89917758148033 | 6.82320726726e-15 |
| 455.7192769254139 | 4.58676500836e-15 |
| 454.54545454545456 | 3.07724266408e-15 |
| 453.3776635937736 | 2.06041321274e-15 |
| 452.2158577027434 | 1.37684240564e-15 |
| 451.05999097880016 | 9.18229975323e-16 |
| 449.9100179964007 | 6.11161549609e-16 |
| 448.7658937920718 | 4.05973704654e-16 |
| 447.6275738585497 | 2.69139294493e-16 |
| 446.49501413900873 | 1.78071180336e-16 |
| 445.36817102137763 | 1.17583798974e-16 |
| 444.247001332741 | 7.74887507796e-17 |
| 443.13146233382565 | 5.09644278447e-17 |
| 442.02151171357 | 3.34528379886e-17 |
| 440.9171075837742 | 2.19147288787e-17 |
| 439.8182084738308 | 1.43277025576e-17 |
| 438.72477332553376 | 9.34876690684e-18 |
| 437.636761487965 | 6.08792685173e-18 |
| 436.5541327124563 | 3.95659719347e-18 |
| 435.4768471476266 | 2.56632450121e-18 |
| 434.4048653344918 | 1.66126387148e-18 |
| 433.3381482016467 | 1.07325516807e-18 |
| 432.2766570605187 | 6.91997737225e-19 |
| 431.22035360069 | 4.4529075428e-19 |
| 430.1691998852882 | 2.85969686055e-19 |
| 429.1231583464454 | 1.83287832923e-19 |
| 428.0821917808219 | 1.17242370593e-19 |
| 427.0462633451957 | 7.48467354032e-20 |
| 426.01533655211585 | 4.76868296358e-20 |
| 424.9893752656184 | 3.03222451206e-20 |
| 423.96834369700395 | 1.92425035025e-20 |
| 422.9522064006767 | 1.21870645178e-20 |
| 421.9409282700422 | 7.7032494573e-21 |
| 420.93447453346425 | 4.85943893859e-21 |
| 419.9328107502799 | 3.0593954197e-21 |
| 418.93590280687056 | 1.92230550778e-21 |
| 417.94371691278906 | 1.20544256769e-21 |
| 416.9562195969423 | 7.54410895465e-22 |
| 415.97337770382694 | 4.7120153372e-22 |
| 414.99515838981876 | 2.93726245201e-22 |
| 414.0215291195142 | 1.82732639119e-22 |
| 413.0524576621231 | 1.13455829194e-22 |
| 412.08791208791206 | 7.03031551766e-23 |
| 411.1278607646978 | 4.3477056446e-23 |
| 410.17227235438884 | 2.68338361863e-23 |
| 409.22111580957574 | 1.65288527082e-23 |
| 408.2743603701687 | 1.01610823072e-23 |
| 407.33197556008145 | 6.23411117767e-24 |
| 406.39393118396094 | 3.81721332413e-24 |
| 405.46019732396263 | 2.33268250989e-24 |
| 404.53074433656957 | 1.4226633682e-24 |
| 403.6055428494551 | 8.65936366693e-25 |
| 402.68456375838923 | 5.2602589374e-25 |
| 401.76777822418643 | 3.18908122403e-25 |
| 400.85515766969536 | 1.9295736156e-25 |
| 399.9466737768297 | 1.16518379863e-25 |
| 399.0422984836393 | 7.0220648553e-26 |
| 398.14200398142003 | 4.22350048965e-26 |
| 397.24576271186436 | 2.5352312246e-26 |
| 396.3535473642489 | 1.51879764455e-26 |
| 395.46533087266016 | 9.08070502335e-27 |
| 394.5810864132579 | 5.41846827906e-27 |
| 393.7007874015748 | 3.22679084185e-27 |
| 392.82440748985204 | 1.91779600485e-27 |
| 391.9519205644107 | 1.13755215342e-27 |
| 391.08330074305826 | 6.73406862219e-28 |
| 390.2185223725286 | 3.97851565976e-28 |
| 389.3575600259571 | 2.34585907803e-28 |
| 388.5003885003885 | 1.3804480901e-28 |
| 387.6469828143171 | 8.10728713071e-29 |
| 386.7973182052604 | 4.75191148065e-29 |
| 385.95137012736393 | 2.77970330057e-29 |
| 385.1091142490372 | 1.62280320531e-29 |
| 384.2705264506212 | 9.4551970509e-30 |
| 383.4355828220859 | 5.49809987595e-30 |
| 382.6042596607575 | 3.19074412744e-30 |
| 381.77653346907607 | 1.84802841944e-30 |
| 380.95238095238096 | 1.06822472496e-30 |
| 380.1317790167258 | 6.16245724666e-31 |
| 379.31470476672143 | 3.54799113384e-31 |
| 378.5011355034065 | 2.03867710493e-31 |
| 377.69104872214524 | 1.16910004867e-31 |
| 376.88442211055275 | 6.69101840765e-32 |
| 376.081233546446 | 3.82181872464e-32 |
| 375.28146109582184 | 2.17863891229e-32 |
| 374.48508301086 | 1.23947491478e-32 |
| 373.69207772795215 | 7.03764798702e-33 |
| 372.9024238657551 | 3.98799542977e-33 |
| 372.11610022326965 | 2.25537663371e-33 |
| 371.33308577794276 | 1.27297775519e-33 |
| 370.55335968379444 | 7.17067213253e-34 |
| 369.7769012695673 | 4.03121748973e-34 |
| 369.0036900369003 | 2.26177764191e-34 |
| 368.23370565852457 | 1.26648746823e-34 |
| 367.4669279764821 | 7.07765191874e-35 |
| 366.7033370003667 | 3.94743344478e-35 |
| 365.9429129055867 | 2.19724126036e-35 |
| 365.1856360316494 | 1.22061299175e-35 |
| 364.4314868804664 | 6.7673017765e-36 |
| 363.68044611468054 | 3.74447048784e-36 |
| 362.93249455601256 | 2.06777173034e-36 |
| 362.1876131836291 | 1.13959907511e-36 |
| 361.4457831325301 | 6.26814299818e-37 |
| 360.7069856919562 | 3.44082841692e-37 |
| 359.97120230381563 | 1.88505683969e-37 |
| 359.2384145611304 | 1.03067845215e-37 |
| 358.50860420650093 | 5.62418057713e-38 |
| 357.7817531305903 | 3.06289866772e-38 |
| 357.057843370626 | 1.66472811655e-38 |
| 356.33685710892024 | 9.0300740844e-39 |
| 355.6187766714082 | 4.88851131885e-39 |
| 354.9035845262037 | 2.64118760101e-39 |
| 354.1912632821723 | 1.42416132439e-39 |
| 353.48179568752204 | 7.66401662766e-40 |
| 352.77516462841015 | 4.11614819157e-40 |
| 352.07135312756714 | 2.20629143386e-40 |
| 351.3703443429374 | 1.18024472631e-40 |
| 350.6721215663355 | 6.30113190883e-41 |
| 349.9766682221185 | 3.35739459582e-41 |
| 349.2839678658749 | 1.78535060798e-41 |
| 348.59400418312805 | 9.47505975405e-42 |
| 347.90676098805517 | 5.01854372337e-42 |
| 347.2222222222222 | 2.6528383059e-42 |
| 346.54037195333257 | 1.39952661093e-42 |
| 345.8611943739912 | 7.36866570617e-43 |
| 345.1846738004832 | 3.87198673724e-43 |
| 344.5107946715664 | 2.03056165493e-43 |
| 343.8395415472779 | 1.06276150068e-43 |
| 343.17089910775564 | 5.5512752175e-44 |
| 342.50485215207215 | 2.89392330795e-44 |
| 341.84138559708293 | 1.50563114305e-44 |
| 341.1804844762879 | 7.8178525286e-45 |
| 340.522133938706 | 4.05129316665e-45 |
| 339.86631924776253 | 2.09525642976e-45 |
| 339.2130257801899 | 1.08147878789e-45 |
| 338.56223902494077 | 5.57103827406e-46 |
| 337.91394458211306 | 2.86412251741e-46 |
| 337.2681281618887 | 1.46954995625e-46 |
| 336.6247755834829 | 7.52513732411e-47 |
| 335.9838727741068 | 3.84575681321e-47 |
| 335.3454057679409 | 1.96149190233e-47 |
| 334.709360705121 | 9.98455086969e-48 |
| 334.07572383073494 | 5.072334215e-48 |
| 333.44448149383123 | 2.57172485231e-48 |
| 332.81562014643885 | 1.30130307228e-48 |
| 332.1891263425977 | 6.5715788236e-49 |
| 331.5649867374005 | 3.3120608014e-49 |
| 330.9431880860452 | 1.66595855522e-49 |
| 330.323717242898 | 8.36310209019e-50 |
| 329.70656116056705 | 4.18994108715e-50 |
| 329.0917068889864 | 2.09500829964e-50 |
| 328.47914157451 | 1.04544428298e-50 |
| 327.86885245901635 | 5.20658963178e-51 |
| 327.26082687902255 | 2.58787395257e-51 |
| 326.6550522648083 | 1.28371968976e-51 |
| 326.05151613955 | 6.35527867128e-52 |
| 325.4502061184639 | 3.1400481501e-52 |
| 324.8511099079588 | 1.54837207341e-52 |
| 324.25421530479895 | 7.61994198423e-53 |
| 323.65951019527455 | 3.7425301936e-53 |
| 323.0669825543829 | 1.83449390752e-53 |
| 322.4766204450177 | 8.97438192015e-54 |
| 321.88841201716735 | 4.38157383676e-54 |
| 321.3023455071222 | 2.13497618311e-54 |
| 320.71840923669015 | 1.03822921985e-54 |
| 320.1365916124213 | 5.03884237914e-55 |
| 319.5568811248402 | 2.44065060713e-55 |
| 318.97926634768737 | 1.17982546055e-55 |
| 318.40373593716834 | 5.69203063414e-56 |
| 317.8302786312109 | 2.74065271266e-56 |
| 317.2588832487309 | 1.31697671753e-56 |
| 316.6895386889053 | 6.31596189175e-57 |
| 316.1222339304531 | 3.02300049053e-57 |
| 315.55695803092453 | 1.44402345451e-57 |
| 314.99370012599746 | 6.88410665081e-58 |
| 314.432449428781 | 3.27535400125e-58 |
| 313.8731952291274 | 1.55527150143e-58 |
| 313.31592689295036 | 7.37040742518e-59 |
| 312.76063386155124 | 3.48589330468e-59 |
| 312.2073056509522 | 1.64540948965e-59 |
| 311.65593185123623 | 7.75124060889e-60 |
| 311.1065021258944 | 3.64422977035e-60 |
| 310.5590062111801 | 1.70992711069e-60 |
| 310.01343391546965 | 8.00731212157e-61 |
| 309.4697751186301 | 3.74225372903e-61 |
| 308.9280197713932 | 1.7454885937e-61 |
| 308.3881578947368 | 8.12527583966e-62 |
| 307.8501795792714 | 3.77482241095e-62 |
| 307.31407498463426 | 1.75021841523e-62 |
| 306.77983433888943 | 8.09888600742e-63 |
| 306.2474479379338 | 3.74020698101e-63 |
| 305.7169061449098 | 1.72386518851e-63 |
| 305.1881993896236 | 7.92954514172e-64 |
| 304.6613181679699 | 3.64024458875e-64 |
| 304.1362530413625 | 1.66782380038e-64 |
| 303.61299463617036 | 7.62618053514e-65 |
| 303.09153364316023 | 3.48017682127e-65 |
| 302.571860816944 | 1.58501308265e-65 |
| 302.0539669754329 | 7.20446449184e-66 |
| 301.5378429992964 | 3.26819432997e-66 |
| 301.02347983142687 | 1.47962394902e-66 |
| 300.5108684764098 | 6.68547309018e-67 |
