# Supplementary material for: Data of electronic, reactivity, optoelectronic, linear and non-linear optical parameters of doping graphene oxide nanosheet with aluminum atom
Source: Data Brief. 2022 Jan 19;41:107840. doi: 10.1016/j.dib.2022.107840 (PMC8801356; doi:10.1016/j.dib.2022.107840)
Supplement: Supplementary file 1 [file mmc1.zip › supplementary file/DATA OF THE UV-VIS SPECTRA/DATA OF THE UV-VIS SPECTRA OF GON1 AND ITS DERIVATIVES (GON1-Alx) B3LYP-D3.docx]

**Data of the UV-Vis spectra of GON1 isomer and its aluminum-doped derivatives (GON1-Alx), computed at the B3LYP-D3/6-31+g(d,p) level of theory**

| **GON1** | | **GON1-Al1** | | **GON1-Al2** | |
| --- | --- | --- | --- | --- | --- |
| Wavelength (nm) | Abs | Wavelength (nm) | Abs | Wavelength (nm) | Abs |
| 2000.0 | 2.62056328875e-06 | 2000.0 | 1.35508706496e-11 | 2000.0 | 2.52915979441 |
| 1977.5873434410018 | 3.4626889006e-06 | 1977.5873434410018 | 1.95389373677e-11 | 1977.5873434410018 | 2.91704026379 |
| 1955.671447196871 | 4.56635435154e-06 | 1955.671447196871 | 2.81171951241e-11 | 1955.671447196871 | 3.35773184015 |
| 1934.2359767891683 | 6.00984204914e-06 | 1934.2359767891683 | 4.03813061013e-11 | 1934.2359767891683 | 3.85733216902 |
| 1913.265306122449 | 7.89393996272e-06 | 1913.265306122449 | 5.78796693825e-11 | 1913.265306122449 | 4.4224764626 |
| 1892.7444794952683 | 1.03481304072e-05 | 1892.7444794952683 | 8.27959387563e-11 | 1892.7444794952683 | 5.06036099691 |
| 1872.6591760299625 | 1.35383980863e-05 | 1872.6591760299625 | 1.1820322901e-10 | 1872.6591760299625 | 5.77876412062 |
| 1852.9956763434218 | 1.767705772e-05 | 1852.9956763434218 | 1.68417400345e-10 | 1852.9956763434218 | 6.58606414556 |
| 1833.7408312958437 | 2.30350942177e-05 | 1833.7408312958437 | 2.39486971062e-10 | 1833.7408312958437 | 7.49125344445 |
| 1814.8820326678765 | 2.99576207734e-05 | 1814.8820326678765 | 3.39871020908e-10 | 1814.8820326678765 | 8.5039480413 |
| 1796.4071856287424 | 3.88831962873e-05 | 1796.4071856287424 | 4.81375179802e-10 | 1796.4071856287424 | 9.63439194721 |
| 1778.3046828689983 | 5.03679075927e-05 | 1778.3046828689983 | 6.80441141715e-10 | 1778.3046828689983 | 10.8934554694 |
| 1760.5633802816901 | 6.51153192822e-05 | 1760.5633802816901 | 9.59919354645e-10 | 1760.5633802816901 | 12.2926267055 |
| 1743.1725740848342 | 8.40136304627e-05 | 1743.1725740848342 | 1.3515006069e-09 | 1743.1725740848342 | 13.8439954329 |
| 1726.1219792865363 | 0.000108181660434 | 1726.1219792865363 | 1.89904412016e-09 | 1726.1219792865363 | 15.5602286097 |
| 1709.4017094017095 | 0.000139025621973 | 1709.4017094017095 | 2.66312270571e-09 | 1709.4017094017095 | 17.4545367301 |
| 1693.002257336343 | 0.000178309040659 | 1693.002257336343 | 3.72721643998e-09 | 1693.002257336343 | 19.5406303163 |
| 1676.9144773616547 | 0.000228238651702 | 1676.9144773616547 | 5.20613423333e-09 | 1676.9144773616547 | 21.832665884 |
| 1661.1295681063123 | 0.000291569663593 | 1661.1295681063123 | 7.2574394847e-09 | 1661.1295681063123 | 24.3451807987 |
| 1645.6390565002741 | 0.000371734433541 | 1645.6390565002741 | 1.00969172679e-08 | 1645.6390565002741 | 27.0930165298 |
| 1630.4347826086955 | 0.000472999367515 | 1630.4347826086955 | 1.40194664378e-08 | 1630.4347826086955 | 30.0912299289 |
| 1615.5088852988692 | 0.000600655753755 | 1615.5088852988692 | 1.94272570155e-08 | 1615.5088852988692 | 33.3549922914 |
| 1600.8537886872998 | 0.000761251280458 | 1600.8537886872998 | 2.68675953764e-08 | 1600.8537886872998 | 36.8994761157 |
| 1586.4621893178212 | 0.000962870195113 | 1586.4621893178212 | 3.70837313051e-08 | 1586.4621893178212 | 40.7397296492 |
| 1572.3270440251572 | 0.00121547145543 | 1572.3270440251572 | 5.10828789106e-08 | 1572.3270440251572 | 44.8905395042 |
| 1558.4415584415583 | 0.00153129582219 | 1558.4415584415583 | 7.0227081034e-08 | 1558.4415584415583 | 49.3662818324 |
| 1544.799176107106 | 0.00192535467619 | 1544.799176107106 | 9.63543182312e-08 | 1544.799176107106 | 54.1807627764 |
| 1531.3935681470139 | 0.0024160154295 | 1531.3935681470139 | 1.31939569035e-07 | 1531.3935681470139 | 59.3470491476 |
| 1518.2186234817814 | 0.00302570077085 | 1518.2186234817814 | 1.80308521595e-07 | 1518.2186234817814 | 64.8772905286 |
| 1505.2684395383842 | 0.00378172166231 | 1505.2684395383842 | 2.45920513367e-07 | 1505.2684395383842 | 70.7825342457 |
| 1492.5373134328358 | 0.00471726701468 | 1492.5373134328358 | 3.3474228319e-07 | 1492.5373134328358 | 77.072534911 |
| 1480.0197335964478 | 0.00587257633816 | 1480.0197335964478 | 4.54740572532e-07 | 1480.0197335964478 | 83.7555604813 |
| 1467.7103718199608 | 0.00729632541476 | 1467.7103718199608 | 6.16529905553e-07 | 1467.7103718199608 | 90.8381970176 |
| 1455.604075691412 | 0.00904725919214 | 1455.604075691412 | 8.34222508625e-07 | 1455.604075691412 | 98.3251545603 |
| 1443.6958614051973 | 0.0111961106704 | 1443.6958614051973 | 1.12654092332e-06 | 1443.6958614051973 | 106.21907674 |
| 1431.9809069212408 | 0.013827849557 | 1431.9809069212408 | 1.51827120306e-06 | 1431.9809069212408 | 114.520356922 |
| 1420.4545454545455 | 0.0170443099049 | 1420.4545454545455 | 2.042156636e-06 | 1420.4545454545455 | 123.22696385 |
| 1409.1122592766555 | 0.0209672518228 | 1409.1122592766555 | 2.74135991456e-06 | 1409.1122592766555 | 132.334279851 |
| 1397.9496738117427 | 0.0257419186406 | 1397.9496738117427 | 3.67265707475e-06 | 1397.9496738117427 | 141.83495476 |
| 1386.9625520110958 | 0.0315411576008 | 1386.9625520110958 | 4.91057120762e-06 | 1386.9625520110958 | 151.718778743 |
| 1376.1467889908256 | 0.0385701791895 | 1376.1467889908256 | 6.55271018156e-06 | 1376.1467889908256 | 161.972577188 |
| 1365.4984069185252 | 0.0470720375578 | 1365.4984069185252 | 8.72664320755e-06 | 1365.4984069185252 | 172.580130755 |
| 1355.0135501355014 | 0.0573339220422 | 1355.0135501355014 | 1.15987394565e-05 | 1355.0135501355014 | 183.522123558 |
| 1344.688480502017 | 0.069694357469 | 1344.688480502017 | 1.53855022772e-05 | 1344.688480502017 | 194.77612227 |
| 1334.5195729537365 | 0.0845514185926 | 1334.5195729537365 | 2.03680699463e-05 | 1334.5195729537365 | 206.316588692 |
| 1324.5033112582782 | 0.102372071532 | 1324.5033112582782 | 2.69107244646e-05 | 1324.5033112582782 | 218.114928041 |
| 1314.6362839614374 | 0.123702762238 | 1314.6362839614374 | 3.54844611315e-05 | 1314.6362839614374 | 230.139574843 |
| 1304.9151805132665 | 0.149181378623 | 1304.9151805132665 | 4.66969324246e-05 | 1304.9151805132665 | 242.35611794 |
| 1295.3367875647668 | 0.179550718811 | 1295.3367875647668 | 6.13304008162e-05 | 1295.3367875647668 | 254.727465608 |
| 1285.8979854264894 | 0.215673602661 | 1285.8979854264894 | 8.0389729386e-05 | 1285.8979854264894 | 267.214051345 |
| 1276.5957446808509 | 0.25854976701 | 1276.5957446808509 | 0.000105162921698 | 1276.5957446808509 | 279.774080301 |
| 1267.427122940431 | 0.30933468666 | 1267.427122940431 | 0.000137297311491 | 1267.427122940431 | 292.363815773 |
| 1258.3892617449665 | 0.369360462435 | 1258.3892617449665 | 0.000178895219674 | 1258.3892617449665 | 304.937904596 |
| 1249.4793835901708 | 0.440158914489 | 1249.4793835901708 | 0.000232633766043 | 1249.4793835901708 | 317.449739645 |
| 1240.6947890818858 | 0.523487012763 | 1240.6947890818858 | 0.000301914575572 | 1240.6947890818858 | 329.851857065 |
| 1232.0328542094455 | 0.621354766715 | 1232.0328542094455 | 0.000391050388429 | 1232.0328542094455 | 342.096365255 |
| 1223.4910277324632 | 0.73605568271 | 1223.4910277324632 | 0.000505497109016 | 1223.4910277324632 | 354.13540202 |
| 1215.0668286755772 | 0.870199879135 | 1215.0668286755772 | 0.000652141658614 | 1215.0668286755772 | 365.921615793 |
| 1206.7578439259853 | 1.02674992602 | 1206.7578439259853 | 0.000839658181814 | 1206.7578439259853 | 377.408666295 |
| 1198.5617259288852 | 1.20905944711 | 1198.5617259288852 | 0.0010789477599 | 1198.5617259288852 | 388.551739551 |
| 1190.4761904761904 | 1.42091448765 | 1190.4761904761904 | 0.00138367987425 | 1190.4761904761904 | 399.308071784 |
| 1182.4990145841543 | 1.66657760987 | 1182.4990145841543 | 0.00177095751867 | 1182.4990145841543 | 409.637476383 |
| 1174.6280344557556 | 1.95083463025 | 1174.6280344557556 | 0.00226213217014 | 1174.6280344557556 | 419.502867901 |
| 1166.8611435239206 | 2.27904385802 | 1166.8611435239206 | 0.0028837998927 | 1166.8611435239206 | 428.870776877 |
| 1159.19629057187 | 2.65718763179 | 1159.19629057187 | 0.00366901578064 | 1159.19629057187 | 437.711849207 |
| 1151.6314779270633 | 3.09192588223 | 1151.6314779270633 | 0.00465877086705 | 1151.6314779270633 | 446.001323833 |
| 1144.1647597254005 | 3.59065137222 | 1144.1647597254005 | 0.00590378366843 | 1144.1647597254005 | 453.719482643 |
| 1136.794240242516 | 4.1615461823 | 1136.794240242516 | 0.00746666785248 | 1136.794240242516 | 460.852066697 |
| 1129.5180722891566 | 4.81363891978 | 1129.5180722891566 | 0.00942454826397 | 1129.5180722891566 | 467.390653243 |
| 1122.334455667789 | 5.55686203432 | 1122.334455667789 | 0.0118722098935 | 1122.334455667789 | 473.332988404 |
| 1115.2416356877322 | 6.40210852229 | 1115.2416356877322 | 0.0149258785079 | 1115.2416356877322 | 478.68327093 |
| 1108.2379017362393 | 7.36128719855 | 1108.2379017362393 | 0.0187277477664 | 1108.2379017362393 | 483.452383026 |
| 1101.3215859030836 | 8.44737560763 | 1101.3215859030836 | 0.0234513859231 | 1101.3215859030836 | 487.65806493 |
| 1094.4910616563297 | 9.67446954001 | 1094.4910616563297 | 0.0293081758569 | 1094.4910616563297 | 491.325030679 |
| 1087.7447425670775 | 11.0578280132 | 1087.7447425670775 | 0.0365549653752 | 1087.7447425670775 | 494.485023283 |
| 1081.081081081081 | 12.6139124756 | 1081.081081081081 | 0.0455031307026 | 1081.081081081081 | 497.176808398 |
| 1074.4985673352435 | 14.3604188963 | 1074.4985673352435 | 0.0565292849554 | 1074.4985673352435 | 499.446106417 |
| 1067.995728017088 | 16.3163013154 | 1067.995728017088 | 0.0700878953912 | 1067.995728017088 | 501.345463843 |
| 1061.5711252653928 | 18.5017853551 | 1061.5711252653928 | 0.0867261084281 | 1061.5711252653928 | 502.934065635 |
| 1055.2233556102708 | 20.9383701329 | 1055.2233556102708 | 0.107101119941 | 1055.2233556102708 | 504.277491135 |
| 1048.951048951049 | 23.6488169725 | 1048.951048951049 | 0.132000470207 | 1048.951048951049 | 505.447416973 |
| 1042.752867570386 | 26.657123291 | 1042.752867570386 | 0.162365688046 | 1042.752867570386 | 506.521271177 |
| 1036.6275051831374 | 29.9884800413 | 1036.6275051831374 | 0.199319757105 | 1036.6275051831374 | 507.581843408 |
| 1030.5736860185502 | 33.6692111224 | 1030.5736860185502 | 0.244198928644 | 1030.5736860185502 | 508.716856884 |
| 1024.5901639344263 | 37.7266932309 | 1024.5901639344263 | 0.298589459245 | 1024.5901639344263 | 510.018508124 |
| 1018.6757215619693 | 42.1892547243 | 1018.6757215619693 | 0.364369908262 | 1018.6757215619693 | 511.58298108 |
| 1012.829169480081 | 47.0860521972 | 1012.829169480081 | 0.443759687759 | 1012.829169480081 | 513.509942541 |
| 1007.0493454179255 | 52.4469236425 | 1007.0493454179255 | 0.539374616556 | 1007.0493454179255 | 515.902025915 |
| 1001.3351134846461 | 58.3022172782 | 1001.3351134846461 | 0.654290288747 | 1001.3351134846461 | 518.864310556 |
| 995.6853634251576 | 64.6825953697 | 995.6853634251576 | 0.792114124501 | 995.6853634251576 | 522.50380372 |
| 990.0990099009902 | 71.618812668 | 990.0990099009902 | 0.957067025732 | 990.0990099009902 | 526.928932036 |
| 984.5749917952082 | 79.1414694116 | 984.5749917952082 | 1.15407560962 | 984.5749917952082 | 532.249049015 |
| 979.1122715404699 | 87.2807392066 | 979.1122715404699 | 1.38887603707 | 979.1122715404699 | 538.573964593 |
| 973.7098344693281 | 96.0660725017 | 973.7098344693281 | 1.66813048887 | 973.7098344693281 | 546.013502103 |
| 968.3666881859263 | 105.525876804 | 968.3666881859263 | 1.999557367 | 968.3666881859263 | 554.677087254 |
| 963.0818619582664 | 115.687175246 | 963.0818619582664 | 2.39207630941 | 963.0818619582664 | 564.673372817 |
| 957.8544061302682 | 126.575245578 | 957.8544061302682 | 2.85596910109 | 957.8544061302682 | 576.109901704 |
| 952.6833915528738 | 138.213242181 | 952.6833915528738 | 3.4030575384 | 952.6833915528738 | 589.092810006 |
| 947.5679090334806 | 150.621804153 | 947.5679090334806 | 4.0468992544 | 947.5679090334806 | 603.726570385 |
| 942.5070688030161 | 163.818653051 | 942.5070688030161 | 4.80300243687 | 942.5070688030161 | 620.11377494 |
| 937.4999999999999 | 177.818184341 | 937.4999999999999 | 5.68906026333 | 937.4999999999999 | 638.354955425 |
| 932.5458501709667 | 192.631057051 | 932.5458501709667 | 6.72520573531 | 932.5458501709667 | 658.548437347 |
| 927.643784786642 | 208.26378658 | 927.643784786642 | 7.93428741385 | 927.643784786642 | 680.790223235 |
| 922.7929867733004 | 224.718345967 | 922.7929867733004 | 9.34216633474 | 922.7929867733004 | 705.173899109 |
| 917.9926560587514 | 241.991781266 | 917.9926560587514 | 10.9780341136 | 917.9926560587514 | 731.790557013 |
| 913.2420091324201 | 260.07584693 | 913.2420091324201 | 12.8747519324 | 913.2420091324201 | 760.72872542 |
| 908.5402786190186 | 278.956667289 | 908.5402786190186 | 15.0692097305 | 908.5402786190186 | 792.074298374 |
| 903.8867128653209 | 298.614430306 | 903.8867128653209 | 17.6027044975 | 903.8867128653209 | 825.910453468 |
| 899.2805755395683 | 319.023119805 | 899.2805755395683 | 20.5213360895 | 899.2805755395683 | 862.317548192 |
| 894.7211452430658 | 340.150292254 | 894.7211452430658 | 23.8764184531 | 894.7211452430658 | 901.372983801 |
| 890.2077151335311 | 361.956903991 | 890.2077151335311 | 27.7249035557 | 890.2077151335311 | 943.151025757 |
| 885.7395925597874 | 384.397194481 | 885.7395925597874 | 32.1298146773 | 885.7395925597874 | 987.722569943 |
| 881.316098707403 | 407.418630725 | 881.316098707403 | 37.1606850295 | 881.316098707403 | 1035.15484425 |
| 876.9365682548962 | 430.961917443 | 876.9365682548962 | 42.8939969337 | 876.9365682548962 | 1085.51103594 |
| 872.6003490401396 | 454.96107699 | 872.6003490401396 | 49.4136160208 | 872.6003490401396 | 1138.84983606 |
| 868.3068017366135 | 479.343602186 | 868.3068017366135 | 56.8112141152 | 868.3068017366135 | 1195.22489369 |
| 864.0552995391705 | 504.030684435 | 864.0552995391705 | 65.186673652 | 864.0552995391705 | 1254.68417434 |
| 859.8452278589854 | 528.937518529 | 859.8452278589854 | 74.6484656585 | 859.8452278589854 | 1317.26921866 |
| 855.6759840273816 | 553.973684547 | 855.6759840273816 | 85.3139925219 | 855.6759840273816 | 1383.01429989 |
| 851.5469770082316 | 579.043606158 | 851.5469770082316 | 97.3098859908 | 851.5469770082316 | 1451.94548094 |
| 847.457627118644 | 604.047083562 | 847.457627118644 | 110.772250122 | 847.457627118644 | 1524.07957437 |
| 843.4073657576608 | 628.879898107 | 843.4073657576608 | 125.84683823 | 843.4073657576608 | 1599.42301161 |
| 839.3956351426972 | 653.434484519 | 839.3956351426972 | 142.689152312 | 839.3956351426972 | 1677.97063037 |
| 835.421888053467 | 677.600665512 | 835.421888053467 | 161.464452988 | 835.421888053467 | 1759.7043924 |
| 831.4855875831485 | 701.266442472 | 831.4855875831485 | 182.347667656 | 831.4855875831485 | 1844.59204645 |
| 827.5862068965516 | 724.318834862 | 827.5862068965516 | 205.523184422 | 827.5862068965516 | 1932.58575422 |
| 823.7232289950576 | 746.644760015 | 823.7232289950576 | 231.184519408 | 823.7232289950576 | 2023.62070004 |
| 819.8961464881114 | 768.131944154 | 819.8961464881114 | 259.533845288 | 819.8961464881114 | 2117.6137072 |
| 816.1044613710554 | 788.669854716 | 816.1044613710554 | 290.781369386 | 816.1044613710554 | 2214.4618863 |
| 812.3476848090983 | 808.150643447 | 812.3476848090983 | 325.144550442 | 812.3476848090983 | 2314.04134291 |
| 808.6253369272237 | 826.470089289 | 808.6253369272237 | 362.847144144 | 808.6253369272237 | 2416.20597331 |
| 804.9369466058491 | 843.528529806 | 804.9369466058491 | 404.11806888 | 804.9369466058491 | 2520.78637795 |
| 801.2820512820513 | 859.231769744 | 801.2820512820513 | 449.190084762 | 801.2820512820513 | 2627.58892304 |
| 797.6601967561818 | 873.491955428 | 797.6601967561818 | 498.2982809 | 797.6601967561818 | 2736.39498037 |
| 794.0709370037056 | 886.228403924 | 794.0709370037056 | 551.678368158 | 794.0709370037056 | 2846.96037503 |
| 790.5138339920949 | 897.368376346 | 790.5138339920949 | 609.564777152 | 790.5138339920949 | 2959.01506903 |
| 786.9884575026232 | 906.847785294 | 786.9884575026232 | 672.18856407 | 786.9884575026232 | 3072.26310739 |
| 783.4943849569078 | 914.611827205 | 783.4943849569078 | 739.775129994 | 783.4943849569078 | 3186.38285019 |
| 780.0312012480499 | 920.615531354 | 780.0312012480499 | 812.541762731 | 780.0312012480499 | 3301.02751113 |
| 776.598498576236 | 924.824218322 | 776.598498576236 | 890.695013677 | 776.598498576236 | 3415.8260194 |
| 773.1958762886597 | 927.213862 | 773.1958762886597 | 974.42792593 | 773.1958762886597 | 3530.38421712 |
| 769.8229407236336 | 927.771350503 | 769.8229407236336 | 1063.91713365 | 769.8229407236336 | 3644.28639996 |
| 766.4793050587633 | 926.494642817 | 766.4793050587633 | 1159.31985646 | 766.4793050587633 | 3757.09720319 |
| 763.1645891630628 | 923.392819461 | 763.1645891630628 | 1260.77081656 | 763.1645891630628 | 3868.36382998 |
| 759.8784194528876 | 918.486026969 | 759.8784194528876 | 1368.37910978 | 759.8784194528876 | 3977.61861285 |
| 756.6204287515762 | 911.805317528 | 756.6204287515762 | 1482.22506545 | 756.6204287515762 | 4084.3818934 |
| 753.390256152687 | 903.392386592 | 753.390256152687 | 1602.35713322 | 753.390256152687 | 4188.16519935 |
| 750.1875468867216 | 893.299212776 | 750.1875468867216 | 1728.78883769 | 750.1875468867216 | 4288.47469231 |
| 747.011952191235 | 881.587605697 | 747.011952191235 | 1861.49584449 | 747.011952191235 | 4384.81485408 |
| 743.86312918423 | 868.328668741 | 743.86312918423 | 2000.41318325 | 743.86312918423 | 4476.69237386 |
| 740.7407407407408 | 853.602184892 | 740.7407407407408 | 2145.43267425 | 740.7407407407408 | 4563.62019438 |
| 737.6444553725104 | 837.49593481 | 737.6444553725104 | 2296.40060648 | 737.6444553725104 | 4645.1216703 |
| 734.5739471106758 | 820.104957213 | 734.5739471106758 | 2453.11571462 | 734.5739471106758 | 4720.73478922 |
| 731.528895391368 | 801.530762352 | 731.528895391368 | 2615.3275019 | 731.528895391368 | 4790.01640248 |
| 728.5089849441475 | 781.880509881 | 728.5089849441475 | 2782.73495403 | 728.5089849441475 | 4852.54641129 |
| 725.5139056831922 | 761.266162794 | 725.5139056831922 | 2954.98568712 | 725.5139056831922 | 4907.93185265 |
| 722.543352601156 | 739.803629264 | 722.543352601156 | 3131.67556904 | 722.543352601156 | 4955.81082938 |
| 719.5970256656271 | 717.61190418 | 719.5970256656271 | 3312.34884971 | 719.5970256656271 | 4995.85622961 |
| 716.6746297181079 | 694.812221993 | 716.6746297181079 | 3496.49883055 | 716.6746297181079 | 5027.77918294 |
| 713.7758743754462 | 671.527232071 | 713.7758743754462 | 3683.56909785 | 713.7758743754462 | 5051.33220296 |
| 710.9004739336492 | 647.880207251 | 710.9004739336492 | 3872.95533795 | 710.9004739336492 | 5066.31197024 |
| 708.0481472740146 | 623.994295531 | 708.0481472740146 | 4064.00774509 | 708.0481472740146 | 5072.56171345 |
| 705.2186177715091 | 599.991824033 | 705.2186177715091 | 4256.03402503 | 705.2186177715091 | 5069.97315274 |
| 702.4116132053383 | 575.993663398 | 702.4116132053383 | 4448.30298906 | 702.4116132053383 | 5058.48797443 |
| 699.6268656716418 | 552.118659704 | 699.6268656716418 | 4640.04872482 | 699.6268656716418 | 5038.09881347 |
| 696.8641114982578 | 528.48313986 | 696.8641114982578 | 4830.47532109 | 696.8641114982578 | 5008.84972673 |
| 694.1230911614992 | 505.200495204 | 694.1230911614992 | 5018.76211531 | 694.1230911614992 | 4970.83614754 |
| 691.4035492048858 | 482.380846803 | 691.4035492048858 | 5204.06942362 | 691.4035492048858 | 4924.20431966 |
| 688.7052341597796 | 460.130794651 | 688.7052341597796 | 5385.54470473 | 688.7052341597796 | 4869.15021614 |
| 686.027898467871 | 438.553251693 | 686.027898467871 | 5562.32910114 | 686.027898467871 | 4805.91795622 |
| 683.371298405467 | 417.74736234 | 683.371298405467 | 5733.56429354 | 683.371298405467 | 4734.79774055 |
| 680.7351940095303 | 397.80850389 | 680.7351940095303 | 5898.39959768 | 680.7351940095303 | 4656.12333186 |
| 678.1193490054249 | 378.828368094 | 678.1193490054249 | 6055.99922728 | 678.1193490054249 | 4570.26911456 |
| 675.5235307363206 | 360.895118941 | 675.5235307363206 | 6205.54964163 | 675.5235307363206 | 4477.64677268 |
| 672.9475100942127 | 344.093621716 | 672.9475100942127 | 6346.26689307 | 672.9475100942127 | 4378.7016303 |
| 670.3910614525139 | 328.505737342 | 670.3910614525139 | 6477.40388712 | 670.3910614525139 | 4273.90870311 |
| 667.8539626001781 | 314.210675157 | 667.8539626001781 | 6598.25746699 | 667.8539626001781 | 4163.76851312 |
| 665.335994677312 | 301.285396439 | 665.335994677312 | 6708.17523446 | 665.335994677312 | 4048.80272086 |
| 662.8369421122403 | 289.805060266 | 662.8369421122403 | 6806.56202087 | 662.8369421122403 | 3929.54963128 |
| 660.3565925599823 | 279.843502702 | 660.3565925599823 | 6892.88592514 | 660.3565925599823 | 3806.55962979 |
| 657.8947368421053 | 271.473739728 | 657.8947368421053 | 6966.68383982 | 657.8947368421053 | 3680.39060496 |
| 655.4511688879178 | 264.768483945 | 655.4511688879178 | 7027.56639233 | 655.4511688879178 | 3551.60341292 |
| 653.0256856769699 | 259.80066471 | 653.0256856769699 | 7075.22223523 | 653.0256856769699 | 3420.75743657 |
| 650.6180871828237 | 256.643941132 | 650.6180871828237 | 7109.42162722 | 650.6180871828237 | 3288.40628981 |
| 648.2281763180639 | 255.373197215 | 648.2281763180639 | 7130.01925606 | 648.2281763180639 | 3155.09371336 |
| 645.8557588805166 | 256.06500835 | 645.8557588805166 | 7136.9562637 | 645.8557588805166 | 3021.34970481 |
| 643.5006435006435 | 258.798068423 | 643.5006435006435 | 7130.26144502 | 643.5006435006435 | 2887.68692029 |
| 641.1626415900834 | 263.653566902 | 641.1626415900834 | 7110.05160184 | 641.1626415900834 | 2754.59738059 |
| 638.8415672913118 | 270.715505495 | 638.8415672913118 | 7076.5310455 | 638.8415672913118 | 2622.54950864 |
| 636.5372374283895 | 280.070944268 | 636.5372374283895 | 7029.99025213 | 636.5372374283895 | 2491.98551978 |
| 634.2494714587738 | 291.810167542 | 634.2494714587738 | 6970.80368614 | 634.2494714587738 | 2363.31918029 |
| 631.9780914261638 | 306.026760336 | 631.9780914261638 | 6899.42681785 | 631.9780914261638 | 2236.93394421 |
| 629.7229219143577 | 322.817586794 | 629.7229219143577 | 6816.39237179 | 629.7229219143577 | 2113.18147235 |
| 627.4837900020916 | 342.282662693 | 627.4837900020916 | 6722.30585152 | 627.4837900020916 | 1992.38053239 |
| 625.2605252188412 | 364.524914977 | 625.2605252188412 | 6617.84039565 | 625.2605252188412 | 1874.81627341 |
| 623.0529595015576 | 389.649822205 | 623.0529595015576 | 6503.73102747 | 623.0529595015576 | 1760.73986379 |
| 620.8609271523178 | 417.764930855 | 620.8609271523178 | 6380.76836743 | 620.8609271523178 | 1650.36847666 |
| 618.6842647968654 | 448.979243621 | 618.6842647968654 | 6249.79188293 | 618.6842647968654 | 1543.88560352 |
| 616.5228113440197 | 483.40247716 | 616.5228113440197 | 6111.68275456 | 616.5228113440197 | 1441.44167321 |
| 614.3764079459348 | 521.144188201 | 614.3764079459348 | 5967.35644053 | 614.3764079459348 | 1343.15495054 |
| 612.2448979591836 | 562.312768473 | 612.2448979591836 | 5817.75502311 | 612.2448979591836 | 1249.11268678 |
| 610.1281269066504 | 607.014310669 | 610.1281269066504 | 5663.83942115 | 610.1281269066504 | 1159.37249249 |
| 608.0259424402108 | 655.351349418 | 608.0259424402108 | 5506.58155207 | 608.0259424402108 | 1073.96390225 |
| 605.9381943041809 | 707.42148321 | 605.9381943041809 | 5346.95652477 | 605.9381943041809 | 992.89009999 |
| 603.864734299517 | 763.315885219 | 603.864734299517 | 5185.93494198 | 603.864734299517 | 916.129774011 |
| 601.8054162487462 | 823.117713061 | 601.8054162487462 | 5024.47538646 | 601.8054162487462 | 843.639070947 |
| 599.7600959616153 | 886.900429671 | 599.7600959616153 | 4863.51716082 | 599.7600959616153 | 775.353618946 |
| 597.7286312014345 | 954.726049665 | 597.7286312014345 | 4703.97334508 | 597.7286312014345 | 711.190591663 |
| 595.7108816521048 | 1026.64332768 | 595.7108816521048 | 4546.72422993 | 595.7108816521048 | 651.05078623 |
| 593.7067088858104 | 1102.68590737 | 593.7067088858104 | 4392.61117716 | 593.7067088858104 | 594.820690343 |
| 591.7159763313609 | 1182.87045162 | 591.7159763313609 | 4242.43095182 | 591.7159763313609 | 542.374515723 |
| 589.7385492431688 | 1267.19477675 | 589.7385492431688 | 4096.93056359 | 589.7385492431688 | 493.576177535 |
| 587.7742946708463 | 1355.63601484 | 587.7742946708463 | 3956.80264768 | 587.7742946708463 | 448.281201805 |
| 585.8230814294083 | 1448.14883014 | 585.8230814294083 | 3822.68140886 | 585.8230814294083 | 406.338545353 |
| 583.8847800700661 | 1544.66371669 | 583.8847800700661 | 3695.13914505 | 583.8847800700661 | 367.592315301 |
| 581.9592628516003 | 1645.08540522 | 581.9592628516003 | 3574.68336065 | 581.9592628516003 | 331.883377717 |
| 580.046403712297 | 1749.29140803 | 580.046403712297 | 3461.75447362 | 580.046403712297 | 299.050847361 |
| 578.1460782424359 | 1857.13073073 | 578.1460782424359 | 3356.7241145 | 578.1460782424359 | 268.933452847 |
| 576.2581636573184 | 1968.42277972 | 576.2581636573184 | 3259.89401057 | 576.2581636573184 | 241.370773711 |
| 574.3825387708214 | 2082.95649315 | 574.3825387708214 | 3171.49544345 | 574.3825387708214 | 216.204347917 |
| 572.5190839694656 | 2200.48972248 | 572.5190839694656 | 3091.68926455 | 572.5190839694656 | 193.278650188 |
| 570.6676811869887 | 2320.74888975 | 570.6676811869887 | 3020.56644891 | 570.6676811869887 | 172.44194321 |
| 568.8282138794084 | 2443.42894353 | 568.8282138794084 | 2958.14916498 | 568.8282138794084 | 153.547005228 |
| 567.000567000567 | 2568.1936339 | 567.000567000567 | 2904.39233544 | 567.000567000567 | 136.451738818 |
| 565.1846269781461 | 2694.67612361 | 565.1846269781461 | 2859.18566177 | 565.1846269781461 | 121.01966665 |
| 563.3802816901408 | 2822.47994883 | 563.3802816901408 | 2822.35608366 | 563.3802816901408 | 107.120320949 |
| 561.5874204417821 | 2951.18033884 | 561.5874204417821 | 2793.67064298 | 561.5874204417821 | 94.6295339957 |
| 559.8059339428997 | 3080.32589943 | 559.8059339428997 | 2772.83972097 | 559.8059339428997 | 83.4296374926 |
| 558.0357142857143 | 3209.44066002 | 558.0357142857143 | 2759.52061637 | 558.0357142857143 | 73.4095789369 |
| 556.2766549230483 | 3338.02647935 | 556.2766549230483 | 2753.32143177 | 556.2766549230483 | 64.4649632815 |
| 554.52865064695 | 3465.56579917 | 554.52865064695 | 2753.80523483 | 554.52865064695 | 56.498028182 |
| 552.791597567717 | 3591.52473005 | 552.791597567717 | 2760.49446073 | 552.791597567717 | 49.417561007 |
| 551.0653930933137 | 3715.35644798 | 551.0653930933137 | 2772.87552193 | 551.0653930933137 | 43.1387655677 |
| 549.3499359091741 | 3836.50487499 | 549.3499359091741 | 2790.40359106 | 549.3499359091741 | 37.5830862076 |
| 547.645125958379 | 3954.40861178 | 547.645125958379 | 2812.50752246 | 547.645125958379 | 32.6779964997 |
| 545.950864422202 | 4068.50508571 | 545.950864422202 | 2838.59487794 | 545.950864422202 | 28.3567593546 |
| 544.2670537010159 | 4178.23487266 | 544.2670537010159 | 2868.05702174 | 544.2670537010159 | 24.5581648442 |
| 542.5935973955508 | 4283.04614742 | 542.5935973955508 | 2900.27425006 | 542.5935973955508 | 21.2262515238 |
| 540.9304002884962 | 4382.39921385 | 540.9304002884962 | 2934.62091979 | 540.9304002884962 | 18.3100164965 |
| 539.2773683264425 | 4475.77106301 | 539.2773683264425 | 2970.47054168 | 539.2773683264425 | 15.763118911 |
| 537.6344086021505 | 4562.65990581 | 537.6344086021505 | 3007.20080267 | 537.6344086021505 | 13.5435810442 |
| 536.0014293371448 | 4642.58962497 | 536.0014293371448 | 3044.19848284 | 536.0014293371448 | 11.6134905857 |
| 534.3783398646241 | 4715.11409111 | 534.3783398646241 | 3080.8642322 | 534.3783398646241 | 9.93870722896 |
| 532.7650506126798 | 4779.82128783 | 532.7650506126798 | 3116.61717383 | 532.7650506126798 | 8.4885761884 |
| 531.1614730878186 | 4836.33719238 | 531.1614730878186 | 3150.89929999 | 531.1614730878186 | 7.23565080096 |
| 529.5675198587819 | 4884.32936046 | 529.5675198587819 | 3183.17962938 | 529.5675198587819 | 6.15542594939 |
| 527.9831045406547 | 4923.51016675 | 527.9831045406547 | 3212.95809511 | 527.9831045406547 | 5.22608365356 |
| 526.4081417792595 | 4953.63965709 | 526.4081417792595 | 3239.76913426 | 526.4081417792595 | 4.42825182468 |
| 524.8425472358292 | 4974.5279724 | 524.8425472358292 | 3263.18495249 | 524.8425472358292 | 3.74477686217 |
| 523.2862375719518 | 4986.03731019 | 523.2862375719518 | 3282.81843927 | 523.2862375719518 | 3.16051049465 |
| 521.7391304347826 | 4988.08339546 | 521.7391304347826 | 3298.32571198 | 521.7391304347826 | 2.66211102436 |
| 520.2011444425177 | 4980.63643914 | 520.2011444425177 | 3309.40827053 | 520.2011444425177 | 2.23785892655 |
| 518.6721991701245 | 4963.7215695 | 518.6721991701245 | 3315.81474711 | 518.6721991701245 | 1.87748658035 |
| 517.1522151353215 | 4937.4187287 | 517.1522151353215 | 3317.34223958 | 517.1522151353215 | 1.57202176286 |
| 515.6411137848057 | 4901.86203453 | 515.6411137848057 | 3313.83722089 | 515.6411137848057 | 1.3136444217 |
| 514.1388174807198 | 4857.23861437 | 514.1388174807198 | 3305.19602068 | 514.1388174807198 | 1.09555615017 |
| 512.6452494873547 | 4803.7869259 | 512.6452494873547 | 3291.36487964 | 512.6452494873547 | 0.91186172102 |
| 511.1603339580848 | 4741.79458609 | 511.1603339580848 | 3272.3395814 | 511.1603339580848 | 0.757461987302 |
| 509.683995922528 | 4671.59573661 | 509.683995922528 | 3248.1646705 | 509.683995922528 | 0.627957428575 |
| 508.2161612739285 | 4593.56798014 | 508.2161612739285 | 3218.93226978 | 508.2161612739285 | 0.519561606483 |
| 506.7567567567567 | 4508.12892748 | 506.7567567567567 | 3184.78051376 | 506.7567567567567 | 0.429023792094 |
| 505.3057099545225 | 4415.73240047 | 505.3057099545225 | 3145.89161883 | 505.3057099545225 | 0.353560036877 |
| 503.8629492777964 | 4316.86433984 | 503.8629492777964 | 3102.48961417 | 503.8629492777964 | 0.290791977491 |
| 502.4284039524367 | 4212.03847025 | 502.4284039524367 | 3054.83776076 | 502.4284039524367 | 0.238692689958 |
| 501.00200400801606 | 4101.79177769 | 501.00200400801606 | 3003.23568807 | 501.00200400801606 | 0.195538939604 |
| 499.5836802664446 | 3986.67985541 | 499.5836802664446 | 2948.0162808 | 499.5836802664446 | 0.159869207967 |
| 498.1733643307871 | 3867.27217566 | 498.1733643307871 | 2889.54234945 | 498.1733643307871 | 0.130446915423 |
| 496.7709885742673 | 3744.14734407 | 496.7709885742673 | 2828.20312001 | 496.7709885742673 | 0.106228297424 |
| 495.3764861294584 | 3617.8883922 | 495.3764861294584 | 2764.41057881 | 495.3764861294584 | 0.0863344321578 |
| 493.98979087765514 | 3489.0781621 | 493.98979087765514 | 2698.59570879 | 493.98979087765514 | 0.0700269572139 |
| 492.61083743842363 | 3358.29483386 | 492.61083743842363 | 2631.20465335 | 492.61083743842363 | 0.0566870520032 |
| 491.2395611593253 | 3226.10764334 | 491.2395611593253 | 2562.69484328 | 491.2395611593253 | 0.0457973005726 |
| 489.8758981058131 | 3093.07283374 | 489.8758981058131 | 2493.53112097 | 489.8758981058131 | 0.0369260858242 |
| 488.5197850512946 | 2959.72987938 | 488.5197850512946 | 2424.18189483 | 488.5197850512946 | 0.0297142006153 |
| 487.17115946735953 | 2826.59801557 | 487.17115946735953 | 2355.1153548 | 487.17115946735953 | 0.0238633936246 |
| 485.82995951416996 | 2694.17310249 | 485.82995951416996 | 2286.79577775 | 485.82995951416996 | 0.0191265980836 |
| 484.49612403100775 | 2562.92484578 | 484.49612403100775 | 2219.67994903 | 484.49612403100775 | 0.0152996194326 |
| 483.16959252697694 | 2433.2943905 | 483.16959252697694 | 2154.21372418 | 483.16959252697694 | 0.0122140836516 |
| 481.8503051718599 | 2305.69229949 | 481.8503051718599 | 2090.82875157 | 481.8503051718599 | 0.00973147147886 |
| 480.5382027871216 | 2180.49692145 | 480.5382027871216 | 2029.93937455 | 480.5382027871216 | 0.00773808502269 |
| 479.23322683706067 | 2058.05314843 | 479.23322683706067 | 1971.93972864 | 479.23322683706067 | 0.00614081248994 |
| 477.9353194201051 | 1938.67155741 | 477.9353194201051 | 1917.20104658 | 477.9353194201051 | 0.00486357400697 |
| 476.64442326024783 | 1822.62792563 | 476.64442326024783 | 1866.06918191 | 476.64442326024783 | 0.00384434691582 |
| 475.3604816986214 | 1710.16310473 | 475.3604816986214 | 1818.86235876 | 475.3604816986214 | 0.00303268262353 |
| 474.08343868520853 | 1601.48323518 | 474.08343868520853 | 1775.869154 | 474.08343868520853 | 0.00238763919698 |
| 472.8132387706856 | 1496.76027844 | 472.8132387706856 | 1737.34671552 | 472.8132387706856 | 0.00187606456558 |
| 471.5498270983967 | 1396.13284181 | 471.5498270983967 | 1703.51921876 | 471.5498270983967 | 0.00147117454969 |
| 470.29314939645707 | 1299.70726838 | 470.29314939645707 | 1674.5765625 | 470.29314939645707 | 0.001151378103 |
| 469.04315196998124 | 1207.55896257 | 469.04315196998124 | 1650.67330329 | 469.04315196998124 | 0.00089930926222 |
| 467.7997816934352 | 1119.73392069 | 467.7997816934352 | 1631.92782729 | 467.7997816934352 | 0.00070103145247 |
| 466.5629860031104 | 1036.2504352 | 466.5629860031104 | 1618.4217574 | 466.5629860031104 | 0.000545385108496 |
| 465.33271288971605 | 957.100941138 | 465.33271288971605 | 1610.1995933 | 465.33271288971605 | 0.000423454138032 |
| 464.10891089108907 | 882.253973521 | 464.10891089108907 | 1607.26858123 | 464.10891089108907 | 0.000328130665283 |
| 462.8915290850177 | 811.656205384 | 462.8915290850177 | 1609.59881075 | 462.8915290850177 | 0.000253760831025 |
| 461.68051708217905 | 745.234537163 | 461.68051708217905 | 1617.12353507 | 461.68051708217905 | 0.000195857265406 |
| 460.47582501918646 | 682.8982098 | 460.47582501918646 | 1629.73971175 | 460.47582501918646 | 0.000150866256393 |
| 459.2774035517452 | 624.540915702 | 459.2774035517452 | 1647.30876039 | 459.2774035517452 | 0.000115979670049 |
| 458.0852038479157 | 570.042883808 | 458.0852038479157 | 1669.65753398 | 458.0852038479157 | 8.89833907397e-05 |
| 456.89917758148033 | 519.272917298 | 456.89917758148033 | 1696.5795001 | 456.89917758148033 | 6.8135486037e-05 |
| 455.7192769254139 | 472.09036488 | 455.7192769254139 | 1727.83612802 | 455.7192769254139 | 5.20685029081e-05 |
| 454.54545454545456 | 428.347009101 | 454.54545454545456 | 1763.15847732 | 454.54545454545456 | 3.97113039522e-05 |
| 453.3776635937736 | 387.888857668 | 453.3776635937736 | 1802.24898273 | 453.3776635937736 | 3.02266855487e-05 |
| 452.2158577027434 | 350.557826278 | 452.2158577027434 | 1844.7834294 | 452.2158577027434 | 2.29617101875e-05 |
| 451.05999097880016 | 316.193303935 | 451.05999097880016 | 1890.4131113 | 451.05999097880016 | 1.74082556724e-05 |
| 449.9100179964007 | 284.633594125 | 449.9100179964007 | 1938.76716455 | 449.9100179964007 | 1.31717537659e-05 |
| 448.7658937920718 | 255.717227437 | 448.7658937920718 | 1989.4550658 | 448.7658937920718 | 9.94647673803e-06 |
| 447.6275738585497 | 229.284143391 | 447.6275738585497 | 2042.06928424 | 447.6275738585497 | 7.49604628849e-06 |
| 446.49501413900873 | 205.176741113 | 446.49501413900873 | 2096.18807383 | 446.49501413900873 | 5.63809732091e-06 |
| 445.36817102137763 | 183.240800283 | 445.36817102137763 | 2151.37839066 | 445.36817102137763 | 4.23223910875e-06 |
| 444.247001332741 | 163.326275335 | 444.247001332741 | 2207.19891824 | 444.247001332741 | 3.17062692351e-06 |
| 443.13146233382565 | 145.287967223 | 443.13146233382565 | 2263.2031814 | 443.13146233382565 | 2.37059524498e-06 |
| 442.02151171357 | 128.98607823 | 442.02151171357 | 2318.94272768 | 442.02151171357 | 1.76891511581e-06 |
| 440.9171075837742 | 114.286656231 | 440.9171075837742 | 2373.97035313 | 440.9171075837742 | 1.31732791613e-06 |
| 439.8182084738308 | 101.061935563 | 439.8182084738308 | 2427.84334749 | 439.8182084738308 | 9.79079855555e-07 |
| 438.72477332553376 | 89.1905822253 | 438.72477332553376 | 2480.1267325 | 438.72477332553376 | 7.26239142007e-07 |
| 437.636761487965 | 78.5578514966 | 437.636761487965 | 2530.39646557 | 437.636761487965 | 5.37623825268e-07 |
| 436.5541327124563 | 69.0556662817 | 436.5541327124563 | 2578.24257996 | 436.5541327124563 | 3.97204973464e-07 |
| 435.4768471476266 | 60.5826245697 | 435.4768471476266 | 2623.27223234 | 435.4768471476266 | 2.92878954397e-07 |
| 434.4048653344918 | 53.0439443136 | 434.4048653344918 | 2665.11262794 | 434.4048653344918 | 2.15525652917e-07 |
| 433.3381482016467 | 46.3513538679 | 433.3381482016467 | 2703.41379408 | 433.3381482016467 | 1.58287671185e-07 |
| 432.2766570605187 | 40.4229358375 | 432.2766570605187 | 2737.85117319 | 432.2766570605187 | 1.16019910367e-07 |
| 431.22035360069 | 35.1829318327 | 431.22035360069 | 2768.12800789 | 431.22035360069 | 8.48702101176e-08 |
| 430.1691998852882 | 30.5615151935 | 430.1691998852882 | 2793.97749189 | 430.1691998852882 | 6.19605614892e-08 |
| 429.1231583464454 | 26.4945382733 | 429.1231583464454 | 2815.16466294 | 429.1231583464454 | 4.51453191318e-08 |
| 428.0821917808219 | 22.9232603503 | 428.0821917808219 | 2831.48801624 | 428.0821917808219 | 3.28282268107e-08 |
| 427.0462633451957 | 19.7940617041 | 427.0462633451957 | 2842.78081969 | 427.0462633451957 | 2.3824261004e-08 |
| 426.01533655211585 | 17.0581488389 | 426.01533655211585 | 2848.91211586 | 426.01533655211585 | 1.72555484013e-08 |
| 424.9893752656184 | 14.6712552888 | 424.9893752656184 | 2849.78739864 | 424.9893752656184 | 1.24731288571e-08 |
| 423.96834369700395 | 12.5933418979 | 423.96834369700395 | 2845.34895717 | 423.96834369700395 | 8.9982771696e-09 |
| 422.9522064006767 | 10.7882999417 | 422.9522064006767 | 2835.57588274 | 422.9522064006767 | 6.47859211958e-09 |
| 421.9409282700422 | 9.22365995483 | 421.9409282700422 | 2820.48373949 | 421.9409282700422 | 4.6552094404e-09 |
| 420.93447453346425 | 7.87030865568 | 420.93447453346425 | 2800.12390321 | 420.93447453346425 | 3.33837504875e-09 |
| 419.9328107502799 | 6.70221591478 | 419.9328107502799 | 2774.58257723 | 419.9328107502799 | 2.38928711442e-09 |
| 418.93590280687056 | 5.696173307 | 418.93590280687056 | 2743.9794983 | 418.93590280687056 | 1.70662801423e-09 |
| 417.94371691278906 | 4.83154541498 | 417.94371691278906 | 2708.46634928 | 417.94371691278906 | 1.21659692312e-09 |
| 416.9562195969423 | 4.09003471741 | 416.9562195969423 | 2668.2248993 | 416.9562195969423 | 8.65549423178e-10 |
| 415.97337770382694 | 3.45546059832 | 415.97337770382694 | 2623.46489521 | 415.97337770382694 | 6.14574225891e-10 |
| 414.99515838981876 | 2.91355275296 | 414.99515838981876 | 2574.42173137 | 414.99515838981876 | 4.35505985477e-10 |
| 414.0215291195142 | 2.45175904077 | 414.0215291195142 | 2521.35392721 | 414.0215291195142 | 3.08000362385e-10 |
| 413.0524576621231 | 2.05906764435 | 413.0524576621231 | 2464.54044424 | 413.0524576621231 | 2.1739304242e-10 |
| 412.08791208791206 | 1.72584323348 | 412.08791208791206 | 2404.27787568 | 412.08791208791206 | 1.53136024142e-10 |
| 411.1278607646978 | 1.44367670267 | 411.1278607646978 | 2340.87754305 | 411.1278607646978 | 1.07658024872e-10 |
| 410.17227235438884 | 1.2052479471 | 410.17227235438884 | 2274.66253455 | 410.17227235438884 | 7.55357876175e-11 |
| 409.22111580957574 | 1.0042010625 | 409.22111580957574 | 2205.96472004 | 409.22111580957574 | 5.28927843801e-11 |
| 408.2743603701687 | 0.835031296817 | 408.2743603701687 | 2135.12177702 | 408.2743603701687 | 3.69638680302e-11 |
| 407.33197556008145 | 0.692983042814 | 407.33197556008145 | 2062.47426071 | 407.33197556008145 | 2.5780759403e-11 |
| 406.39393118396094 | 0.573958139179 | 406.39393118396094 | 1988.36275017 | 406.39393118396094 | 1.79453244265e-11 |
| 405.46019732396263 | 0.474433739623 | 405.46019732396263 | 1913.12510009 | 405.46019732396263 | 1.24664912329e-11 |
| 404.53074433656957 | 0.391389013989 | 404.53074433656957 | 1837.09382572 | 404.53074433656957 | 8.6431981816e-12 |
| 403.6055428494551 | 0.322239959426 | 403.6055428494551 | 1760.59364574 | 403.6055428494551 | 5.98056231238e-12 |
| 402.68456375838923 | 0.26478162184 | 402.68456375838923 | 1683.93920462 | 402.68456375838923 | 4.12996985456e-12 |
| 401.76777822418643 | 0.2171370562 | 401.76777822418643 | 1607.4329932 | 401.76777822418643 | 2.84635494906e-12 |
| 400.85515766969536 | 0.177712387347 | 400.85515766969536 | 1531.36348244 | 400.85515766969536 | 1.95780099804e-12 |
| 399.9466737768297 | 0.145157369363 | 399.9466737768297 | 1456.00348216 | 399.9466737768297 | 1.34395691894e-12 |
| 399.0422984836393 | 0.118330880121 | 399.0422984836393 | 1381.60873285 | 399.0422984836393 | 9.20745198208e-13 |
| 398.14200398142003 | 0.0962708273647 | 398.14200398142003 | 1308.41673526 | 398.14200398142003 | 6.29550960787e-13 |
| 397.24576271186436 | 0.0781679827034 | 397.24576271186436 | 1236.64581917 | 397.24576271186436 | 4.29595410216e-13 |
| 396.3535473642489 | 0.0633432995306 | 396.3535473642489 | 1166.49444928 | 396.3535473642489 | 2.92567235217e-13 |
| 395.46533087266016 | 0.0512283095565 | 395.46533087266016 | 1098.14076333 | 395.46533087266016 | 1.98851584403e-13 |
| 394.5810864132579 | 0.0413482299124 | 394.5810864132579 | 1031.7423346 | 394.5810864132579 | 1.3488688858e-13 |
| 393.7007874015748 | 0.0333074483187 | 393.7007874015748 | 967.436148551 | 393.7007874015748 | 9.13161791405e-14 |
| 392.82440748985204 | 0.0267770873513 | 392.82440748985204 | 905.3387809 | 392.82440748985204 | 6.16968567902e-14 |
| 391.9519205644107 | 0.0214843802497 | 391.9519205644107 | 845.546762657 | 391.9519205644107 | 4.1602138909e-14 |
| 391.08330074305826 | 0.017203619876 | 391.08330074305826 | 788.137116081 | 391.08330074305826 | 2.79966194222e-14 |
| 390.2185223725286 | 0.0137484693324 | 390.2185223725286 | 733.168044139 | 390.2185223725286 | 1.88032465539e-14 |
| 389.3575600259571 | 0.0109654473839 | 389.3575600259571 | 680.679755257 | 389.3575600259571 | 1.26036809478e-14 |
| 388.5003885003885 | 0.00872842426352 | 388.5003885003885 | 630.695404533 | 388.5003885003885 | 8.43139185783e-15 |
| 387.6469828143171 | 0.00693398374089 | 387.6469828143171 | 583.222132355 | 387.6469828143171 | 5.62909347403e-15 |
| 386.7973182052604 | 0.00549752560726 | 386.7973182052604 | 538.252181434 | 386.7973182052604 | 3.75072272541e-15 |
| 385.95137012736393 | 0.00434999909534 | 385.95137012736393 | 495.76407358 | 385.95137012736393 | 2.49418562771e-15 |
| 385.1091142490372 | 0.0034351723313 | 385.1091142490372 | 455.723828202 | 385.1091142490372 | 1.65531213154e-15 |
| 384.2705264506212 | 0.00270735584534 | 384.2705264506212 | 418.086205279 | 384.2705264506212 | 1.0963982574e-15 |
| 383.4355828220859 | 0.0021295095798 | 383.4355828220859 | 382.795956643 | 383.4355828220859 | 7.24759786403e-16 |
| 382.6042596607575 | 0.00167167286313 | 382.6042596607575 | 349.789070551 | 382.6042596607575 | 4.78142285483e-16 |
| 381.77653346907607 | 0.0013096655956 | 381.77653346907607 | 318.993995875 | 381.77653346907607 | 3.14816532357e-16 |
| 380.95238095238096 | 0.00102401654139 | 380.95238095238096 | 290.33283366 | 380.95238095238096 | 2.0686890794e-16 |
| 380.1317790167258 | 0.000799081261626 | 380.1317790167258 | 263.722485255 | 380.1317790167258 | 1.35665751769e-16 |
| 379.31470476672143 | 0.000622317964064 | 379.31470476672143 | 239.075747783 | 379.31470476672143 | 8.87937796232e-17 |
| 378.5011355034065 | 0.000483694490321 | 378.5011355034065 | 216.302349199 | 378.5011355034065 | 5.8000558124e-17 |
| 377.69104872214524 | 0.000375203905589 | 377.69104872214524 | 195.309916729 | 377.69104872214524 | 3.78110829066e-17 |
| 376.88442211055275 | 0.000290469784982 | 376.88442211055275 | 176.004873902 | 376.88442211055275 | 2.46004681436e-17 |
| 376.081233546446 | 0.000224425382896 | 376.081233546446 | 158.293262818 | 376.081233546446 | 1.59736785594e-17 |
| 375.28146109582184 | 0.000173053497565 | 375.28146109582184 | 142.081489588 | 375.28146109582184 | 1.03515127861e-17 |
| 374.48508301086 | 0.000133176065042 | 374.48508301086 | 127.276992099 | 374.48508301086 | 6.69483715948e-18 |
| 373.69207772795215 | 0.000102284390956 | 373.69207772795215 | 113.788830379 | 373.69207772795215 | 4.32129112937e-18 |
| 372.9024238657551 | 7.84025038547e-05 | 372.9024238657551 | 101.528200806 | 372.9024238657551 | 2.78371229256e-18 |
| 372.11610022326965 | 5.99774341011e-05 | 372.11610022326965 | 90.4088763029 | 372.11610022326965 | 1.78966803534e-18 |
| 371.33308577794276 | 4.57913249341e-05 | 371.33308577794276 | 80.3475753955 | 371.33308577794276 | 1.14830677857e-18 |
| 370.55335968379444 | 3.48912004467e-05 | 370.55335968379444 | 71.2642636334 | 370.55335968379444 | 7.35327297682e-19 |
| 369.7769012695673 | 2.65329773203e-05 | 369.7769012695673 | 63.0823913931 | 369.7769012695673 | 4.69938213773e-19 |
| 369.0036900369003 | 2.01369378178e-05 | 369.0036900369003 | 55.7290724626 | 369.0036900369003 | 2.99735475909e-19 |
| 368.23370565852457 | 1.52524018426e-05 | 368.23370565852457 | 49.1352080935 | 368.23370565852457 | 1.90797573457e-19 |
| 367.4669279764821 | 1.15297638605e-05 | 367.4669279764821 | 43.2355613852 | 367.4669279764821 | 1.21211788463e-19 |
| 366.7033370003667 | 8.6984114831e-06 | 366.7033370003667 | 37.9687869517 | 366.7033370003667 | 7.6851824862e-20 |
| 365.9429129055867 | 6.54932954587e-06 | 365.9429129055867 | 33.2774208243 | 365.9429129055867 | 4.86296140166e-20 |
| 365.1856360316494 | 4.92142755162e-06 | 365.1856360316494 | 29.107835473 | 365.1856360316494 | 3.07103505356e-20 |
| 364.4314868804664 | 3.69081863114e-06 | 364.4314868804664 | 25.4101646917 | 364.4314868804664 | 1.93555732265e-20 |
| 363.68044611468054 | 2.76243244177e-06 | 363.68044611468054 | 22.1382029046 | 363.68044611468054 | 1.21748779111e-20 |
| 362.93249455601256 | 2.06346914227e-06 | 362.93249455601256 | 19.2492832153 | 362.93249455601256 | 7.64294096264e-21 |
| 362.1876131836291 | 1.53830213038e-06 | 362.1876131836291 | 16.704138254 | 362.1876131836291 | 4.78843622913e-21 |
| 361.4457831325301 | 1.14451806621e-06 | 361.4457831325301 | 14.4667475799 | 361.4457831325301 | 2.9940856795e-21 |
| 360.7069856919562 | 8.49847516865e-07 | 360.7069856919562 | 12.5041750858 | 360.7069856919562 | 1.86840944109e-21 |
| 359.97120230381563 | 6.29791380056e-07 | 359.97120230381563 | 10.7863995288 | 359.97120230381563 | 1.16363612426e-21 |
| 359.2384145611304 | 4.65789568345e-07 | 359.2384145611304 | 9.28614098396 | 359.2384145611304 | 7.23268665174e-22 |
| 358.50860420650093 | 3.43811299567e-07 | 358.50860420650093 | 7.97868569351 | 358.50860420650093 | 4.48662134515e-22 |
| 357.7817531305903 | 2.53272405176e-07 | 357.7817531305903 | 6.84171146959 | 357.7817531305903 | 2.7776434631e-22 |
| 357.057843370626 | 1.86205687828e-07 | 357.057843370626 | 5.85511550085 | 357.057843370626 | 1.71621183732e-22 |
| 356.33685710892024 | 1.36626627779e-07 | 356.33685710892024 | 5.00084612572 | 356.33685710892024 | 1.05828490142e-22 |
| 355.6187766714082 | 1.00049539196e-07 | 355.6187766714082 | 4.26273986188 | 355.6187766714082 | 6.51285815997e-23 |
| 354.9035845262037 | 7.31193247769e-08 | 354.9035845262037 | 3.62636472972 | 354.9035845262037 | 4.00016543583e-23 |
| 354.1912632821723 | 5.33318423535e-08 | 354.1912632821723 | 3.07887067619 | 354.1912632821723 | 2.45200639668e-23 |
| 353.48179568752204 | 3.88220384209e-08 | 353.48179568752204 | 2.60884769624 | 353.48179568752204 | 1.5000390246e-23 |
| 352.77516462841015 | 2.82037863674e-08 | 352.77516462841015 | 2.20619206191 | 352.77516462841015 | 9.15842576524e-24 |
| 352.07135312756714 | 2.04490826292e-08 | 352.07135312756714 | 1.86198090373 | 352.07135312756714 | 5.58054242815e-24 |
| 351.3703443429374 | 1.4797132898e-08 | 351.3703443429374 | 1.56835524527 | 351.3703443429374 | 3.39366767822e-24 |
| 350.6721215663355 | 1.06860856131e-08 | 350.6721215663355 | 1.31841146805 | 350.6721215663355 | 2.05967891421e-24 |
| 349.9766682221185 | 7.70188542585e-09 | 349.9766682221185 | 1.10610108018 | 349.9766682221185 | 1.24757610117e-24 |
| 349.2839678658749 | 5.54003855253e-09 | 349.2839678658749 | 0.926138575841 | 349.2839678658749 | 7.54174568474e-25 |
| 348.59400418312805 | 3.9770939306e-09 | 348.59400418312805 | 0.773917103373 | 348.59400418312805 | 4.55002762239e-25 |
| 347.90676098805517 | 2.84941848177e-09 | 347.90676098805517 | 0.645431605669 | 347.90676098805517 | 2.7396399836e-25 |
| 347.2222222222222 | 2.03743589056e-09 | 347.2222222222222 | 0.537209055743 | 347.2222222222222 | 1.64630490284e-25 |
| 346.54037195333257 | 1.45394839925e-09 | 346.54037195333257 | 0.446245381924 | 346.54037195333257 | 9.8733460813e-26 |
| 345.8611943739912 | 1.03550299785e-09 | 345.8611943739912 | 0.369948659118 | 345.8611943739912 | 5.9095683741e-26 |
| 345.1846738004832 | 7.36022434811e-10 | 345.1846738004832 | 0.306088133717 | 345.1846738004832 | 3.53007942116e-26 |
| 344.5107946715664 | 5.2211728194e-10 | 344.5107946715664 | 0.252748648646 | 344.5107946715664 | 2.10450762692e-26 |
| 343.8395415472779 | 3.69642933744e-10 | 343.8395415472779 | 0.208290040361 | 343.8395415472779 | 1.25214275377e-26 |
| 343.17089910775564 | 2.61176484429e-10 | 343.17089910775564 | 0.171311090251 | 343.17089910775564 | 7.43523155177e-27 |
| 342.50485215207215 | 1.84171768287e-10 | 342.50485215207215 | 0.140617627711 | 342.50485215207215 | 4.40628378813e-27 |
| 341.84138559708293 | 1.29613239839e-10 | 341.84138559708293 | 0.115194400198 | 341.84138559708293 | 2.60608000455e-27 |
| 341.1804844762879 | 9.1035964332e-11 | 341.1804844762879 | 0.094180346009 | 341.1804844762879 | 1.53829750809e-27 |
| 340.522133938706 | 6.38137033467e-11 | 340.522133938706 | 0.0768469275621 | 340.522133938706 | 9.0621290387e-28 |
| 339.86631924776253 | 4.46428818811e-11 | 339.86631924776253 | 0.0625792059783 | 339.86631924776253 | 5.32791711924e-28 |
| 339.2130257801899 | 3.1169355073e-11 | 339.2130257801899 | 0.0508593612201 | 339.2130257801899 | 3.12623766903e-28 |
| 338.56223902494077 | 2.1719045284e-11 | 338.56223902494077 | 0.0412523854571 | 338.56223902494077 | 1.83072805141e-28 |
| 337.91394458211306 | 1.51039648186e-11 | 337.91394458211306 | 0.0333937003573 | 337.91394458211306 | 1.06994879364e-28 |
| 337.2681281618887 | 1.04828294391e-11 | 337.2681281618887 | 0.0269784713257 | 337.2681281618887 | 6.24078848709e-29 |
| 336.6247755834829 | 7.26111630984e-12 | 336.6247755834829 | 0.0217524131079 | 336.6247755834829 | 3.63289835583e-29 |
| 335.9838727741068 | 5.01955926603e-12 | 335.9838727741068 | 0.0175039014806 | 335.9838727741068 | 2.11059227014e-29 |
| 335.3454057679409 | 3.46310052416e-12 | 335.3454057679409 | 0.0140572248318 | 335.3454057679409 | 1.22375013428e-29 |
| 334.709360705121 | 2.38452526577e-12 | 334.709360705121 | 0.0112668272302 | 334.709360705121 | 7.08138936916e-30 |
| 334.07572383073494 | 1.63861179691e-12 | 334.07572383073494 | 0.00901241105107 | 334.07572383073494 | 4.08960638159e-30 |
| 333.44448149383123 | 1.12379616046e-12 | 333.44448149383123 | 0.00719478235864 | 333.44448149383123 | 2.3571208963e-30 |
| 332.81562014643885 | 7.69194780531e-13 | 332.81562014643885 | 0.0057323360685 | 332.81562014643885 | 1.35587458145e-30 |
| 332.1891263425977 | 5.2543915285e-13 | 332.1891263425977 | 0.00455809045418 | 332.1891263425977 | 7.78385062728e-31 |
| 331.5649867374005 | 3.58216718058e-13 | 331.5649867374005 | 0.00361719188626 | 331.5649867374005 | 4.45971165685e-31 |
| 330.9431880860452 | 2.43728652566e-13 | 330.9431880860452 | 0.00286482085527 | 330.9431880860452 | 2.5500951208e-31 |
| 330.323717242898 | 1.65502531659e-13 | 330.323717242898 | 0.00226443940718 | 330.323717242898 | 1.45526905084e-31 |
| 329.70656116056705 | 1.12160519521e-13 | 329.70656116056705 | 0.00178632818918 | 329.70656116056705 | 8.28833925831e-32 |
| 329.0917068889864 | 7.58599757769e-14 | 329.0917068889864 | 0.00140636844079 | 329.0917068889864 | 4.71117306419e-32 |
| 328.47914157451 | 5.12062183316e-14 | 328.47914157451 | 0.00110503055213 | 328.47914157451 | 2.67256274544e-32 |
| 327.86885245901635 | 3.44961020435e-14 | 327.86885245901635 | 0.000866536323319 | 327.86885245901635 | 1.51308761026e-32 |
| 327.26082687902255 | 2.31928788964e-14 | 327.26082687902255 | 0.0006781668729 | 327.26082687902255 | 8.54943772079e-33 |
| 326.6550522648083 | 1.5562401396e-14 | 326.6550522648083 | 0.000529692329501 | 326.6550522648083 | 4.82112446749e-33 |
| 326.05151613955 | 1.04216355376e-14 | 326.05151613955 | 0.000412903067512 | 326.05151613955 | 2.71329145867e-33 |
| 325.4502061184639 | 6.96518193837e-15 | 325.4502061184639 | 0.000321225377132 | 325.4502061184639 | 1.52398911288e-33 |
| 324.8511099079588 | 4.64586263592e-15 | 324.8511099079588 | 0.000249407149611 | 324.8511099079588 | 8.54288567109e-34 |
| 324.25421530479895 | 3.09269847432e-15 | 324.25421530479895 | 0.000193261463195 | 324.25421530479895 | 4.77930379302e-34 |
| 323.65951019527455 | 2.05468907007e-15 | 323.65951019527455 | 0.00014945792229 | 323.65951019527455 | 2.66846796058e-34 |
| 323.0669825543829 | 1.36236022801e-15 | 323.0669825543829 | 0.00011535327545 | 323.0669825543829 | 1.48695101415e-34 |
| 322.4766204450177 | 9.01519484688e-16 | 322.4766204450177 | 8.88542558475e-05 | 322.4766204450177 | 8.26929800498e-35 |
| 321.88841201716735 | 5.95381853505e-16 | 321.88841201716735 | 6.83067858432e-05 | 321.88841201716735 | 4.58963277001e-35 |
| 321.3023455071222 | 3.92422034341e-16 | 321.3023455071222 | 5.24066958875e-05 | 321.3023455071222 | 2.5422868166e-35 |
| 320.71840923669015 | 2.58135949335e-16 | 320.71840923669015 | 4.01279544196e-05 | 320.71840923669015 | 1.40542756095e-35 |
| 320.1365916124213 | 1.6946534949e-16 | 320.1365916124213 | 3.06651135106e-05 | 320.1365916124213 | 7.75406968276e-36 |
| 319.5568811248402 | 1.11032635147e-16 | 319.5568811248402 | 2.33872654337e-05 | 319.5568811248402 | 4.26961038426e-36 |
| 318.97926634768737 | 7.26035233945e-17 | 318.97926634768737 | 1.78012961664e-05 | 318.97926634768737 | 2.34630305224e-36 |
| 318.40373593716834 | 4.73807642791e-17 | 318.40373593716834 | 1.35226285851e-05 | 318.40373593716834 | 1.28681844704e-36 |
| 317.8302786312109 | 3.08591344777e-17 | 317.8302786312109 | 1.02519841669e-05 | 317.8302786312109 | 7.04348774827e-37 |
| 317.2588832487309 | 2.00586977604e-17 | 317.2588832487309 | 7.75696883815e-06 | 317.2588832487309 | 3.84765001955e-37 |
| 316.6895386889053 | 1.3012449063e-17 | 316.6895386889053 | 5.85751594818e-06 | 316.6895386889053 | 2.0976869352e-37 |
| 316.1222339304531 | 8.42466546869e-18 | 316.1222339304531 | 4.41440551432e-06 | 316.1222339304531 | 1.14136118128e-37 |
| 315.55695803092453 | 5.44356735677e-18 | 315.55695803092453 | 3.32023088078e-06 | 315.55695803092453 | 6.19787531418e-38 |
| 314.99370012599746 | 3.5103615719e-18 | 314.99370012599746 | 2.49230768373e-06 | 314.99370012599746 | 3.35892174093e-38 |
| 314.432449428781 | 2.25921423792e-18 | 314.432449428781 | 1.86712047398e-06 | 314.432449428781 | 1.81674615069e-38 |
| 313.8731952291274 | 1.4511098669e-18 | 313.8731952291274 | 1.39598367339e-06 | 313.8731952291274 | 9.80676853506e-39 |
| 313.31592689295036 | 9.30208894593e-19 | 313.31592689295036 | 1.04165919022e-06 | 313.31592689295036 | 5.28317399873e-39 |
| 312.76063386155124 | 5.95111023019e-19 | 312.76063386155124 | 7.75725867249e-07 | 312.76063386155124 | 2.84054200858e-39 |
| 312.2073056509522 | 3.79973067807e-19 | 312.2073056509522 | 5.76538362594e-07 | 312.2073056509522 | 1.52421008771e-39 |
| 311.65593185123623 | 2.42127963783e-19 | 311.65593185123623 | 4.27647029845e-07 | 311.65593185123623 | 8.16254821795e-40 |
| 311.1065021258944 | 1.53983574927e-19 | 311.1065021258944 | 3.16577483647e-07 | 311.1065021258944 | 4.36258599563e-40 |
| 310.5590062111801 | 9.77329842991e-20 | 310.5590062111801 | 2.33890131677e-07 | 310.5590062111801 | 2.3270169945e-40 |
| 310.01343391546965 | 6.19077807933e-20 | 310.01343391546965 | 1.7245710325e-07 | 310.01343391546965 | 1.23877497122e-40 |
| 309.4697751186301 | 3.91369183086e-20 | 309.4697751186301 | 1.26907587593e-07 | 309.4697751186301 | 6.581465395e-41 |
| 308.9280197713932 | 2.46925151589e-20 | 308.9280197713932 | 9.3203324406e-08 | 308.9280197713932 | 3.4897160845e-41 |
| 308.3881578947368 | 1.55482439489e-20 | 308.3881578947368 | 6.83144428511e-08 | 308.3881578947368 | 1.84669384276e-41 |
| 307.8501795792714 | 9.77090243082e-21 | 307.8501795792714 | 4.99724878709e-08 | 307.8501795792714 | 9.75297296003e-42 |
| 307.31407498463426 | 6.12809262193e-21 | 307.31407498463426 | 3.64826792315e-08 | 307.31407498463426 | 5.14063125125e-42 |
| 306.77983433888943 | 3.83577636182e-21 | 306.77983433888943 | 2.65815187642e-08 | 306.77983433888943 | 2.70416505972e-42 |
| 306.2474479379338 | 2.39617509374e-21 | 306.2474479379338 | 1.9329034919e-08 | 306.2474479379338 | 1.41966950914e-42 |
| 305.7169061449098 | 1.49389864893e-21 | 305.7169061449098 | 1.40274220189e-08 | 305.7169061449098 | 7.43838453263e-43 |
| 305.1881993896236 | 9.29524909965e-22 | 305.1881993896236 | 1.01597464209e-08 | 305.1881993896236 | 3.88962120915e-43 |
| 304.6613181679699 | 5.77215845234e-22 | 304.6613181679699 | 7.34387348931e-09 | 304.6613181679699 | 2.02989375015e-43 |
| 304.1362530413625 | 3.57727864211e-22 | 304.1362530413625 | 5.29791295001e-09 | 304.1362530413625 | 1.05724737665e-43 |
| 303.61299463617036 | 2.21260866171e-22 | 303.61299463617036 | 3.81436072893e-09 | 303.61299463617036 | 5.49562687865e-44 |
| 303.09153364316023 | 1.36582091916e-22 | 303.09153364316023 | 2.74079163228e-09 | 303.09153364316023 | 2.85098659094e-44 |
| 302.571860816944 | 8.41434327146e-23 | 302.571860816944 | 1.96547529782e-09 | 302.571860816944 | 1.47608179305e-44 |
| 302.0539669754329 | 5.17349464896e-23 | 302.0539669754329 | 1.40668375862e-09 | 302.0539669754329 | 7.62716224989e-45 |
| 301.5378429992964 | 3.17457137764e-23 | 301.5378429992964 | 1.00476076838e-09 | 301.5378429992964 | 3.93326184968e-45 |
| 301.02347983142687 | 1.94412194095e-23 | 301.02347983142687 | 7.16252545703e-10 | 301.02347983142687 | 2.02432410861e-45 |
| 300.5108684764098 | 1.18822646303e-23 | 300.5108684764098 | 5.09573694707e-10 | 300.5108684764098 | 1.03978739411e-45 |

| **GON1-Al3** | | **GON1-Al4** | | **GON1-Al5** | |
| --- | --- | --- | --- | --- | --- |
| Wavelength (nm) | Abs | Wavelength (nm) | Abs | Wavelength (nm) | Abs |
| 2000.0 | 3.10503483183 | 2000.0 | 1.26747811361e-16 | 2000.0 | 0.0669422447233 |
| 1977.5873434410018 | 3.63535609815 | 1977.5873434410018 | 1.92018825283e-16 | 1977.5873434410018 | 0.0808796299244 |
| 1955.671447196871 | 4.2478312101 | 1955.671447196871 | 2.90325017646e-16 | 1955.671447196871 | 0.0975363250368 |
| 1934.2359767891683 | 4.9536744841 | 1934.2359767891683 | 4.38089078812e-16 | 1934.2359767891683 | 0.11740418767 |
| 1913.265306122449 | 5.76537700423 | 1913.265306122449 | 6.59747427241e-16 | 1913.265306122449 | 0.141056275209 |
| 1892.7444794952683 | 6.69681204189 | 1892.7444794952683 | 9.91585795577e-16 | 1892.7444794952683 | 0.169158783998 |
| 1872.6591760299625 | 7.76334315111 | 1872.6591760299625 | 1.48737405446e-15 | 1872.6591760299625 | 0.202484484854 |
| 1852.9956763434218 | 8.98193400455 | 1852.9956763434218 | 2.22662673331e-15 | 1852.9956763434218 | 0.241927799522 |
| 1833.7408312958437 | 10.3712588589 | 1833.7408312958437 | 3.3266870647e-15 | 1833.7408312958437 | 0.288521669969 |
| 1814.8820326678765 | 11.9518123533 | 1814.8820326678765 | 4.96036681073e-15 | 1814.8820326678765 | 0.34345637885 |
| 1796.4071856287424 | 13.7460171501 | 1796.4071856287424 | 7.38164150469e-15 | 1796.4071856287424 | 0.408100485002 |
| 1778.3046828689983 | 15.7783277331 | 1778.3046828689983 | 1.09630001233e-14 | 1778.3046828689983 | 0.48402404206 |
| 1760.5633802816901 | 18.0753284734 | 1760.5633802816901 | 1.62496197036e-14 | 1760.5633802816901 | 0.573024271073 |
| 1743.1725740848342 | 20.6658238826 | 1743.1725740848342 | 2.40377767256e-14 | 1743.1725740848342 | 0.677153858873 |
| 1726.1219792865363 | 23.5809187745 | 1726.1219792865363 | 3.54880964967e-14 | 1726.1219792865363 | 0.798752052786 |
| 1709.4017094017095 | 26.8540858815 | 1709.4017094017095 | 5.22887701047e-14 | 1709.4017094017095 | 0.940478718543 |
| 1693.002257336343 | 30.5212183001 | 1693.002257336343 | 7.68902833359e-14 | 1693.002257336343 | 1.10535152171 |
| 1676.9144773616547 | 34.6206639993 | 1676.9144773616547 | 1.12842268597e-13 | 1676.9144773616547 | 1.29678638318 |
| 1661.1295681063123 | 39.1932395033 | 1661.1295681063123 | 1.65275876543e-13 | 1661.1295681063123 | 1.51864134598 |
| 1645.6390565002741 | 44.2822197742 | 1645.6390565002741 | 2.4159305331e-13 | 1645.6390565002741 | 1.77526397319 |
| 1630.4347826086955 | 49.9333012707 | 1630.4347826086955 | 3.52449362207e-13 | 1630.4347826086955 | 2.0715423751 |
| 1615.5088852988692 | 56.194535155 | 1615.5088852988692 | 5.13152357492e-13 | 1615.5088852988692 | 2.41295993739 |
| 1600.8537886872998 | 63.1162276625 | 1600.8537886872998 | 7.45646941685e-13 | 1600.8537886872998 | 2.80565379041 |
| 1586.4621893178212 | 70.7508047523 | 1586.4621893178212 | 1.08132804381e-12 | 1586.4621893178212 | 3.25647702272 |
| 1572.3270440251572 | 79.1526383153 | 1572.3270440251572 | 1.56501680342e-12 | 1572.3270440251572 | 3.77306459937 |
| 1558.4415584415583 | 88.3778314446 | 1558.4415584415583 | 2.26056946185e-12 | 1558.4415584415583 | 4.36390289672 |
| 1544.799176107106 | 98.4839605654 | 1544.799176107106 | 3.25877233639e-12 | 1544.799176107106 | 5.03840271051 |
| 1531.3935681470139 | 109.529772591 | 1531.3935681470139 | 4.68843067261e-12 | 1531.3935681470139 | 5.80697553289 |
| 1518.2186234817814 | 121.574835712 | 1518.2186234817814 | 6.73190981315e-12 | 1518.2186234817814 | 6.68111282633 |
| 1505.2684395383842 | 134.679142927 | 1505.2684395383842 | 9.64686926096e-12 | 1505.2684395383842 | 7.67346794814 |
| 1492.5373134328358 | 148.902668034 | 1492.5373134328358 | 1.37965916253e-11 | 1492.5373134328358 | 8.79794029934 |
| 1480.0197335964478 | 164.304874422 | 1480.0197335964478 | 1.96922136631e-11 | 1480.0197335964478 | 10.0697611848 |
| 1467.7103718199608 | 180.944177744 | 1467.7103718199608 | 2.80514026514e-11 | 1467.7103718199608 | 11.5055807796 |
| 1455.604075691412 | 198.877364327 | 1455.604075691412 | 3.98797050886e-11 | 1455.604075691412 | 13.1235554994 |
| 1443.6958614051973 | 218.158967982 | 1443.6958614051973 | 5.65830830249e-11 | 1443.6958614051973 | 14.9434349697 |
| 1431.9809069212408 | 238.840608763 | 1431.9809069212408 | 8.01232559187e-11 | 1431.9809069212408 | 16.9866476849 |
| 1420.4545454545455 | 260.970298102 | 1420.4545454545455 | 1.13231662642e-10 | 1420.4545454545455 | 19.2763843356 |
| 1409.1122592766555 | 284.591715648 | 1409.1122592766555 | 1.59703521549e-10 | 1409.1122592766555 | 21.8376776766 |
| 1397.9496738117427 | 309.743464056 | 1397.9496738117427 | 2.24801090755e-10 | 1397.9496738117427 | 24.6974776928 |
| 1386.9625520110958 | 336.458308825 | 1386.9625520110958 | 3.15805470506e-10 | 1386.9625520110958 | 27.8847207108 |
| 1376.1467889908256 | 364.762411167 | 1376.1467889908256 | 4.42770009256e-10 | 1376.1467889908256 | 31.4303909979 |
| 1365.4984069185252 | 394.674562627 | 1365.4984069185252 | 6.1954671349e-10 | 1365.4984069185252 | 35.3675732835 |
| 1355.0135501355014 | 426.205430942 | 1355.0135501355014 | 8.65181514472e-10 | 1355.0135501355014 | 39.7314945426 |
| 1344.688480502017 | 459.356827185 | 1344.688480502017 | 1.20580677342e-09 | 1344.688480502017 | 44.5595532925 |
| 1334.5195729537365 | 494.121004791 | 1334.5195729537365 | 1.67720257222e-09 | 1334.5195729537365 | 49.8913345728 |
| 1324.5033112582782 | 530.480001399 | 1324.5033112582782 | 2.32825547951e-09 | 1324.5033112582782 | 55.7686087184 |
| 1314.6362839614374 | 568.405034671 | 1314.6362839614374 | 3.22561894366e-09 | 1314.6362839614374 | 62.2353119817 |
| 1304.9151805132665 | 607.85596331 | 1304.9151805132665 | 4.45997887449e-09 | 1304.9151805132665 | 69.3375070343 |
| 1295.3367875647668 | 648.780824366 | 1295.3367875647668 | 6.15445861388e-09 | 1295.3367875647668 | 77.1233213639 |
| 1285.8979854264894 | 691.115457604 | 1285.8979854264894 | 8.47586875019e-09 | 1285.8979854264894 | 85.6428616047 |
| 1276.5957446808509 | 734.783227204 | 1276.5957446808509 | 1.1649731175e-08 | 1276.5957446808509 | 94.9481018731 |
| 1267.427122940431 | 779.694850348 | 1267.427122940431 | 1.59802989002e-08 | 1267.427122940431 | 105.09274426 |
| 1258.3892617449665 | 825.74834135 | 1258.3892617449665 | 2.18771732535e-08 | 1258.3892617449665 | 116.132049733 |
| 1249.4793835901708 | 872.829078869 | 1249.4793835901708 | 2.98906134137e-08 | 1249.4793835901708 | 128.122637829 |
| 1240.6947890818858 | 920.81000246 | 1240.6947890818858 | 4.07582719695e-08 | 1240.6947890818858 | 141.122253729 |
| 1232.0328542094455 | 969.55194326 | 1232.0328542094455 | 5.54669150311e-08 | 1232.0328542094455 | 155.189501463 |
| 1223.4910277324632 | 1018.90409197 | 1223.4910277324632 | 7.53337479475e-08 | 1223.4910277324632 | 170.383542318 |
| 1215.0668286755772 | 1068.70460552 | 1215.0668286755772 | 1.02113332871e-07 | 1215.0668286755772 | 186.763757754 |
| 1206.7578439259853 | 1118.78135206 | 1206.7578439259853 | 1.38137834001e-07 | 1206.7578439259853 | 204.389376523 |
| 1198.5617259288852 | 1168.95279166 | 1198.5617259288852 | 1.8650056281e-07 | 1198.5617259288852 | 223.319066031 |
| 1190.4761904761904 | 1219.02898855 | 1190.4761904761904 | 2.51295646972e-07 | 1190.4761904761904 | 243.61048843 |
| 1182.4990145841543 | 1268.81274828 | 1182.4990145841543 | 3.37930272786e-07 | 1182.4990145841543 | 265.319822358 |
| 1174.6280344557556 | 1318.1008714 | 1174.6280344557556 | 4.53530549919e-07 | 1174.6280344557556 | 288.501251785 |
| 1166.8611435239206 | 1366.68551337 | 1166.8611435239206 | 6.07467865069e-07 | 1166.8611435239206 | 313.206423899 |
| 1159.19629057187 | 1414.35563831 | 1159.19629057187 | 8.12039931203e-07 | 1159.19629057187 | 339.483878573 |
| 1151.6314779270633 | 1460.89855269 | 1151.6314779270633 | 1.08334997485e-06 | 1151.6314779270633 | 367.378452512 |
| 1144.1647597254005 | 1506.1015035 | 1144.1647597254005 | 1.44243909421e-06 | 1144.1647597254005 | 396.930661762 |
| 1136.794240242516 | 1549.75332387 | 1136.794240242516 | 1.91674132642e-06 | 1136.794240242516 | 428.176066909 |
| 1129.5180722891566 | 1591.64610821 | 1129.5180722891566 | 2.54194906701e-06 | 1129.5180722891566 | 461.144625862 |
| 1122.334455667789 | 1631.5768979 | 1122.334455667789 | 3.36439901972e-06 | 1122.334455667789 | 495.860039727 |
| 1115.2416356877322 | 1669.34935813 | 1115.2416356877322 | 4.44411682713e-06 | 1115.2416356877322 | 532.339097863 |
| 1108.2379017362393 | 1704.77542593 | 1108.2379017362393 | 5.85869315676e-06 | 1108.2379017362393 | 570.59102874 |
| 1101.3215859030836 | 1737.67690971 | 1101.3215859030836 | 7.70820675603e-06 | 1101.3215859030836 | 610.616863713 |
| 1094.4910616563297 | 1767.88702067 | 1094.4910616563297 | 1.01214625933e-05 | 1094.4910616563297 | 652.408821319 |
| 1087.7447425670775 | 1795.25181739 | 1087.7447425670775 | 1.3263877766e-05 | 1087.7447425670775 | 695.949720005 |
| 1081.081081081081 | 1819.6315455 | 1081.081081081081 | 1.73474268668e-05 | 1081.081081081081 | 741.21242757 |
| 1074.4985673352435 | 1840.90185571 | 1074.4985673352435 | 2.26431549123e-05 | 1074.4985673352435 | 788.159355758 |
| 1067.995728017088 | 1858.95488518 | 1067.995728017088 | 2.94968832329e-05 | 1067.995728017088 | 836.742008562 |
| 1061.5711252653928 | 1873.70018846 | 1061.5711252653928 | 3.83488760067e-05 | 1061.5711252653928 | 886.900592778 |
| 1055.2233556102708 | 1885.06550682 | 1055.2233556102708 | 4.97584071871e-05 | 1055.2233556102708 | 938.563699231 |
| 1048.951048951049 | 1892.9973664 | 1048.951048951049 | 6.4434375033e-05 | 1048.951048951049 | 991.648062793 |
| 1042.752867570386 | 1897.46149823 | 1042.752867570386 | 8.32733607995e-05 | 1042.752867570386 | 1046.05840895 |
| 1036.6275051831374 | 1898.44307566 | 1036.6275051831374 | 0.000107406826919 | 1036.6275051831374 | 1101.68739407 |
| 1030.5736860185502 | 1895.94676678 | 1030.5736860185502 | 0.000138259506841 | 1030.5736860185502 | 1158.41564597 |
| 1024.5901639344263 | 1889.99660257 | 1024.5901639344263 | 0.000177621463535 | 1024.5901639344263 | 1216.11191025 |
| 1018.6757215619693 | 1880.63566333 | 1018.6757215619693 | 0.000227736797783 | 1018.6757215619693 | 1274.63330735 |
| 1012.829169480081 | 1867.92558895 | 1012.829169480081 | 0.000291412583592 | 1012.829169480081 | 1333.82570377 |
| 1007.0493454179255 | 1851.94592049 | 1007.0493454179255 | 0.000372152310834 | 1007.0493454179255 | 1393.52419977 |
| 1001.3351134846461 | 1832.79328279 | 1001.3351134846461 | 0.000474318940666 | 1001.3351134846461 | 1453.55373484 |
| 995.6853634251576 | 1810.58042006 | 995.6853634251576 | 0.00060333364547 | 995.6853634251576 | 1513.72981027 |
| 990.0990099009902 | 1785.43509778 | 990.0990099009902 | 0.000765917431944 | 990.0990099009902 | 1573.85932721 |
| 984.5749917952082 | 1757.49888606 | 984.5749917952082 | 0.000970384155325 | 984.5749917952082 | 1633.74153656 |
| 979.1122715404699 | 1726.92584072 | 979.1122715404699 | 0.00122699494856 | 979.1122715404699 | 1693.16909587 |
| 973.7098344693281 | 1693.8810995 | 973.7098344693281 | 0.00154838583813 | 973.7098344693281 | 1751.92922657 |
| 968.3666881859263 | 1658.5394112 | 968.3666881859263 | 0.00195008232601 | 968.3666881859263 | 1809.80496335 |
| 963.0818619582664 | 1621.08361633 | 963.0818619582664 | 0.00245111701358 | 963.0818619582664 | 1866.57648612 |
| 957.8544061302682 | 1581.70309767 | 957.8544061302682 | 0.00307476895943 | 957.8544061302682 | 1922.02252332 |
| 952.6833915528738 | 1540.59221909 | 952.6833915528738 | 0.00384944642964 | 952.6833915528738 | 1975.92181428 |
| 947.5679090334806 | 1497.94877051 | 947.5679090334806 | 0.00480973804848 | 947.5679090334806 | 2028.05461685 |
| 942.5070688030161 | 1453.97243611 | 942.5070688030161 | 0.00599766112099 | 942.5070688030161 | 2078.20424584 |
| 937.4999999999999 | 1408.86330213 | 937.4999999999999 | 0.00746414010719 | 937.4999999999999 | 2126.15862654 |
| 932.5458501709667 | 1362.82041916 | 932.5458501709667 | 0.00927075290888 | 932.5458501709667 | 2171.71184728 |
| 927.643784786642 | 1316.04043288 | 927.643784786642 | 0.0114917878088 | 927.643784786642 | 2214.66569448 |
| 922.7929867733004 | 1268.71629526 | 922.7929867733004 | 0.0142166595992 | 922.7929867733004 | 2254.83115325 |
| 917.9926560587514 | 1221.03606708 | 917.9926560587514 | 0.0175527396665 | 917.9926560587514 | 2292.02985699 |
| 913.2420091324201 | 1173.18182065 | 913.2420091324201 | 0.0216286615623 | 913.2420091324201 | 2326.09546943 |
| 908.5402786190186 | 1125.32864989 | 908.5402786190186 | 0.0265981708917 | 908.5402786190186 | 2356.87498338 |
| 903.8867128653209 | 1077.64379341 | 903.8867128653209 | 0.0326445961573 | 903.8867128653209 | 2384.22992103 |
| 899.2805755395683 | 1030.28587413 | 899.2805755395683 | 0.0399860254905 | 899.2805755395683 | 2408.03742181 |
| 894.7211452430658 | 983.404257548 | 894.7211452430658 | 0.0488812829172 | 894.7211452430658 | 2428.19120517 |
| 890.2077151335311 | 937.138528936 | 890.2077151335311 | 0.0596368068757 | 890.2077151335311 | 2444.60239682 |
| 885.7395925597874 | 891.61808834 | 885.7395925597874 | 0.0726145430314 | 885.7395925597874 | 2457.20020907 |
| 881.316098707403 | 846.96186066 | 881.316098707403 | 0.0882409728861 | 881.316098707403 | 2465.93246722 |
| 876.9365682548962 | 803.278116864 | 876.9365682548962 | 0.107017409106 | 876.9365682548962 | 2470.76597632 |
| 872.6003490401396 | 760.66440118 | 872.6003490401396 | 0.129531697697 | 872.6003490401396 | 2471.68672438 |
| 868.3068017366135 | 719.207558074 | 868.3068017366135 | 0.156471475913 | 868.3068017366135 | 2468.69992031 |
| 864.0552995391705 | 678.98385195 | 864.0552995391705 | 0.18863914283 | 864.0552995391705 | 2461.82986701 |
| 859.8452278589854 | 640.059171803 | 859.8452278589854 | 0.226968706516 | 859.8452278589854 | 2451.11967188 |
| 855.6759840273816 | 602.489312506 | 855.6759840273816 | 0.27254467739 | 855.6759840273816 | 2436.63079951 |
| 851.5469770082316 | 566.320324001 | 851.5469770082316 | 0.3266231812 | 851.5469770082316 | 2418.4424728 |
| 847.457627118644 | 531.588919446 | 847.457627118644 | 0.390655466704 | 847.457627118644 | 2396.65093091 |
| 843.4073657576608 | 498.322933253 | 843.4073657576608 | 0.4663139821 | 843.4073657576608 | 2371.36855393 |
| 839.3956351426972 | 466.541820011 | 839.3956351426972 | 0.555521189982 | 839.3956351426972 | 2342.72286592 |
| 835.421888053467 | 436.25718544 | 835.421888053467 | 0.660481282559 | 835.421888053467 | 2310.85542888 |
| 831.4855875831485 | 407.473340781 | 831.4855875831485 | 0.783714946534 | 831.4855875831485 | 2275.92064181 |
| 827.5862068965516 | 380.187872439 | 827.5862068965516 | 0.928097309679 | 827.5862068965516 | 2238.08445943 |
| 823.7232289950576 | 354.392219111 | 823.7232289950576 | 1.09689917834 | 823.7232289950576 | 2197.523046 |
| 819.8961464881114 | 330.072249185 | 819.8961464881114 | 1.29383164602 | 819.8961464881114 | 2154.42138006 |
| 816.1044613710554 | 307.208831764 | 816.1044613710554 | 1.52309411761 | 816.1044613710554 | 2108.97182584 |
| 812.3476848090983 | 285.778395311 | 812.3476848090983 | 1.78942575059 | 812.3476848090983 | 2061.37268724 |
| 808.6253369272237 | 265.753468553 | 808.6253369272237 | 2.09816026419 | 808.6253369272237 | 2011.82675973 |
| 804.9369466058491 | 247.103198974 | 804.9369466058491 | 2.45528400827 | 804.9369466058491 | 1960.53989506 |
| 801.2820512820513 | 229.793844901 | 801.2820512820513 | 2.86749711672 | 801.2820512820513 | 1907.71959301 |
| 797.6601967561818 | 213.789237866 | 797.6601967561818 | 3.34227749421 | 797.6601967561818 | 1853.57363323 |
| 794.0709370037056 | 199.051212625 | 794.0709370037056 | 3.88794730084 | 794.0709370037056 | 1798.3087593 |
| 790.5138339920949 | 185.540002828 | 790.5138339920949 | 4.51374150637 | 790.5138339920949 | 1742.12942617 |
| 786.9884575026232 | 173.214600985 | 786.9884575026232 | 5.22987798536 | 786.9884575026232 | 1685.23662014 |
| 783.4943849569078 | 162.033081978 | 783.4943849569078 | 6.04762851614 | 783.4943849569078 | 1627.82676012 |
| 780.0312012480499 | 151.952889902 | 780.0312012480499 | 6.97938993276 | 780.0312012480499 | 1570.09068648 |
| 776.598498576236 | 142.931088562 | 776.598498576236 | 8.03875455924 | 776.598498576236 | 1512.21274331 |
| 773.1958762886597 | 134.924576417 | 773.1958762886597 | 9.24057893262 | 773.1958762886597 | 1454.36995763 |
| 769.8229407236336 | 127.890267209 | 769.8229407236336 | 10.6010496968 | 769.8229407236336 | 1396.73131836 |
| 766.4793050587633 | 121.7852379 | 766.4793050587633 | 12.1377454244 | 766.4793050587633 | 1339.4571561 |
| 763.1645891630628 | 116.566845863 | 763.1645891630628 | 13.8696930033 | 763.1645891630628 | 1282.69862371 |
| 759.8784194528876 | 112.192817604 | 759.8784194528876 | 15.8174171089 | 759.8784194528876 | 1226.59727631 |
| 756.6204287515762 | 108.621311509 | 756.6204287515762 | 18.0029811764 | 756.6204287515762 | 1171.28474849 |
| 753.390256152687 | 105.810957321 | 753.390256152687 | 20.4500181951 | 753.390256152687 | 1116.88252537 |
| 750.1875468867216 | 103.720875214 | 750.1875468867216 | 23.1837495673 | 750.1875468867216 | 1063.50180329 |
| 747.011952191235 | 102.310677421 | 747.011952191235 | 26.2309902182 | 747.011952191235 | 1011.24343535 |
| 743.86312918423 | 101.540455466 | 743.86312918423 | 29.62013811 | 743.86312918423 | 960.197956332 |
| 740.7407407407408 | 101.370756046 | 740.7407407407408 | 33.3811463078 | 740.7407407407408 | 910.445681017 |
| 737.6444553725104 | 101.762548593 | 737.6444553725104 | 37.5454757711 | 737.6444553725104 | 862.056869688 |
| 734.5739471106758 | 102.677187513 | 734.5739471106758 | 42.1460271107 | 734.5739471106758 | 815.091954319 |
| 731.528895391368 | 104.076371954 | 731.528895391368 | 47.2170496503 | 731.528895391368 | 769.601818845 |
| 728.5089849441475 | 105.922105864 | 728.5089849441475 | 52.7940262805 | 728.5089849441475 | 725.628126895 |
| 725.5139056831922 | 108.176660902 | 725.5139056831922 | 58.9135327829 | 725.5139056831922 | 683.203690448 |
| 722.543352601156 | 110.802544582 | 722.543352601156 | 65.6130705429 | 722.543352601156 | 642.352873022 |
| 719.5970256656271 | 113.762475792 | 719.5970256656271 | 72.9308718567 | 719.5970256656271 | 603.092021249 |
| 716.6746297181079 | 117.019369566 | 716.6746297181079 | 80.9056773777 | 716.6746297181079 | 565.429918934 |
| 713.7758743754462 | 120.536332744 | 713.7758743754462 | 89.5764856347 | 713.7758743754462 | 529.368258057 |
| 710.9004739336492 | 124.27667181 | 710.9004739336492 | 98.982274987 | 710.9004739336492 | 494.902121517 |
| 708.0481472740146 | 128.203913928 | 708.0481472740146 | 109.161698865 | 708.0481472740146 | 462.020472806 |
| 705.2186177715091 | 132.281841851 | 705.2186177715091 | 120.152755661 | 705.2186177715091 | 430.706648214 |
| 702.4116132053383 | 136.474543068 | 702.4116132053383 | 131.992435187 | 702.4116132053383 | 400.938847552 |
| 699.6268656716418 | 140.746473203 | 699.6268656716418 | 144.716344204 | 699.6268656716418 | 372.690619802 |
| 696.8641114982578 | 145.062533377 | 696.8641114982578 | 158.358314129 | 696.8641114982578 | 345.93134051 |
| 694.1230911614992 | 149.388160921 | 694.1230911614992 | 172.949994624 | 694.1230911614992 | 320.626678103 |
| 691.4035492048858 | 153.689432501 | 691.4035492048858 | 188.520437396 | 691.4035492048858 | 296.739046705 |
| 688.7052341597796 | 157.933178476 | 688.7052341597796 | 205.095675124 | 688.7052341597796 | 274.228043373 |
| 686.027898467871 | 162.087107003 | 686.027898467871 | 222.698301 | 686.027898467871 | 253.050868005 |
| 683.371298405467 | 166.119936186 | 683.371298405467 | 241.347054899 | 683.371298405467 | 233.16272447 |
| 680.7351940095303 | 170.001532361 | 680.7351940095303 | 261.056422653 | 680.7351940095303 | 214.517201825 |
| 678.1193490054249 | 173.703052437 | 678.1193490054249 | 281.836255346 | 678.1193490054249 | 197.066634698 |
| 675.5235307363206 | 177.197088069 | 675.5235307363206 | 303.691415793 | 675.5235307363206 | 180.76244221 |
| 672.9475100942127 | 180.45780936 | 672.9475100942127 | 326.621459668 | 672.9475100942127 | 165.555444968 |
| 670.3910614525139 | 183.461105727 | 670.3910614525139 | 350.62035877 | 670.3910614525139 | 151.3961599 |
| 667.8539626001781 | 186.184721557 | 667.8539626001781 | 375.676273959 | 667.8539626001781 | 138.235072851 |
| 665.335994677312 | 188.608384319 | 665.335994677312 | 401.771385083 | 665.335994677312 | 126.02288903 |
| 662.8369421122403 | 190.713922859 | 662.8369421122403 | 428.881784955 | 662.8369421122403 | 114.710761519 |
| 660.3565925599823 | 192.485373746 | 660.3565925599823 | 456.977443934 | 660.3565925599823 | 104.250498208 |
| 657.8947368421053 | 193.909073656 | 657.8947368421053 | 486.022251091 | 657.8947368421053 | 94.5947475987 |
| 655.4511688879178 | 194.973736022 | 655.4511688879178 | 515.974137137 | 655.4511688879178 | 85.6971640368 |
| 653.0256856769699 | 195.670510347 | 653.0256856769699 | 546.78528336 | 653.0256856769699 | 77.5125530165 |
| 650.6180871828237 | 195.993022881 | 650.6180871828237 | 578.402419737 | 650.6180871828237 | 69.9969972672 |
| 648.2281763180639 | 195.937397599 | 648.2281763180639 | 610.767214156 | 648.2281763180639 | 63.1079644087 |
| 645.8557588805166 | 195.502256739 | 645.8557588805166 | 643.816753327 | 645.8557588805166 | 56.8043970114 |
| 643.5006435006435 | 194.688700465 | 643.5006435006435 | 677.484114474 | 643.5006435006435 | 51.0467859481 |
| 641.1626415900834 | 193.500265507 | 641.1626415900834 | 711.699025343 | 641.1626415900834 | 45.7972279655 |
| 638.8415672913118 | 191.942863 | 638.8415672913118 | 746.388608422 | 638.8415672913118 | 41.0194684329 |
| 636.5372374283895 | 190.024695998 | 636.5372374283895 | 781.478203574 | 636.5372374283895 | 36.6789302524 |
| 634.2494714587738 | 187.756157489 | 634.2494714587738 | 816.892261599 | 634.2494714587738 | 32.7427299321 |
| 631.9780914261638 | 185.149710004 | 631.9780914261638 | 852.555299537 | 631.9780914261638 | 29.1796818331 |
| 629.7229219143577 | 182.219748173 | 629.7229219143577 | 888.392906933 | 629.7229219143577 | 25.9602916038 |
| 627.4837900020916 | 178.982445842 | 627.4837900020916 | 924.332790679 | 627.4837900020916 | 23.0567398134 |
| 625.2605252188412 | 175.455589561 | 625.2605252188412 | 960.305844688 | 625.2605252188412 | 20.442856783 |
| 623.0529595015576 | 171.658400438 | 623.0529595015576 | 996.247229326 | 623.0529595015576 | 18.0940895968 |
| 620.8609271523178 | 167.611346505 | 620.8609271523178 | 1032.0974445 | 620.8609271523178 | 15.9874622554 |
| 618.6842647968654 | 163.335947836 | 618.6842647968654 | 1067.80337941 | 618.6842647968654 | 14.1015298993 |
| 616.5228113440197 | 158.854576731 | 616.5228113440197 | 1103.31932137 | 616.5228113440197 | 12.4163280013 |
| 614.3764079459348 | 154.190255318 | 614.3764079459348 | 1138.6079058 | 614.3764079459348 | 10.9133173856 |
| 612.2448979591836 | 149.3664529 | 612.2448979591836 | 1173.6409895 | 612.2448979591836 | 9.57532588716 |
| 610.1281269066504 | 144.406885339 | 610.1281269066504 | 1208.40042943 | 610.1281269066504 | 8.38648742173 |
| 608.0259424402108 | 139.335318679 | 608.0259424402108 | 1242.87875021 | 608.0259424402108 | 7.33217918192 |
| 605.9381943041809 | 134.175379108 | 605.9381943041809 | 1277.07968405 | 605.9381943041809 | 6.39895762753 |
| 603.864734299517 | 128.950371196 | 603.864734299517 | 1311.01856859 | 603.864734299517 | 5.57449388106 |
| 601.8054162487462 | 123.683106188 | 601.8054162487462 | 1344.72258934 | 601.8054162487462 | 4.84750908559 |
| 599.7600959616153 | 118.395741934 | 599.7600959616153 | 1378.23085569 | 599.7600959616153 | 4.20771022656 |
| 597.7286312014345 | 113.10963582 | 597.7286312014345 | 1411.59430141 | 597.7286312014345 | 3.64572686373 |
| 595.7108816521048 | 107.845211854 | 595.7108816521048 | 1444.87540335 | 595.7108816521048 | 3.15304916497 |
| 593.7067088858104 | 102.621842797 | 593.7067088858104 | 1478.14771445 | 593.7067088858104 | 2.72196758058 |
| 591.7159763313609 | 97.4577480324 | 591.7159763313609 | 1511.4952103 | 591.7159763313609 | 2.34551444524 |
| 589.7385492431688 | 92.3699075833 | 589.7385492431688 | 1545.01145149 | 589.7385492431688 | 2.01740774577 |
| 587.7742946708463 | 87.3739924784 | 587.7742946708463 | 1578.7985671 | 587.7742946708463 | 1.7319972462 |
| 585.8230814294083 | 82.4843114366 | 585.8230814294083 | 1612.96606777 | 585.8230814294083 | 1.48421311805 |
| 583.8847800700661 | 77.7137736149 | 583.8847800700661 | 1647.62950004 | 583.8847800700661 | 1.2695171834 |
| 581.9592628516003 | 73.0738669713 | 581.9592628516003 | 1682.90895661 | 581.9592628516003 | 1.08385684087 |
| 580.046403712297 | 68.5746516067 | 580.046403712297 | 1718.92746011 | 580.046403712297 | 0.923621711249 |
| 578.1460782424359 | 64.2247672878 | 578.1460782424359 | 1755.80924054 | 578.1460782424359 | 0.785603008959 |
| 576.2581636573184 | 60.0314542097 | 576.2581636573184 | 1793.677929 | 576.2581636573184 | 0.666955618954 |
| 574.3825387708214 | 56.0005859356 | 574.3825387708214 | 1832.65469248 | 574.3825387708214 | 0.565162835256 |
| 572.5190839694656 | 52.1367133595 | 572.5190839694656 | 1872.85633597 | 572.5190839694656 | 0.478003697279 |
| 570.6676811869887 | 48.4431184588 | 570.6676811869887 | 1914.39339957 | 570.6676811869887 | 0.403522843306 |
| 568.8282138794084 | 44.9218765605 | 568.8282138794084 | 1957.36827908 | 568.8282138794084 | 0.340002786659 |
| 567.000567000567 | 41.5739258148 | 567.000567000567 | 2001.87339872 | 567.000567000567 | 0.285938509146 |
| 565.1846269781461 | 38.3991425658 | 565.1846269781461 | 2047.9894646 | 565.1846269781461 | 0.240014258011 |
| 563.3802816901408 | 35.3964213262 | 563.3802816901408 | 2095.78382681 | 563.3802816901408 | 0.201082426687 |
| 561.5874204417821 | 32.5637580944 | 561.5874204417821 | 2145.30897654 | 561.5874204417821 | 0.168144395849 |
| 559.8059339428997 | 29.8983358064 | 559.8059339428997 | 2196.60120324 | 559.8059339428997 | 0.14033320941 |
| 558.0357142857143 | 27.3966107763 | 558.0357142857143 | 2249.67943442 | 558.0357142857143 | 0.116897959973 |
| 556.2766549230483 | 25.05439906 | 556.2766549230483 | 2304.54427779 | 556.2766549230483 | 0.0971897595146 |
| 554.52865064695 | 22.8669617625 | 554.52865064695 | 2361.1772828 | 554.52865064695 | 0.080649173665 |
| 552.791597567717 | 20.8290884048 | 552.791597567717 | 2419.54043478 | 552.791597567717 | 0.0667950015088 |
| 551.0653930933137 | 18.9351775669 | 551.0653930933137 | 2479.57589131 | 551.0653930933137 | 0.0552142872773 |
| 549.3499359091741 | 17.1793141271 | 549.3499359091741 | 2541.20596641 | 549.3499359091741 | 0.0455534553714 |
| 547.645125958379 | 15.555342524 | 547.645125958379 | 2604.33336405 | 547.645125958379 | 0.0375104657405 |
| 545.950864422202 | 14.0569355694 | 545.950864422202 | 2668.84165834 | 545.950864422202 | 0.0308278925624 |
| 544.2670537010159 | 12.6776584462 | 544.2670537010159 | 2734.59601328 | 544.2670537010159 | 0.0252868353069 |
| 542.5935973955508 | 11.4110276193 | 542.5935973955508 | 2801.44413141 | 542.5935973955508 | 0.020701577496 |
| 540.9304002884962 | 10.2505644837 | 540.9304002884962 | 2869.21741624 | 540.9304002884962 | 0.0169149147093 |
| 539.2773683264425 | 9.18984365881 | 539.2773683264425 | 2937.73233003 | 539.2773683264425 | 0.0137940795291 |
| 537.6344086021505 | 8.22253591849 | 537.6344086021505 | 3006.79192525 | 537.6344086021505 | 0.0112271971137 |
| 536.0014293371448 | 7.34244581683 | 536.0014293371448 | 3076.1875249 | 536.0014293371448 | 0.00912021086971 |
| 534.3783398646241 | 6.54354413484 | 534.3783398646241 | 3145.70052485 | 534.3783398646241 | 0.00739422322622 |
| 532.7650506126798 | 5.81999532686 | 532.7650506126798 | 3215.10428919 | 532.7650506126798 | 0.00598320175461 |
| 531.1614730878186 | 5.16618019292 | 531.1614730878186 | 3284.16610849 | 531.1614730878186 | 0.0048320058134 |
| 529.5675198587819 | 4.57671404122 | 529.5675198587819 | 3352.64919018 | 529.5675198587819 | 0.00389469350843 |
| 527.9831045406547 | 4.04646063505 | 527.9831045406547 | 3420.31465015 | 527.9831045406547 | 0.00313307303922 |
| 526.4081417792595 | 3.57054224088 | 526.4081417792595 | 3486.92347524 | 526.4081417792595 | 0.00251546645228 |
| 524.8425472358292 | 3.14434610954 | 524.8425472358292 | 3552.23842762 | 524.8425472358292 | 0.00201565744617 |
| 523.2862375719518 | 2.76352773077 | 523.2862375719518 | 3616.02586355 | 523.2862375719518 | 0.00161199817924 |
| 521.7391304347826 | 2.42401120386 | 521.7391304347826 | 3678.05744163 | 521.7391304347826 | 0.00128665303232 |
| 520.2011444425177 | 2.12198706425 | 520.2011444425177 | 3738.11169819 | 520.2011444425177 | 0.00102495998898 |
| 518.6721991701245 | 1.85390789805 | 518.6721991701245 | 3795.97547092 | 518.6721991701245 | 0.000814892732894 |
| 517.1522151353215 | 1.61648206493 | 517.1522151353215 | 3851.44515522 | 517.1522151353215 | 0.000646608741188 |
| 515.6411137848057 | 1.40666583478 | 515.6411137848057 | 3904.32778178 | 515.6411137848057 | 0.000512070595479 |
| 514.1388174807198 | 1.22165422568 | 514.1388174807198 | 3954.44190771 | 514.1388174807198 | 0.000404729454155 |
| 512.6452494873547 | 1.05887081145 | 512.6452494873547 | 4001.61831798 | 512.6452494873547 | 0.000319261151377 |
| 511.1603339580848 | 0.915956745237 | 511.1603339580848 | 4045.70053766 | 511.1603339580848 | 0.000251346726682 |
| 509.683995922528 | 0.790759224244 | 509.683995922528 | 4086.54515964 | 509.683995922528 | 0.000197490362037 |
| 508.2161612739285 | 0.681319597577 | 508.2161612739285 | 4124.0219964 | 508.2161612739285 | 0.000154868727116 |
| 506.7567567567567 | 0.585861297153 | 506.7567567567567 | 4158.0140676 | 506.7567567567567 | 0.000121206624007 |
| 505.3057099545225 | 0.502777749271 | 505.3057099545225 | 4188.4174387 | 505.3057099545225 | 9.46745940902e-05 |
| 503.8629492777964 | 0.430620403102 | 503.8629492777964 | 4215.1409283 | 503.8629492777964 | 7.38048159789e-05 |
| 502.4284039524367 | 0.368086991899 | 502.4284039524367 | 4238.10570415 | 502.4284039524367 | 5.74221964767e-05 |
| 501.00200400801606 | 0.314010123418 | 501.00200400801606 | 4257.24478949 | 501.00200400801606 | 4.45880478538e-05 |
| 499.5836802664446 | 0.267346278055 | 499.5836802664446 | 4272.50250232 | 499.5836802664446 | 3.4554164555e-05 |
| 498.1733643307871 | 0.227165276661 | 498.1733643307871 | 4283.83385065 | 498.1733643307871 | 2.67254699643e-05 |
| 496.7709885742673 | 0.192640264902 | 496.7709885742673 | 4291.2039069 | 496.7709885742673 | 2.06297073024e-05 |
| 495.3764861294584 | 0.163038247522 | 495.3764861294584 | 4294.58718341 | 495.3764861294584 | 1.58929054633e-05 |
| 493.98979087765514 | 0.137711193827 | 493.98979087765514 | 4293.96703035 | 493.98979087765514 | 1.22195671026e-05 |
| 492.61083743842363 | 0.116087725172 | 492.61083743842363 | 4289.3350748 | 492.61083743842363 | 9.37670830696e-06 |
| 491.2395611593253 | 0.0976653861772 | 491.2395611593253 | 4280.69071821 | 491.2395611593253 | 7.18103170985e-06 |
| 489.8758981058131 | 0.0820034936517 | 489.8758981058131 | 4268.04070623 | 489.8758981058131 | 5.4886423621e-06 |
| 488.5197850512946 | 0.0687165507781 | 488.5197850512946 | 4251.39878214 | 488.5197850512946 | 4.18682181854e-06 |
| 487.17115946735953 | 0.057468208882 | 487.17115946735953 | 4230.78543191 | 487.17115946735953 | 3.18746405448e-06 |
| 485.82995951416996 | 0.0479657549384 | 485.82995951416996 | 4206.22772549 | 485.82995951416996 | 2.42184981435e-06 |
| 484.49612403100775 | 0.0399550998083 | 484.49612403100775 | 4177.75925525 | 484.49612403100775 | 1.83649624884e-06 |
| 483.16959252697694 | 0.0332162398987 | 483.16959252697694 | 4145.42016964 | 483.16959252697694 | 1.38986829362e-06 |
| 481.8503051718599 | 0.0275591634161 | 481.8503051718599 | 4109.25729622 | 481.8503051718599 | 1.04977894908e-06 |
| 480.5382027871216 | 0.0228201715329 | 480.5382027871216 | 4069.3243455 | 480.5382027871216 | 7.91338935207e-07 |
| 479.23322683706067 | 0.0188585845007 | 479.23322683706067 | 4025.68218417 | 479.23322683706067 | 5.95343382172e-07 |
| 477.9353194201051 | 0.0155538029487 | 477.9353194201051 | 3978.39916385 | 477.9353194201051 | 4.47005342149e-07 |
| 476.64442326024783 | 0.0128026951967 | 476.64442326024783 | 3927.55148928 | 476.64442326024783 | 3.34963861642e-07 |
| 475.3604816986214 | 0.0105172823309 | 475.3604816986214 | 3873.22360865 | 475.3604816986214 | 2.50508883479e-07 |
| 474.08343868520853 | 0.00862269395354 | 474.08343868520853 | 3815.50860742 | 474.08343868520853 | 1.86976973505e-07 |
| 472.8132387706856 | 0.00705536886594 | 472.8132387706856 | 3754.50858648 | 472.8132387706856 | 1.39281304589e-07 |
| 471.5498270983967 | 0.00576147643156 | 471.5498270983967 | 3690.33500556 | 471.5498270983967 | 1.03546905518e-07 |
| 470.29314939645707 | 0.00469553592895 | 470.29314939645707 | 3623.10897327 | 470.29314939645707 | 7.682824617e-08 |
| 469.04315196998124 | 0.00381921281864 | 469.04315196998124 | 3552.96146597 | 469.04315196998124 | 5.68910713202e-08 |
| 467.7997816934352 | 0.00310027246829 | 467.7997816934352 | 3480.03345939 | 467.7997816934352 | 4.20442499802e-08 |
| 466.5629860031104 | 0.00251167348482 | 466.5629860031104 | 3404.47595857 | 466.5629860031104 | 3.10104681387e-08 |
| 465.33271288971605 | 0.00203078436492 | 465.33271288971605 | 3326.44991404 | 465.33271288971605 | 2.28270171649e-08 |
| 464.10891089108907 | 0.00163870868073 | 464.10891089108907 | 3246.12601471 | 464.10891089108907 | 1.67698453215e-08 |
| 462.8915290850177 | 0.00131970545084 | 462.8915290850177 | 3163.68435057 | 462.8915290850177 | 1.22955488337e-08 |
| 461.68051708217905 | 0.00106069269932 | 461.68051708217905 | 3079.31394152 | 461.68051708217905 | 8.99716484133e-09 |
| 460.47582501918646 | 0.000850823470675 | 460.47582501918646 | 2993.21213131 | 460.47582501918646 | 6.57055811932e-09 |
| 459.2774035517452 | 0.00068112474273 | 459.2774035517452 | 2905.58384902 | 459.2774035517452 | 4.78892022186e-09 |
| 458.0852038479157 | 0.00054419076185 | 458.0852038479157 | 2816.64074304 | 458.0852038479157 | 3.48346647993e-09 |
| 456.89917758148033 | 0.000433923315298 | 456.89917758148033 | 2726.60019581 | 456.89917758148033 | 2.52885716412e-09 |
| 455.7192769254139 | 0.000345312357133 | 455.7192769254139 | 2635.68422973 | 455.7192769254139 | 1.83221120895e-09 |
| 454.54545454545456 | 0.000274251219252 | 454.54545454545456 | 2544.11831754 | 454.54545454545456 | 1.32484565664e-09 |
| 453.3776635937736 | 0.000217381372757 | 453.3776635937736 | 2452.130112 | 453.3776635937736 | 9.56078380719e-10 |
| 452.2158577027434 | 0.000171962361271 | 452.2158577027434 | 2359.94811197 | 452.2158577027434 | 6.8858906038e-10 |
| 451.05999097880016 | 0.000135763112436 | 451.05999097880016 | 2267.8002828 | 451.05999097880016 | 4.94954298024e-10 |
| 449.9100179964007 | 0.000106971351972 | 449.9100179964007 | 2175.91265032 | 449.9100179964007 | 3.55065432984e-10 |
| 448.7658937920718 | 8.4118301824e-05 | 448.7658937920718 | 2084.50788786 | 448.7658937920718 | 2.54208434707e-10 |
| 447.6275738585497 | 6.60162455121e-05 | 447.6275738585497 | 1993.80391619 | 447.6275738585497 | 1.81639268451e-10 |
| 446.49501413900873 | 5.17068950078e-05 | 446.49501413900873 | 1904.01253559 | 446.49501413900873 | 1.29529209783e-10 |
| 445.36817102137763 | 4.04187994857e-05 | 445.36817102137763 | 1815.33810897 | 445.36817102137763 | 9.2185758435e-11 |
| 444.247001332741 | 3.1532301739e-05 | 444.247001332741 | 1727.97631356 | 444.247001332741 | 6.54783998796e-11 |
| 443.13146233382565 | 2.45507775065e-05 | 443.13146233382565 | 1642.11297752 | 443.13146233382565 | 4.64162824942e-11 |
| 442.02151171357 | 1.90770905099e-05 | 442.02151171357 | 1557.92301622 | 442.02151171357 | 3.28383017525e-11 |
| 440.9171075837742 | 1.47943654647e-05 | 440.9171075837742 | 1475.56948076 | 440.9171075837742 | 2.31861707209e-11 |
| 439.8182084738308 | 1.1450326183e-05 | 439.8182084738308 | 1395.20272965 | 439.8182084738308 | 1.63386189193e-11 |
| 438.72477332553376 | 8.84456925942e-06 | 438.72477332553376 | 1316.95973195 | 438.72477332553376 | 1.14905178589e-11 |
| 437.636761487965 | 6.81824854875e-06 | 437.636761487965 | 1240.96350816 | 437.636761487965 | 8.06495181112e-12 |
| 436.5541327124563 | 5.24573422489e-06 | 436.5541327124563 | 1167.32271265 | 436.5541327124563 | 5.64939368906e-12 |
| 435.4768471476266 | 4.02788489138e-06 | 435.4768471476266 | 1096.13135926 | 435.4768471476266 | 3.94947862019e-12 |
| 434.4048653344918 | 3.08663396872e-06 | 434.4048653344918 | 1027.46868927 | 434.4048653344918 | 2.75559591498e-12 |
| 433.3381482016467 | 2.36064414755e-06 | 433.3381482016467 | 961.39917898 | 433.3381482016467 | 1.91879735903e-12 |
| 432.2766570605187 | 1.80182758429e-06 | 432.2766570605187 | 897.972681992 | 432.2766570605187 | 1.33346150865e-12 |
| 431.22035360069 | 1.37256604418e-06 | 431.22035360069 | 837.224699616 | 431.22035360069 | 9.2484643433e-13 |
| 430.1691998852882 | 1.04349550617e-06 | 430.1691998852882 | 779.176771032 | 430.1691998852882 | 6.40171770233e-13 |
| 429.1231583464454 | 7.91744816004e-07 | 429.1231583464454 | 723.836973608 | 429.1231583464454 | 4.42243177743e-13 |
| 428.0821917808219 | 5.9953865161e-07 | 428.0821917808219 | 671.200522523 | 428.0821917808219 | 3.04904248995e-13 |
| 427.0462633451957 | 4.53092066783e-07 | 427.0462633451957 | 621.250457987 | 427.0462633451957 | 2.09799043913e-13 |
| 426.01533655211585 | 3.41737818255e-07 | 426.01533655211585 | 573.958407667 | 426.01533655211585 | 1.44072538873e-13 |
| 424.9893752656184 | 2.57239075283e-07 | 424.9893752656184 | 529.285411549 | 424.9893752656184 | 9.87407847287e-14 |
| 423.96834369700395 | 1.93249398116e-07 | 423.96834369700395 | 487.182796285 | 423.96834369700395 | 6.7538215876e-14 |
| 422.9522064006767 | 1.44889419752e-07 | 422.9522064006767 | 447.593086136 | 422.9522064006767 | 4.61041716062e-14 |
| 421.9409282700422 | 1.08415782663e-07 | 421.9409282700422 | 410.45093789 | 421.9409282700422 | 3.14100391474e-14 |
| 420.93447453346425 | 8.09628259551e-08 | 420.93447453346425 | 375.684087617 | 420.93447453346425 | 2.13567091941e-14 |
| 419.9328107502799 | 6.03415026085e-08 | 419.9328107502799 | 343.214297726 | 419.9328107502799 | 1.44923154726e-14 |
| 418.93590280687056 | 4.48832085662e-08 | 418.93590280687056 | 312.958293581 | 418.93590280687056 | 9.81474003723e-15 |
| 417.94371691278906 | 3.33187717869e-08 | 417.94371691278906 | 284.828679828 | 417.94371691278906 | 6.63372382674e-15 |
| 416.9562195969423 | 2.46849007217e-08 | 416.9562195969423 | 258.734827561 | 416.9562195969423 | 4.47479894047e-15 |
| 415.97337770382694 | 1.8252026848e-08 | 415.97337770382694 | 234.583724523 | 415.97337770382694 | 3.01250106037e-15 |
| 414.99515838981876 | 1.34687758676e-08 | 414.99515838981876 | 212.280781629 | 414.99515838981876 | 2.02403688423e-15 |
| 414.0215291195142 | 9.91933290365e-09 | 414.0215291195142 | 191.730590219 | 414.0215291195142 | 1.35721033681e-15 |
| 413.0524576621231 | 7.29078210341e-09 | 413.0524576621231 | 172.837625565 | 413.0524576621231 | 9.08266733258e-16 |
| 412.08791208791206 | 5.34814390817e-09 | 412.08791208791206 | 155.506893236 | 412.08791208791206 | 6.06620608923e-16 |
| 411.1278607646978 | 3.91533893977e-09 | 411.1278607646978 | 139.644516007 | 411.1278607646978 | 4.04350895097e-16 |
| 410.17227235438884 | 2.86070421273e-09 | 410.17227235438884 | 125.15825995 | 410.17227235438884 | 2.6899062503e-16 |
| 409.22111580957574 | 2.0859978687e-09 | 409.22111580957574 | 111.957999319 | 409.22111580957574 | 1.7858844611e-16 |
| 408.2743603701687 | 1.51807097986e-09 | 408.2743603701687 | 99.9561206464 | 408.2743603701687 | 1.18333318721e-16 |
| 407.33197556008145 | 1.10257364933e-09 | 407.33197556008145 | 89.0678672797 | 407.33197556008145 | 7.82524953665e-17 |
| 406.39393118396094 | 7.99209159776e-10 | 406.39393118396094 | 79.2116261999 | 406.39393118396094 | 5.16448242788e-17 |
| 405.46019732396263 | 5.78163418344e-10 | 405.46019732396263 | 70.3091595963 | 405.46019732396263 | 3.40167535762e-17 |
| 404.53074433656957 | 4.17424637945e-10 | 404.53074433656957 | 62.2857841171 | 404.53074433656957 | 2.23612650201e-17 |
| 403.6055428494551 | 3.00775780075e-10 | 403.6055428494551 | 55.0705011189 | 403.6055428494551 | 1.46702431738e-17 |
| 402.68456375838923 | 2.16294241652e-10 | 402.68456375838923 | 48.5960815334 | 402.68456375838923 | 9.60540520099e-18 |
| 401.76777822418643 | 1.55233112754e-10 | 401.76777822418643 | 42.7991091788 | 401.76777822418643 | 6.27670179026e-18 |
| 400.85515766969536 | 1.11188811404e-10 | 400.85515766969536 | 37.619986477 | 400.85515766969536 | 4.09340507611e-18 |
| 399.9466737768297 | 7.94831595067e-11 | 399.9466737768297 | 33.0029065917 | 399.9466737768297 | 2.66425242591e-18 |
| 399.0422984836393 | 5.67056678637e-11 | 399.0422984836393 | 28.8957959941 | 399.0422984836393 | 1.73062681742e-18 |
| 398.14200398142003 | 4.03752412483e-11 | 398.14200398142003 | 25.2502313927 | 398.14200398142003 | 1.12193810147e-18 |
| 397.24576271186436 | 2.86906973215e-11 | 397.24576271186436 | 22.0213348429 | 397.24576271186436 | 7.25891597623e-19 |
| 396.3535473642489 | 2.03471876467e-11 | 396.3535473642489 | 19.1676506901 | 396.3535473642489 | 4.68718442487e-19 |
| 395.46533087266016 | 1.44014091719e-11 | 395.46533087266016 | 16.6510078 | 395.46533087266016 | 3.02057560576e-19 |
| 394.5810864132579 | 1.01728561206e-11 | 394.5810864132579 | 14.4363703039 | 394.5810864132579 | 1.9426956695e-19 |
| 393.7007874015748 | 7.17163416507e-12 | 393.7007874015748 | 12.4916798406 | 393.7007874015748 | 1.246973495e-19 |
| 392.82440748985204 | 5.04580735938e-12 | 392.82440748985204 | 10.7876920163 | 392.82440748985204 | 7.98816557902e-20 |
| 391.9519205644107 | 3.54307639977e-12 | 391.9519205644107 | 9.29780953492 | 391.9519205644107 | 5.10709899926e-20 |
| 391.08330074305826 | 2.48294832696e-12 | 391.08330074305826 | 7.99791418398 | 391.08330074305826 | 3.25865868149e-20 |
| 390.2185223725286 | 1.73656945868e-12 | 390.2185223725286 | 6.86619959039 | 390.2185223725286 | 2.07510868251e-20 |
| 389.3575600259571 | 1.21214325447e-12 | 389.3575600259571 | 5.88300640386 | 389.3575600259571 | 1.31880381404e-20 |
| 388.5003885003885 | 8.44409386327e-13 | 388.5003885003885 | 5.03066131284 | 388.5003885003885 | 8.36482601096e-21 |
| 387.6469828143171 | 5.87069432854e-13 | 387.6469828143171 | 4.29332106249 | 387.6469828143171 | 5.29506136592e-21 |
| 386.7973182052604 | 4.07345757567e-13 | 386.7973182052604 | 3.65682242174 | 386.7973182052604 | 3.34520277814e-21 |
| 385.95137012736393 | 2.82081263147e-13 | 385.95137012736393 | 3.1085388417 | 385.95137012736393 | 2.10916843457e-21 |
| 385.1091142490372 | 1.949497106e-13 | 385.1091142490372 | 2.63724436085 | 385.1091142490372 | 1.32720325587e-21 |
| 384.2705264506212 | 1.3446469246e-13 | 384.2705264506212 | 2.23298514326 | 384.2705264506212 | 8.33491110891e-22 |
| 383.4355828220859 | 9.25616832988e-14 | 383.4355828220859 | 1.8869588866 | 383.4355828220859 | 5.22398430232e-22 |
| 382.6042596607575 | 6.3590397244e-14 | 382.6042596607575 | 1.59140220444 | 382.6042596607575 | 3.2676844299e-22 |
| 381.77653346907607 | 4.36002666167e-14 | 381.77653346907607 | 1.33948597362 | 381.77653346907607 | 2.03993207722e-22 |
| 380.95238095238096 | 2.9834864171e-14 | 380.95238095238096 | 1.12521854073 | 380.95238095238096 | 1.27095055934e-22 |
| 380.1317790167258 | 2.03749380961e-14 | 380.1317790167258 | 0.943356600489 | 380.1317790167258 | 7.9027629553e-23 |
| 379.31470476672143 | 1.38869171295e-14 | 379.31470476672143 | 0.789323493465 | 379.31470476672143 | 4.90418225894e-23 |
| 378.5011355034065 | 9.4461035643e-15 | 378.5011355034065 | 0.659134617999 | 378.5011355034065 | 3.0373273065e-23 |
| 377.69104872214524 | 6.41264022036e-15 | 377.69104872214524 | 0.549329611742 | 377.69104872214524 | 1.8773875433e-23 |
| 376.88442211055275 | 4.34468564938e-15 | 376.88442211055275 | 0.456910929589 | 376.88442211055275 | 1.15812013559e-23 |
| 376.081233546446 | 2.93776570926e-15 | 376.081233546446 | 0.379288426053 | 376.081233546446 | 7.13001814311e-24 |
| 375.28146109582184 | 1.98250032899e-15 | 375.28146109582184 | 0.314229539993 | 375.28146109582184 | 4.38091672548e-24 |
| 374.48508301086 | 1.33520113184e-15 | 374.48508301086 | 0.259814676754 | 374.48508301086 | 2.68643724749e-24 |
| 373.69207772795215 | 8.97464809697e-16 | 373.69207772795215 | 0.214397386163 | 373.69207772795215 | 1.64409057933e-24 |
| 372.9024238657551 | 6.02040181858e-16 | 372.9024238657551 | 0.176568943265 | 372.9024238657551 | 1.00418127391e-24 |
| 372.11610022326965 | 4.03061058372e-16 | 372.11610022326965 | 0.14512695129 | 372.11610022326965 | 6.12118960096e-25 |
| 371.33308577794276 | 2.69310642864e-16 | 371.33308577794276 | 0.11904760212 | 371.33308577794276 | 3.7238903107e-25 |
| 370.55335968379444 | 1.79586425051e-16 | 370.55335968379444 | 0.0974612477858 | 370.55335968379444 | 2.26097240763e-25 |
| 369.7769012695673 | 1.19517309173e-16 | 369.7769012695673 | 0.07963095642 | 369.7769012695673 | 1.37003284045e-25 |
| 369.0036900369003 | 7.9382618689e-17 | 369.0036900369003 | 0.0649337471673 | 369.0036900369003 | 8.28521961803e-26 |
| 368.23370565852457 | 5.26207881883e-17 | 368.23370565852457 | 0.0528442201306 | 368.23370565852457 | 5.00051118843e-26 |
| 367.4669279764821 | 3.48118087936e-17 | 367.4669279764821 | 0.0429203191793 | 367.4669279764821 | 3.0120495515e-26 |
| 366.7033370003667 | 2.29843985092e-17 | 366.7033370003667 | 0.0347909869338 | 366.7033370003667 | 1.81070270626e-26 |
| 365.9429129055867 | 1.51452696291e-17 | 365.9429129055867 | 0.0281454921911 | 365.9429129055867 | 1.08634937295e-26 |
| 365.1856360316494 | 9.9599736362e-18 | 365.1856360316494 | 0.0227242302411 | 365.1856360316494 | 6.50472908683e-27 |
| 364.4314868804664 | 6.53697285083e-18 | 364.4314868804664 | 0.0183108157574 | 364.4314868804664 | 3.88710468055e-27 |
| 363.68044611468054 | 4.28186022861e-18 | 363.68044611468054 | 0.0147253061009 | 363.68044611468054 | 2.31825169596e-27 |
| 362.93249455601256 | 2.79914634605e-18 | 362.93249455601256 | 0.0118184098764 | 362.93249455601256 | 1.37985122063e-27 |
| 362.1876131836291 | 1.8262323698e-18 | 362.1876131836291 | 0.00946655136757 | 362.1876131836291 | 8.19674196465e-28 |
| 361.4457831325301 | 1.18911478952e-18 | 361.4457831325301 | 0.00756767604017 | 361.4457831325301 | 4.85945528378e-28 |
| 360.7069856919562 | 7.7273189416e-19 | 360.7069856919562 | 0.00603769564362 | 360.7069856919562 | 2.87522106217e-28 |
| 359.97120230381563 | 5.01154008205e-19 | 359.97120230381563 | 0.00480748359287 | 359.97120230381563 | 1.69782223207e-28 |
| 359.2384145611304 | 3.24377626267e-19 | 359.2384145611304 | 0.00382034231176 | 359.2384145611304 | 1.00057699754e-28 |
| 358.50860420650093 | 2.09540457404e-19 | 358.50860420650093 | 0.00302987412443 | 358.50860420650093 | 5.88499551252e-29 |
| 357.7817531305903 | 1.35089687414e-19 | 357.7817531305903 | 0.00239819615408 | 357.7817531305903 | 3.45445134384e-29 |
| 357.057843370626 | 8.69188194293e-20 | 357.057843370626 | 0.0018944475971 | 357.057843370626 | 2.02371497837e-29 |
| 356.33685710892024 | 5.58139493615e-20 | 356.33685710892024 | 0.00149354475662 | 356.33685710892024 | 1.18319665687e-29 |
| 355.6187766714082 | 3.57691814779e-20 | 355.6187766714082 | 0.00117514541515 | 355.6187766714082 | 6.90401685544e-30 |
| 354.9035845262037 | 2.28777106656e-20 | 354.9035845262037 | 0.000922789573623 | 354.9035845262037 | 4.02053709125e-30 |
| 354.1912632821723 | 1.46033817119e-20 | 354.1912632821723 | 0.000723188353322 | 354.1912632821723 | 2.33670357846e-30 |
| 353.48179568752204 | 9.30318429173e-21 | 353.48179568752204 | 0.000565637015498 | 353.48179568752204 | 1.35537819486e-30 |
| 352.77516462841015 | 5.91489615313e-21 | 352.77516462841015 | 0.000441531664549 | 352.77516462841015 | 7.84611528866e-31 |
| 352.07135312756714 | 3.7531846992e-21 | 352.07135312756714 | 0.000343972324601 | 352.07135312756714 | 4.53300497387e-31 |
| 351.3703443429374 | 2.37678590367e-21 | 351.3703443429374 | 0.000267437771387 | 351.3703443429374 | 2.61369571121e-31 |
| 350.6721215663355 | 1.50216452664e-21 | 350.6721215663355 | 0.000207519812747 | 350.6721215663355 | 1.50404619011e-31 |
| 349.9766682221185 | 9.47506621933e-22 | 349.9766682221185 | 0.000160706688571 | 349.9766682221185 | 8.63782977691e-32 |
| 349.2839678658749 | 5.96464112677e-22 | 349.2839678658749 | 0.000124206946716 | 349.2839678658749 | 4.95091450947e-32 |
| 348.59400418312805 | 3.74734515685e-22 | 348.59400418312805 | 9.58065835532e-05 | 348.59400418312805 | 2.83206705056e-32 |
| 347.90676098805517 | 2.34963491626e-22 | 347.90676098805517 | 7.37534502538e-05 | 347.90676098805517 | 1.61680987618e-32 |
| 347.2222222222222 | 1.47032855296e-22 | 347.2222222222222 | 5.66639490408e-05 | 347.2222222222222 | 9.21195257053e-33 |
| 346.54037195333257 | 9.18260087174e-23 | 346.54037195333257 | 4.34479040833e-05 | 346.54037195333257 | 5.23819607344e-33 |
| 345.8611943739912 | 5.72340314112e-23 | 345.8611943739912 | 3.32482130813e-05 | 345.8611943739912 | 2.97268659995e-33 |
| 345.1846738004832 | 3.56024823826e-23 | 345.1846738004832 | 2.53924883779e-05 | 345.1846738004832 | 1.683657764e-33 |
| 344.5107946715664 | 2.21026087391e-23 | 344.5107946715664 | 1.93543985873e-05 | 344.5107946715664 | 9.5169070198e-34 |
| 343.8395415472779 | 1.36944345427e-23 | 343.8395415472779 | 1.47228387371e-05 | 343.8395415472779 | 5.36877436276e-34 |
| 343.17089910775564 | 8.46802216473e-24 | 343.17089910775564 | 1.11774021615e-05 | 343.17089910775564 | 3.0226773676e-34 |
| 342.50485215207215 | 5.22585289828e-24 | 342.50485215207215 | 8.4689123541e-06 | 342.50485215207215 | 1.69842257203e-34 |
| 341.84138559708293 | 3.21861987128e-24 | 341.84138559708293 | 6.40400749175e-06 | 341.84138559708293 | 9.52438687753e-35 |
| 341.1804844762879 | 1.97842462408e-24 | 341.1804844762879 | 4.83296276178e-06 | 341.1804844762879 | 5.33047141992e-35 |
| 340.522133938706 | 1.21368683813e-24 | 340.522133938706 | 3.64009304067e-06 | 340.522133938706 | 2.97736121263e-35 |
| 339.86631924776253 | 7.43072328387e-25 | 339.86631924776253 | 2.73620688498e-06 | 339.86631924776253 | 1.65971970187e-35 |
| 339.2130257801899 | 4.54038675558e-25 | 339.2130257801899 | 2.05268724635e-06 | 339.2130257801899 | 9.23368995976e-36 |
| 338.56223902494077 | 2.76880213121e-25 | 338.56223902494077 | 1.5368590422e-06 | 338.56223902494077 | 5.12687988262e-36 |
| 337.91394458211306 | 1.68511019443e-25 | 337.91394458211306 | 1.14837223612e-06 | 337.91394458211306 | 2.84098088956e-36 |
| 337.2681281618887 | 1.02353336505e-25 | 337.2681281618887 | 8.56384348623e-07 | 337.2681281618887 | 1.57116138805e-36 |
| 336.6247755834829 | 6.20458895854e-26 | 336.6247755834829 | 6.37370771966e-07 | 336.6247755834829 | 8.67182685746e-37 |
| 335.9838727741068 | 3.75371538154e-26 | 335.9838727741068 | 4.73426918259e-07 | 335.9838727741068 | 4.77680721114e-37 |
| 335.3454057679409 | 2.26645435598e-26 | 335.3454057679409 | 3.50954752491e-07 | 335.3454057679409 | 2.62604488289e-37 |
| 334.709360705121 | 1.36574596941e-26 | 334.709360705121 | 2.59649016e-07 | 334.709360705121 | 1.4408006318e-37 |
| 334.07572383073494 | 8.2135364866e-27 | 334.07572383073494 | 1.91716549355e-07 | 334.07572383073494 | 7.88938138155e-38 |
| 333.44448149383123 | 4.92978259221e-27 | 333.44448149383123 | 1.41276488646e-07 | 333.44448149383123 | 4.31141001532e-38 |
| 332.81562014643885 | 2.95299462249e-27 | 332.81562014643885 | 1.03900477509e-07 | 332.81562014643885 | 2.35143520672e-38 |
| 332.1891263425977 | 1.76536640728e-27 | 332.1891263425977 | 7.62610103176e-08 | 332.1891263425977 | 1.27992352391e-38 |
| 331.5649867374005 | 1.05328129902e-27 | 331.5649867374005 | 5.58630858945e-08 | 331.5649867374005 | 6.95300177753e-39 |
| 330.9431880860452 | 6.27178556847e-28 | 330.9431880860452 | 4.08398976395e-08 | 330.9431880860452 | 3.76962348283e-39 |
| 330.323717242898 | 3.72713687921e-28 | 330.323717242898 | 2.97976297158e-08 | 330.323717242898 | 2.03967476325e-39 |
| 329.70656116056705 | 2.21053195094e-28 | 329.70656116056705 | 2.16978220263e-08 | 329.70656116056705 | 1.10144086406e-39 |
| 329.0917068889864 | 1.30844527604e-28 | 329.0917068889864 | 1.57684103574e-08 | 329.0917068889864 | 5.93606655891e-40 |
| 328.47914157451 | 7.72950430784e-29 | 328.47914157451 | 1.14366025767e-08 | 328.47914157451 | 3.19281424917e-40 |
| 327.86885245901635 | 4.55706304098e-29 | 327.86885245901635 | 8.27834437622e-09 | 327.86885245901635 | 1.71390149763e-40 |
| 327.26082687902255 | 2.68136380369e-29 | 327.26082687902255 | 5.98035947279e-09 | 327.26082687902255 | 9.18195960236e-41 |
| 326.6550522648083 | 1.5745764851e-29 | 326.6550522648083 | 4.31169895183e-09 | 326.6550522648083 | 4.90932983338e-41 |
| 326.05151613955 | 9.22803202926e-30 | 326.05151613955 | 3.10246512953e-09 | 326.05151613955 | 2.61966862471e-41 |
| 325.4502061184639 | 5.39748865397e-30 | 325.4502061184639 | 2.22793606706e-09 | 325.4502061184639 | 1.3951079712e-41 |
| 324.8511099079588 | 3.15073368365e-30 | 324.8511099079588 | 1.5967461386e-09 | 324.8511099079588 | 7.41492214764e-42 |
| 324.25421530479895 | 1.83556161282e-30 | 324.25421530479895 | 1.14210585738e-09 | 324.25421530479895 | 3.93316970019e-42 |
| 323.65951019527455 | 1.06724357873e-30 | 323.65951019527455 | 8.15293867624e-10 | 323.65951019527455 | 2.08216953559e-42 |
| 323.0669825543829 | 6.19291967733e-31 | 323.0669825543829 | 5.80843754497e-10 | 323.0669825543829 | 1.10008642345e-42 |
| 322.4766204450177 | 3.58644897905e-31 | 322.4766204450177 | 4.12992155437e-10 | 322.4766204450177 | 5.80062560954e-43 |
| 321.88841201716735 | 2.07286576848e-31 | 321.88841201716735 | 2.93063417921e-10 | 321.88841201716735 | 3.05253168167e-43 |
| 321.3023455071222 | 1.19568013866e-31 | 321.3023455071222 | 2.07548092579e-10 | 321.3023455071222 | 1.60318203749e-43 |
| 320.71840923669015 | 6.88329153272e-32 | 320.71840923669015 | 1.4669429014e-10 | 320.71840923669015 | 8.40316343026e-44 |
| 320.1365916124213 | 3.95470984935e-32 | 320.1365916124213 | 1.03477278923e-10 | 320.1365916124213 | 4.39582197267e-44 |
| 319.5568811248402 | 2.2676206337e-32 | 319.5568811248402 | 7.28474099751e-11 | 319.5568811248402 | 2.2949577498e-44 |
| 318.97926634768737 | 1.29766767857e-32 | 318.97926634768737 | 5.1182389317e-11 | 318.97926634768737 | 1.19576711933e-44 |
| 318.40373593716834 | 7.41129137991e-33 | 318.40373593716834 | 3.58892448704e-11 | 318.40373593716834 | 6.21807324421e-45 |
| 317.8302786312109 | 4.22436661433e-33 | 317.8302786312109 | 2.51157070526e-11 | 317.8302786312109 | 3.22702532084e-45 |
| 317.2588832487309 | 2.40307114222e-33 | 317.2588832487309 | 1.75413825497e-11 | 317.2588832487309 | 1.6714224313e-45 |
| 316.6895386889053 | 1.36429713651e-33 | 316.6895386889053 | 1.22269898956e-11 | 316.6895386889053 | 8.6398736822e-46 |
| 316.1222339304531 | 7.73016243707e-34 | 316.1222339304531 | 8.5057498936e-12 | 316.1222339304531 | 4.45723849992e-46 |
| 315.55695803092453 | 4.37124939707e-34 | 315.55695803092453 | 5.90531385894e-12 | 315.55695803092453 | 2.29488890714e-46 |
| 314.99370012599746 | 2.4669472533e-34 | 314.99370012599746 | 4.09176498596e-12 | 314.99370012599746 | 1.17921982977e-46 |
| 314.432449428781 | 1.38947729696e-34 | 314.432449428781 | 2.82953909538e-12 | 314.432449428781 | 6.04735124245e-47 |
| 313.8731952291274 | 7.81052738479e-35 | 313.8731952291274 | 1.95280121188e-12 | 313.8731952291274 | 3.09508747497e-47 |
| 313.31592689295036 | 4.38173972281e-35 | 313.31592689295036 | 1.34504771905e-12 | 313.31592689295036 | 1.58094941744e-47 |
| 312.76063386155124 | 2.45329688256e-35 | 312.76063386155124 | 9.24601657703e-13 | 312.76063386155124 | 8.05935591361e-48 |
| 312.2073056509522 | 1.37085320013e-35 | 312.2073056509522 | 6.34320813461e-13 | 312.2073056509522 | 4.10034131398e-48 |
| 311.65593185123623 | 7.64485242729e-36 | 311.65593185123623 | 4.34310745038e-13 | 311.65593185123623 | 2.08198212306e-48 |
| 311.1065021258944 | 4.25485314943e-36 | 311.1065021258944 | 2.9677649708e-13 | 311.1065021258944 | 1.05504575994e-48 |
| 310.5590062111801 | 2.36340068864e-36 | 310.5590062111801 | 2.0239312331e-13 | 310.5590062111801 | 5.33584137238e-49 |
| 310.01343391546965 | 1.31016940278e-36 | 310.01343391546965 | 1.37752445583e-13 | 310.01343391546965 | 2.69322003766e-49 |
| 309.4697751186301 | 7.24861212142e-37 | 309.4697751186301 | 9.35707693547e-14 | 309.4697751186301 | 1.35668204419e-49 |
| 308.9280197713932 | 4.00239163339e-37 | 308.9280197713932 | 6.34334598442e-14 | 308.9280197713932 | 6.82058507679e-50 |
| 308.3881578947368 | 2.20557391133e-37 | 308.3881578947368 | 4.29174505825e-14 | 308.3881578947368 | 3.42217722808e-50 |
| 307.8501795792714 | 1.21300045308e-37 | 307.8501795792714 | 2.89792237565e-14 | 307.8501795792714 | 1.71364428485e-50 |
| 307.31407498463426 | 6.65790544533e-38 | 307.31407498463426 | 1.95288602168e-14 | 307.31407498463426 | 8.56399036269e-51 |
| 306.77983433888943 | 3.64713295814e-38 | 306.77983433888943 | 1.31342222686e-14 | 306.77983433888943 | 4.2713875366e-51 |
| 306.2474479379338 | 1.99389774394e-38 | 306.2474479379338 | 8.81595047968e-15 | 306.2474479379338 | 2.12617543165e-51 |
| 305.7169061449098 | 1.0879062728e-38 | 305.7169061449098 | 5.90569804813e-15 | 305.7169061449098 | 1.0562495284e-51 |
| 305.1881993896236 | 5.92403195858e-39 | 305.1881993896236 | 3.94830460038e-15 | 305.1881993896236 | 5.23686373762e-52 |
| 304.6613181679699 | 3.21944209019e-39 | 304.6613181679699 | 2.63443432839e-15 | 304.6613181679699 | 2.59127397113e-52 |
| 304.1362530413625 | 1.74614842049e-39 | 304.1362530413625 | 1.75429012656e-15 | 304.1362530413625 | 1.27965443057e-52 |
| 303.61299463617036 | 9.45189756335e-40 | 303.61299463617036 | 1.1658771117e-15 | 303.61299463617036 | 6.30680480526e-53 |
| 303.09153364316023 | 5.10615707059e-40 | 303.09153364316023 | 7.73288316659e-16 | 303.09153364316023 | 3.10215424631e-53 |
| 302.571860816944 | 2.7530027598e-40 | 302.571860816944 | 5.11879144783e-16 | 302.571860816944 | 1.522841486e-53 |
| 302.0539669754329 | 1.48134575222e-40 | 302.0539669754329 | 3.38166620576e-16 | 302.0539669754329 | 7.46076437055e-54 |
| 301.5378429992964 | 7.95506145747e-41 | 301.5378429992964 | 2.22962257945e-16 | 301.5378429992964 | 3.64795323093e-54 |
| 301.02347983142687 | 4.26351656653e-41 | 301.02347983142687 | 1.46713232101e-16 | 301.02347983142687 | 1.78013325138e-54 |
| 300.5108684764098 | 2.28049793582e-41 | 300.5108684764098 | 9.63484051153e-17 | 300.5108684764098 | 8.6694805456e-55 |

| **GON1-Al6** | |
| --- | --- |
| Wavelength (nm) | Abs |
| 2000.0 | 15.2580351259 |
| 1977.5873434410018 | 17.3725166702 |
| 1955.671447196871 | 19.7459379799 |
| 1934.2359767891683 | 22.404859807 |
| 1913.265306122449 | 25.3778403282 |
| 1892.7444794952683 | 28.695487716 |
| 1872.6591760299625 | 32.390500069 |
| 1852.9956763434218 | 36.4976905516 |
| 1833.7408312958437 | 41.0539955183 |
| 1814.8820326678765 | 46.098463337 |
| 1796.4071856287424 | 51.6722216096 |
| 1778.3046828689983 | 57.8184204922 |
| 1760.5633802816901 | 64.5821498702 |
| 1743.1725740848342 | 72.0103282344 |
| 1726.1219792865363 | 80.1515612428 |
| 1709.4017094017095 | 89.0559681386 |
| 1693.002257336343 | 98.7749744358 |
| 1676.9144773616547 | 109.361069577 |
| 1661.1295681063123 | 120.867528611 |
| 1645.6390565002741 | 133.348097346 |
| 1630.4347826086955 | 146.856640879 |
| 1615.5088852988692 | 161.446755909 |
| 1600.8537886872998 | 177.171347792 |
| 1586.4621893178212 | 194.082173877 |
| 1572.3270440251572 | 212.229355302 |
| 1558.4415584415583 | 231.660860035 |
| 1544.799176107106 | 252.421960669 |
| 1531.3935681470139 | 274.554671077 |
| 1518.2186234817814 | 298.097166752 |
| 1505.2684395383842 | 323.083194238 |
| 1492.5373134328358 | 349.541475718 |
| 1480.0197335964478 | 377.49511532 |
| 1467.7103718199608 | 406.961014239 |
| 1455.604075691412 | 437.949302147 |
| 1443.6958614051973 | 470.462792719 |
| 1431.9809069212408 | 504.496471267 |
| 1420.4545454545455 | 540.037022634 |
| 1409.1122592766555 | 577.062407408 |
| 1397.9496738117427 | 615.541494386 |
| 1386.9625520110958 | 655.433756906 |
| 1376.1467889908256 | 696.689040191 |
| 1365.4984069185252 | 739.247406283 |
| 1355.0135501355014 | 783.039062366 |
| 1344.688480502017 | 827.984377416 |
| 1334.5195729537365 | 873.993991117 |
| 1324.5033112582782 | 920.969017833 |
| 1314.6362839614374 | 968.801347211 |
| 1304.9151805132665 | 1017.3740417 |
| 1295.3367875647668 | 1066.56182987 |
| 1285.8979854264894 | 1116.23169303 |
| 1276.5957446808509 | 1166.24354123 |
| 1267.427122940431 | 1216.45097337 |
| 1258.3892617449665 | 1266.70211462 |
| 1249.4793835901708 | 1316.84052336 |
| 1240.6947890818858 | 1366.70615832 |
| 1232.0328542094455 | 1416.1363959 |
| 1223.4910277324632 | 1464.9670864 |
| 1215.0668286755772 | 1513.03363736 |
| 1206.7578439259853 | 1560.1721117 |
| 1198.5617259288852 | 1606.22032775 |
| 1190.4761904761904 | 1651.01894852 |
| 1182.4990145841543 | 1694.41254723 |
| 1174.6280344557556 | 1736.2506369 |
| 1166.8611435239206 | 1776.38865183 |
| 1159.19629057187 | 1814.68887015 |
| 1151.6314779270633 | 1851.02126695 |
| 1144.1647597254005 | 1885.26428923 |
| 1136.794240242516 | 1917.30554461 |
| 1129.5180722891566 | 1947.04239779 |
| 1122.334455667789 | 1974.38246966 |
| 1115.2416356877322 | 1999.24403601 |
| 1108.2379017362393 | 2021.55632417 |
| 1101.3215859030836 | 2041.25970763 |
| 1094.4910616563297 | 2058.30580018 |
| 1087.7447425670775 | 2072.65745282 |
| 1081.081081081081 | 2084.28865785 |
| 1074.4985673352435 | 2093.18436605 |
| 1067.995728017088 | 2099.34022389 |
| 1061.5711252653928 | 2102.76223867 |
| 1055.2233556102708 | 2103.46638021 |
| 1048.951048951049 | 2101.47812848 |
| 1042.752867570386 | 2096.83197641 |
| 1036.6275051831374 | 2089.57089791 |
| 1030.5736860185502 | 2079.74579033 |
| 1024.5901639344263 | 2067.41490083 |
| 1018.6757215619693 | 2052.64324552 |
| 1012.829169480081 | 2035.5020292 |
| 1007.0493454179255 | 2016.06807339 |
| 1001.3351134846461 | 1994.42325884 |
| 995.6853634251576 | 1970.65398802 |
| 990.0990099009902 | 1944.85067192 |
| 984.5749917952082 | 1917.10724429 |
| 979.1122715404699 | 1887.52070566 |
| 973.7098344693281 | 1856.1906979 |
| 968.3666881859263 | 1823.21910956 |
| 963.0818619582664 | 1788.70971101 |
| 957.8544061302682 | 1752.76781761 |
| 952.6833915528738 | 1715.49997838 |
| 947.5679090334806 | 1677.01368734 |
| 942.5070688030161 | 1637.41711374 |
| 937.4999999999999 | 1596.81884775 |
| 932.5458501709667 | 1555.32765745 |
| 927.643784786642 | 1513.05225345 |
| 922.7929867733004 | 1470.10105738 |
| 917.9926560587514 | 1426.58197085 |
| 913.2420091324201 | 1382.6021419 |
| 908.5402786190186 | 1338.26772644 |
| 903.8867128653209 | 1293.68364285 |
| 899.2805755395683 | 1248.95331838 |
| 894.7211452430658 | 1204.17842684 |
| 890.2077151335311 | 1159.45861766 |
| 885.7395925597874 | 1114.89123715 |
| 881.316098707403 | 1070.57104328 |
| 876.9365682548962 | 1026.58991615 |
| 872.6003490401396 | 983.036566629 |
| 868.3068017366135 | 939.996246192 |
| 864.0552995391705 | 897.550461378 |
| 859.8452278589854 | 855.776696475 |
| 855.6759840273816 | 814.748148246 |
| 851.5469770082316 | 774.533476575 |
| 847.457627118644 | 735.196574799 |
| 843.4073657576608 | 696.796363403 |
| 839.3956351426972 | 659.386610456 |
| 835.421888053467 | 623.015781847 |
| 831.4855875831485 | 587.726923966 |
| 827.5862068965516 | 553.557580998 |
| 823.7232289950576 | 520.539748464 |
| 819.8961464881114 | 488.699864098 |
| 816.1044613710554 | 458.058836551 |
| 812.3476848090983 | 428.63211184 |
| 808.6253369272237 | 400.429776863 |
| 804.9369466058491 | 373.456698777 |
| 801.2820512820513 | 347.71269847 |
| 797.6601967561818 | 323.192755904 |
| 794.0709370037056 | 299.887244684 |
| 790.5138339920949 | 277.782192823 |
| 786.9884575026232 | 256.859566373 |
| 783.4943849569078 | 237.097572389 |
| 780.0312012480499 | 218.470977488 |
| 776.598498576236 | 200.951438216 |
| 773.1958762886597 | 184.507839379 |
| 769.8229407236336 | 169.106636582 |
| 766.4793050587633 | 154.712199278 |
| 763.1645891630628 | 141.287150829 |
| 759.8784194528876 | 128.792702286 |
| 756.6204287515762 | 117.188976826 |
| 753.390256152687 | 106.435322112 |
| 750.1875468867216 | 96.4906081293 |
| 747.011952191235 | 87.3135083917 |
| 743.86312918423 | 78.8627627733 |
| 740.7407407407408 | 71.0974205568 |
| 737.6444553725104 | 63.977062644 |
| 734.5739471106758 | 57.4620022179 |
| 731.528895391368 | 51.5134634638 |
| 728.5089849441475 | 46.0937382713 |
| 725.5139056831922 | 41.1663211156 |
| 722.543352601156 | 36.6960225797 |
| 719.5970256656271 | 32.6490622026 |
| 716.6746297181079 | 28.9931415417 |
| 713.7758743754462 | 25.6974985009 |
| 710.9004739336492 | 22.7329441166 |
| 708.0481472740146 | 20.0718830975 |
| 705.2186177715091 | 17.6883194916 |
| 702.4116132053383 | 15.5578489036 |
| 699.6268656716418 | 13.6576387082 |
| 696.8641114982578 | 11.9663977051 |
| 694.1230911614992 | 10.464336641 |
| 691.4035492048858 | 9.13312098144 |
| 688.7052341597796 | 7.95581726283 |
| 686.027898467871 | 6.91683428341 |
| 683.371298405467 | 6.00186031488 |
| 680.7351940095303 | 5.19779742752 |
| 678.1193490054249 | 4.49269392969 |
| 675.5235307363206 | 3.8756758261 |
| 672.9475100942127 | 3.33687810189 |
| 670.3910614525139 | 2.86737654219 |
| 667.8539626001781 | 2.4591207019 |
| 665.335994677312 | 2.10486854839 |
| 662.8369421122403 | 1.79812321239 |
| 660.3565925599823 | 1.53307220001 |
| 657.8947368421053 | 1.30452934248 |
| 655.4511688879178 | 1.10787969011 |
| 653.0256856769699 | 0.939027493444 |
| 650.6180871828237 | 0.794347358146 |
| 648.2281763180639 | 0.670638609766 |
| 645.8557588805166 | 0.565082861427 |
| 643.5006435006435 | 0.475204740198 |
| 641.1626415900834 | 0.398835696882 |
| 638.8415672913118 | 0.334080798438 |
| 636.5372374283895 | 0.279288381997 |
| 634.2494714587738 | 0.233022433873 |
| 631.9780914261638 | 0.194037545674 |
| 629.7229219143577 | 0.161256292147 |
| 627.4837900020916 | 0.133748871167 |
| 625.2605252188412 | 0.110714844996 |
| 623.0529595015576 | 0.0914668230096 |
| 620.8609271523178 | 0.0754159292022 |
| 618.6842647968654 | 0.0620589025701 |
| 616.5228113440197 | 0.050966684479 |
| 614.3764079459348 | 0.0417743541875 |
| 612.2448979591836 | 0.0341722814317 |
| 610.1281269066504 | 0.0278983731868 |
| 608.0259424402108 | 0.0227313001966 |
| 605.9381943041809 | 0.0184845974152 |
| 603.864734299517 | 0.0150015409909 |
| 601.8054162487462 | 0.0121507127329 |
| 599.7600959616153 | 0.00982217101892 |
| 597.7286312014345 | 0.00792415477514 |
| 595.7108816521048 | 0.00638025441878 |
| 593.7067088858104 | 0.00512699047025 |
| 591.7159763313609 | 0.00411174688906 |
| 589.7385492431688 | 0.00329101205739 |
| 587.7742946708463 | 0.00262888572696 |
| 585.8230814294083 | 0.00209581516667 |
| 583.8847800700661 | 0.00166752821514 |
| 581.9592628516003 | 0.00132413497415 |
| 580.046403712297 | 0.00104937349844 |
| 578.1460782424359 | 0.000829978071318 |
| 576.2581636573184 | 0.000655151530744 |
| 574.3825387708214 | 0.000516125655146 |
| 572.5190839694656 | 0.000405795860445 |
| 570.6676811869887 | 0.000318418427055 |
| 568.8282138794084 | 0.000249360194456 |
| 567.000567000567 | 0.00019489215674 |
| 565.1846269781461 | 0.000152019689215 |
| 563.3802816901408 | 0.000118343255823 |
| 561.5874204417821 | 9.19444104908e-05 |
| 559.8059339428997 | 7.12927312644e-05 |
| 558.0357142857143 | 5.5170031491e-05 |
| 556.2766549230483 | 4.26087926559e-05 |
| 554.52865064695 | 3.28422728184e-05 |
| 552.791597567717 | 2.5264175161e-05 |
| 551.0653930933137 | 1.93961240044e-05 |
| 549.3499359091741 | 1.48615003795e-05 |
| 547.645125958379 | 1.13644443887e-05 |
| 545.950864422202 | 8.67304451936e-06 |
| 544.2670537010159 | 6.60591122661e-06 |
| 542.5935973955508 | 5.02147904057e-06 |
| 540.9304002884962 | 3.80950295392e-06 |
| 539.2773683264425 | 2.88431501666e-06 |
| 537.6344086021505 | 2.17948940185e-06 |
| 536.0014293371448 | 1.64363168743e-06 |
| 534.3783398646241 | 1.23706324359e-06 |
| 532.7650506126798 | 9.2921654934e-07 |
| 531.1614730878186 | 6.96593770993e-07 |
| 529.5675198587819 | 5.21170515904e-07 |
| 527.9831045406547 | 3.89150574371e-07 |
| 526.4081417792595 | 2.89996718622e-07 |
| 524.8425472358292 | 2.15678099735e-07 |
| 523.2862375719518 | 1.60087180782e-07 |
| 521.7391304347826 | 1.18589051501e-07 |
| 520.2011444425177 | 8.76738652345e-08 |
| 518.6721991701245 | 6.46894143433e-08 |
| 517.1522151353215 | 4.76358350115e-08 |
| 515.6411137848057 | 3.500836535e-08 |
| 514.1388174807198 | 2.56771820766e-08 |
| 512.6452494873547 | 1.87957801907e-08 |
| 511.1603339580848 | 1.37312734066e-08 |
| 509.683995922528 | 1.00114901397e-08 |
| 508.2161612739285 | 7.28490811011e-09 |
| 506.7567567567567 | 5.29038002253e-09 |
| 505.3057099545225 | 3.83430885166e-09 |
| 503.8629492777964 | 2.77347808731e-09 |
| 502.4284039524367 | 2.00216471832e-09 |
| 501.00200400801606 | 1.44248824181e-09 |
| 499.5836802664446 | 1.03719918449e-09 |
| 498.1733643307871 | 7.44302458539e-10 |
| 496.7709885742673 | 5.33057597554e-10 |
| 495.3764861294584 | 3.81009859652e-10 |
| 493.98979087765514 | 2.71791380385e-10 |
| 492.61083743842363 | 1.93496239239e-10 |
| 491.2395611593253 | 1.37482290203e-10 |
| 489.8758981058131 | 9.74896170068e-11 |
| 488.5197850512946 | 6.89933676023e-11 |
| 487.17115946735953 | 4.87296949716e-11 |
| 485.82995951416996 | 3.43492618713e-11 |
| 484.49612403100775 | 2.4164537591e-11 |
| 483.16959252697694 | 1.69659010626e-11 |
| 481.8503051718599 | 1.1888107666e-11 |
| 480.5382027871216 | 8.31353798244e-12 |
| 479.23322683706067 | 5.80224943119e-12 |
| 477.9353194201051 | 4.04151556209e-12 |
| 476.64442326024783 | 2.80950277621e-12 |
| 475.3604816986214 | 1.94918034373e-12 |
| 474.08343868520853 | 1.34962130767e-12 |
| 472.8132387706856 | 9.32629552751e-13 |
| 471.5498270983967 | 6.43196632527e-13 |
| 470.29314939645707 | 4.42706288874e-13 |
| 469.04315196998124 | 3.04105979676e-13 |
| 467.7997816934352 | 2.08483440712e-13 |
| 466.5629860031104 | 1.42644656675e-13 |
| 465.33271288971605 | 9.74039984913e-14 |
| 464.10891089108907 | 6.63797169646e-14 |
| 462.8915290850177 | 4.51472539664e-14 |
| 461.68051708217905 | 3.06453520751e-14 |
| 460.47582501918646 | 2.07603762898e-14 |
| 459.2774035517452 | 1.40359934353e-14 |
| 458.0852038479157 | 9.47083827344e-15 |
| 456.89917758148033 | 6.3778017086e-15 |
| 455.7192769254139 | 4.28638258258e-15 |
| 454.54545454545456 | 2.87506837023e-15 |
| 453.3776635937736 | 1.92461004294e-15 |
| 452.2158577027434 | 1.28580361787e-15 |
| 451.05999097880016 | 8.57321792543e-16 |
| 449.9100179964007 | 5.70493114197e-16 |
| 448.7658937920718 | 3.78873537955e-16 |
| 447.6275738585497 | 2.51116623095e-16 |
| 446.49501413900873 | 1.66109309747e-16 |
| 445.36817102137763 | 1.09660395045e-16 |
| 444.247001332741 | 7.22508498087e-17 |
| 443.13146233382565 | 4.7508731384e-17 |
| 442.02151171357 | 3.11774956973e-17 |
| 440.9171075837742 | 2.0419558228e-17 |
| 439.8182084738308 | 1.33471571975e-17 |
| 438.72477332553376 | 8.70699966879e-18 |
| 437.636761487965 | 5.66872774183e-18 |
| 436.5541327124563 | 3.68332455942e-18 |
| 435.4768471476266 | 2.38853547848e-18 |
| 434.4048653344918 | 1.54582640745e-18 |
| 433.3381482016467 | 9.98451710322e-19 |
| 432.2766570605187 | 6.43621770557e-19 |
| 431.22035360069 | 4.14068033003e-19 |
| 430.1691998852882 | 2.65858161838e-19 |
| 429.1231583464454 | 1.70359206577e-19 |
| 428.0821917808219 | 1.08947817901e-19 |
| 427.0462633451957 | 6.95358519572e-20 |
| 426.01533655211585 | 4.42931268209e-20 |
| 424.9893752656184 | 2.8157961814e-20 |
| 423.96834369700395 | 1.78650159035e-20 |
| 422.9522064006767 | 1.13120916282e-20 |
| 421.9409282700422 | 7.14857926083e-21 |
| 420.93447453346425 | 4.5085186937e-21 |
| 419.9328107502799 | 2.83782315399e-21 |
| 418.93590280687056 | 1.78268288917e-21 |
| 417.94371691278906 | 1.11763547705e-21 |
| 416.9562195969423 | 6.99300078662e-22 |
| 415.97337770382694 | 4.36680993615e-22 |
| 414.99515838981876 | 2.72146226691e-22 |
| 414.0215291195142 | 1.69269089891e-22 |
| 413.0524576621231 | 1.05072802416e-22 |
| 412.08791208791206 | 6.50938990958e-23 |
| 411.1278607646978 | 4.02464493249e-23 |
| 410.17227235438884 | 2.48343141667e-23 |
| 409.22111580957574 | 1.5293753441e-23 |
| 408.2743603701687 | 9.39968526389e-24 |
| 407.33197556008145 | 5.76567095568e-24 |
| 406.39393118396094 | 3.52958552297e-24 |
| 405.46019732396263 | 2.15642758694e-24 |
| 404.53074433656957 | 1.31487165286e-24 |
| 403.6055428494551 | 8.00145855205e-25 |
| 402.68456375838923 | 4.85950765996e-25 |
| 401.76777822418643 | 2.94545706059e-25 |
| 400.85515766969536 | 1.78176504435e-25 |
| 399.9466737768297 | 1.07568592652e-25 |
| 399.0422984836393 | 6.48123625213e-26 |
| 398.14200398142003 | 3.89733315369e-26 |
| 397.24576271186436 | 2.33891546212e-26 |
| 396.3535473642489 | 1.40087321459e-26 |
| 395.46533087266016 | 8.3737588816e-27 |
| 394.5810864132579 | 4.99550484932e-27 |
| 393.7007874015748 | 2.97423740936e-27 |
| 392.82440748985204 | 1.76729558331e-27 |
| 391.9519205644107 | 1.04804532678e-27 |
| 391.08330074305826 | 6.20280677918e-28 |
| 390.2185223725286 | 3.6638168299e-28 |
| 389.3575600259571 | 2.15981506946e-28 |
| 388.5003885003885 | 1.27068146202e-28 |
| 387.6469828143171 | 7.46095008278e-29 |
| 386.7973182052604 | 4.37208793209e-29 |
| 385.95137012736393 | 2.55694245878e-29 |
| 385.1091142490372 | 1.49241750682e-29 |
| 384.2705264506212 | 8.6935474366e-30 |
| 383.4355828220859 | 5.05406746333e-30 |
| 382.6042596607575 | 2.93239421757e-30 |
| 381.77653346907607 | 1.69801290798e-30 |
| 380.95238095238096 | 9.81289006513e-31 |
| 380.1317790167258 | 5.6596580533e-31 |
| 379.31470476672143 | 3.25777259082e-31 |
| 378.5011355034065 | 1.87149491134e-31 |
| 377.69104872214524 | 1.07298549055e-31 |
| 376.88442211055275 | 6.13954746211e-32 |
| 376.081233546446 | 3.50603450523e-32 |
| 375.28146109582184 | 1.99817409337e-32 |
| 374.48508301086 | 1.13654798175e-32 |
| 373.69207772795215 | 6.45177984255e-33 |
| 372.9024238657551 | 3.65517871973e-33 |
| 372.11610022326965 | 2.06668842449e-33 |
| 371.33308577794276 | 1.16621523466e-33 |
| 370.55335968379444 | 6.56779709085e-34 |
| 369.7769012695673 | 3.69145906273e-34 |
| 369.0036900369003 | 2.07068338721e-34 |
| 368.23370565852457 | 1.15922210744e-34 |
| 367.4669279764821 | 6.47674686699e-35 |
| 366.7033370003667 | 3.6114741073e-35 |
| 365.9429129055867 | 2.00978407354e-35 |
| 365.1856360316494 | 1.11622464812e-35 |
| 364.4314868804664 | 6.18715689623e-36 |
| 363.68044611468054 | 3.4226931298e-36 |
| 362.93249455601256 | 1.88965321229e-36 |
| 362.1876131836291 | 1.0411985871e-36 |
| 361.4457831325301 | 5.72561765202e-37 |
| 360.7069856919562 | 3.14230567088e-37 |
| 359.97120230381563 | 1.72112268285e-37 |
| 359.2384145611304 | 9.40832996369e-38 |
| 358.50860420650093 | 5.13275536282e-38 |
| 357.7817531305903 | 2.79464021019e-38 |
| 357.057843370626 | 1.51858307291e-38 |
| 356.33685710892024 | 8.23547245147e-39 |
| 355.6187766714082 | 4.45734034007e-39 |
| 354.9035845262037 | 2.40768898568e-39 |
| 354.1912632821723 | 1.29796293006e-39 |
| 353.48179568752204 | 6.98331295551e-40 |
| 352.77516462841015 | 3.74971305144e-40 |
| 352.07135312756714 | 2.00942534868e-40 |
| 351.3703443429374 | 1.07468956416e-40 |
| 350.6721215663355 | 5.736295292e-41 |
| 349.9766682221185 | 3.05574620363e-41 |
| 349.2839678658749 | 1.62457727897e-41 |
| 348.59400418312805 | 8.6198713096e-42 |
| 347.90676098805517 | 4.56455560682e-42 |
| 347.2222222222222 | 2.41231232214e-42 |
| 346.54037195333257 | 1.27234791155e-42 |
| 345.8611943739912 | 6.69754345893e-43 |
| 345.1846738004832 | 3.51854016013e-43 |
| 344.5107946715664 | 1.8447894121e-43 |
| 343.8395415472779 | 9.6531353448e-44 |
| 343.17089910775564 | 5.04112314306e-44 |
| 342.50485215207215 | 2.62738387599e-44 |
| 341.84138559708293 | 1.36664924987e-44 |
| 341.1804844762879 | 7.09460004397e-45 |
| 340.522133938706 | 3.67566633088e-45 |
| 339.86631924776253 | 1.90055987488e-45 |
| 339.2130257801899 | 9.80763613272e-46 |
| 338.56223902494077 | 5.05108232414e-46 |
| 337.91394458211306 | 2.59622221486e-46 |
| 337.2681281618887 | 1.33179257569e-46 |
| 336.6247755834829 | 6.81818263594e-47 |
| 335.9838727741068 | 3.48367782522e-47 |
| 335.3454057679409 | 1.77641589655e-47 |
| 334.709360705121 | 9.04042048322e-48 |
| 334.07572383073494 | 4.59166212609e-48 |
| 333.44448149383123 | 2.32749377998e-48 |
| 332.81562014643885 | 1.17745535104e-48 |
| 332.1891263425977 | 5.9448059008e-49 |
| 331.5649867374005 | 2.99549241346e-49 |
| 330.9431880860452 | 1.50638534383e-49 |
| 330.323717242898 | 7.56033870107e-50 |
| 329.70656116056705 | 3.78689911791e-50 |
| 329.0917068889864 | 1.89305630926e-50 |
| 328.47914157451 | 9.44453633652e-51 |
| 327.86885245901635 | 4.70256778428e-51 |
| 327.26082687902255 | 2.3368282801e-51 |
| 326.6550522648083 | 1.15892637908e-51 |
| 326.05151613955 | 5.73617258665e-52 |
| 325.4502061184639 | 2.83351740106e-52 |
| 324.8511099079588 | 1.39690499866e-52 |
| 324.25421530479895 | 6.87298145017e-53 |
| 323.65951019527455 | 3.37489903718e-53 |
| 323.0669825543829 | 1.65391700082e-53 |
| 322.4766204450177 | 8.08916977879e-54 |
| 321.88841201716735 | 3.94849418636e-54 |
| 321.3023455071222 | 1.92351850227e-54 |
| 320.71840923669015 | 9.35187183552e-55 |
| 320.1365916124213 | 4.53772358654e-55 |
| 319.5568811248402 | 2.19742894906e-55 |
| 318.97926634768737 | 1.06201086231e-55 |
| 318.40373593716834 | 5.12248138493e-56 |
| 317.8302786312109 | 2.46586406631e-56 |
| 317.2588832487309 | 1.18466399128e-56 |
| 316.6895386889053 | 5.68013368228e-57 |
| 316.1222339304531 | 2.71806124922e-57 |
| 315.55695803092453 | 1.29806737268e-57 |
| 314.99370012599746 | 6.18689209888e-58 |
| 314.432449428781 | 2.94296544463e-58 |
| 313.8731952291274 | 1.39712446189e-58 |
| 313.31592689295036 | 6.61945666761e-59 |
| 312.76063386155124 | 3.13001849211e-59 |
| 312.2073056509522 | 1.47709618401e-59 |
| 311.65593185123623 | 6.95677507312e-60 |
| 311.1065021258944 | 3.26997521346e-60 |
| 310.5590062111801 | 1.53397496711e-60 |
| 310.01343391546965 | 7.18173534974e-61 |
| 309.4697751186301 | 3.35565910158e-61 |
| 308.9280197713932 | 1.56481710759e-61 |
| 308.3881578947368 | 7.2826032244e-62 |
| 307.8501795792714 | 3.38257181384e-62 |
| 307.31407498463426 | 1.5679951508e-62 |
| 306.77983433888943 | 7.25403618777e-63 |
| 306.2474479379338 | 3.3492844514e-63 |
| 305.7169061449098 | 1.5433401759e-63 |
| 305.1881993896236 | 7.09755233017e-64 |
| 304.6613181679699 | 3.2575632561e-64 |
| 304.1362530413625 | 1.49215667182e-64 |
| 303.61299463617036 | 6.82139667989e-65 |
| 303.09153364316023 | 3.11221433559e-65 |
| 302.571860816944 | 1.4171081808e-65 |
| 302.0539669754329 | 6.43982142282e-66 |
| 301.5378429992964 | 2.92066641536e-66 |
| 301.02347983142687 | 1.32198766221e-66 |
| 300.5108684764098 | 5.97186745761e-67 |
