# Supplementary material for: Data of electronic, reactivity, optoelectronic, linear and non-linear optical parameters of doping graphene oxide nanosheet with aluminum atom
Source: Data Brief. 2022 Jan 19;41:107840. doi: 10.1016/j.dib.2022.107840 (PMC8801356; doi:10.1016/j.dib.2022.107840)
Supplement: Supplementary file 1 [file mmc1.zip › supplementary file/DATA OF THE UV-VIS SPECTRA/DATA OF THE UV-VIS SPECTRA OF GON2 AND ITS DERIVATIVES (GON2-Alx) B3LYP.docx]

**Data of the UV-Vis spectra of GON2 isomer and its aluminum-doped derivatives (GON2-Alx), computed at the B3LYP/6-31+G(d,p) level of theory**

| **GON2** | | **GON2-Al1** | | **GON2-Al2** | |
| --- | --- | --- | --- | --- | --- |
| Wavelength (nm) | Abs | Wavelength (nm) | Abs | Wavelength (nm) | Abs |
| 2000.0 | 9.74458811691e-09 | 2000.0 | 0.0358441329701 | 2000.0 | 2.12021278921e-13 |
| 1977.5873434410018 | 1.33198633104e-08 | 1977.5873434410018 | 0.043803088288 | 1977.5873434410018 | 3.12436155192e-13 |
| 1955.671447196871 | 1.81707712872e-08 | 1955.671447196871 | 0.0534230531869 | 1955.671447196871 | 4.59494621974e-13 |
| 1934.2359767891683 | 2.47391212376e-08 | 1934.2359767891683 | 0.0650264435545 | 1934.2359767891683 | 6.74430020503e-13 |
| 1913.265306122449 | 3.36149514854e-08 | 1913.265306122449 | 0.0789930029536 | 1913.265306122449 | 9.87940222362e-13 |
| 1892.7444794952683 | 4.55845867101e-08 | 1892.7444794952683 | 0.0957689160394 | 1892.7444794952683 | 1.44431444306e-12 |
| 1872.6591760299625 | 6.16937071311e-08 | 1872.6591760299625 | 0.115877161169 | 1872.6591760299625 | 2.10731837462e-12 |
| 1852.9956763434218 | 8.33299317791e-08 | 1852.9956763434218 | 0.139929232175 | 1852.9956763434218 | 3.06856881229e-12 |
| 1833.7408312958437 | 1.12330708486e-07 | 1833.7408312958437 | 0.168638366055 | 1833.7408312958437 | 4.45942528996e-12 |
| 1814.8820326678765 | 1.51123945259e-07 | 1814.8820326678765 | 0.20283441915 | 1814.8820326678765 | 6.46783945312e-12 |
| 1796.4071856287424 | 2.02910901574e-07 | 1796.4071856287424 | 0.24348053894 | 1796.4071856287424 | 9.36217776015e-12 |
| 1778.3046828689983 | 2.7190349579e-07 | 1778.3046828689983 | 0.291691781536 | 1778.3046828689983 | 1.35248310131e-11 |
| 1760.5633802816901 | 3.63631514625e-07 | 1760.5633802816901 | 0.348755825854 | 1760.5633802816901 | 1.94995291688e-11 |
| 1743.1725740848342 | 4.85339402972e-07 | 1743.1725740848342 | 0.416155933961 | 1743.1725740848342 | 2.80578064945e-11 |
| 1726.1219792865363 | 6.46497576498e-07 | 1726.1219792865363 | 0.495596302678 | 1726.1219792865363 | 4.02921664401e-11 |
| 1709.4017094017095 | 8.59459791705e-07 | 1709.4017094017095 | 0.589029943708 | 1709.4017094017095 | 5.77463894962e-11 |
| 1693.002257336343 | 1.14030634111e-06 | 1693.002257336343 | 0.698689217874 | 1693.002257336343 | 8.25973978161e-11 |
| 1676.9144773616547 | 1.50992309516e-06 | 1676.9144773616547 | 0.827119132929 | 1676.9144773616547 | 1.17908525357e-10 |
| 1661.1295681063123 | 1.99537914817e-06 | 1661.1295681063123 | 0.977213493295 | 1661.1295681063123 | 1.67981464527e-10 |
| 1645.6390565002741 | 2.6316816001e-06 | 1645.6390565002741 | 1.15225396351 | 1645.6390565002741 | 2.38844272711e-10 |
| 1630.4347826086955 | 3.46400548912e-06 | 1630.4347826086955 | 1.35595207457 | 1630.4347826086955 | 3.3892656737e-10 |
| 1615.5088852988692 | 4.5505208859e-06 | 1615.5088852988692 | 1.59249416336 | 1615.5088852988692 | 4.79991677755e-10 |
| 1600.8537886872998 | 5.96596863075e-06 | 1600.8537886872998 | 1.86658918928 | 1600.8537886872998 | 6.78420737804e-10 |
| 1586.4621893178212 | 7.80617228267e-06 | 1586.4621893178212 | 2.18351931909 | 1586.4621893178212 | 9.56977738459e-10 |
| 1572.3270440251572 | 1.01937179142e-05 | 1572.3270440251572 | 2.54919311037 | 1572.3270440251572 | 1.34723038278e-09 |
| 1558.4415584415583 | 1.3285087031e-05 | 1558.4415584415583 | 2.97020105541 | 1558.4415584415583 | 1.89286314231e-09 |
| 1544.799176107106 | 1.72795930109e-05 | 1544.799176107106 | 3.45387317172 | 1544.799176107106 | 2.65420140058e-09 |
| 1531.3935681470139 | 2.24305502581e-05 | 1531.3935681470139 | 4.00833824165 | 1531.3935681470139 | 3.7143758435e-09 |
| 1518.2186234817814 | 2.90592003399e-05 | 1518.2186234817814 | 4.64258421341 | 1518.2186234817814 | 5.18770332239e-09 |
| 1505.2684395383842 | 3.75720337243e-05 | 1505.2684395383842 | 5.36651917841 | 1505.2684395383842 | 7.23105607823e-09 |
| 1492.5373134328358 | 4.84822828436e-05 | 1492.5373134328358 | 6.19103223737 | 1492.5373134328358 | 1.0059250904e-08 |
| 1480.0197335964478 | 6.24365260872e-05 | 1480.0197335964478 | 7.12805346007 | 1480.0197335964478 | 1.39658336523e-08 |
| 1467.7103718199608 | 8.02475375617e-05 | 1467.7103718199608 | 8.19061203306 | 1467.7103718199608 | 1.9351088624e-08 |
| 1455.604075691412 | 0.000102934749308 | 1455.604075691412 | 9.39289157699 | 1455.604075691412 | 2.67597007196e-08 |
| 1443.6958614051973 | 0.000131773967068 | 1443.6958614051973 | 10.7502815032 | 1443.6958614051973 | 3.69312846683e-08 |
| 1431.9809069212408 | 0.000168358304374 | 1431.9809069212408 | 12.2794231691 | 1431.9809069212408 | 5.08680267513e-08 |
| 1420.4545454545455 | 0.00021467268024 | 1420.4545454545455 | 13.9982494881 | 1420.4545454545455 | 6.99250329976e-08 |
| 1409.1122592766555 | 0.000273184671428 | 1409.1122592766555 | 15.9260165496 | 1409.1122592766555 | 9.59307372058e-08 |
| 1397.9496738117427 | 0.000346955030525 | 1397.9496738117427 | 18.0833257225 | 1397.9496738117427 | 1.31347011802e-07 |
| 1386.9625520110958 | 0.00043977178607 | 1386.9625520110958 | 20.4921346383 | 1386.9625520110958 | 1.79481596831e-07 |
| 1376.1467889908256 | 0.000556312541923 | 1376.1467889908256 | 23.1757553976 | 1376.1467889908256 | 2.44769311151e-07 |
| 1365.4984069185252 | 0.000702340402105 | 1365.4984069185252 | 26.1588383063 | 1365.4984069185252 | 3.3314348109e-07 |
| 1355.0135501355014 | 0.000884939877328 | 1355.0135501355014 | 29.4673394406 | 1355.0135501355014 | 4.52525426412e-07 |
| 1344.688480502017 | 0.00111280019429 | 1344.688480502017 | 33.1284703526 | 1344.688480502017 | 6.13468073856e-07 |
| 1334.5195729537365 | 0.00139655464277 | 1334.5195729537365 | 37.1706282816 | 1334.5195729537365 | 8.30000318122e-07 |
| 1324.5033112582782 | 0.00174918597386 | 1324.5033112582782 | 41.6233053139 | 1324.5033112582782 | 1.12073223779e-06 |
| 1314.6362839614374 | 0.00218650941974 | 1314.6362839614374 | 46.5169750576 | 1314.6362839614374 | 1.51029847292e-06 |
| 1304.9151805132665 | 0.00272774665784 | 1304.9151805132665 | 51.8829555572 | 1304.9151805132665 | 2.03123894626e-06 |
| 1295.3367875647668 | 0.00339620600238 | 1295.3367875647668 | 57.7532473732 | 1295.3367875647668 | 2.72644386012e-06 |
| 1285.8979854264894 | 0.00422008629077 | 1285.8979854264894 | 64.1603459972 | 1285.8979854264894 | 3.65232501021e-06 |
| 1276.5957446808509 | 0.00523342435037 | 1276.5957446808509 | 71.1370280618 | 1276.5957446808509 | 4.88291976184e-06 |
| 1267.427122940431 | 0.00647720859591 | 1267.427122940431 | 78.7161111323 | 1267.427122940431 | 6.51518979014e-06 |
| 1258.3892617449665 | 0.00800068422368 | 1258.3892617449665 | 86.9301872463 | 1258.3892617449665 | 8.67584666839e-06 |
| 1249.4793835901708 | 0.00986287864113 | 1249.4793835901708 | 95.8113307748 | 1249.4793835901708 | 1.15301239883e-05 |
| 1240.6947890818858 | 0.0121343791972 | 1240.6947890818858 | 105.390781634 | 1240.6947890818858 | 1.52930250522e-05 |
| 1232.0328542094455 | 0.0148993989516 | 1232.0328542094455 | 115.698605354 | 1232.0328542094455 | 2.02437113149e-05 |
| 1223.4910277324632 | 0.0182581701277 | 1223.4910277324632 | 126.763332033 | 1223.4910277324632 | 2.67438657728e-05 |
| 1215.0668286755772 | 0.0223297090078 | 1215.0668286755772 | 138.61157671 | 1215.0668286755772 | 3.5261074743e-05 |
| 1206.7578439259853 | 0.0272550003206 | 1206.7578439259853 | 151.267644271 | 1206.7578439259853 | 4.63985298106e-05 |
| 1198.5617259288852 | 0.0332006535912 | 1198.5617259288852 | 164.75312251 | 1198.5617259288852 | 6.09326697466e-05 |
| 1190.4761904761904 | 0.040363088422 | 1190.4761904761904 | 179.086467539 | 1190.4761904761904 | 7.98607725935e-05 |
| 1182.4990145841543 | 0.0489733101658 | 1182.4990145841543 | 194.282586248 | 1182.4990145841543 | 0.000104460985967 |
| 1174.6280344557556 | 0.0593023418631 | 1174.6280344557556 | 210.352421022 | 1174.6280344557556 | 0.000136367866813 |
| 1166.8611435239206 | 0.0716673825316 | 1166.8611435239206 | 227.30254239 | 1166.8611435239206 | 0.000177667211529 |
| 1159.19629057187 | 0.0864387657918 | 1159.19629057187 | 245.134755663 | 1159.19629057187 | 0.000231014818341 |
| 1151.6314779270633 | 0.104047796249 | 1151.6314779270633 | 263.845728 | 1151.6314779270633 | 0.000299784865289 |
| 1144.1647597254005 | 0.124995543856 | 1144.1647597254005 | 283.426642594 | 1144.1647597254005 | 0.000388254843046 |
| 1136.794240242516 | 0.149862678467 | 1136.794240242516 | 303.862886856 | 1136.794240242516 | 0.000501835491591 |
| 1129.5180722891566 | 0.179320427783 | 1129.5180722891566 | 325.133781584 | 1129.5180722891566 | 0.000647355999149 |
| 1122.334455667789 | 0.214142741559 | 1122.334455667789 | 347.212358075 | 1122.334455667789 | 0.000833416883305 |
| 1115.2416356877322 | 0.255219743223 | 1115.2416356877322 | 370.06519004 | 1115.2416356877322 | 0.00107082554811 |
| 1108.2379017362393 | 0.303572546457 | 1108.2379017362393 | 393.652286916 | 1108.2379017362393 | 0.0013731325659 |
| 1101.3215859030836 | 0.36036950874 | 1101.3215859030836 | 417.927054822 | 1101.3215859030836 | 0.00175729034625 |
| 1094.4910616563297 | 0.426943985913 | 1094.4910616563297 | 442.836330931 | 1094.4910616563297 | 0.00224446011497 |
| 1087.7447425670775 | 0.5048136413 | 1087.7447425670775 | 468.320496387 | 1087.7447425670775 | 0.00286099813142 |
| 1081.081081081081 | 0.595701349446 | 1081.081081081081 | 494.313672217 | 1081.081081081081 | 0.00363965793249 |
| 1074.4985673352435 | 0.701557717862 | 1074.4985673352435 | 520.744001784 | 1074.4985673352435 | 0.00462105222758 |
| 1067.995728017088 | 0.824585230028 | 1067.995728017088 | 547.534022434 | 1067.995728017088 | 0.00585542601429 |
| 1061.5711252653928 | 0.96726398903 | 1061.5711252653928 | 574.601127892 | 1061.5711252653928 | 0.00740480168403 |
| 1055.2233556102708 | 1.13237901339 | 1055.2233556102708 | 601.858121849 | 1055.2233556102708 | 0.00934556749831 |
| 1048.951048951049 | 1.32304900478 | 1048.951048951049 | 629.213861977 | 1048.951048951049 | 0.011771593007 |
| 1042.752867570386 | 1.54275647112 | 1042.752867570386 | 656.573992348 | 1042.752867570386 | 0.0147979689281 |
| 1036.6275051831374 | 1.79537904836 | 1036.6275051831374 | 683.841760928 | 1036.6275051831374 | 0.0185654849022 |
| 1030.5736860185502 | 2.08522181959 | 1030.5736860185502 | 710.918917532 | 1030.5736860185502 | 0.0232459765591 |
| 1024.5901639344263 | 2.41705038158 | 1024.5901639344263 | 737.706686307 | 1024.5901639344263 | 0.0290486936986 |
| 1018.6757215619693 | 2.79612435673 | 1018.6757215619693 | 764.106805538 | 1018.6757215619693 | 0.0362278642628 |
| 1012.829169480081 | 3.22823099232 | 1012.829169480081 | 790.022626359 | 1012.829169480081 | 0.0450916543757 |
| 1007.0493454179255 | 3.71971843093 | 1007.0493454179255 | 815.3602608 | 1007.0493454179255 | 0.0560127532001 |
| 1001.3351134846461 | 4.27752817465 | 1001.3351134846461 | 840.029768507 | 1001.3351134846461 | 0.0694408428763 |
| 995.6853634251576 | 4.90922620398 | 995.6853634251576 | 863.946370575 | 995.6853634251576 | 0.0859172484884 |
| 990.0990099009902 | 5.62303214978 | 990.0990099009902 | 887.031678074 | 990.0990099009902 | 0.106092100925 |
| 984.5749917952082 | 6.42784585475 | 984.5749917952082 | 909.214922167 | 984.5749917952082 | 0.130744386716 |
| 979.1122715404699 | 7.33327060164 | 979.1122715404699 | 930.43417224 | 979.1122715404699 | 0.160805303377 |
| 973.7098344693281 | 8.34963222895 | 973.7098344693281 | 950.637528045 | 973.7098344693281 | 0.197385386409 |
| 968.3666881859263 | 9.48799330414 | 968.3666881859263 | 969.784271736 | 968.3666881859263 | 0.241805924645 |
| 963.0818619582664 | 10.7601614801 | 963.0818619582664 | 987.845965635 | 963.0818619582664 | 0.295635233776 |
| 957.8544061302682 | 12.1786911252 | 957.8544061302682 | 1004.80748174 | 957.8544061302682 | 0.360730413273 |
| 952.6833915528738 | 13.7568772921 | 952.6833915528738 | 1020.66794937 | 952.6833915528738 | 0.439285268804 |
| 947.5679090334806 | 15.508741078 | 947.5679090334806 | 1035.44160777 | 947.5679090334806 | 0.53388513998 |
| 942.5070688030161 | 17.449005432 | 942.5070688030161 | 1049.15855136 | 942.5070688030161 | 0.647569430851 |
| 937.4999999999999 | 19.5930604809 | 937.4999999999999 | 1061.86535582 | 937.4999999999999 | 0.783902696811 |
| 932.5458501709667 | 21.956917484 | 932.5458501709667 | 1073.62557453 | 932.5458501709667 | 0.947055195101 |
| 927.643784786642 | 24.5571505818 | 927.643784786642 | 1084.52009572 | 927.643784786642 | 1.1418938553 |
| 922.7929867733004 | 27.4108255817 | 922.7929867733004 | 1094.64735183 | 922.7929867733004 | 1.37408466908 |
| 917.9926560587514 | 30.5354151213 | 917.9926560587514 | 1104.12337406 | 917.9926560587514 | 1.650207533 |
| 913.2420091324201 | 33.9486996774 | 913.2420091324201 | 1113.08168624 | 913.2420091324201 | 1.9778846018 |
| 908.5402786190186 | 37.6686540296 | 908.5402786190186 | 1121.67303352 | 908.5402786190186 | 2.36592321951 |
| 903.8867128653209 | 41.7133189634 | 903.8867128653209 | 1130.06494274 | 903.8867128653209 | 2.82447448962 |
| 899.2805755395683 | 46.1006581877 | 899.2805755395683 | 1138.44111307 | 899.2805755395683 | 3.3652085188 |
| 894.7211452430658 | 50.8484006599 | 894.7211452430658 | 1147.00063624 | 894.7211452430658 | 4.00150731967 |
| 890.2077151335311 | 55.9738687493 | 890.2077151335311 | 1155.95704789 | 890.2077151335311 | 4.74867628182 |
| 885.7395925597874 | 61.4937929233 | 885.7395925597874 | 1165.5372121 | 885.7395925597874 | 5.62417501344 |
| 881.316098707403 | 67.4241139154 | 881.316098707403 | 1175.98004309 | 881.316098707403 | 6.64786821521 |
| 876.9365682548962 | 73.7797736182 | 876.9365682548962 | 1187.53506912 | 876.9365682548962 | 7.84229706889 |
| 872.6003490401396 | 80.5744962362 | 872.6003490401396 | 1200.46084454 | 872.6003490401396 | 9.23297140226 |
| 868.3068017366135 | 87.8205615332 | 868.3068017366135 | 1215.02321782 | 868.3068017366135 | 10.8486826263 |
| 864.0552995391705 | 95.5285723043 | 864.0552995391705 | 1231.49346369 | 864.0552995391705 | 12.7218371263 |
| 859.8452278589854 | 103.707218494 | 859.8452278589854 | 1250.14628917 | 859.8452278589854 | 14.888809423 |
| 855.6759840273816 | 112.363040657 | 855.6759840273816 | 1271.25772425 | 855.6759840273816 | 17.3903140035 |
| 851.5469770082316 | 121.500195726 | 851.5469770082316 | 1295.10290855 | 851.5469770082316 | 20.2717942469 |
| 847.457627118644 | 131.120228268 | 847.457627118644 | 1321.95378691 | 847.457627118644 | 23.5838263466 |
| 843.4073657576608 | 141.221850644 | 843.4073657576608 | 1352.07672744 | 843.4073657576608 | 27.382535548 |
| 839.3956351426972 | 151.800735614 | 839.3956351426972 | 1385.73007641 | 839.3956351426972 | 31.7300213922 |
| 835.421888053467 | 162.849325091 | 835.421888053467 | 1423.16166584 | 835.421888053467 | 36.6947879737 |
| 831.4855875831485 | 174.356658795 | 831.4855875831485 | 1464.60628996 | 831.4855875831485 | 42.3521745035 |
| 827.5862068965516 | 186.308226584 | 827.5862068965516 | 1510.28316815 | 827.5862068965516 | 48.7847807058 |
| 823.7232289950576 | 198.685848207 | 823.7232289950576 | 1560.39341258 | 823.7232289950576 | 56.0828807986 |
| 819.8961464881114 | 211.467584116 | 819.8961464881114 | 1615.11751989 | 819.8961464881114 | 64.3448190053 |
| 816.1044613710554 | 224.627680811 | 816.1044613710554 | 1674.61290711 | 816.1044613710554 | 73.6773787425 |
| 812.3476848090983 | 238.136553961 | 812.3476848090983 | 1739.01151274 | 812.3476848090983 | 84.1961168403 |
| 808.6253369272237 | 251.960812248 | 808.6253369272237 | 1808.41748481 | 808.6253369272237 | 96.0256533879 |
| 804.9369466058491 | 266.063324524 | 804.9369466058491 | 1882.90497857 | 804.9369466058491 | 109.299907084 |
| 801.2820512820513 | 280.403332436 | 801.2820512820513 | 1962.51608669 | 801.2820512820513 | 124.162265326 |
| 797.6601967561818 | 294.936610217 | 797.6601967561818 | 2047.25892576 | 797.6601967561818 | 140.765677719 |
| 794.0709370037056 | 309.615672781 | 794.0709370037056 | 2137.10590276 | 794.0709370037056 | 159.272661236 |
| 790.5138339920949 | 324.390032713 | 790.5138339920949 | 2231.99218553 | 790.5138339920949 | 179.855204973 |
| 786.9884575026232 | 339.206506096 | 786.9884575026232 | 2331.81440086 | 786.9884575026232 | 202.694562284 |
| 783.4943849569078 | 354.009566508 | 783.4943849569078 | 2436.42958364 | 783.4943849569078 | 227.980918145 |
| 780.0312012480499 | 368.741745848 | 780.0312012480499 | 2545.65439943 | 780.0312012480499 | 255.912919833 |
| 776.598498576236 | 383.344079962 | 776.598498576236 | 2659.264662 | 776.598498576236 | 286.697059532 |
| 773.1958762886597 | 397.756596429 | 773.1958762886597 | 2776.99516573 | 773.1958762886597 | 320.546898177 |
| 769.8229407236336 | 411.918841163 | 769.8229407236336 | 2898.53985094 | 769.8229407236336 | 357.68212091 |
| 766.4793050587633 | 425.770439916 | 766.4793050587633 | 3023.55231804 | 766.4793050587633 | 398.327415809 |
| 763.1645891630628 | 439.251690164 | 763.1645891630628 | 3151.6467036 | 763.1645891630628 | 442.711169135 |
| 759.8784194528876 | 452.304178361 | 759.8784194528876 | 3282.39892849 | 759.8784194528876 | 491.063972288 |
| 756.6204287515762 | 464.871417048 | 756.6204287515762 | 3415.34832468 | 756.6204287515762 | 543.616937824 |
| 753.390256152687 | 476.899495967 | 753.390256152687 | 3549.99964344 | 753.390256152687 | 600.59982441 |
| 750.1875468867216 | 488.337740977 | 750.1875468867216 | 3685.8254437 | 750.1875468867216 | 662.238973376 |
| 747.011952191235 | 499.139374385 | 747.011952191235 | 3822.2688546 | 747.011952191235 | 728.755062537 |
| 743.86312918423 | 509.262170166 | 743.86312918423 | 3958.74670174 | 743.86312918423 | 800.360686254 |
| 740.7407407407408 | 518.669097524 | 740.7407407407408 | 4094.65298171 | 740.7407407407408 | 877.25777415 |
| 737.6444553725104 | 527.328946312 | 737.6444553725104 | 4229.36266447 | 737.6444553725104 | 959.634864501 |
| 734.5739471106758 | 535.216928012 | 734.5739471106758 | 4362.23579826 | 734.5739471106758 | 1047.66425205 |
| 731.528895391368 | 542.315246218 | 731.528895391368 | 4492.62188653 | 731.528895391368 | 1141.4990337 |
| 728.5089849441475 | 548.613630946 | 728.5089849441475 | 4619.86450202 | 728.5089849441475 | 1241.27007929 |
| 725.5139056831922 | 554.109831489 | 725.5139056831922 | 4743.30609808 | 725.5139056831922 | 1347.08295821 |
| 722.543352601156 | 558.810063101 | 722.543352601156 | 4862.29297361 | 722.543352601156 | 1459.01485614 |
| 719.5970256656271 | 562.72940331 | 719.5970256656271 | 4976.18034383 | 719.5970256656271 | 1577.11151922 |
| 716.6746297181079 | 565.892134302 | 716.6746297181079 | 5084.33746634 | 716.6746297181079 | 1701.38426592 |
| 713.7758743754462 | 568.332028506 | 713.7758743754462 | 5186.15276879 | 713.7758743754462 | 1831.80710924 |
| 710.9004739336492 | 570.09257512 | 710.9004739336492 | 5281.03892309 | 710.9004739336492 | 1968.31403375 |
| 708.0481472740146 | 571.227146079 | 708.0481472740146 | 5368.43780945 | 708.0481472740146 | 2110.79647327 |
| 705.2186177715091 | 571.799100587 | 705.2186177715091 | 5447.82531361 | 705.2186177715091 | 2259.10103577 |
| 702.4116132053383 | 571.881828016 | 702.4116132053383 | 5518.71590077 | 702.4116132053383 | 2413.02752199 |
| 699.6268656716418 | 571.558729571 | 699.6268656716418 | 5580.66691128 | 699.6268656716418 | 2572.32728332 |
| 696.8641114982578 | 570.923139686 | 696.8641114982578 | 5633.2825252 | 696.8641114982578 | 2736.70196317 |
| 694.1230911614992 | 570.078188605 | 694.1230911614992 | 5676.21734626 | 694.1230911614992 | 2905.8026633 |
| 691.4035492048858 | 569.136608007 | 691.4035492048858 | 5709.17955906 | 691.4035492048858 | 3079.22957347 |
| 688.7052341597796 | 568.220481859 | 688.7052341597796 | 5731.93361882 | 688.7052341597796 | 3256.5320986 |
| 686.027898467871 | 567.460944915 | 686.027898467871 | 5744.30243769 | 686.027898467871 | 3437.20951262 |
| 683.371298405467 | 566.997831388 | 683.371298405467 | 5746.16903824 | 683.371298405467 | 3620.71216246 |
| 680.7351940095303 | 566.979276374 | 680.7351940095303 | 5737.47765093 | 680.7351940095303 | 3806.44323926 |
| 678.1193490054249 | 567.561272478 | 678.1193490054249 | 5718.23423985 | 678.1193490054249 | 3993.76112656 |
| 675.5235307363206 | 568.907183969 | 675.5235307363206 | 5688.506448 | 675.5235307363206 | 4181.98232785 |
| 672.9475100942127 | 571.187220472 | 672.9475100942127 | 5648.42296124 | 672.9475100942127 | 4370.38496737 |
| 670.3910614525139 | 574.577871873 | 670.3910614525139 | 5598.17229753 | 670.3910614525139 | 4558.21285009 |
| 667.8539626001781 | 579.261305667 | 667.8539626001781 | 5538.00103585 | 667.8539626001781 | 4744.68005773 |
| 665.335994677312 | 585.424727499 | 665.335994677312 | 5468.21150634 | 665.335994677312 | 4928.97604935 |
| 662.8369421122403 | 593.259705112 | 662.8369421122403 | 5389.15897053 | 662.8369421122403 | 5110.27122649 |
| 660.3565925599823 | 602.961455374 | 660.3565925599823 | 5301.24832704 | 660.3565925599823 | 5287.72291434 |
| 657.8947368421053 | 614.728093475 | 657.8947368421053 | 5204.93038415 | 657.8947368421053 | 5460.48170313 |
| 655.4511688879178 | 628.759842893 | 655.4511688879178 | 5100.69774603 | 655.4511688879178 | 5627.69808627 |
| 653.0256856769699 | 645.258204191 | 653.0256856769699 | 4989.08036437 | 653.0256856769699 | 5788.52932561 |
| 650.6180871828237 | 664.425080314 | 650.6180871828237 | 4870.64081043 | 650.6180871828237 | 5942.14646874 |
| 648.2281763180639 | 686.461855713 | 648.2281763180639 | 4745.96932587 | 648.2281763180639 | 6087.74143851 |
| 645.8557588805166 | 711.568426398 | 645.8557588805166 | 4615.67871265 | 645.8557588805166 | 6224.53411194 |
| 643.5006435006435 | 739.942177941 | 643.5006435006435 | 4480.39912309 | 643.5006435006435 | 6351.77930333 |
| 641.1626415900834 | 771.776908513 | 641.1626415900834 | 4340.77281163 | 641.1626415900834 | 6468.77356576 |
| 638.8415672913118 | 807.261694283 | 638.8415672913118 | 4197.44890864 | 638.8415672913118 | 6574.86172579 |
| 636.5372374283895 | 846.579694939 | 636.5372374283895 | 4051.07827517 | 636.5372374283895 | 6669.44306789 |
| 634.2494714587738 | 889.906897699 | 634.2494714587738 | 3902.30849468 | 634.2494714587738 | 6751.97708886 |
| 631.9780914261638 | 937.410799021 | 631.9780914261638 | 3751.77905456 | 631.9780914261638 | 6821.98874678 |
| 629.7229219143577 | 989.249024253 | 629.7229219143577 | 3600.11676611 | 629.7229219143577 | 6879.07313529 |
| 627.4837900020916 | 1045.5678867 | 627.4837900020916 | 3447.93146686 | 627.4837900020916 | 6922.89952097 |
| 625.2605252188412 | 1106.50088899 | 625.2605252188412 | 3295.81204395 | 625.2605252188412 | 6953.21468987 |
| 623.0529595015576 | 1172.16717137 | 623.0529595015576 | 3144.32281166 | 623.0529595015576 | 6969.84555844 |
| 620.8609271523178 | 1242.66991309 | 620.8609271523178 | 2994.00027015 | 620.8609271523178 | 6972.70101398 |
| 618.6842647968654 | 1318.09469521 | 618.6842647968654 | 2845.35026641 | 618.6842647968654 | 6961.77296037 |
| 616.5228113440197 | 1398.50783512 | 616.5228113440197 | 2698.84557215 | 616.5228113440197 | 6937.13655564 |
| 614.3764079459348 | 1483.95470501 | 614.3764079459348 | 2554.92388734 | 614.3764079459348 | 6898.94963948 |
| 612.2448979591836 | 1574.45804897 | 612.2448979591836 | 2413.98627177 | 612.2448979591836 | 6847.45135952 |
| 610.1281269066504 | 1670.0163152 | 610.1281269066504 | 2276.39600173 | 610.1281269066504 | 6782.96001674 |
| 608.0259424402108 | 1770.60202209 | 608.0259424402108 | 2142.4778428 | 608.0259424402108 | 6705.87016071 |
| 605.9381943041809 | 1876.16017888 | 605.9381943041809 | 2012.51772522 | 605.9381943041809 | 6616.6489756 |
| 603.864734299517 | 1986.6067832 | 603.864734299517 | 1886.76280352 | 603.864734299517 | 6515.83200709 |
| 601.8054162487462 | 2101.82741961 | 601.8054162487462 | 1765.42187786 | 601.8054162487462 | 6404.01828904 |
| 599.7600959616153 | 2221.67598439 | 599.7600959616153 | 1648.66615138 | 599.7600959616153 | 6281.86493586 |
| 597.7286312014345 | 2345.97356281 | 597.7286312014345 | 1536.63029463 | 597.7286312014345 | 6150.08127284 |
| 595.7108816521048 | 2474.50748569 | 595.7108816521048 | 1429.41378601 | 595.7108816521048 | 6009.42258177 |
| 593.7067088858104 | 2607.0305923 | 593.7067088858104 | 1327.08249545 | 593.7067088858104 | 5860.68354256 |
| 591.7159763313609 | 2743.26072615 | 591.7159763313609 | 1229.67047748 | 591.7159763313609 | 5704.69145408 |
| 589.7385492431688 | 2882.88048954 | 589.7385492431688 | 1137.18193925 | 589.7385492431688 | 5542.29931798 |
| 587.7742946708463 | 3025.53728142 | 587.7742946708463 | 1049.59334913 | 587.7742946708463 | 5374.37886904 |
| 585.8230814294083 | 3170.843641 | 585.8230814294083 | 966.855652272 | 585.8230814294083 | 5201.81363346 |
| 583.8847800700661 | 3318.37791737 | 583.8847800700661 | 888.89656021 | 583.8847800700661 | 5025.49209364 |
| 581.9592628516003 | 3467.68528222 | 581.9592628516003 | 815.622883297 | 581.9592628516003 | 4846.30103356 |
| 580.046403712297 | 3618.27909932 | 580.046403712297 | 746.922876462 | 580.046403712297 | 4665.11913347 |
| 578.1460782424359 | 3769.64266037 | 578.1460782424359 | 682.668570909 | 578.1460782424359 | 4482.81087668 |
| 576.2581636573184 | 3921.23129225 | 576.2581636573184 | 622.718066741 | 576.2581636573184 | 4300.22082369 |
| 574.3825387708214 | 4072.47483617 | 574.3825387708214 | 566.91776403 | 574.3825387708214 | 4118.16830175 |
| 572.5190839694656 | 4222.78049349 | 572.5190839694656 | 515.104512549 | 572.5190839694656 | 3937.44254941 |
| 570.6676811869887 | 4371.53602807 | 570.6676811869887 | 467.107663093 | 570.6676811869887 | 3758.79834762 |
| 568.8282138794084 | 4518.1133089 | 568.8282138794084 | 422.751006116 | 568.8282138794084 | 3582.9521599 |
| 567.000567000567 | 4661.87217138 | 567.000567000567 | 381.854586126 | 567.000567000567 | 3410.57879632 |
| 565.1846269781461 | 4802.16456981 | 565.1846269781461 | 344.236382958 | 565.1846269781461 | 3242.30860704 |
| 563.3802816901408 | 4938.33898814 | 563.3802816901408 | 309.713853586 | 563.3802816901408 | 3078.72520382 |
| 561.5874204417821 | 5069.7450707 | 561.5874204417821 | 278.10533057 | 561.5874204417821 | 2920.3637001 |
| 559.8059339428997 | 5195.73842981 | 559.8059339428997 | 249.231275449 | 559.8059339428997 | 2767.7094537 |
| 558.0357142857143 | 5315.68558256 | 558.0357142857143 | 222.915387455 | 558.0357142857143 | 2621.19728978 |
| 556.2766549230483 | 5428.96896537 | 556.2766549230483 | 198.98556978 | 556.2766549230483 | 2481.21117672 |
| 554.52865064695 | 5534.99197151 | 554.52865064695 | 177.274757211 | 554.52865064695 | 2348.08432267 |
| 552.791597567717 | 5633.18395443 | 552.791597567717 | 157.621610399 | 552.791597567717 | 2222.09965729 |
| 551.0653930933137 | 5723.00513801 | 551.0653930933137 | 139.871083176 | 551.0653930933137 | 2103.49066035 |
| 549.3499359091741 | 5803.95137437 | 549.3499359091741 | 123.8748703 | 549.3499359091741 | 1992.44249693 |
| 547.645125958379 | 5875.55868954 | 547.645125958379 | 109.491743763 | 547.645125958379 | 1889.09341851 |
| 545.950864422202 | 5937.40755904 | 545.950864422202 | 96.5877863215 | 545.950864422202 | 1793.53638855 |
| 544.2670537010159 | 5989.12685713 | 544.2670537010159 | 85.0365313029 | 544.2670537010159 | 1705.82089272 |
| 542.5935973955508 | 6030.3974266 | 542.5935973955508 | 74.7190178712 | 542.5935973955508 | 1625.9548946 |
| 540.9304002884962 | 6060.95522013 | 540.9304002884962 | 65.5237710126 | 540.9304002884962 | 1553.90690064 |
| 539.2773683264425 | 6080.59396903 | 539.2773683264425 | 57.3467153538 | 539.2773683264425 | 1489.6081002 |
| 537.6344086021505 | 6089.16734062 | 537.6344086021505 | 50.0910317056 | 537.6344086021505 | 1432.9545502 |
| 536.0014293371448 | 6086.59055221 | 536.0014293371448 | 43.6669648834 | 536.0014293371448 | 1383.80937716 |
| 534.3783398646241 | 6072.84141645 | 534.3783398646241 | 37.9915909362 | 534.3783398646241 | 1342.00497339 |
| 532.7650506126798 | 6047.96080024 | 532.7650506126798 | 32.9885514223 | 532.7650506126798 | 1307.34516792 |
| 531.1614730878186 | 6012.05248736 | 531.1614730878186 | 28.5877618339 | 531.1614730878186 | 1279.60735701 |
| 529.5675198587819 | 5965.28244289 | 529.5675198587819 | 24.7251006934 | 529.5675198587819 | 1258.54458279 |
| 527.9831045406547 | 5907.8774859 | 527.9831045406547 | 21.3420852477 | 527.9831045406547 | 1243.88755261 |
| 526.4081417792595 | 5840.1233846 | 526.4081417792595 | 18.3855390812 | 526.4081417792595 | 1235.34659523 |
| 524.8425472358292 | 5762.36239634 | 524.8425472358292 | 15.8072563612 | 524.8425472358292 | 1232.61355321 |
| 523.2862375719518 | 5674.99028235 | 523.2862375719518 | 13.5636668411 | 523.2862375719518 | 1235.36361395 |
| 521.7391304347826 | 5578.45283394 | 521.7391304347826 | 11.6155051703 | 521.7391304347826 | 1243.25708414 |
| 520.2011444425177 | 5473.24195356 | 520.2011444425177 | 9.92748751748 | 520.2011444425177 | 1255.94111464 |
| 518.6721991701245 | 5359.89133978 | 518.6721991701245 | 8.46799799761 | 518.6721991701245 | 1273.05138415 |
| 517.1522151353215 | 5238.97183026 | 517.1522151353215 | 7.20878691882 | 517.1522151353215 | 1294.21375129 |
| 515.6411137848057 | 5111.08646052 | 515.6411137848057 | 6.12468242257 | 515.6411137848057 | 1319.04588501 |
| 514.1388174807198 | 4976.86529974 | 514.1388174807198 | 5.19331669508 | 514.1388174807198 | 1347.15888356 |
| 512.6452494873547 | 4836.96012684 | 512.6452494873547 | 4.39486756878 | 512.6452494873547 | 1378.15889149 |
| 511.1603339580848 | 4692.03901074 | 511.1603339580848 | 3.71181601659 | 511.1603339580848 | 1411.64872354 |
| 509.683995922528 | 4542.78085949 | 509.683995922528 | 3.1287197649 | 509.683995922528 | 1447.22950287 |
| 508.2161612739285 | 4389.87000118 | 508.2161612739285 | 2.63200301308 | 508.2161612739285 | 1484.50231931 |
| 506.7567567567567 | 4233.9908583 | 506.7567567567567 | 2.20976204535 | 506.7567567567567 | 1523.06991159 |
| 505.3057099545225 | 4075.8227739 | 505.3057099545225 | 1.85158635392 | 505.3057099545225 | 1562.53837526 |
| 503.8629492777964 | 3916.03504458 | 503.8629492777964 | 1.5483947558 | 503.8629492777964 | 1602.51889551 |
| 502.4284039524367 | 3755.28221071 | 502.4284039524367 | 1.29228587939 | 502.4284039524367 | 1642.62950191 |
| 501.00200400801606 | 3594.19964933 | 501.00200400801606 | 1.07640231556 | 501.00200400801606 | 1682.49683947 |
| 499.5836802664446 | 3433.39950956 | 499.5836802664446 | 0.894807670445 | 499.5836802664446 | 1721.75794822 |
| 498.1733643307871 | 3273.46702436 | 498.1733643307871 | 0.74237571965 | 498.1733643307871 | 1760.06204076 |
| 496.7709885742673 | 3114.9572261 | 496.7709885742673 | 0.61469084412 | 496.7709885742673 | 1797.07226587 |
| 495.3764861294584 | 2958.39208695 | 495.3764861294584 | 0.507958922894 | 495.3764861294584 | 1832.46744353 |
| 493.98979087765514 | 2804.2580986 | 493.98979087765514 | 0.418927866197 | 493.98979087765514 | 1865.94375596 |
| 492.61083743842363 | 2653.00429907 | 492.61083743842363 | 0.344816990474 | 492.61083743842363 | 1897.21637726 |
| 491.2395611593253 | 2505.04074838 | 491.2395611593253 | 0.283254463657 | 491.2395611593253 | 1926.02102399 |
| 489.8758981058131 | 2360.73744854 | 489.8758981058131 | 0.23222208194 | 489.8758981058131 | 1952.11540822 |
| 488.5197850512946 | 2220.42369757 | 488.5197850512946 | 0.190006677262 | 488.5197850512946 | 1975.28057434 |
| 487.17115946735953 | 2084.38786235 | 487.17115946735953 | 0.155157495837 | 487.17115946735953 | 1995.32210184 |
| 485.82995951416996 | 1952.87754971 | 485.82995951416996 | 0.126448931412 | 485.82995951416996 | 2012.07115626 |
| 484.49612403100775 | 1826.1001518 | 484.49612403100775 | 0.10284804121 | 484.49612403100775 | 2025.38537233 |
| 483.16959252697694 | 1704.22373736 | 483.16959252697694 | 0.0834863170197 | 483.16959252697694 | 2035.14955442 |
| 481.8503051718599 | 1587.37825821 | 481.8503051718599 | 0.067635227677 | 481.8503051718599 | 2041.27618143 |
| 480.5382027871216 | 1475.65703762 | 480.5382027871216 | 0.0546850918873 | 480.5382027871216 | 2043.70570553 |
| 479.23322683706067 | 1369.11850575 | 479.23322683706067 | 0.0441268812871 | 479.23322683706067 | 2042.40663633 |
| 477.9353194201051 | 1267.7881461 | 477.9353194201051 | 0.0355365926504 | 477.9353194201051 | 2037.37540509 |
| 476.64442326024783 | 1171.6606169 | 476.64442326024783 | 0.0285618648759 | 476.64442326024783 | 2028.63600602 |
| 475.3604816986214 | 1080.70201107 | 475.3604816986214 | 0.022910550718 | 475.3604816986214 | 2016.23941484 |
| 474.08343868520853 | 994.852219508 | 474.08343868520853 | 0.0183409850594 | 474.08343868520853 | 2000.26278775 |
| 472.8132387706856 | 914.02736336 | 472.8132387706856 | 0.0146537208366 | 472.8132387706856 | 1980.80844668 |
| 471.5498270983967 | 838.12226274 | 471.5498270983967 | 0.0116845305452 | 471.5498270983967 | 1958.00265978 |
| 470.29314939645707 | 767.012911271 | 470.29314939645707 | 0.00929849563544 | 470.29314939645707 | 1931.99422877 |
| 469.04315196998124 | 700.558928161 | 469.04315196998124 | 0.00738502815826 | 469.04315196998124 | 1902.95289715 |
| 467.7997816934352 | 638.605962061 | 467.7997816934352 | 0.00585368883916 | 467.7997816934352 | 1871.06759578 |
| 466.5629860031104 | 580.988023721 | 466.5629860031104 | 0.00463068349711 | 466.5629860031104 | 1836.5445444 |
| 465.33271288971605 | 527.529727255 | 465.33271288971605 | 0.00365593550829 | 465.33271288971605 | 1799.60522915 |
| 464.10891089108907 | 478.048422824 | 464.10891089108907 | 0.0028806460036 | 464.10891089108907 | 1760.48427788 |
| 462.8915290850177 | 432.356206401 | 462.8915290850177 | 0.00226526582526 | 462.8915290850177 | 1719.4272559 |
| 461.68051708217905 | 390.261795201 | 461.68051708217905 | 0.00177781410202 | 461.68051708217905 | 1676.68840554 |
| 460.47582501918646 | 351.572260159 | 460.47582501918646 | 0.00139248777667 | 460.47582501918646 | 1632.52835336 |
| 459.2774035517452 | 316.094609483 | 459.2774035517452 | 0.00108851467076 | 459.2774035517452 | 1587.21180868 |
| 458.0852038479157 | 283.63721985 | 458.0852038479157 | 0.000849209829096 | 458.0852038479157 | 1541.00527689 |
| 456.89917758148033 | 254.011114092 | 456.89917758148033 | 0.000661201071654 | 456.89917758148033 | 1494.17481009 |
| 455.7192769254139 | 227.031086383 | 455.7192769254139 | 0.000513795005091 | 455.7192769254139 | 1446.98381695 |
| 454.54545454545456 | 202.516677777 | 454.54545454545456 | 0.000398459312915 | 454.54545454545456 | 1399.69095203 |
| 453.3776635937736 | 180.293006642 | 453.3776635937736 | 0.000308401046518 | 453.3776635937736 | 1352.54810356 |
| 452.2158577027434 | 160.191459921 | 452.2158577027434 | 0.000238223963175 | 452.2158577027434 | 1305.79849693 |
| 451.05999097880016 | 142.050252361 | 451.05999097880016 | 0.000183650778143 | 451.05999097880016 | 1259.67492911 |
| 449.9100179964007 | 125.714861781 | 449.9100179964007 | 0.000141298584086 | 449.9100179964007 | 1214.39814728 |
| 448.7658937920718 | 111.038349181 | 448.7658937920718 | 0.00010849770253 | 448.7658937920718 | 1170.17538301 |
| 447.6275738585497 | 97.881573024 | 447.6275738585497 | 8.31459222307e-05 | 447.6275738585497 | 1127.19905092 |
| 446.49501413900873 | 86.1133073205 | 446.49501413900873 | 6.35914949171e-05 | 446.49501413900873 | 1085.64561875 |
| 445.36817102137763 | 75.6102733133 | 445.36817102137763 | 4.85394407439e-05 | 445.36817102137763 | 1045.67465368 |
| 444.247001332741 | 66.2570945166 | 444.247001332741 | 3.6976699428e-05 | 444.247001332741 | 1007.42804745 |
| 443.13146233382565 | 57.946184719 | 443.13146233382565 | 2.81124791817e-05 | 443.13146233382565 | 971.029421171 |
| 442.02151171357 | 50.5775782654 | 442.02151171357 | 2.13308306516e-05 | 442.02151171357 | 936.583708458 |
| 440.9171075837742 | 44.0587115522 | 440.9171075837742 | 1.61530298124e-05 | 440.9171075837742 | 904.176914126 |
| 439.8182084738308 | 38.3041641923 | 439.8182084738308 | 1.22078115461e-05 | 439.8182084738308 | 873.876043811 |
| 438.72477332553376 | 33.2353677722 | 438.72477332553376 | 9.20787092172e-06 | 438.72477332553376 | 845.729198593 |
| 437.636761487965 | 28.7802895353 | 437.636761487965 | 6.93135596886e-06 | 437.636761487965 | 819.765827332 |
| 436.5541327124563 | 24.8730977059 | 436.5541327124563 | 5.20732577475e-06 | 436.5541327124563 | 795.997128344 |
| 435.4768471476266 | 21.4538145283 | 435.4768471476266 | 3.90435095579e-06 | 435.4768471476266 | 774.416591076 |
| 434.4048653344918 | 18.4679624545 | 434.4048653344918 | 2.92159824795e-06 | 434.4048653344918 | 755.000667652 |
| 433.3381482016467 | 15.8662082725 | 433.3381482016467 | 2.18187406264e-06 | 433.3381482016467 | 737.709563509 |
| 432.2766570605187 | 13.6040093469 | 432.2766570605187 | 1.62620912224e-06 | 432.2766570605187 | 722.488135881 |
| 431.22035360069 | 11.6412655441 | 431.22035360069 | 1.2096525001e-06 | 431.22035360069 | 709.266888478 |
| 430.1691998852882 | 9.94197984565 | 430.1691998852882 | 8.98012486065e-07 | 430.1691998852882 | 697.963050537 |
| 429.1231583464454 | 8.47393012006 | 429.1231583464454 | 6.6533692842e-07 | 429.1231583464454 | 688.481728222 |
| 428.0821917808219 | 7.20835403134 | 428.0821917808219 | 4.91969728982e-07 | 428.0821917808219 | 680.717116392 |
| 427.0462633451957 | 6.11964860863 | 427.0462633451957 | 3.63055169978e-07 | 427.0462633451957 | 674.553758773 |
| 426.01533655211585 | 5.18508559425 | 426.01533655211585 | 2.67389503574e-07 | 426.01533655211585 | 669.867844708 |
| 424.9893752656184 | 4.38454332166 | 424.9893752656184 | 1.96541181759e-07 | 424.9893752656184 | 666.52853088 |
| 423.96834369700395 | 3.70025555327 | 423.96834369700395 | 1.44178413942e-07 | 423.96834369700395 | 664.399276642 |
| 422.9522064006767 | 3.11657742829 | 422.9522064006767 | 1.05556356432e-07 | 422.9522064006767 | 663.339181893 |
| 421.9409282700422 | 2.61976843091 | 421.9409282700422 | 7.71269218747e-08 | 421.9409282700422 | 663.204316802 |
| 420.93447453346425 | 2.19779208799 | 420.93447453346425 | 5.62425577053e-08 | 420.93447453346425 | 663.849033059 |
| 419.9328107502799 | 1.84013193879 | 419.9328107502799 | 4.09318690598e-08 | 419.9328107502799 | 665.127246776 |
| 418.93590280687056 | 1.53762318639 | 418.93590280687056 | 2.97300428187e-08 | 418.93590280687056 | 666.893683595 |
| 417.94371691278906 | 1.28229933607 | 417.94371691278906 | 2.1550975505e-08 | 417.94371691278906 | 669.005077109 |
| 416.9562195969423 | 1.06725304924 | 416.9562195969423 | 1.55910644627e-08 | 416.9562195969423 | 671.32131218 |
| 415.97337770382694 | 0.886510387612 | 415.97337770382694 | 1.12569835598e-08 | 415.97337770382694 | 673.706505362 |
| 414.99515838981876 | 0.734917589497 | 414.99515838981876 | 8.11158498874e-09 | 414.99515838981876 | 676.03001523 |
| 414.0215291195142 | 0.608039505075 | 414.0215291195142 | 5.83346821694e-09 | 414.0215291195142 | 678.167376062 |
| 413.0524576621231 | 0.502068817233 | 413.0524576621231 | 4.18683038431e-09 | 413.0524576621231 | 680.001149038 |
| 412.08791208791206 | 0.413745187201 | 412.08791208791206 | 2.99903346997e-09 | 412.08791208791206 | 681.421685843 |
| 411.1278607646978 | 0.340283486877 | 411.1278607646978 | 2.14395007719e-09 | 411.1278607646978 | 682.32780034 |
| 410.17227235438884 | 0.279310310598 | 410.17227235438884 | 1.52962661276e-09 | 410.17227235438884 | 682.627344804 |
| 409.22111580957574 | 0.228807996151 | 409.22111580957574 | 1.08916479882e-09 | 409.22111580957574 | 682.237688052 |
| 408.2743603701687 | 0.187065426408 | 408.2743603701687 | 7.73996795203e-10 | 408.2743603701687 | 681.086093689 |
| 407.33197556008145 | 0.152634927646 | 407.33197556008145 | 5.48936520077e-10 | 407.33197556008145 | 679.109997596 |
| 406.39393118396094 | 0.124294627058 | 406.39393118396094 | 3.88546034347e-10 | 406.39393118396094 | 676.257184725 |
| 405.46019732396263 | 0.101015679184 | 405.46019732396263 | 2.74473377936e-10 | 405.46019732396263 | 672.485866172 |
| 404.53074433656957 | 0.0819338180105 | 404.53074433656957 | 1.93506412444e-10 | 404.53074433656957 | 667.764658457 |
| 403.6055428494551 | 0.0663247376834 | 403.6055428494551 | 1.36153208557e-10 | 403.6055428494551 | 662.072467837 |
| 402.68456375838923 | 0.0535828494609 | 402.68456375838923 | 9.56087810948e-11 | 402.68456375838923 | 655.398283394 |
| 401.76777822418643 | 0.0432030053387 | 401.76777822418643 | 6.70046683991e-11 | 401.76777822418643 | 647.740883476 |
| 400.85515766969536 | 0.0347648193532 | 400.85515766969536 | 4.68651185717e-11 | 400.85515766969536 | 639.108460899 |
| 399.9466737768297 | 0.0279192556701 | 399.9466737768297 | 3.27138571404e-11 | 399.9466737768297 | 629.518173039 |
| 399.0422984836393 | 0.0223771880753 | 399.0422984836393 | 2.27903585559e-11 | 399.0422984836393 | 618.99562365 |
| 398.14200398142003 | 0.0178996683291 | 398.14200398142003 | 1.584557244e-11 | 398.14200398142003 | 607.574283822 |
| 397.24576271186436 | 0.0142896710073 | 397.24576271186436 | 1.09951732429e-11 | 397.24576271186436 | 595.294859975 |
| 396.3535473642489 | 0.011385109993 | 396.3535473642489 | 7.61436315649e-12 | 396.3535473642489 | 582.204617227 |
| 395.46533087266016 | 0.00905294676468 | 395.46533087266016 | 5.26262542453e-12 | 395.46533087266016 | 568.356666716 |
| 394.5810864132579 | 0.00718423316663 | 394.5810864132579 | 3.63001730315e-12 | 394.5810864132579 | 553.809225682 |
| 393.7007874015748 | 0.00568995158046 | 393.7007874015748 | 2.49891964461e-12 | 393.7007874015748 | 538.624859139 |
| 392.82440748985204 | 0.00449753347707 | 392.82440748985204 | 1.7168535297e-12 | 392.82440748985204 | 522.86971195 |
| 391.9519205644107 | 0.00354795338201 | 391.9519205644107 | 1.17720353365e-12 | 391.9519205644107 | 506.612739928 |
| 391.08330074305826 | 0.00279330948317 | 391.08330074305826 | 8.0557730695e-13 | 391.08330074305826 | 489.924948331 |
| 390.2185223725286 | 0.00219481461179 | 390.2185223725286 | 5.50174229532e-13 | 390.2185223725286 | 472.878645722 |
| 389.3575600259571 | 0.00172113228758 | 389.3575600259571 | 3.74999437535e-13 | 389.3575600259571 | 455.546720703 |
| 388.5003885003885 | 0.00134700208932 | 388.5003885003885 | 2.55092881399e-13 | 388.5003885003885 | 438.001948455 |
| 387.6469828143171 | 0.00105210693312 | 387.6469828143171 | 1.73182263554e-13 | 387.6469828143171 | 420.316333388 |
| 386.7973182052604 | 0.000820142049084 | 386.7973182052604 | 1.1733993194e-13 | 386.7973182052604 | 402.560493482 |
| 385.95137012736393 | 0.000638051665918 | 385.95137012736393 | 7.93461024788e-14 | 385.95137012736393 | 384.803091147 |
| 385.1091142490372 | 0.000495404760035 | 385.1091142490372 | 5.35479329698e-14 | 385.1091142490372 | 367.110314638 |
| 384.2705264506212 | 0.000383885804431 | 384.2705264506212 | 3.60659326593e-14 | 384.2705264506212 | 349.545413228 |
| 383.4355828220859 | 0.000296880361124 | 383.4355828220859 | 2.4243145435e-14 | 383.4355828220859 | 332.16828851 |
| 382.6042596607575 | 0.000229138684637 | 382.6042596607575 | 1.62636533155e-14 | 382.6042596607575 | 315.035143371 |
| 381.77653346907607 | 0.000176503321156 | 381.77653346907607 | 1.08889145601e-14 | 381.77653346907607 | 298.198189365 |
| 380.95238095238096 | 0.000135689067677 | 380.95238095238096 | 7.27592832072e-15 | 380.95238095238096 | 281.705412427 |
| 380.1317790167258 | 0.000104105658944 | 380.1317790167258 | 4.85209809581e-15 | 380.1317790167258 | 265.600396112 |
| 379.31470476672143 | 7.97152312532e-05 | 379.31470476672143 | 3.2292976078e-15 | 379.31470476672143 | 249.922200883 |
| 378.5011355034065 | 6.09180186172e-05 | 378.5011355034065 | 2.14498325467e-15 | 378.5011355034065 | 234.705297281 |
| 377.69104872214524 | 4.64609094625e-05 | 377.69104872214524 | 1.42192626831e-15 | 377.69104872214524 | 219.979550302 |
| 376.88442211055275 | 3.53644668852e-05 | 376.88442211055275 | 9.40735609682e-16 | 376.88442211055275 | 205.770251758 |
| 376.081233546446 | 2.68648233175e-05 | 376.081233546446 | 6.21148476487e-16 | 376.081233546446 | 192.098197015 |
| 375.28146109582184 | 2.03675278864e-05 | 375.28146109582184 | 4.09317769178e-16 | 375.28146109582184 | 178.979802144 |
| 374.48508301086 | 1.54109745054e-05 | 374.48508301086 | 2.69192592742e-16 | 374.48508301086 | 166.42725726 |
| 373.69207772795215 | 1.16374902407e-05 | 373.69207772795215 | 1.76686327699e-16 | 373.69207772795215 | 154.44871165 |
| 372.9024238657551 | 8.77053320728e-06 | 372.9024238657551 | 1.15739103629e-16 | 372.9024238657551 | 143.048486187 |
| 372.11610022326965 | 6.59675110981e-06 | 372.11610022326965 | 7.56649266418e-17 | 372.11610022326965 | 132.227308491 |
| 371.33308577794276 | 4.95189730183e-06 | 371.33308577794276 | 4.93681037532e-17 | 371.33308577794276 | 121.982566331 |
| 370.55335968379444 | 3.70980073894e-06 | 370.55335968379444 | 3.21466414437e-17 | 370.55335968379444 | 112.308574902 |
| 369.7769012695673 | 2.77374770891e-06 | 369.7769012695673 | 2.08911378631e-17 | 369.7769012695673 | 103.196853708 |
| 369.0036900369003 | 2.06976359541e-06 | 369.0036900369003 | 1.35495824492e-17 | 369.0036900369003 | 94.6364090656 |
| 368.23370565852457 | 1.54138788548e-06 | 368.23370565852457 | 8.7705545986e-18 | 368.23370565852457 | 86.6140184377 |
| 367.4669279764821 | 1.14561988792e-06 | 367.4669279764821 | 5.66585594052e-18 | 367.4669279764821 | 79.1145131273 |
| 366.7033370003667 | 8.49780085113e-07 | 366.7033370003667 | 3.65292979791e-18 | 366.7033370003667 | 72.1210561797 |
| 365.9429129055867 | 6.29085900316e-07 | 365.9429129055867 | 2.35046857949e-18 | 365.9429129055867 | 65.6154126653 |
| 365.1856360316494 | 4.64783549158e-07 | 365.1856360316494 | 1.509401925e-18 | 365.1856360316494 | 59.578209877 |
| 364.4314868804664 | 3.42711702932e-07 | 364.4314868804664 | 9.67370131691e-19 | 364.4314868804664 | 53.9891853311 |
| 363.68044611468054 | 2.52199667206e-07 | 363.68044611468054 | 6.18753649123e-19 | 363.68044611468054 | 48.8274208151 |
| 362.93249455601256 | 1.85224088913e-07 | 362.93249455601256 | 3.94984630199e-19 | 362.93249455601256 | 44.0715610796 |
| 362.1876131836291 | 1.3576499443e-07 | 362.1876131836291 | 2.51640154727e-19 | 362.1876131836291 | 39.7000161047 |
| 361.4457831325301 | 9.93151545763e-08 | 361.4457831325301 | 1.59998906282e-19 | 361.4457831325301 | 35.6911461998 |
| 360.7069856919562 | 7.25071132331e-08 | 360.7069856919562 | 1.01529303583e-19 | 360.7069856919562 | 32.0234294923 |
| 359.97120230381563 | 5.28303002301e-08 | 359.97120230381563 | 6.42988378035e-20 | 359.97120230381563 | 28.6756116422 |
| 359.2384145611304 | 3.8416952672e-08 | 359.2384145611304 | 4.06398560796e-20 | 359.2384145611304 | 25.626837872 |
| 358.50860420650093 | 2.78804714745e-08 | 358.50860420650093 | 2.56353069124e-20 | 358.50860420650093 | 22.8567676272 |
| 357.7817531305903 | 2.0193643253e-08 | 357.7817531305903 | 1.61384641877e-20 | 357.7817531305903 | 20.3456723804 |
| 357.057843370626 | 1.45971008387e-08 | 357.057843370626 | 1.01396556343e-20 | 357.057843370626 | 18.0745172604 |
| 356.33685710892024 | 1.05306674238e-08 | 356.33685710892024 | 6.3580147239e-21 | 356.33685710892024 | 16.0250273289 |
| 355.6187766714082 | 7.58197843154e-09 | 355.6187766714082 | 3.97884639763e-21 | 355.6187766714082 | 14.1797394407 |
| 354.9035845262037 | 5.44811854129e-09 | 354.9035845262037 | 2.48502143e-21 | 354.9035845262037 | 12.5220407066 |
| 354.1912632821723 | 3.90704044337e-09 | 354.1912632821723 | 1.54896078551e-21 | 354.1912632821723 | 11.0361946444 |
| 353.48179568752204 | 2.7963183835e-09 | 353.48179568752204 | 9.63580555355e-22 | 353.48179568752204 | 9.70735613642 |
| 352.77516462841015 | 1.99738911966e-09 | 352.77516462841015 | 5.98236561486e-22 | 352.77516462841015 | 8.52157633194 |
| 352.07135312756714 | 1.42388885622e-09 | 352.07135312756714 | 3.70676620593e-22 | 352.07135312756714 | 7.46579862938 |
| 351.3703443429374 | 1.01304059777e-09 | 351.3703443429374 | 2.29221187127e-22 | 351.3703443429374 | 6.52784685435 |
| 350.6721215663355 | 7.19308107469e-10 | 350.6721215663355 | 1.41465858232e-22 | 350.6721215663355 | 5.69640671636 |
| 349.9766682221185 | 5.09730247318e-10 | 349.9766682221185 | 8.71336362909e-23 | 349.9766682221185 | 4.96100158175 |
| 349.2839678658749 | 3.60498281411e-10 | 349.2839678658749 | 5.35620705049e-23 | 349.2839678658749 | 4.31196354479 |
| 348.59400418312805 | 2.54450514906e-10 | 348.59400418312805 | 3.28598966617e-23 | 348.59400418312805 | 3.74040071617 |
| 347.90676098805517 | 1.79242424705e-10 | 347.90676098805517 | 2.01192759733e-23 | 347.90676098805517 | 3.23816157968 |
| 347.2222222222222 | 1.26013079781e-10 | 347.2222222222222 | 1.2294073923e-23 | 347.2222222222222 | 2.79779719517 |
| 346.54037195333257 | 8.84153723095e-11 | 346.54037195333257 | 7.49750234267e-24 | 346.54037195333257 | 2.4125219518 |
| 345.8611943739912 | 6.19123475286e-11 | 345.8611943739912 | 4.56325478185e-24 | 345.8611943739912 | 2.07617350035 |
| 345.1846738004832 | 4.32677288189e-11 | 345.1846738004832 | 2.77185266651e-24 | 345.1846738004832 | 1.78317241932 |
| 344.5107946715664 | 3.01778476668e-11 | 344.5107946715664 | 1.68036212545e-24 | 344.5107946715664 | 1.52848209706 |
| 343.8395415472779 | 2.10063094571e-11 | 343.8395415472779 | 1.01665346004e-24 | 343.8395415472779 | 1.30756924283 |
| 343.17089910775564 | 1.45931346426e-11 | 343.17089910775564 | 6.13875525268e-25 | 343.17089910775564 | 1.11636537357 |
| 342.50485215207215 | 1.0117768827e-11 | 342.50485215207215 | 3.69934649692e-25 | 342.50485215207215 | 0.951229561633 |
| 341.84138559708293 | 7.00097048539e-12 | 341.84138559708293 | 2.2248821927e-25 | 341.84138559708293 | 0.808912671556 |
| 341.1804844762879 | 4.83469489359e-12 | 341.1804844762879 | 1.3354460425e-25 | 341.1804844762879 | 0.686523261657 |
| 340.522133938706 | 3.33209386642e-12 | 340.522133938706 | 7.99987105791e-26 | 340.522133938706 | 0.581495279177 |
| 339.86631924776253 | 2.29193718937e-12 | 339.86631924776253 | 4.78274190036e-26 | 339.86631924776253 | 0.49155763553 |
| 339.2130257801899 | 1.57335065253e-12 | 339.2130257801899 | 2.85369936602e-26 | 339.2130257801899 | 0.414705711173 |
| 338.56223902494077 | 1.07791785412e-12 | 338.56223902494077 | 1.69932641596e-26 | 338.56223902494077 | 0.349174807419 |
| 337.91394458211306 | 7.37026551191e-13 | 337.91394458211306 | 1.00991009679e-26 | 337.91394458211306 | 0.293415534977 |
| 337.2681281618887 | 5.02942027399e-13 | 337.2681281618887 | 5.9899877677e-27 | 337.2681281618887 | 0.246071105883 |
| 336.6247755834829 | 3.42523244199e-13 | 336.6247755834829 | 3.54573661768e-27 | 336.6247755834829 | 0.205956476453 |
| 335.9838727741068 | 2.32808854973e-13 | 335.9838727741068 | 2.09471202054e-27 | 335.9838727741068 | 0.172039273619 |
| 335.3454057679409 | 1.57923320098e-13 | 335.3454057679409 | 1.2350356468e-27 | 335.3454057679409 | 0.143422425163 |
| 334.709360705121 | 1.06912961847e-13 | 334.709360705121 | 7.26728138281e-28 | 334.709360705121 | 0.119328405586 |
| 334.07572383073494 | 7.22356824751e-14 | 334.07572383073494 | 4.26777754935e-28 | 334.07572383073494 | 0.0990850032652 |
| 333.44448149383123 | 4.87091468504e-14 | 333.44448149383123 | 2.50131788312e-28 | 333.44448149383123 | 0.0821125108591 |
| 332.81562014643885 | 3.27798218084e-14 | 332.81562014643885 | 1.46309766358e-28 | 332.81562014643885 | 0.0679122392434 |
| 332.1891263425977 | 2.2016079076e-14 | 332.1891263425977 | 8.54112465842e-29 | 332.1891263425977 | 0.0560562553164 |
| 331.5649867374005 | 1.47574284594e-14 | 331.5649867374005 | 4.97615751564e-29 | 331.5649867374005 | 0.0461782455004 |
| 330.9431880860452 | 9.87230850362e-15 | 330.9431880860452 | 2.89341342738e-29 | 330.9431880860452 | 0.0379654094151 |
| 330.323717242898 | 6.59119364999e-15 | 330.323717242898 | 1.67905213302e-29 | 330.323717242898 | 0.0311512917688 |
| 329.70656116056705 | 4.39184238292e-15 | 329.70656116056705 | 9.72422913581e-30 | 329.70656116056705 | 0.0255094647911 |
| 329.0917068889864 | 2.92056408424e-15 | 329.0917068889864 | 5.62061064201e-30 | 329.0917068889864 | 0.020847978314 |
| 328.47914157451 | 1.93831365405e-15 | 328.47914157451 | 3.24226966884e-30 | 328.47914157451 | 0.0170044997359 |
| 327.86885245901635 | 1.28386302054e-15 | 327.86885245901635 | 1.86660351898e-30 | 327.86885245901635 | 0.0138420714336 |
| 327.26082687902255 | 8.48693047485e-16 | 327.26082687902255 | 1.07248775262e-30 | 327.26082687902255 | 0.0112454185881 |
| 326.6550522648083 | 5.59912184272e-16 | 326.6550522648083 | 6.1499263812e-31 | 326.6550522648083 | 0.00911774577226 |
| 326.05151613955 | 3.68660418051e-16 | 326.05151613955 | 3.51953109834e-31 | 326.05151613955 | 0.00737796591987 |
| 325.4502061184639 | 2.42253702293e-16 | 325.4502061184639 | 2.01018955109e-31 | 325.4502061184639 | 0.00595831039872 |
| 324.8511099079588 | 1.58873569528e-16 | 324.8511099079588 | 1.14584673004e-31 | 324.8511099079588 | 0.00480227378796 |
| 324.25421530479895 | 1.0398488165e-16 | 324.25421530479895 | 6.51858544268e-32 | 324.25421530479895 | 0.00386285158169 |
| 323.65951019527455 | 6.79244403061e-17 | 323.65951019527455 | 3.70098657146e-32 | 323.65951019527455 | 0.00310103337703 |
| 323.0669825543829 | 4.42811869007e-17 | 323.0669825543829 | 2.09709917945e-32 | 323.0669825543829 | 0.00248451814237 |
| 322.4766204450177 | 2.88104309955e-17 | 322.4766204450177 | 1.18592642283e-32 | 322.4766204450177 | 0.00198662189301 |
| 321.88841201716735 | 1.87075784391e-17 | 321.88841201716735 | 6.69320049505e-33 | 321.88841201716735 | 0.00158535152618 |
| 321.3023455071222 | 1.21233519072e-17 | 321.3023455071222 | 3.7700511074e-33 | 321.3023455071222 | 0.00126262169102 |
| 320.71840923669015 | 7.84088646935e-18 | 320.71840923669015 | 2.11932692018e-33 | 320.71840923669015 | 0.00100359440137 |
| 320.1365916124213 | 5.06110020953e-18 | 320.1365916124213 | 1.1890113099e-33 | 320.1365916124213 | 0.000796123652625 |
| 319.5568811248402 | 3.26033348131e-18 | 319.5568811248402 | 6.65750238238e-34 | 319.5568811248402 | 0.000630289593789 |
| 318.97926634768737 | 2.0961213811e-18 | 318.97926634768737 | 3.72026596642e-34 | 318.97926634768737 | 0.000498008849249 |
| 318.40373593716834 | 1.34495622193e-18 | 318.40373593716834 | 2.07478911929e-34 | 318.40373593716834 | 0.000392709399251 |
| 317.8302786312109 | 8.61265766033e-19 | 317.8302786312109 | 1.1548118936e-34 | 317.8302786312109 | 0.00030906003184 |
| 317.2588832487309 | 5.50431827157e-19 | 317.2588832487309 | 6.41484028924e-35 | 317.2588832487309 | 0.000242745790272 |
| 316.6895386889053 | 3.51080902845e-19 | 316.6895386889053 | 3.55629485932e-35 | 316.6895386889053 | 0.000190282076494 |
| 316.1222339304531 | 2.23484903191e-19 | 316.1222339304531 | 1.96764610182e-35 | 316.1222339304531 | 0.000148861150144 |
| 315.55695803092453 | 1.41979777533e-19 | 315.55695803092453 | 1.08650950678e-35 | 315.55695803092453 | 0.000116225700181 |
| 314.99370012599746 | 9.00206422745e-20 | 314.99370012599746 | 5.98766350014e-36 | 314.99370012599746 | 9.05649778473e-05 |
| 314.432449428781 | 5.69632865684e-20 | 314.432449428781 | 3.29320339113e-36 | 314.432449428781 | 7.04296795267e-05 |
| 313.8731952291274 | 3.5973714631e-20 | 313.8731952291274 | 1.80766119855e-36 | 313.8731952291274 | 5.46623693394e-05 |
| 313.31592689295036 | 2.26732014723e-20 | 313.31592689295036 | 9.90268196855e-37 | 313.31592689295036 | 4.23407459683e-05 |
| 312.76063386155124 | 1.42619110757e-20 | 312.76063386155124 | 5.41409585767e-37 | 312.76063386155124 | 3.2731497201e-05 |
| 312.2073056509522 | 8.95323361131e-21 | 312.2073056509522 | 2.95417598573e-37 | 312.2073056509522 | 2.52528587866e-05 |
| 311.65593185123623 | 5.6094389216e-21 | 311.65593185123623 | 1.60873347811e-37 | 311.65593185123623 | 1.94443102559e-05 |
| 311.1065021258944 | 3.50748841773e-21 | 311.1065021258944 | 8.7431745617e-38 | 311.1065021258944 | 1.49421071714e-05 |
| 310.5590062111801 | 2.18882168369e-21 | 310.5590062111801 | 4.74232717192e-38 | 310.5590062111801 | 1.14595737766e-05 |
| 310.01343391546965 | 1.36320708309e-21 | 310.01343391546965 | 2.56714966706e-38 | 310.01343391546965 | 8.77126830364e-06 |
| 309.4697751186301 | 8.47326131386e-22 | 309.4697751186301 | 1.38690967955e-38 | 309.4697751186301 | 6.7002906102e-06 |
| 308.9280197713932 | 5.25625808246e-22 | 308.9280197713932 | 7.47794872026e-39 | 308.9280197713932 | 5.10813300087e-06 |
| 308.3881578947368 | 3.25416879513e-22 | 308.3881578947368 | 4.02396411901e-39 | 308.3881578947368 | 3.88658407017e-06 |
| 307.8501795792714 | 2.01066994357e-22 | 307.8501795792714 | 2.16104111566e-39 | 307.8501795792714 | 2.95128566986e-06 |
| 307.31407498463426 | 1.23987758871e-22 | 307.31407498463426 | 1.15826857433e-39 | 307.31407498463426 | 2.23661763504e-06 |
| 306.77983433888943 | 7.63052023582e-23 | 306.77983433888943 | 6.19573494659e-40 | 306.77983433888943 | 1.69164626263e-06 |
| 306.2474479379338 | 4.68669618251e-23 | 306.2474479379338 | 3.30760546397e-40 | 306.2474479379338 | 1.27692289924e-06 |
| 305.7169061449098 | 2.87287525037e-23 | 305.7169061449098 | 1.76226771243e-40 | 305.7169061449098 | 9.61960213326e-07 |
| 305.1881993896236 | 1.75753526903e-23 | 305.1881993896236 | 9.37059960295e-41 | 305.1881993896236 | 7.23247360985e-07 |
| 304.6613181679699 | 1.07307145927e-23 | 304.6613181679699 | 4.97279082534e-41 | 304.6613181679699 | 5.4269262322e-07 |
| 304.1362530413625 | 6.53868706738e-24 | 304.1362530413625 | 2.63372432602e-41 | 304.1362530413625 | 4.06404297663e-07 |
| 303.61299463617036 | 3.97639735502e-24 | 303.61299463617036 | 1.39212345883e-41 | 303.61299463617036 | 3.03738594146e-07 |
| 303.09153364316023 | 2.41338335626e-24 | 303.09153364316023 | 7.34382808907e-42 | 303.09153364316023 | 2.26557778741e-07 |
| 302.571860816944 | 1.46184108756e-24 | 302.571860816944 | 3.86638026415e-42 | 302.571860816944 | 1.68653473675e-07 |
| 302.0539669754329 | 8.83713173715e-25 | 302.0539669754329 | 2.03153328308e-42 | 302.0539669754329 | 1.25299378279e-07 |
| 301.5378429992964 | 5.33162760453e-25 | 301.5378429992964 | 1.06532135225e-42 | 301.5378429992964 | 9.2905164056e-08 |
| 301.02347983142687 | 3.21029986368e-25 | 301.02347983142687 | 5.57538207799e-43 | 301.02347983142687 | 6.87492723673e-08 |
| 300.5108684764098 | 1.92916203477e-25 | 300.5108684764098 | 2.91209776454e-43 | 300.5108684764098 | 5.07730988268e-08 |

| **GON2-Al3** | | **GON2-Al4** | | **GON2-Al5** | |
| --- | --- | --- | --- | --- | --- |
| Wavelength (nm) | Abs | Wavelength (nm) | Abs | Wavelength (nm) | Abs |
| 2000.0 | 1.44191932755e-05 | 2000.0 | 8.88192612483e-15 | 2000.0 | 0.0769892086799 |
| 1977.5873434410018 | 1.88347823971e-05 | 1977.5873434410018 | 1.32303139008e-14 | 1977.5873434410018 | 0.0937847458986 |
| 1955.671447196871 | 2.45537373818e-05 | 1955.671447196871 | 1.96684643533e-14 | 1955.671447196871 | 0.114049889861 |
| 1934.2359767891683 | 3.1945667308e-05 | 1934.2359767891683 | 2.91815308243e-14 | 1934.2359767891683 | 0.138458179618 |
| 1913.265306122449 | 4.14804715062e-05 | 1913.265306122449 | 4.32098741382e-14 | 1913.265306122449 | 0.167804711597 |
| 1892.7444794952683 | 5.37542453981e-05 | 1892.7444794952683 | 6.38550493551e-14 | 1892.7444794952683 | 0.203026209058 |
| 1872.6591760299625 | 6.95215144294e-05 | 1872.6591760299625 | 9.41769890666e-14 | 1872.6591760299625 | 0.245224042737 |
| 1852.9956763434218 | 8.97352447519e-05 | 1852.9956763434218 | 1.38621843796e-13 | 1852.9956763434218 | 0.295690569516 |
| 1833.7408312958437 | 0.000115596386088 | 1833.7408312958437 | 2.03636607658e-13 | 1833.7408312958437 | 0.355939189615 |
| 1814.8820326678765 | 0.000148615067897 | 1814.8820326678765 | 2.98550188082e-13 | 1814.8820326678765 | 0.427738557306 |
| 1796.4071856287424 | 0.000190686004427 | 1796.4071856287424 | 4.36833722646e-13 | 1796.4071856287424 | 0.51315141526 |
| 1778.3046828689983 | 0.000244181178711 | 1778.3046828689983 | 6.37899525478e-13 | 1778.3046828689983 | 0.61457855766 |
| 1760.5633802816901 | 0.000312063482774 | 1760.5633802816901 | 9.29663357236e-13 | 1760.5633802816901 | 0.734808461628 |
| 1743.1725740848342 | 0.000398025705464 | 1743.1725740848342 | 1.35218608053e-12 | 1743.1725740848342 | 0.87707315955 |
| 1726.1219792865363 | 0.000506660096345 | 1726.1219792865363 | 1.96283829005e-12 | 1726.1219792865363 | 1.04511095551 |
| 1709.4017094017095 | 0.000643664711649 | 1709.4017094017095 | 2.84360909615e-12 | 1709.4017094017095 | 1.24323661644 |
| 1693.002257336343 | 0.000816093885803 | 1693.002257336343 | 4.11142697693e-12 | 1693.002257336343 | 1.47641969137 |
| 1676.9144773616547 | 0.00103266149075 | 1676.9144773616547 | 5.93270263208e-12 | 1676.9144773616547 | 1.75037162893 |
| 1661.1295681063123 | 0.00130410716822 | 1661.1295681063123 | 8.54377677356e-12 | 1661.1295681063123 | 2.07164237306 |
| 1645.6390565002741 | 0.00164363747216 | 1645.6390565002741 | 1.22796084262e-11 | 1645.6390565002741 | 2.44772711672 |
| 1630.4347826086955 | 0.00206745586542 | 1630.4347826086955 | 1.76139377233e-11 | 1630.4347826086955 | 2.88718388341 |
| 1615.5088852988692 | 0.0025953978054 | 1615.5088852988692 | 2.52153910526e-11 | 1615.5088852988692 | 3.39976258254 |
| 1600.8537886872998 | 0.00325168975446 | 1600.8537886872998 | 3.60256870534e-11 | 1600.8537886872998 | 3.99654614623 |
| 1586.4621893178212 | 0.00406585389395 | 1586.4621893178212 | 5.13684130863e-11 | 1586.4621893178212 | 4.69010429985 |
| 1572.3270440251572 | 0.00507378363266 | 1572.3270440251572 | 7.30999937172e-11 | 1572.3270440251572 | 5.49466044291 |
| 1558.4415584415583 | 0.00631901871231 | 1558.4415584415583 | 1.03818761311e-10 | 1558.4415584415583 | 6.42627202048 |
| 1544.799176107106 | 0.00785425284892 | 1544.799176107106 | 1.47153860192e-10 | 1544.799176107106 | 7.50302464386 |
| 1531.3935681470139 | 0.00974311143618 | 1531.3935681470139 | 2.0816359923e-10 | 1531.3935681470139 | 8.74524007162 |
| 1518.2186234817814 | 0.0120622418945 | 1518.2186234817814 | 2.93883518428e-10 | 1518.2186234817814 | 10.1756979863 |
| 1505.2684395383842 | 0.0149037647923 | 1505.2684395383842 | 4.14078790316e-10 | 1505.2684395383842 | 11.8198712946 |
| 1492.5373134328358 | 0.0183781399051 | 1492.5373134328358 | 5.82274879489e-10 | 1492.5373134328358 | 13.7061744407 |
| 1480.0197335964478 | 0.0226175079071 | 1480.0197335964478 | 8.17166276385e-10 | 1480.0197335964478 | 15.8662239489 |
| 1467.7103718199608 | 0.0277795754053 | 1467.7103718199608 | 1.14453777174e-09 | 1467.7103718199608 | 18.3351101046 |
| 1455.604075691412 | 0.034052118497 | 1455.604075691412 | 1.59987899677e-09 | 1455.604075691412 | 21.1516783407 |
| 1443.6958614051973 | 0.0416581879161 | 1443.6958614051973 | 2.2319345617e-09 | 1443.6958614051973 | 24.3588185204 |
| 1431.9809069212408 | 0.0508621070825 | 1431.9809069212408 | 3.10751397684e-09 | 1431.9809069212408 | 28.0037598947 |
| 1420.4545454545455 | 0.061976362887 | 1420.4545454545455 | 4.3179940249e-09 | 1420.4545454545455 | 32.1383690735 |
| 1409.1122592766555 | 0.0753694977411 | 1409.1122592766555 | 5.98808968759e-09 | 1409.1122592766555 | 36.8194478783 |
| 1397.9496738117427 | 0.0914751201533 | 1397.9496738117427 | 8.28765891181e-09 | 1397.9496738117427 | 42.1090274462 |
| 1386.9625520110958 | 0.110802159707 | 1386.9625520110958 | 1.14475554108e-08 | 1386.9625520110958 | 48.0746544447 |
| 1376.1467889908256 | 0.133946500608 | 1376.1467889908256 | 1.57808703888e-08 | 1376.1467889908256 | 54.7896647246 |
| 1365.4984069185252 | 0.161604135728 | 1365.4984069185252 | 2.17113318037e-08 | 1365.4984069185252 | 62.3334392068 |
| 1355.0135501355014 | 0.194585989988 | 1355.0135501355014 | 2.98111883992e-08 | 1355.0135501355014 | 70.7916362683 |
| 1344.688480502017 | 0.233834567756 | 1344.688480502017 | 4.0851633495e-08 | 1344.688480502017 | 80.2563943757 |
| 1334.5195729537365 | 0.280442583278 | 1334.5195729537365 | 5.58697692637e-08 | 1334.5195729537365 | 90.8264982251 |
| 1324.5033112582782 | 0.335673735637 | 1324.5033112582782 | 7.62573381609e-08 | 1324.5033112582782 | 102.607501192 |
| 1314.6362839614374 | 0.400985789988 | 1314.6362839614374 | 1.03878033333e-07 | 1314.6362839614374 | 115.711796497 |
| 1304.9151805132665 | 0.478056124273 | 1304.9151805132665 | 1.41222244283e-07 | 1304.9151805132665 | 130.258629158 |
| 1295.3367875647668 | 0.568809894867 | 1295.3367875647668 | 1.91610724187e-07 | 1295.3367875647668 | 146.374040548 |
| 1285.8979854264894 | 0.675450965148 | 1285.8979854264894 | 2.59462040237e-07 | 1285.8979854264894 | 164.190737229 |
| 1276.5957446808509 | 0.800495727138 | 1276.5957446808509 | 3.50642987946e-07 | 1276.5957446808509 | 183.847875695 |
| 1267.427122940431 | 0.946809927799 | 1267.427122940431 | 4.7292666476e-07 | 1267.427122940431 | 205.490754748 |
| 1258.3892617449665 | 1.1176485875 | 1258.3892617449665 | 6.36589919715e-07 | 1258.3892617449665 | 229.270407481 |
| 1249.4793835901708 | 1.31669906826 | 1249.4793835901708 | 8.55190812853e-07 | 1249.4793835901708 | 255.343085249 |
| 1240.6947890818858 | 1.54812731295 | 1240.6947890818858 | 1.14657801266e-06 | 1240.6947890818858 | 283.869626575 |
| 1232.0328542094455 | 1.81662723347 | 1232.0328542094455 | 1.53419832818e-06 | 1232.0328542094455 | 315.014704732 |
| 1223.4910277324632 | 2.12747317516 | 1223.4910277324632 | 2.04878654994e-06 | 1223.4910277324632 | 348.945948688 |
| 1215.0668286755772 | 2.48657532672 | 1215.0668286755772 | 2.7305443634e-06 | 1215.0668286755772 | 385.832933285 |
| 1206.7578439259853 | 2.90053787887 | 1206.7578439259853 | 3.63194340877e-06 | 1206.7578439259853 | 425.846035877 |
| 1198.5617259288852 | 3.37671966113 | 1198.5617259288852 | 4.82132294165e-06 | 1198.5617259288852 | 469.155158281 |
| 1190.4761904761904 | 3.92329690449 | 1190.4761904761904 | 6.3874966484e-06 | 1190.4761904761904 | 515.92831461 |
| 1182.4990145841543 | 4.54932768885 | 1182.4990145841543 | 8.44563798274e-06 | 1182.4990145841543 | 566.330087592 |
| 1174.6280344557556 | 5.26481753763 | 1174.6280344557556 | 1.11447813321e-05 | 1174.6280344557556 | 620.519958094 |
| 1166.8611435239206 | 6.08078552018 | 1166.8611435239206 | 1.46773603009e-05 | 1166.8611435239206 | 678.65051487 |
| 1159.19629057187 | 7.00933011429 | 1159.19629057187 | 1.92913079092e-05 | 1159.19629057187 | 740.86555401 |
| 1151.6314779270633 | 8.06369397008 | 1151.6314779270633 | 2.53053707225e-05 | 1151.6314779270633 | 807.298080072 |
| 1144.1647597254005 | 9.25832660174 | 1144.1647597254005 | 3.31284448435e-05 | 1144.1647597254005 | 878.068223489 |
| 1136.794240242516 | 10.6089439192 | 1136.794240242516 | 4.3283932213e-05 | 1136.794240242516 | 953.281091445 |
| 1129.5180722891566 | 12.1325833975 | 1129.5180722891566 | 5.64403477994e-05 | 1129.5180722891566 | 1033.02457202 |
| 1122.334455667789 | 13.8476535739 | 1122.334455667789 | 7.34496902416e-05 | 1122.334455667789 | 1117.3671139 |
| 1115.2416356877322 | 15.7739764559 | 1115.2416356877322 | 9.53954300388e-05 | 1115.2416356877322 | 1206.35550634 |
| 1108.2379017362393 | 17.9328213346 | 1108.2379017362393 | 0.000123652381769 | 1108.2379017362393 | 1300.01268627 |
| 1101.3215859030836 | 20.3469284126 | 1101.3215859030836 | 0.00015996122324 | 1101.3215859030836 | 1398.33560141 |
| 1094.4910616563297 | 23.0405205932 | 1094.4910616563297 | 0.000206521020197 | 1094.4910616563297 | 1501.29315992 |
| 1087.7447425670775 | 26.0393017297 | 1087.7447425670775 | 0.000266103827836 | 1087.7447425670775 | 1608.82429831 |
| 1081.081081081081 | 29.3704396148 | 1081.081081081081 | 0.000342196290149 | 1081.081081081081 | 1720.83620053 |
| 1074.4985673352435 | 33.0625319894 | 1074.4985673352435 | 0.000439174168056 | 1074.4985673352435 | 1837.20270119 |
| 1067.995728017088 | 37.1455538869 | 1067.995728017088 | 0.000562516923794 | 1067.995728017088 | 1957.76290614 |
| 1061.5711252653928 | 41.6507846959 | 1061.5711252653928 | 0.000719070901558 | 1061.5711252653928 | 2082.32006289 |
| 1055.2233556102708 | 46.6107134257 | 1055.2233556102708 | 0.000917371306487 | 1055.2233556102708 | 2210.64071218 |
| 1048.951048951049 | 52.0589208017 | 1048.951048951049 | 0.0011680351329 | 1048.951048951049 | 2342.45415033 |
| 1042.752867570386 | 58.0299369994 | 1042.752867570386 | 0.00148423946952 | 1042.752867570386 | 2477.45222937 |
| 1036.6275051831374 | 64.5590740505 | 1036.6275051831374 | 0.00188230226006 | 1036.6275051831374 | 2615.28951949 |
| 1030.5736860185502 | 71.6822322195 | 1030.5736860185502 | 0.00238238567123 | 1030.5736860185502 | 2755.58385421 |
| 1024.5901639344263 | 79.4356799601 | 1024.5901639344263 | 0.00300934577218 | 1024.5901639344263 | 2897.91727499 |
| 1018.6757215619693 | 87.8558074091 | 1018.6757215619693 | 0.00379375631613 | 1018.6757215619693 | 3041.83738708 |
| 1012.829169480081 | 96.9788537646 | 1012.829169480081 | 0.00477313909995 | 1012.829169480081 | 3186.85913366 |
| 1007.0493454179255 | 106.840609322 | 1007.0493454179255 | 0.00599343872441 | 1007.0493454179255 | 3332.46698924 |
| 1001.3351134846461 | 117.476093393 | 1001.3351134846461 | 0.00751078565548 | 1001.3351134846461 | 3478.11756818 |
| 995.6853634251576 | 128.919209821 | 995.6853634251576 | 0.00939359836308 | 995.6853634251576 | 3623.24263745 |
| 990.0990099009902 | 141.202382296 | 990.0990099009902 | 0.0117250830587 | 990.0990099009902 | 3767.25251701 |
| 984.5749917952082 | 154.356172199 | 984.5749917952082 | 0.0146061982334 | 984.5749917952082 | 3909.53984467 |
| 979.1122715404699 | 168.408882207 | 979.1122715404699 | 0.0181591608786 | 979.1122715404699 | 4049.48367629 |
| 973.7098344693281 | 183.386149397 | 973.7098344693281 | 0.0225315820105 | 973.7098344693281 | 4186.45388608 |
| 968.3666881859263 | 199.31053209 | 968.3666881859263 | 0.0279013309637 | 968.3666881859263 | 4319.81582621 |
| 963.0818619582664 | 216.201095116 | 963.0818619582664 | 0.0344822409123 | 963.0818619582664 | 4448.93519951 |
| 957.8544061302682 | 234.072998608 | 957.8544061302682 | 0.0425307822283 | 957.8544061302682 | 4573.18309466 |
| 952.6833915528738 | 252.937095798 | 952.6833915528738 | 0.0523538456082 | 952.6833915528738 | 4691.94112879 |
| 947.5679090334806 | 272.799545602 | 947.5679090334806 | 0.0643177933568 | 947.5679090334806 | 4804.60663943 |
| 942.5070688030161 | 293.661445961 | 942.5070688030161 | 0.0788589547549 | 942.5070688030161 | 4910.59786511 |
| 937.4999999999999 | 315.51849411 | 937.4999999999999 | 0.0964957599667 | 937.4999999999999 | 5009.35905224 |
| 932.5458501709667 | 338.360679926 | 932.5458501709667 | 0.117842726315 | 932.5458501709667 | 5100.36542542 |
| 927.643784786642 | 362.172018481 | 927.643784786642 | 0.143626530788 | 927.643784786642 | 5183.12795842 |
| 922.7929867733004 | 386.930327741 | 922.7929867733004 | 0.174704423099 | 922.7929867733004 | 5257.19788478 |
| 917.9926560587514 | 412.60705705 | 917.9926560587514 | 0.212085254156 | 917.9926560587514 | 5322.17088887 |
| 913.2420091324201 | 439.167171655 | 913.2420091324201 | 0.256953415089 | 913.2420091324201 | 5377.690922 |
| 908.5402786190186 | 466.569097959 | 908.5402786190186 | 0.310696001511 | 908.5402786190186 | 5423.4535923 |
| 903.8867128653209 | 494.764733584 | 903.8867128653209 | 0.374933535935 | 903.8867128653209 | 5459.20908217 |
| 899.2805755395683 | 523.699525541 | 899.2805755395683 | 0.451554597609 | 899.2805755395683 | 5484.76455336 |
| 894.7211452430658 | 553.312618936 | 894.7211452430658 | 0.542754722705 | 894.7211452430658 | 5499.98600611 |
| 890.2077151335311 | 583.537077718 | 890.2077151335311 | 0.651079948027 | 890.2077151335311 | 5504.79956648 |
| 885.7395925597874 | 614.300177896 | 885.7395925597874 | 0.779475377216 | 885.7395925597874 | 5499.19218335 |
| 881.316098707403 | 645.523772584 | 881.316098707403 | 0.931339148877 | 881.316098707403 | 5483.21172517 |
| 876.9365682548962 | 677.124727057 | 876.9365682548962 | 1.11058217993 | 876.9365682548962 | 5456.96647435 |
| 872.6003490401396 | 709.015420866 | 872.6003490401396 | 1.32169404376 | 872.6003490401396 | 5420.62402614 |
| 868.3068017366135 | 741.104312835 | 868.3068017366135 | 1.56981532004 | 868.3068017366135 | 5374.40960647 |
| 864.0552995391705 | 773.296563653 | 864.0552995391705 | 1.8608167201 | 864.0552995391705 | 5318.60383196 |
| 859.8452278589854 | 805.494709638 | 859.8452278589854 | 2.20138524721 | 859.8452278589854 | 5253.53994221 |
| 855.6759840273816 | 837.59938021 | 855.6759840273816 | 2.59911759374 | 855.6759840273816 | 5179.60054228 |
| 851.5469770082316 | 869.510050667 | 851.5469770082316 | 3.06262090553 | 851.5469770082316 | 5097.21389921 |
| 847.457627118644 | 901.125821027 | 847.457627118644 | 3.60162095645 | 847.457627118644 | 5006.84984239 |
| 843.4073657576608 | 932.346210996 | 843.4073657576608 | 4.22707767265 | 843.4073657576608 | 4909.0153224 |
| 839.3956351426972 | 963.071960598 | 839.3956351426972 | 4.95130782434 | 839.3956351426972 | 4804.24968682 |
| 835.421888053467 | 993.205825642 | 835.421888053467 | 5.78811456338 | 835.421888053467 | 4693.11973445 |
| 831.4855875831485 | 1022.65335701 | 831.4855875831485 | 6.75292332591 | 831.4855875831485 | 4576.21461126 |
| 827.5862068965516 | 1051.32365283 | 827.5862068965516 | 7.86292344148 | 827.5862068965516 | 4454.14061237 |
| 823.7232289950576 | 1079.13007275 | 823.7232289950576 | 9.13721459319 | 823.7232289950576 | 4327.51595397 |
| 819.8961464881114 | 1105.99090403 | 819.8961464881114 | 10.5969570576 | 819.8961464881114 | 4196.96557815 |
| 816.1044613710554 | 1131.82996988 | 816.1044613710554 | 12.2655244209 | 816.1044613710554 | 4063.11605129 |
| 812.3476848090983 | 1156.57717106 | 812.3476848090983 | 14.1686572184 | 812.3476848090983 | 3926.59061368 |
| 808.6253369272237 | 1180.16895312 | 808.6253369272237 | 16.3346156834 | 808.6253369272237 | 3788.00443412 |
| 804.9369466058491 | 1202.54869262 | 804.9369466058491 | 18.7943295185 | 804.9369466058491 | 3647.96011879 |
| 801.2820512820513 | 1223.66699696 | 801.2820512820513 | 21.5815423215 | 801.2820512820513 | 3507.04351814 |
| 797.6601967561818 | 1243.48191425 | 797.6601967561818 | 24.7329480189 | 797.6601967561818 | 3365.81987017 |
| 794.0709370037056 | 1261.95905069 | 794.0709370037056 | 28.2883163757 | 794.0709370037056 | 3224.83031205 |
| 790.5138339920949 | 1279.07159499 | 790.5138339920949 | 32.2906043827 | 790.5138339920949 | 3084.58878577 |
| 786.9884575026232 | 1294.80025076 | 786.9884575026232 | 36.7860500612 | 786.9884575026232 | 2945.57935681 |
| 783.4943849569078 | 1309.13307938 | 783.4943849569078 | 41.8242449936 | 783.4943849569078 | 2808.25395856 |
| 780.0312012480499 | 1322.06525775 | 780.0312012480499 | 47.4581816772 | 780.0312012480499 | 2673.03056814 |
| 776.598498576236 | 1333.59875637 | 776.598498576236 | 53.7442716343 | 776.598498576236 | 2540.29181379 |
| 773.1958762886597 | 1343.74194506 | 773.1958762886597 | 60.7423300867 | 773.1958762886597 | 2410.38400715 |
| 769.8229407236336 | 1352.5091345 | 769.8229407236336 | 68.5155229345 | 769.8229407236336 | 2283.61658905 |
| 766.4793050587633 | 1359.92006318 | 766.4793050587633 | 77.1302717768 | 766.4793050587633 | 2160.26197148 |
| 763.1645891630628 | 1365.99933992 | 763.1645891630628 | 86.6561127777 | 763.1645891630628 | 2040.55575462 |
| 759.8784194528876 | 1370.77585311 | 759.8784194528876 | 97.1655053317 | 759.8784194528876 | 1924.69729332 |
| 756.6204287515762 | 1374.28215797 | 756.6204287515762 | 108.733586717 | 756.6204287515762 | 1812.85058425 |
| 753.390256152687 | 1376.55385358 | 753.390256152687 | 121.437869263 | 753.390256152687 | 1705.14544246 |
| 750.1875468867216 | 1377.62896119 | 750.1875468867216 | 135.357876972 | 750.1875468867216 | 1601.67893373 |
| 747.011952191235 | 1377.54731513 | 747.011952191235 | 150.574719109 | 747.011952191235 | 1502.51702803 |
| 743.86312918423 | 1376.34997713 | 743.86312918423 | 167.170598869 | 743.86312918423 | 1407.69643857 |
| 740.7407407407408 | 1374.07868399 | 740.7407407407408 | 185.228256025 | 740.7407407407408 | 1317.22661082 |
| 737.6444553725104 | 1370.77533767 | 737.6444553725104 | 204.830343292 | 737.6444553725104 | 1231.09182644 |
| 734.5739471106758 | 1366.48154577 | 734.5739471106758 | 226.058737116 | 734.5739471106758 | 1149.25338792 |
| 731.528895391368 | 1361.23821887 | 731.528895391368 | 248.993784637 | 731.528895391368 | 1071.65185132 |
| 728.5089849441475 | 1355.08523005 | 728.5089849441475 | 273.713489734 | 728.5089849441475 | 998.209276414 |
| 725.5139056831922 | 1348.06114007 | 725.5139056831922 | 300.292642251 | 725.5139056831922 | 928.831465587 |
| 722.543352601156 | 1340.2029904 | 722.543352601156 | 328.801895803 | 722.543352601156 | 863.41016551 |
| 719.5970256656271 | 1331.54616454 | 719.5970256656271 | 359.306800827 | 719.5970256656271 | 801.825208298 |
| 716.6746297181079 | 1322.12431655 | 716.6746297181079 | 391.86680093 | 716.6746297181079 | 743.946571695 |
| 713.7758743754462 | 1311.96936406 | 713.7758743754462 | 426.534201864 | 713.7758743754462 | 689.636340788 |
| 710.9004739336492 | 1301.11154189 | 710.9004739336492 | 463.353123774 | 710.9004739336492 | 638.750556774 |
| 708.0481472740146 | 1289.57951078 | 708.0481472740146 | 502.358448613 | 708.0481472740146 | 591.140941192 |
| 705.2186177715091 | 1277.4005148 | 705.2186177715091 | 543.57477574 | 705.2186177715091 | 546.656486939 |
| 702.4116132053383 | 1264.60058 | 702.4116132053383 | 587.015399796 | 702.4116132053383 | 505.144910113 |
| 699.6268656716418 | 1251.20474596 | 699.6268656716418 | 632.681325803 | 699.6268656716418 | 466.453959309 |
| 696.8641114982578 | 1237.23732154 | 696.8641114982578 | 680.560337183 | 696.8641114982578 | 430.432581369 |
| 694.1230911614992 | 1222.72215567 | 694.1230911614992 | 730.626132868 | 694.1230911614992 | 396.931944751 |
| 691.4035492048858 | 1207.68291394 | 691.4035492048858 | 782.837549997 | 691.4035492048858 | 365.806323628 |
| 688.7052341597796 | 1192.14335189 | 688.7052341597796 | 837.137888675 | 688.7052341597796 | 336.913847465 |
| 686.027898467871 | 1176.12757625 | 686.027898467871 | 893.454355057 | 686.027898467871 | 310.117122291 |
| 683.371298405467 | 1159.66028591 | 683.371298405467 | 951.697638446 | 683.371298405467 | 285.283731011 |
| 680.7351940095303 | 1142.7669852 | 680.7351940095303 | 1011.76163726 | 680.7351940095303 | 262.286621055 |
| 678.1193490054249 | 1125.47416283 | 678.1193490054249 | 1073.52334758 | 678.1193490054249 | 241.004388327 |
| 675.5235307363206 | 1107.80943111 | 675.5235307363206 | 1136.84292643 | 675.5235307363206 | 221.321466871 |
| 672.9475100942127 | 1089.80162104 | 672.9475100942127 | 1201.56394027 | 672.9475100942127 | 203.128233918 |
| 670.3910614525139 | 1071.4808302 | 670.3910614525139 | 1267.51380708 | 670.3910614525139 | 186.32104005 |
| 667.8539626001781 | 1052.87842172 | 667.8539626001781 | 1334.5044379 | 667.8539626001781 | 170.802174072 |
| 665.335994677312 | 1034.02697382 | 665.335994677312 | 1402.33308137 | 665.335994677312 | 156.479771947 |
| 662.8369421122403 | 1014.96018079 | 662.8369421122403 | 1470.78337169 | 662.8369421122403 | 143.267678758 |
| 660.3565925599823 | 995.712707505 | 660.3565925599823 | 1539.62657763 | 660.3565925599823 | 131.085272153 |
| 657.8947368421053 | 976.320000741 | 657.8947368421053 | 1608.62304699 | 657.8947368421053 | 119.857255175 |
| 655.4511688879178 | 956.818061755 | 655.4511688879178 | 1677.52383769 | 655.4511688879178 | 109.513425744 |
| 653.0256856769699 | 937.243185301 | 653.0256856769699 | 1746.07252343 | 653.0256856769699 | 99.9884293738 |
| 650.6180871828237 | 917.631671275 | 650.6180871828237 | 1814.00715868 | 650.6180871828237 | 91.2215010208 |
| 648.2281763180639 | 898.019515724 | 648.2281763180639 | 1881.06238458 | 648.2281763180639 | 83.1562012489 |
| 645.8557588805166 | 878.44208839 | 645.8557588805166 | 1946.97165435 | 645.8557588805166 | 75.7401512109 |
| 643.5006435006435 | 858.933804294 | 643.5006435006435 | 2011.46955414 | 643.5006435006435 | 68.9247702603 |
| 641.1626415900834 | 839.527796905 | 641.1626415900834 | 2074.29419274 | 641.1626415900834 | 62.6650193654 |
| 638.8415672913118 | 820.255600361 | 638.8415672913118 | 2135.18963141 | 638.8415672913118 | 56.9191528952 |
| 636.5372374283895 | 801.146847938 | 636.5372374283895 | 2193.90832324 | 636.5372374283895 | 51.6484807789 |
| 634.2494714587738 | 782.228993529 | 634.2494714587738 | 2250.21353055 | 634.2494714587738 | 46.8171425331 |
| 631.9780914261638 | 763.527062278 | 631.9780914261638 | 2303.88168739 | 631.9780914261638 | 42.3918941922 |
| 629.7229219143577 | 745.063435832 | 629.7229219143577 | 2354.70467459 | 629.7229219143577 | 38.3419087686 |
| 627.4837900020916 | 726.857676805 | 627.4837900020916 | 2402.49197439 | 627.4837900020916 | 34.6385905235 |
| 625.2605252188412 | 708.926396134 | 625.2605252188412 | 2447.07267301 | 625.2605252188412 | 31.2554030282 |
| 623.0529595015576 | 691.283166027 | 623.0529595015576 | 2488.29728052 | 623.0529595015576 | 28.1677107486 |
| 620.8609271523178 | 673.938480149 | 620.8609271523178 | 2526.03933952 | 620.8609271523178 | 25.3526336892 |
| 618.6842647968654 | 656.899761666 | 618.6842647968654 | 2560.19679631 | 618.6842647968654 | 22.7889144753 |
| 616.5228113440197 | 640.171418728 | 616.5228113440197 | 2590.69311153 | 616.5228113440197 | 20.4567971405 |
| 614.3764079459348 | 623.754945961 | 614.3764079459348 | 2617.47809029 | 614.3764079459348 | 18.3379168066 |
| 612.2448979591836 | 607.649069593 | 612.2448979591836 | 2640.52841608 | 612.2448979591836 | 16.4151993995 |
| 610.1281269066504 | 591.849932991 | 610.1281269066504 | 2659.84787642 | 610.1281269066504 | 14.6727705232 |
| 608.0259424402108 | 576.351318578 | 608.0259424402108 | 2675.46727328 | 608.0259424402108 | 13.095872621 |
| 605.9381943041809 | 561.14490145 | 605.9381943041809 | 2687.44401549 | 605.9381943041809 | 11.6707895753 |
| 603.864734299517 | 546.220529478 | 603.864734299517 | 2695.86139555 | 603.864734299517 | 10.3847779352 |
| 601.8054162487462 | 531.566524229 | 601.8054162487462 | 2700.82755796 | 601.8054162487462 | 9.22600401221 |
| 599.7600959616153 | 517.169996793 | 599.7600959616153 | 2702.4741711 | 599.7600959616153 | 8.1834861414 |
| 597.7286312014345 | 503.017172428 | 597.7286312014345 | 2700.95481943 | 597.7286312014345 | 7.24704146802 |
| 595.7108816521048 | 489.093717937 | 595.7108816521048 | 2696.44313728 | 595.7108816521048 | 6.40723668633 |
| 593.7067088858104 | 475.385065791 | 593.7067088858104 | 2689.13070959 | 593.7067088858104 | 5.65534222265 |
| 591.7159763313609 | 461.876729277 | 591.7159763313609 | 2679.22476909 | 591.7159763313609 | 4.98328942002 |
| 589.7385492431688 | 448.554603256 | 589.7385492431688 | 2666.94572247 | 589.7385492431688 | 4.38363034383 |
| 587.7742946708463 | 435.405245616 | 587.7742946708463 | 2652.52454109 | 587.7742946708463 | 3.8494998861 |
| 585.8230814294083 | 422.416135003 | 585.8230814294083 | 2636.20005417 | 585.8230814294083 | 3.37457989994 |
| 583.8847800700661 | 409.575901034 | 583.8847800700661 | 2618.2161838 | 583.8847800700661 | 2.95306514395 |
| 581.9592628516003 | 396.874523863 | 581.9592628516003 | 2598.81916242 | 581.9592628516003 | 2.57963085966 |
| 580.046403712297 | 384.303500665 | 580.046403712297 | 2578.25477341 | 580.046403712297 | 2.24940184226 |
| 578.1460782424359 | 371.855977315 | 578.1460782424359 | 2556.76565537 | 578.1460782424359 | 1.95792289703 |
| 576.2581636573184 | 359.526844285 | 576.2581636573184 | 2534.58870946 | 576.2581636573184 | 1.70113060044 |
| 574.3825387708214 | 347.312796484 | 574.3825387708214 | 2511.95264735 | 574.3825387708214 | 1.47532630646 |
| 572.5190839694656 | 335.212357446 | 572.5190839694656 | 2489.07571508 | 572.5190839694656 | 1.27715035593 |
| 570.6676811869887 | 323.225868948 | 570.6676811869887 | 2466.16362513 | 570.6676811869887 | 1.10355745945 |
| 568.8282138794084 | 311.355447705 | 568.8282138794084 | 2443.40772542 | 568.8282138794084 | 0.951793233831 |
| 567.000567000567 | 299.604911351 | 567.000567000567 | 2420.98342981 | 567.000567000567 | 0.819371878055 |
| 565.1846269781461 | 287.979676374 | 565.1846269781461 | 2399.04893049 | 565.1846269781461 | 0.704054978161 |
| 563.3802816901408 | 276.486631059 | 563.3802816901408 | 2377.74420749 | 563.3802816901408 | 0.603831431813 |
| 561.5874204417821 | 265.13398681 | 561.5874204417821 | 2357.19034576 | 561.5874204417821 | 0.516898482634 |
| 559.8059339428997 | 253.931111459 | 559.8059339428997 | 2337.48916494 | 559.8059339428997 | 0.441643852618 |
| 558.0357142857143 | 242.888348295 | 558.0357142857143 | 2318.72316152 | 558.0357142857143 | 0.376628958074 |
| 556.2766549230483 | 232.016824639 | 556.2766549230483 | 2300.95575803 | 556.2766549230483 | 0.320573191057 |
| 554.52865064695 | 221.328253763 | 554.52865064695 | 2284.23184843 | 554.52865064695 | 0.27233924445 |
| 552.791597567717 | 210.834733862 | 552.791597567717 | 2268.57862425 | 552.791597567717 | 0.230919454855 |
| 551.0653930933137 | 200.548547663 | 551.0653930933137 | 2254.00666097 | 551.0653930933137 | 0.195423133658 |
| 549.3499359091741 | 190.481966003 | 549.3499359091741 | 2240.51124013 | 549.3499359091741 | 0.165064852925 |
| 547.645125958379 | 180.647058503 | 547.645125958379 | 2228.07387866 | 547.645125958379 | 0.139153649598 |
| 545.950864422202 | 171.055514089 | 545.950864422202 | 2216.66403356 | 545.950864422202 | 0.117083108577 |
| 544.2670537010159 | 161.718473846 | 544.2670537010159 | 2206.24094736 | 544.2670537010159 | 0.0983222829935 |
| 542.5935973955508 | 152.646378269 | 542.5935973955508 | 2196.75559768 | 542.5935973955508 | 0.0824074081713 |
| 540.9304002884962 | 143.84883062 | 540.9304002884962 | 2188.15271257 | 540.9304002884962 | 0.0689343645151 |
| 539.2773683264425 | 135.334477732 | 539.2773683264425 | 2180.37281268 | 539.2773683264425 | 0.0575518438617 |
| 537.6344086021505 | 127.11090916 | 537.6344086021505 | 2173.35424115 | 537.6344086021505 | 0.0479551735823 |
| 536.0014293371448 | 119.184575275 | 536.0014293371448 | 2167.03514264 | 536.0014293371448 | 0.039880752979 |
| 534.3783398646241 | 111.560724463 | 534.3783398646241 | 2161.3553542 | 534.3783398646241 | 0.0331010571804 |
| 532.7650506126798 | 104.24335932 | 532.7650506126798 | 2156.2581727 | 532.7650506126798 | 0.027420164776 |
| 531.1614730878186 | 97.2352113568 | 531.1614730878186 | 2151.6919661 | 531.1614730878186 | 0.0226697667851 |
| 529.5675198587819 | 90.5377335032 | 529.5675198587819 | 2147.6115986 | 529.5675198587819 | 0.0187056161831 |
| 527.9831045406547 | 84.1511094179 | 527.9831045406547 | 2143.97964403 | 527.9831045406547 | 0.0154043790431 |
| 526.4081417792595 | 78.0742784105 | 526.4081417792595 | 2140.7673652 | 526.4081417792595 | 0.012660850355 |
| 524.8425472358292 | 72.3049746138 | 524.8425472358292 | 2137.95544193 | 524.8425472358292 | 0.0103854997091 |
| 523.2862375719518 | 66.8397789058 | 523.2862375719518 | 2135.53443497 | 523.2862375719518 | 0.0085023142224 |
| 521.7391304347826 | 61.6741819829 | 521.7391304347826 | 2133.50497809 | 521.7391304347826 | 0.00694690832202 |
| 520.2011444425177 | 56.8026569298 | 520.2011444425177 | 2131.87769575 | 520.2011444425177 | 0.00566487223246 |
| 518.6721991701245 | 52.2187396002 | 518.6721991701245 | 2130.67284876 | 518.6721991701245 | 0.00461033321942 |
| 517.1522151353215 | 47.9151151345 | 517.1522151353215 | 2129.91971567 | 517.1522151353215 | 0.00374470579672 |
| 515.6411137848057 | 43.8837089704 | 515.6411137848057 | 2129.65572222 | 515.6411137848057 | 0.00303560918325 |
| 514.1388174807198 | 40.1157807685 | 514.1388174807198 | 2129.92533623 | 514.1388174807198 | 0.00245593228742 |
| 512.6452494873547 | 36.602019756 | 512.6452494873547 | 2130.77874941 | 512.6452494873547 | 0.00198302838639 |
| 511.1603339580848 | 33.3326400944 | 511.1603339580848 | 2132.2703718 | 511.1603339580848 | 0.00159802344623 |
| 509.683995922528 | 30.2974749961 | 509.683995922528 | 2134.4571678 | 509.683995922528 | 0.00128522369211 |
| 508.2161612739285 | 27.486068444 | 508.2161612739285 | 2137.39686603 | 508.2161612739285 | 0.00103160958177 |
| 506.7567567567567 | 24.8877635027 | 506.7567567567567 | 2141.14607753 | 506.7567567567567 | 0.000826404759977 |
| 505.3057099545225 | 22.4917863556 | 505.3057099545225 | 2145.75835859 | 505.3057099545225 | 0.000660709878317 |
| 503.8629492777964 | 20.287325342 | 503.8629492777964 | 2151.28225584 | 503.8629492777964 | 0.000527192355821 |
| 502.4284039524367 | 18.2636044121 | 502.4284039524367 | 2157.75937138 | 502.4284039524367 | 0.000419824236758 |
| 501.00200400801606 | 16.4099505543 | 501.00200400801606 | 2165.22248598 | 501.00200400801606 | 0.000333661276793 |
| 499.5836802664446 | 14.7158548819 | 499.5836802664446 | 2173.69377718 | 499.5836802664446 | 0.000264657264274 |
| 498.1733643307871 | 13.1710271894 | 498.1733643307871 | 2183.18316758 | 498.1733643307871 | 0.000209508365698 |
| 496.7709885742673 | 11.7654439039 | 496.7709885742673 | 2193.68683685 | 496.7709885742673 | 0.000165522980312 |
| 495.3764861294584 | 10.4893894592 | 495.3764861294584 | 2205.18592775 | 495.3764861294584 | 0.000130513205078 |
| 493.98979087765514 | 9.33349121507 | 493.98979087765514 | 2217.64547348 | 493.98979087765514 | 0.000102704554658 |
| 492.61083743842363 | 8.28874812303 | 492.61083743842363 | 2231.01356984 | 492.61083743842363 | 8.06610582479e-05 |
| 491.2395611593253 | 7.34655340928 | 491.2395611593253 | 2245.22081155 | 491.2395611593253 | 6.32232724543e-05 |
| 489.8758981058131 | 6.49871160275 | 489.8758981058131 | 2260.18000732 | 489.8758981058131 | 4.94571129254e-05 |
| 488.5197850512946 | 5.73745028065 | 488.5197850512946 | 2275.78618392 | 488.5197850512946 | 3.86117229434e-05 |
| 487.17115946735953 | 5.05542693858 | 487.17115946735953 | 2291.91688404 | 487.17115946735953 | 3.00848699152e-05 |
| 485.82995951416996 | 4.44573141553 | 485.82995951416996 | 2308.43275817 | 485.82995951416996 | 2.33945956122e-05 |
| 484.49612403100775 | 3.90188431852 | 484.49612403100775 | 2325.17844527 | 484.49612403100775 | 1.81560476244e-05 |
| 483.16959252697694 | 3.41783189647 | 483.16959252697694 | 2341.9837324 | 483.16959252697694 | 1.40625919132e-05 |
| 481.8503051718599 | 2.98793781077 | 481.8503051718599 | 2358.66497846 | 481.8503051718599 | 1.08704532942e-05 |
| 480.5382027871216 | 2.60697224025 | 480.5382027871216 | 2375.02678292 | 480.5382027871216 | 8.38625548603e-06 |
| 479.23322683706067 | 2.27009874352 | 479.23322683706067 | 2390.86387601 | 479.23322683706067 | 6.45693801201e-06 |
| 477.9353194201051 | 1.97285928137 | 477.9353194201051 | 2405.96320332 | 477.9353194201051 | 4.96161637762e-06 |
| 476.64442326024783 | 1.71115777835 | 476.64442326024783 | 2420.10617435 | 476.64442326024783 | 3.80502691175e-06 |
| 475.3604816986214 | 1.48124257568 | 475.3604816986214 | 2433.07104202 | 475.3604816986214 | 2.91226049843e-06 |
| 474.08343868520853 | 1.27968809904 | 474.08343868520853 | 2444.63537761 | 474.08343868520853 | 2.22454193878e-06 |
| 472.8132387706856 | 1.10337603439 | 472.8132387706856 | 2454.57860453 | 472.8132387706856 | 1.69585542817e-06 |
| 471.5498270983967 | 0.949476274434 | 471.5498270983967 | 2462.68455318 | 471.5498270983967 | 1.29025296253e-06 |
| 470.29314939645707 | 0.815427867431 | 470.29314939645707 | 2468.74399881 | 470.29314939645707 | 9.79712567429e-07 |
| 469.04315196998124 | 0.698920169903 | 469.04315196998124 | 2472.55714494 | 469.04315196998124 | 7.42438073183e-07 |
| 467.7997816934352 | 0.597874375582 | 467.7997816934352 | 2473.93601572 | 467.7997816934352 | 5.61512594374e-07 |
| 466.5629860031104 | 0.510425565039 | 466.5629860031104 | 2472.70672239 | 466.5629860031104 | 4.23834641698e-07 |
| 465.33271288971605 | 0.434905394232 | 465.33271288971605 | 2468.71157108 | 465.33271288971605 | 3.19279514729e-07 |
| 464.10891089108907 | 0.369825515888 | 464.10891089108907 | 2461.81098197 | 464.10891089108907 | 2.40039818484e-07 |
| 462.8915290850177 | 0.313861805271 | 462.8915290850177 | 2451.88519322 | 462.8915290850177 | 1.80108053429e-07 |
| 461.68051708217905 | 0.265839441686 | 461.68051708217905 | 2438.83572617 | 461.68051708217905 | 1.34871616207e-07 |
| 460.47582501918646 | 0.224718878993 | 460.47582501918646 | 2422.58659305 | 460.47582501918646 | 1.00796524547e-07 |
| 459.2774035517452 | 0.189582722455 | 459.2774035517452 | 2403.08523181 | 459.2774035517452 | 7.51810007738e-08 |
| 458.0852038479157 | 0.159623515395 | 458.0852038479157 | 2380.30315771 | 458.0852038479157 | 5.59639266384e-08 |
| 456.89917758148033 | 0.134132427296 | 456.89917758148033 | 2354.23632548 | 456.89917758148033 | 4.1576293572e-08 |
| 455.7192769254139 | 0.112488825031 | 455.7192769254139 | 2324.90520044 | 455.7192769254139 | 3.08262618576e-08 |
| 454.54545454545456 | 0.094150700747 | 454.54545454545456 | 2292.35454141 | 454.54545454545456 | 2.28104284963e-08 |
| 453.3776635937736 | 0.078645923391 | 453.3776635937736 | 2256.65290263 | 453.3776635937736 | 1.68454842078e-08 |
| 452.2158577027434 | 0.06556427584 | 452.2158577027434 | 2217.89186589 | 452.2158577027434 | 1.24156944874e-08 |
| 451.05999097880016 | 0.0545502358901 | 451.05999097880016 | 2176.1850179 | 451.05999097880016 | 9.13263263417e-09 |
| 449.9100179964007 | 0.045296456852 | 449.9100179964007 | 2131.66669148 | 449.9100179964007 | 6.70437659685e-09 |
| 448.7658937920718 | 0.0375379020211 | 448.7658937920718 | 2084.49049223 | 448.7658937920718 | 4.91199874937e-09 |
| 447.6275738585497 | 0.0310465867111 | 447.6275738585497 | 2034.8276348 | 447.6275738585497 | 3.59166248128e-09 |
| 446.49501413900873 | 0.0256268817108 | 446.49501413900873 | 1982.86511532 | 446.49501413900873 | 2.62101921473e-09 |
| 445.36817102137763 | 0.0211113328236 | 445.36817102137763 | 1928.80374791 | 445.36817102137763 | 1.90889614258e-09 |
| 444.247001332741 | 0.0173569524604 | 444.247001332741 | 1872.85609478 | 444.247001332741 | 1.38749618549e-09 |
| 443.13146233382565 | 0.0142419409668 | 443.13146233382565 | 1815.24431947 | 443.13146233382565 | 1.0065113829e-09 |
| 442.02151171357 | 0.0116627973841 | 442.02151171357 | 1756.19799362 | 442.02151171357 | 7.2869027462e-10 |
| 440.9171075837742 | 0.009531781582 | 440.9171075837742 | 1695.95188666 | 440.9171075837742 | 5.26507594321e-10 |
| 439.8182084738308 | 0.00777469208438 | 439.8182084738308 | 1634.74376744 | 439.8182084738308 | 3.79667729572e-10 |
| 438.72477332553376 | 0.00632892637834 | 438.72477332553376 | 1572.81224532 | 438.72477332553376 | 2.73237375192e-10 |
| 437.636761487965 | 0.00514179299034 | 437.636761487965 | 1510.39467673 | 437.636761487965 | 1.96251910798e-10 |
| 436.5541327124563 | 0.00416904709272 | 436.5541327124563 | 1447.72516107 | 436.5541327124563 | 1.40677631822e-10 |
| 435.4768471476266 | 0.00337362382875 | 435.4768471476266 | 1385.03264762 | 435.4768471476266 | 1.0064068067e-10 |
| 434.4048653344918 | 0.00272454588792 | 434.4048653344918 | 1322.53917257 | 434.4048653344918 | 7.18554048002e-11 |
| 433.3381482016467 | 0.00219598410183 | 433.3381482016467 | 1260.4582425 | 433.3381482016467 | 5.12014983538e-11 |
| 432.2766570605187 | 0.00176645194993 | 432.2766570605187 | 1198.9933778 | 432.2766570605187 | 3.64118925299e-11 |
| 431.22035360069 | 0.00141811685099 | 431.22035360069 | 1138.33682659 | 431.22035360069 | 2.5842896208e-11 |
| 430.1691998852882 | 0.00113621296556 | 430.1691998852882 | 1078.66845674 | 430.1691998852882 | 1.83052835705e-11 |
| 429.1231583464454 | 0.000908541942464 | 429.1231583464454 | 1020.15483061 | 429.1231583464454 | 1.29404411729e-11 |
| 428.0821917808219 | 0.000725049609639 | 428.0821917808219 | 962.948464335 | 428.0821917808219 | 9.12975338599e-12 |
| 427.0462633451957 | 0.000577468038685 | 427.0462633451957 | 907.18727084 | 427.0462633451957 | 6.42845117835e-12 |
| 426.01533655211585 | 0.00045901370769 | 426.01533655211585 | 852.994183021 | 426.01533655211585 | 4.51742541932e-12 |
| 424.9893752656184 | 0.00036413365457 | 424.9893752656184 | 800.476951429 | 424.9893752656184 | 3.16820276517e-12 |
| 423.96834369700395 | 0.00028829256012 | 423.96834369700395 | 749.728108517 | 423.96834369700395 | 2.21754417725e-12 |
| 422.9522064006767 | 0.000227794634045 | 422.9522064006767 | 700.825089746 | 422.9522064006767 | 1.54906244484e-12 |
| 421.9409282700422 | 0.000179635006679 | 421.9409282700422 | 653.830500236 | 421.9409282700422 | 1.07994814093e-12 |
| 420.93447453346425 | 0.000141376062071 | 420.93447453346425 | 608.792514366 | 420.93447453346425 | 7.51405219225e-13 |
| 419.9328107502799 | 0.000111044793172 | 419.9328107502799 | 565.745394706 | 419.9328107502799 | 5.21774490081e-13 |
| 418.93590280687056 | 8.70478249315e-05 | 418.93590280687056 | 524.710115955 | 418.93590280687056 | 3.61600319074e-13 |
| 417.94371691278906 | 6.81012441909e-05 | 417.94371691278906 | 485.695079076 | 417.94371691278906 | 2.50099080115e-13 |
| 416.9562195969423 | 5.31728037393e-05 | 416.9562195969423 | 448.69690068 | 416.9562195969423 | 1.72636534477e-13 |
| 415.97337770382694 | 4.14344387825e-05 | 415.97337770382694 | 413.701262729 | 415.97337770382694 | 1.18929789195e-13 |
| 414.99515838981876 | 3.22233539019e-05 | 414.99515838981876 | 380.683807944 | 414.99515838981876 | 8.17684777436e-14 |
| 414.0215291195142 | 2.50102133448e-05 | 414.0215291195142 | 349.611066797 | 414.0215291195142 | 5.61071875237e-14 |
| 413.0524576621231 | 1.93732026816e-05 | 413.0524576621231 | 320.441402642 | 413.0524576621231 | 3.84227467699e-14 |
| 412.08791208791206 | 1.49769304721e-05 | 412.08791208791206 | 293.125962369 | 412.08791208791206 | 2.62600554815e-14 |
| 411.1278607646978 | 1.15553090994e-05 | 411.1278607646978 | 267.60962093 | 411.1278607646978 | 1.79118395866e-14 |
| 410.17227235438884 | 8.89769836293e-06 | 410.17227235438884 | 243.831909153 | 410.17227235438884 | 1.21933225823e-14 |
| 409.22111580957574 | 6.83771736228e-06 | 409.22111580957574 | 221.727915379 | 409.22111580957574 | 8.28402223231e-15 |
| 408.2743603701687 | 5.24423280819e-06 | 408.2743603701687 | 201.229152647 | 408.2743603701687 | 5.61691390917e-15 |
| 407.33197556008145 | 4.01411795123e-06 | 407.33197556008145 | 182.264384366 | 407.33197556008145 | 3.80094511171e-15 |
| 406.39393118396094 | 3.0664482659e-06 | 406.39393118396094 | 164.760402601 | 406.39393118396094 | 2.5669815507e-15 |
| 405.46019732396263 | 2.33785998643e-06 | 405.46019732396263 | 148.642754289 | 405.46019732396263 | 1.73017970994e-15 |
| 404.53074433656957 | 1.7788474055e-06 | 404.53074433656957 | 133.836411856 | 404.53074433656957 | 1.16384997013e-15 |
| 403.6055428494551 | 1.35081610679e-06 | 403.6055428494551 | 120.266385782 | 403.6055428494551 | 7.8134007934e-16 |
| 402.68456375838923 | 1.02374337206e-06 | 402.68456375838923 | 107.858277683 | 402.68456375838923 | 5.23504623816e-16 |
| 401.76777822418643 | 7.74325052791e-07 | 401.76777822418643 | 96.5387734392 | 401.76777822418643 | 3.50056590344e-16 |
| 400.85515766969536 | 5.84511219839e-07 | 400.85515766969536 | 86.2360767158 | 400.85515766969536 | 2.33611012252e-16 |
| 399.9466737768297 | 4.40351748346e-07 | 399.9466737768297 | 76.880284031 | 399.9466737768297 | 1.55591432915e-16 |
| 399.0422984836393 | 3.31088372025e-07 | 399.0422984836393 | 68.4037031457 | 399.0422984836393 | 1.03422579547e-16 |
| 398.14200398142003 | 2.4844225409e-07 | 398.14200398142003 | 60.7411171465 | 398.14200398142003 | 6.86092017885e-17 |
| 397.24576271186436 | 1.86056274775e-07 | 397.24576271186436 | 53.8299970497 | 397.24576271186436 | 4.5424136823e-17 |
| 396.3535473642489 | 1.39059450307e-07 | 396.3535473642489 | 47.6106661326 | 396.3535473642489 | 3.00143066539e-17 |
| 395.46533087266016 | 1.03727526326e-07 | 395.46533087266016 | 42.0264194846 | 395.46533087266016 | 1.97928012026e-17 |
| 394.5810864132579 | 7.72191218573e-08 | 394.5810864132579 | 37.0236024684 | 394.5810864132579 | 1.30263734596e-17 |
| 393.7007874015748 | 5.73710792394e-08 | 393.7007874015748 | 32.5516519069 | 393.7007874015748 | 8.55612463681e-18 |
| 392.82440748985204 | 4.25400997506e-08 | 392.82440748985204 | 28.5631038557 | 392.82440748985204 | 5.60877469049e-18 |
| 391.9519205644107 | 3.14804774407e-08 | 391.9519205644107 | 25.0135718122 | 391.9519205644107 | 3.66940966957e-18 |
| 391.08330074305826 | 2.32499186837e-08 | 391.08330074305826 | 21.8616991373 | 391.08330074305826 | 2.39586157488e-18 |
| 390.2185223725286 | 1.71371614035e-08 | 390.2185223725286 | 19.0690893497 | 390.2185223725286 | 1.56122162415e-18 |
| 389.3575600259571 | 1.26064746873e-08 | 389.3575600259571 | 16.600217791 | 389.3575600259571 | 1.01532413065e-18 |
| 388.5003885003885 | 9.25519865341e-09 | 388.5003885003885 | 14.4223279707 | 388.5003885003885 | 6.58995079893e-19 |
| 387.6469828143171 | 6.7813342759e-09 | 387.6469828143171 | 12.5053156789 | 387.6469828143171 | 4.26871293852e-19 |
| 386.7973182052604 | 4.95886036682e-09 | 386.7973182052604 | 10.8216037232 | 386.7973182052604 | 2.75961846876e-19 |
| 385.95137012736393 | 3.61897784476e-09 | 385.95137012736393 | 9.34600989463 | 385.95137012736393 | 1.78048553251e-19 |
| 385.1091142490372 | 2.63589002849e-09 | 385.1091142490372 | 8.05561051547 | 385.1091142490372 | 1.14647653214e-19 |
| 384.2705264506212 | 1.9160461595e-09 | 384.2705264506212 | 6.92960166471 | 384.2705264506212 | 7.36765367893e-20 |
| 383.4355828220859 | 1.39002293132e-09 | 383.4355828220859 | 5.94915992623 | 383.4355828220859 | 4.72531267547e-20 |
| 382.6042596607575 | 1.0064107781e-09 | 382.6042596607575 | 5.09730425771 | 382.6042596607575 | 3.0246088093e-20 |
| 381.77653346907607 | 7.27220160157e-10 | 381.77653346907607 | 4.35876034419 | 381.77653346907607 | 1.93216934682e-20 |
| 380.95238095238096 | 5.24437643253e-10 | 380.95238095238096 | 3.71982857688 | 380.95238095238096 | 1.23185184039e-20 |
| 380.1317790167258 | 3.77449730966e-10 | 380.1317790167258 | 3.16825659014 | 380.1317790167258 | 7.83806894033e-21 |
| 379.31470476672143 | 2.71120089397e-10 | 379.31470476672143 | 2.69311709704 | 379.31470476672143 | 4.9773364089e-21 |
| 378.5011355034065 | 1.94357629482e-10 | 378.5011355034065 | 2.28469158898 | 378.5011355034065 | 3.15443965895e-21 |
| 377.69104872214524 | 1.39052500612e-10 | 377.69104872214524 | 1.93436030646 | 377.69104872214524 | 1.9951923319e-21 |
| 376.88442211055275 | 9.92872148518e-11 | 376.88442211055275 | 1.63449874784 | 376.88442211055275 | 1.25946070817e-21 |
| 376.081233546446 | 7.07530503708e-11 | 376.081233546446 | 1.37838085899 | 376.081233546446 | 7.93454071092e-22 |
| 375.28146109582184 | 5.0319268977e-11 | 375.28146109582184 | 1.16008894008 | 375.28146109582184 | 4.98880209285e-22 |
| 374.48508301086 | 3.57158337533e-11 | 374.48508301086 | 0.974430214419 | 374.48508301086 | 3.1304593733e-22 |
| 373.69207772795215 | 2.53002362729e-11 | 373.69207772795215 | 0.81685992813 | 373.69207772795215 | 1.96045636984e-22 |
| 372.9024238657551 | 1.78865127155e-11 | 372.9024238657551 | 0.683410786749 | 372.9024238657551 | 1.22530330317e-22 |
| 372.11610022326965 | 1.26201375555e-11 | 372.11610022326965 | 0.570628485354 | 372.11610022326965 | 7.64306125688e-23 |
| 371.33308577794276 | 8.88668560428e-12 | 371.33308577794276 | 0.475513050304 | 371.33308577794276 | 4.75804329635e-23 |
| 370.55335968379444 | 6.24529356041e-12 | 370.55335968379444 | 0.395465682734 | 370.55335968379444 | 2.95615194008e-23 |
| 369.7769012695673 | 4.38029351457e-12 | 369.7769012695673 | 0.328240774934 | 369.7769012695673 | 1.83299984328e-23 |
| 369.0036900369003 | 3.06613224258e-12 | 369.0036900369003 | 0.27190275968 | 369.0036900369003 | 1.13431954867e-23 |
| 368.23370565852457 | 2.14198223897e-12 | 368.23370565852457 | 0.22478744822 | 368.23370565852457 | 7.00560616278e-24 |
| 367.4669279764821 | 1.49340693084e-12 | 367.4669279764821 | 0.185467514015 | 367.4669279764821 | 4.31810634634e-24 |
| 366.7033370003667 | 1.03914887899e-12 | 366.7033370003667 | 0.152721785422 | 366.7033370003667 | 2.6563069726e-24 |
| 365.9429129055867 | 7.21630196747e-13 | 365.9429129055867 | 0.125508020398 | 365.9429129055867 | 1.63079925251e-24 |
| 365.1856360316494 | 5.0013694423e-13 | 365.1856360316494 | 0.102938849245 | 365.1856360316494 | 9.99217561541e-25 |
| 364.4314868804664 | 3.45939766659e-13 | 364.4314868804664 | 0.0842605865506 | 364.4314868804664 | 6.11022110519e-25 |
| 363.68044611468054 | 2.38808265091e-13 | 363.68044611468054 | 0.0688346303195 | 363.68044611468054 | 3.7289890384e-25 |
| 362.93249455601256 | 1.6452637629e-13 | 362.93249455601256 | 0.0561211841325 | 362.93249455601256 | 2.27123776381e-25 |
| 362.1876131836291 | 1.13125112678e-13 | 362.1876131836291 | 0.0456650566335 | 362.1876131836291 | 1.38061124863e-25 |
| 361.4457831325301 | 7.76282562118e-14 | 361.4457831325301 | 0.0370833112838 | 361.4457831325301 | 8.37563084313e-26 |
| 360.7069856919562 | 5.31640367762e-14 | 360.7069856919562 | 0.0300545578069 | 360.7069856919562 | 5.07108582271e-26 |
| 359.97120230381563 | 3.63373608996e-14 | 359.97120230381563 | 0.0243096948138 | 359.97120230381563 | 3.06423244501e-26 |
| 359.2384145611304 | 2.47871233596e-14 | 359.2384145611304 | 0.0196239305288 | 359.2384145611304 | 1.84790553319e-26 |
| 358.50860420650093 | 1.68747049223e-14 | 358.50860420650093 | 0.0158099251726 | 358.50860420650093 | 1.11218014615e-26 |
| 357.7817531305903 | 1.14652508132e-14 | 357.7817531305903 | 0.0127119142816 | 357.7817531305903 | 6.68048238751e-27 |
| 357.057843370626 | 7.77442441289e-15 | 357.057843370626 | 0.0102006869686 | 357.057843370626 | 4.00477223802e-27 |
| 356.33685710892024 | 5.26126581729e-15 | 356.33685710892024 | 0.00816930682624 | 356.33685710892024 | 2.39599072736e-27 |
| 355.6187766714082 | 3.55344466212e-15 | 355.6187766714082 | 0.00652947580342 | 355.6187766714082 | 1.43063800302e-27 |
| 354.9035845262037 | 2.39522426069e-15 | 354.9035845262037 | 0.00520845296754 | 354.9035845262037 | 8.52533973583e-28 |
| 354.1912632821723 | 1.61131377704e-15 | 354.1912632821723 | 0.00414645061342 | 354.1912632821723 | 5.07026834528e-28 |
| 353.48179568752204 | 1.08181094769e-15 | 353.48179568752204 | 0.00329443973077 | 353.48179568752204 | 3.00945260752e-28 |
| 352.77516462841015 | 7.24869684254e-16 | 352.77516462841015 | 0.00261230544526 | 352.77516462841015 | 1.78271280887e-28 |
| 352.07135312756714 | 4.84736602848e-16 | 352.07135312756714 | 0.00206730075375 | 352.07135312756714 | 1.05393195966e-28 |
| 351.3703443429374 | 3.2351096563e-16 | 351.3703443429374 | 0.0016327537438 | 351.3703443429374 | 6.21843468059e-29 |
| 350.6721215663355 | 2.15481261885e-16 | 350.6721215663355 | 0.00128698958104 | 350.6721215663355 | 3.66173484023e-29 |
| 349.9766682221185 | 1.43240994457e-16 | 349.9766682221185 | 0.00101243392905 | 349.9766682221185 | 2.15193931837e-29 |
| 349.2839678658749 | 9.50303782399e-17 | 349.2839678658749 | 0.000794869197439 | 349.2839678658749 | 1.26214851928e-29 |
| 348.59400418312805 | 6.29208964233e-17 | 348.59400418312805 | 0.000622819154688 | 348.59400418312805 | 7.38802262196e-30 |
| 347.90676098805517 | 4.15781017635e-17 | 347.90676098805517 | 0.000487041053281 | 347.90676098805517 | 4.31601836539e-30 |
| 347.2222222222222 | 2.74202700384e-17 | 347.2222222222222 | 0.000380107549148 | 347.2222222222222 | 2.51637675329e-30 |
| 346.54037195333257 | 1.80474609227e-17 | 346.54037195333257 | 0.000296063409117 | 346.54037195333257 | 1.46421670681e-30 |
| 345.8611943739912 | 1.18548975688e-17 | 345.8611943739912 | 0.000230144336288 | 345.8611943739912 | 8.5030036264e-31 |
| 345.1846738004832 | 7.7717140801e-18 | 345.1846738004832 | 0.000178547248858 | 345.1846738004832 | 4.92806790801e-31 |
| 344.5107946715664 | 5.08479134632e-18 | 344.5107946715664 | 0.000138243063347 | 344.5107946715664 | 2.85048208669e-31 |
| 343.8395415472779 | 3.32021931157e-18 | 343.8395415472779 | 0.000106824495308 | 343.8395415472779 | 1.64549763143e-31 |
| 343.17089910775564 | 2.16370335816e-18 | 343.17089910775564 | 8.23826324228e-05 | 343.17089910775564 | 9.48011312766e-32 |
| 342.50485215207215 | 1.40723289996e-18 | 342.50485215207215 | 6.34070860387e-05 | 342.50485215207215 | 5.45088589908e-32 |
| 341.84138559708293 | 9.13422181818e-19 | 341.84138559708293 | 4.87054140053e-05 | 341.84138559708293 | 3.12793684342e-32 |
| 341.1804844762879 | 5.91717537032e-19 | 341.1804844762879 | 3.73382533122e-05 | 341.1804844762879 | 1.79137361366e-32 |
| 340.522133938706 | 3.82555667918e-19 | 340.522133938706 | 2.85672259821e-05 | 340.522133938706 | 1.02388618499e-32 |
| 339.86631924776253 | 2.46838076669e-19 | 339.86631924776253 | 2.18132037942e-05 | 339.86631924776253 | 5.84056022498e-33 |
| 339.2130257801899 | 1.58952359779e-19 | 339.2130257801899 | 1.66229522493e-05 | 339.2130257801899 | 3.325022916e-33 |
| 338.56223902494077 | 1.0215488018e-19 | 338.56223902494077 | 1.26425352042e-05 | 338.56223902494077 | 1.88917457484e-33 |
| 337.91394458211306 | 6.55222150379e-20 | 337.91394458211306 | 9.59616042407e-06 | 337.91394458211306 | 1.07124018124e-33 |
| 337.2681281618887 | 4.19425987492e-20 | 337.2681281618887 | 7.26939288238e-06 | 337.2681281618887 | 6.06232101369e-34 |
| 336.6247755834829 | 2.67953487368e-20 | 336.6247755834829 | 5.49586551328e-06 | 336.6247755834829 | 3.42395712456e-34 |
| 335.9838727741068 | 1.70844422327e-20 | 335.9838727741068 | 4.14678353689e-06 | 335.9838727741068 | 1.92998982282e-34 |
| 335.3454057679409 | 1.08712506397e-20 | 335.3454057679409 | 3.12265459256e-06 | 335.3454057679409 | 1.08572298756e-34 |
| 334.709360705121 | 6.9039164281e-21 | 334.709360705121 | 2.34678791969e-06 | 334.709360705121 | 6.0956547366e-35 |
| 334.07572383073494 | 4.3757132658e-21 | 334.07572383073494 | 1.76019609543e-06 | 334.07572383073494 | 3.41553704693e-35 |
| 333.44448149383123 | 2.76783054975e-21 | 333.44448149383123 | 1.31760603683e-06 | 333.44448149383123 | 1.91000697582e-35 |
| 332.81562014643885 | 1.74729990965e-21 | 332.81562014643885 | 9.84345163985e-07 | 332.81562014643885 | 1.06597794551e-35 |
| 332.1891263425977 | 1.1008616009e-21 | 332.1891263425977 | 7.33916347293e-07 | 332.1891263425977 | 5.93743404701e-36 |
| 331.5649867374005 | 6.92205913746e-22 | 331.5649867374005 | 5.46113640119e-07 | 331.5649867374005 | 3.30055284446e-36 |
| 330.9431880860452 | 4.3438538031e-22 | 330.9431880860452 | 4.05561573886e-07 | 330.9431880860452 | 1.8310992955e-36 |
| 330.323717242898 | 2.72052303876e-22 | 330.323717242898 | 3.00585404572e-07 | 330.323717242898 | 1.01385165285e-36 |
| 329.70656116056705 | 1.70046199097e-22 | 329.70656116056705 | 2.22339329254e-07 | 329.70656116056705 | 5.60240169155e-37 |
| 329.0917068889864 | 1.06076397678e-22 | 329.0917068889864 | 1.64135305049e-07 | 329.0917068889864 | 3.08966496606e-37 |
| 328.47914157451 | 6.60401285002e-23 | 328.47914157451 | 1.20927489064e-07 | 328.47914157451 | 1.70053605876e-37 |
| 327.86885245901635 | 4.10331038582e-23 | 327.86885245901635 | 8.89171178494e-08 | 327.86885245901635 | 9.34109126288e-38 |
| 327.26082687902255 | 2.54447473081e-23 | 327.26082687902255 | 6.5250378013e-08 | 327.26082687902255 | 5.12090475115e-38 |
| 326.6550522648083 | 1.5747050782e-23 | 326.6550522648083 | 4.77879059222e-08 | 326.6550522648083 | 2.80177395529e-38 |
| 326.05151613955 | 9.72607528207e-24 | 326.05151613955 | 3.4929332086e-08 | 326.05151613955 | 1.52987801142e-38 |
| 325.4502061184639 | 5.99533339081e-24 | 325.4502061184639 | 2.54800265057e-08 | 325.4502061184639 | 8.33715401508e-39 |
| 324.8511099079588 | 3.6883010586e-24 | 324.8511099079588 | 1.8550122536e-08 | 324.8511099079588 | 4.53436168881e-39 |
| 324.25421530479895 | 2.26452281701e-24 | 324.25421530479895 | 1.34781722763e-08 | 324.25421530479895 | 2.46122777192e-39 |
| 323.65951019527455 | 1.38760019308e-24 | 323.65951019527455 | 9.7735544456e-09 | 323.65951019527455 | 1.33329044765e-39 |
| 323.0669825543829 | 8.48573207186e-25 | 323.0669825543829 | 7.07312581307e-09 | 323.0669825543829 | 7.20833631898e-40 |
| 322.4766204450177 | 5.1790677452e-25 | 322.4766204450177 | 5.10866638698e-09 | 322.4766204450177 | 3.88940016197e-40 |
| 321.88841201716735 | 3.15464999305e-25 | 321.88841201716735 | 3.6824852446e-09 | 321.88841201716735 | 2.0944380905e-40 |
| 321.3023455071222 | 1.91773274604e-25 | 321.3023455071222 | 2.64918203316e-09 | 321.3023455071222 | 1.1256146586e-40 |
| 320.71840923669015 | 1.16348904805e-25 | 320.71840923669015 | 1.90204111125e-09 | 320.71840923669015 | 6.03739048197e-41 |
| 320.1365916124213 | 7.04488370274e-26 | 320.1365916124213 | 1.3629041419e-09 | 320.1365916124213 | 3.2318121124e-41 |
| 319.5568811248402 | 4.25718643505e-26 | 319.5568811248402 | 9.74648538946e-10 | 319.5568811248402 | 1.72655435719e-41 |
| 318.97926634768737 | 2.56749037473e-26 | 318.97926634768737 | 6.95613615207e-10 | 318.97926634768737 | 9.20559194997e-42 |
| 318.40373593716834 | 1.54536933992e-26 | 318.40373593716834 | 4.95479196439e-10 | 318.40373593716834 | 4.89847050936e-42 |
| 317.8302786312109 | 9.28310092397e-27 | 317.8302786312109 | 3.52224927456e-10 | 317.8302786312109 | 2.60139671937e-42 |
| 317.2588832487309 | 5.56533312973e-27 | 317.2588832487309 | 2.49891834403e-10 | 317.2588832487309 | 1.37876417882e-42 |
| 316.6895386889053 | 3.32986457997e-27 | 316.6895386889053 | 1.7693809816e-10 | 316.6895386889053 | 7.29307547475e-43 |
| 316.1222339304531 | 1.98837956519e-27 | 316.1222339304531 | 1.25033951812e-10 | 316.1222339304531 | 3.85007099011e-43 |
| 315.55695803092453 | 1.18497537002e-27 | 315.55695803092453 | 8.81803612018e-11 | 315.55695803092453 | 2.02844893804e-43 |
| 314.99370012599746 | 7.04785026717e-28 | 314.99370012599746 | 6.20659064099e-11 | 314.99370012599746 | 1.06658810273e-43 |
| 314.432449428781 | 4.18351496758e-28 | 314.432449428781 | 4.35985096652e-11 | 314.432449428781 | 5.59714687393e-44 |
| 313.8731952291274 | 2.47835376433e-28 | 313.8731952291274 | 3.0565218075e-11 | 313.8731952291274 | 2.93139258237e-44 |
| 313.31592689295036 | 1.4652866155e-28 | 313.31592689295036 | 2.13855619366e-11 | 313.31592689295036 | 1.53221101028e-44 |
| 312.76063386155124 | 8.64607861012e-29 | 312.76063386155124 | 1.49331403614e-11 | 312.76063386155124 | 7.99282834316e-45 |
| 312.2073056509522 | 5.09158607428e-29 | 312.2073056509522 | 1.04068415182e-11 | 312.2073056509522 | 4.16121069651e-45 |
| 311.65593185123623 | 2.99243215757e-29 | 311.65593185123623 | 7.23809114484e-12 | 311.65593185123623 | 2.16210230279e-45 |
| 311.1065021258944 | 1.7552252153e-29 | 311.1065021258944 | 5.02419477238e-12 | 311.1065021258944 | 1.12116642957e-45 |
| 310.5590062111801 | 1.02749258963e-29 | 310.5590062111801 | 3.48053655973e-12 | 310.5590062111801 | 5.80231422745e-46 |
| 310.01343391546965 | 6.0029103914e-30 | 310.01343391546965 | 2.40637465984e-12 | 310.01343391546965 | 2.99688250627e-46 |
| 309.4697751186301 | 3.50011517505e-30 | 309.4697751186301 | 1.66041865569e-12 | 309.4697751186301 | 1.54481152803e-46 |
| 308.9280197713932 | 2.03676124874e-30 | 308.9280197713932 | 1.14342919261e-12 | 308.9280197713932 | 7.94728156017e-47 |
| 308.3881578947368 | 1.18286511702e-30 | 308.3881578947368 | 7.85847472115e-13 | 308.3881578947368 | 4.08036497181e-47 |
| 307.8501795792714 | 6.85594995042e-31 | 307.8501795792714 | 5.39019600789e-13 | 307.8501795792714 | 2.09082042797e-47 |
| 307.31407498463426 | 3.96585987642e-31 | 307.31407498463426 | 3.68984538714e-13 | 307.31407498463426 | 1.06923156601e-47 |
| 306.77983433888943 | 2.28951980877e-31 | 306.77983433888943 | 2.52086211894e-13 | 306.77983433888943 | 5.4571277608e-48 |
| 306.2474479379338 | 1.31913352634e-31 | 306.2474479379338 | 1.71880785072e-13 | 306.2474479379338 | 2.77967348474e-48 |
| 305.7169061449098 | 7.58525916918e-32 | 305.7169061449098 | 1.16961486213e-13 | 305.7169061449098 | 1.4130605121e-48 |
| 305.1881993896236 | 4.35300737509e-32 | 305.1881993896236 | 7.94320444349e-14 | 305.1881993896236 | 7.16910678064e-49 |
| 304.6613181679699 | 2.49313442109e-32 | 304.6613181679699 | 5.38376278472e-14 | 304.6613181679699 | 3.63000021584e-49 |
| 304.1362530413625 | 1.42508017869e-32 | 304.1362530413625 | 3.64177746752e-14 | 304.1362530413625 | 1.83436419853e-49 |
| 303.61299463617036 | 8.12961946324e-33 | 303.61299463617036 | 2.45854524962e-14 | 303.61299463617036 | 9.25127931486e-50 |
| 303.09153364316023 | 4.6284806032e-33 | 303.09153364316023 | 1.65645758653e-14 | 303.09153364316023 | 4.6564541832e-50 |
| 302.571860816944 | 2.62992880455e-33 | 302.571860816944 | 1.11383214561e-14 | 302.571860816944 | 2.33908599037e-50 |
| 302.0539669754329 | 1.4913749647e-33 | 302.0539669754329 | 7.47474683497e-15 | 302.0539669754329 | 1.17266605662e-50 |
| 301.5378429992964 | 8.44047753081e-34 | 301.5378429992964 | 5.00622703574e-15 | 301.5378429992964 | 5.86732079841e-51 |
| 301.02347983142687 | 4.76743192847e-34 | 301.02347983142687 | 3.34627714758e-15 | 301.02347983142687 | 2.92983138349e-51 |
| 300.5108684764098 | 2.6874432139e-34 | 300.5108684764098 | 2.23228986144e-15 | 300.5108684764098 | 1.46010044161e-51 |

| **GON2-Al6** | |
| --- | --- |
| Wavelength (nm) | Abs |
| 2000.0 | 4.24548180293 |
| 1977.5873434410018 | 4.88678900059 |
| 1955.671447196871 | 5.61586164154 |
| 1934.2359767891683 | 6.44328146095 |
| 1913.265306122449 | 7.38069695972 |
| 1892.7444794952683 | 8.4409024633 |
| 1872.6591760299625 | 9.63791934812 |
| 1852.9956763434218 | 10.9870789811 |
| 1833.7408312958437 | 12.505106838 |
| 1814.8820326678765 | 14.2102071815 |
| 1796.4071856287424 | 16.1221475948 |
| 1778.3046828689983 | 18.2623425735 |
| 1760.5633802816901 | 20.6539352888 |
| 1743.1725740848342 | 23.3218765419 |
| 1726.1219792865363 | 26.2929998348 |
| 1709.4017094017095 | 29.5960913938 |
| 1693.002257336343 | 33.2619538915 |
| 1676.9144773616547 | 37.3234625293 |
| 1661.1295681063123 | 41.8156120622 |
| 1645.6390565002741 | 46.7755532786 |
| 1630.4347826086955 | 52.2426173793 |
| 1615.5088852988692 | 58.2583266547 |
| 1600.8537886872998 | 64.866389812 |
| 1586.4621893178212 | 72.1126802817 |
| 1572.3270440251572 | 80.045195822 |
| 1558.4415584415583 | 88.7139977444 |
| 1544.799176107106 | 98.1711281102 |
| 1531.3935681470139 | 108.470503295 |
| 1518.2186234817814 | 119.667782386 |
| 1505.2684395383842 | 131.82020896 |
| 1492.5373134328358 | 144.986424926 |
| 1480.0197335964478 | 159.226255227 |
| 1467.7103718199608 | 174.600462387 |
| 1455.604075691412 | 191.170470073 |
| 1443.6958614051973 | 208.998055055 |
| 1431.9809069212408 | 228.145007206 |
| 1420.4545454545455 | 248.672757439 |
| 1409.1122592766555 | 270.641973798 |
| 1397.9496738117427 | 294.112126228 |
| 1386.9625520110958 | 319.141020895 |
| 1376.1467889908256 | 345.78430531 |
| 1365.4984069185252 | 374.094945891 |
| 1355.0135501355014 | 404.122680004 |
| 1344.688480502017 | 435.913444959 |
| 1334.5195729537365 | 469.508786852 |
| 1324.5033112582782 | 504.94525262 |
| 1314.6362839614374 | 542.253769097 |
| 1304.9151805132665 | 581.459013314 |
| 1295.3367875647668 | 622.578778762 |
| 1285.8979854264894 | 665.623342717 |
| 1276.5957446808509 | 710.5948402 |
| 1267.427122940431 | 757.486650517 |
| 1258.3892617449665 | 806.282802673 |
| 1249.4793835901708 | 856.957406344 |
| 1240.6947890818858 | 909.474115302 |
| 1232.0328542094455 | 963.785630494 |
| 1223.4910277324632 | 1019.83325011 |
| 1215.0668286755772 | 1077.54647407 |
| 1206.7578439259853 | 1136.8426705 |
| 1198.5617259288852 | 1197.62681147 |
| 1190.4761904761904 | 1259.79128551 |
| 1182.4990145841543 | 1323.21579369 |
| 1174.6280344557556 | 1387.76733618 |
| 1166.8611435239206 | 1453.30029544 |
| 1159.19629057187 | 1519.65662167 |
| 1151.6314779270633 | 1586.66612558 |
| 1144.1647597254005 | 1654.14688262 |
| 1136.794240242516 | 1721.90575195 |
| 1129.5180722891566 | 1789.73901229 |
| 1122.334455667789 | 1857.43311588 |
| 1115.2416356877322 | 1924.76556025 |
| 1108.2379017362393 | 1991.50587643 |
| 1101.3215859030836 | 2057.41673059 |
| 1094.4910616563297 | 2122.25513495 |
| 1087.7447425670775 | 2185.77376182 |
| 1081.081081081081 | 2247.7223537 |
| 1074.4985673352435 | 2307.84922029 |
| 1067.995728017088 | 2365.90281211 |
| 1061.5711252653928 | 2421.63335882 |
| 1055.2233556102708 | 2474.79455902 |
| 1048.951048951049 | 2525.14530704 |
| 1042.752867570386 | 2572.45144094 |
| 1036.6275051831374 | 2616.48749526 |
| 1030.5736860185502 | 2657.03844095 |
| 1024.5901639344263 | 2693.90139444 |
| 1018.6757215619693 | 2726.88727752 |
| 1012.829169480081 | 2755.82240939 |
| 1007.0493454179255 | 2780.55001253 |
| 1001.3351134846461 | 2800.93161433 |
| 995.6853634251576 | 2816.84832718 |
| 990.0990099009902 | 2828.20199055 |
| 984.5749917952082 | 2834.91615989 |
| 979.1122715404699 | 2836.93692864 |
| 973.7098344693281 | 2834.23357126 |
| 968.3666881859263 | 2826.79899727 |
| 963.0818619582664 | 2814.65000821 |
| 957.8544061302682 | 2797.82735202 |
| 952.6833915528738 | 2776.39557139 |
| 947.5679090334806 | 2750.44264551 |
| 942.5070688030161 | 2720.07942697 |
| 937.4999999999999 | 2685.43887826 |
| 932.5458501709667 | 2646.67511485 |
| 927.643784786642 | 2603.96226434 |
| 922.7929867733004 | 2557.49315343 |
| 917.9926560587514 | 2507.47783683 |
| 913.2420091324201 | 2454.14198405 |
| 908.5402786190186 | 2397.72514199 |
| 903.8867128653209 | 2338.47889251 |
| 899.2805755395683 | 2276.66492571 |
| 894.7211452430658 | 2212.55305025 |
| 890.2077151335311 | 2146.41916289 |
| 885.7395925597874 | 2078.54319951 |
| 881.316098707403 | 2009.20708998 |
| 876.9365682548962 | 1938.69273862 |
| 872.6003490401396 | 1867.28005168 |
| 868.3068017366135 | 1795.24503171 |
| 864.0552995391705 | 1722.85795795 |
| 859.8452278589854 | 1650.38166996 |
| 855.6759840273816 | 1578.06997012 |
| 851.5469770082316 | 1506.16615865 |
| 847.457627118644 | 1434.90171263 |
| 843.4073657576608 | 1364.49511848 |
| 839.3956351426972 | 1295.15086494 |
| 835.421888053467 | 1227.05860124 |
| 831.4855875831485 | 1160.39246312 |
| 827.5862068965516 | 1095.3105668 |
| 823.7232289950576 | 1031.9546691 |
| 819.8961464881114 | 970.449989774 |
| 816.1044613710554 | 910.905190119 |
| 812.3476848090983 | 853.412500513 |
| 808.6253369272237 | 798.04798765 |
| 804.9369466058491 | 744.871951184 |
| 801.2820512820513 | 693.929438288 |
| 797.6601967561818 | 645.250863785 |
| 794.0709370037056 | 598.852722895 |
| 790.5138339920949 | 554.73838319 |
| 786.9884575026232 | 512.898942214 |
| 783.4943849569078 | 473.314137196 |
| 780.0312012480499 | 435.953293551 |
| 776.598498576236 | 400.776299216 |
| 773.1958762886597 | 367.734592489 |
| 769.8229407236336 | 336.772151693 |
| 766.4793050587633 | 307.826475849 |
| 763.1645891630628 | 280.829546444 |
| 759.8784194528876 | 255.708761401 |
| 756.6204287515762 | 232.38783339 |
| 753.390256152687 | 210.787645724 |
| 750.1875468867216 | 190.82706016 |
| 747.011952191235 | 172.423672028 |
| 743.86312918423 | 155.494509174 |
| 740.7407407407408 | 139.956672206 |
| 737.6444553725104 | 125.727914526 |
| 734.5739471106758 | 112.727161514 |
| 731.528895391368 | 100.874969067 |
| 728.5089849441475 | 90.0939224416 |
| 725.5139056831922 | 80.3089770075 |
| 722.543352601156 | 71.4477431032 |
| 719.5970256656271 | 63.4407176559 |
| 716.6746297181079 | 56.2214656343 |
| 713.7758743754462 | 49.7267547106 |
| 710.9004739336492 | 43.8966467385 |
| 708.0481472740146 | 38.6745498061 |
| 705.2186177715091 | 34.0072347047 |
| 702.4116132053383 | 29.8448196713 |
| 699.6268656716418 | 26.1407272229 |
| 696.8641114982578 | 22.8516168078 |
| 694.1230911614992 | 19.9372968675 |
| 691.4035492048858 | 17.3606197299 |
| 688.7052341597796 | 15.0873625585 |
| 686.027898467871 | 13.0860973567 |
| 683.371298405467 | 11.3280527948 |
| 680.7351940095303 | 9.78697037422 |
| 678.1193490054249 | 8.43895719747 |
| 675.5235307363206 | 7.2623373543 |
| 672.9475100942127 | 6.23750369191 |
| 670.3910614525139 | 5.34677149326 |
| 667.8539626001781 | 4.57423535874 |
| 665.335994677312 | 3.90563036891 |
| 662.8369421122403 | 3.32819840331 |
| 660.3565925599823 | 2.83056030317 |
| 657.8947368421053 | 2.40259439532 |
| 655.4511688879178 | 2.03532174139 |
| 653.0256856769699 | 1.72079833952 |
| 650.6180871828237 | 1.45201438645 |
| 648.2281763180639 | 1.22280060384 |
| 645.8557588805166 | 1.02774154511 |
| 643.5006435006435 | 0.862095725023 |
| 641.1626415900834 | 0.721722354548 |
| 638.8415672913118 | 0.60301441569 |
| 636.5372374283895 | 0.502837774588 |
| 634.2494714587738 | 0.418476004795 |
| 631.9780914261638 | 0.347580575124 |
| 629.7229219143577 | 0.288126046614 |
| 627.4837900020916 | 0.238369920014 |
| 625.2605252188412 | 0.196816777562 |
| 623.0529595015576 | 0.162186369854 |
| 620.8609271523178 | 0.133385309388 |
| 618.6842647968654 | 0.109482046022 |
| 616.5228113440197 | 0.0896848155099 |
| 614.3764079459348 | 0.0733222697594 |
| 612.2448979591836 | 0.0598265159707 |
| 610.1281269066504 | 0.0487183108757 |
| 608.0259424402108 | 0.0395941755206 |
| 605.9381943041809 | 0.0321152150743 |
| 603.864734299517 | 0.0259974467423 |
| 601.8054162487462 | 0.0210034567983 |
| 599.7600959616153 | 0.0169352248681 |
| 597.7286312014345 | 0.0136279697734 |
| 595.7108816521048 | 0.0109448864125 |
| 593.7067088858104 | 0.00877265725027 |
| 591.7159763313609 | 0.00701763501289 |
| 589.7385492431688 | 0.00560260512152 |
| 587.7742946708463 | 0.00446404728616 |
| 585.8230814294083 | 0.00354982554512 |
| 583.8847800700661 | 0.0028172449279 |
| 581.9592628516003 | 0.00223142089194 |
| 580.046403712297 | 0.00176391479738 |
| 578.1460782424359 | 0.00139159500054 |
| 576.2581636573184 | 0.00109568873049 |
| 574.3825387708214 | 0.000860994826789 |
| 572.5190839694656 | 0.000675231722892 |
| 570.6676811869887 | 0.000528498817444 |
| 568.8282138794084 | 0.000412832642747 |
| 567.000567000567 | 0.000321842067959 |
| 565.1846269781461 | 0.00025040921462 |
| 563.3802816901408 | 0.000194444858923 |
| 561.5874204417821 | 0.000150688890728 |
| 559.8059339428997 | 0.00011654793147 |
| 558.0357142857143 | 8.99635159133e-05 |
| 556.2766549230483 | 6.93053467586e-05 |
| 554.52865064695 | 5.32850636285e-05 |
| 552.791597567717 | 4.08867529186e-05 |
| 551.0653930933137 | 3.13110837366e-05 |
| 549.3499359091741 | 2.39305060946e-05 |
| 547.645125958379 | 1.82534069233e-05 |
| 545.950864422202 | 1.38955013379e-05 |
| 544.2670537010159 | 1.05570530264e-05 |
| 542.5935973955508 | 8.0047790707e-06 |
| 540.9304002884962 | 6.05750985728e-06 |
| 539.2773683264425 | 4.57485159645e-06 |
| 537.6344086021505 | 3.44824379514e-06 |
| 536.0014293371448 | 2.59392227996e-06 |
| 534.3783398646241 | 1.94739464393e-06 |
| 532.7650506126798 | 1.45911315224e-06 |
| 531.1614730878186 | 1.09109342025e-06 |
| 529.5675198587819 | 8.14278261322e-07 |
| 527.9831045406547 | 6.06487228782e-07 |
| 526.4081417792595 | 4.50825395477e-07 |
| 524.8425472358292 | 3.34451351771e-07 |
| 523.2862375719518 | 2.47625513323e-07 |
| 521.7391304347826 | 1.82976641428e-07 |
| 520.2011444425177 | 1.34937830982e-07 |
| 518.6721991701245 | 9.93137977492e-08 |
| 517.1522151353215 | 7.29496525368e-08 |
| 515.6411137848057 | 5.34779341109e-08 |
| 514.1388174807198 | 3.91258472737e-08 |
| 512.6452494873547 | 2.85687087886e-08 |
| 511.1603339580848 | 2.081877525e-08 |
| 509.683995922528 | 1.51411002277e-08 |
| 508.2161612739285 | 1.09899918522e-08 |
| 506.7567567567567 | 7.9611346867e-09 |
| 505.3057099545225 | 5.75559496326e-09 |
| 503.8629492777964 | 4.15282007336e-09 |
| 502.4284039524367 | 2.99042994449e-09 |
| 501.00200400801606 | 2.1491254406e-09 |
| 499.5836802664446 | 1.54144311006e-09 |
| 498.1733643307871 | 1.10339453566e-09 |
| 496.7709885742673 | 7.88264083229e-10 |
| 495.3764861294584 | 5.62017993465e-10 |
| 493.98979087765514 | 3.99913682715e-10 |
| 492.61083743842363 | 2.84000993459e-10 |
| 491.2395611593253 | 2.01284808402e-10 |
| 489.8758981058131 | 1.42376945867e-10 |
| 488.5197850512946 | 1.00509213164e-10 |
| 487.17115946735953 | 7.08124453405e-11 |
| 485.82995951416996 | 4.97909968736e-11 |
| 484.49612403100775 | 3.49405352868e-11 |
| 483.16959252697694 | 2.44706656213e-11 |
| 481.8503051718599 | 1.71040713817e-11 |
| 480.5382027871216 | 1.19313806258e-11 |
| 479.23322683706067 | 8.30652500229e-12 |
| 477.9353194201051 | 5.77145777545e-12 |
| 476.64442326024783 | 4.00211102676e-12 |
| 475.3604816986214 | 2.76968386484e-12 |
| 474.08343868520853 | 1.91297247892e-12 |
| 472.8132387706856 | 1.31863527877e-12 |
| 471.5498270983967 | 9.07147904582e-13 |
| 470.29314939645707 | 6.22829178505e-13 |
| 469.04315196998124 | 4.26773290914e-13 |
| 467.7997816934352 | 2.91852179989e-13 |
| 466.5629860031104 | 1.99189332113e-13 |
| 465.33271288971605 | 1.35677123966e-13 |
| 464.10891089108907 | 9.22326312086e-14 |
| 462.8915290850177 | 6.25748733947e-14 |
| 461.68051708217905 | 4.23694443951e-14 |
| 460.47582501918646 | 2.86314249566e-14 |
| 459.2774035517452 | 1.93094790903e-14 |
| 458.0852038479157 | 1.2996773515e-14 |
| 456.89917758148033 | 8.73047631196e-15 |
| 455.7192769254139 | 5.85298912618e-15 |
| 454.54545454545456 | 3.91611002274e-15 |
| 453.3776635937736 | 2.61498642056e-15 |
| 452.2158577027434 | 1.74269495557e-15 |
| 451.05999097880016 | 1.15907273642e-15 |
| 449.9100179964007 | 7.6937378553e-16 |
| 448.7658937920718 | 5.09684532621e-16 |
| 447.6275738585497 | 3.3697904625e-16 |
| 446.49501413900873 | 2.22352342935e-16 |
| 445.36817102137763 | 1.46425896511e-16 |
| 444.247001332741 | 9.62346430773e-17 |
| 443.13146233382565 | 6.31222327831e-17 |
| 442.02151171357 | 4.13209813831e-17 |
| 440.9171075837742 | 2.69958038079e-17 |
| 439.8182084738308 | 1.76018881808e-17 |
| 438.72477332553376 | 1.14540641023e-17 |
| 437.636761487965 | 7.4387046616e-18 |
| 436.5541327124563 | 4.82139126897e-18 |
| 435.4768471476266 | 3.11878035644e-18 |
| 434.4048653344918 | 2.01342081474e-18 |
| 433.3381482016467 | 1.29724398982e-18 |
| 432.2766570605187 | 8.34153792168e-19 |
| 431.22035360069 | 5.35313180085e-19 |
| 430.1691998852882 | 3.42852317719e-19 |
| 429.1231583464454 | 2.1915106583e-19 |
| 428.0821917808219 | 1.39803301533e-19 |
| 427.0462633451957 | 8.90079113349e-20 |
| 426.01533655211585 | 5.65557977978e-20 |
| 424.9893752656184 | 3.58643526241e-20 |
| 423.96834369700395 | 2.26979271343e-20 |
| 422.9522064006767 | 1.4336618766e-20 |
| 421.9409282700422 | 9.03742305913e-21 |
| 420.93447453346425 | 5.68564611541e-21 |
| 419.9328107502799 | 3.56986976033e-21 |
| 418.93590280687056 | 2.23698078325e-21 |
| 417.94371691278906 | 1.39897343574e-21 |
| 416.9562195969423 | 8.73160368262e-22 |
| 415.97337770382694 | 5.43896037233e-22 |
| 414.99515838981876 | 3.38123304752e-22 |
| 414.0215291195142 | 2.09783654146e-22 |
| 413.0524576621231 | 1.2989891295e-22 |
| 412.08791208791206 | 8.02743350321e-23 |
| 411.1278607646978 | 4.95091243563e-23 |
| 410.17227235438884 | 3.04741149781e-23 |
| 409.22111580957574 | 1.87203638641e-23 |
| 408.2743603701687 | 1.14771695293e-23 |
| 407.33197556008145 | 7.02251432679e-24 |
| 406.39393118396094 | 4.28832603777e-24 |
| 405.46019732396263 | 2.61348664656e-24 |
| 404.53074433656957 | 1.58960821606e-24 |
| 403.6055428494551 | 9.64933171998e-25 |
| 402.68456375838923 | 5.84576965381e-25 |
| 401.76777822418643 | 3.5344633599e-25 |
| 400.85515766969536 | 2.13276297403e-25 |
| 399.9466737768297 | 1.28439623548e-25 |
| 399.0422984836393 | 7.71956410247e-26 |
| 398.14200398142003 | 4.63045698224e-26 |
| 397.24576271186436 | 2.77199378113e-26 |
| 396.3535473642489 | 1.65614352415e-26 |
| 395.46533087266016 | 9.87508883107e-27 |
| 394.5810864132579 | 5.87653560781e-27 |
| 393.7007874015748 | 3.49010950525e-27 |
| 392.82440748985204 | 2.06868352276e-27 |
| 391.9519205644107 | 1.22373216951e-27 |
| 391.08330074305826 | 7.22463678888e-28 |
| 390.2185223725286 | 4.25679738408e-28 |
| 389.3575600259571 | 2.50315221154e-28 |
| 388.5003885003885 | 1.46902388757e-28 |
| 387.6469828143171 | 8.60414608536e-29 |
| 386.7973182052604 | 5.02949072436e-29 |
| 385.95137012736393 | 2.93411793419e-29 |
| 385.1091142490372 | 1.70831690442e-29 |
| 384.2705264506212 | 9.9265110139e-30 |
| 383.4355828220859 | 5.75654808722e-30 |
| 382.6042596607575 | 3.33169289374e-30 |
| 381.77653346907607 | 1.92444323588e-30 |
| 380.95238095238096 | 1.10938570555e-30 |
| 380.1317790167258 | 6.38259581196e-31 |
| 379.31470476672143 | 3.66479289689e-31 |
| 378.5011355034065 | 2.10009454989e-31 |
| 377.69104872214524 | 1.20106241099e-31 |
| 376.88442211055275 | 6.85534978836e-32 |
| 376.081233546446 | 3.90508938829e-32 |
| 375.28146109582184 | 2.22008527253e-32 |
| 374.48508301086 | 1.25963769441e-32 |
| 373.69207772795215 | 7.13278209793e-33 |
| 372.9024238657551 | 4.03097014036e-33 |
| 372.11610022326965 | 2.27351341512e-33 |
| 371.33308577794276 | 1.27974304164e-33 |
| 370.55335968379444 | 7.18927916729e-34 |
| 369.7769012695673 | 4.03074417192e-34 |
| 369.0036900369003 | 2.25539408287e-34 |
| 368.23370565852457 | 1.25949646805e-34 |
| 367.4669279764821 | 7.01954215349e-35 |
| 366.7033370003667 | 3.90443263282e-35 |
| 365.9429129055867 | 2.16742658715e-35 |
| 365.1856360316494 | 1.20079307282e-35 |
| 364.4314868804664 | 6.63940660521e-36 |
| 363.68044611468054 | 3.6637655378e-36 |
| 362.93249455601256 | 2.01773159755e-36 |
| 362.1876131836291 | 1.10901247522e-36 |
| 361.4457831325301 | 6.08340573106e-37 |
| 360.7069856919562 | 3.3303850485e-37 |
| 359.97120230381563 | 1.81961466445e-37 |
| 359.2384145611304 | 9.92205711459e-38 |
| 358.50860420650093 | 5.39959680209e-38 |
| 357.7817531305903 | 2.93263662841e-38 |
| 357.057843370626 | 1.58961697548e-38 |
| 356.33685710892024 | 8.59931867736e-39 |
| 355.6187766714082 | 4.64272445654e-39 |
| 354.9035845262037 | 2.50160703247e-39 |
| 354.1912632821723 | 1.34524870623e-39 |
| 353.48179568752204 | 7.21977047404e-40 |
| 352.77516462841015 | 3.86706536439e-40 |
| 352.07135312756714 | 2.06717359475e-40 |
| 351.3703443429374 | 1.10283286203e-40 |
| 350.6721215663355 | 5.87191502858e-41 |
| 349.9766682221185 | 3.12023382049e-41 |
| 349.2839678658749 | 1.65474787197e-41 |
| 348.59400418312805 | 8.75817938946e-42 |
| 347.90676098805517 | 4.62629321852e-42 |
| 347.2222222222222 | 2.43887638119e-42 |
| 346.54037195333257 | 1.28316863128e-42 |
| 345.8611943739912 | 6.73775157356e-43 |
| 345.1846738004832 | 3.53088495785e-43 |
| 344.5107946715664 | 1.84667068475e-43 |
| 343.8395415472779 | 9.63901499273e-44 |
| 343.17089910775564 | 5.02126532435e-44 |
| 342.50485215207215 | 2.61054388315e-44 |
| 341.84138559708293 | 1.35452223809e-44 |
| 341.1804844762879 | 7.01420725917e-45 |
| 340.522133938706 | 3.62500293811e-45 |
| 339.86631924776253 | 1.86971514024e-45 |
| 339.2130257801899 | 9.62453686081e-46 |
| 338.56223902494077 | 4.94449052392e-46 |
| 337.91394458211306 | 2.5351319641e-46 |
| 337.2681281618887 | 1.29722976986e-46 |
| 336.6247755834829 | 6.62476619627e-47 |
| 335.9838727741068 | 3.37645929085e-47 |
| 335.3454057679409 | 1.71747250654e-47 |
| 334.709360705121 | 8.71877323953e-48 |
| 334.07572383073494 | 4.41731413373e-48 |
| 333.44448149383123 | 2.2335645108e-48 |
| 332.81562014643885 | 1.12713524292e-48 |
| 332.1891263425977 | 5.67663370603e-49 |
| 331.5649867374005 | 2.85327103083e-49 |
| 330.9431880860452 | 1.43130601294e-49 |
| 330.323717242898 | 7.16571081948e-50 |
| 329.70656116056705 | 3.58033260022e-50 |
| 329.0917068889864 | 1.78535581076e-50 |
| 328.47914157451 | 8.88512420189e-51 |
| 327.86885245901635 | 4.41305700981e-51 |
| 327.26082687902255 | 2.18752429947e-51 |
| 326.6550522648083 | 1.0821900765e-51 |
| 326.05151613955 | 5.34307809159e-52 |
| 325.4502061184639 | 2.63279357387e-52 |
| 324.8511099079588 | 1.29473059311e-52 |
| 324.25421530479895 | 6.35446985787e-53 |
| 323.65951019527455 | 3.11255153669e-53 |
| 323.0669825543829 | 1.52156703741e-53 |
| 322.4766204450177 | 7.42340140406e-54 |
| 321.88841201716735 | 3.61453230998e-54 |
| 321.3023455071222 | 1.75646132189e-54 |
| 320.71840923669015 | 8.51848540462e-55 |
| 320.1365916124213 | 4.12309640545e-55 |
| 319.5568811248402 | 1.99169075974e-55 |
| 318.97926634768737 | 9.60191021336e-56 |
| 318.40373593716834 | 4.61987990178e-56 |
| 317.8302786312109 | 2.21840606595e-56 |
| 317.2588832487309 | 1.06313573309e-56 |
| 316.6895386889053 | 5.08479794435e-57 |
| 316.1222339304531 | 2.42714653817e-57 |
| 315.55695803092453 | 1.15626028315e-57 |
| 314.99370012599746 | 5.49733916162e-58 |
| 314.432449428781 | 2.6084755399e-58 |
| 313.8731952291274 | 1.23525982958e-58 |
| 313.31592689295036 | 5.83804154725e-59 |
| 312.76063386155124 | 2.7536792838e-59 |
| 312.2073056509522 | 1.29627410779e-59 |
| 311.65593185123623 | 6.09000498958e-60 |
| 311.1065021258944 | 2.85545791407e-60 |
| 310.5590062111801 | 1.33619915188e-60 |
| 310.01343391546965 | 6.24027793322e-61 |
| 309.4697751186301 | 2.9085329438e-61 |
| 308.9280197713932 | 1.35294877744e-61 |
| 308.3881578947368 | 6.28096010495e-62 |
| 307.8501795792714 | 2.91010077274e-62 |
| 307.31407498463426 | 1.34563518607e-62 |
| 306.77983433888943 | 6.20989066933e-63 |
| 306.2474479379338 | 2.86007806061e-63 |
| 305.7169061449098 | 1.31464693145e-63 |
| 305.1881993896236 | 6.03083838789e-64 |
| 304.6613181679699 | 2.76110906695e-64 |
| 304.1362530413625 | 1.2616147175e-64 |
| 303.61299463617036 | 5.75317049124e-65 |
| 303.09153364316023 | 2.61833405781e-65 |
| 302.571860816944 | 1.18926921881e-65 |
| 302.0539669754329 | 5.39104075552e-66 |
| 301.5378429992964 | 2.43894700999e-66 |
| 301.02347983142687 | 1.10120817634e-66 |
| 300.5108684764098 | 4.96219472773e-67 |
