# Supplementary material for: Data of electronic, reactivity, optoelectronic, linear and non-linear optical parameters of doping graphene oxide nanosheet with aluminum atom
Source: Data Brief. 2022 Jan 19;41:107840. doi: 10.1016/j.dib.2022.107840 (PMC8801356; doi:10.1016/j.dib.2022.107840)
Supplement: Supplementary file 1 [file mmc1.zip › supplementary file/DATA OF THE UV-VIS SPECTRA/DATA OF THE UV-VIS SPECTRA OF GON2 AND ITS DERIVATIVES (GON2-Alx) B3LYP-D3.docx]

**Data of the UV-Vis spectra of GON2 isomer and its aluminum-doped derivatives (GON2-Alx), computed at the B3LYP-D3/6-31+G(d,p) level of theory**

| **GON2** | | **GON2-Al1** | | **GON2-Al2** | |
| --- | --- | --- | --- | --- | --- |
| Wavelength (nm) | Abs | Wavelength (nm) | Abs | Wavelength (nm) | Abs |
| 2000.0 | 8.11054256596e-09 | 2000.0 | 0.0336140015517 | 2000.0 | 2.05889209233e-13 |
| 1977.5873434410018 | 1.10963035216e-08 | 1977.5873434410018 | 0.0411125571156 | 1977.5873434410018 | 3.03459823493e-13 |
| 1955.671447196871 | 1.5151096363e-08 | 1955.671447196871 | 0.0501840937223 | 1955.671447196871 | 4.46381440273e-13 |
| 1934.2359767891683 | 2.06465316404e-08 | 1934.2359767891683 | 0.0611357161638 | 1934.2359767891683 | 6.55312376587e-13 |
| 1913.265306122449 | 2.80793768e-08 | 1913.265306122449 | 0.074329505954 | 1913.265306122449 | 9.60125323733e-13 |
| 1892.7444794952683 | 3.81122980715e-08 | 1892.7444794952683 | 0.0901913345568 | 1892.7444794952683 | 1.40392785781e-12 |
| 1872.6591760299625 | 5.16273841703e-08 | 1872.6591760299625 | 0.109220888811 | 1872.6591760299625 | 2.04879719971e-12 |
| 1852.9956763434218 | 6.97963028793e-08 | 1852.9956763434218 | 0.132003038218 | 1852.9956763434218 | 2.98394260221e-12 |
| 1833.7408312958437 | 9.41720510023e-08 | 1833.7408312958437 | 0.159220681003 | 1833.7408312958437 | 4.33729809093e-12 |
| 1814.8820326678765 | 1.26808671493e-07 | 1814.8820326678765 | 0.191669212275 | 1814.8820326678765 | 6.29195184523e-12 |
| 1796.4071856287424 | 1.70417107302e-07 | 1796.4071856287424 | 0.230272762855 | 1796.4071856287424 | 9.10938006339e-12 |
| 1778.3046828689983 | 2.28567637141e-07 | 1778.3046828689983 | 0.276102361095 | 1778.3046828689983 | 1.31622327788e-11 |
| 1760.5633802816901 | 3.05952214642e-07 | 1760.5633802816901 | 0.330396171891 | 1760.5633802816901 | 1.89804989677e-11 |
| 1743.1725740848342 | 4.08723658095e-07 | 1743.1725740848342 | 0.394581966664 | 1743.1725740848342 | 2.73163705988e-11 |
| 1726.1219792865363 | 5.44933196261e-07 | 1726.1219792865363 | 0.470301974887 | 1726.1219792865363 | 3.92351827717e-11 |
| 1709.4017094017095 | 7.25093591019e-07 | 1709.4017094017095 | 0.55944026133 | 1709.4017094017095 | 5.62426372684e-11 |
| 1693.002257336343 | 9.6290220424e-07 | 1693.002257336343 | 0.66415276297 | 1693.002257336343 | 8.04624008222e-11 |
| 1676.9144773616547 | 1.27616728827e-06 | 1676.9144773616547 | 0.78690010505 | 1676.9144773616547 | 1.14883487174e-10 |
| 1661.1295681063123 | 1.68799186235e-06 | 1661.1295681063123 | 0.930483296431 | 1661.1295681063123 | 1.63704095839e-10 |
| 1645.6390565002741 | 2.228283282e-06 | 1645.6390565002741 | 1.09808237966 | 1645.6390565002741 | 2.32808479431e-10 |
| 1630.4347826086955 | 2.93567360632e-06 | 1630.4347826086955 | 1.29329808056 | 1630.4347826086955 | 3.30426869663e-10 |
| 1615.5088852988692 | 3.85995683033e-06 | 1615.5088852988692 | 1.52019646512 | 1615.5088852988692 | 4.68046746595e-10 |
| 1600.8537886872998 | 5.06517482711e-06 | 1600.8537886872998 | 1.78335656746 | 1600.8537886872998 | 6.61668437226e-10 |
| 1586.4621893178212 | 6.63351545228e-06 | 1586.4621893178212 | 2.08792090161 | 1586.4621893178212 | 9.3353137605e-10 |
| 1572.3270440251572 | 8.67022490929e-06 | 1572.3270440251572 | 2.43964871094 | 1572.3270440251572 | 1.31448227068e-09 |
| 1558.4415584415583 | 1.13097835844e-05 | 1558.4415584415583 | 2.84497174273 | 1558.4415584415583 | 1.84721675643e-09 |
| 1544.799176107106 | 1.47236518229e-05 | 1544.799176107106 | 3.31105226091 | 1544.799176107106 | 2.59070700319e-09 |
| 1531.3935681470139 | 1.91299615089e-05 | 1531.3935681470139 | 3.84584292802 | 1531.3935681470139 | 3.62623588779e-09 |
| 1518.2186234817814 | 2.48056131483e-05 | 1518.2186234817814 | 4.45814809799 | 1518.2186234817814 | 5.06560261322e-09 |
| 1505.2684395383842 | 3.21013391327e-05 | 1505.2684395383842 | 5.15768596494 | 1505.2684395383842 | 7.06225664197e-09 |
| 1492.5373134328358 | 4.14604151075e-05 | 1492.5373134328358 | 5.95515091029 | 1492.5373134328358 | 9.82637168134e-09 |
| 1480.0197335964478 | 5.34418465006e-05 | 1480.0197335964478 | 6.86227528304 | 1480.0197335964478 | 1.36452089902e-08 |
| 1467.7103718199608 | 6.87490304187e-05 | 1467.7103718199608 | 7.8918897356 | 1467.7103718199608 | 1.89105651959e-08 |
| 1455.604075691412 | 8.82650990575e-05 | 1455.604075691412 | 9.05798112355 | 1455.604075691412 | 2.61556874802e-08 |
| 1443.6958614051973 | 0.000113096394872 | 1443.6958614051973 | 10.3757468625 | 1443.6958614051973 | 3.61048115869e-08 |
| 1431.9809069212408 | 0.000144625816137 | 1431.9809069212408 | 11.8616445217 | 1431.9809069212408 | 4.97394913033e-08 |
| 1420.4545454545455 | 0.000184578111066 | 1420.4545454545455 | 13.5334353237 | 1420.4545454545455 | 6.83872135478e-08 |
| 1409.1122592766555 | 0.000235099597024 | 1409.1122592766555 | 15.4102201176 | 1409.1122592766555 | 9.38395228805e-08 |
| 1397.9496738117427 | 0.000298855247117 | 1397.9496738117427 | 17.5124662982 | 1397.9496738117427 | 1.28509130832e-07 |
| 1386.9625520110958 | 0.000379146628975 | 1386.9625520110958 | 19.8620240636 | 1386.9625520110958 | 1.75638409029e-07 |
| 1376.1467889908256 | 0.000480054810244 | 1376.1467889908256 | 22.4821303381 | 1376.1467889908256 | 2.39575451753e-07 |
| 1365.4984069185252 | 0.000606613073259 | 1365.4984069185252 | 25.3973986438 | 1365.4984069185252 | 3.26138785753e-07 |
| 1355.0135501355014 | 0.000765015119837 | 1355.0135501355014 | 28.6337931789 | 1355.0135501355014 | 4.43098109525e-07 |
| 1344.688480502017 | 0.000962865408833 | 1344.688480502017 | 32.218585371 | 1344.688480502017 | 6.00806548013e-07 |
| 1334.5195729537365 | 0.0012094793677 | 1334.5195729537365 | 36.1802912029 | 1334.5195729537365 | 8.13030298529e-07 |
| 1324.5033112582782 | 0.00151624246889 | 1324.5033112582782 | 40.5485876823 | 1324.5033112582782 | 1.0980348305e-06 |
| 1314.6362839614374 | 0.0018970385771 | 1314.6362839614374 | 45.3542069307 | 1314.6362839614374 | 1.48000374915e-06 |
| 1304.9151805132665 | 0.00236875956831 | 1304.9151805132665 | 50.628806511 | 1304.9151805132665 | 1.99088799394e-06 |
| 1295.3367875647668 | 0.00295191001145 | 1295.3367875647668 | 56.4048148034 | 1295.3367875647668 | 2.67281040071e-06 |
| 1285.8979854264894 | 0.00367132270064 | 1285.8979854264894 | 62.7152504675 | 1285.8979854264894 | 3.58118527899e-06 |
| 1276.5957446808509 | 0.00455700304459 | 1276.5957446808509 | 69.5935153044 | 1276.5957446808509 | 4.78875635744e-06 |
| 1267.427122940431 | 0.00564512276858 | 1267.427122940431 | 77.0731601527 | 1267.427122940431 | 6.39081146271e-06 |
| 1258.3892617449665 | 0.00697918607411 | 1258.3892617449665 | 85.1876238142 | 1258.3892617449665 | 8.51190136434e-06 |
| 1249.4793835901708 | 0.00861139433419 | 1249.4793835901708 | 93.9699454123 | 1249.4793835901708 | 1.13144766959e-05 |
| 1240.6947890818858 | 0.010604238582 | 1240.6947890818858 | 103.452451029 | 1240.6947890818858 | 1.50099648464e-05 |
| 1232.0328542094455 | 0.0130323524698 | 1232.0328542094455 | 113.666415946 | 1232.0328542094455 | 1.98729431855e-05 |
| 1223.4910277324632 | 0.0159846620268 | 1223.4910277324632 | 124.641704329 | 1223.4910277324632 | 2.62592319729e-05 |
| 1215.0668286755772 | 0.019566872405 | 1215.0668286755772 | 136.406388722 | 1215.0668286755772 | 3.46289370989e-05 |
| 1206.7578439259853 | 0.0239043358505 | 1206.7578439259853 | 148.986352272 | 1206.7578439259853 | 4.55757281793e-05 |
| 1198.5617259288852 | 0.0291453493314 | 1198.5617259288852 | 162.404877164 | 1198.5617259288852 | 5.98639519978e-05 |
| 1190.4761904761904 | 0.0354649345429 | 1190.4761904761904 | 176.682223304 | 1190.4761904761904 | 7.84755674383e-05 |
| 1182.4990145841543 | 0.0430691573381 | 1182.4990145841543 | 191.835201821 | 1182.4990145841543 | 0.000102669360831 |
| 1174.6280344557556 | 0.0522000479141 | 1174.6280344557556 | 207.876748505 | 1174.6280344557556 | 0.000134055477819 |
| 1166.8611435239206 | 0.0631411872274 | 1166.8611435239206 | 224.815502759 | 1166.8611435239206 | 0.000174689010394 |
| 1159.19629057187 | 0.0762240290117 | 1159.19629057187 | 242.655398108 | 1159.19629057187 | 0.000227187230394 |
| 1151.6314779270633 | 0.091835030285 | 1151.6314779270633 | 261.395270668 | 1151.6314779270633 | 0.000294876092349 |
| 1144.1647597254005 | 0.110423666227 | 1144.1647597254005 | 281.028492318 | 1144.1647597254005 | 0.000381972873015 |
| 1136.794240242516 | 0.132511407591 | 1136.794240242516 | 301.542635518 | 1136.794240242516 | 0.000493813311399 |
| 1129.5180722891566 | 0.158701740228 | 1129.5180722891566 | 322.91917688 | 1129.5180722891566 | 0.000637133407074 |
| 1122.334455667789 | 0.189691306586 | 1122.334455667789 | 345.133246626 | 1122.334455667789 | 0.000820418178383 |
| 1115.2416356877322 | 0.226282248043 | 1115.2416356877322 | 368.153430973 | 1115.2416356877322 | 0.00105433223575 |
| 1108.2379017362393 | 0.269395824306 | 1108.2379017362393 | 391.941634326 | 1108.2379017362393 | 0.00135225005719 |
| 1101.3215859030836 | 0.320087381705 | 1101.3215859030836 | 416.453007822 | 1101.3215859030836 | 0.00173090744072 |
| 1094.4910616563297 | 0.379562735626 | 1094.4910616563297 | 441.635950317 | 1094.4910616563297 | 0.00221119983959 |
| 1087.7447425670775 | 0.449196023413 | 1087.7447425670775 | 467.432187373 | 1087.7447425670775 | 0.00281915825881 |
| 1081.081081081081 | 0.530549072447 | 1081.081081081081 | 493.776933062 | 1081.081081081081 | 0.00358713921536 |
| 1074.4985673352435 | 0.625392313549 | 1074.4985673352435 | 520.599138642 | 1074.4985673352435 | 0.00455527206075 |
| 1067.995728017088 | 0.735727252046 | 1067.995728017088 | 547.821831183 | 1067.995728017088 | 0.0057732148663 |
| 1061.5711252653928 | 0.86381048759 | 1061.5711252653928 | 575.362544219 | 1061.5711252653928 | 0.0073022792246 |
| 1055.2233556102708 | 1.01217924888 | 1055.2233556102708 | 603.13384137 | 1055.2233556102708 | 0.0092179948821 |
| 1048.951048951049 | 1.18367838062 | 1048.951048951049 | 631.043932672 | 1048.951048951049 | 0.0116131972561 |
| 1042.752867570386 | 1.38148868711 | 1042.752867570386 | 658.997382083 | 1042.752867570386 | 0.0146017347839 |
| 1036.6275051831374 | 1.60915650034 | 1036.6275051831374 | 686.89590334 | 1036.6275051831374 | 0.0183229088878 |
| 1030.5736860185502 | 1.87062429901 | 1030.5736860185502 | 714.639239993 | 1030.5736860185502 | 0.0229467773162 |
| 1024.5901639344263 | 2.17026216053 | 1024.5901639344263 | 742.126124121 | 1024.5901639344263 | 0.028680471923 |
| 1018.6757215619693 | 2.51289977915 | 1018.6757215619693 | 769.255306905 | 1018.6757215619693 | 0.035775704786 |
| 1012.829169480081 | 2.90385873148 | 1012.829169480081 | 795.926652985 | 1012.829169480081 | 0.044537662114 |
| 1007.0493454179255 | 3.34898461553 | 1007.0493454179255 | 822.042289278 | 1007.0493454179255 | 0.0553355138415 |
| 1001.3351134846461 | 3.85467863211 | 1001.3351134846461 | 847.507797857 | 1001.3351134846461 | 0.068614798306 |
| 995.6853634251576 | 4.42792811832 | 995.6853634251576 | 872.233441435 | 995.6853634251576 | 0.0849119760861 |
| 990.0990099009902 | 5.07633548278 | 990.0990099009902 | 896.135409113 | 990.0990099009902 | 0.104871485028 |
| 984.5749917952082 | 5.80814493305 | 984.5749917952082 | 919.137069318 | 984.5749917952082 | 0.129265669757 |
| 979.1122715404699 | 6.63226632673 | 979.1122715404699 | 941.170216216 | 979.1122715404699 | 0.159018003512 |
| 973.7098344693281 | 7.55829542307 | 973.7098344693281 | 962.176295498 | 973.7098344693281 | 0.195230067909 |
| 968.3666881859263 | 8.59652975971 | 968.3666881859263 | 982.107595145 | 968.3666881859263 | 0.239212806934 |
| 963.0818619582664 | 9.75797933455 | 963.0818619582664 | 1000.92838672 | 963.0818619582664 | 0.292522624924 |
| 957.8544061302682 | 11.054371234 | 957.8544061302682 | 1018.61600286 | 957.8544061302682 | 0.357002953935 |
| 952.6833915528738 | 12.4981473205 | 952.6833915528738 | 1035.16183682 | 952.6833915528738 | 0.434831973222 |
| 947.5679090334806 | 14.1024540753 | 947.5679090334806 | 1050.57225062 | 947.5679090334806 | 0.528577221791 |
| 942.5070688030161 | 15.8811236862 | 942.5070688030161 | 1064.86937865 | 942.5070688030161 | 0.641257903159 |
| 937.4999999999999 | 17.8486454828 | 937.4999999999999 | 1078.09181468 | 937.4999999999999 | 0.776415738412 |
| 932.5458501709667 | 20.0201268456 | 932.5458501709667 | 1090.29517097 | 932.5458501709667 | 0.938195278044 |
| 927.643784786642 | 22.4112427647 | 927.643784786642 | 1101.5524993 | 927.643784786642 | 1.13143463323 |
| 922.7929867733004 | 25.0381732843 | 922.7929867733004 | 1111.9545649 | 922.7929867733004 | 1.36176763115 |
| 917.9926560587514 | 27.917528159 | 917.9926560587514 | 1121.60996553 | 917.9926560587514 | 1.63573843493 |
| 913.2420091324201 | 31.0662581535 | 913.2420091324201 | 1130.64508932 | 913.2420091324201 | 1.96092969347 |
| 908.5402786190186 | 34.5015525506 | 908.5402786190186 | 1139.20390624 | 908.5402786190186 | 2.34610529861 |
| 903.8867128653209 | 38.2407225836 | 903.8867128653209 | 1147.44758978 | 903.8867128653209 | 2.80136882168 |
| 899.2805755395683 | 42.3010706876 | 899.2805755395683 | 1155.55396646 | 899.2805755395683 | 3.33833867782 |
| 894.7211452430658 | 46.6997456616 | 894.7211452430658 | 1163.71679267 | 894.7211452430658 | 3.97034101856 |
| 890.2077151335311 | 51.4535840553 | 890.2077151335311 | 1172.14485938 | 890.2077151335311 | 4.71262127932 |
| 885.7395925597874 | 56.578938328 | 885.7395925597874 | 1181.06092688 | 885.7395925597874 | 5.58257520407 |
| 881.316098707403 | 62.0914925895 | 881.316098707403 | 1190.70049309 | 881.316098707403 | 6.60000003071 |
| 876.9365682548962 | 68.0060669946 | 876.9365682548962 | 1201.31040007 | 876.9365682548962 | 7.78736634381 |
| 872.6003490401396 | 74.3364121482 | 872.6003490401396 | 1213.14728497 | 872.6003490401396 | 9.17011088312 |
| 868.3068017366135 | 81.0949951601 | 868.3068017366135 | 1226.47588253 | 868.3068017366135 | 10.7769503325 |
| 864.0552995391705 | 88.2927792766 | 864.0552995391705 | 1241.56718756 | 864.0552995391705 | 12.6402158015 |
| 859.8452278589854 | 95.9389993002 | 859.8452278589854 | 1258.69648708 | 859.8452278589854 | 14.796207349 |
| 855.6759840273816 | 104.040935282 | 855.6759840273816 | 1278.14127259 | 855.6759840273816 | 17.2855674816 |
| 851.5469770082316 | 112.60368723 | 851.5469770082316 | 1300.17904408 | 851.5469770082316 | 20.1536720882 |
| 847.457627118644 | 121.629953823 | 847.457627118644 | 1325.0850186 | 847.457627118644 | 23.4510367466 |
| 843.4073657576608 | 131.119818314 | 843.4073657576608 | 1353.12975667 | 843.4073657576608 | 27.2337357588 |
| 839.3956351426972 | 141.070545009 | 839.3956351426972 | 1384.57672144 | 839.3956351426972 | 31.5638306373 |
| 835.421888053467 | 151.476389822 | 835.421888053467 | 1419.67978581 | 835.421888053467 | 36.509804086 |
| 831.4855875831485 | 162.328428523 | 831.4855875831485 | 1458.68070399 | 831.4855875831485 | 42.1469947945 |
| 827.5862068965516 | 173.61440633 | 827.5862068965516 | 1501.80656486 | 827.5862068965516 | 48.5580276034 |
| 823.7232289950576 | 185.318612481 | 823.7232289950576 | 1549.26724537 | 823.7232289950576 | 55.8332328082 |
| 819.8961464881114 | 197.421783368 | 819.8961464881114 | 1601.252883 | 819.8961464881114 | 64.0710475667 |
| 816.1044613710554 | 209.901037692 | 816.1044613710554 | 1657.93138732 | 816.1044613710554 | 73.3783915606 |
| 812.3476848090983 | 222.729846862 | 812.3476848090983 | 1719.4460114 | 812.3476848090983 | 83.8710082648 |
| 808.6253369272237 | 235.878043689 | 808.6253369272237 | 1785.91300471 | 808.6253369272237 | 95.6737624041 |
| 804.9369466058491 | 249.311872019 | 804.9369466058491 | 1857.41936968 | 804.9369466058491 | 108.920883451 |
| 801.2820512820513 | 262.994079645 | 801.2820512820513 | 1934.02074473 | 801.2820512820513 | 123.756144361 |
| 797.6601967561818 | 276.884056353 | 797.6601967561818 | 2015.73943722 | 797.6601967561818 | 140.332964162 |
| 794.0709370037056 | 290.938018503 | 794.0709370037056 | 2102.56262971 | 794.0709370037056 | 158.81442258 |
| 790.5138339920949 | 305.109240999 | 790.5138339920949 | 2194.44078339 | 790.5138339920949 | 179.373174523 |
| 786.9884575026232 | 319.348336912 | 786.9884575026232 | 2291.28626218 | 786.9884575026232 | 202.191252129 |
| 783.4943849569078 | 333.603584448 | 783.4943849569078 | 2392.97220068 | 783.4943849569078 | 227.459742084 |
| 780.0312012480499 | 347.821300295 | 780.0312012480499 | 2499.33163847 | 780.0312012480499 | 255.378326156 |
| 776.598498576236 | 361.946257758 | 776.598498576236 | 2610.15694239 | 776.598498576236 | 286.154673375 |
| 773.1958762886597 | 375.922147448 | 773.1958762886597 | 2725.19953671 | 773.1958762886597 | 320.003673012 |
| 769.8229407236336 | 389.692077645 | 769.8229407236336 | 2844.16995986 | 769.8229407236336 | 357.146498497 |
| 766.4793050587633 | 403.199110881 | 766.4793050587633 | 2966.73826383 | 766.4793050587633 | 397.80949372 |
| 763.1645891630628 | 416.386832658 | 763.1645891630628 | 3092.5347701 | 763.1645891630628 | 442.222874749 |
| 759.8784194528876 | 429.199947745 | 759.8784194528876 | 3221.15119298 | 759.8784194528876 | 490.619241861 |
| 756.6204287515762 | 441.584898959 | 756.6204287515762 | 3352.14213788 | 756.6204287515762 | 543.23189902 |
| 753.390256152687 | 453.490502971 | 753.390256152687 | 3485.02697835 | 753.390256152687 | 600.292980396 |
| 750.1875468867216 | 464.86859731 | 750.1875468867216 | 3619.29211192 | 750.1875468867216 | 662.031386303 |
| 747.011952191235 | 475.674692474 | 747.011952191235 | 3754.39359 | 747.011952191235 | 728.670534004 |
| 743.86312918423 | 485.868622901 | 743.86312918423 | 3889.7601131 | 743.86312918423 | 800.425932057 |
| 740.7407407407408 | 495.415190455 | 740.7407407407408 | 4024.79637734 | 740.7407407407408 | 877.502590407 |
| 737.6444553725104 | 504.284794081 | 737.6444553725104 | 4158.88675368 | 737.6444553725104 | 960.092282021 |
| 734.5739471106758 | 512.454039405 | 734.5739471106758 | 4291.39927616 | 734.5739471106758 | 1048.37067561 |
| 731.528895391368 | 519.906322209 | 731.528895391368 | 4421.68991058 | 731.528895391368 | 1142.49436274 |
| 728.5089849441475 | 526.632380021 | 728.5089849441475 | 4549.1070704 | 728.5089849441475 | 1242.59780648 |
| 725.5139056831922 | 532.630806383 | 725.5139056831922 | 4672.99634181 | 725.5139056831922 | 1348.79024214 |
| 722.543352601156 | 537.908522812 | 722.543352601156 | 4792.7053759 | 722.543352601156 | 1461.15256452 |
| 719.5970256656271 | 542.481203936 | 719.5970256656271 | 4907.58890182 | 719.5970256656271 | 1579.73423895 |
| 716.6746297181079 | 546.373651837 | 716.6746297181079 | 5017.01381171 | 716.6746297181079 | 1704.55027646 |
| 713.7758743754462 | 549.620116229 | 713.7758743754462 | 5120.36426502 | 713.7758743754462 | 1835.578316 |
| 710.9004739336492 | 552.264557662 | 710.9004739336492 | 5217.04675812 | 710.9004739336492 | 1972.75585832 |
| 708.0481472740146 | 554.360851615 | 708.0481472740146 | 5306.49510337 | 708.0481472740146 | 2115.97769781 |
| 705.2186177715091 | 555.972931899 | 705.2186177715091 | 5388.17526133 | 705.2186177715091 | 2265.09359911 |
| 702.4116132053383 | 557.174872441 | 702.4116132053383 | 5461.58997007 | 702.4116132053383 | 2419.90626555 |
| 699.6268656716418 | 558.050907037 | 699.6268656716418 | 5526.28311636 | 699.6268656716418 | 2580.16964555 |
| 696.8641114982578 | 558.695387225 | 696.8641114982578 | 5581.84379568 | 696.8641114982578 | 2745.58762161 |
| 694.1230911614992 | 559.212678858 | 694.1230911614992 | 5627.91001055 | 694.1230911614992 | 2915.81312435 |
| 691.4035492048858 | 559.716998369 | 691.4035492048858 | 5664.17196037 | 691.4035492048858 | 3090.44771036 |
| 688.7052341597796 | 560.332190049 | 688.7052341597796 | 5690.3748804 | 688.7052341597796 | 3269.0416391 |
| 686.027898467871 | 561.19144586 | 686.027898467871 | 5706.32139254 | 686.027898467871 | 3451.09447856 |
| 683.371298405467 | 562.436969496 | 683.371298405467 | 5711.87333645 | 683.371298405467 | 3636.05626426 |
| 680.7351940095303 | 564.219586413 | 680.7351940095303 | 5706.95305586 | 680.7351940095303 | 3823.32922915 |
| 678.1193490054249 | 566.698301556 | 678.1193490054249 | 5691.54412183 | 678.1193490054249 | 4012.27011542 |
| 675.5235307363206 | 570.039806369 | 675.5235307363206 | 5665.69148191 | 675.5235307363206 | 4202.19307088 |
| 672.9475100942127 | 574.417936463 | 672.9475100942127 | 5629.50103164 | 672.9475100942127 | 4392.37312518 |
| 670.3910614525139 | 580.013081077 | 670.3910614525139 | 5583.13861231 | 670.3910614525139 | 4582.05023194 |
| 667.8539626001781 | 587.0115451 | 667.8539626001781 | 5526.82844658 | 667.8539626001781 | 4770.43385469 |
| 665.335994677312 | 595.604864059 | 665.335994677312 | 5460.851031 | 665.335994677312 | 4956.70806552 |
| 662.8369421122403 | 605.989072084 | 662.8369421122403 | 5385.5405114 | 662.8369421122403 | 5140.0371169 |
| 660.3565925599823 | 618.363922403 | 660.3565925599823 | 5301.28157408 | 660.3565925599823 | 5319.57143864 |
| 657.8947368421053 | 632.932059567 | 657.8947368421053 | 5208.50589187 | 657.8947368421053 | 5494.45400409 |
| 655.4511688879178 | 649.89814217 | 655.4511688879178 | 5107.68816977 | 655.4511688879178 | 5663.82700257 |
| 653.0256856769699 | 669.467914551 | 653.0256856769699 | 4999.34183958 | 653.0256856769699 | 5826.83874804 |
| 650.6180871828237 | 691.847225678 | 650.6180871828237 | 4884.0144573 | 650.6180871828237 | 5982.65074886 |
| 648.2281763180639 | 717.240993284 | 648.2281763180639 | 4762.28286005 | 648.2281763180639 | 6130.44485849 |
| 645.8557588805166 | 745.852111282 | 645.8557588805166 | 4634.7481417 | 645.8557588805166 | 6269.43042363 |
| 643.5006435006435 | 777.880298578 | 643.5006435006435 | 4502.03050768 | 643.5006435006435 | 6398.85134414 |
| 641.1626415900834 | 813.520887669 | 641.1626415900834 | 4364.76407004 | 641.1626415900834 | 6517.99295801 |
| 638.8415672913118 | 852.963551837 | 638.8415672913118 | 4223.59164305 | 638.8415672913118 | 6626.18866528 |
| 636.5372374283895 | 896.390970353 | 636.5372374283895 | 4079.15959842 | 636.5372374283895 | 6722.82620639 |
| 634.2494714587738 | 943.977431896 | 634.2494714587738 | 3932.11283694 | 634.2494714587738 | 6807.35351415 |
| 631.9780914261638 | 995.8873774 | 631.9780914261638 | 3783.08993003 | 631.9780914261638 | 6879.28406241 |
| 629.7229219143577 | 1052.2738847 | 629.7229219143577 | 3632.71848118 | 629.7229219143577 | 6938.20164119 |
| 627.4837900020916 | 1113.27709871 | 627.4837900020916 | 3481.61075241 | 627.4837900020916 | 6983.76449448 |
| 625.2605252188412 | 1179.02261248 | 625.2605252188412 | 3330.35959638 | 625.2605252188412 | 7015.70876548 |
| 623.0529595015576 | 1249.61980595 | 623.0529595015576 | 3179.53472863 | 623.0529595015576 | 7033.85120315 |
| 620.8609271523178 | 1325.16015148 | 620.8609271523178 | 3029.67936937 | 620.8609271523178 | 7038.09109382 |
| 618.6842647968654 | 1405.71549651 | 618.6842647968654 | 2881.30727754 | 618.6842647968654 | 7028.4113923 |
| 616.5228113440197 | 1491.33633651 | 616.5228113440197 | 2734.90019427 | 616.5228113440197 | 7004.87903793 |
| 614.3764079459348 | 1582.05009265 | 614.3764079459348 | 2590.90570633 | 614.3764079459348 | 6967.64445246 |
| 612.2448979591836 | 1677.85941109 | 612.2448979591836 | 2449.73553431 | 612.2448979591836 | 6916.94022758 |
| 610.1281269066504 | 1778.74050283 | 610.1281269066504 | 2311.76424448 | 610.1281269066504 | 6853.07902172 |
| 608.0259424402108 | 1884.64154436 | 608.0259424402108 | 2177.32837751 | 608.0259424402108 | 6776.45069594 |
| 605.9381943041809 | 1995.48116179 | 605.9381943041809 | 2046.72598229 | 605.9381943041809 | 6687.51872946 |
| 603.864734299517 | 2111.14702199 | 603.864734299517 | 1920.21653829 | 603.864734299517 | 6586.81596455 |
| 601.8054162487462 | 2231.49455588 | 601.8054162487462 | 1798.02124553 | 601.8054162487462 | 6474.93973949 |
| 599.7600959616153 | 2356.34583962 | 599.7600959616153 | 1680.3236578 | 599.7600959616153 | 6352.54647546 |
| 597.7286312014345 | 2485.48866025 | 597.7286312014345 | 1567.27063147 | 597.7286312014345 | 6220.34579011 |
| 595.7108816521048 | 2618.67579211 | 595.7108816521048 | 1458.97355976 | 595.7108816521048 | 6079.09421518 |
| 593.7067088858104 | 2755.62451037 | 593.7067088858104 | 1355.50986056 | 593.7067088858104 | 5929.58859983 |
| 591.7159763313609 | 2896.01636676 | 591.7159763313609 | 1256.92468449 | 591.7159763313609 | 5772.65928333 |
| 589.7385492431688 | 3039.49725161 | 589.7385492431688 | 1163.23280905 | 589.7385492431688 | 5609.16312205 |
| 587.7742946708463 | 3185.6777641 | 587.7742946708463 | 1074.420685 | 587.7742946708463 | 5439.9764552 |
| 585.8230814294083 | 3334.13391036 | 585.8230814294083 | 990.448600863 | 585.8230814294083 | 5265.98809199 |
| 583.8847800700661 | 3484.40814612 | 583.8847800700661 | 911.252932901 | 583.8847800700661 | 5088.09239992 |
| 581.9592628516003 | 3636.01077684 | 581.9592628516003 | 836.748448745 | 581.9592628516003 | 4907.18256978 |
| 580.046403712297 | 3788.42172472 | 580.046403712297 | 766.830634807 | 580.046403712297 | 4724.14412748 |
| 578.1460782424359 | 3941.09266712 | 578.1460782424359 | 701.378019461 | 578.1460782424359 | 4539.84875668 |
| 576.2581636573184 | 4093.44954641 | 576.2581636573184 | 640.254466305 | 576.2581636573184 | 4355.14848922 |
| 574.3825387708214 | 4244.89544594 | 574.3825387708214 | 583.31141422 | 574.3825387708214 | 4170.8703126 |
| 572.5190839694656 | 4394.81382148 | 572.5190839694656 | 530.390043619 | 572.5190839694656 | 3987.81123549 |
| 570.6676811869887 | 4542.57207202 | 570.6676811869887 | 481.323350927 | 570.6676811869887 | 3806.733844 |
| 568.8282138794084 | 4687.52542789 | 568.8282138794084 | 435.938116116 | 568.8282138794084 | 3628.36237258 |
| 567.000567000567 | 4829.02112886 | 567.000567000567 | 394.056750821 | 567.000567000567 | 3453.37930513 |
| 565.1846269781461 | 4966.40285922 | 565.1846269781461 | 355.499017264 | 565.1846269781461 | 3282.4225131 |
| 563.3802816901408 | 5099.01540167 | 563.3802816901408 | 320.083610774 | 563.3802816901408 | 3116.08292965 |
| 561.5874204417821 | 5226.20946692 | 561.5874204417821 | 287.629601151 | 561.5874204417821 | 2954.90275115 |
| 559.8059339428997 | 5347.34665152 | 559.8059339428997 | 257.957730403 | 559.8059339428997 | 2799.37415043 |
| 558.0357142857143 | 5461.80447267 | 558.0357142857143 | 230.89156651 | 558.0357142857143 | 2649.93847963 |
| 556.2766549230483 | 5568.98142534 | 556.2766549230483 | 206.25851474 | 556.2766549230483 | 2506.98593537 |
| 554.52865064695 | 5668.30200474 | 554.52865064695 | 183.89068978 | 554.52865064695 | 2370.8556539 |
| 552.791597567717 | 5759.22163567 | 552.791597567717 | 163.625653387 | 552.791597567717 | 2241.83620041 |
| 551.0653930933137 | 5841.23144913 | 551.0653930933137 | 145.307023516 | 551.0653930933137 | 2120.16641351 |
| 549.3499359091741 | 5913.8628472 | 549.3499359091741 | 128.784961948 | 549.3499359091741 | 2006.03656443 |
| 547.645125958379 | 5976.69179797 | 547.645125958379 | 113.916548207 | 547.645125958379 | 1899.58978873 |
| 545.950864422202 | 6029.34280455 | 545.950864422202 | 100.566048212 | 545.950864422202 | 1800.92374901 |
| 544.2670537010159 | 6071.49249519 | 544.2670537010159 | 88.6050865203 | 544.2670537010159 | 1710.09248688 |
| 542.5935973955508 | 6102.8727854 | 542.5935973955508 | 77.9127312432 | 542.5935973955508 | 1627.10842465 |
| 540.9304002884962 | 6123.27356793 | 540.9304002884962 | 68.3755008185 | 540.9304002884962 | 1551.9444787 |
| 539.2773683264425 | 6132.54489184 | 539.2773683264425 | 59.8873017564 | 539.2773683264425 | 1484.53624943 |
| 537.6344086021505 | 6130.59859838 | 537.6344086021505 | 52.349306276 | 537.6344086021505 | 1424.78425589 |
| 536.0014293371448 | 6117.40938838 | 536.0014293371448 | 45.6697784606 | 536.0014293371448 | 1372.55618666 |
| 534.3783398646241 | 6093.015303 | 534.3783398646241 | 39.7638571644 | 534.3783398646241 | 1327.68914221 |
| 532.7650506126798 | 6057.51760785 | 532.7650506126798 | 34.5533034442 | 532.7650506126798 | 1289.99184855 |
| 531.1614730878186 | 6011.08007813 | 531.1614730878186 | 29.9662197673 | 531.1614730878186 | 1259.24682529 |
| 529.5675198587819 | 5953.92769102 | 529.5675198587819 | 25.9367476885 | 529.5675198587819 | 1235.21249609 |
| 527.9831045406547 | 5886.34473905 | 527.9831045406547 | 22.4047501012 | 527.9831045406547 | 1217.62523276 |
| 526.4081417792595 | 5808.67238664 | 526.4081417792595 | 19.3154835687 | 526.4081417792595 | 1206.20132845 |
| 524.8425472358292 | 5721.30569911 | 524.8425472358292 | 16.6192656385 | 524.8425472358292 | 1200.6388987 |
| 523.2862375719518 | 5624.69018067 | 523.2862375719518 | 14.2711414512 | 523.2862375719518 | 1200.61971204 |
| 521.7391304347826 | 5519.31786451 | 521.7391304347826 | 12.2305533762 | 521.7391304347826 | 1205.81095482 |
| 520.2011444425177 | 5405.72300355 | 520.2011444425177 | 10.4610168582 | 520.2011444425177 | 1215.86693686 |
| 518.6721991701245 | 5284.47741576 | 518.6721991701245 | 8.92980513324 | 518.6721991701245 | 1230.43074661 |
| 517.1522151353215 | 5156.18554166 | 517.1522151353215 | 7.60764498641 | 517.1522151353215 | 1249.13586532 |
| 515.6411137848057 | 5021.47927513 | 515.6411137848057 | 6.46842527384 | 515.6411137848057 | 1271.60775085 |
| 514.1388174807198 | 4881.01263036 | 514.1388174807198 | 5.48891951974 | 514.1388174807198 | 1297.46540169 |
| 512.6452494873547 | 4735.45630928 | 512.6452494873547 | 4.6485235299 | 512.6452494873547 | 1326.32291163 |
| 511.1603339580848 | 4585.49223379 | 511.1603339580848 | 3.92900863334 | 511.1603339580848 | 1357.79102467 |
| 509.683995922528 | 4431.80810599 | 509.683995922528 | 3.31429087461 | 509.683995922528 | 1391.47869883 |
| 508.2161612739285 | 4275.09205822 | 508.2161612739285 | 2.79021622853 | 508.2161612739285 | 1426.9946857 |
| 506.7567567567567 | 4116.02745149 | 506.7567567567567 | 2.34436169596 | 506.7567567567567 | 1463.94913098 |
| 505.3057099545225 | 3955.28787759 | 505.3057099545225 | 1.96585196013 | 505.3057099545225 | 1501.9551991 |
| 503.8629492777964 | 3793.53241571 | 503.8629492777964 | 1.64519113706 | 503.8629492777964 | 1540.63072261 |
| 502.4284039524367 | 3631.40118934 | 502.4284039524367 | 1.37410903703 | 502.4284039524367 | 1579.59987492 |
| 501.00200400801606 | 3469.5112638 | 501.00200400801606 | 1.14542126412 | 501.00200400801606 | 1618.49486211 |
| 499.5836802664446 | 3308.45291865 | 499.5836802664446 | 0.952902415589 | 499.5836802664446 | 1656.95762755 |
| 498.1733643307871 | 3148.78632301 | 498.1733643307871 | 0.791171598181 | 498.1733643307871 | 1694.64156034 |
| 496.7709885742673 | 2991.03863526 | 496.7709885742673 | 0.655589452919 | 496.7709885742673 | 1731.21319671 |
| 495.3764861294584 | 2835.70154211 | 495.3764861294584 | 0.542165869733 | 495.3764861294584 | 1766.35390131 |
| 493.98979087765514 | 2683.22924551 | 493.98979087765514 | 0.447477576903 | 493.98979087765514 | 1799.76151367 |
| 492.61083743842363 | 2534.0368994 | 492.61083743842363 | 0.368594804784 | 492.61083743842363 | 1831.15194384 |
| 491.2395611593253 | 2388.49949237 | 491.2395611593253 | 0.303016246844 | 491.2395611593253 | 1860.26069994 |
| 489.8758981058131 | 2246.95116646 | 489.8758981058131 | 0.248611571654 | 489.8758981058131 | 1886.84432983 |
| 488.5197850512946 | 2109.68495702 | 488.5197850512946 | 0.203570775436 | 488.5197850512946 | 1910.68175887 |
| 487.17115946735953 | 1976.95293377 | 487.17115946735953 | 0.166359704591 | 487.17115946735953 | 1931.57550556 |
| 485.82995951416996 | 1848.966719 | 485.82995951416996 | 0.135681119949 | 485.82995951416996 | 1949.35275785 |
| 484.49612403100775 | 1725.89835509 | 484.49612403100775 | 0.11044071822 | 484.49612403100775 | 1963.8662934 |
| 483.16959252697694 | 1607.88149071 | 483.16959252697694 | 0.0897175702989 | 483.16959252697694 | 1974.99522872 |
| 481.8503051718599 | 1495.01285242 | 481.8503051718599 | 0.072738479901 | 481.8503051718599 | 1982.64558347 |
| 480.5382027871216 | 1387.35396708 | 480.5382027871216 | 0.0588558088519 | 480.5382027871216 | 1986.75064849 |
| 479.23322683706067 | 1284.93309891 | 479.23322683706067 | 0.0475283567151 | 479.23322683706067 | 1987.27114824 |
| 477.9353194201051 | 1187.74736508 | 477.9353194201051 | 0.0383049219255 | 477.9353194201051 | 1984.19519069 |
| 476.64442326024783 | 1095.76499347 | 476.64442326024783 | 0.0308102089289 | 476.64442326024783 | 1977.53800065 |
| 475.3604816986214 | 1008.92768702 | 475.3604816986214 | 0.0247327808227 | 475.3604816986214 | 1967.3414352 |
| 474.08343868520853 | 927.153060296 | 474.08343868520853 | 0.0198147895338 | 474.08343868520853 | 1953.67328259 |
| 472.8132387706856 | 850.337115468 | 472.8132387706856 | 0.0158432456117 | 472.8132387706856 | 1936.6263491 |
| 471.5498270983967 | 778.356726765 | 471.5498270983967 | 0.0126426172671 | 471.5498270983967 | 1916.31734109 |
| 470.29314939645707 | 711.072104888 | 470.29314939645707 | 0.0100685733935 | 470.29314939645707 | 1892.88555206 |
| 469.04315196998124 | 648.329215311 | 469.04315196998124 | 0.0080027080578 | 469.04315196998124 | 1866.49136737 |
| 467.7997816934352 | 589.962127161 | 467.7997816934352 | 0.00634810444512 | 467.7997816934352 | 1837.31460132 |
| 466.5629860031104 | 535.795272206 | 466.5629860031104 | 0.00502561461066 | 466.5629860031104 | 1805.5526839 |
| 465.33271288971605 | 485.645596388 | 465.33271288971605 | 0.00397074778025 | 465.33271288971605 | 1771.4187157 |
| 464.10891089108907 | 439.324589297 | 464.10891089108907 | 0.00313107448072 | 464.10891089108907 | 1735.13941179 |
| 462.8915290850177 | 396.640179854 | 462.8915290850177 | 0.00246406663124 | 462.8915290850177 | 1696.95295596 |
| 461.68051708217905 | 357.398489283 | 461.68051708217905 | 0.00193530502819 | 461.68051708217905 | 1657.10678798 |
| 460.47582501918646 | 321.405435152 | 460.47582501918646 | 0.00151699555524 | 460.47582501918646 | 1615.85534692 |
| 459.2774035517452 | 288.468182788 | 459.2774035517452 | 0.00118674408463 | 459.2774035517452 | 1573.45779389 |
| 458.0852038479157 | 258.396442697 | 458.0852038479157 | 0.000926547536868 | 458.0852038479157 | 1530.17573714 |
| 456.89917758148033 | 231.003614803 | 456.89917758148033 | 0.00072196505769 | 456.89917758148033 | 1486.27098242 |
| 455.7192769254139 | 206.107782179 | 455.7192769254139 | 0.0005614388672 | 455.7192769254139 | 1442.00333017 |
| 454.54545454545456 | 183.532558674 | 454.54545454545456 | 0.000435739142833 | 454.54545454545456 | 1397.62844071 |
| 453.3776635937736 | 163.107796254 | 453.3776635937736 | 0.000337511411318 | 453.3776635937736 | 1353.39578656 |
| 452.2158577027434 | 144.670159104 | 452.2158577027434 | 0.000260908432644 | 452.2158577027434 | 1309.5467102 |
| 451.05999097880016 | 128.063572495 | 451.05999097880016 | 0.000201291540033 | 451.05999097880016 | 1266.3126033 |
| 449.9100179964007 | 113.139555187 | 449.9100179964007 | 0.000154988924588 | 449.9100179964007 | 1223.91322194 |
| 448.7658937920718 | 99.7574446715 | 448.7658937920718 | 0.000119100484224 | 448.7658937920718 | 1182.5551503 |
| 447.6275738585497 | 87.7845248943 | 447.6275738585497 | 9.13406493e-05 | 447.6275738585497 | 1142.43042317 |
| 446.49501413900873 | 77.0960662738 | 446.49501413900873 | 6.9912100766e-05 | 446.49501413900873 | 1103.71531564 |
| 445.36817102137763 | 67.5752878063 | 445.36817102137763 | 5.34045532874e-05 | 445.36817102137763 | 1066.56930636 |
| 444.247001332741 | 59.1132509194 | 444.247001332741 | 4.07138229954e-05 | 444.247001332741 | 1031.13421849 |
| 443.13146233382565 | 51.608694453 | 443.13146233382565 | 3.0977269345e-05 | 443.13146233382565 | 997.533540682 |
| 442.02151171357 | 44.9678197711 | 442.02151171357 | 2.35224209343e-05 | 442.02151171357 | 965.871928468 |
| 440.9171075837742 | 39.1040345428 | 440.9171075837742 | 1.78261898989e-05 | 440.9171075837742 | 936.234884617 |
| 439.8182084738308 | 33.9376631972 | 439.8182084738308 | 1.34825690796e-05 | 439.8182084738308 | 908.688615395 |
| 438.72477332553376 | 29.3956314737 | 438.72477332553376 | 1.01771079853e-05 | 438.72477332553376 | 883.280058076 |
| 437.636761487965 | 25.4111318706 | 437.636761487965 | 7.66679239847e-06 | 437.636761487965 | 860.037073701 |
| 436.5541327124563 | 21.923276154 | 436.5541327124563 | 5.76422077768e-06 | 436.5541327124563 | 838.968797779 |
| 435.4768471476266 | 18.8767404472 | 435.4768471476266 | 4.32518891844e-06 | 435.4768471476266 | 820.066140568 |
| 434.4048653344918 | 16.2214077765 | 434.4048653344918 | 3.23897144476e-06 | 434.4048653344918 | 803.30242758 |
| 433.3381482016467 | 13.9120123239 | 433.3381482016467 | 2.42073199402e-06 | 433.3381482016467 | 788.634170147 |
| 432.2766570605187 | 11.9077890349 | 432.2766570605187 | 1.8056095601e-06 | 432.2766570605187 | 776.001955221 |
| 431.22035360069 | 10.1721316541 | 431.22035360069 | 1.34412146546e-06 | 431.22035360069 | 765.331443003 |
| 430.1691998852882 | 8.67226172484 | 430.1691998852882 | 9.98598054681e-07 | 430.1691998852882 | 756.534460584 |
| 429.1231583464454 | 7.37891058747 | 429.1231583464454 | 7.404239094e-07 | 429.1231583464454 | 749.510179461 |
| 428.0821917808219 | 6.26601595491 | 428.0821917808219 | 5.4790802957e-07 | 428.0821917808219 | 744.14636456 |
| 427.0462633451957 | 5.3104342281 | 427.0462633451957 | 4.04643343483e-07 | 427.0462633451957 | 740.320682284 |
| 426.01533655211585 | 4.49166934393 | 426.01533655211585 | 2.98246004024e-07 | 426.01533655211585 | 737.902055071 |
| 424.9893752656184 | 3.79161862013 | 424.9893752656184 | 2.19388751927e-07 | 424.9893752656184 | 736.752049965 |
| 423.96834369700395 | 3.19433577671 | 423.96834369700395 | 1.61061435552e-07 | 423.96834369700395 | 736.72628883 |
| 422.9522064006767 | 2.68581106853 | 422.9522064006767 | 1.18006587524e-07 | 422.9522064006767 | 737.675868017 |
| 421.9409282700422 | 2.25376825737 | 421.9409282700422 | 8.62895908348e-08 | 421.9409282700422 | 739.448775514 |
| 420.93447453346425 | 1.88747798144 | 420.93447453346425 | 6.29720792796e-08 | 420.93447453346425 | 741.891293939 |
| 419.9328107502799 | 1.5775869428 | 419.9328107502799 | 4.58643367723e-08 | 419.9328107502799 | 744.849378066 |
| 418.93590280687056 | 1.31596222585 | 418.93590280687056 | 3.3338008789e-08 | 418.93590280687056 | 748.169996033 |
| 417.94371691278906 | 1.09554997968 | 417.94371691278906 | 2.4184752394e-08 | 417.94371691278906 | 751.702423825 |
| 416.9562195969423 | 0.910247641037 | 416.9562195969423 | 1.75097956896e-08 | 416.9562195969423 | 755.299483187 |
| 415.97337770382694 | 0.754788839186 | 415.97337770382694 | 1.26519641176e-08 | 415.97337770382694 | 758.81871373 |
| 414.99515838981876 | 0.62464010704 | 414.99515838981876 | 9.1237265301e-09 | 414.99515838981876 | 762.123470644 |
| 414.0215291195142 | 0.515908521239 | 414.0215291195142 | 6.56634954842e-09 | 414.0215291195142 | 765.083940169 |
| 413.0524576621231 | 0.425259405064 | 413.0524576621231 | 4.71642749588e-09 | 413.0524576621231 | 767.57806576 |
| 412.08791208791206 | 0.349843249843 | 412.08791208791206 | 3.38095779288e-09 | 412.08791208791206 | 769.492378753 |
| 411.1278607646978 | 0.287231040618 | 411.1278607646978 | 2.4188211296e-09 | 411.1278607646978 | 770.722728207 |
| 410.17227235438884 | 0.235357208383 | 410.17227235438884 | 1.72705103916e-09 | 410.17227235438884 | 771.174905601 |
| 409.22111580957574 | 0.192469472523 | 409.22111580957574 | 1.23067676525e-09 | 409.22111580957574 | 770.765161047 |
| 408.2743603701687 | 0.157084881584 | 408.2743603701687 | 8.75226053333e-10 | 408.2743603701687 | 769.420608734 |
| 407.33197556008145 | 0.127951407023 | 407.33197556008145 | 6.21203474372e-10 | 407.33197556008145 | 767.079520401 |
| 406.39393118396094 | 0.104014491876 | 406.39393118396094 | 4.4003266325e-10 | 406.39393118396094 | 763.691506729 |
| 405.46019732396263 | 0.0843880036012 | 405.46019732396263 | 3.11080902787e-10 | 405.46019732396263 | 759.21758766 |
| 404.53074433656957 | 0.068329086773 | 404.53074433656957 | 2.19482136929e-10 | 404.53074433656957 | 753.630153731 |
| 403.6055428494551 | 0.0552164564037 | 403.6055428494551 | 1.54547647921e-10 | 403.6055428494551 | 746.912821623 |
| 402.68456375838923 | 0.0445317158407 | 402.68456375838923 | 1.08608297915e-10 | 402.68456375838923 | 739.060188152 |
| 401.76777822418643 | 0.0358433241853 | 401.76777822418643 | 7.61729894951e-11 | 401.76777822418643 | 730.077487943 |
| 400.85515766969536 | 0.028792876722 | 400.85515766969536 | 5.33183100004e-11 | 400.85515766969536 | 719.980160963 |
| 399.9466737768297 | 0.0230833977923 | 399.9466737768297 | 3.72468140032e-11 | 399.9466737768297 | 708.793336959 |
| 399.0422984836393 | 0.0184693788249 | 399.0422984836393 | 2.5968045439e-11 | 399.0422984836393 | 696.551244615 |
| 398.14200398142003 | 0.0147483248258 | 398.14200398142003 | 1.80686942709e-11 | 398.14200398142003 | 683.296553911 |
| 397.24576271186436 | 0.0117536005749 | 397.24576271186436 | 1.25473395057e-11 | 397.24576271186436 | 669.079660743 |
| 396.3535473642489 | 0.00934839314793 | 396.3535473642489 | 8.69588723783e-12 | 396.3535473642489 | 653.957923286 |
| 395.46533087266016 | 0.0074206302901 | 395.46533087266016 | 6.01469373241e-12 | 395.46533087266016 | 637.994859895 |
| 394.5810864132579 | 0.00587871473896 | 394.5810864132579 | 4.15193458833e-12 | 394.5810864132579 | 621.259318538 |
| 393.7007874015748 | 0.00464795297409 | 393.7007874015748 | 2.86038737094e-12 | 393.7007874015748 | 603.824627776 |
| 392.82440748985204 | 0.00366757321287 | 392.82440748985204 | 1.96669296097e-12 | 392.82440748985204 | 585.767739222 |
| 391.9519205644107 | 0.00288824193431 | 391.9519205644107 | 1.34953960851e-12 | 391.9519205644107 | 567.16837122 |
| 391.08330074305826 | 0.00227000095501 | 391.08330074305826 | 9.24212985834e-13 | 391.08330074305826 | 548.108163093 |
| 390.2185223725286 | 0.0017805582584 | 390.2185223725286 | 6.31678149488e-13 | 390.2185223725286 | 528.669848888 |
| 389.3575600259571 | 0.00139387554302 | 389.3575600259571 | 4.30880656621e-13 | 389.3575600259571 | 508.936458954 |
| 388.5003885003885 | 0.00108900395025 | 388.5003885003885 | 2.9332933342e-13 | 388.5003885003885 | 488.990557015 |
| 387.6469828143171 | 0.000849126793908 | 387.6469828143171 | 1.9929267897e-13 | 387.6469828143171 | 468.91351967 |
| 386.7973182052604 | 0.00066077446906 | 386.7973182052604 | 1.35133973166e-13 | 386.7973182052604 | 448.784864389 |
| 385.95137012736393 | 0.000513182183173 | 385.95137012736393 | 9.14481862522e-14 | 385.95137012736393 | 428.681631242 |
| 385.1091142490372 | 0.000397765836394 | 385.1091142490372 | 6.17622337621e-14 | 385.1091142490372 | 408.677822644 |
| 384.2705264506212 | 0.000307695376626 | 384.2705264506212 | 4.16301753527e-14 | 384.2705264506212 | 388.843904454 |
| 383.4355828220859 | 0.000237548357784 | 383.4355828220859 | 2.80046939773e-14 | 383.4355828220859 | 369.246370867 |
| 382.6042596607575 | 0.000183029314715 | 382.6042596607575 | 1.88014250053e-14 | 382.6042596607575 | 349.94737452 |
| 381.77653346907607 | 0.000140743006475 | 381.77653346907607 | 1.25976067008e-14 | 381.77653346907607 | 331.004422398 |
| 380.95238095238096 | 0.000108011633282 | 380.95238095238096 | 8.42408356654e-15 | 380.95238095238096 | 312.47013718 |
| 380.1317790167258 | 8.27278565282e-05 | 380.1317790167258 | 5.62204902439e-15 | 380.1317790167258 | 294.392082877 |
| 379.31470476672143 | 6.32368940356e-05 | 379.31470476672143 | 3.744587044e-15 | 379.31470476672143 | 276.812652832 |
| 378.5011355034065 | 4.82421662646e-05 | 378.5011355034065 | 2.48914714983e-15 | 378.5011355034065 | 259.76901745 |
| 377.69104872214524 | 3.67299700618e-05 | 377.69104872214524 | 1.651332584e-15 | 377.69104872214524 | 243.293128418 |
| 376.88442211055275 | 2.79094862687e-05 | 376.88442211055275 | 1.09334158494e-15 | 376.88442211055275 | 227.411775635 |
| 376.081233546446 | 2.11651133048e-05 | 376.081233546446 | 7.22461185077e-16 | 376.081233546446 | 212.146692618 |
| 375.28146109582184 | 1.60186839492e-05 | 375.28146109582184 | 4.76442513396e-16 | 375.28146109582184 | 197.514705817 |
| 374.48508301086 | 1.20995868283e-05 | 374.48508301086 | 3.13576731806e-16 | 374.48508301086 | 183.527922977 |
| 373.69207772795215 | 9.1211944435e-06 | 373.69207772795215 | 2.05974987716e-16 | 373.69207772795215 | 170.193955536 |
| 372.9024238657551 | 6.8623102185e-06 | 372.9024238657551 | 1.35027577081e-16 | 372.9024238657551 | 157.516169942 |
| 372.11610022326965 | 5.15260004543e-06 | 372.11610022326965 | 8.83421157573e-17 | 372.11610022326965 | 145.493962775 |
| 371.33308577794276 | 3.86117927354e-06 | 371.33308577794276 | 5.76833466513e-17 | 371.33308577794276 | 134.123054626 |
| 370.55335968379444 | 2.88769247417e-06 | 370.55335968379444 | 3.75898361775e-17 | 370.55335968379444 | 123.39579783 |
| 369.7769012695673 | 2.15535768227e-06 | 369.7769012695673 | 2.44471224042e-17 | 369.7769012695673 | 113.301493336 |
| 369.0036900369003 | 1.60555502203e-06 | 369.0036900369003 | 1.58680072619e-17 | 369.0036900369003 | 103.826712283 |
| 368.23370565852457 | 1.19362652352e-06 | 368.23370565852457 | 1.02790827681e-17 | 368.23370565852457 | 94.9556181333 |
| 367.4669279764821 | 8.85623534625e-07 | 367.4669279764821 | 6.64543881689e-18 | 367.4669279764821 | 86.6702855432 |
| 366.7033370003667 | 6.55793720368e-07 | 366.7033370003667 | 4.28775826295e-18 | 366.7033370003667 | 78.9510125643 |
| 365.9429129055867 | 4.84643921866e-07 | 365.9429129055867 | 2.76104985399e-18 | 365.9429129055867 | 71.7766231059 |
| 365.1856360316494 | 3.57450323897e-07 | 365.1856360316494 | 1.7744163518e-18 | 365.1856360316494 | 65.1247570241 |
| 364.4314868804664 | 2.63115251774e-07 | 364.4314868804664 | 1.13808353354e-18 | 364.4314868804664 | 58.972145595 |
| 363.68044611468054 | 1.93291942849e-07 | 363.68044611468054 | 7.28500869152e-19 | 363.68044611468054 | 53.2948705315 |
| 362.93249455601256 | 1.41715996746e-07 | 362.93249455601256 | 4.6539673695e-19 | 362.93249455601256 | 48.0686050993 |
| 362.1876131836291 | 1.03695854019e-07 | 362.1876131836291 | 2.96724849677e-19 | 362.1876131836291 | 43.2688362601 |
| 361.4457831325301 | 7.57253516926e-08 | 361.4457831325301 | 1.88808624072e-19 | 361.4457831325301 | 38.8710671299 |
| 360.7069856919562 | 5.51897709103e-08 | 360.7069856919562 | 1.19902174718e-19 | 360.7069856919562 | 34.8509993722 |
| 359.97120230381563 | 4.01433180774e-08 | 359.97120230381563 | 7.599230478e-20 | 359.97120230381563 | 31.1846954498 |
| 359.2384145611304 | 2.91410584361e-08 | 359.2384145611304 | 4.80672743207e-20 | 359.2384145611304 | 27.8487209365 |
| 358.50860420650093 | 2.11122613833e-08 | 358.50860420650093 | 3.03435715943e-20 | 358.50860420650093 | 24.8202673327 |
| 357.7817531305903 | 1.52651670335e-08 | 357.7817531305903 | 1.91170652624e-20 | 357.7817531305903 | 22.0772560401 |
| 357.057843370626 | 1.10155386651e-08 | 357.057843370626 | 1.20202380739e-20 | 357.057843370626 | 19.5984243324 |
| 356.33685710892024 | 7.93317960905e-09 | 356.33685710892024 | 7.54296752262e-21 | 356.33685710892024 | 17.3633943044 |
| 355.6187766714082 | 5.70198672018e-09 | 355.6187766714082 | 4.72398736724e-21 | 355.6187766714082 | 15.352725899 |
| 354.9035845262037 | 4.09018056399e-09 | 354.9035845262037 | 2.95265386583e-21 | 354.9035845262037 | 13.5479551979 |
| 354.1912632821723 | 2.92816889013e-09 | 354.1912632821723 | 1.84184750463e-21 | 354.1912632821723 | 11.9316192239 |
| 353.48179568752204 | 2.09212256692e-09 | 353.48179568752204 | 1.14665329177e-21 | 353.48179568752204 | 10.487268532 |
| 352.77516462841015 | 1.49181676755e-09 | 352.77516462841015 | 7.12439342995e-22 | 352.77516462841015 | 9.19946888183 |
| 352.07135312756714 | 1.06164960044e-09 | 352.07135312756714 | 4.41774848628e-22 | 352.07135312756714 | 8.05379327067 |
| 351.3703443429374 | 7.5402244133e-10 | 351.3703443429374 | 2.73395523139e-22 | 351.3703443429374 | 7.03680558058 |
| 350.6721215663355 | 5.34471676391e-10 | 350.6721215663355 | 1.68857023715e-22 | 350.6721215663355 | 6.13603704897 |
| 349.9766682221185 | 3.780963371e-10 | 349.9766682221185 | 1.04084049532e-22 | 349.9766682221185 | 5.33995671642 |
| 349.2839678658749 | 2.66942420569e-10 | 349.2839678658749 | 6.40304494478e-23 | 349.2839678658749 | 4.63793693939 |
| 348.59400418312805 | 1.88091888224e-10 | 348.59400418312805 | 3.93120995217e-23 | 348.59400418312805 | 4.02021498144 |
| 347.90676098805517 | 1.32269552654e-10 | 347.90676098805517 | 2.40881388249e-23 | 347.90676098805517 | 3.47785161727 |
| 347.2222222222222 | 9.28297216346e-11 | 347.2222222222222 | 1.47305027879e-23 | 347.2222222222222 | 3.00268760045 |
| 346.54037195333257 | 6.5020687195e-11 | 346.54037195333257 | 8.99019711403e-24 | 346.54037195333257 | 2.58729876133 |
| 345.8611943739912 | 4.54520425455e-11 | 345.8611943739912 | 5.47593362342e-24 | 345.8611943739912 | 2.22495041646 |
| 345.1846738004832 | 3.17097337087e-11 | 345.1846738004832 | 3.32877510019e-24 | 345.1846738004832 | 1.90955168771 |
| 344.5107946715664 | 2.20784776748e-11 | 344.5107946715664 | 2.01951949916e-24 | 344.5107946715664 | 1.63561024789 |
| 343.8395415472779 | 1.53420358843e-11 | 343.8395415472779 | 1.22278179653e-24 | 343.8395415472779 | 1.39818793296 |
| 343.17089910775564 | 1.06398180406e-11 | 343.17089910775564 | 7.38902602227e-25 | 343.17089910775564 | 1.19285758727 |
| 342.50485215207215 | 7.36415200079e-12 | 342.50485215207215 | 4.45618019747e-25 | 342.50485215207215 | 1.01566144065 |
| 341.84138559708293 | 5.0868463853e-12 | 341.84138559708293 | 2.68210397406e-25 | 341.84138559708293 | 0.86307125327 |
| 341.1804844762879 | 3.50680676938e-12 | 341.1804844762879 | 1.61111221996e-25 | 341.1804844762879 | 0.731950407104 |
| 340.522133938706 | 2.41275032793e-12 | 340.522133938706 | 9.65858020176e-26 | 340.522133938706 | 0.619518071598 |
| 339.86631924776253 | 1.65672437983e-12 | 339.86631924776253 | 5.7788058748e-26 | 339.86631924776253 | 0.523315525434 |
| 339.2130257801899 | 1.13533878711e-12 | 339.2130257801899 | 3.45064464686e-26 | 339.2130257801899 | 0.441174676429 |
| 338.56223902494077 | 7.76493846043e-13 | 338.56223902494077 | 2.05636254566e-26 | 338.56223902494077 | 0.371188787063 |
| 337.91394458211306 | 5.30014671482e-13 | 337.91394458211306 | 1.22302813851e-26 | 337.91394458211306 | 0.311685383814 |
| 337.2681281618887 | 3.61056439137e-13 | 337.2681281618887 | 7.25956384551e-27 | 337.2681281618887 | 0.261201304028 |
| 336.6247755834829 | 2.45470680572e-13 | 336.6247755834829 | 4.30052943942e-27 | 336.6247755834829 | 0.218459814113 |
| 335.9838727741068 | 1.66556462008e-13 | 335.9838727741068 | 2.54255664181e-27 | 335.9838727741068 | 0.182349716993 |
| 335.3454057679409 | 1.12787423258e-13 | 335.3454057679409 | 1.50022591802e-27 | 335.3454057679409 | 0.151906354704 |
| 334.709360705121 | 7.62249573015e-14 | 334.709360705121 | 8.8344599669e-28 | 334.709360705121 | 0.126294403199 |
| 334.07572383073494 | 5.14127732872e-14 | 334.07572383073494 | 5.19207151284e-28 | 334.07572383073494 | 0.104792350603 |
| 333.44448149383123 | 3.46084539083e-14 | 333.44448149383123 | 3.04536000259e-28 | 333.44448149383123 | 0.0867785468531 |
| 332.81562014643885 | 2.32504140058e-14 | 332.81562014643885 | 1.78268220233e-28 | 332.81562014643885 | 0.0717187115282 |
| 332.1891263425977 | 1.55889372049e-14 | 332.1891263425977 | 1.04146944524e-28 | 332.1891263425977 | 0.0591547873665 |
| 331.5649867374005 | 1.04313291421e-14 | 331.5649867374005 | 6.07234520476e-29 | 331.5649867374005 | 0.0486950291749 |
| 330.9431880860452 | 6.9662669723e-15 | 330.9431880860452 | 3.53348854127e-29 | 330.9431880860452 | 0.0400052212621 |
| 330.323717242898 | 4.64299164995e-15 | 330.323717242898 | 2.05205140975e-29 | 330.323717242898 | 0.0328009208863 |
| 329.70656116056705 | 3.08839623226e-15 | 329.70656116056705 | 1.18935116256e-29 | 329.70656116056705 | 0.0268406302973 |
| 329.0917068889864 | 2.05024351909e-15 | 329.0917068889864 | 6.87969651837e-30 | 329.0917068889864 | 0.0219198055382 |
| 328.47914157451 | 1.35836099863e-15 | 328.47914157451 | 3.97160249553e-30 | 328.47914157451 | 0.0178656160856 |
| 327.86885245901635 | 8.98177726691e-16 | 327.86885245901635 | 2.28822951839e-30 | 327.86885245901635 | 0.0145323754904 |
| 327.26082687902255 | 5.92716039535e-16 | 327.26082687902255 | 1.31574190646e-30 | 327.26082687902255 | 0.0117975693114 |
| 326.6550522648083 | 3.90362777667e-16 | 326.6550522648083 | 7.55055969163e-31 | 326.6550522648083 | 0.00955841269142 |
| 326.05151613955 | 2.56582740808e-16 | 326.05151613955 | 4.32439043266e-31 | 326.05151613955 | 0.00772887584279 |
| 325.4502061184639 | 1.68315379471e-16 | 325.4502061184639 | 2.47176947633e-31 | 325.4502061184639 | 0.00623712139932 |
| 324.8511099079588 | 1.10193881522e-16 | 324.8511099079588 | 1.4100299787e-31 | 324.8511099079588 | 0.00502330301978 |
| 324.25421530479895 | 7.19993343625e-17 | 324.25421530479895 | 8.02760587441e-32 | 324.25421530479895 | 0.00403767974784 |
| 323.65951019527455 | 4.69501302038e-17 | 323.65951019527455 | 4.56122033862e-32 | 323.65951019527455 | 0.00323900542446 |
| 323.0669825543829 | 3.05550106722e-17 | 323.0669825543829 | 2.58650532308e-32 | 323.0669825543829 | 0.00259315689713 |
| 322.4766204450177 | 1.98456531765e-17 | 322.4766204450177 | 1.46380428819e-32 | 322.4766204450177 | 0.00207196887051 |
| 321.88841201716735 | 1.28642856073e-17 | 321.88841201716735 | 8.26780010273e-33 | 321.88841201716735 | 0.00165224699733 |
| 321.3023455071222 | 8.32229810316e-18 | 321.3023455071222 | 4.6605184825e-33 | 321.3023455071222 | 0.00131493422529 |
| 320.71840923669015 | 5.37326402294e-18 | 320.71840923669015 | 2.62189814036e-33 | 320.71840923669015 | 0.00104440850684 |
| 320.1365916124213 | 3.4623455666e-18 | 320.1365916124213 | 1.47209117937e-33 | 320.1365916124213 | 0.000827892760144 |
| 319.5568811248402 | 2.22658848972e-18 | 319.5568811248402 | 8.24880276428e-34 | 319.5568811248402 | 0.000654960459329 |
| 318.97926634768737 | 1.42904802963e-18 | 318.97926634768737 | 4.6130104908e-34 | 318.97926634768737 | 0.00051712244982 |
| 318.40373593716834 | 9.15358057042e-19 | 318.40373593716834 | 2.57463268945e-34 | 318.40373593716834 | 0.000407482550155 |
| 317.8302786312109 | 5.85157140296e-19 | 317.8302786312109 | 1.43411318867e-34 | 317.8302786312109 | 0.000320451236507 |
| 317.2588832487309 | 3.73328655289e-19 | 317.2588832487309 | 7.97239658254e-35 | 317.2588832487309 | 0.000251508230067 |
| 316.6895386889053 | 2.37710004336e-19 | 316.6895386889053 | 4.42315005139e-35 | 316.6895386889053 | 0.000197006140645 |
| 316.1222339304531 | 1.51057019202e-19 | 316.1222339304531 | 2.44912958662e-35 | 316.1222339304531 | 0.000154008481399 |
| 315.55695803092453 | 9.58013607061e-20 | 315.55695803092453 | 1.35340934455e-35 | 315.55695803092453 | 0.000120156377535 |
| 314.99370012599746 | 6.0637286839e-20 | 314.99370012599746 | 7.46421070284e-36 | 314.99370012599746 | 9.35591630377e-05 |
| 314.432449428781 | 3.83040909981e-20 | 314.432449428781 | 4.10843030166e-36 | 314.432449428781 | 7.27048097406e-05 |
| 313.8731952291274 | 2.4148373034e-20 | 313.8731952291274 | 2.25686338515e-36 | 313.8731952291274 | 5.63867767364e-05 |
| 313.31592689295036 | 1.51938525521e-20 | 313.31592689295036 | 1.23729122067e-36 | 313.31592689295036 | 4.36444183222e-05 |
| 312.76063386155124 | 9.54081016598e-21 | 312.76063386155124 | 6.76980104607e-37 | 312.76063386155124 | 3.37145574196e-05 |
| 312.2073056509522 | 5.97915645539e-21 | 312.2073056509522 | 3.69672541688e-37 | 312.2073056509522 | 2.59922292695e-05 |
| 311.65593185123623 | 3.73965808008e-21 | 311.65593185123623 | 2.01463231103e-37 | 311.65593185123623 | 1.9998936816e-05 |
| 311.1065021258944 | 2.33432429471e-21 | 311.1065021258944 | 1.09575058193e-37 | 311.1065021258944 | 1.53570430052e-05 |
| 310.5590062111801 | 1.45421225173e-21 | 310.5590062111801 | 5.94791753362e-38 | 310.5590062111801 | 1.17691637539e-05 |
| 310.01343391546965 | 9.04131756812e-22 | 310.01343391546965 | 3.22222216359e-38 | 310.01343391546965 | 9.00162514611e-06 |
| 309.4697751186301 | 5.61013049485e-22 | 309.4697751186301 | 1.7421411381e-38 | 309.4697751186301 | 6.87121526852e-06 |
| 308.9280197713932 | 3.47417361731e-22 | 308.9280197713932 | 9.40044699814e-39 | 308.9280197713932 | 5.23459997604e-06 |
| 308.3881578947368 | 2.14717473112e-22 | 308.3881578947368 | 5.06233623989e-39 | 308.3881578947368 | 3.97988713251e-06 |
| 307.8501795792714 | 1.3244042692e-22 | 307.8501795792714 | 2.72076344193e-39 | 307.8501795792714 | 3.01991921835e-06 |
| 307.31407498463426 | 8.15288039759e-23 | 307.31407498463426 | 1.45937832861e-39 | 307.31407498463426 | 2.28695283963e-06 |
| 306.77983433888943 | 5.00885990938e-23 | 306.77983433888943 | 7.81236121966e-40 | 306.77983433888943 | 1.72844834409e-06 |
| 306.2474479379338 | 3.07117111064e-23 | 306.2474479379338 | 4.17382322792e-40 | 306.2474479379338 | 1.30374577435e-06 |
| 305.7169061449098 | 1.8793447586e-23 | 305.7169061449098 | 2.22547695312e-40 | 305.7169061449098 | 9.8144673762e-07 |
| 305.1881993896236 | 1.14774712813e-23 | 305.1881993896236 | 1.18426655353e-40 | 305.1881993896236 | 7.37357107157e-07 |
| 304.6613181679699 | 6.99557290323e-24 | 304.6613181679699 | 6.28945682434e-41 | 304.6613181679699 | 5.52874193056e-07 |
| 304.1362530413625 | 4.25537338887e-24 | 304.1362530413625 | 3.33360492946e-41 | 304.1362530413625 | 4.13725299813e-07 |
| 303.61299463617036 | 2.5833864312e-24 | 303.61299463617036 | 1.76340648044e-41 | 303.61299463617036 | 3.08983403845e-07 |
| 303.09153364316023 | 1.56523081466e-24 | 303.09153364316023 | 9.30953627676e-42 | 303.09153364316023 | 2.30300849519e-07 |
| 302.571860816944 | 9.46465348728e-25 | 302.571860816944 | 4.90502220784e-42 | 302.571860816944 | 1.71314151559e-07 |
| 302.0539669754329 | 5.71173910491e-25 | 302.0539669754329 | 2.57923682451e-42 | 302.0539669754329 | 1.27182762073e-07 |
| 301.5378429992964 | 3.44008615587e-25 | 301.5378429992964 | 1.35356394242e-42 | 301.5378429992964 | 9.42324707151e-08 |
| 301.02347983142687 | 2.06779552469e-25 | 301.02347983142687 | 7.08930475013e-43 | 301.02347983142687 | 6.96803329032e-08 |
| 300.5108684764098 | 1.24046117708e-25 | 300.5108684764098 | 3.70566220779e-43 | 300.5108684764098 | 5.14229714533e-08 |

| **GON2-Al3** | | **GON2-Al4** | | **GON2-Al5** | |
| --- | --- | --- | --- | --- | --- |
| Wavelength (nm) | Abs | Wavelength (nm) | Abs | Wavelength (nm) | Abs |
| 2000.0 | 1.44922708398e-05 | 2000.0 | 4.74380239343e-15 | 2000.0 | 0.213357603998 |
| 1977.5873434410018 | 1.89275708258e-05 | 1977.5873434410018 | 7.08762604958e-15 | 1977.5873434410018 | 0.257430559224 |
| 1955.671447196871 | 2.46712229514e-05 | 1955.671447196871 | 1.0568474757e-14 | 1955.671447196871 | 0.310092535469 |
| 1934.2359767891683 | 3.20939993277e-05 | 1934.2359767891683 | 1.57275525936e-14 | 1934.2359767891683 | 0.372908345555 |
| 1913.265306122449 | 4.16672047892e-05 | 1913.265306122449 | 2.33586258779e-14 | 1913.265306122449 | 0.447705800707 |
| 1892.7444794952683 | 5.39886248552e-05 | 1892.7444794952683 | 3.46234825485e-14 | 1892.7444794952683 | 0.536615636397 |
| 1872.6591760299625 | 6.98148065468e-05 | 1872.6591760299625 | 5.12190500268e-14 | 1872.6591760299625 | 0.642116708802 |
| 1852.9956763434218 | 9.01011203758e-05 | 1852.9956763434218 | 7.56187689457e-14 | 1852.9956763434218 | 0.767087017555 |
| 1833.7408312958437 | 0.000116051358182 | 1833.7408312958437 | 1.11420473442e-13 | 1833.7408312958437 | 0.914861144732 |
| 1814.8820326678765 | 0.000149178986587 | 1814.8820326678765 | 1.63846703899e-13 | 1814.8820326678765 | 1.08929473201 |
| 1796.4071856287424 | 0.000191382609497 | 1796.4071856287424 | 2.40462706914e-13 | 1796.4071856287424 | 1.29483664653 |
| 1778.3046828689983 | 0.000245038706514 | 1778.3046828689983 | 3.52204635239e-13 | 1778.3046828689983 | 1.53660950978 |
| 1760.5633802816901 | 0.000313115319891 | 1760.5633802816901 | 5.14848813013e-13 | 1760.5633802816901 | 1.820499282 |
| 1743.1725740848342 | 0.000399311075009 | 1743.1725740848342 | 7.51106771534e-13 | 1743.1725740848342 | 2.15325460443 |
| 1726.1219792865363 | 0.000508224754049 | 1726.1219792865363 | 1.09360617012e-12 | 1726.1219792865363 | 2.54259660308 |
| 1709.4017094017095 | 0.000645561617073 | 1709.4017094017095 | 1.58912309489e-12 | 1709.4017094017095 | 2.99733984692 |
| 1693.002257336343 | 0.000818383798531 | 1693.002257336343 | 2.30457814926e-12 | 1693.002257336343 | 3.52752513016 |
| 1676.9144773616547 | 0.00103541342116 | 1676.9144773616547 | 3.33551312028e-12 | 1676.9144773616547 | 4.14456470856 |
| 1661.1295681063123 | 0.00130739858642 | 1661.1295681063123 | 4.81804863666e-12 | 1661.1295681063123 | 4.86140056325 |
| 1645.6390565002741 | 0.00164755414523 | 1645.6390565002741 | 6.94571596015e-12 | 1645.6390565002741 | 5.69267618782 |
| 1630.4347826086955 | 0.00207209115135 | 1630.4347826086955 | 9.99309857906e-12 | 1630.4347826086955 | 6.65492229483 |
| 1615.5088852988692 | 0.0026008511788 | 1615.5088852988692 | 1.43489670083e-11 | 1615.5088852988692 | 7.76675671269 |
| 1600.8537886872998 | 0.00325806427377 | 1600.8537886872998 | 2.05626183756e-11 | 1600.8537886872998 | 9.04909859139 |
| 1586.4621893178212 | 0.00407325223809 | 1586.4621893178212 | 2.9408542883e-11 | 1586.4621893178212 | 10.5253968532 |
| 1572.3270440251572 | 0.00508230223537 | 1572.3270440251572 | 4.19764697469e-11 | 1572.3270440251572 | 12.2218726104 |
| 1558.4415584415583 | 0.00632873939988 | 1558.4415584415583 | 5.97964812778e-11 | 1558.4415584415583 | 14.1677750232 |
| 1544.799176107106 | 0.00786523123916 | 1544.799176107106 | 8.50124747976e-11 | 1544.799176107106 | 16.3956497908 |
| 1531.3935681470139 | 0.00975536117729 | 1531.3935681470139 | 1.20622132597e-10 | 1531.3935681470139 | 18.9416191449 |
| 1518.2186234817814 | 0.0120757136079 | 1518.2186234817814 | 1.70808177539e-10 | 1518.2186234817814 | 21.8456718647 |
| 1505.2684395383842 | 0.0149183183275 | 1505.2684395383842 | 2.41394643179e-10 | 1505.2684395383842 | 25.1519614358 |
| 1492.5373134328358 | 0.0183935082107 | 1492.5373134328358 | 3.40473966944e-10 | 1492.5373134328358 | 28.9091100529 |
| 1480.0197335964478 | 0.0226332504631 | 1480.0197335964478 | 4.79266977586e-10 | 1480.0197335964478 | 33.1705157029 |
| 1467.7103718199608 | 0.0277950187414 | 1467.7103718199608 | 6.73299690437e-10 | 1467.7103718199608 | 37.9946590778 |
| 1455.604075691412 | 0.0340662808289 | 1455.604075691412 | 9.44010090124e-10 | 1455.604075691412 | 43.4454065492 |
| 1443.6958614051973 | 0.0416696843618 | 1443.6958614051973 | 1.32093719997e-09 | 1443.6958614051973 | 49.592304902 |
| 1431.9809069212408 | 0.05086903126 | 1431.9809069212408 | 1.84469690222e-09 | 1431.9809069212408 | 56.5108629726 |
| 1420.4545454545455 | 0.0619761399371 | 1420.4545454545455 | 2.57101836642e-09 | 1420.4545454545455 | 64.2828147841 |
| 1409.1122592766555 | 0.0753587029527 | 1409.1122592766555 | 3.57620703177e-09 | 1409.1122592766555 | 72.9963582174 |
| 1397.9496738117427 | 0.0914492563844 | 1397.9496738117427 | 4.96452199937e-09 | 1397.9496738117427 | 82.7463627192 |
| 1386.9625520110958 | 0.110755385682 | 1386.9625520110958 | 6.87811668716e-09 | 1386.9625520110958 | 93.6345390407 |
| 1376.1467889908256 | 0.133871300927 | 1376.1467889908256 | 9.5104036972e-09 | 1376.1467889908256 | 105.769563525 |
| 1365.4984069185252 | 0.161490922019 | 1365.4984069185252 | 1.31239835761e-08 | 1365.4984069185252 | 119.267149054 |
| 1355.0135501355014 | 0.194422621105 | 1355.0135501355014 | 1.80746425498e-08 | 1355.0135501355014 | 134.250054403 |
| 1344.688480502017 | 0.233605775202 | 1344.688480502017 | 2.48434021481e-08 | 1344.688480502017 | 150.848023529 |
| 1334.5195729537365 | 0.280129286183 | 1334.5195729537365 | 3.40792269562e-08 | 1334.5195729537365 | 169.197646112 |
| 1324.5033112582782 | 0.335252227593 | 1324.5033112582782 | 4.66558078018e-08 | 1324.5033112582782 | 189.442130683 |
| 1314.6362839614374 | 0.400426777856 | 1314.6362839614374 | 6.374689044e-08 | 1314.6362839614374 | 211.730981763 |
| 1304.9151805132665 | 0.477323596756 | 1304.9151805132665 | 8.69259828002e-08 | 1304.9151805132665 | 236.219572669 |
| 1295.3367875647668 | 0.567859796181 | 1295.3367875647668 | 1.18298034112e-07 | 1295.3367875647668 | 263.068606131 |
| 1285.8979854264894 | 0.674229646522 | 1285.8979854264894 | 1.60672946735e-07 | 1285.8979854264894 | 292.443455415 |
| 1276.5957446808509 | 0.798938146238 | 1276.5957446808509 | 2.17793695028e-07 | 1276.5957446808509 | 324.51337951 |
| 1267.427122940431 | 0.944837563413 | 1267.427122940431 | 2.94635559605e-07 | 1267.427122940431 | 359.450606917 |
| 1258.3892617449665 | 1.1151670341 | 1258.3892617449665 | 3.97797758506e-07 | 1258.3892617449665 | 397.429283839 |
| 1249.4793835901708 | 1.31359527233 | 1249.4793835901708 | 5.36014842756e-07 | 1249.4793835901708 | 438.624283982 |
| 1240.6947890818858 | 1.54426641047 | 1240.6947890818858 | 7.20822963167e-07 | 1240.6947890818858 | 483.209878873 |
| 1232.0328542094455 | 1.81184894531 | 1232.0328542094455 | 9.67425931708e-07 | 1232.0328542094455 | 531.358269435 |
| 1223.4910277324632 | 2.12158771521 | 1223.4910277324632 | 1.29581841894e-06 | 1223.4910277324632 | 583.237981646 |
| 1215.0668286755772 | 2.47935877566 | 1215.0668286755772 | 1.73223929685e-06 | 1215.0668286755772 | 639.012131347 |
| 1206.7578439259853 | 2.89172697503 | 1206.7578439259853 | 2.31104785176e-06 | 1206.7578439259853 | 698.83656567 |
| 1198.5617259288852 | 3.36600595936 | 1198.5617259288852 | 3.07714033373e-06 | 1198.5617259288852 | 762.857891103 |
| 1190.4761904761904 | 3.91032025366 | 1190.4761904761904 | 4.08905527496e-06 | 1190.4761904761904 | 831.211400837 |
| 1182.4990145841543 | 4.53366897953 | 1182.4990145841543 | 5.42295466038e-06 | 1182.4990145841543 | 904.018916749 |
| 1174.6280344557556 | 5.24599067325 | 1174.6280344557556 | 7.1777161437e-06 | 1174.6280344557556 | 981.386564096 |
| 1166.8611435239206 | 6.05822856775 | 1166.8611435239206 | 9.48143122219e-06 | 1166.8611435239206 | 1063.40249967 |
| 1159.19629057187 | 6.98239559536 | 1159.19629057187 | 1.24996782071e-05 | 1159.19629057187 | 1150.1346168 |
| 1151.6314779270633 | 8.03163825776 | 1151.6314779270633 | 1.64460300778e-05 | 1151.6314779270633 | 1241.62825298 |
| 1144.1647597254005 | 9.22029839749 | 1144.1647597254005 | 2.15953696261e-05 | 1144.1647597254005 | 1337.90392828 |
| 1136.794240242516 | 10.5639717915 | 1136.794240242516 | 2.83007221542e-05 | 1136.794240242516 | 1438.95514465 |
| 1129.5180722891566 | 12.0795623766 | 1129.5180722891566 | 3.7014484702e-05 | 1129.5180722891566 | 1544.74627786 |
| 1122.334455667789 | 13.7853308074 | 1122.334455667789 | 4.8315136653e-05 | 1122.334455667789 | 1655.21059528 |
| 1115.2416356877322 | 15.7009359487 | 1115.2416356877322 | 6.2940767047e-05 | 1115.2416356877322 | 1770.24843352 |
| 1108.2379017362393 | 17.8474678102 | 1108.2379017362393 | 8.18310577705e-05 | 1108.2379017362393 | 1889.72557029 |
| 1101.3215859030836 | 20.2474703542 | 1101.3215859030836 | 0.00010617972926 | 1101.3215859030836 | 2013.47182491 |
| 1094.4910616563297 | 22.9249525441 | 1094.4910616563297 | 0.000137499898406 | 1094.4910616563297 | 2141.27992099 |
| 1087.7447425670775 | 25.9053859568 | 1087.7447425670775 | 0.000177705330888 | 1087.7447425670775 | 2272.90464371 |
| 1081.081081081081 | 29.2156872629 | 1081.081081081081 | 0.000229211208269 | 1081.081081081081 | 2408.06232213 |
| 1074.4985673352435 | 32.8841838825 | 1074.4985673352435 | 0.000295058792344 | 1074.4985673352435 | 2546.43066445 |
| 1067.995728017088 | 36.9405611628 | 1067.995728017088 | 0.00037906927664 | 1067.995728017088 | 2687.64897098 |
| 1061.5711252653928 | 41.4157894879 | 1061.5711252653928 | 0.000486033191744 | 1061.5711252653928 | 2831.31874589 |
| 1055.2233556102708 | 46.3420298377 | 1055.2233556102708 | 0.000621943004714 | 1055.2233556102708 | 2977.00472446 |
| 1048.951048951049 | 51.7525164544 | 1048.951048951049 | 0.000794278053967 | 1048.951048951049 | 3124.23632748 |
| 1042.752867570386 | 57.6814154554 | 1042.752867570386 | 0.0010123527244 | 1042.752867570386 | 3272.50954949 |
| 1036.6275051831374 | 64.1636584561 | 1036.6275051831374 | 0.00128774083155 | 1036.6275051831374 | 3421.28928142 |
| 1030.5736860185502 | 71.2347505307 | 1030.5736860185502 | 0.00163479159085 | 1030.5736860185502 | 3570.01206227 |
| 1024.5901639344263 | 78.930552145 | 1024.5901639344263 | 0.00207125534568 | 1024.5901639344263 | 3718.08924824 |
| 1018.6757215619693 | 87.2870350417 | 1018.6757215619693 | 0.00261904046592 | 1018.6757215619693 | 3864.91058088 |
| 1012.829169480081 | 96.3400124458 | 1012.829169480081 | 0.00330512656366 | 1012.829169480081 | 4009.84812995 |
| 1007.0493454179255 | 106.124844373 | 1007.0493454179255 | 0.00416266346211 | 1007.0493454179255 | 4152.26057964 |
| 1001.3351134846461 | 116.676119282 | 1001.3351134846461 | 0.00523229026048 | 1001.3351134846461 | 4291.49782117 |
| 995.6853634251576 | 128.027313771 | 995.6853634251576 | 0.00656371442749 | 995.6853634251576 | 4426.90580857 |
| 990.0990099009902 | 140.210432534 | 990.0990099009902 | 0.00821759719487 | 990.0990099009902 | 4557.83162929 |
| 984.5749917952082 | 153.255631272 | 984.5749917952082 | 0.0102677986797 | 984.5749917952082 | 4683.62873629 |
| 979.1122715404699 | 167.190825777 | 979.1122715404699 | 0.012804044206 | 979.1122715404699 | 4803.66228442 |
| 973.7098344693281 | 182.041290893 | 973.7098344693281 | 0.0159350822898 | 973.7098344693281 | 4917.31451006 |
| 968.3666881859263 | 197.829253539 | 968.3666881859263 | 0.0197924147528 | 968.3666881859263 | 5023.99009107 |
| 963.0818619582664 | 214.573484427 | 963.0818619582664 | 0.0245346905002 | 963.0818619582664 | 5123.12142205 |
| 957.8544061302682 | 232.288893513 | 957.8544061302682 | 0.0303528666707 | 957.8544061302682 | 5214.17373969 |
| 952.6833915528738 | 250.986134582 | 952.6833915528738 | 0.0374762541769 | 952.6833915528738 | 5296.65003326 |
| 947.5679090334806 | 270.671224643 | 947.5679090334806 | 0.0461795791154 | 947.5679090334806 | 5370.09567683 |
| 942.5070688030161 | 291.345184027 | 942.5070688030161 | 0.0567912071145 | 942.5070688030161 | 5434.1027226 |
| 937.4999999999999 | 313.003703236 | 937.4999999999999 | 0.0697026943819 | 937.4999999999999 | 5488.31379787 |
| 932.5458501709667 | 335.636842575 | 932.5458501709667 | 0.0853798469238 | 932.5458501709667 | 5532.42555321 |
| 927.643784786642 | 359.228770574 | 927.643784786642 | 0.104375488032 | 927.643784786642 | 5566.19161454 |
| 922.7929867733004 | 383.75754701 | 922.7929867733004 | 0.127344153512 | 922.7929867733004 | 5589.42499829 |
| 917.9926560587514 | 409.194956033 | 917.9926560587514 | 0.155058954039 | 917.9926560587514 | 5601.99995584 |
| 913.2420091324201 | 435.50639451 | 913.2420091324201 | 0.188430864216 | 913.2420091324201 | 5603.853221 |
| 908.5402786190186 | 462.650820161 | 908.5402786190186 | 0.22853071802 | 908.5402786190186 | 5594.98464251 |
| 903.8867128653209 | 490.580763415 | 903.8867128653209 | 0.276614209974 | 903.8867128653209 | 5575.45719197 |
| 899.2805755395683 | 519.242406184 | 899.2805755395683 | 0.334150220023 | 899.2805755395683 | 5545.3963463 |
| 894.7211452430658 | 548.575729858 | 894.7211452430658 | 0.402852797225 | 894.7211452430658 | 5504.98885239 |
| 890.2077151335311 | 578.514733933 | 890.2077151335311 | 0.484717152276 | 890.2077151335311 | 5454.48089037 |
| 885.7395925597874 | 608.987725621 | 885.7395925597874 | 0.582060020798 | 885.7395925597874 | 5394.17566005 |
| 881.316098707403 | 639.917679732 | 881.316098707403 | 0.697564767432 | 881.316098707403 | 5324.43042309 |
| 876.9365682548962 | 671.222666981 | 876.9365682548962 | 0.834331604094 | 876.9365682548962 | 5245.65304068 |
| 872.6003490401396 | 702.816347736 | 872.6003490401396 | 0.995933293271 | 872.6003490401396 | 5158.29805317 |
| 868.3068017366135 | 734.608527068 | 868.3068017366135 | 1.18647669782 | 868.3068017366135 | 5062.86235404 |
| 864.0552995391705 | 766.505765828 | 864.0552995391705 | 1.41067052112 | 864.0552995391705 | 4959.88051536 |
| 859.8452278589854 | 798.412041429 | 859.8452278589854 | 1.6738995546 | 859.8452278589854 | 4849.919826 |
| 855.6759840273816 | 830.229450947 | 855.6759840273816 | 1.98230571193 | 855.6759840273816 | 4733.57510677 |
| 851.5469770082316 | 861.8589483 | 851.5469770082316 | 2.34287607958 | 851.5469770082316 | 4611.46336856 |
| 847.457627118644 | 893.20110641 | 847.457627118644 | 2.76353815065 | 847.457627118644 | 4484.21838027 |
| 843.4073657576608 | 924.156894628 | 843.4073657576608 | 3.25326233105 | 843.4073657576608 | 4352.48521332 |
| 839.3956351426972 | 954.628461174 | 839.3956351426972 | 3.82217171445 | 839.3956351426972 | 4216.91482779 |
| 835.421888053467 | 984.51991002 | 835.421888053467 | 4.48165901163 | 835.421888053467 | 4078.15876341 |
| 831.4855875831485 | 1013.73806151 | 831.4855875831485 | 5.24451039286 | 831.4855875831485 | 3936.8639951 |
| 827.5862068965516 | 1042.19318601 | 827.5862068965516 | 6.12503585515 | 827.5862068965516 | 3793.6680087 |
| 823.7232289950576 | 1069.79970023 | 823.7232289950576 | 7.13920556193 | 823.7232289950576 | 3649.1941478 |
| 819.8961464881114 | 1096.47681613 | 819.8961464881114 | 8.30479141856 | 819.8961464881114 | 3504.04727713 |
| 816.1044613710554 | 1122.14913327 | 816.1044613710554 | 9.64151294524 | 816.1044613710554 | 3358.80980171 |
| 812.3476848090983 | 1146.74716589 | 812.3476848090983 | 11.1711862891 | 812.3476848090983 | 3214.03807499 |
| 808.6253369272237 | 1170.20779743 | 808.6253369272237 | 12.9178749807 | 808.6253369272237 | 3070.25922207 |
| 804.9369466058491 | 1192.47465611 | 804.9369466058491 | 14.9080407907 | 804.9369466058491 | 2927.96839764 |
| 801.2820512820513 | 1213.49840662 | 801.2820512820513 | 17.170692778 | 801.2820512820513 | 2787.6264912 |
| 797.6601967561818 | 1233.23695433 | 797.6601967561818 | 19.7375323523 | 797.6601967561818 | 2649.65828554 |
| 794.0709370037056 | 1251.65555995 | 794.0709370037056 | 22.6430918945 | 794.0709370037056 | 2514.45106771 |
| 790.5138339920949 | 1268.72686428 | 790.5138339920949 | 25.9248642051 | 790.5138339920949 | 2382.35368583 |
| 786.9884575026232 | 1284.43082402 | 786.9884575026232 | 29.6234197768 | 786.9884575026232 | 2253.67603895 |
| 783.4943849569078 | 1298.75456135 | 783.4943849569078 | 33.7825086288 | 783.4943849569078 | 2128.68898208 |
| 780.0312012480499 | 1311.69213164 | 780.0312012480499 | 38.449143196 | 780.0312012480499 | 2007.62462364 |
| 776.598498576236 | 1323.24421469 | 776.598498576236 | 43.6736585495 | 776.598498576236 | 1890.67698868 |
| 773.1958762886597 | 1333.41773672 | 773.1958762886597 | 49.509746039 | 773.1958762886597 | 1778.00301736 |
| 769.8229407236336 | 1342.2254312 | 769.8229407236336 | 56.014456303 | 769.8229407236336 | 1669.72386577 |
| 766.4793050587633 | 1349.68534776 | 766.4793050587633 | 63.2481674978 | 766.4793050587633 | 1565.92647385 |
| 763.1645891630628 | 1355.82031918 | 763.1645891630628 | 71.2745145611 | 763.1645891630628 | 1466.66536375 |
| 759.8784194528876 | 1360.65739722 | 759.8784194528876 | 80.1602753517 | 759.8784194528876 | 1371.96463136 |
| 756.6204287515762 | 1364.22726819 | 756.6204287515762 | 89.9752096166 | 756.6204287515762 | 1281.82009335 |
| 753.390256152687 | 1366.56365963 | 753.390256152687 | 100.791846919 | 753.390256152687 | 1196.20155285 |
| 750.1875468867216 | 1367.70274907 | 750.1875468867216 | 112.685219944 | 750.1875468867216 | 1115.05514769 |
| 747.011952191235 | 1367.68258578 | 747.011952191235 | 125.73253996 | 747.011952191235 | 1038.30574665 |
| 743.86312918423 | 1366.54253568 | 743.86312918423 | 140.012811715 | 743.86312918423 | 965.859361331 |
| 740.7407407407408 | 1364.32275895 | 740.7407407407408 | 155.606385576 | 740.7407407407408 | 897.605543217 |
| 737.6444553725104 | 1361.06372875 | 737.6444553725104 | 172.594445469 | 737.6444553725104 | 833.419738494 |
| 734.5739471106758 | 1356.80579856 | 734.5739471106758 | 191.058431898 | 734.5739471106758 | 773.165575699 |
| 731.528895391368 | 1351.58882409 | 731.528895391368 | 211.079400273 | 731.528895391368 | 716.697064502 |
| 728.5089849441475 | 1345.45184456 | 728.5089849441475 | 232.737315708 | 728.5089849441475 | 663.860686918 |
| 725.5139056831922 | 1338.43282644 | 725.5139056831922 | 256.110286585 | 725.5139056831922 | 614.497365401 |
| 722.543352601156 | 1330.56847147 | 722.543352601156 | 281.273740273 | 722.543352601156 | 568.444295409 |
| 719.5970256656271 | 1321.89408891 | 719.5970256656271 | 308.299545669 | 719.5970256656271 | 525.536633031 |
| 716.6746297181079 | 1312.44353089 | 716.6746297181079 | 337.255088443 | 716.6746297181079 | 485.609031202 |
| 713.7758743754462 | 1302.24918774 | 713.7758743754462 | 368.202306212 | 713.7758743754462 | 448.49702078 |
| 710.9004739336492 | 1291.34203949 | 710.9004739336492 | 401.196692133 | 710.9004739336492 | 414.038235312 |
| 708.0481472740146 | 1279.7517578 | 708.0481472740146 | 436.286276707 | 708.0481472740146 | 382.073480646 |
| 705.2186177715091 | 1267.50685211 | 705.2186177715091 | 473.510598842 | 705.2186177715091 | 352.447652632 |
| 702.4116132053383 | 1254.63485256 | 702.4116132053383 | 512.899678381 | 702.4116132053383 | 325.01050799 |
| 699.6268656716418 | 1241.16252173 | 699.6268656716418 | 554.473003367 | 699.6268656716418 | 299.617294985 |
| 696.8641114982578 | 1227.11608651 | 696.8641114982578 | 598.238546295 | 696.8641114982578 | 276.129251841 |
| 694.1230911614992 | 1212.52148159 | 694.1230911614992 | 644.191824345 | 694.1230911614992 | 254.413981877 |
| 691.4035492048858 | 1197.40459559 | 691.4035492048858 | 692.315019239 | 691.4035492048858 | 234.345715113 |
| 688.7052341597796 | 1181.79151123 | 688.7052341597796 | 742.576172694 | 688.7052341597796 | 215.80546664 |
| 686.027898467871 | 1165.70873141 | 686.027898467871 | 794.928473652 | 686.027898467871 | 198.681102367 |
| 683.371298405467 | 1149.18338349 | 683.371298405467 | 849.309653292 | 683.371298405467 | 182.867322856 |
| 680.7351940095303 | 1132.24339495 | 680.7351940095303 | 905.641503484 | 680.7351940095303 | 168.265575874 |
| 678.1193490054249 | 1114.91763453 | 678.1193490054249 | 963.829533617 | 678.1193490054249 | 154.783908058 |
| 675.5235307363206 | 1097.23601387 | 675.5235307363206 | 1023.76277976 | 675.5235307363206 | 142.336765686 |
| 672.9475100942127 | 1079.22954618 | 672.9475100942127 | 1085.31377881 | 672.9475100942127 | 130.844754055 |
| 670.3910614525139 | 1060.93035922 | 670.3910614525139 | 1148.33871863 | 670.3910614525139 | 120.234364348 |
| 667.8539626001781 | 1042.37166154 | 667.8539626001781 | 1212.67777342 | 667.8539626001781 | 110.437676234 |
| 665.335994677312 | 1023.58766211 | 665.335994677312 | 1278.15563109 | 665.335994677312 | 101.39204365 |
| 662.8369421122403 | 1004.61344447 | 662.8369421122403 | 1344.58221729 | 662.8369421122403 | 93.0397705409 |
| 660.3565925599823 | 985.484798202 | 660.3565925599823 | 1411.75361792 | 660.3565925599823 | 85.327782476 |
| 657.8947368421053 | 966.238011198 | 657.8947368421053 | 1479.45319898 | 657.8947368421053 | 78.2072993354 |
| 655.4511688879178 | 946.909627419 | 655.4511688879178 | 1547.45291975 | 655.4511688879178 | 71.633513487 |
| 653.0256856769699 | 927.536175697 | 653.0256856769699 | 1615.51483218 | 653.0256856769699 | 65.5652771352 |
| 650.6180871828237 | 908.153875802 | 650.6180871828237 | 1683.39275601 | 650.6180871828237 | 59.9648018361 |
| 648.2281763180639 | 888.798328561 | 648.2281763180639 | 1750.83411623 | 648.2281763180639 | 54.7973725197 |
| 645.8557588805166 | 869.504197204 | 645.8557588805166 | 1817.58192607 | 645.8557588805166 | 50.0310777622 |
| 643.5006435006435 | 850.304887299 | 643.5006435006435 | 1883.37689608 | 643.5006435006435 | 45.6365575099 |
| 641.1626415900834 | 831.232232647 | 641.1626415900834 | 1947.95964678 | 641.1626415900834 | 41.5867689689 |
| 638.8415672913118 | 812.316194327 | 638.8415672913118 | 2011.07299991 | 638.8415672913118 | 37.8567709517 |
| 636.5372374283895 | 793.584579781 | 636.5372374283895 | 2072.46432117 | 636.5372374283895 | 34.4235266071 |
| 634.2494714587738 | 775.062788278 | 634.2494714587738 | 2131.88788536 | 634.2494714587738 | 31.2657241468 |
| 631.9780914261638 | 756.77358851 | 631.9780914261638 | 2189.10723336 | 631.9780914261638 | 28.3636149357 |
| 629.7229219143577 | 738.736933257 | 629.7229219143577 | 2243.89748943 | 629.7229219143577 | 25.6988681049 |
| 627.4837900020916 | 720.969815246 | 627.4837900020916 | 2296.04760686 | 627.4837900020916 | 23.2544407011 |
| 625.2605252188412 | 703.486167326 | 625.2605252188412 | 2345.36250994 | 625.2605252188412 | 21.0144622705 |
| 623.0529595015576 | 686.296809127 | 623.0529595015576 | 2391.66510077 | 623.0529595015576 | 18.964132712 |
| 620.8609271523178 | 669.409441313 | 620.8609271523178 | 2434.79810066 | 620.8609271523178 | 17.0896321969 |
| 618.6842647968654 | 652.828687498 | 618.6842647968654 | 2474.62569732 | 618.6842647968654 | 15.3780419481 |
| 616.5228113440197 | 636.556182915 | 616.5228113440197 | 2511.03497159 | 616.5228113440197 | 13.8172746937 |
| 614.3764079459348 | 620.590707892 | 614.3764079459348 | 2543.93707976 | 614.3764079459348 | 12.3960136519 |
| 612.2448979591836 | 604.928363339 | 612.2448979591836 | 2573.26817105 | 612.2448979591836 | 11.103658959 |
| 610.1281269066504 | 589.562784585 | 610.1281269066504 | 2598.99002324 | 610.1281269066504 | 9.93028052846 |
| 608.0259424402108 | 574.485389189 | 608.0259424402108 | 2621.09038362 | 608.0259424402108 | 8.8665764027 |
| 605.9381943041809 | 559.685653733 | 605.9381943041809 | 2639.58300651 | 605.9381943041809 | 7.9038357498 |
| 603.864734299517 | 545.151414119 | 603.864734299517 | 2654.50738342 | 603.864734299517 | 7.03390574155 |
| 601.8054162487462 | 530.869183526 | 601.8054162487462 | 2665.92816637 | 601.8054162487462 | 6.24916164053 |
| 599.7600959616153 | 516.824481961 | 599.7600959616153 | 2673.9342899 | 599.7600959616153 | 5.54247950942 |
| 597.7286312014345 | 503.002171263 | 597.7286312014345 | 2678.63780192 | 597.7286312014345 | 4.90721104002 |
| 595.7108816521048 | 489.386789429 | 595.7108816521048 | 2680.17241844 | 595.7108816521048 | 4.33716007814 |
| 593.7067088858104 | 475.962878349 | 593.7067088858104 | 2678.69182133 | 593.7067088858104 | 3.82656049386 |
| 591.7159763313609 | 462.71529929 | 591.7159763313609 | 2674.36772309 | 591.7159763313609 | 3.37005511371 |
| 589.7385492431688 | 449.629530876 | 589.7385492431688 | 2667.38772582 | 589.7385492431688 | 2.96267549131 |
| 587.7742946708463 | 436.691944831 | 587.7742946708463 | 2657.95300565 | 587.7742946708463 | 2.59982234623 |
| 585.8230814294083 | 423.890055281 | 585.8230814294083 | 2646.27585632 | 585.8230814294083 | 2.27724654689 |
| 583.8847800700661 | 411.212738093 | 583.8847800700661 | 2632.57712843 | 583.8847800700661 | 1.99103055296 |
| 581.9592628516003 | 398.650417386 | 581.9592628516003 | 2617.08360234 | 581.9592628516003 | 1.73757026506 |
| 580.046403712297 | 386.195217101 | 580.046403712297 | 2600.02533406 | 580.046403712297 | 1.5135572568 |
| 578.1460782424359 | 373.841076204 | 578.1460782424359 | 2581.63301393 | 578.1460782424359 | 1.3159613849 |
| 576.2581636573184 | 361.583826899 | 576.2581636573184 | 2562.1353775 | 576.2581636573184 | 1.1420137895 |
| 574.3825387708214 | 349.421235855 | 574.3825387708214 | 2541.75670736 | 574.3825387708214 | 0.989190307874 |
| 572.5190839694656 | 337.353009231 | 572.5190839694656 | 2520.71446295 | 572.5190839694656 | 0.855195332904 |
| 570.6676811869887 | 325.380762838 | 570.6676811869887 | 2499.21707323 | 570.6676811869887 | 0.737946151265 |
| 568.8282138794084 | 313.507959422 | 568.8282138794084 | 2477.46192425 | 568.8282138794084 | 0.635557798155 |
| 567.000567000567 | 301.739815532 | 567.000567000567 | 2455.63357017 | 567.000567000567 | 0.54632846428 |
| 565.1846269781461 | 290.083180901 | 565.1846269781461 | 2433.90219262 | 565.1846269781461 | 0.468725488159 |
| 563.3802816901408 | 278.546393635 | 563.3802816901408 | 2412.4223287 | 563.3802816901408 | 0.401371962797 |
| 561.5874204417821 | 267.139114766 | 561.5874204417821 | 2391.3318833 | 561.5874204417821 | 0.343033980706 |
| 559.8059339428997 | 255.872145958 | 559.8059339428997 | 2370.75143676 | 559.8059339428997 | 0.292608535645 |
| 558.0357142857143 | 244.757234248 | 558.0357142857143 | 2350.78385316 | 558.0357142857143 | 0.24911209348 |
| 556.2766549230483 | 233.806867745 | 556.2766549230483 | 2331.51418994 | 556.2766549230483 | 0.211669838519 |
| 554.52865064695 | 223.034066167 | 554.52865064695 | 2313.00990395 | 554.52865064695 | 0.179505595808 |
| 552.791597567717 | 212.452169999 | 552.791597567717 | 2295.32134399 | 552.791597567717 | 0.151932424227 |
| 551.0653930933137 | 202.074631841 | 551.0653930933137 | 2278.48251497 | 551.0653930933137 | 0.128343870102 |
| 549.3499359091741 | 191.914813311 | 549.3499359091741 | 2262.51209434 | 549.3499359091741 | 0.108205866334 |
| 547.645125958379 | 181.985790564 | 547.645125958379 | 2247.41467692 | 547.645125958379 | 0.0910492579788 |
| 545.950864422202 | 172.300171175 | 545.950864422202 | 2233.18222054 | 545.950864422202 | 0.0764629316956 |
| 544.2670537010159 | 162.869924742 | 544.2670537010159 | 2219.79566163 | 544.2670537010159 | 0.0640875236314 |
| 542.5935973955508 | 153.706229242 | 542.5935973955508 | 2207.22666705 | 542.5935973955508 | 0.0536096779912 |
| 540.9304002884962 | 144.819334715 | 540.9304002884962 | 2195.43948631 | 540.9304002884962 | 0.0447568268739 |
| 539.2773683264425 | 136.218445511 | 539.2773683264425 | 2184.39286692 | 539.2773683264425 | 0.0372924607863 |
| 537.6344086021505 | 127.911621891 | 537.6344086021505 | 2174.0419947 | 537.6344086021505 | 0.0310118585945 |
| 536.0014293371448 | 119.905701433 | 536.0014293371448 | 2164.34042079 | 536.0014293371448 | 0.0257382454694 |
| 534.3783398646241 | 112.206240308 | 534.3783398646241 | 2155.24193768 | 534.3783398646241 | 0.0213193475778 |
| 532.7650506126798 | 104.817474144 | 532.7650506126798 | 2146.70236784 | 532.7650506126798 | 0.0176243128107 |
| 531.1614730878186 | 97.74229792 | 531.1614730878186 | 2138.68123027 | 531.1614730878186 | 0.0145409676694 |
| 529.5675198587819 | 90.9822640095 | 529.5675198587819 | 2131.14325298 | 529.5675198587819 | 0.0119733815041 |
| 527.9831045406547 | 84.5375972907 | 527.9831045406547 | 2124.05970239 | 527.9831045406547 | 0.00983971055554 |
| 526.4081417792595 | 78.4072260152 | 526.4081417792595 | 2117.40950425 | 526.4081417792595 | 0.00807029565855 |
| 524.8425472358292 | 72.5888269704 | 524.8425472358292 | 2111.1801346 | 524.8425472358292 | 0.00660598897285 |
| 523.2862375719518 | 67.0788833449 | 523.2862375719518 | 2105.36826386 | 523.2862375719518 | 0.00539668668012 |
| 521.7391304347826 | 61.8727536193 | 521.7391304347826 | 2099.98014176 | 521.7391304347826 | 0.00440004619306 |
| 520.2011444425177 | 56.9647497543 | 520.2011444425177 | 2095.03171564 | 520.2011444425177 | 0.00358036803327 |
| 518.6721991701245 | 52.3482229336 | 518.6721991701245 | 2090.54847978 | 518.6721991701245 | 0.00290762412638 |
| 517.1522151353215 | 48.0156551347 | 517.1522151353215 | 2086.5650584 | 517.1522151353215 | 0.00235661581576 |
| 515.6411137848057 | 43.9587548478 | 515.6411137848057 | 2083.12453007 | 515.6411137848057 | 0.0019062463935 |
| 514.1388174807198 | 40.1685553333 | 514.1388174807198 | 2080.27750596 | 514.1388174807198 | 0.00153889437786 |
| 512.6452494873547 | 36.6355139016 | 512.6452494873547 | 2078.08097908 | 512.6452494873547 | 0.00123987512004 |
| 511.1603339580848 | 33.3496108103 | 511.1603339580848 | 2076.59696599 | 511.1603339580848 | 0.000996979594001 |
| 509.683995922528 | 30.3004465001 | 509.683995922528 | 2075.89096628 | 509.683995922528 | 0.00080008040746 |
| 508.2161612739285 | 27.4773360254 | 508.2161612739285 | 2076.03026864 | 508.2161612739285 | 0.000640796167856 |
| 506.7567567567567 | 24.8693996812 | 506.7567567567567 | 2077.08213535 | 506.7567567567567 | 0.000512206344812 |
| 505.3057099545225 | 22.4656489721 | 505.3057099545225 | 2079.11189923 | 505.3057099545225 | 0.000408609691125 |
| 503.8629492777964 | 20.2550672193 | 503.8629492777964 | 2082.18100901 | 503.8629492777964 | 0.000325320120905 |
| 502.4284039524367 | 18.2266842452 | 502.4284039524367 | 2086.3450603 | 502.4284039524367 | 0.000258494699358 |
| 501.00200400801606 | 16.3696447158 | 501.00200400801606 | 2091.65184942 | 501.00200400801606 | 0.000204989078216 |
| 499.5836802664446 | 14.6732698558 | 499.5836802664446 | 2098.13948782 | 499.5836802664446 | 0.00016223631863 |
| 498.1733643307871 | 13.127112375 | 498.1733643307871 | 2105.83461324 | 498.1733643307871 | 0.000128145584501 |
| 496.7709885742673 | 11.7210045599 | 496.7709885742673 | 2114.75073303 | 496.7709885742673 | 0.0001010176687 |
| 495.3764861294584 | 10.4450995888 | 495.3764861294584 | 2124.88673222 | 495.3764861294584 | 7.94747377447e-05 |
| 493.98979087765514 | 9.28990622016 | 493.98979087765514 | 2136.2255769 | 493.98979087765514 | 6.24020521286e-05 |
| 492.61083743842363 | 8.24631708256 | 492.61083743842363 | 2148.73323987 | 492.61083743842363 | 4.88997446978e-05 |
| 491.2395611593253 | 7.305630865 | 491.2395611593253 | 2162.35787185 | 491.2395611593253 | 3.82430227529e-05 |
| 489.8758981058131 | 6.45956875775 | 489.8758981058131 | 2177.02923773 | 489.8758981058131 | 2.98494054831e-05 |
| 488.5197850512946 | 5.70028554002 | 488.5197850512946 | 2192.65843263 | 488.5197850512946 | 2.32518209404e-05 |
| 487.17115946735953 | 5.02037574164 | 487.17115946735953 | 2209.13788791 | 487.17115946735953 | 1.80765699008e-05 |
| 485.82995951416996 | 4.41287532763 | 485.82995951416996 | 2226.34167255 | 485.82995951416996 | 1.40253211438e-05 |
| 484.49612403100775 | 3.87125936661 | 484.49612403100775 | 2244.12609014 | 484.49612403100775 | 1.0860437093e-05 |
| 483.16959252697694 | 3.38943614691 | 483.16959252697694 | 2262.33056694 | 483.16959252697694 | 8.39304331228e-06 |
| 481.8503051718599 | 2.96173819984 | 481.8503051718599 | 2280.77882149 | 481.8503051718599 | 6.47335263188e-06 |
| 480.5382027871216 | 2.58291067804 | 480.5382027871216 | 2299.28030152 | 480.5382027871216 | 4.98283701636e-06 |
| 479.23322683706067 | 2.24809751997 | 479.23322683706067 | 2317.63186949 | 479.23322683706067 | 3.82790974732e-06 |
| 477.9353194201051 | 1.95282580972 | 477.9353194201051 | 2335.61971392 | 477.9353194201051 | 2.93483889524e-06 |
| 476.64442326024783 | 1.69298871612 | 476.64442326024783 | 2353.02145987 | 476.64442326024783 | 2.24566200508e-06 |
| 475.3604816986214 | 1.4648273667 | 475.3604816986214 | 2369.60844867 | 475.3604816986214 | 1.71491281557e-06 |
| 474.08343868520853 | 1.26491198214 | 474.08343868520853 | 2385.14815425 | 474.08343868520853 | 1.30700489175e-06 |
| 472.8132387706856 | 1.09012256534 | 472.8132387706856 | 2399.40670113 | 472.8132387706856 | 9.9414532999e-07 |
| 471.5498270983967 | 0.937629407826 | 471.5498270983967 | 2412.15144754 | 471.5498270983967 | 7.54675102451e-07 |
| 470.29314939645707 | 0.804873644294 | 470.29314939645707 | 2423.15359611 | 470.29314939645707 | 5.71751926275e-07 |
| 469.04315196998124 | 0.689548055467 | 469.04315196998124 | 2432.19079424 | 469.04315196998124 | 4.32307436784e-07 |
| 467.7997816934352 | 0.589578289612 | 467.7997816934352 | 2439.04968644 | 467.7997816934352 | 3.26223483793e-07 |
| 466.5629860031104 | 0.503104644757 | 466.5629860031104 | 2443.52838189 | 466.5629860031104 | 2.4568303599e-07 |
| 465.33271288971605 | 0.428464527182 | 465.33271288971605 | 2445.43880203 | 465.33271288971605 | 1.84659877684e-07 |
| 464.10891089108907 | 0.364175677234 | 464.10891089108907 | 2444.60887468 | 464.10891089108907 | 1.38518357275e-07 |
| 462.8915290850177 | 0.308920231012 | 462.8915290850177 | 2440.88454441 | 462.8915290850177 | 1.03700184446e-07 |
| 461.68051708217905 | 0.26152966631 | 461.68051708217905 | 2434.13157131 | 461.68051708217905 | 7.74799131206e-08 |
| 460.47582501918646 | 0.220970663068 | 460.47582501918646 | 2424.23709425 | 460.47582501918646 | 5.77744891706e-08 |
| 459.2774035517452 | 0.186331892782 | 459.2774035517452 | 2411.11093826 | 459.2774035517452 | 4.29952511311e-08 |
| 458.0852038479157 | 0.156811737582 | 458.0852038479157 | 2394.68665005 | 458.0852038479157 | 3.19331856898e-08 |
| 456.89917758148033 | 0.131706927973 | 456.89917758148033 | 2374.92224975 | 456.89917758148033 | 2.36701700583e-08 |
| 455.7192769254139 | 0.110402078495 | 455.7192769254139 | 2351.80069152 | 455.7192769254139 | 1.75104731051e-08 |
| 454.54545454545456 | 0.0923600925583 | 454.54545454545456 | 2325.33003028 | 454.54545454545456 | 1.29280120433e-08 |
| 453.3776635937736 | 0.0771134014093 | 453.3776635937736 | 2295.54329592 | 453.3776635937736 | 9.52583330406e-09 |
| 452.2158577027434 | 0.0642559973139 | 452.2158577027434 | 2262.49808117 | 452.2158577027434 | 7.00505524065e-09 |
| 451.05999097880016 | 0.0534362175705 | 451.05999097880016 | 2226.27585289 | 451.05999097880016 | 5.14111721344e-09 |
| 449.9100179964007 | 0.0443502336362 | 449.9100179964007 | 2186.98100106 | 449.9100179964007 | 3.76565733254e-09 |
| 448.7658937920718 | 0.036736198361 | 448.7658937920718 | 2144.73964276 | 448.7658937920718 | 2.75271619101e-09 |
| 447.6275738585497 | 0.030369003918 | 447.6275738585497 | 2099.69820191 | 447.6275738585497 | 2.00825761371e-09 |
| 446.49501413900873 | 0.0250556033498 | 446.49501413900873 | 2052.02178843 | 446.49501413900873 | 1.46222683351e-09 |
| 445.36817102137763 | 0.0206308496008 | 445.36817102137763 | 2001.89240248 | 445.36817102137763 | 1.06254520765e-09 |
| 444.247001332741 | 0.0169538073524 | 444.247001332741 | 1949.50699155 | 444.247001332741 | 7.70579448709e-10 |
| 443.13146233382565 | 0.0139044948046 | 443.13146233382565 | 1895.0753894 | 443.13146233382565 | 5.57730973427e-10 |
| 442.02151171357 | 0.0113810146787 | 442.02151171357 | 1838.81816671 | 442.02151171357 | 4.02874184164e-10 |
| 440.9171075837742 | 0.00929703604333 | 440.9171075837742 | 1780.96442349 | 440.9171075837742 | 2.90436668843e-10 |
| 439.8182084738308 | 0.00757959103361 | 439.8182084738308 | 1721.74955341 | 439.8182084738308 | 2.08963669367e-10 |
| 438.72477332553376 | 0.00616715306989 | 438.72477332553376 | 1661.41300922 | 438.72477332553376 | 1.50047047472e-10 |
| 437.636761487965 | 0.00500796573556 | 437.636761487965 | 1600.19609756 | 437.636761487965 | 1.0752797089e-10 |
| 436.5541327124563 | 0.00405859400126 | 436.5541327124563 | 1538.33982992 | 436.5541327124563 | 7.69046807934e-11 |
| 435.4768471476266 | 0.00328267194793 | 435.4768471476266 | 1476.08285444 | 435.4768471476266 | 5.4893563309e-11 |
| 434.4048653344918 | 0.00264982351655 | 434.4048653344918 | 1413.65949147 | 434.4048653344918 | 3.91045594106e-11 |
| 433.3381482016467 | 0.00213473507658 | 433.3381482016467 | 1351.29789275 | 433.3381482016467 | 2.78016578934e-11 |
| 432.2766570605187 | 0.00171636074305 | 432.2766570605187 | 1289.21834208 | 432.2766570605187 | 1.97265580924e-11 |
| 431.22035360069 | 0.00137724337325 | 431.22035360069 | 1227.63171195 | 431.22035360069 | 1.39691267767e-11 |
| 430.1691998852882 | 0.00110293603327 | 430.1691998852882 | 1166.73808794 | 430.1691998852882 | 9.87244046176e-12 |
| 429.1231583464454 | 0.000881510438658 | 429.1231583464454 | 1106.72556991 | 429.1231583464454 | 6.96333210359e-12 |
| 428.0821917808219 | 0.00070314044432 | 428.0821917808219 | 1047.76925551 | 428.0821917808219 | 4.90170323356e-12 |
| 427.0462633451957 | 0.000559750088659 | 427.0462633451957 | 990.030409389 | 427.0462633451957 | 3.4436122098e-12 |
| 426.01533655211585 | 0.000444716991629 | 426.01533655211585 | 933.655817973 | 426.01533655211585 | 2.41445315161e-12 |
| 424.9893752656184 | 0.000352623071666 | 424.9893752656184 | 878.777327646 | 424.9893752656184 | 1.68950952948e-12 |
| 423.96834369700395 | 0.000279045590239 | 423.96834369700395 | 825.511561367 | 423.96834369700395 | 1.1798853981e-12 |
| 422.9522064006767 | 0.00022038246286 | 422.9522064006767 | 773.959806728 | 422.9522064006767 | 8.22349295966e-13 |
| 421.9409282700422 | 0.000173706600309 | 421.9409282700422 | 724.208066425 | 421.9409282700422 | 5.72018586876e-13 |
| 420.93447453346425 | 0.000136644772161 | 420.93447453346425 | 676.327260434 | 420.93447453346425 | 3.97101267759e-13 |
| 419.9328107502799 | 0.000107277124832 | 419.9328107502799 | 630.373567766 | 419.9328107502799 | 2.75124789347e-13 |
| 418.93590280687056 | 8.40540467252e-05 | 418.93590280687056 | 586.38889453 | 418.93590280687056 | 1.90237219147e-13 |
| 417.94371691278906 | 6.57275614168e-05 | 417.94371691278906 | 544.401454193 | 417.94371691278906 | 1.31280000842e-13 |
| 416.9562195969423 | 5.12948538865e-05 | 416.9562195969423 | 504.426445346 | 416.9562195969423 | 9.04146839704e-14 |
| 415.97337770382694 | 3.99519014585e-05 | 415.97337770382694 | 466.466811982 | 415.97337770382694 | 6.21465010333e-14 |
| 414.99515838981876 | 3.10554970659e-05 | 414.99515838981876 | 430.51407127 | 414.99515838981876 | 4.26316077059e-14 |
| 414.0215291195142 | 2.40922236123e-05 | 414.0215291195142 | 396.549193964 | 414.0215291195142 | 2.91866368796e-14 |
| 413.0524576621231 | 1.86531701272e-05 | 413.0524576621231 | 364.543523027 | 413.0524576621231 | 1.99422296894e-14 |
| 412.08791208791206 | 1.44133780626e-05 | 412.08791208791206 | 334.459716651 | 412.08791208791206 | 1.3598803176e-14 |
| 411.1278607646978 | 1.111517393e-05 | 411.1278607646978 | 306.252702584 | 411.1278607646978 | 9.25475607569e-15 |
| 410.17227235438884 | 8.55468655688e-06 | 410.17227235438884 | 279.870631647 | 410.17227235438884 | 6.28588715097e-15 |
| 409.22111580957574 | 6.5709670791e-06 | 409.22111580957574 | 255.25581928 | 409.22111580957574 | 4.26094077433e-15 |
| 408.2743603701687 | 5.03723055592e-06 | 408.2743603701687 | 232.345665115 | 408.2743603701687 | 2.88258239399e-15 |
| 407.33197556008145 | 3.85382253831e-06 | 407.33197556008145 | 211.073541707 | 407.33197556008145 | 1.94623498944e-15 |
| 406.39393118396094 | 2.94258446843e-06 | 406.39393118396094 | 191.36964477 | 406.39393118396094 | 1.31143308668e-15 |
| 405.46019732396263 | 2.24235053729e-06 | 405.46019732396263 | 173.161798478 | 405.46019732396263 | 8.81930395922e-16 |
| 404.53074433656957 | 1.7053574004e-06 | 404.53074433656957 | 156.376210562 | 404.53074433656957 | 5.91915618151e-16 |
| 403.6055428494551 | 1.29438851807e-06 | 403.6055428494551 | 140.938173157 | 403.6055428494551 | 3.96481202237e-16 |
| 402.68456375838923 | 9.80508196069e-07 | 402.68456375838923 | 126.772706403 | 402.68456375838923 | 2.65046893047e-16 |
| 401.76777822418643 | 7.41267804454e-07 | 401.76777822418643 | 113.805142924 | 401.76777822418643 | 1.76831710333e-16 |
| 400.85515766969536 | 5.59289130101e-07 | 400.85515766969536 | 101.961652252 | 400.85515766969536 | 1.17742944115e-16 |
| 399.9466737768297 | 4.21148201065e-07 | 399.9466737768297 | 91.1697051698 | 399.9466737768297 | 7.82432619947e-17 |
| 399.0422984836393 | 3.16497912465e-07 | 399.0422984836393 | 81.3584787665 | 399.0422984836393 | 5.18915112261e-17 |
| 398.14200398142003 | 2.373799737e-07 | 398.14200398142003 | 72.4592036938 | 398.14200398142003 | 3.43465407338e-17 |
| 397.24576271186436 | 1.7768658121e-07 | 397.24576271186436 | 64.4054557369 | 397.24576271186436 | 2.26885635368e-17 |
| 396.3535473642489 | 1.32740213181e-07 | 396.3535473642489 | 57.1333943247 | 396.3535473642489 | 1.49578203108e-17 |
| 395.46533087266016 | 9.89663867866e-08 | 395.46533087266016 | 50.581951028 | 395.46533087266016 | 9.84162779475e-18 |
| 394.5810864132579 | 7.36394001484e-08 | 394.5810864132579 | 44.6929714193 | 394.5810864132579 | 6.46253449273e-18 |
| 393.7007874015748 | 5.46852362204e-08 | 393.7007874015748 | 39.4113139115 | 393.7007874015748 | 4.23522146721e-18 |
| 392.82440748985204 | 4.05291284603e-08 | 392.82440748985204 | 34.6849093487 | 392.82440748985204 | 2.77004414187e-18 |
| 391.9519205644107 | 2.99779380929e-08 | 391.9519205644107 | 30.4647852064 | 391.9519205644107 | 1.80815046574e-18 |
| 391.08330074305826 | 2.21296004474e-08 | 391.08330074305826 | 26.7050582726 | 391.08330074305826 | 1.17793074834e-18 |
| 390.2185223725286 | 1.63035698026e-08 | 390.2185223725286 | 23.3628996384 | 390.2185223725286 | 7.65847436315e-19 |
| 389.3575600259571 | 1.19875149344e-08 | 389.3575600259571 | 20.3984757282 | 389.3575600259571 | 4.96937854222e-19 |
| 388.5003885003885 | 8.79656131681e-09 | 388.5003885003885 | 17.7748689572 | 388.5003885003885 | 3.21809759698e-19 |
| 387.6469828143171 | 6.44219736677e-09 | 387.6469828143171 | 15.4579814296 | 387.6469828143171 | 2.07985785582e-19 |
| 386.7973182052604 | 4.70860691196e-09 | 386.7973182052604 | 13.4164248805 | 386.7973182052604 | 1.34154551565e-19 |
| 385.95137012736393 | 3.4346948855e-09 | 385.95137012736393 | 11.6213998405 | 385.95137012736393 | 8.6360367556e-20 |
| 385.1091142490372 | 2.50046746681e-09 | 385.1091142490372 | 10.0465667535 | 385.1091142490372 | 5.54831187772e-20 |
| 384.2705264506212 | 1.81673494701e-09 | 384.2705264506212 | 8.66791153229 | 384.2705264506212 | 3.5574971574e-20 |
| 383.4355828220859 | 1.31734415617e-09 | 383.4355828220859 | 7.46360777359 | 383.4355828220859 | 2.27648907918e-20 |
| 382.6042596607575 | 9.53332161267e-10 | 382.6042596607575 | 6.41387760582 | 382.6042596607575 | 1.45386437155e-20 |
| 381.77653346907607 | 6.88535847326e-10 | 381.77653346907607 | 5.50085288825 | 381.77653346907607 | 9.26658106894e-21 |
| 380.95238095238096 | 4.96302180521e-10 | 380.95238095238096 | 4.70843824224 | 380.95238095238096 | 5.89457473466e-21 |
| 380.1317790167258 | 3.57028698199e-10 | 380.1317790167258 | 4.02217716458 | 380.1317790167258 | 3.74216334194e-21 |
| 379.31470476672143 | 2.56328788555e-10 | 379.31470476672143 | 3.4291222576 | 379.31470476672143 | 2.370993236e-21 |
| 378.5011355034065 | 1.83666080845e-10 | 378.5011355034065 | 2.91771041036 | 378.5011355034065 | 1.4992539543e-21 |
| 377.69104872214524 | 1.31340252826e-10 | 377.69104872214524 | 2.4776435812 | 377.69104872214524 | 9.46144358027e-22 |
| 376.88442211055275 | 9.37354895644e-11 | 376.88442211055275 | 2.09977566547 | 376.88442211055275 | 5.959048471e-22 |
| 376.081233546446 | 6.67648028341e-11 | 376.081233546446 | 1.77600578275 | 376.081233546446 | 3.7457065038e-22 |
| 375.28146109582184 | 4.74600732653e-11 | 375.28146109582184 | 1.49917818594 | 375.28146109582184 | 2.34978369088e-22 |
| 374.48508301086 | 3.36702663854e-11 | 374.48508301086 | 1.26298887947 | 374.48508301086 | 1.47115806959e-22 |
| 373.69207772795215 | 2.3839767459e-11 | 373.69207772795215 | 1.06189893493 | 373.69207772795215 | 9.19238286344e-23 |
| 372.9024238657551 | 1.68459222379e-11 | 372.9024238657551 | 0.8910544088 | 372.9024238657551 | 5.73236959268e-23 |
| 372.11610022326965 | 1.18802309299e-11 | 372.11610022326965 | 0.746212698143 | 372.11610022326965 | 3.56761166947e-23 |
| 371.33308577794276 | 8.36165582859e-12 | 371.33308577794276 | 0.623675113592 | 371.33308577794276 | 2.215941449e-23 |
| 370.55335968379444 | 5.87350045682e-12 | 370.55335968379444 | 0.520225405766 | 370.55335968379444 | 1.37365066018e-23 |
| 369.7769012695673 | 4.11755150158e-12 | 369.7769012695673 | 0.433073947832 | 369.7769012695673 | 8.49829163054e-24 |
| 369.0036900369003 | 2.88083501433e-12 | 369.0036900369003 | 0.359807253826 | 369.0036900369003 | 5.24715960646e-24 |
| 368.23370565852457 | 2.0115695258e-12 | 368.23370565852457 | 0.298342497445 | 368.23370565852457 | 3.23336126942e-24 |
| 367.4669279764821 | 1.40180957527e-12 | 367.4669279764821 | 0.246886688449 | 367.4669279764821 | 1.9884812583e-24 |
| 366.7033370003667 | 9.74945435534e-13 | 366.7033370003667 | 0.203900162565 | 366.7033370003667 | 1.22046712412e-24 |
| 365.9429129055867 | 6.76719844512e-13 | 365.9429129055867 | 0.168064044571 | 365.9429129055867 | 7.47597742821e-25 |
| 365.1856360316494 | 4.6878620971e-13 | 365.1856360316494 | 0.138251352449 | 365.1856360316494 | 4.57032611988e-25 |
| 364.4314868804664 | 3.24099272716e-13 | 364.4314868804664 | 0.11350142196 | 364.4314868804664 | 2.78845546612e-25 |
| 363.68044611468054 | 2.23624096479e-13 | 363.68044611468054 | 0.0929973451491 | 363.68044611468054 | 1.69792126695e-25 |
| 362.93249455601256 | 1.53991395524e-13 | 362.93249455601256 | 0.0760461323739 | 362.93249455601256 | 1.03183129616e-25 |
| 362.1876131836291 | 1.05830689104e-13 | 362.1876131836291 | 0.0620613248299 | 362.1876131836291 | 6.25802303422e-26 |
| 361.4457831325301 | 7.25878790048e-14 | 361.4457831325301 | 0.0505478027775 | 361.4457831325301 | 3.78793860628e-26 |
| 360.7069856919562 | 4.96882730301e-14 | 360.7069856919562 | 0.0410885532608 | 360.7069856919562 | 2.2882634229e-26 |
| 359.97120230381563 | 3.39454063575e-14 | 359.97120230381563 | 0.0333331797044 | 359.97120230381563 | 1.37957850385e-26 |
| 359.2384145611304 | 2.31443737813e-14 | 359.2384145611304 | 0.0269879540738 | 359.2384145611304 | 8.30087992708e-27 |
| 358.50860420650093 | 1.57487892174e-14 | 358.50860420650093 | 0.0218072300508 | 358.50860420650093 | 4.98470151607e-27 |
| 357.7817531305903 | 1.06951337363e-14 | 357.7817531305903 | 0.0175860527165 | 357.7817531305903 | 2.98738706983e-27 |
| 357.057843370626 | 7.24874098894e-15 | 357.057843370626 | 0.0141538164174 | 357.057843370626 | 1.78682141551e-27 |
| 356.33685710892024 | 4.90316216812e-15 | 356.33685710892024 | 0.011368837712 | 356.33685710892024 | 1.06661604876e-27 |
| 355.6187766714082 | 3.30999430198e-15 | 355.6187766714082 | 0.00911372450392 | 355.6187766714082 | 6.35436843151e-28 |
| 354.9035845262037 | 2.23005486571e-15 | 354.9035845262037 | 0.00729143561589 | 354.9035845262037 | 3.77810464687e-28 |
| 354.1912632821723 | 1.49948167659e-15 | 354.1912632821723 | 0.00582193715905 | 354.1912632821723 | 2.24188270642e-28 |
| 353.48179568752204 | 1.0062458247e-15 | 353.48179568752204 | 0.00463937310143 | 353.48179568752204 | 1.32766682607e-28 |
| 352.77516462841015 | 6.73913773581e-16 | 352.77516462841015 | 0.00368967747554 | 352.77516462841015 | 7.84698159073e-29 |
| 352.07135312756714 | 4.50445120887e-16 | 352.07135312756714 | 0.00292856472836 | 352.07135312756714 | 4.6286407726e-29 |
| 351.3703443429374 | 3.0048081924e-16 | 351.3703443429374 | 0.00231984285535 | 351.3703443429374 | 2.7248439307e-29 |
| 350.6721215663355 | 2.00045568582e-16 | 350.6721215663355 | 0.00183400123383 | 350.6721215663355 | 1.60091067792e-29 |
| 349.9766682221185 | 1.32916356852e-16 | 349.9766682221185 | 0.00144703153721 | 349.9766682221185 | 9.38706625442e-30 |
| 349.2839678658749 | 8.81384149132e-17 | 349.2839678658749 | 0.00113944583554 | 349.2839678658749 | 5.4932577607e-30 |
| 348.59400418312805 | 5.83296480181e-17 | 348.59400418312805 | 0.000895461030143 | 348.59400418312805 | 3.20824396295e-30 |
| 347.90676098805517 | 3.85257221984e-17 | 347.90676098805517 | 0.00070232319371 | 347.90676098805517 | 1.87000215258e-30 |
| 347.2222222222222 | 2.53950770419e-17 | 347.2222222222222 | 0.000549749251573 | 347.2222222222222 | 1.08781273849e-30 |
| 346.54037195333257 | 1.67065045668e-17 | 346.54037195333257 | 0.000429466801527 | 346.54037195333257 | 6.31543815911e-31 |
| 345.8611943739912 | 1.09687961614e-17 | 345.8611943739912 | 0.000334835783065 | 345.8611943739912 | 3.65923369661e-31 |
| 345.1846738004832 | 7.1873641815e-18 | 345.1846738004832 | 0.000260538221672 | 345.1846738004832 | 2.1159924764e-31 |
| 344.5107946715664 | 4.70021418833e-18 | 344.5107946715664 | 0.000202324436657 | 344.5107946715664 | 1.2211679704e-31 |
| 343.8395415472779 | 3.06762988715e-18 | 343.8395415472779 | 0.000156805954213 | 343.8395415472779 | 7.03354067633e-32 |
| 343.17089910775564 | 1.99813848489e-18 | 343.17089910775564 | 0.00012128694965 | 343.17089910775564 | 4.04305747307e-32 |
| 342.50485215207215 | 1.29892932539e-18 | 342.50485215207215 | 9.36273889426e-05 | 342.50485215207215 | 2.31943998394e-32 |
| 341.84138559708293 | 8.42718973592e-19 | 341.84138559708293 | 7.21321811119e-05 | 341.84138559708293 | 1.32798655176e-32 |
| 341.1804844762879 | 5.45653990089e-19 | 341.1804844762879 | 5.54616174388e-05 | 341.1804844762879 | 7.58824819969e-33 |
| 340.522133938706 | 3.52605605052e-19 | 340.522133938706 | 4.25591858249e-05 | 340.522133938706 | 4.32739651518e-33 |
| 339.86631924776253 | 2.27404157767e-19 | 339.86631924776253 | 3.25935304888e-05 | 339.86631924776253 | 2.46291354651e-33 |
| 339.2130257801899 | 1.46367583637e-19 | 339.2130257801899 | 2.49118977715e-05 | 339.2130257801899 | 1.3989717892e-33 |
| 338.56223902494077 | 9.40218340317e-20 | 338.56223902494077 | 1.90028847557e-05 | 338.56223902494077 | 7.93060024785e-34 |
| 337.91394458211306 | 6.02767526056e-20 | 337.91394458211306 | 1.44667031373e-05 | 337.91394458211306 | 4.48683888092e-34 |
| 337.2681281618887 | 3.85663277807e-20 | 337.2681281618887 | 1.09914988372e-05 | 337.2681281618887 | 2.53344921511e-34 |
| 336.6247755834829 | 2.4626576523e-20 | 336.6247755834829 | 8.33453894825e-06 | 336.6247755834829 | 1.42764833861e-34 |
| 335.9838727741068 | 1.56941250829e-20 | 335.9838727741068 | 6.30730098444e-06 | 335.9838727741068 | 8.02911352554e-35 |
| 335.3454057679409 | 9.98176842053e-21 | 335.3454057679409 | 4.76368290602e-06 | 335.3454057679409 | 4.50662338369e-35 |
| 334.709360705121 | 6.33600021291e-21 | 334.709360705121 | 3.5907026926e-06 | 334.709360705121 | 2.52448181164e-35 |
| 334.07572383073494 | 4.01384122751e-21 | 334.07572383073494 | 2.70117898981e-06 | 334.07572383073494 | 1.41133638899e-35 |
| 333.44448149383123 | 2.53771302467e-21 | 333.44448149383123 | 2.02798431647e-06 | 333.44448149383123 | 7.87455726183e-36 |
| 332.81562014643885 | 1.60126105428e-21 | 332.81562014643885 | 1.51954348996e-06 | 332.81562014643885 | 4.38489365008e-36 |
| 332.1891263425977 | 1.00836807198e-21 | 332.1891263425977 | 1.13631565444e-06 | 332.1891263425977 | 2.43685280351e-36 |
| 331.5649867374005 | 6.33743246551e-22 | 331.5649867374005 | 8.48051366873e-07 | 331.5649867374005 | 1.35156470437e-36 |
| 330.9431880860452 | 3.97507123236e-22 | 330.9431880860452 | 6.31658930105e-07 | 330.9431880860452 | 7.48137975116e-37 |
| 330.323717242898 | 2.48836337008e-22 | 330.323717242898 | 4.69548476154e-07 | 330.323717242898 | 4.13298539818e-37 |
| 329.70656116056705 | 1.55460477332e-22 | 329.70656116056705 | 3.48349782477e-07 | 329.70656116056705 | 2.27868007556e-37 |
| 329.0917068889864 | 9.69311816274e-23 | 329.0917068889864 | 2.57921748946e-07 | 329.0917068889864 | 1.25383431638e-37 |
| 328.47914157451 | 6.03176384997e-23 | 328.47914157451 | 1.90588942151e-07 | 328.47914157451 | 6.88548064612e-38 |
| 327.86885245901635 | 3.7459542262e-23 | 327.86885245901635 | 1.40554497602e-07 | 327.86885245901635 | 3.77368534123e-38 |
| 327.26082687902255 | 2.32176315691e-23 | 327.26082687902255 | 1.03449669578e-07 | 327.26082687902255 | 2.06411752309e-38 |
| 326.6550522648083 | 1.43618596178e-23 | 326.6550522648083 | 7.5989009155e-08 | 326.6550522648083 | 1.12678347626e-38 |
| 326.05151613955 | 8.86626591402e-24 | 326.05151613955 | 5.57069996877e-08 | 326.05151613955 | 6.13880490565e-39 |
| 325.4502061184639 | 5.46270993382e-24 | 325.4502061184639 | 4.07573621589e-08 | 325.4502061184639 | 3.33783224886e-39 |
| 324.8511099079588 | 3.35902194667e-24 | 324.8511099079588 | 2.97604628594e-08 | 324.8511099079588 | 1.81126708126e-39 |
| 324.25421530479895 | 2.06136479819e-24 | 324.25421530479895 | 2.16875556973e-08 | 324.25421530479895 | 9.80929498697e-40 |
| 323.65951019527455 | 1.26250812661e-24 | 323.65951019527455 | 1.57731650055e-08 | 323.65951019527455 | 5.30188628416e-40 |
| 323.0669825543829 | 7.7170412594e-25 | 323.0669825543829 | 1.14489167829e-08 | 323.0669825543829 | 2.85996247842e-40 |
| 322.4766204450177 | 4.70765659361e-25 | 322.4766204450177 | 8.29367979251e-09 | 322.4766204450177 | 1.53966974237e-40 |
| 321.88841201716735 | 2.86613102993e-25 | 321.88841201716735 | 5.9960803235e-09 | 321.88841201716735 | 8.27241146344e-41 |
| 321.3023455071222 | 1.74150457563e-25 | 321.3023455071222 | 4.32638269922e-09 | 321.3023455071222 | 4.43582080381e-41 |
| 320.71840923669015 | 1.05606466486e-25 | 320.71840923669015 | 3.11544248075e-09 | 320.71840923669015 | 2.37384944768e-41 |
| 320.1365916124213 | 6.39136641903e-26 | 320.1365916124213 | 2.23898845742e-09 | 320.1365916124213 | 1.26785522165e-41 |
| 319.5568811248402 | 3.86041713191e-26 | 319.5568811248402 | 1.60591030492e-09 | 319.5568811248402 | 6.75808217344e-42 |
| 318.97926634768737 | 2.32708402617e-26 | 318.97926634768737 | 1.14955042985e-09 | 318.97926634768737 | 3.5951298892e-42 |
| 318.40373593716834 | 1.39999733438e-26 | 318.40373593716834 | 8.2124376324e-10 | 318.40373593716834 | 1.90872348112e-42 |
| 317.8302786312109 | 8.40581186272e-27 | 317.8302786312109 | 5.85535800591e-10 | 317.8302786312109 | 1.01136695535e-42 |
| 317.2588832487309 | 5.03697082277e-27 | 317.2588832487309 | 4.16650719304e-10 | 317.2588832487309 | 5.34825143257e-43 |
| 316.6895386889053 | 3.01228818113e-27 | 316.6895386889053 | 2.95888530638e-10 | 316.6895386889053 | 2.82261851261e-43 |
| 316.1222339304531 | 1.79788087844e-27 | 316.1222339304531 | 2.09711110212e-10 | 316.1222339304531 | 1.48672234228e-43 |
| 315.55695803092453 | 1.07093379114e-27 | 315.55695803092453 | 1.48337877516e-10 | 315.55695803092453 | 7.8152857795e-44 |
| 314.99370012599746 | 6.36651319685e-28 | 314.99370012599746 | 1.04717675883e-10 | 314.99370012599746 | 4.10012570357e-44 |
| 314.432449428781 | 3.77726955475e-28 | 314.432449428781 | 7.37777220818e-11 | 314.432449428781 | 2.14677630463e-44 |
| 313.8731952291274 | 2.23661702154e-28 | 313.8731952291274 | 5.1876157755e-11 | 313.8731952291274 | 1.12179558733e-44 |
| 313.31592689295036 | 1.32172950914e-28 | 313.31592689295036 | 3.64038801125e-11 | 313.31592689295036 | 5.85029781221e-45 |
| 312.76063386155124 | 7.79526457093e-29 | 312.76063386155124 | 2.549557797e-11 | 312.76063386155124 | 3.04494557742e-45 |
| 312.2073056509522 | 4.58834902404e-29 | 312.2073056509522 | 1.78204784842e-11 | 312.2073056509522 | 1.58167921687e-45 |
| 311.65593185123623 | 2.69537592898e-29 | 311.65593185123623 | 1.2431146165e-11 | 311.65593185123623 | 8.19963639157e-46 |
| 311.1065021258944 | 1.58022729124e-29 | 311.1065021258944 | 8.65446643694e-12 | 311.1065021258944 | 4.2423656083e-46 |
| 310.5590062111801 | 9.24606800507e-30 | 310.5590062111801 | 6.01321505502e-12 | 310.5590062111801 | 2.19057892719e-46 |
| 310.01343391546965 | 5.39923116212e-30 | 310.01343391546965 | 4.16975451101e-12 | 310.01343391546965 | 1.12887805519e-46 |
| 309.4697751186301 | 3.14661836477e-30 | 309.4697751186301 | 2.88570247041e-12 | 309.4697751186301 | 5.8059390017e-47 |
| 308.9280197713932 | 1.83017887147e-30 | 308.9280197713932 | 1.99310385104e-12 | 308.9280197713932 | 2.98013007135e-47 |
| 308.3881578947368 | 1.06238104789e-30 | 308.3881578947368 | 1.37386993031e-12 | 308.3881578947368 | 1.52663524227e-47 |
| 307.8501795792714 | 6.1546647971e-31 | 307.8501795792714 | 9.45145391978e-13 | 307.8501795792714 | 7.80499556855e-48 |
| 307.31407498463426 | 3.55849047793e-31 | 307.31407498463426 | 6.48916679531e-13 | 307.31407498463426 | 3.9824226635e-48 |
| 306.77983433888943 | 2.05335728914e-31 | 306.77983433888943 | 4.44648228295e-13 | 306.77983433888943 | 2.02795985583e-48 |
| 306.2474479379338 | 1.18249837718e-31 | 306.2474479379338 | 3.04075555026e-13 | 306.2474479379338 | 1.03064397708e-48 |
| 305.7169061449098 | 6.79632119562e-32 | 305.7169061449098 | 2.07531374773e-13 | 305.7169061449098 | 5.22751513092e-49 |
| 305.1881993896236 | 3.89838341407e-32 | 305.1881993896236 | 1.41358956604e-13 | 305.1881993896236 | 2.6461791331e-49 |
| 304.6613181679699 | 2.23168306964e-32 | 304.6613181679699 | 9.60948718073e-14 | 304.6613181679699 | 1.33684327369e-49 |
| 304.1362530413625 | 1.27502235631e-32 | 304.1362530413625 | 6.51950174015e-14 | 304.1362530413625 | 6.74029746e-50 |
| 303.61299463617036 | 7.27010015404e-33 | 303.61299463617036 | 4.41434134342e-14 | 303.61299463617036 | 3.391680573e-50 |
| 303.09153364316023 | 4.13714079284e-33 | 303.09153364316023 | 2.98301015733e-14 | 303.09153364316023 | 1.70328819949e-50 |
| 302.571860816944 | 2.34961940777e-33 | 302.571860816944 | 2.01178175377e-14 | 302.571860816944 | 8.53686953462e-51 |
| 302.0539669754329 | 1.3317786665e-33 | 302.0539669754329 | 1.35407995982e-14 | 302.0539669754329 | 4.27018277178e-51 |
| 301.5378429992964 | 7.53362328364e-34 | 301.5378429992964 | 9.09588727142e-15 | 301.5378429992964 | 2.131727053e-51 |
| 301.02347983142687 | 4.25317310114e-34 | 301.02347983142687 | 6.09793991807e-15 | 301.02347983142687 | 1.06207220278e-51 |
| 300.5108684764098 | 2.3964012112e-34 | 300.5108684764098 | 4.07998461726e-15 | 300.5108684764098 | 5.28097122351e-52 |

| **GON2-Al6** | |
| --- | --- |
| Wavelength (nm) | Abs |
| 2000.0 | 3.43141839154 |
| 1977.5873434410018 | 3.96601294854 |
| 1955.671447196871 | 4.57638605391 |
| 1934.2359767891683 | 5.27205943296 |
| 1913.265306122449 | 6.0635658483 |
| 1892.7444794952683 | 6.96253013275 |
| 1872.6591760299625 | 7.9817531675 |
| 1852.9956763434218 | 9.13529841071 |
| 1833.7408312958437 | 10.4385804972 |
| 1814.8820326678765 | 11.908455339 |
| 1796.4071856287424 | 13.5633110626 |
| 1778.3046828689983 | 15.4231590163 |
| 1760.5633802816901 | 17.509723983 |
| 1743.1725740848342 | 19.8465326224 |
| 1726.1219792865363 | 22.4589990643 |
| 1709.4017094017095 | 25.374506468 |
| 1693.002257336343 | 28.6224832531 |
| 1676.9144773616547 | 32.2344726137 |
| 1661.1295681063123 | 36.2441938218 |
| 1645.6390565002741 | 40.6875937443 |
| 1630.4347826086955 | 45.6028869116 |
| 1615.5088852988692 | 51.0305824089 |
| 1600.8537886872998 | 57.0134958023 |
| 1586.4621893178212 | 63.5967442739 |
| 1572.3270440251572 | 70.827723111 |
| 1558.4415584415583 | 78.7560616937 |
| 1544.799176107106 | 87.433557141 |
| 1531.3935681470139 | 96.9140838137 |
| 1518.2186234817814 | 107.25347694 |
| 1505.2684395383842 | 118.509388721 |
| 1492.5373134328358 | 130.741115387 |
| 1480.0197335964478 | 144.009393839 |
| 1467.7103718199608 | 158.376166667 |
| 1455.604075691412 | 173.904314563 |
| 1443.6958614051973 | 190.657355374 |
| 1431.9809069212408 | 208.699109317 |
| 1420.4545454545455 | 228.093330168 |
| 1409.1122592766555 | 248.903302566 |
| 1397.9496738117427 | 271.191405924 |
| 1386.9625520110958 | 295.018645829 |
| 1376.1467889908256 | 320.44415418 |
| 1365.4984069185252 | 347.524659776 |
| 1355.0135501355014 | 376.313931465 |
| 1344.688480502017 | 406.862196434 |
| 1334.5195729537365 | 439.215536673 |
| 1324.5033112582782 | 473.4152671 |
| 1314.6362839614374 | 509.497299318 |
| 1304.9151805132665 | 547.4914954 |
| 1295.3367875647668 | 587.421016582 |
| 1285.8979854264894 | 629.301672134 |
| 1276.5957446808509 | 673.14127413 |
| 1267.427122940431 | 718.939004172 |
| 1258.3892617449665 | 766.684798514 |
| 1249.4793835901708 | 816.358758293 |
| 1240.6947890818858 | 867.93059186 |
| 1232.0328542094455 | 921.359096384 |
| 1223.4910277324632 | 976.591686048 |
| 1215.0668286755772 | 1033.56397422 |
| 1206.7578439259853 | 1092.19941701 |
| 1198.5617259288852 | 1152.40902543 |
| 1190.4761904761904 | 1214.09115337 |
| 1182.4990145841543 | 1277.13136819 |
| 1174.6280344557556 | 1341.4024104 |
| 1166.8611435239206 | 1406.76424853 |
| 1159.19629057187 | 1473.06423454 |
| 1151.6314779270633 | 1540.13736463 |
| 1144.1647597254005 | 1607.80664954 |
| 1136.794240242516 | 1675.88359719 |
| 1129.5180722891566 | 1744.16881024 |
| 1122.334455667789 | 1812.45269927 |
| 1115.2416356877322 | 1880.51631167 |
| 1108.2379017362393 | 1948.13227505 |
| 1101.3215859030836 | 2015.06585221 |
| 1094.4910616563297 | 2081.0761041 |
| 1087.7447425670775 | 2145.91715503 |
| 1081.081081081081 | 2209.33955358 |
| 1074.4985673352435 | 2271.09172083 |
| 1067.995728017088 | 2330.92147635 |
| 1061.5711252653928 | 2388.57763096 |
| 1055.2233556102708 | 2443.81163388 |
| 1048.951048951049 | 2496.3792609 |
| 1042.752867570386 | 2546.04232873 |
| 1036.6275051831374 | 2592.57042015 |
| 1030.5736860185502 | 2635.74260344 |
| 1024.5901639344263 | 2675.34912911 |
| 1018.6757215619693 | 2711.19308633 |
| 1012.829169480081 | 2743.09200154 |
| 1007.0493454179255 | 2770.87936144 |
| 1001.3351134846461 | 2794.40604298 |
| 995.6853634251576 | 2813.54163341 |
| 990.0990099009902 | 2828.1756242 |
| 984.5749917952082 | 2838.21846371 |
| 979.1122715404699 | 2843.60245474 |
| 973.7098344693281 | 2844.28248437 |
| 968.3666881859263 | 2840.23657561 |
| 963.0818619582664 | 2831.46625183 |
| 957.8544061302682 | 2817.9967074 |
| 952.6833915528738 | 2799.87677988 |
| 947.5679090334806 | 2777.17872177 |
| 942.5070688030161 | 2749.99777178 |
| 937.4999999999999 | 2718.45152864 |
| 932.5458501709667 | 2682.67913225 |
| 927.643784786642 | 2642.84025998 |
| 922.7929867733004 | 2599.1139478 |
| 917.9926560587514 | 2551.69724851 |
| 913.2420091324201 | 2500.80374097 |
| 908.5402786190186 | 2446.66190657 |
| 903.8867128653209 | 2389.51339042 |
| 899.2805755395683 | 2329.61116625 |
| 894.7211452430658 | 2267.21762521 |
| 890.2077151335311 | 2202.60260951 |
| 885.7395925597874 | 2136.04141215 |
| 881.316098707403 | 2067.81276453 |
| 876.9365682548962 | 1998.19683335 |
| 872.6003490401396 | 1927.47324777 |
| 868.3068017366135 | 1855.91917724 |
| 864.0552995391705 | 1783.80747923 |
| 859.8452278589854 | 1711.40493476 |
| 855.6759840273816 | 1638.97058837 |
| 851.5469770082316 | 1566.75420717 |
| 847.457627118644 | 1494.9948718 |
| 843.4073657576608 | 1423.91971019 |
| 839.3956351426972 | 1353.74278269 |
| 835.421888053467 | 1284.66412518 |
| 831.4855875831485 | 1216.86895433 |
| 827.5862068965516 | 1150.52703704 |
| 823.7232289950576 | 1085.79222407 |
| 819.8961464881114 | 1022.8021456 |
| 816.1044613710554 | 961.67806471 |
| 812.3476848090983 | 902.524882661 |
| 808.6253369272237 | 845.431288686 |
| 804.9369466058491 | 790.470045048 |
| 801.2820512820513 | 737.698397212 |
| 797.6601967561818 | 687.158597809 |
| 794.0709370037056 | 638.878532268 |
| 790.5138339920949 | 592.872433409 |
| 786.9884575026232 | 549.141671907 |
| 783.4943849569078 | 507.675609389 |
| 780.0312012480499 | 468.452500944 |
| 776.598498576236 | 431.440434067 |
| 773.1958762886597 | 396.598291481 |
| 769.8229407236336 | 363.87672581 |
| 766.4793050587633 | 333.219134791 |
| 763.1645891630628 | 304.562626533 |
| 759.8784194528876 | 277.83896522 |
| 756.6204287515762 | 252.97548864 |
| 753.390256152687 | 229.895989966 |
| 750.1875468867216 | 208.52155724 |
| 747.011952191235 | 188.771365118 |
| 743.86312918423 | 170.563414465 |
| 740.7407407407408 | 153.815216435 |
| 737.6444553725104 | 138.444418666 |
| 734.5739471106758 | 124.369372153 |
| 731.528895391368 | 111.509638251 |
| 728.5089849441475 | 99.7864360582 |
| 725.5139056831922 | 89.1230311443 |
| 722.543352601156 | 79.4450672614 |
| 719.5970256656271 | 70.6808431966 |
| 716.6746297181079 | 62.7615374183 |
| 713.7758743754462 | 55.6213835433 |
| 710.9004739336492 | 49.197799955 |
| 708.0481472740146 | 43.4314771225 |
| 705.2186177715091 | 38.2664263191 |
| 702.4116132053383 | 33.6499935141 |
| 699.6268656716418 | 29.5328422269 |
| 696.8641114982578 | 25.8689090924 |
| 694.1230911614992 | 22.6153357924 |
| 691.4035492048858 | 19.732380878 |
| 688.7052341597796 | 17.1833148388 |
| 686.027898467871 | 14.9343015796 |
| 683.371298405467 | 12.954269246 |
| 680.7351940095303 | 11.2147731101 |
| 678.1193490054249 | 9.68985298238 |
| 675.5235307363206 | 8.35588737054 |
| 672.9475100942127 | 7.19144635741 |
| 670.3910614525139 | 6.17714492773 |
| 667.8539626001781 | 5.29549823749 |
| 665.335994677312 | 4.53078009372 |
| 662.8369421122403 | 3.86888569996 |
| 660.3565925599823 | 3.29719952311 |
| 657.8947368421053 | 2.8044689545 |
| 655.4511688879178 | 2.38068427049 |
| 653.0256856769699 | 2.01696524735 |
| 650.6180871828237 | 1.70545465156 |
| 648.2281763180639 | 1.43921870872 |
| 645.8557588805166 | 1.21215455326 |
| 643.5006435006435 | 1.01890457413 |
| 641.1626415900834 | 0.854777499874 |
| 638.8415672913118 | 0.715676007377 |
| 636.5372374283895 | 0.598030591901 |
| 634.2494714587738 | 0.498739400175 |
| 631.9780914261638 | 0.415113702529 |
| 629.7229219143577 | 0.344828662801 |
| 627.4837900020916 | 0.285879055173 |
| 625.2605252188412 | 0.236539573995 |
| 623.0529595015576 | 0.195329385046 |
| 620.8609271523178 | 0.160980573634 |
| 618.6842647968654 | 0.132410155535 |
| 616.5228113440197 | 0.108695330271 |
| 614.3764079459348 | 0.0890516719299 |
| 612.2448979591836 | 0.0728139699278 |
| 610.1281269066504 | 0.0594194503841 |
| 608.0259424402108 | 0.0483931275734 |
| 605.9381943041809 | 0.0393350538566 |
| 603.864734299517 | 0.0319092552714 |
| 601.8054162487462 | 0.0258341583016 |
| 599.7600959616153 | 0.0208743310306 |
| 597.7286312014345 | 0.0168333787696 |
| 595.7108816521048 | 0.0135478502151 |
| 593.7067088858104 | 0.0108820251484 |
| 591.7159763313609 | 0.00872346860992 |
| 589.7385492431688 | 0.00697924932881 |
| 587.7742946708463 | 0.00557273198069 |
| 585.8230814294083 | 0.00444086359259 |
| 583.8847800700661 | 0.00353188415926 |
| 581.9592628516003 | 0.00280340031767 |
| 580.046403712297 | 0.00222076880437 |
| 578.1460782424359 | 0.00175574344989 |
| 576.2581636573184 | 0.00138534570781 |
| 574.3825387708214 | 0.00109092423604 |
| 572.5190839694656 | 0.000857373906645 |
| 570.6676811869887 | 0.00067248887925 |
| 568.8282138794084 | 0.000526428090478 |
| 567.000567000567 | 0.000411274743877 |
| 565.1846269781461 | 0.000320674183835 |
| 563.3802816901408 | 0.000249536951911 |
| 561.5874204417821 | 0.000193795899815 |
| 559.8059339428997 | 0.000150208011209 |
| 558.0357142857143 | 0.000116193101815 |
| 556.2766549230483 | 8.97028578791e-05 |
| 554.52865064695 | 6.91147668119e-05 |
| 552.791597567717 | 5.31464179452e-05 |
| 551.0653930933137 | 4.07864293255e-05 |
| 549.3499359091741 | 3.12389095012e-05 |
| 547.645125958379 | 2.38789095336e-05 |
| 545.950864422202 | 1.82167760658e-05 |
| 544.2670537010159 | 1.38696950555e-05 |
| 542.5935973955508 | 1.05390297311e-05 |
| 540.9304002884962 | 7.9923157493e-06 |
| 539.2773683264425 | 6.04899027564e-06 |
| 537.6344086021505 | 4.56910727257e-06 |
| 536.0014293371448 | 3.44443507992e-06 |
| 534.3783398646241 | 2.59144980663e-06 |
| 532.7650506126798 | 1.94583368179e-06 |
| 531.1614730878186 | 1.45816516536e-06 |
| 529.5675198587819 | 1.09055049604e-06 |
| 527.9831045406547 | 8.13997124287e-07 |
| 526.4081417792595 | 6.06370360963e-07 |
| 524.8425472358292 | 4.50807400853e-07 |
| 523.2862375719518 | 3.34489170935e-07 |
| 521.7391304347826 | 2.47691450129e-07 |
| 520.2011444425177 | 1.83053431785e-07 |
| 518.6721991701245 | 1.35015185736e-07 |
| 517.1522151353215 | 9.93860026396e-08 |
| 515.6411137848057 | 7.30139207659e-08 |
| 514.1388174807198 | 5.35332905117e-08 |
| 512.6452494873547 | 3.91723846371e-08 |
| 511.1603339580848 | 2.86071021043e-08 |
| 509.683995922528 | 2.08499726035e-08 |
| 508.2161612739285 | 1.51661330277e-08 |
| 506.7567567567567 | 1.10098643156e-08 |
| 505.3057099545225 | 7.97676486351e-09 |
| 503.8629492777964 | 5.76778835934e-09 |
| 502.4284039524367 | 4.16226311159e-09 |
| 501.00200400801606 | 2.99769482224e-09 |
| 499.5836802664446 | 2.15468091961e-09 |
| 498.1733643307871 | 1.5456678059e-09 |
| 496.7709885742673 | 1.10659063755e-09 |
| 495.3764861294584 | 7.90670334211e-10 |
| 493.98979087765514 | 5.63821338933e-10 |
| 492.61083743842363 | 4.01259356807e-10 |
| 491.2395611593253 | 2.85001027652e-10 |
| 489.8758981058131 | 2.02025066395e-10 |
| 488.5197850512946 | 1.42922847187e-10 |
| 487.17115946735953 | 1.00910328432e-10 |
| 485.82995951416996 | 7.11061443529e-11 |
| 484.49612403100775 | 5.00053166683e-11 |
| 483.16959252697694 | 3.50964157643e-11 |
| 481.8503051718599 | 2.45836789149e-11 |
| 480.5382027871216 | 1.71857504128e-11 |
| 479.23322683706067 | 1.19902333264e-11 |
| 477.9353194201051 | 8.34880408842e-12 |
| 476.64442326024783 | 5.80174184171e-12 |
| 475.3604816986214 | 4.02374099752e-12 |
| 474.08343868520853 | 2.78508924976e-12 |
| 472.8132387706856 | 1.92391415832e-12 |
| 471.5498270983967 | 1.32638540109e-12 |
| 470.29314939645707 | 9.12622640342e-13 |
| 469.04315196998124 | 6.26686300791e-13 |
| 467.7997816934352 | 4.2948363068e-13 |
| 466.5629860031104 | 2.93751759802e-13 |
| 465.33271288971605 | 2.00517268528e-13 |
| 464.10891089108907 | 1.3660308887e-13 |
| 462.8915290850177 | 9.28766819486e-14 |
| 461.68051708217905 | 6.3021725738e-14 |
| 460.47582501918646 | 4.26787129951e-14 |
| 459.2774035517452 | 2.88449477714e-14 |
| 458.0852038479157 | 1.94565401532e-14 |
| 456.89917758148033 | 1.30978159625e-14 |
| 455.7192769254139 | 8.7997343329e-15 |
| 454.54545454545456 | 5.90034820586e-15 |
| 453.3776635937736 | 3.94841800884e-15 |
| 452.2158577027434 | 2.63697494622e-15 |
| 451.05999097880016 | 1.75762522757e-15 |
| 449.9100179964007 | 1.16918690521e-15 |
| 448.7658937920718 | 7.76209575848e-16 |
| 447.6275738585497 | 5.14293973469e-16 |
| 446.49501413900873 | 3.40080129352e-16 |
| 445.36817102137763 | 2.24433901521e-16 |
| 444.247001332741 | 1.47819945899e-16 |
| 443.13146233382565 | 9.71661497592e-17 |
| 442.02151171357 | 6.37432675238e-17 |
| 440.9171075837742 | 4.17340969454e-17 |
| 439.8182084738308 | 2.72699968429e-17 |
| 438.72477332553376 | 1.77834705971e-17 |
| 437.636761487965 | 1.15740490668e-17 |
| 436.5541327124563 | 7.5178123227e-18 |
| 435.4768471476266 | 4.87343301096e-18 |
| 434.4048653344918 | 3.1529412663e-18 |
| 433.3381482016467 | 2.03579534871e-18 |
| 432.2766570605187 | 1.31186670641e-18 |
| 431.22035360069 | 8.4368952445e-19 |
| 430.1691998852882 | 5.4151807292e-19 |
| 429.1231583464454 | 3.4688107407e-19 |
| 428.0821917808219 | 2.21761221131e-19 |
| 427.0462633451957 | 1.4149071838e-19 |
| 426.01533655211585 | 9.00964430372e-20 |
| 424.9893752656184 | 5.72564852764e-20 |
| 423.96834369700395 | 3.6314416378e-20 |
| 422.9522064006767 | 2.29863913138e-20 |
| 421.9409282700422 | 1.45211116487e-20 |
| 420.93447453346425 | 9.1551670771e-21 |
| 419.9328107502799 | 5.76063055954e-21 |
| 418.93590280687056 | 3.617521429e-21 |
| 417.94371691278906 | 2.2671984643e-21 |
| 416.9562195969423 | 1.41809492236e-21 |
| 415.97337770382694 | 8.85234642795e-22 |
| 414.99515838981876 | 5.51504214696e-22 |
| 414.0215291195142 | 3.42907200389e-22 |
| 413.0524576621231 | 2.12785341609e-22 |
| 412.08791208791206 | 1.31778373241e-22 |
| 411.1278607646978 | 8.14486535334e-23 |
| 410.17227235438884 | 5.02413161192e-23 |
| 409.22111580957574 | 3.09296807072e-23 |
| 408.2743603701687 | 1.90032199814e-23 |
| 407.33197556008145 | 1.16524241374e-23 |
| 406.39393118396094 | 7.13087299128e-24 |
| 405.46019732396263 | 4.35518338276e-24 |
| 404.53074433656957 | 2.65465152344e-24 |
| 403.6055428494551 | 1.61490105789e-24 |
| 402.68456375838923 | 9.80441408739e-25 |
| 401.76777822418643 | 5.94066000422e-25 |
| 400.85515766969536 | 3.59240320592e-25 |
| 399.9466737768297 | 2.16806739401e-25 |
| 399.0422984836393 | 1.30586356684e-25 |
| 398.14200398142003 | 7.84982828821e-26 |
| 397.24576271186436 | 4.70933767619e-26 |
| 396.3535473642489 | 2.81966056541e-26 |
| 395.46533087266016 | 1.68488844508e-26 |
| 394.5810864132579 | 1.00480733309e-26 |
| 393.7007874015748 | 5.9804210582e-27 |
| 392.82440748985204 | 3.55236882461e-27 |
| 391.9519205644107 | 2.10591898397e-27 |
| 391.08330074305826 | 1.24595566863e-27 |
| 390.2185223725286 | 7.35700145472e-28 |
| 389.3575600259571 | 4.33547228324e-28 |
| 388.5003885003885 | 2.54981872799e-28 |
| 387.6469828143171 | 1.49664748132e-28 |
| 386.7973182052604 | 8.76732390048e-29 |
| 385.95137012736393 | 5.12568488486e-29 |
| 385.1091142490372 | 2.99070842624e-29 |
| 384.2705264506212 | 1.74154044342e-29 |
| 383.4355828220859 | 1.01211619321e-29 |
| 382.6042596607575 | 5.8703568323e-30 |
| 381.77653346907607 | 3.39809836839e-30 |
| 380.95238095238096 | 1.96311034083e-30 |
| 380.1317790167258 | 1.13185499754e-30 |
| 379.31470476672143 | 6.51289673902e-31 |
| 378.5011355034065 | 3.74020072415e-31 |
| 377.69104872214524 | 2.14364539248e-31 |
| 376.88442211055275 | 1.226163254e-31 |
| 376.081233546446 | 6.9997248072e-32 |
| 375.28146109582184 | 3.98796145742e-32 |
| 374.48508301086 | 2.26755722182e-32 |
| 373.69207772795215 | 1.28677577116e-32 |
| 372.9024238657551 | 7.28760476413e-33 |
| 372.11610022326965 | 4.11911639041e-33 |
| 371.33308577794276 | 2.32359606552e-33 |
| 370.55335968379444 | 1.30814087877e-33 |
| 369.7769012695673 | 7.34997257025e-34 |
| 369.0036900369003 | 4.1214898551e-34 |
| 368.23370565852457 | 2.30653509951e-34 |
| 367.4669279764821 | 1.28825909189e-34 |
| 366.7033370003667 | 7.18097938733e-35 |
| 365.9429129055867 | 3.9948590385e-35 |
| 365.1856360316494 | 2.21797452843e-35 |
| 364.4314868804664 | 1.22899174122e-35 |
| 363.68044611468054 | 6.79639622176e-36 |
| 362.93249455601256 | 3.75098847909e-36 |
| 362.1876131836291 | 2.06609404405e-36 |
| 361.4457831325301 | 1.13577356484e-36 |
| 360.7069856919562 | 6.23118638107e-37 |
| 359.97120230381563 | 3.41182731516e-37 |
| 359.2384145611304 | 1.86440670797e-37 |
| 358.50860420650093 | 1.01679074952e-37 |
| 357.7817531305903 | 5.53426346974e-38 |
| 357.057843370626 | 3.00625204818e-38 |
| 356.33685710892024 | 1.62977729409e-38 |
| 355.6187766714082 | 8.81796662535e-39 |
| 354.9035845262037 | 4.76152372869e-39 |
| 354.1912632821723 | 2.56602432769e-39 |
| 353.48179568752204 | 1.38010745008e-39 |
| 352.77516462841015 | 7.40802339726e-40 |
| 352.07135312756714 | 3.96852485435e-40 |
| 351.3703443429374 | 2.12174495385e-40 |
| 350.6721215663355 | 1.13212548149e-40 |
| 349.9766682221185 | 6.02883318075e-41 |
| 349.2839678658749 | 3.20412376078e-41 |
| 348.59400418312805 | 1.69950564755e-41 |
| 347.90676098805517 | 8.99649319086e-42 |
| 347.2222222222222 | 4.7529277545e-42 |
| 346.54037195333257 | 2.50603126112e-42 |
| 345.8611943739912 | 1.31870930101e-42 |
| 345.1846738004832 | 6.92546546973e-43 |
| 344.5107946715664 | 3.62982912406e-43 |
| 343.8395415472779 | 1.89871904817e-43 |
| 343.17089910775564 | 9.91225685744e-44 |
| 342.50485215207215 | 5.16442174712e-44 |
| 341.84138559708293 | 2.68539495525e-44 |
| 341.1804844762879 | 1.3935801441e-44 |
| 340.522133938706 | 7.21760390196e-45 |
| 339.86631924776253 | 3.73070967e-45 |
| 339.2130257801899 | 1.92454103128e-45 |
| 338.56223902494077 | 9.90832424238e-46 |
| 337.91394458211306 | 5.09108747359e-46 |
| 337.2681281618887 | 2.61070752788e-46 |
| 336.6247755834829 | 1.33611302388e-46 |
| 335.9838727741068 | 6.82441590857e-47 |
| 335.3454057679409 | 3.47876498017e-47 |
| 334.709360705121 | 1.76979109569e-47 |
| 334.07572383073494 | 8.9857893903e-48 |
| 333.44448149383123 | 4.55331586356e-48 |
| 332.81562014643885 | 2.30269614955e-48 |
| 332.1891263425977 | 1.16220517395e-48 |
| 331.5649867374005 | 5.8541829109e-49 |
| 330.9431880860452 | 2.94297846398e-49 |
| 330.323717242898 | 1.47653992229e-49 |
| 329.70656116056705 | 7.39333894706e-50 |
| 329.0917068889864 | 3.69465042466e-50 |
| 328.47914157451 | 1.84265233923e-50 |
| 327.86885245901635 | 9.1717195328e-51 |
| 327.26082687902255 | 4.55612301914e-51 |
| 326.6550522648083 | 2.25879819116e-51 |
| 326.05151613955 | 1.11762661106e-51 |
| 325.4502061184639 | 5.51891048231e-52 |
| 324.8511099079588 | 2.71986456432e-52 |
| 324.25421530479895 | 1.33776097054e-52 |
| 323.65951019527455 | 6.56669849833e-53 |
| 323.0669825543829 | 3.2170139589e-53 |
| 322.4766204450177 | 1.57288194496e-53 |
| 321.88841201716735 | 7.6749688633e-54 |
| 321.3023455071222 | 3.7376138505e-54 |
| 320.71840923669015 | 1.81655918389e-54 |
| 320.1365916124213 | 8.81134054437e-55 |
| 319.5568811248402 | 4.26551755864e-55 |
| 318.97926634768737 | 2.06081399432e-55 |
| 318.40373593716834 | 9.93672266506e-56 |
| 317.8302786312109 | 4.78172786167e-56 |
| 317.2588832487309 | 2.29648628484e-56 |
| 316.6895386889053 | 1.10072839358e-56 |
| 316.1222339304531 | 5.26542942126e-57 |
| 315.55695803092453 | 2.51376534844e-57 |
| 314.99370012599746 | 1.19771361918e-57 |
| 314.432449428781 | 5.69532554828e-58 |
| 313.8731952291274 | 2.70284681442e-58 |
| 313.31592689295036 | 1.2801522515e-58 |
| 312.76063386155124 | 6.05116686924e-59 |
| 312.2073056509522 | 2.85465709311e-59 |
| 311.65593185123623 | 1.34402107443e-59 |
| 311.1065021258944 | 6.31532234755e-60 |
| 310.5590062111801 | 2.96157189522e-60 |
| 310.01343391546965 | 1.38607378711e-60 |
| 309.4697751186301 | 6.4742241852e-61 |
| 308.9280197713932 | 3.0180499986e-61 |
| 308.3881578947368 | 1.40411422687e-61 |
| 307.8501795792714 | 6.51952210717e-62 |
| 307.31407498463426 | 3.02110902271e-62 |
| 306.77983433888943 | 1.39718639021e-62 |
| 306.2474479379338 | 6.44881039634e-63 |
| 305.7169061449098 | 2.97058636306e-63 |
| 305.1881993896236 | 1.36565836996e-63 |
| 304.6613181679699 | 6.26583957803e-64 |
| 304.1362530413625 | 2.86915351178e-64 |
| 303.61299463617036 | 1.31118995158e-64 |
| 303.09153364316023 | 5.98018683016e-65 |
| 302.571860816944 | 2.72208200876e-65 |
| 302.0539669754329 | 1.23658783874e-65 |
| 301.5378429992964 | 5.60642543019e-66 |
| 301.02347983142687 | 2.53678956208e-66 |
| 300.5108684764098 | 1.14556608344e-66 |
